# Supplementary material for: Stereotypy is strongly linked to multiple biomarkers of oxidative stress—A potential common etiology for Abnormal Repetitive Behaviors
Source: PLoS One. 2025 Nov 5;20(11):e0326902. doi: 10.1371/journal.pone.0326902 (PMC12588453; doi:10.1371/journal.pone.0326902)
Supplement: S1 File — (PDF) [file pone.0326902.s001.pdf]

Table S1: q-values and P-values for Olink proteins correlated with GSH level in C57BL/6 Mice.

Protein	q-value	P-value
Tnni3	q = 0.0429	P = 0.0010
Dlk1	q = 0.0429	P = 0.0013
Yes1	q = 0.0429	P = 0.0014
Ddah1	q = 0.5750	P = 0.0025
Pdgfb	q = 0.1196	P = 0.0065
Prdx5	q = 0.1196	P = 0.0078
Parp1	q = 0.4733	P = 0.0390
Ghrl	q = 0.4733	P = 0.0414
Ahr	q = 0.4733	P = 0.4630

We used an REML mixed model blocked for individual differences to examine the relationship between protein expression and plasma GSH level in C57BL/6 mice. We found protein expression to correlate with plasma GSH level as a function of age. BH corrected q-values and associated P-values for the 9 proteins correlated with GSH level are presented here and ordered by descending BH corrected q-values and secondarily ordered by descending associated P-values.

Table S2: q-values and P-values for Olink proteins correlated with severity of stereotypy in C57BL/6 mice.

Protein	q-value	P-value
Tnni3	q < 0.0001	P < 0.0001
Ddah1	q < 0.0001	P < 0.0001
Riox2	q < 0.0001	P < 0.0001
Nadk	q = 0.0001	P < 0.0001
Plin1	q = 0.0003	P < 0.0001
Gcg	q = 0.0003	P < 0.0001
Qdpr	q = 0.0004	P < 0.0001
Yes1	q = 0.0040	P = 0.0004
Plxna4	q = 0.0198	P = 0.0021
Ahr	q = 0.0198	P = 0.0022
Parp1	q = 0.0956	P = 0.0115
Prdx5	q = 0.1195	P = 0.0156
Fst	q = 0.1195	P = 0.0169
Ccl20	q = 0.2452	P = 0.0373
Pdgfb	q = 0.2876	P = 0.0496

We used an REML GLM blocked for individual differences to examine the relationship between expression of 92 proteins and severity of stereotypy in C57BL/6 mice. We found a significant interaction between protein expression and stereotypy. Of the 92 proteins, expression of 15 was significantly correlated with severity of stereotypy. BH corrected q-values and P-values are

presented here and descending BH corrected q-values and secondarily ordered by descending associated P-values.

Table S3: q-values and P-values for Olink proteins correlated with GSH level in C57BL/6 mice, stratified by age.

Protein	Young Mice	Old Mice
Tnni3	q = 0.0076; P = 0.0021	q = 0.0931; P = 0.0569
Dlk1	q = 0.1143; P = 0.0762	q = 0.0185; P = 0.0072
Yes1	q = 0.0099; P < 0.0001	q = 0.4241; P = 0.3534
Ddah1	q = 0.0099; P < 0.0001	q = 0.5923; P = 0.5265
Pdgfb	q = 0.0896; P = 0.0498	q = 0.0896; P = 0.0487
Prdx5	q = 0.0059; P = 0.0013	q = 0.3753; P = 0.2894
Parp1	q = 0.0093; P = 0.0031	q = 0.6361; P = 0.6008
Ghrl	q = 0.0786; P = 0.0350	q = 0.3753; P = 0.2919
Ahr	q = 0.0012; P = 0.0002	q = 0.9694; P = 0.9694

Given the age interaction noted in the REML analysis of protein expression and GSH level, we examined the expression profile of the nine proteins and age group. BH corrected q-values and P-values for the young and old groups are presented here and ordered by descending BH corrected q-values and secondarily ordered by descending associated P-values.

Table S4: q-values and P-values for Olink proteins correlated with stereotypy severity in CD1 mice.

**Protein**	**q-value**	**P-value**
Pdgfb	q = 0.0149	P = 0.0002
Riox2	q = 0.1782	P = 0.0043
Parp1	q = 0.1782	P = 0.0058
Snap29	q = 0.3309	P = 0.0144
Vsig2	q = 0.4385	P = 0.0269
Map2k6	q = 0.4385	P = 0.0286
Cxcl9	q = 0.5017	P = 0.0500

We used an REML GLM controlled for individual differences to examine the relationship between expression of 92 proteins and stereotypy severity in CD1. Of the 92 proteins, expression of seven were significantly correlated with stereotypy severity. BH corrected q-values and associated P-values are presented here and ordered by descending BH corrected q-values and secondarily ordered by descending associated P-values.

Table S5: Mouse behavioral ethogram used for video scoring.

Behavior	Definition	Stereotypy
Inactive (IN)	Sleeping or wakeful but unmoving	No
Maintenance (MA)	Drinking, feeding, grooming, or nesting	No
General Activity (GE)	Locomotion or exploration	No
Bar Mouthing (BAR)	Biting or sliding mouth along feeder bars	Yes
Circling (CIR)	Tracing a loosely circular path while hanging from feeder bars or on cage floor	Yes
Climbing (CLI)	Climbing up on the feeder with all four limbs leaving the cage floor	Yes
Digging (DIG)	Scraping at nesting material, not followed by nesting or defecation	Yes
Jumping (JUM)	Jumping repeatedly (any more than 1x) toward the cage lid in an upright posture, with hindlimbs leaving the cage floor	Yes
Looping (LOO)	Planting forepaws on the cage wall, followed by a backflip and landing on the cage floor	Yes
Route Tracing (ROU)	Tracing the same route along the cage floor or lid repeated at least 3x during recording interval	Yes
Tail Carry (TAI)	Holding tail tip in mouth and turning in circles	Yes

Twirling (TWI)	Grabbing the feeder bars with forelimbs and twisting in tight circles. Hindlimbs may or may not leave cage floor	Yes
Wave (WAV)	Rearing against cage walls and moving the body in an arc from side to side	Yes

This ethogram includes definitions used to quantify home cage behavior of C57Bl/6 mice and CD1 mice. Behaviors include inactivity (e.g., sleeping), maintenance (e.g., grooming, eating), general activity (e.g., exploration), and stereotypies. See mousebehavior.org for detailed descriptions of behavior included in ethogram.
