# Supplementary material for: Stereotypy is strongly linked to multiple biomarkers of oxidative stress—A potential common etiology for Abnormal Repetitive Behaviors
Source: PLoS One. 2025 Nov 5;20(11):e0326902. doi: 10.1371/journal.pone.0326902 (PMC12588453; doi:10.1371/journal.pone.0326902)
Supplement: S2 File — (PDF) [file pone.0326902.s002.pdf]

Human Readable Data and Code for Coden et al.

Human readable data and analyses are provided as SAS code. The code is annotated to explain the implementation of our analysis plan, the particular options within SAS, and other information needed to implement these analyses in other software. For further reference see (Littell, Stroup et al. 2002, Kiernan, Tao et al. 2012)

GSH and Stereotypy

```
DATA GSH_and_Stereotypy_20241126;

/*See text for details on data processing*/

INPUT  Sex &$16. Strain &$16. Square_Root_SumAllST ln_total_min TwoGroups &$; Lines;
F  Vav-iCre  2.64575131106459  3.673020777  old
F  Vav-iCre  7.87400787401181  3.452670411  old
M  Vav-iCre  3.16227766016838  2.987933377  old
M  Vav-iCre  4.58257569495584  3.004426169  old
F  Vav-iCre  4.89897948556636  2.941781611  old
M  Vav-iCre  2  3.071586234  old
M  Vav-iCre  4.12310562561766  2.889891876  old
F  unknown  3.87298334620742  2.831567603  young
F  unknown  8.94427190999916  3.944260132  young
M  Vav-iCre  2.64575131106459  3.752831679  old
M  b6-129-micro36  5.56776436283002  4.284000526  young
F  b6-129-micro36  3  3.138551401  young
M  unknown  3  2.8910225  young
F  b6-129-micro36  4.79583152331272  3.60986079  young
M  b6-129-micro36  2.44948974278318  3.581540396  young
F  b6-129-micro36  6.48074069840786  3.176208089  old
M  b6-129-micro36  0  2.755697812  young
F  unknown  6.32455532033676  3.468953103  young
F  unknown  2.44948974278318  3.302837472  young
;
RUN;

/*This GLM tests whether Plasma GSH Level predicts stereotypy, and
whether that relationship is dependent on age. Because mice were
drawn from the general population, we include Sex and Strain as blocking factors
which controls for these effects statistically. This is best practice for
epidemiological designs of this kind. The SS3 option stipulates Type III SS -
i.e. all factors are tested controlled for all others in the model.
Transformations were identified in JMP.*/

PROC GLM  DATA=GSH_and_Stereotypy_20241126 ALPHA=0.05;
CLASS Sex Strain TwoGroups;
MODEL  Square_Root_SumAllST =
/*Blocking (control) factors */
Sex Strain

/*Risk (experimental) factors. Note that due to the
paramterization of GLM models in SAS, marginal terms to an
do not necessarily test something meaningful, and should be ignored.*/
ln_total_min TwoGroups ln_total_min*TwoGroups/ SS3;

/*Given the significant interaction, we can figure the
slope of each line and test against alpha = 0.05/2 = 0.025 */

ESTIMATE "Old Slope" ln_total_min 1 ln_total_min*TwoGroups 1 0;
ESTIMATE "Young Slope" ln_total_min 1 ln_total_min*TwoGroups 0 1;

RUN;
```

GSH and Proteomics

```
DATA Olink_And_GSH_20241125;

/*See text for details on data processing*/

INPUT  SubjectID &$ Log_Total_GSH TwoGroups &$16. Protein &$9. Norm_NPX; Lines;
StEt 01  3.67302077664802  old  Clmp  2.8683
StEt 01  3.67302077664802  old  Matn2  -0.28036
StEt 01  3.67302077664802  old  Cpe  2.25311
StEt 01  3.67302077664802  old  Gcg  -0.61261
StEt 01  3.67302077664802  old  Gdnf  -3.82092
StEt 01  3.67302077664802  old  Yes1  -2.12839
StEt 01  3.67302077664802  old  Il17a  -1.68211
StEt 01  3.67302077664802  old  Foxo1  -2.86781
StEt 01  3.67302077664802  old  Tnfrsf11b  0.48707
StEt 01  3.67302077664802  old  Tgfb1  -0.17296
StEt 01  3.67302077664802  old  Pla2g4a  1.66151
StEt 01  3.67302077664802  old  Il6  -3.95119
StEt 01  3.67302077664802  old  Prdx5  -1.08282
StEt 01  3.67302077664802  old  Tgfa  2.72297
StEt 01  3.67302077664802  old  Ccl5  -1.62222
StEt 01  3.67302077664802  old  Epo  -1.44117
StEt 01  3.67302077664802  old  Axin1  -2.66709
StEt 01  3.67302077664802  old  Fst  2.94974
StEt 01  3.67302077664802  old  Rgma  -0.65767
StEt 01  3.67302077664802  old  Nadk  2.90602
StEt 01  3.67302077664802  old  Tnni3  8.91236
StEt 01  3.67302077664802  old  Notch3  2.09404
StEt 01  3.67302077664802  old  Snap29  1.48582
StEt 01  3.67302077664802  old  Cntn1  5.34002
StEt 01  3.67302077664802  old  Clstn2  -2.4647
StEt 01  3.67302077664802  old  S100a4  6.0986
StEt 01  3.67302077664802  old  Ca13  -3.16719
StEt 01  3.67302077664802  old  Mia  1.26241
StEt 01  3.67302077664802  old  Cant1  -1.59288
StEt 01  3.67302077664802  old  Kitlg  -4.81995
StEt 01  3.67302077664802  old  Gfra1  -1.28563
StEt 01  3.67302077664802  old  Ppp1r2  -2.6189
StEt 01  3.67302077664802  old  Adam23  -1.14121
StEt 01  3.67302077664802  old  Itgb6  -2.55977
StEt 01  3.67302077664802  old  Cyr61  0.67965
StEt 01  3.67302077664802  old  Dlk1  3.93637
StEt 01  3.67302077664802  old  Ahr  -2.76967
StEt 01  3.67302077664802  old  Ccl2  6.35886
StEt 01  3.67302077664802  old  Eno2  -0.87518
StEt 01  3.67302077664802  old  Plin1  -2.62681
StEt 01  3.67302077664802  old  Wfikkn2  2.58723
StEt 01  3.67302077664802  old  Flrt2  0.00058
StEt 01  3.67302077664802  old  Qdpr  -0.31268
StEt 01  3.67302077664802  old  Fas  1.79292
StEt 01  3.67302077664802  old  Erbb4  0.87806
StEt 01  3.67302077664802  old  Riox2  -0.49543
StEt 01  3.67302077664802  old  Plxna4  -2.65013
StEt 01  3.67302077664802  old  Epcam  -1.07313
StEt 01  3.67302077664802  old  Ccl3  0.40848
StEt 01  3.67302077664802  old  Crim1  -1.35769
StEt 01  3.67302077664802  old  Vsig2  1.32577
StEt 01  3.67302077664802  old  Hgf  -1.14655
StEt 01  3.67302077664802  old  Sez6l2  2.63902
StEt 01  3.67302077664802  old  Il1a  0.07517
StEt 01  3.67302077664802  old  Il23r  4.62269
StEt 01  3.67302077664802  old  Dll1  1.41911
StEt 01  3.67302077664802  old  Ddah1  -2.07286
StEt 01  3.67302077664802  old  Il10  -1.96483
StEt 01  3.67302077664802  old  Tnfrsf12a  1.81618
StEt 01  3.67302077664802  old  Acvrl1  0.26476
StEt 01  3.67302077664802  old  Lgmn  1.47517
StEt 01  3.67302077664802  old  Csf2  -2.60837
StEt 01  3.67302077664802  old  Cxcl9  1.84353
StEt 01  3.67302077664802  old  Map2k6  1.0381
StEt 01  3.67302077664802  old  Itgb1bp2  -5.06866
StEt 01  3.67302077664802  old  Il17f  -4.80761
StEt 01  3.67302077664802  old  Il1b  -3.51375
StEt 01  3.67302077664802  old  Casp3  1.85158
StEt 01  3.67302077664802  old  Apbb1ip  -3.8027
StEt 01  3.67302077664802  old  Wisp1  0.90946
StEt 01  3.67302077664802  old  Cdh6  0.95205
StEt 01  3.67302077664802  old  Pdgfb  0.39275
StEt 01  3.67302077664802  old  Igsf3  -0.35396
StEt 01  3.67302077664802  old  Tgfbr3  0.53982
StEt 01  3.67302077664802  old  Cxcl1  5.75837
StEt 01  3.67302077664802  old  Pak4  -4.01841
StEt 01  3.67302077664802  old  Cntn4  1.20574
StEt 01  3.67302077664802  old  Ghrl  2.61238
StEt 01  3.67302077664802  old  Lpl  0.58407
StEt 01  3.67302077664802  old  Fstl3  1.58249
StEt 01  3.67302077664802  old  Dctn2  -1.71411
StEt 01  3.67302077664802  old  Il5  -3.35842
StEt 01  3.67302077664802  old  Eda2r  1.26479
StEt 01  3.67302077664802  old  Ntf3  -2.33484
StEt 01  3.67302077664802  old  Tnfsf12  0.25906
```

StEt 01	3.67302077664802	old	Ccl20	6.20999
StEt 01	3.67302077664802	old	Fli1	-4.30614
StEt 01	3.67302077664802	old	Tpp1	3.18126
StEt 01	3.67302077664802	old	Tnr	-0.39026
StEt 01	3.67302077664802	old	Vegfd	-0.58948
StEt 01	3.67302077664802	old	Parp1	-1.36957
StEt 01	3.67302077664802	old	Tnf	-3.28854
Stet 02	3.45267041102974	old	Clmp	3.05087
Stet 02	3.45267041102974	old	Matn2	0.63205
Stet 02	3.45267041102974	old	Cpe	2.12765
Stet 02	3.45267041102974	old	Gcg	-2.13358
Stet 02	3.45267041102974	old	Gdnf	-3.93587
Stet 02	3.45267041102974	old	Yes1	-1.23876
Stet 02	3.45267041102974	old	Il17a	-2.09183
Stet 02	3.45267041102974	old	Foxo1	-2.30232
Stet 02	3.45267041102974	old	Tnfrsf11b	0.62819
Stet 02	3.45267041102974	old	Tgfb1	-0.48833
Stet 02	3.45267041102974	old	Pla2g4a	2.56277
Stet 02	3.45267041102974	old	Il6	-4.19358
Stet 02	3.45267041102974	old	Prdx5	-0.71358
Stet 02	3.45267041102974	old	Tgfa	2.2597
Stet 02	3.45267041102974	old	Ccl5	-1.39874
Stet 02	3.45267041102974	old	Epo	0.17692
Stet 02	3.45267041102974	old	Axin1	-2.40962
Stet 02	3.45267041102974	old	Fst	2.64017
Stet 02	3.45267041102974	old	Rgma	-0.73759
Stet 02	3.45267041102974	old	Nadk	4.3223
Stet 02	3.45267041102974	old	Tnni3	9.30576
Stet 02	3.45267041102974	old	Notch3	1.73709
Stet 02	3.45267041102974	old	Snap29	2.49932
Stet 02	3.45267041102974	old	Cntn1	5.09719
Stet 02	3.45267041102974	old	Clstn2	-2.26892
Stet 02	3.45267041102974	old	S100a4	5.99458
Stet 02	3.45267041102974	old	Ca13	-3.0006
Stet 02	3.45267041102974	old	Mia	1.047
Stet 02	3.45267041102974	old	Cant1	-1.78886
Stet 02	3.45267041102974	old	Kitlg	-5.17391
Stet 02	3.45267041102974	old	Gfra1	-1.975
Stet 02	3.45267041102974	old	Ppp1r2	-2.11522
Stet 02	3.45267041102974	old	Adam23	-1.06003
Stet 02	3.45267041102974	old	Itgb6	-3.07225
Stet 02	3.45267041102974	old	Cyr61	0.47948
Stet 02	3.45267041102974	old	Dlk1	0.78281
Stet 02	3.45267041102974	old	Ahr	-1.74437
Stet 02	3.45267041102974	old	Ccl2	6.5617
Stet 02	3.45267041102974	old	Eno2	-0.9463
Stet 02	3.45267041102974	old	Plin1	-1.02285
Stet 02	3.45267041102974	old	Wfikkn2	1.76069
Stet 02	3.45267041102974	old	Flrt2	0.13895
Stet 02	3.45267041102974	old	Qdpr	1.17834
Stet 02	3.45267041102974	old	Fas	1.52895
Stet 02	3.45267041102974	old	Erbb4	0.90071
Stet 02	3.45267041102974	old	Riox2	1.03634
Stet 02	3.45267041102974	old	Plxna4	-2.94623
Stet 02	3.45267041102974	old	Epcam	-1.06176
Stet 02	3.45267041102974	old	Ccl3	-1.04699
Stet 02	3.45267041102974	old	Crim1	-1.84959
Stet 02	3.45267041102974	old	Vsig2	1.53819
Stet 02	3.45267041102974	old	Hgf	-1.07291
Stet 02	3.45267041102974	old	Sez6l2	2.2669
Stet 02	3.45267041102974	old	Il1a	0.21146
Stet 02	3.45267041102974	old	Il23r	4.09248
Stet 02	3.45267041102974	old	Dll1	1.30379
Stet 02	3.45267041102974	old	Ddah1	-0.42534
Stet 02	3.45267041102974	old	Il10	-1.98081
Stet 02	3.45267041102974	old	Tnfrsf12a	2.2169
Stet 02	3.45267041102974	old	Acvrl1	0.10168
Stet 02	3.45267041102974	old	Lgmn	1.97691
Stet 02	3.45267041102974	old	Csf2	-3.42544
Stet 02	3.45267041102974	old	Cxcl9	1.0041
Stet 02	3.45267041102974	old	Map2k6	1.35305
Stet 02	3.45267041102974	old	Itgb1bp2	-5.29071
Stet 02	3.45267041102974	old	Il17f	-5.16294
Stet 02	3.45267041102974	old	Il1b	-3.46715
Stet 02	3.45267041102974	old	Casp3	2.53604
Stet 02	3.45267041102974	old	Apbb1ip	-3.55452
Stet 02	3.45267041102974	old	Wisp1	0.88791
Stet 02	3.45267041102974	old	Cdh6	0.8346
Stet 02	3.45267041102974	old	Pdgfb	1.32723
Stet 02	3.45267041102974	old	Igsf3	-0.72057
Stet 02	3.45267041102974	old	Tgfbr3	0.42348
Stet 02	3.45267041102974	old	Cxcl1	4.37285
Stet 02	3.45267041102974	old	Pak4	-3.65676
Stet 02	3.45267041102974	old	Cntn4	0.48381
Stet 02	3.45267041102974	old	Ghrl	2.70304
Stet 02	3.45267041102974	old	Lpl	0.69613
Stet 02	3.45267041102974	old	Fstl3	1.71683
Stet 02	3.45267041102974	old	Dctn2	-1.51861
Stet 02	3.45267041102974	old	Il5	-3.15302
Stet 02	3.45267041102974	old	Eda2r	0.57831
Stet 02	3.45267041102974	old	Ntf3	-2.47061
Stet 02	3.45267041102974	old	Tnfsf12	-0.14764
Stet 02	3.45267041102974	old	Ccl20	4.48003
Stet 02	3.45267041102974	old	Fli1	-4.58615
Stet 02	3.45267041102974	old	Tpp1	3.27324

Stet	02	3.45267041102974	old	Tnr	0.02873
Stet	02	3.45267041102974	old	Vegfd	-0.73882
Stet	02	3.45267041102974	old	Parp1	-1.67648
Stet	02	3.45267041102974	old	Tnf	-3.09172
StEt	03	2.98793337756922	old	Clmp	3.34347
StEt	03	2.98793337756922	old	Matn2	0.66087
StEt	03	2.98793337756922	old	Cpe	2.25543
StEt	03	2.98793337756922	old	Gcg	0.45925
StEt	03	2.98793337756922	old	Gdnf	-3.90842
StEt	03	2.98793337756922	old	Yes1	-2.10034
StEt	03	2.98793337756922	old	Il17a	-1.19419
StEt	03	2.98793337756922	old	Foxo1	-3.13255
StEt	03	2.98793337756922	old	Tnfrsf11b	1.21489
StEt	03	2.98793337756922	old	Tgfb1	-0.11431
StEt	03	2.98793337756922	old	Pla2g4a	3.39979
StEt	03	2.98793337756922	old	Il6	-3.75407
StEt	03	2.98793337756922	old	Prdx5	-1.23139
StEt	03	2.98793337756922	old	Tgfa	1.84402
StEt	03	2.98793337756922	old	Ccl5	-1.61224
StEt	03	2.98793337756922	old	Epo	0.57383
StEt	03	2.98793337756922	old	Axin1	-2.77797
StEt	03	2.98793337756922	old	Fst	3.56543
StEt	03	2.98793337756922	old	Rgma	-0.81699
StEt	03	2.98793337756922	old	Nadk	1.16004
StEt	03	2.98793337756922	old	Tnni3	8.23016
StEt	03	2.98793337756922	old	Notch3	2.06501
StEt	03	2.98793337756922	old	Snap29	2.05091
StEt	03	2.98793337756922	old	Cntn1	5.41652
StEt	03	2.98793337756922	old	Clstn2	-2.1992
StEt	03	2.98793337756922	old	S100a4	5.73276
StEt	03	2.98793337756922	old	Ca13	-3.37403
StEt	03	2.98793337756922	old	Mia	1.16275
StEt	03	2.98793337756922	old	Cant1	-1.28717
StEt	03	2.98793337756922	old	Kitlg	-4.77739
StEt	03	2.98793337756922	old	Gfra1	-2.11539
StEt	03	2.98793337756922	old	Ppp1r2	-2.78485
StEt	03	2.98793337756922	old	Adam23	-0.7549
StEt	03	2.98793337756922	old	Itgb6	-2.15703
StEt	03	2.98793337756922	old	Cyr61	1.38393
StEt	03	2.98793337756922	old	Dlk1	0.61485
StEt	03	2.98793337756922	old	Ahr	-3.12868
StEt	03	2.98793337756922	old	Ccl2	5.81401
StEt	03	2.98793337756922	old	Eno2	-0.6037
StEt	03	2.98793337756922	old	Plin1	-2.88109
StEt	03	2.98793337756922	old	Wfikkn2	2.11634
StEt	03	2.98793337756922	old	Flrt2	0.29344
StEt	03	2.98793337756922	old	Qdpr	-1.57552
StEt	03	2.98793337756922	old	Fas	1.58556
StEt	03	2.98793337756922	old	Erbb4	1.3025
StEt	03	2.98793337756922	old	Riox2	-2.25249
StEt	03	2.98793337756922	old	Plxna4	0.00154
StEt	03	2.98793337756922	old	Epcam	-1.29581
StEt	03	2.98793337756922	old	Ccl3	-0.884
StEt	03	2.98793337756922	old	Crim1	-1.88958
StEt	03	2.98793337756922	old	Vsig2	1.59367
StEt	03	2.98793337756922	old	Hgf	-1.01478
StEt	03	2.98793337756922	old	Sez6l2	3.09585
StEt	03	2.98793337756922	old	Il1a	1.09365
StEt	03	2.98793337756922	old	Il23r	4.25662
StEt	03	2.98793337756922	old	Dll1	1.61314
StEt	03	2.98793337756922	old	Ddah1	-3.54132
StEt	03	2.98793337756922	old	Il10	-2.11933
StEt	03	2.98793337756922	old	Tnfrsf12a	2.02742
StEt	03	2.98793337756922	old	Acvrl1	0.44909
StEt	03	2.98793337756922	old	Lgmn	1.57327
StEt	03	2.98793337756922	old	Csf2	-2.99764
StEt	03	2.98793337756922	old	Cxcl9	1.24003
StEt	03	2.98793337756922	old	Map2k6	1.72816
StEt	03	2.98793337756922	old	Itgb1bp2	-5.40578
StEt	03	2.98793337756922	old	Il17f	-4.93621
StEt	03	2.98793337756922	old	Il1b	-3.30839
StEt	03	2.98793337756922	old	Casp3	2.11764
StEt	03	2.98793337756922	old	Apbb1ip	-3.53737
StEt	03	2.98793337756922	old	Wisp1	0.77798
StEt	03	2.98793337756922	old	Cdh6	1.08023
StEt	03	2.98793337756922	old	Pdgfb	1.63406
StEt	03	2.98793337756922	old	Igsf3	-0.30871
StEt	03	2.98793337756922	old	Tgfbr3	0.72284
StEt	03	2.98793337756922	old	Cxcl1	5.09483
StEt	03	2.98793337756922	old	Pak4	-3.81065
StEt	03	2.98793337756922	old	Cntn4	0.42184
StEt	03	2.98793337756922	old	Ghrl	2.06717
StEt	03	2.98793337756922	old	Lpl	0.64366
StEt	03	2.98793337756922	old	Fstl3	1.93628
StEt	03	2.98793337756922	old	Dctn2	-2.26418
StEt	03	2.98793337756922	old	Il5	-2.6995
StEt	03	2.98793337756922	old	Eda2r	0.8805
StEt	03	2.98793337756922	old	Ntf3	-1.13016
StEt	03	2.98793337756922	old	Tnfsf12	-0.20807
StEt	03	2.98793337756922	old	Ccl20	5.49602
StEt	03	2.98793337756922	old	Fli1	-4.54442
StEt	03	2.98793337756922	old	Tpp1	3.66258
StEt	03	2.98793337756922	old	Tnr	0.64117
StEt	03	2.98793337756922	old	Vegfd	-0.43825
StEt	03	2.98793337756922	old	Parp1	-2.07371

StEt 03	2.98793337756922	old	Tnf	-3.1531
StEt 04	3.00442616888787	old	Clmp	2.62312
StEt 04	3.00442616888787	old	Matn2	0.39493
StEt 04	3.00442616888787	old	Cpe	1.88616
StEt 04	3.00442616888787	old	Gcg	0.12832
StEt 04	3.00442616888787	old	Gdnf	-4.5977
StEt 04	3.00442616888787	old	Yes1	-0.05845
StEt 04	3.00442616888787	old	Il17a	-2.0778
StEt 04	3.00442616888787	old	Foxo1	-2.23253
StEt 04	3.00442616888787	old	Tnfrsf11b	-0.24401
StEt 04	3.00442616888787	old	Tgfb1	-0.28483
StEt 04	3.00442616888787	old	Pla2g4a	3.36659
StEt 04	3.00442616888787	old	Il6	-5.20267
StEt 04	3.00442616888787	old	Prdx5	0.64364
StEt 04	3.00442616888787	old	Tgfa	2.83293
StEt 04	3.00442616888787	old	Ccl5	-2.23972
StEt 04	3.00442616888787	old	Epo	-0.65893
StEt 04	3.00442616888787	old	Axin1	-1.58248
StEt 04	3.00442616888787	old	Fst	3.02473
StEt 04	3.00442616888787	old	Rgma	-0.89883
StEt 04	3.00442616888787	old	Nadk	3.75466
StEt 04	3.00442616888787	old	Tnni3	10.37774
StEt 04	3.00442616888787	old	Notch3	1.33509
StEt 04	3.00442616888787	old	Snap29	2.83475
StEt 04	3.00442616888787	old	Cntn1	4.48659
StEt 04	3.00442616888787	old	Clstn2	-3.0104
StEt 04	3.00442616888787	old	S100a4	5.53599
StEt 04	3.00442616888787	old	Ca13	-1.10455
StEt 04	3.00442616888787	old	Mia	0.40292
StEt 04	3.00442616888787	old	Cant1	-1.4367
StEt 04	3.00442616888787	old	Kitlg	-5.43481
StEt 04	3.00442616888787	old	Gfra1	-2.40971
StEt 04	3.00442616888787	old	Ppp1r2	-0.91027
StEt 04	3.00442616888787	old	Adam23	-1.64209
StEt 04	3.00442616888787	old	Itgb6	-3.6265
StEt 04	3.00442616888787	old	Cyr61	0.62093
StEt 04	3.00442616888787	old	Dlk1	-0.12399
StEt 04	3.00442616888787	old	Ahr	-2.26501
StEt 04	3.00442616888787	old	Ccl2	5.27498
StEt 04	3.00442616888787	old	Eno2	-0.86411
StEt 04	3.00442616888787	old	Plin1	1.17651
StEt 04	3.00442616888787	old	Wfikkn2	1.04952
StEt 04	3.00442616888787	old	Flrt2	1.8702
StEt 04	3.00442616888787	old	Qdpr	0.50989
StEt 04	3.00442616888787	old	Fas	1.38445
StEt 04	3.00442616888787	old	Erbb4	0.38031
StEt 04	3.00442616888787	old	Riox2	0.98442
StEt 04	3.00442616888787	old	Plxna4	-0.74947
StEt 04	3.00442616888787	old	Epcam	-1.70303
StEt 04	3.00442616888787	old	Ccl3	-1.06051
StEt 04	3.00442616888787	old	Crim1	-1.6311
StEt 04	3.00442616888787	old	Vsig2	1.24253
StEt 04	3.00442616888787	old	Hgf	0.4431
StEt 04	3.00442616888787	old	Sez6l2	2.15871
StEt 04	3.00442616888787	old	Il1a	0.6116
StEt 04	3.00442616888787	old	Il23r	3.27942
StEt 04	3.00442616888787	old	Dll1	0.62134
StEt 04	3.00442616888787	old	Ddah1	-2.20178
StEt 04	3.00442616888787	old	Il10	-2.84683
StEt 04	3.00442616888787	old	Tnfrsf12a	1.56805
StEt 04	3.00442616888787	old	Acvrl1	-0.03611
StEt 04	3.00442616888787	old	Lgmn	0.97395
StEt 04	3.00442616888787	old	Csf2	-3.79696
StEt 04	3.00442616888787	old	Cxcl9	0.98627
StEt 04	3.00442616888787	old	Map2k6	2.67686
StEt 04	3.00442616888787	old	Itgb1bp2	-6.2872
StEt 04	3.00442616888787	old	Il17f	-5.34008
StEt 04	3.00442616888787	old	Il1b	-4.29378
StEt 04	3.00442616888787	old	Casp3	2.67287
StEt 04	3.00442616888787	old	Apbb1ip	-3.12157
StEt 04	3.00442616888787	old	Wisp1	0.27909
StEt 04	3.00442616888787	old	Cdh6	0.17557
StEt 04	3.00442616888787	old	Pdgfb	5.80848
StEt 04	3.00442616888787	old	Igsf3	0.33948
StEt 04	3.00442616888787	old	Tgfbr3	-0.02983
StEt 04	3.00442616888787	old	Cxcl1	4.22814
StEt 04	3.00442616888787	old	Pak4	-3.78828
StEt 04	3.00442616888787	old	Cntn4	-0.37203
StEt 04	3.00442616888787	old	Ghrl	0.79972
StEt 04	3.00442616888787	old	Lpl	0.59936
StEt 04	3.00442616888787	old	Fstl3	1.37194
StEt 04	3.00442616888787	old	Dctn2	-1.06188
StEt 04	3.00442616888787	old	Il5	-4.40246
StEt 04	3.00442616888787	old	Eda2r	0.84394
StEt 04	3.00442616888787	old	Ntf3	-2.70778
StEt 04	3.00442616888787	old	Tnfsf12	0.20823
StEt 04	3.00442616888787	old	Ccl20	5.07031
StEt 04	3.00442616888787	old	Fli1	-3.78194
StEt 04	3.00442616888787	old	Tpp1	3.10541
StEt 04	3.00442616888787	old	Tnr	0.03941
StEt 04	3.00442616888787	old	Vegfd	-1.1949
StEt 04	3.00442616888787	old	Parp1	0.61051
StEt 04	3.00442616888787	old	Tnf	-4.28027
StEt 05	2.94178161096271	old	Clmp	3.3045
StEt 05	2.94178161096271	old	Matn2	0.77116

StEt 05	2.94178161096271	old	Cpe	2.42883
StEt 05	2.94178161096271	old	Gcg	-1.46561
StEt 05	2.94178161096271	old	Gdnf	-3.86328
StEt 05	2.94178161096271	old	Yes1	-1.56228
StEt 05	2.94178161096271	old	Il17a	-3.06094
StEt 05	2.94178161096271	old	Foxo1	-2.90161
StEt 05	2.94178161096271	old	Tnfrsf11b	0.65218
StEt 05	2.94178161096271	old	Tgfb1	-0.27251
StEt 05	2.94178161096271	old	Pla2g4a	2.41254
StEt 05	2.94178161096271	old	Il6	-4.12924
StEt 05	2.94178161096271	old	Prdx5	-0.96324
StEt 05	2.94178161096271	old	Tgfa	2.14297
StEt 05	2.94178161096271	old	Ccl5	-1.78556
StEt 05	2.94178161096271	old	Epo	-0.40537
StEt 05	2.94178161096271	old	Axin1	-2.61966
StEt 05	2.94178161096271	old	Fst	3.12116
StEt 05	2.94178161096271	old	Rgma	-0.75991
StEt 05	2.94178161096271	old	Nadk	3.39793
StEt 05	2.94178161096271	old	Tnni3	7.86763
StEt 05	2.94178161096271	old	Notch3	1.8953
StEt 05	2.94178161096271	old	Snap29	1.55437
StEt 05	2.94178161096271	old	Cntn1	5.30482
StEt 05	2.94178161096271	old	Clstn2	-2.00014
StEt 05	2.94178161096271	old	S100a4	6.11828
StEt 05	2.94178161096271	old	Ca13	-3.43998
StEt 05	2.94178161096271	old	Mia	1.03481
StEt 05	2.94178161096271	old	Cant1	-1.58247
StEt 05	2.94178161096271	old	Kitlg	-4.6022
StEt 05	2.94178161096271	old	Gfra1	-1.65775
StEt 05	2.94178161096271	old	Ppp1r2	-2.67695
StEt 05	2.94178161096271	old	Adam23	-1.02032
StEt 05	2.94178161096271	old	Itgb6	-2.88912
StEt 05	2.94178161096271	old	Cyr61	0.88375
StEt 05	2.94178161096271	old	Dlk1	1.63434
StEt 05	2.94178161096271	old	Ahr	-2.44108
StEt 05	2.94178161096271	old	Ccl2	6.03572
StEt 05	2.94178161096271	old	Eno2	-0.73855
StEt 05	2.94178161096271	old	Plin1	-2.90675
StEt 05	2.94178161096271	old	Wfikkn2	1.99514
StEt 05	2.94178161096271	old	Flrt2	0.5159
StEt 05	2.94178161096271	old	Qdpr	0.49186
StEt 05	2.94178161096271	old	Fas	1.43293
StEt 05	2.94178161096271	old	Erbb4	0.6029
StEt 05	2.94178161096271	old	Riox2	0.14015
StEt 05	2.94178161096271	old	Plxna4	-1.66065
StEt 05	2.94178161096271	old	Epcam	-1.0913
StEt 05	2.94178161096271	old	Ccl3	-1.29577
StEt 05	2.94178161096271	old	Crim1	-2.02988
StEt 05	2.94178161096271	old	Vsig2	1.74725
StEt 05	2.94178161096271	old	Hgf	-1.08114
StEt 05	2.94178161096271	old	Sez6l2	2.59687
StEt 05	2.94178161096271	old	Il1a	0.30258
StEt 05	2.94178161096271	old	Il23r	4.20982
StEt 05	2.94178161096271	old	Dll1	1.1947
StEt 05	2.94178161096271	old	Ddah1	-1.18427
StEt 05	2.94178161096271	old	Il10	-2.34791
StEt 05	2.94178161096271	old	Tnfrsf12a	1.83752
StEt 05	2.94178161096271	old	Acvrl1	0.04461
StEt 05	2.94178161096271	old	Lgmn	1.48683
StEt 05	2.94178161096271	old	Csf2	-2.67794
StEt 05	2.94178161096271	old	Cxcl9	1.71215
StEt 05	2.94178161096271	old	Map2k6	1.18481
StEt 05	2.94178161096271	old	Itgb1bp2	-5.0158
StEt 05	2.94178161096271	old	Il17f	-4.89924
StEt 05	2.94178161096271	old	Il1b	-3.30016
StEt 05	2.94178161096271	old	Casp3	2.18461
StEt 05	2.94178161096271	old	Apbb1ip	-3.84749
StEt 05	2.94178161096271	old	Wisp1	0.80265
StEt 05	2.94178161096271	old	Cdh6	1.24904
StEt 05	2.94178161096271	old	Pdgfb	0.63228
StEt 05	2.94178161096271	old	Igsf3	-0.29162
StEt 05	2.94178161096271	old	Tgfbr3	0.49581
StEt 05	2.94178161096271	old	Cxcl1	5.12701
StEt 05	2.94178161096271	old	Pak4	-4.32047
StEt 05	2.94178161096271	old	Cntn4	1.00806
StEt 05	2.94178161096271	old	Ghrl	2.24656
StEt 05	2.94178161096271	old	Lpl	1.07018
StEt 05	2.94178161096271	old	Fstl3	1.61288
StEt 05	2.94178161096271	old	Dctn2	-1.86107
StEt 05	2.94178161096271	old	Il5	-0.77023
StEt 05	2.94178161096271	old	Eda2r	0.85302
StEt 05	2.94178161096271	old	Ntf3	-2.4723
StEt 05	2.94178161096271	old	Tnfsf12	0.30747
StEt 05	2.94178161096271	old	Ccl20	6.32196
StEt 05	2.94178161096271	old	Fli1	-4.70574
StEt 05	2.94178161096271	old	Tpp1	3.64844
StEt 05	2.94178161096271	old	Tnr	0.58005
StEt 05	2.94178161096271	old	Vegfd	-0.37017
StEt 05	2.94178161096271	old	Parp1	-2.10279
StEt 05	2.94178161096271	old	Tnf	-3.12595
StEt 06	3.0715862335834	old	Clmp	3.33252
StEt 06	3.0715862335834	old	Matn2	0.70617
StEt 06	3.0715862335834	old	Cpe	2.12769
StEt 06	3.0715862335834	old	Gcg	0.27696
StEt 06	3.0715862335834	old	Gdnf	-3.00186

StEt 06 3.0715862335834 old Yes1 -2.19521
StEt 06 3.0715862335834 old Il17a -1.53925
StEt 06 3.0715862335834 old Foxo1 -2.78895
StEt 06 3.0715862335834 old Tnfrsf11b 0.54469
StEt 06 3.0715862335834 old Tgfb1 -0.2317
StEt 06 3.0715862335834 old Pla2g4a 2.92877
StEt 06 3.0715862335834 old Il6 -4.06333
StEt 06 3.0715862335834 old Prdx5 -1.5944
StEt 06 3.0715862335834 old Tgfa 2.50107
StEt 06 3.0715862335834 old Ccl5 -1.67006
StEt 06 3.0715862335834 old Epo 0.14963
StEt 06 3.0715862335834 old Axin1 -2.63148
StEt 06 3.0715862335834 old Fst 4.34222
StEt 06 3.0715862335834 old Rgma -0.85811
StEt 06 3.0715862335834 old Nadk 1.62157
StEt 06 3.0715862335834 old Tnni3 6.1858
StEt 06 3.0715862335834 old Notch3 2.07648
StEt 06 3.0715862335834 old Snap29 1.87918
StEt 06 3.0715862335834 old Cntn1 5.37453
StEt 06 3.0715862335834 old Clstn2 -2.25556
StEt 06 3.0715862335834 old S100a4 5.84556
StEt 06 3.0715862335834 old Ca13 -3.44944
StEt 06 3.0715862335834 old Mia 1.55923
StEt 06 3.0715862335834 old Cant1 -1.41038
StEt 06 3.0715862335834 old Kitlg -4.40421
StEt 06 3.0715862335834 old Gfra1 -1.8169
StEt 06 3.0715862335834 old Ppp1r2 -2.40791
StEt 06 3.0715862335834 old Adam23 -0.69146
StEt 06 3.0715862335834 old Itgb6 -2.54895
StEt 06 3.0715862335834 old Cyr61 1.37599
StEt 06 3.0715862335834 old Dlk1 0.40692
StEt 06 3.0715862335834 old Ahr -2.88126
StEt 06 3.0715862335834 old Ccl2 5.41162
StEt 06 3.0715862335834 old Eno2 -0.71528
StEt 06 3.0715862335834 old Plin1 -3.19092
StEt 06 3.0715862335834 old Wfikkn2 2.16041
StEt 06 3.0715862335834 old Flrt2 0.0442
StEt 06 3.0715862335834 old Qdpr -1.56677
StEt 06 3.0715862335834 old Fas 1.77014
StEt 06 3.0715862335834 old Erbb4 1.33328
StEt 06 3.0715862335834 old Riox2 -1.32303
StEt 06 3.0715862335834 old Plxna4 -1.13619
StEt 06 3.0715862335834 old Epcam -1.44987
StEt 06 3.0715862335834 old Ccl3 -0.86685
StEt 06 3.0715862335834 old Crim1 -1.54724
StEt 06 3.0715862335834 old Vsig2 1.46895
StEt 06 3.0715862335834 old Hgf -1.11998
StEt 06 3.0715862335834 old Sez6l2 3.19272
StEt 06 3.0715862335834 old Il1a 0.86332
StEt 06 3.0715862335834 old Il23r 4.50649
StEt 06 3.0715862335834 old Dll1 1.67518
StEt 06 3.0715862335834 old Ddah1 -3.26063
StEt 06 3.0715862335834 old Il10 -2.13593
StEt 06 3.0715862335834 old Tnfrsf12a 1.8059
StEt 06 3.0715862335834 old Acvrl1 0.66909
StEt 06 3.0715862335834 old Lgmn 1.66492
StEt 06 3.0715862335834 old Csf2 -3.02627
StEt 06 3.0715862335834 old Cxcl9 1.5852
StEt 06 3.0715862335834 old Map2k6 1.80137
StEt 06 3.0715862335834 old Itgb1bp2 -5.10411
StEt 06 3.0715862335834 old Il17f -4.05484
StEt 06 3.0715862335834 old Il1b -3.45542
StEt 06 3.0715862335834 old Casp3 1.98927
StEt 06 3.0715862335834 old Apbb1ip -3.51612
StEt 06 3.0715862335834 old Wisp1 0.97185
StEt 06 3.0715862335834 old Cdh6 0.68115
StEt 06 3.0715862335834 old Pdgfb 0.79927
StEt 06 3.0715862335834 old Igsf3 -0.04698
StEt 06 3.0715862335834 old Tgfbr3 0.79886
StEt 06 3.0715862335834 old Cxcl1 4.72088
StEt 06 3.0715862335834 old Pak4 -3.6365
StEt 06 3.0715862335834 old Cntn4 0.36007
StEt 06 3.0715862335834 old Ghrl 2.56829
StEt 06 3.0715862335834 old Lpl 0.88448
StEt 06 3.0715862335834 old Fstl3 2.13834
StEt 06 3.0715862335834 old Dctn2 -2.04055
StEt 06 3.0715862335834 old Il5 -2.91506
StEt 06 3.0715862335834 old Eda2r 1.09678
StEt 06 3.0715862335834 old Ntf3 -1.70498
StEt 06 3.0715862335834 old Tnfsf12 0.21974
StEt 06 3.0715862335834 old Ccl20 5.54355
StEt 06 3.0715862335834 old Fli1 -4.17933
StEt 06 3.0715862335834 old Tpp1 3.63739
StEt 06 3.0715862335834 old Tnr 0.69646
StEt 06 3.0715862335834 old Vegfd -0.42181
StEt 06 3.0715862335834 old Parp1 -2.30285
StEt 06 3.0715862335834 old Tnf -3.13597
StEt 07 2.88989187610465 old Clmp 2.88243
StEt 07 2.88989187610465 old Matn2 0.28957
StEt 07 2.88989187610465 old Cpe 1.80444
StEt 07 2.88989187610465 old Gcg 1.17464
StEt 07 2.88989187610465 old Gdnf -4.21351
StEt 07 2.88989187610465 old Yes1 -1.51142
StEt 07 2.88989187610465 old Il17a -2.17857
StEt 07 2.88989187610465 old Foxo1 -2.26049

StEt 07	2.88989187610465	old	Tnfrsf11b	0.31973
StEt 07	2.88989187610465	old	Tgfb1	-0.39104
StEt 07	2.88989187610465	old	Pla2g4a	3.01169
StEt 07	2.88989187610465	old	Il6	-3.73865
StEt 07	2.88989187610465	old	Prdx5	-0.56892
StEt 07	2.88989187610465	old	Tgfa	1.81848
StEt 07	2.88989187610465	old	Ccl5	-1.69475
StEt 07	2.88989187610465	old	Epo	-0.93545
StEt 07	2.88989187610465	old	Axin1	-2.20586
StEt 07	2.88989187610465	old	Fst	3.57765
StEt 07	2.88989187610465	old	Rgma	-0.88372
StEt 07	2.88989187610465	old	Nadk	3.88448
StEt 07	2.88989187610465	old	Tnni3	8.54472
StEt 07	2.88989187610465	old	Notch3	1.73221
StEt 07	2.88989187610465	old	Snap29	2.38603
StEt 07	2.88989187610465	old	Cntn1	4.9714
StEt 07	2.88989187610465	old	Clstn2	-2.98196
StEt 07	2.88989187610465	old	S100a4	5.30008
StEt 07	2.88989187610465	old	Ca13	-2.93708
StEt 07	2.88989187610465	old	Mia	0.94086
StEt 07	2.88989187610465	old	Cant1	-1.69831
StEt 07	2.88989187610465	old	Kitlg	-4.44805
StEt 07	2.88989187610465	old	Gfra1	-2.39116
StEt 07	2.88989187610465	old	Ppp1r2	-1.79482
StEt 07	2.88989187610465	old	Adam23	-1.04579
StEt 07	2.88989187610465	old	Itgb6	-2.28549
StEt 07	2.88989187610465	old	Cyr61	0.88995
StEt 07	2.88989187610465	old	Dlk1	0.15946
StEt 07	2.88989187610465	old	Ahr	-1.91795
StEt 07	2.88989187610465	old	Ccl2	5.57374
StEt 07	2.88989187610465	old	Eno2	-1.2456
StEt 07	2.88989187610465	old	Plin1	-2.27228
StEt 07	2.88989187610465	old	Wfikkn2	1.67567
StEt 07	2.88989187610465	old	Flrt2	-0.09643
StEt 07	2.88989187610465	old	Qdpr	0.8464
StEt 07	2.88989187610465	old	Fas	1.46826
StEt 07	2.88989187610465	old	Erbb4	0.99294
StEt 07	2.88989187610465	old	Riox2	0.88357
StEt 07	2.88989187610465	old	Plxna4	-0.56331
StEt 07	2.88989187610465	old	Epcam	-1.22403
StEt 07	2.88989187610465	old	Ccl3	-0.87706
StEt 07	2.88989187610465	old	Crim1	-1.78354
StEt 07	2.88989187610465	old	Vsig2	1.11033
StEt 07	2.88989187610465	old	Hgf	-1.24413
StEt 07	2.88989187610465	old	Sez6l2	2.49606
StEt 07	2.88989187610465	old	Il1a	0.68325
StEt 07	2.88989187610465	old	Il23r	3.84836
StEt 07	2.88989187610465	old	Dll1	1.4875
StEt 07	2.88989187610465	old	Ddah1	-0.35115
StEt 07	2.88989187610465	old	Il10	-1.92027
StEt 07	2.88989187610465	old	Tnfrsf12a	1.84895
StEt 07	2.88989187610465	old	Acvrl1	0.14647
StEt 07	2.88989187610465	old	Lgmn	1.51744
StEt 07	2.88989187610465	old	Csf2	-3.19903
StEt 07	2.88989187610465	old	Cxcl9	1.31399
StEt 07	2.88989187610465	old	Map2k6	1.78046
StEt 07	2.88989187610465	old	Itgb1bp2	-5.15162
StEt 07	2.88989187610465	old	Il17f	-4.43867
StEt 07	2.88989187610465	old	Il1b	-3.35597
StEt 07	2.88989187610465	old	Casp3	2.21422
StEt 07	2.88989187610465	old	Apbb1ip	-3.74618
StEt 07	2.88989187610465	old	Wisp1	0.54372
StEt 07	2.88989187610465	old	Cdh6	0.75188
StEt 07	2.88989187610465	old	Pdgfb	1.17264
StEt 07	2.88989187610465	old	Igsf3	-0.52442
StEt 07	2.88989187610465	old	Tgfbr3	0.46703
StEt 07	2.88989187610465	old	Cxcl1	4.79788
StEt 07	2.88989187610465	old	Pak4	-2.79777
StEt 07	2.88989187610465	old	Cntn4	-0.24068
StEt 07	2.88989187610465	old	Ghrl	2.52539
StEt 07	2.88989187610465	old	Lpl	-0.03184
StEt 07	2.88989187610465	old	Fstl3	1.60821
StEt 07	2.88989187610465	old	Dctn2	-1.97253
StEt 07	2.88989187610465	old	Il5	-2.72408
StEt 07	2.88989187610465	old	Eda2r	0.75414
StEt 07	2.88989187610465	old	Ntf3	-1.83157
StEt 07	2.88989187610465	old	Tnfsf12	-0.22855
StEt 07	2.88989187610465	old	Ccl20	4.79349
StEt 07	2.88989187610465	old	Fli1	-4.01718
StEt 07	2.88989187610465	old	Tpp1	3.18592
StEt 07	2.88989187610465	old	Tnr	0.52833
StEt 07	2.88989187610465	old	Vegfd	-0.78799
StEt 07	2.88989187610465	old	Parp1	-1.87276
StEt 07	2.88989187610465	old	Tnf	-4.12245
StEt 08	2.83156760291981	young	Clmp	3.13069
StEt 08	2.83156760291981	young	Matn2	0.06004
StEt 08	2.83156760291981	young	Cpe	2.3244
StEt 08	2.83156760291981	young	Gcg	-0.55291
StEt 08	2.83156760291981	young	Gdnf	-4.01906
StEt 08	2.83156760291981	young	Yes1	-1.77857
StEt 08	2.83156760291981	young	Il17a	-1.50495
StEt 08	2.83156760291981	young	Foxo1	-2.54627
StEt 08	2.83156760291981	young	Tnfrsf11b	0.5485
StEt 08	2.83156760291981	young	Tgfb1	-0.60888
StEt 08	2.83156760291981	young	Pla2g4a	2.78785

StEt 08	2.83156760291981	young	Il6	-4.56869
StEt 08	2.83156760291981	young	Prdx5	-1.0683
StEt 08	2.83156760291981	young	Tgfa	1.83942
StEt 08	2.83156760291981	young	Ccl5	-1.05519
StEt 08	2.83156760291981	young	Epo	-0.55119
StEt 08	2.83156760291981	young	Axin1	-2.70136
StEt 08	2.83156760291981	young	Fst	3.97794
StEt 08	2.83156760291981	young	Rgma	-0.96722
StEt 08	2.83156760291981	young	Nadk	2.40875
StEt 08	2.83156760291981	young	Tnni3	6.96268
StEt 08	2.83156760291981	young	Notch3	1.63323
StEt 08	2.83156760291981	young	Snap29	1.45679
StEt 08	2.83156760291981	young	Cntn1	5.14533
StEt 08	2.83156760291981	young	Clstn2	-2.67392
StEt 08	2.83156760291981	young	S100a4	5.98188
StEt 08	2.83156760291981	young	Ca13	-3.06412
StEt 08	2.83156760291981	young	Mia	1.70931
StEt 08	2.83156760291981	young	Cant1	-1.25394
StEt 08	2.83156760291981	young	Kitlg	-5.41876
StEt 08	2.83156760291981	young	Gfra1	-2.01322
StEt 08	2.83156760291981	young	Ppp1r2	-3.15179
StEt 08	2.83156760291981	young	Adam23	-1.49641
StEt 08	2.83156760291981	young	Itgb6	-2.31871
StEt 08	2.83156760291981	young	Cyr61	0.35546
StEt 08	2.83156760291981	young	Dlk1	1.15483
StEt 08	2.83156760291981	young	Ahr	-2.95857
StEt 08	2.83156760291981	young	Ccl2	6.27167
StEt 08	2.83156760291981	young	Eno2	-0.7564
StEt 08	2.83156760291981	young	Plin1	-0.29575
StEt 08	2.83156760291981	young	Wfikkn2	2.25377
StEt 08	2.83156760291981	young	Flrt2	-0.31317
StEt 08	2.83156760291981	young	Qdpr	-0.69082
StEt 08	2.83156760291981	young	Fas	1.33731
StEt 08	2.83156760291981	young	Erbb4	1.10676
StEt 08	2.83156760291981	young	Riox2	-0.75331
StEt 08	2.83156760291981	young	Plxna4	-0.2122
StEt 08	2.83156760291981	young	Epcam	-1.03075
StEt 08	2.83156760291981	young	Ccl3	-0.49755
StEt 08	2.83156760291981	young	Crim1	-1.77837
StEt 08	2.83156760291981	young	Vsig2	1.76457
StEt 08	2.83156760291981	young	Hgf	-0.91394
StEt 08	2.83156760291981	young	Sez6l2	2.11123
StEt 08	2.83156760291981	young	Il1a	0.96909
StEt 08	2.83156760291981	young	Il23r	4.65518
StEt 08	2.83156760291981	young	Dll1	1.29856
StEt 08	2.83156760291981	young	Ddah1	-1.99629
StEt 08	2.83156760291981	young	Il10	-2.21387
StEt 08	2.83156760291981	young	Tnfrsf12a	2.08241
StEt 08	2.83156760291981	young	Acvrl1	0.29274
StEt 08	2.83156760291981	young	Lgmn	1.90791
StEt 08	2.83156760291981	young	Csf2	-3.22895
StEt 08	2.83156760291981	young	Cxcl9	1.21805
StEt 08	2.83156760291981	young	Map2k6	1.67409
StEt 08	2.83156760291981	young	Itgb1bp2	-3.78783
StEt 08	2.83156760291981	young	Il17f	-4.56744
StEt 08	2.83156760291981	young	Il1b	-3.65087
StEt 08	2.83156760291981	young	Casp3	1.61987
StEt 08	2.83156760291981	young	Apbb1ip	-3.87207
StEt 08	2.83156760291981	young	Wisp1	1.15474
StEt 08	2.83156760291981	young	Cdh6	1.04983
StEt 08	2.83156760291981	young	Pdgfb	1.86251
StEt 08	2.83156760291981	young	Igsf3	-0.72942
StEt 08	2.83156760291981	young	Tgfbr3	0.65116
StEt 08	2.83156760291981	young	Cxcl1	4.48177
StEt 08	2.83156760291981	young	Pak4	-4.25362
StEt 08	2.83156760291981	young	Cntn4	1.11734
StEt 08	2.83156760291981	young	Ghrl	3.43283
StEt 08	2.83156760291981	young	Lpl	0.61673
StEt 08	2.83156760291981	young	Fstl3	1.86097
StEt 08	2.83156760291981	young	Dctn2	-2.10716
StEt 08	2.83156760291981	young	Il5	-2.17131
StEt 08	2.83156760291981	young	Eda2r	0.90561
StEt 08	2.83156760291981	young	Ntf3	-1.9242
StEt 08	2.83156760291981	young	Tnfsf12	0.22816
StEt 08	2.83156760291981	young	Ccl20	5.86854
StEt 08	2.83156760291981	young	Fli1	-4.50732
StEt 08	2.83156760291981	young	Tpp1	3.34997
StEt 08	2.83156760291981	young	Tnr	-0.16651
StEt 08	2.83156760291981	young	Vegfd	-0.4
StEt 08	2.83156760291981	young	Parp1	-2.10551
StEt 08	2.83156760291981	young	Tnf	-3.42356
StEt 09	3.9442601317361	young	Clmp	2.43883
StEt 09	3.9442601317361	young	Matn2	-0.487
StEt 09	3.9442601317361	young	Cpe	1.77448
StEt 09	3.9442601317361	young	Gcg	-1.89502
StEt 09	3.9442601317361	young	Gdnf	-4.72918
StEt 09	3.9442601317361	young	Yes1	0.5085
StEt 09	3.9442601317361	young	Il17a	-0.9332
StEt 09	3.9442601317361	young	Foxo1	-1.14832
StEt 09	3.9442601317361	young	Tnfrsf11b	-0.06051
StEt 09	3.9442601317361	young	Tgfb1	-0.592
StEt 09	3.9442601317361	young	Pla2g4a	3.20186
StEt 09	3.9442601317361	young	Il6	-3.66829
StEt 09	3.9442601317361	young	Prdx5	0.13906
StEt 09	3.9442601317361	young	Tgfa	1.6914

StEt 09	3.9442601317361	young	Ccl5	-1.44652
StEt 09	3.9442601317361	young	Epo	-1.20626
StEt 09	3.9442601317361	young	Axin1	-1.25028
StEt 09	3.9442601317361	young	Fst	3.37671
StEt 09	3.9442601317361	young	Rgma	-1.06517
StEt 09	3.9442601317361	young	Nadk	5.32679
StEt 09	3.9442601317361	young	Tnni3	9.66264
StEt 09	3.9442601317361	young	Notch3	1.9144
StEt 09	3.9442601317361	young	Snap29	3.72886
StEt 09	3.9442601317361	young	Cntn1	4.56178
StEt 09	3.9442601317361	young	Clstn2	-3.91091
StEt 09	3.9442601317361	young	S100a4	5.98433
StEt 09	3.9442601317361	young	Ca13	-1.69205
StEt 09	3.9442601317361	young	Mia	0.87015
StEt 09	3.9442601317361	young	Cant1	-2.10222
StEt 09	3.9442601317361	young	Kitlg	-5.73446
StEt 09	3.9442601317361	young	Gfra1	-3.30165
StEt 09	3.9442601317361	young	Ppp1r2	-1.42139
StEt 09	3.9442601317361	young	Adam23	-2.50186
StEt 09	3.9442601317361	young	Itgb6	-3.31137
StEt 09	3.9442601317361	young	Cyr61	-0.22579
StEt 09	3.9442601317361	young	Dlk1	0.40216
StEt 09	3.9442601317361	young	Ahr	-0.45503
StEt 09	3.9442601317361	young	Ccl2	6.57284
StEt 09	3.9442601317361	young	Eno2	-1.38367
StEt 09	3.9442601317361	young	Plin1	0.9319
StEt 09	3.9442601317361	young	Wfikkn2	1.67182
StEt 09	3.9442601317361	young	Flrt2	-0.14344
StEt 09	3.9442601317361	young	Qdpr	1.92102
StEt 09	3.9442601317361	young	Fas	1.1316
StEt 09	3.9442601317361	young	Erbb4	0.31142
StEt 09	3.9442601317361	young	Riox2	2.06138
StEt 09	3.9442601317361	young	Plxna4	-1.13831
StEt 09	3.9442601317361	young	Epcam	-2.07841
StEt 09	3.9442601317361	young	Ccl3	-0.77586
StEt 09	3.9442601317361	young	Crim1	-2.03114
StEt 09	3.9442601317361	young	Vsig2	0.97551
StEt 09	3.9442601317361	young	Hgf	-0.95104
StEt 09	3.9442601317361	young	Sez6l2	1.68345
StEt 09	3.9442601317361	young	Il1a	1.19452
StEt 09	3.9442601317361	young	Il23r	3.8705
StEt 09	3.9442601317361	young	Dll1	0.46625
StEt 09	3.9442601317361	young	Ddah1	1.27854
StEt 09	3.9442601317361	young	Il10	-2.64709
StEt 09	3.9442601317361	young	Tnfrsf12a	0.9619
StEt 09	3.9442601317361	young	Acvrl1	-0.23399
StEt 09	3.9442601317361	young	Lgmn	1.57201
StEt 09	3.9442601317361	young	Csf2	-3.23904
StEt 09	3.9442601317361	young	Cxcl9	0.38794
StEt 09	3.9442601317361	young	Map2k6	3.16054
StEt 09	3.9442601317361	young	Itgb1bp2	-6.60631
StEt 09	3.9442601317361	young	Il17f	-4.74381
StEt 09	3.9442601317361	young	Il1b	-3.03513
StEt 09	3.9442601317361	young	Casp3	3.14571
StEt 09	3.9442601317361	young	Apbb1ip	-3.39278
StEt 09	3.9442601317361	young	Wisp1	0.34629
StEt 09	3.9442601317361	young	Cdh6	0.39881
StEt 09	3.9442601317361	young	Pdgfb	3.14126
StEt 09	3.9442601317361	young	Igsf3	-1.02822
StEt 09	3.9442601317361	young	Tgfbr3	0.05155
StEt 09	3.9442601317361	young	Cxcl1	4.41689
StEt 09	3.9442601317361	young	Pak4	-3.37655
StEt 09	3.9442601317361	young	Cntn4	0.72708
StEt 09	3.9442601317361	young	Ghrl	1.9423
StEt 09	3.9442601317361	young	Lpl	-0.33052
StEt 09	3.9442601317361	young	Fstl3	1.10526
StEt 09	3.9442601317361	young	Dctn2	-1.57517
StEt 09	3.9442601317361	young	Il5	-2.02524
StEt 09	3.9442601317361	young	Eda2r	0.09026
StEt 09	3.9442601317361	young	Ntf3	-2.92551
StEt 09	3.9442601317361	young	Tnfsf12	-0.30971
StEt 09	3.9442601317361	young	Ccl20	3.90072
StEt 09	3.9442601317361	young	Fli1	-4.36768
StEt 09	3.9442601317361	young	Tpp1	2.80957
StEt 09	3.9442601317361	young	Tnr	-0.52792
StEt 09	3.9442601317361	young	Vegfd	-1.24083
StEt 09	3.9442601317361	young	Parp1	-0.79426
StEt 09	3.9442601317361	young	Tnf	-3.77085
StEt 10	3.75283167919481	old	Clmp	3.04362
StEt 10	3.75283167919481	old	Matn2	0.48851
StEt 10	3.75283167919481	old	Cpe	2.15745
StEt 10	3.75283167919481	old	Gcg	1.31749
StEt 10	3.75283167919481	old	Gdnf	-3.82005
StEt 10	3.75283167919481	old	Yes1	-1.92813
StEt 10	3.75283167919481	old	Il17a	-2.86199
StEt 10	3.75283167919481	old	Foxo1	-2.04615
StEt 10	3.75283167919481	old	Tnfrsf11b	0.58996
StEt 10	3.75283167919481	old	Tgfb1	-0.50103
StEt 10	3.75283167919481	old	Pla2g4a	3.20865
StEt 10	3.75283167919481	old	Il6	-4.47039
StEt 10	3.75283167919481	old	Prdx5	-1.43147
StEt 10	3.75283167919481	old	Tgfa	2.22871
StEt 10	3.75283167919481	old	Ccl5	-1.61465
StEt 10	3.75283167919481	old	Epo	-0.81652
StEt 10	3.75283167919481	old	Axin1	-2.14714

StEt 10	3.75283167919481	old	Fst	3.95173
StEt 10	3.75283167919481	old	Rgma	-0.98259
StEt 10	3.75283167919481	old	Nadk	2.54333
StEt 10	3.75283167919481	old	Tnni3	5.48902
StEt 10	3.75283167919481	old	Notch3	2.08603
StEt 10	3.75283167919481	old	Snap29	2.20215
StEt 10	3.75283167919481	old	Cntn1	5.41875
StEt 10	3.75283167919481	old	Clstn2	-2.62386
StEt 10	3.75283167919481	old	S100a4	5.76552
StEt 10	3.75283167919481	old	Ca13	-2.59454
StEt 10	3.75283167919481	old	Mia	1.15608
StEt 10	3.75283167919481	old	Cant1	-1.42933
StEt 10	3.75283167919481	old	Kitlg	-4.75416
StEt 10	3.75283167919481	old	Gfra1	-1.93532
StEt 10	3.75283167919481	old	Ppp1r2	-2.27899
StEt 10	3.75283167919481	old	Adam23	-0.86143
StEt 10	3.75283167919481	old	Itgb6	-2.6507
StEt 10	3.75283167919481	old	Cyr61	1.20898
StEt 10	3.75283167919481	old	Dlk1	0.46275
StEt 10	3.75283167919481	old	Ahr	-2.36357
StEt 10	3.75283167919481	old	Ccl2	5.05355
StEt 10	3.75283167919481	old	Eno2	-0.81203
StEt 10	3.75283167919481	old	Plin1	-1.95761
StEt 10	3.75283167919481	old	Wfikkn2	1.87505
StEt 10	3.75283167919481	old	Flrt2	0.17474
StEt 10	3.75283167919481	old	Qdpr	-0.57952
StEt 10	3.75283167919481	old	Fas	1.75912
StEt 10	3.75283167919481	old	Erbb4	1.32424
StEt 10	3.75283167919481	old	Riox2	-0.06285
StEt 10	3.75283167919481	old	Plxna4	0.28776
StEt 10	3.75283167919481	old	Epcam	-1.32831
StEt 10	3.75283167919481	old	Ccl3	-0.90762
StEt 10	3.75283167919481	old	Crim1	-1.85194
StEt 10	3.75283167919481	old	Vsig2	1.51855
StEt 10	3.75283167919481	old	Hgf	-1.15263
StEt 10	3.75283167919481	old	Sez6l2	3.10126
StEt 10	3.75283167919481	old	Il1a	0.75143
StEt 10	3.75283167919481	old	Il23r	4.15406
StEt 10	3.75283167919481	old	Dll1	1.56978
StEt 10	3.75283167919481	old	Ddah1	-2.7377
StEt 10	3.75283167919481	old	Il10	-2.35514
StEt 10	3.75283167919481	old	Tnfrsf12a	2.06745
StEt 10	3.75283167919481	old	Acvrl1	0.25085
StEt 10	3.75283167919481	old	Lgmn	1.23358
StEt 10	3.75283167919481	old	Csf2	-2.97548
StEt 10	3.75283167919481	old	Cxcl9	1.21627
StEt 10	3.75283167919481	old	Map2k6	1.84359
StEt 10	3.75283167919481	old	Itgb1bp2	-7.08385
StEt 10	3.75283167919481	old	Il17f	-4.63101
StEt 10	3.75283167919481	old	Il1b	-3.4707
StEt 10	3.75283167919481	old	Casp3	2.35727
StEt 10	3.75283167919481	old	Apbb1ip	-3.43104
StEt 10	3.75283167919481	old	Wisp1	0.85891
StEt 10	3.75283167919481	old	Cdh6	1.32323
StEt 10	3.75283167919481	old	Pdgfb	1.31571
StEt 10	3.75283167919481	old	Igsf3	-0.18661
StEt 10	3.75283167919481	old	Tgfbr3	0.85717
StEt 10	3.75283167919481	old	Cxcl1	5.05874
StEt 10	3.75283167919481	old	Pak4	-2.84291
StEt 10	3.75283167919481	old	Cntn4	0.29
StEt 10	3.75283167919481	old	Ghrl	2.46588
StEt 10	3.75283167919481	old	Lpl	0.7118
StEt 10	3.75283167919481	old	Fstl3	1.91596
StEt 10	3.75283167919481	old	Dctn2	-2.29571
StEt 10	3.75283167919481	old	Il5	-3.38201
StEt 10	3.75283167919481	old	Eda2r	1.03641
StEt 10	3.75283167919481	old	Ntf3	-1.79519
StEt 10	3.75283167919481	old	Tnfsf12	0.07601
StEt 10	3.75283167919481	old	Ccl20	5.20826
StEt 10	3.75283167919481	old	Fli1	-3.46902
StEt 10	3.75283167919481	old	Tpp1	3.37404
StEt 10	3.75283167919481	old	Tnr	0.71903
StEt 10	3.75283167919481	old	Vegfd	-0.49498
StEt 10	3.75283167919481	old	Parp1	-2.27002
StEt 10	3.75283167919481	old	Tnf	-2.88286
StEt 11	4.28400052568302	young	Clmp	1.9663
StEt 11	4.28400052568302	young	Matn2	-0.33552
StEt 11	4.28400052568302	young	Cpe	1.20808
StEt 11	4.28400052568302	young	Gcg	-2.71216
StEt 11	4.28400052568302	young	Gdnf	-4.53374
StEt 11	4.28400052568302	young	Yes1	1.90643
StEt 11	4.28400052568302	young	Il17a	-2.71524
StEt 11	4.28400052568302	young	Foxo1	-0.455
StEt 11	4.28400052568302	young	Tnfrsf11b	-0.63632
StEt 11	4.28400052568302	young	Tgfb1	-0.82621
StEt 11	4.28400052568302	young	Pla2g4a	3.17619
StEt 11	4.28400052568302	young	Il6	-4.94184
StEt 11	4.28400052568302	young	Prdx5	2.5111
StEt 11	4.28400052568302	young	Tgfa	2.16989
StEt 11	4.28400052568302	young	Ccl5	-1.24046
StEt 11	4.28400052568302	young	Epo	-1.56284
StEt 11	4.28400052568302	young	Axin1	-0.35035
StEt 11	4.28400052568302	young	Fst	2.92397
StEt 11	4.28400052568302	young	Rgma	-1.41573
StEt 11	4.28400052568302	young	Nadk	5.17834

StEt 11	4.28400052568302	young	Tnni3	10.26491
StEt 11	4.28400052568302	young	Notch3	1.20112
StEt 11	4.28400052568302	young	Snap29	4.11401
StEt 11	4.28400052568302	young	Cntn1	4.13952
StEt 11	4.28400052568302	young	Clstn2	-3.44919
StEt 11	4.28400052568302	young	S100a4	4.86009
StEt 11	4.28400052568302	young	Ca13	-2.25067
StEt 11	4.28400052568302	young	Mia	0.13899
StEt 11	4.28400052568302	young	Cant1	-2.21042
StEt 11	4.28400052568302	young	Kitlg	-5.58389
StEt 11	4.28400052568302	young	Gfra1	-3.60097
StEt 11	4.28400052568302	young	Ppp1r2	-1.07096
StEt 11	4.28400052568302	young	Adam23	-2.0296
StEt 11	4.28400052568302	young	Itgb6	-3.49595
StEt 11	4.28400052568302	young	Cyr61	-0.29498
StEt 11	4.28400052568302	young	Dlk1	-0.40931
StEt 11	4.28400052568302	young	Ahr	0.20414
StEt 11	4.28400052568302	young	Ccl2	5.27727
StEt 11	4.28400052568302	young	Eno2	-1.43863
StEt 11	4.28400052568302	young	Plin1	1.04221
StEt 11	4.28400052568302	young	Wfikkn2	0.95811
StEt 11	4.28400052568302	young	Flrt2	0.70991
StEt 11	4.28400052568302	young	Qdpr	2.07284
StEt 11	4.28400052568302	young	Fas	1.80744
StEt 11	4.28400052568302	young	Erbb4	0.60518
StEt 11	4.28400052568302	young	Riox2	2.36454
StEt 11	4.28400052568302	young	Plxna4	0.1885
StEt 11	4.28400052568302	young	Epcam	-2.34069
StEt 11	4.28400052568302	young	Ccl3	-1.32466
StEt 11	4.28400052568302	young	Crim1	-1.90625
StEt 11	4.28400052568302	young	Vsig2	0.55527
StEt 11	4.28400052568302	young	Hgf	-0.99284
StEt 11	4.28400052568302	young	Sez6l2	1.76978
StEt 11	4.28400052568302	young	Il1a	1.38757
StEt 11	4.28400052568302	young	Il23r	3.53357
StEt 11	4.28400052568302	young	Dll1	0.45824
StEt 11	4.28400052568302	young	Ddah1	3.04804
StEt 11	4.28400052568302	young	Il10	-2.77094
StEt 11	4.28400052568302	young	Tnfrsf12a	0.6213
StEt 11	4.28400052568302	young	Acvrl1	-0.44377
StEt 11	4.28400052568302	young	Lgmn	1.53211
StEt 11	4.28400052568302	young	Csf2	-4.27197
StEt 11	4.28400052568302	young	Cxcl9	0.54947
StEt 11	4.28400052568302	young	Map2k6	2.99533
StEt 11	4.28400052568302	young	Itgb1bp2	-4.65003
StEt 11	4.28400052568302	young	Il17f	-5.70008
StEt 11	4.28400052568302	young	Il1b	-3.50847
StEt 11	4.28400052568302	young	Casp3	3.96329
StEt 11	4.28400052568302	young	Apbb1ip	-3.19629
StEt 11	4.28400052568302	young	Wisp1	-0.22337
StEt 11	4.28400052568302	young	Cdh6	-0.64498
StEt 11	4.28400052568302	young	Pdgfb	3.21662
StEt 11	4.28400052568302	young	Igsf3	-0.89048
StEt 11	4.28400052568302	young	Tgfbr3	-0.11652
StEt 11	4.28400052568302	young	Cxcl1	3.61749
StEt 11	4.28400052568302	young	Pak4	-3.01062
StEt 11	4.28400052568302	young	Cntn4	-0.58005
StEt 11	4.28400052568302	young	Ghrl	1.2533
StEt 11	4.28400052568302	young	Lpl	-0.38905
StEt 11	4.28400052568302	young	Fstl3	0.70131
StEt 11	4.28400052568302	young	Dctn2	0.68061
StEt 11	4.28400052568302	young	Il5	-4.40606
StEt 11	4.28400052568302	young	Eda2r	-0.33828
StEt 11	4.28400052568302	young	Ntf3	-2.77446
StEt 11	4.28400052568302	young	Tnfsf12	-0.11123
StEt 11	4.28400052568302	young	Ccl20	4.43514
StEt 11	4.28400052568302	young	Fli1	-4.25603
StEt 11	4.28400052568302	young	Tpp1	2.59851
StEt 11	4.28400052568302	young	Tnr	-0.60112
StEt 11	4.28400052568302	young	Vegfd	-1.67915
StEt 11	4.28400052568302	young	Parp1	0.05497
StEt 11	4.28400052568302	young	Tnf	-3.9235
StEt 12	3.13855140101639	young	Clmp	2.92156
StEt 12	3.13855140101639	young	Matn2	0.18178
StEt 12	3.13855140101639	young	Cpe	1.94489
StEt 12	3.13855140101639	young	Gcg	-1.82807
StEt 12	3.13855140101639	young	Gdnf	-4.39794
StEt 12	3.13855140101639	young	Yes1	-1.97324
StEt 12	3.13855140101639	young	Il17a	-1.50401
StEt 12	3.13855140101639	young	Foxo1	-2.9104
StEt 12	3.13855140101639	young	Tnfrsf11b	0.14894
StEt 12	3.13855140101639	young	Tgfb1	-0.25864
StEt 12	3.13855140101639	young	Pla2g4a	4.02371
StEt 12	3.13855140101639	young	Il6	-4.25705
StEt 12	3.13855140101639	young	Prdx5	-0.13004
StEt 12	3.13855140101639	young	Tgfa	1.74972
StEt 12	3.13855140101639	young	Ccl5	-1.23996
StEt 12	3.13855140101639	young	Epo	-1.36546
StEt 12	3.13855140101639	young	Axin1	-1.73077
StEt 12	3.13855140101639	young	Fst	2.81584
StEt 12	3.13855140101639	young	Rgma	-1.08031
StEt 12	3.13855140101639	young	Nadk	3.31624
StEt 12	3.13855140101639	young	Tnni3	8.74332
StEt 12	3.13855140101639	young	Notch3	1.89444
StEt 12	3.13855140101639	young	Snap29	2.96687

StEt	12	3.13855140101639	young	Cntn1	5.02079
StEt	12	3.13855140101639	young	Clstn2	-2.9155
StEt	12	3.13855140101639	young	S100a4	5.42784
StEt	12	3.13855140101639	young	Ca13	-3.43508
StEt	12	3.13855140101639	young	Mia	1.03935
StEt	12	3.13855140101639	young	Cant1	-1.65397
StEt	12	3.13855140101639	young	Kitlg	-5.09275
StEt	12	3.13855140101639	young	Gfra1	-2.4038
StEt	12	3.13855140101639	young	Ppp1r2	-3.02929
StEt	12	3.13855140101639	young	Adam23	-1.70201
StEt	12	3.13855140101639	young	Itgb6	-3.11958
StEt	12	3.13855140101639	young	Cyr61	-0.04757
StEt	12	3.13855140101639	young	Dlk1	2.35273
StEt	12	3.13855140101639	young	Ahr	-2.77346
StEt	12	3.13855140101639	young	Ccl2	6.01697
StEt	12	3.13855140101639	young	Eno2	-1.49515
StEt	12	3.13855140101639	young	Plin1	1.20945
StEt	12	3.13855140101639	young	Wfikkn2	1.66649
StEt	12	3.13855140101639	young	Flrt2	-0.56132
StEt	12	3.13855140101639	young	Qdpr	0.27147
StEt	12	3.13855140101639	young	Fas	1.34749
StEt	12	3.13855140101639	young	Erbb4	0.637
StEt	12	3.13855140101639	young	Riox2	0.51481
StEt	12	3.13855140101639	young	Plxna4	1.24823
StEt	12	3.13855140101639	young	Epcam	-1.30828
StEt	12	3.13855140101639	young	Ccl3	-1.12447
StEt	12	3.13855140101639	young	Crim1	-2.0014
StEt	12	3.13855140101639	young	Vsig2	1.05071
StEt	12	3.13855140101639	young	Hgf	-0.87779
StEt	12	3.13855140101639	young	Sez6l2	2.33139
StEt	12	3.13855140101639	young	Il1a	2.60888
StEt	12	3.13855140101639	young	Il23r	4.45834
StEt	12	3.13855140101639	young	Dll1	0.98433
StEt	12	3.13855140101639	young	Ddah1	-1.29933
StEt	12	3.13855140101639	young	Il10	-2.70917
StEt	12	3.13855140101639	young	Tnfrsf12a	1.5614
StEt	12	3.13855140101639	young	Acvrl1	0.10657
StEt	12	3.13855140101639	young	Lgmn	1.49729
StEt	12	3.13855140101639	young	Csf2	-3.15951
StEt	12	3.13855140101639	young	Cxcl9	1.86278
StEt	12	3.13855140101639	young	Map2k6	3.16425
StEt	12	3.13855140101639	young	Itgb1bp2	-9.05602
StEt	12	3.13855140101639	young	Il17f	-4.9514
StEt	12	3.13855140101639	young	Il1b	-3.64183
StEt	12	3.13855140101639	young	Casp3	3.57504
StEt	12	3.13855140101639	young	Apbb1ip	-3.44288
StEt	12	3.13855140101639	young	Wisp1	0.83435
StEt	12	3.13855140101639	young	Cdh6	0.89463
StEt	12	3.13855140101639	young	Pdgfb	3.83449
StEt	12	3.13855140101639	young	Igsf3	-0.54517
StEt	12	3.13855140101639	young	Tgfbr3	0.34498
StEt	12	3.13855140101639	young	Cxcl1	4.45504
StEt	12	3.13855140101639	young	Pak4	-4.4473
StEt	12	3.13855140101639	young	Cntn4	1.07313
StEt	12	3.13855140101639	young	Ghrl	2.59759
StEt	12	3.13855140101639	young	Lpl	-0.05252
StEt	12	3.13855140101639	young	Fstl3	1.37709
StEt	12	3.13855140101639	young	Dctn2	-0.59507
StEt	12	3.13855140101639	young	Il5	-2.2708
StEt	12	3.13855140101639	young	Eda2r	0.67338
StEt	12	3.13855140101639	young	Ntf3	-2.33675
StEt	12	3.13855140101639	young	Tnfsf12	0.12571
StEt	12	3.13855140101639	young	Ccl20	4.74193
StEt	12	3.13855140101639	young	Fli1	-3.65208
StEt	12	3.13855140101639	young	Tpp1	3.04667
StEt	12	3.13855140101639	young	Tnr	-0.17656
StEt	12	3.13855140101639	young	Vegfd	-0.83407
StEt	12	3.13855140101639	young	Parp1	-1.53717
StEt	12	3.13855140101639	young	Tnf	-3.73536
StEt	14	3.60986079057401	young	Clmp	2.56955
StEt	14	3.60986079057401	young	Matn2	0.3496
StEt	14	3.60986079057401	young	Cpe	1.68929
StEt	14	3.60986079057401	young	Gcg	-1.51044
StEt	14	3.60986079057401	young	Gdnf	-4.37599
StEt	14	3.60986079057401	young	Yes1	0.68192
StEt	14	3.60986079057401	young	Il17a	-1.68404
StEt	14	3.60986079057401	young	Foxo1	-1.33417
StEt	14	3.60986079057401	young	Tnfrsf11b	-0.20033
StEt	14	3.60986079057401	young	Tgfb1	-0.41962
StEt	14	3.60986079057401	young	Pla2g4a	3.38542
StEt	14	3.60986079057401	young	Il6	-4.74154
StEt	14	3.60986079057401	young	Prdx5	1.03777
StEt	14	3.60986079057401	young	Tgfa	2.20313
StEt	14	3.60986079057401	young	Ccl5	-1.76873
StEt	14	3.60986079057401	young	Epo	-1.85933
StEt	14	3.60986079057401	young	Axin1	-0.89036
StEt	14	3.60986079057401	young	Fst	2.42261
StEt	14	3.60986079057401	young	Rgma	-1.39978
StEt	14	3.60986079057401	young	Nadk	4.67885
StEt	14	3.60986079057401	young	Tnni3	7.92697
StEt	14	3.60986079057401	young	Notch3	1.1479
StEt	14	3.60986079057401	young	Snap29	3.17641
StEt	14	3.60986079057401	young	Cntn1	4.42962
StEt	14	3.60986079057401	young	Clstn2	-2.96739
StEt	14	3.60986079057401	young	S100a4	5.94702

StEt 14 3.60986079057401 young Ca13 -1.32649
StEt 14 3.60986079057401 young Mia 0.34071
StEt 14 3.60986079057401 young Cant1 -2.45246
StEt 14 3.60986079057401 young Kitlg -5.68124
StEt 14 3.60986079057401 young Gfra1 -2.62554
StEt 14 3.60986079057401 young Ppp1r2 -2.09858
StEt 14 3.60986079057401 young Adam23 -2.38299
StEt 14 3.60986079057401 young Itgb6 -3.82315
StEt 14 3.60986079057401 young Cyr61 -0.43705
StEt 14 3.60986079057401 young Dlk1 0.0093
StEt 14 3.60986079057401 young Ahr -0.8317
StEt 14 3.60986079057401 young Ccl2 5.54698
StEt 14 3.60986079057401 young Eno2 -0.84846
StEt 14 3.60986079057401 young Plin1 2.21802
StEt 14 3.60986079057401 young Wfikkn2 1.00902
StEt 14 3.60986079057401 young Flrt2 0.38206
StEt 14 3.60986079057401 young Qdpr 1.58915
StEt 14 3.60986079057401 young Fas 1.59698
StEt 14 3.60986079057401 young Erbb4 -0.26963
StEt 14 3.60986079057401 young Riox2 2.49534
StEt 14 3.60986079057401 young Plxna4 -0.04755
StEt 14 3.60986079057401 young Epcam -1.97951
StEt 14 3.60986079057401 young Ccl3 -1.24063
StEt 14 3.60986079057401 young Crim1 -2.01428
StEt 14 3.60986079057401 young Vsig2 0.75941
StEt 14 3.60986079057401 young Hgf -0.71878
StEt 14 3.60986079057401 young Sez6l2 2.11796
StEt 14 3.60986079057401 young Il1a 1.89418
StEt 14 3.60986079057401 young Il23r 3.45282
StEt 14 3.60986079057401 young Dll1 0.46906
StEt 14 3.60986079057401 young Ddah1 1.70515
StEt 14 3.60986079057401 young Il10 -2.22236
StEt 14 3.60986079057401 young Tnfrsf12a 1.42921
StEt 14 3.60986079057401 young Acvrl1 -0.20762
StEt 14 3.60986079057401 young Lgmn 1.30676
StEt 14 3.60986079057401 young Csf2 -3.97576
StEt 14 3.60986079057401 young Cxcl9 1.28702
StEt 14 3.60986079057401 young Map2k6 2.53882
StEt 14 3.60986079057401 young Itgb1bp2 -6.38184
StEt 14 3.60986079057401 young Il17f -5.58118
StEt 14 3.60986079057401 young Il1b -3.34651
StEt 14 3.60986079057401 young Casp3 3.89435
StEt 14 3.60986079057401 young Apbb1ip -2.65806
StEt 14 3.60986079057401 young Wisp1 0.3327
StEt 14 3.60986079057401 young Cdh6 0.07938
StEt 14 3.60986079057401 young Pdgfb 4.56074
StEt 14 3.60986079057401 young Igsf3 -0.34357
StEt 14 3.60986079057401 young Tgfbr3 -0.30116
StEt 14 3.60986079057401 young Cxcl1 4.01641
StEt 14 3.60986079057401 young Pak4 -3.48849
StEt 14 3.60986079057401 young Cntn4 0.26782
StEt 14 3.60986079057401 young Ghrl 1.64927
StEt 14 3.60986079057401 young Lpl -0.32286
StEt 14 3.60986079057401 young Fstl3 1.17337
StEt 14 3.60986079057401 young Dctn2 -0.47047
StEt 14 3.60986079057401 young Il5 -3.76287
StEt 14 3.60986079057401 young Eda2r 0.26135
StEt 14 3.60986079057401 young Ntf3 -2.57788
StEt 14 3.60986079057401 young Tnfsf12 -0.14316
StEt 14 3.60986079057401 young Ccl20 3.49891
StEt 14 3.60986079057401 young Fli1 -2.7496
StEt 14 3.60986079057401 young Tpp1 2.52128
StEt 14 3.60986079057401 young Tnr -0.79879
StEt 14 3.60986079057401 young Vegfd -1.41759
StEt 14 3.60986079057401 young Parp1 0.89643
StEt 14 3.60986079057401 young Tnf -4.26604
StEt 15 3.58154039576409 young Clmp 2.92171
StEt 15 3.58154039576409 young Matn2 0.22438
StEt 15 3.58154039576409 young Cpe 1.76516
StEt 15 3.58154039576409 young Gcg -1.28425
StEt 15 3.58154039576409 young Gdnf -4.4252
StEt 15 3.58154039576409 young Yes1 -1.33357
StEt 15 3.58154039576409 young Il17a -1.96613
StEt 15 3.58154039576409 young Foxo1 -2.10099
StEt 15 3.58154039576409 young Tnfrsf11b 0.04315
StEt 15 3.58154039576409 young Tgfb1 -0.24224
StEt 15 3.58154039576409 young Pla2g4a 3.5635
StEt 15 3.58154039576409 young Il6 -4.69575
StEt 15 3.58154039576409 young Prdx5 0.39996
StEt 15 3.58154039576409 young Tgfa 1.69566
StEt 15 3.58154039576409 young Ccl5 -0.38224
StEt 15 3.58154039576409 young Epo -1.19892
StEt 15 3.58154039576409 young Axin1 -1.95848
StEt 15 3.58154039576409 young Fst 3.32807
StEt 15 3.58154039576409 young Rgma -1.35042
StEt 15 3.58154039576409 young Nadk 4.07486
StEt 15 3.58154039576409 young Tnni3 6.88776
StEt 15 3.58154039576409 young Notch3 2.01394
StEt 15 3.58154039576409 young Snap29 3.16353
StEt 15 3.58154039576409 young Cntn1 4.87606
StEt 15 3.58154039576409 young Clstn2 -3.08925
StEt 15 3.58154039576409 young S100a4 5.13015
StEt 15 3.58154039576409 young Ca13 -3.06263
StEt 15 3.58154039576409 young Mia 0.95433
StEt 15 3.58154039576409 young Cant1 -1.48364

StEt 15	3.58154039576409	young	Kitlg	-5.49492
StEt 15	3.58154039576409	young	Gfra1	-2.4809
StEt 15	3.58154039576409	young	Ppp1r2	-2.75412
StEt 15	3.58154039576409	young	Adam23	-0.96755
StEt 15	3.58154039576409	young	Itgb6	-2.75992
StEt 15	3.58154039576409	young	Cyr61	0.36804
StEt 15	3.58154039576409	young	Dlk1	0.36633
StEt 15	3.58154039576409	young	Ahr	-1.97066
StEt 15	3.58154039576409	young	Ccl2	5.74602
StEt 15	3.58154039576409	young	Eno2	-1.16405
StEt 15	3.58154039576409	young	Plin1	-1.88675
StEt 15	3.58154039576409	young	Wfikkn2	1.60456
StEt 15	3.58154039576409	young	Flrt2	0.10235
StEt 15	3.58154039576409	young	Qdpr	1.24258
StEt 15	3.58154039576409	young	Fas	1.41124
StEt 15	3.58154039576409	young	Erbb4	1.21867
StEt 15	3.58154039576409	young	Riox2	0.77466
StEt 15	3.58154039576409	young	Plxna4	0.08502
StEt 15	3.58154039576409	young	Epcam	-1.97328
StEt 15	3.58154039576409	young	Ccl3	-0.6241
StEt 15	3.58154039576409	young	Crim1	-1.79488
StEt 15	3.58154039576409	young	Vsig2	1.6057
StEt 15	3.58154039576409	young	Hgf	-0.98485
StEt 15	3.58154039576409	young	Sez6l2	2.63717
StEt 15	3.58154039576409	young	Il1a	1.89062
StEt 15	3.58154039576409	young	Il23r	4.4353
StEt 15	3.58154039576409	young	Dll1	1.28865
StEt 15	3.58154039576409	young	Ddah1	0.59293
StEt 15	3.58154039576409	young	Il10	-2.48976
StEt 15	3.58154039576409	young	Tnfrsf12a	1.58738
StEt 15	3.58154039576409	young	Acvrl1	0.13446
StEt 15	3.58154039576409	young	Lgmn	1.59397
StEt 15	3.58154039576409	young	Csf2	-3.72578
StEt 15	3.58154039576409	young	Cxcl9	1.22769
StEt 15	3.58154039576409	young	Map2k6	2.94015
StEt 15	3.58154039576409	young	Itgb1bp2	-6.00718
StEt 15	3.58154039576409	young	Il17f	-4.99899
StEt 15	3.58154039576409	young	Il1b	-3.71913
StEt 15	3.58154039576409	young	Casp3	3.44048
StEt 15	3.58154039576409	young	Apbb1ip	-3.66176
StEt 15	3.58154039576409	young	Wisp1	0.67045
StEt 15	3.58154039576409	young	Cdh6	0.6775
StEt 15	3.58154039576409	young	Pdgfb	3.20568
StEt 15	3.58154039576409	young	Igsf3	-0.67411
StEt 15	3.58154039576409	young	Tgfbr3	0.56113
StEt 15	3.58154039576409	young	Cxcl1	4.39103
StEt 15	3.58154039576409	young	Pak4	-3.49392
StEt 15	3.58154039576409	young	Cntn4	0.23279
StEt 15	3.58154039576409	young	Ghrl	2.43649
StEt 15	3.58154039576409	young	Lpl	-0.13293
StEt 15	3.58154039576409	young	Fstl3	1.5297
StEt 15	3.58154039576409	young	Dctn2	-0.85098
StEt 15	3.58154039576409	young	Il5	-3.56883
StEt 15	3.58154039576409	young	Eda2r	0.53301
StEt 15	3.58154039576409	young	Ntf3	-1.96347
StEt 15	3.58154039576409	young	Tnfsf12	-0.11235
StEt 15	3.58154039576409	young	Ccl20	4.66651
StEt 15	3.58154039576409	young	Fli1	-3.97135
StEt 15	3.58154039576409	young	Tpp1	3.516
StEt 15	3.58154039576409	young	Tnr	0.22242
StEt 15	3.58154039576409	young	Vegfd	-0.90966
StEt 15	3.58154039576409	young	Parp1	-2.07485
StEt 15	3.58154039576409	young	Tnf	-4.19379
StEt 16	3.17620808884795	old	Clmp	2.82676
StEt 16	3.17620808884795	old	Matn2	0.62573
StEt 16	3.17620808884795	old	Cpe	1.43515
StEt 16	3.17620808884795	old	Gcg	-1.31057
StEt 16	3.17620808884795	old	Gdnf	-4.31583
StEt 16	3.17620808884795	old	Yes1	-0.64189
StEt 16	3.17620808884795	old	Il17a	-1.33951
StEt 16	3.17620808884795	old	Foxo1	-1.69306
StEt 16	3.17620808884795	old	Tnfrsf11b	0.40356
StEt 16	3.17620808884795	old	Tgfb1	-0.84279
StEt 16	3.17620808884795	old	Pla2g4a	3.48254
StEt 16	3.17620808884795	old	Il6	-2.40517
StEt 16	3.17620808884795	old	Prdx5	0.23643
StEt 16	3.17620808884795	old	Tgfa	1.93992
StEt 16	3.17620808884795	old	Ccl5	-1.87645
StEt 16	3.17620808884795	old	Epo	0.88123
StEt 16	3.17620808884795	old	Axin1	-1.36937
StEt 16	3.17620808884795	old	Fst	1.79893
StEt 16	3.17620808884795	old	Rgma	-0.9519
StEt 16	3.17620808884795	old	Nadk	4.85924
StEt 16	3.17620808884795	old	Tnni3	9.07413
StEt 16	3.17620808884795	old	Notch3	1.47333
StEt 16	3.17620808884795	old	Snap29	2.96747
StEt 16	3.17620808884795	old	Cntn1	4.85464
StEt 16	3.17620808884795	old	Clstn2	-2.81804
StEt 16	3.17620808884795	old	S100a4	5.63482
StEt 16	3.17620808884795	old	Ca13	-3.50909
StEt 16	3.17620808884795	old	Mia	0.26627
StEt 16	3.17620808884795	old	Cant1	-1.92775
StEt 16	3.17620808884795	old	Kitlg	-5.35967
StEt 16	3.17620808884795	old	Gfra1	-1.95109
StEt 16	3.17620808884795	old	Ppp1r2	-1.31749

StEt	16	3.17620808884795	old	Adam23	-1.32982
StEt	16	3.17620808884795	old	Itgb6	-3.25049
StEt	16	3.17620808884795	old	Cyr61	0.44511
StEt	16	3.17620808884795	old	Dlk1	-0.04255
StEt	16	3.17620808884795	old	Ahr	-1.28792
StEt	16	3.17620808884795	old	Ccl2	5.93907
StEt	16	3.17620808884795	old	Eno2	-1.09411
StEt	16	3.17620808884795	old	Plin1	-1.42443
StEt	16	3.17620808884795	old	Wfikkn2	1.14403
StEt	16	3.17620808884795	old	Flrt2	-0.71025
StEt	16	3.17620808884795	old	Qdpr	1.56839
StEt	16	3.17620808884795	old	Fas	1.7234
StEt	16	3.17620808884795	old	Erbb4	-0.12386
StEt	16	3.17620808884795	old	Riox2	2.00207
StEt	16	3.17620808884795	old	Plxna4	-1.4075
StEt	16	3.17620808884795	old	Epcam	-1.78256
StEt	16	3.17620808884795	old	Ccl3	-1.46792
StEt	16	3.17620808884795	old	Crim1	-2.01568
StEt	16	3.17620808884795	old	Vsig2	0.5255
StEt	16	3.17620808884795	old	Hgf	-1.01868
StEt	16	3.17620808884795	old	Sez6l2	2.48626
StEt	16	3.17620808884795	old	Il1a	1.5802
StEt	16	3.17620808884795	old	Il23r	3.88767
StEt	16	3.17620808884795	old	Dll1	1.29747
StEt	16	3.17620808884795	old	Ddah1	1.06453
StEt	16	3.17620808884795	old	Il10	-1.75399
StEt	16	3.17620808884795	old	Tnfrsf12a	1.87133
StEt	16	3.17620808884795	old	Acvrl1	-0.11515
StEt	16	3.17620808884795	old	Lgmn	1.56272
StEt	16	3.17620808884795	old	Csf2	-3.74151
StEt	16	3.17620808884795	old	Cxcl9	1.48281
StEt	16	3.17620808884795	old	Map2k6	2.45369
StEt	16	3.17620808884795	old	Itgb1bp2	-6.29867
StEt	16	3.17620808884795	old	Il17f	-5.0789
StEt	16	3.17620808884795	old	Il1b	-2.35248
StEt	16	3.17620808884795	old	Casp3	3.14099
StEt	16	3.17620808884795	old	Apbb1ip	-3.64697
StEt	16	3.17620808884795	old	Wisp1	0.46391
StEt	16	3.17620808884795	old	Cdh6	0.56513
StEt	16	3.17620808884795	old	Pdgfb	1.42823
StEt	16	3.17620808884795	old	Igsf3	-0.51349
StEt	16	3.17620808884795	old	Tgfbr3	0.19333
StEt	16	3.17620808884795	old	Cxcl1	4.70569
StEt	16	3.17620808884795	old	Pak4	-3.43788
StEt	16	3.17620808884795	old	Cntn4	-0.12788
StEt	16	3.17620808884795	old	Ghrl	2.08605
StEt	16	3.17620808884795	old	Lpl	-0.42014
StEt	16	3.17620808884795	old	Fstl3	1.60957
StEt	16	3.17620808884795	old	Dctn2	-1.0302
StEt	16	3.17620808884795	old	Il5	-2.76451
StEt	16	3.17620808884795	old	Eda2r	0.19498
StEt	16	3.17620808884795	old	Ntf3	-2.76229
StEt	16	3.17620808884795	old	Tnfsf12	-0.37738
StEt	16	3.17620808884795	old	Ccl20	4.1692
StEt	16	3.17620808884795	old	Fli1	-4.18736
StEt	16	3.17620808884795	old	Tpp1	3.13
StEt	16	3.17620808884795	old	Tnr	-0.03498
StEt	16	3.17620808884795	old	Vegfd	-1.02405
StEt	16	3.17620808884795	old	Parp1	-1.63143
StEt	16	3.17620808884795	old	Tnf	-3.59716
StEt	17	2.75569781248927	young	Clmp	2.67601
StEt	17	2.75569781248927	young	Matn2	-0.30139
StEt	17	2.75569781248927	young	Cpe	1.57555
StEt	17	2.75569781248927	young	Gcg	-0.82814
StEt	17	2.75569781248927	young	Gdnf	-4.23716
StEt	17	2.75569781248927	young	Yes1	-0.29514
StEt	17	2.75569781248927	young	Il17a	-3.23551
StEt	17	2.75569781248927	young	Foxo1	-1.2004
StEt	17	2.75569781248927	young	Tnfrsf11b	-0.01264
StEt	17	2.75569781248927	young	Tgfb1	-0.92535
StEt	17	2.75569781248927	young	Pla2g4a	2.84689
StEt	17	2.75569781248927	young	Il6	-4.391
StEt	17	2.75569781248927	young	Prdx5	0.54677
StEt	17	2.75569781248927	young	Tgfa	1.6075
StEt	17	2.75569781248927	young	Ccl5	-1.73845
StEt	17	2.75569781248927	young	Epo	-1.32634
StEt	17	2.75569781248927	young	Axin1	-1.15838
StEt	17	2.75569781248927	young	Fst	2.97629
StEt	17	2.75569781248927	young	Rgma	-0.80848
StEt	17	2.75569781248927	young	Nadk	4.924
StEt	17	2.75569781248927	young	Tnni3	9.55432
StEt	17	2.75569781248927	young	Notch3	1.81221
StEt	17	2.75569781248927	young	Snap29	3.4811
StEt	17	2.75569781248927	young	Cntn1	4.84748
StEt	17	2.75569781248927	young	Clstn2	-2.99139
StEt	17	2.75569781248927	young	S100a4	5.37486
StEt	17	2.75569781248927	young	Ca13	-3.81724
StEt	17	2.75569781248927	young	Mia	0.76075
StEt	17	2.75569781248927	young	Cant1	-1.83355
StEt	17	2.75569781248927	young	Kitlg	-5.02471
StEt	17	2.75569781248927	young	Gfra1	-2.45777
StEt	17	2.75569781248927	young	Ppp1r2	-1.88073
StEt	17	2.75569781248927	young	Adam23	-1.11617
StEt	17	2.75569781248927	young	Itgb6	-3.23384
StEt	17	2.75569781248927	young	Cyr61	0.68959

StEt 17	2.75569781248927	young	Dlk1	0.02007
StEt 17	2.75569781248927	young	Ahr	-1.17671
StEt 17	2.75569781248927	young	Ccl2	5.1753
StEt 17	2.75569781248927	young	Eno2	-1.07994
StEt 17	2.75569781248927	young	Plin1	-0.86511
StEt 17	2.75569781248927	young	Wfikkn2	1.29358
StEt 17	2.75569781248927	young	Flrt2	-0.31224
StEt 17	2.75569781248927	young	Qdpr	1.97477
StEt 17	2.75569781248927	young	Fas	1.63466
StEt 17	2.75569781248927	young	Erbb4	0.91314
StEt 17	2.75569781248927	young	Riox2	1.91457
StEt 17	2.75569781248927	young	Plxna4	0.39264
StEt 17	2.75569781248927	young	Epcam	-1.47198
StEt 17	2.75569781248927	young	Ccl3	-1.08747
StEt 17	2.75569781248927	young	Crim1	-1.75324
StEt 17	2.75569781248927	young	Vsig2	1.30443
StEt 17	2.75569781248927	young	Hgf	-1.25183
StEt 17	2.75569781248927	young	Sez6l2	2.5657
StEt 17	2.75569781248927	young	Il1a	1.38093
StEt 17	2.75569781248927	young	Il23r	3.78734
StEt 17	2.75569781248927	young	Dll1	1.10531
StEt 17	2.75569781248927	young	Ddah1	1.79929
StEt 17	2.75569781248927	young	Il10	-2.56996
StEt 17	2.75569781248927	young	Tnfrsf12a	1.55918
StEt 17	2.75569781248927	young	Acvrl1	-0.05924
StEt 17	2.75569781248927	young	Lgmn	1.34954
StEt 17	2.75569781248927	young	Csf2	-3.46222
StEt 17	2.75569781248927	young	Cxcl9	0.90532
StEt 17	2.75569781248927	young	Map2k6	2.7436
StEt 17	2.75569781248927	young	Itgb1bp2	-6.62644
StEt 17	2.75569781248927	young	Il17f	-4.87324
StEt 17	2.75569781248927	young	Il1b	-3.68247
StEt 17	2.75569781248927	young	Casp3	3.43028
StEt 17	2.75569781248927	young	Apbb1ip	-3.44473
StEt 17	2.75569781248927	young	Wisp1	0.08146
StEt 17	2.75569781248927	young	Cdh6	-0.02025
StEt 17	2.75569781248927	young	Pdgfb	1.88864
StEt 17	2.75569781248927	young	Igsf3	-0.69553
StEt 17	2.75569781248927	young	Tgfbr3	0.29329
StEt 17	2.75569781248927	young	Cxcl1	4.98373
StEt 17	2.75569781248927	young	Pak4	-3.28974
StEt 17	2.75569781248927	young	Cntn4	0.03358
StEt 17	2.75569781248927	young	Ghrl	2.1185
StEt 17	2.75569781248927	young	Lpl	-0.10326
StEt 17	2.75569781248927	young	Fstl3	1.38326
StEt 17	2.75569781248927	young	Dctn2	-0.84627
StEt 17	2.75569781248927	young	Il5	-4.06547
StEt 17	2.75569781248927	young	Eda2r	0.36723
StEt 17	2.75569781248927	young	Ntf3	-2.3471
StEt 17	2.75569781248927	young	Tnfsf12	-0.22023
StEt 17	2.75569781248927	young	Ccl20	4.35187
StEt 17	2.75569781248927	young	Fli1	-3.14768
StEt 17	2.75569781248927	young	Tpp1	3.08026
StEt 17	2.75569781248927	young	Tnr	0.0678
StEt 17	2.75569781248927	young	Vegfd	-1.02023
StEt 17	2.75569781248927	young	Parp1	-1.74144
StEt 17	2.75569781248927	young	Tnf	-3.54456
StEt 18	3.46895310330584	young	Clmp	2.39444
StEt 18	3.46895310330584	young	Matn2	0.06651
StEt 18	3.46895310330584	young	Cpe	1.67969
StEt 18	3.46895310330584	young	Gcg	-1.14526
StEt 18	3.46895310330584	young	Gdnf	-4.55243
StEt 18	3.46895310330584	young	Yes1	0.64369
StEt 18	3.46895310330584	young	Il17a	-1.67799
StEt 18	3.46895310330584	young	Foxo1	-1.30451
StEt 18	3.46895310330584	young	Tnfrsf11b	-0.07676
StEt 18	3.46895310330584	young	Tgfb1	-0.75471
StEt 18	3.46895310330584	young	Pla2g4a	2.6647
StEt 18	3.46895310330584	young	Il6	-4.44505
StEt 18	3.46895310330584	young	Prdx5	0.77327
StEt 18	3.46895310330584	young	Tgfa	1.87761
StEt 18	3.46895310330584	young	Ccl5	-1.74286
StEt 18	3.46895310330584	young	Epo	-1.57996
StEt 18	3.46895310330584	young	Axin1	-1.08283
StEt 18	3.46895310330584	young	Fst	2.29019
StEt 18	3.46895310330584	young	Rgma	-0.6749
StEt 18	3.46895310330584	young	Nadk	5.45681
StEt 18	3.46895310330584	young	Tnni3	10.70942
StEt 18	3.46895310330584	young	Notch3	1.53103
StEt 18	3.46895310330584	young	Snap29	3.79708
StEt 18	3.46895310330584	young	Cntn1	4.6567
StEt 18	3.46895310330584	young	Clstn2	-3.30652
StEt 18	3.46895310330584	young	S100a4	5.23817
StEt 18	3.46895310330584	young	Ca13	-3.62412
StEt 18	3.46895310330584	young	Mia	0.62634
StEt 18	3.46895310330584	young	Cant1	-2.33264
StEt 18	3.46895310330584	young	Kitlg	-5.86649
StEt 18	3.46895310330584	young	Gfra1	-2.71566
StEt 18	3.46895310330584	young	Ppp1r2	-1.01124
StEt 18	3.46895310330584	young	Adam23	-1.77088
StEt 18	3.46895310330584	young	Itgb6	-3.6147
StEt 18	3.46895310330584	young	Cyr61	-0.26764
StEt 18	3.46895310330584	young	Dlk1	0.08545
StEt 18	3.46895310330584	young	Ahr	-0.34453
StEt 18	3.46895310330584	young	Ccl2	5.48848

StEt	18	3.46895310330584	young	Eno2	-1.07848
StEt	18	3.46895310330584	young	Plin1	-0.23163
StEt	18	3.46895310330584	young	Wfikkn2	0.84506
StEt	18	3.46895310330584	young	Flrt2	-0.81739
StEt	18	3.46895310330584	young	Qdpr	2.06663
StEt	18	3.46895310330584	young	Fas	1.81713
StEt	18	3.46895310330584	young	Erbb4	0.13726
StEt	18	3.46895310330584	young	Riox2	2.76034
StEt	18	3.46895310330584	young	Plxna4	-0.49652
StEt	18	3.46895310330584	young	Epcam	-1.95178
StEt	18	3.46895310330584	young	Ccl3	-1.37393
StEt	18	3.46895310330584	young	Crim1	-1.95477
StEt	18	3.46895310330584	young	Vsig2	0.98065
StEt	18	3.46895310330584	young	Hgf	-1.0559
StEt	18	3.46895310330584	young	Sez6l2	2.08424
StEt	18	3.46895310330584	young	Il1a	1.48315
StEt	18	3.46895310330584	young	Il23r	4.0391
StEt	18	3.46895310330584	young	Dll1	0.66742
StEt	18	3.46895310330584	young	Ddah1	2.05349
StEt	18	3.46895310330584	young	Il10	-2.94875
StEt	18	3.46895310330584	young	Tnfrsf12a	1.76239
StEt	18	3.46895310330584	young	Acvrl1	-0.20155
StEt	18	3.46895310330584	young	Lgmn	1.73389
StEt	18	3.46895310330584	young	Csf2	-3.57593
StEt	18	3.46895310330584	young	Cxcl9	1.12695
StEt	18	3.46895310330584	young	Map2k6	2.72744
StEt	18	3.46895310330584	young	Itgb1bp2	-5.4558
StEt	18	3.46895310330584	young	Il17f	-5.29642
StEt	18	3.46895310330584	young	Il1b	-3.47064
StEt	18	3.46895310330584	young	Casp3	3.36387
StEt	18	3.46895310330584	young	Apbb1ip	-3.75403
StEt	18	3.46895310330584	young	Wisp1	0.30854
StEt	18	3.46895310330584	young	Cdh6	0.36483
StEt	18	3.46895310330584	young	Pdgfb	2.1279
StEt	18	3.46895310330584	young	Igsf3	-0.90763
StEt	18	3.46895310330584	young	Tgfbr3	0.07754
StEt	18	3.46895310330584	young	Cxcl1	4.26128
StEt	18	3.46895310330584	young	Pak4	-3.7303
StEt	18	3.46895310330584	young	Cntn4	0.3533
StEt	18	3.46895310330584	young	Ghrl	2.28813
StEt	18	3.46895310330584	young	Lpl	-0.2883
StEt	18	3.46895310330584	young	Fstl3	0.93641
StEt	18	3.46895310330584	young	Dctn2	0.03818
StEt	18	3.46895310330584	young	Il5	-2.85121
StEt	18	3.46895310330584	young	Eda2r	0.1832
StEt	18	3.46895310330584	young	Ntf3	-2.49406
StEt	18	3.46895310330584	young	Tnfsf12	-0.18813
StEt	18	3.46895310330584	young	Ccl20	3.47752
StEt	18	3.46895310330584	young	Fli1	-4.09782
StEt	18	3.46895310330584	young	Tpp1	3.16856
StEt	18	3.46895310330584	young	Tnr	-0.54124
StEt	18	3.46895310330584	young	Vegfd	-1.11778
StEt	18	3.46895310330584	young	Parp1	0.17035
StEt	18	3.46895310330584	young	Tnf	-3.61256
StEt	20	3.30283747262421	young	Clmp	2.78969
StEt	20	3.30283747262421	young	Matn2	0.09881
StEt	20	3.30283747262421	young	Cpe	1.96049
StEt	20	3.30283747262421	young	Gcg	0.23129
StEt	20	3.30283747262421	young	Gdnf	-4.07882
StEt	20	3.30283747262421	young	Yes1	-1.3906
StEt	20	3.30283747262421	young	Il17a	-0.38706
StEt	20	3.30283747262421	young	Foxo1	-2.58384
StEt	20	3.30283747262421	young	Tnfrsf11b	0.06074
StEt	20	3.30283747262421	young	Tgfb1	-0.17747
StEt	20	3.30283747262421	young	Pla2g4a	3.88184
StEt	20	3.30283747262421	young	Il6	-4.65681
StEt	20	3.30283747262421	young	Prdx5	-0.38414
StEt	20	3.30283747262421	young	Tgfa	1.5871
StEt	20	3.30283747262421	young	Ccl5	-1.6084
StEt	20	3.30283747262421	young	Epo	-0.38291
StEt	20	3.30283747262421	young	Axin1	-1.61959
StEt	20	3.30283747262421	young	Fst	2.74758
StEt	20	3.30283747262421	young	Rgma	-1.15977
StEt	20	3.30283747262421	young	Nadk	4.06776
StEt	20	3.30283747262421	young	Tnni3	2.99306
StEt	20	3.30283747262421	young	Notch3	1.80533
StEt	20	3.30283747262421	young	Snap29	3.60267
StEt	20	3.30283747262421	young	Cntn1	5.01681
StEt	20	3.30283747262421	young	Clstn2	-2.87586
StEt	20	3.30283747262421	young	S100a4	5.38327
StEt	20	3.30283747262421	young	Ca13	-3.37088
StEt	20	3.30283747262421	young	Mia	0.66098
StEt	20	3.30283747262421	young	Cant1	-1.70559
StEt	20	3.30283747262421	young	Kitlg	-5.52263
StEt	20	3.30283747262421	young	Gfra1	-2.68936
StEt	20	3.30283747262421	young	Ppp1r2	-1.94478
StEt	20	3.30283747262421	young	Adam23	-1.9786
StEt	20	3.30283747262421	young	Itgb6	-3.19841
StEt	20	3.30283747262421	young	Cyr61	-0.17495
StEt	20	3.30283747262421	young	Dlk1	0.46379
StEt	20	3.30283747262421	young	Ahr	-2.4221
StEt	20	3.30283747262421	young	Ccl2	6.39438
StEt	20	3.30283747262421	young	Eno2	-1.11376
StEt	20	3.30283747262421	young	Plin1	-0.59959
StEt	20	3.30283747262421	young	Wfikkn2	1.83174

```
StEt 20  3.30283747262421  young  Flrt2  -0.35062
StEt 20  3.30283747262421  young  Qdpr  0.9492
StEt 20  3.30283747262421  young  Fas  1.28511
StEt 20  3.30283747262421  young  Erbb4  0.65431
StEt 20  3.30283747262421  young  Riox2  0.64305
StEt 20  3.30283747262421  young  Plxna4  1.81061
StEt 20  3.30283747262421  young  Epcam  -1.59221
StEt 20  3.30283747262421  young  Ccl3  -0.79394
StEt 20  3.30283747262421  young  Crim1  -2.24987
StEt 20  3.30283747262421  young  Vsig2  0.81426
StEt 20  3.30283747262421  young  Hgf  -1.01924
StEt 20  3.30283747262421  young  Sez6l2  2.18858
StEt 20  3.30283747262421  young  Il1a  2.53307
StEt 20  3.30283747262421  young  Il23r  4.41664
StEt 20  3.30283747262421  young  Dll1  1.09114
StEt 20  3.30283747262421  young  Ddah1  0.56448
StEt 20  3.30283747262421  young  Il10  -2.46202
StEt 20  3.30283747262421  young  Tnfrsf12a  1.46609
StEt 20  3.30283747262421  young  Acvrl1  -0.0148
StEt 20  3.30283747262421  young  Lgmn  1.70319
StEt 20  3.30283747262421  young  Csf2  -3.43217
StEt 20  3.30283747262421  young  Cxcl9  0.9618
StEt 20  3.30283747262421  young  Map2k6  3.00954
StEt 20  3.30283747262421  young  Itgb1bp2  -4.77398
StEt 20  3.30283747262421  young  Il17f  -5.0945
StEt 20  3.30283747262421  young  Il1b  -3.80929
StEt 20  3.30283747262421  young  Casp3  3.39295
StEt 20  3.30283747262421  young  Apbb1ip  -3.5575
StEt 20  3.30283747262421  young  Wisp1  0.62278
StEt 20  3.30283747262421  young  Cdh6  1.02528
StEt 20  3.30283747262421  young  Pdgfb  3.01943
StEt 20  3.30283747262421  young  Igsf3  -0.54035
StEt 20  3.30283747262421  young  Tgfbr3  0.36041
StEt 20  3.30283747262421  young  Cxcl1  4.39815
StEt 20  3.30283747262421  young  Pak4  -3.9549
StEt 20  3.30283747262421  young  Cntn4  1.02256
StEt 20  3.30283747262421  young  Ghrl  2.57272
StEt 20  3.30283747262421  young  Lpl  0.2592
StEt 20  3.30283747262421  young  Fstl3  1.37222
StEt 20  3.30283747262421  young  Dctn2  -0.45718
StEt 20  3.30283747262421  young  Il5  -2.39602
StEt 20  3.30283747262421  young  Eda2r  0.29734
StEt 20  3.30283747262421  young  Ntf3  -2.04424
StEt 20  3.30283747262421  young  Tnfsf12  -0.11364
StEt 20  3.30283747262421  young  Ccl20  3.71265
StEt 20  3.30283747262421  young  Fli1  -3.50509
StEt 20  3.30283747262421  young  Tpp1  3.18439
StEt 20  3.30283747262421  young  Tnr  -0.3688
StEt 20  3.30283747262421  young  Vegfd  -0.95293
StEt 20  3.30283747262421  young  Parp1  -1.37141
StEt 20  3.30283747262421  young  Tnf  -4.02772
;
RUN;

/*The code below performs a REML repeated measures mixed model approach to
protein expression profiles, which is much more powerful than conventional
univariate approaches, controls for sources of noise that such approaches
cannot, and provides a family level test that justifies the use of
Benjamini Hochberg.

Thus, the model builds an average protein expression profile, represented
by the "Protein" main effect and then tests the effects of other variables
on the profile as a whole with relevant interactions with "Protein". The analysis
generates contrasts and estimates as needed for further Benjamini-Hochberg correction
and graphing.

One important feature of REML Mixed Models is that Random effects (like "Subject")
are represented in a separate design matrix from fixed effects. This allows the
analysis to both use each subject as its own control, and to test continuous fixed effects
that would otherwise be collinear with subject (specifically Plasma GSH Level). This
is a well-documented advantage of these models over GLM and GLS solutions

We initially specified the model with JMP defaults of unbounded variance estimates
(specified by the NOBOUND option) and Kenward-Roger DF calculations (Specified by
the DDFM=KR option). However, note that REML can cause a variety of issues when model
fit is too good, as is the case with this data set. In particular, between-subject
variance was effectively explained by other factors in the model leading to model
separation and nonsensical error estimates. This was resolved by following standard
strategies to troubleshoot these issues (see Littell et al.; Kiernan et al.), specifically
using SAS's default of bounded variance components and its default of the DDFM=CONTAIN option.*/

PROC MIXED DATA=Olink_And_GSH_20241125 ALPHA=0.05;
CLASS SubjectID TwoGroups Protein;
MODEL  Norm_NPX =  Log_Total_GSH TwoGroups Log_Total_GSH*TwoGroups
Protein Log_Total_GSH*Protein TwoGroups*Protein Log_Total_GSH*TwoGroups*Protein/ DDFM=CONTAIN;
RANDOM SubjectID(TwoGroups ) /  ;

/*The following Contrasts test for differences in the regression slope between Plasma GSH Level and
protein expression for the two age groups. The resulting test values are used for further
Benjamini-Hochberg calculation of q-values in Excel*/

/*After the Contrasts for each protein, are corresponding Estimates, which give the regression
slope for each protein in each age group, including significance tests shown in the figure.
Note that the Contrasts are equal to the subtraction of the two estimates for each protein.*/
```

```
/*Note that for Contrasts and Estimates in PROC MIXED, omitted higher and lower order terms
are inferred as needed, rather than specifying a full matrix. We always confirm that
SAS has properly inferred the intended matrix by applying the "/E" option before building
the full list of Contrasts and/or Estimates.*/


Contrast "Acvrl1 Slope Diff" Log_Total_GSH*TwoGroups 1 -1 Log_Total_GSH*TwoGroups*Protein  1 0 0 0 0 0 0 0 0 0 0 0 0 0 0 0 0 0 0
0 0 0 0 0 0 0 0 0 0 0 0 0 0 0 0 0 0 0 0 0 0 0 0 0 0 0 0 0 0 0 0 0 0 0 0 0 0 0 0 0 0 0 0 0 0 0 0 0 0 0 0 0 0 0 0 0 0 0 0 0 0 0 0 0
0 0 0 0 0 0 0 0 -1 0 0 0 0 0 0 0 0 0 0 0 0 0 0 0 0 0 0 0 0 0 0 0 0 0 0 0 0 0 0 0 0 0 0 0 0 0 0 0 0 0 0 0 0 0 0 0 0 0 0 0 0 0 0 0
0 0 0 0 0 0 0 0 0 0 0 0 0 0 0 0 0 0 0 0 0 0 0 0 0 0 0 0 0 0 0 0 0 0 0 0;
Contrast "Adam23 Slope Diff" Log_Total_GSH*TwoGroups 1 -1 Log_Total_GSH*TwoGroups*Protein  0 1 0 0 0 0 0 0 0 0 0 0 0 0 0 0 0 0 0
0 0 0 0 0 0 0 0 0 0 0 0 0 0 0 0 0 0 0 0 0 0 0 0 0 0 0 0 0 0 0 0 0 0 0 0 0 0 0 0 0 0 0 0 0 0 0 0 0 0 0 0 0 0 0 0 0 0 0 0 0 0 0 0 0
0 0 0 0 0 0 0 0 0 -1 0 0 0 0 0 0 0 0 0 0 0 0 0 0 0 0 0 0 0 0 0 0 0 0 0 0 0 0 0 0 0 0 0 0 0 0 0 0 0 0 0 0 0 0 0 0 0 0 0 0 0 0 0 0
0 0 0 0 0 0 0 0 0 0 0 0 0 0 0 0 0 0 0 0 0 0 0 0 0 0 0 0 0 0 0 0 0 0 0 0;
Contrast "Ahr Slope Diff" Log_Total_GSH*TwoGroups 1 -1 Log_Total_GSH*TwoGroups*Protein  0 0 1 0 0 0 0 0 0 0 0 0 0 0 0 0 0 0 0 0 0
0 0 0 0 0 0 0 0 0 0 0 0 0 0 0 0 0 0 0 0 0 0 0 0 0 0 0 0 0 0 0 0 0 0 0 0 0 0 0 0 0 0 0 0 0 0 0 0 0 0 0 0 0 0 0 0 0 0 0 0 0 0 0 0 0
0 0 0 0 0 0 0 0 -1 0 0 0 0 0 0 0 0 0 0 0 0 0 0 0 0 0 0 0 0 0 0 0 0 0 0 0 0 0 0 0 0 0 0 0 0 0 0 0 0 0 0 0 0 0 0 0 0 0 0 0 0 0 0 0
0 0 0 0 0 0 0 0 0 0 0 0 0 0 0 0 0 0 0 0 0 0 0 0 0 0 0 0 0 0 0 0 0 0;
Contrast "Apbb1ip Slope Diff" Log_Total_GSH*TwoGroups 1 -1 Log_Total_GSH*TwoGroups*Protein  0 0 0 1 0 0 0 0 0 0 0 0 0 0 0 0 0 0 0
0 0 0 0 0 0 0 0 0 0 0 0 0 0 0 0 0 0 0 0 0 0 0 0 0 0 0 0 0 0 0 0 0 0 0 0 0 0 0 0 0 0 0 0 0 0 0 0 0 0 0 0 0 0 0 0 0 0 0 0 0 0 0 0 0
0 0 0 0 0 0 0 0 0 0 0 -1 0 0 0 0 0 0 0 0 0 0 0 0 0 0 0 0 0 0 0 0 0 0 0 0 0 0 0 0 0 0 0 0 0 0 0 0 0 0 0 0 0 0 0 0 0 0 0 0 0 0 0 0
0 0 0 0 0 0 0 0 0 0 0 0 0 0 0 0 0 0 0 0 0 0 0 0 0 0 0 0 0 0 0 0 0 0 0 0;
Contrast "Axin1 Slope Diff" Log_Total_GSH*TwoGroups 1 -1 Log_Total_GSH*TwoGroups*Protein  0 0 0 0 1 0 0 0 0 0 0 0 0 0 0 0 0 0 0 0
0 0 0 0 0 0 0 0 0 0 0 0 0 0 0 0 0 0 0 0 0 0 0 0 0 0 0 0 0 0 0 0 0 0 0 0 0 0 0 0 0 0 0 0 0 0 0 0 0 0 0 0 0 0 0 0 0 0 0 0 0 0 0 0 0
0 0 0 0 0 0 0 0 0 0 0 -1 0 0 0 0 0 0 0 0 0 0 0 0 0 0 0 0 0 0 0 0 0 0 0 0 0 0 0 0 0 0 0 0 0 0 0 0 0 0 0 0 0 0 0 0 0 0 0 0 0 0 0 0
0 0 0 0 0 0 0 0 0 0 0 0 0 0 0 0 0 0 0 0 0 0 0 0 0 0 0 0 0 0 0 0 0 0 0;
Contrast "Ca13 Slope Diff" Log_Total_GSH*TwoGroups 1 -1 Log_Total_GSH*TwoGroups*Protein  0 0 0 0 0 1 0 0 0 0 0 0 0 0 0 0 0 0 0 0
0 0 0 0 0 0 0 0 0 0 0 0 0 0 0 0 0 0 0 0 0 0 0 0 0 0 0 0 0 0 0 0 0 0 0 0 0 0 0 0 0 0 0 0 0 0 0 0 0 0 0 0 0 0 0 0 0 0 0 0 0 0 0 0 0
0 0 0 0 0 0 0 0 0 0 0 0 -1 0 0 0 0 0 0 0 0 0 0 0 0 0 0 0 0 0 0 0 0 0 0 0 0 0 0 0 0 0 0 0 0 0 0 0 0 0 0 0 0 0 0 0 0 0 0 0 0 0 0 0
0 0 0 0 0 0 0 0 0 0 0 0 0 0 0 0 0 0 0 0 0 0 0 0 0 0 0 0 0 0 0 0 0 0 0;
Contrast "Cant1 Slope Diff" Log_Total_GSH*TwoGroups 1 -1 Log_Total_GSH*TwoGroups*Protein  0 0 0 0 0 0 1 0 0 0 0 0 0 0 0 0 0 0 0 0
0 0 0 0 0 0 0 0 0 0 0 0 0 0 0 0 0 0 0 0 0 0 0 0 0 0 0 0 0 0 0 0 0 0 0 0 0 0 0 0 0 0 0 0 0 0 0 0 0 0 0 0 0 0 0 0 0 0 0 0 0 0 0 0 0
0 0 0 0 0 0 0 0 0 0 0 0 0 -1 0 0 0 0 0 0 0 0 0 0 0 0 0 0 0 0 0 0 0 0 0 0 0 0 0 0 0 0 0 0 0 0 0 0 0 0 0 0 0 0 0 0 0 0 0 0 0 0 0 0
0 0 0 0 0 0 0 0 0 0 0 0 0 0 0 0 0 0 0 0 0 0 0 0 0 0 0 0 0 0 0 0 0 0 0;
Contrast "Casp3 Slope Diff" Log_Total_GSH*TwoGroups 1 -1 Log_Total_GSH*TwoGroups*Protein  0 0 0 0 0 0 0 1 0 0 0 0 0 0 0 0 0 0 0 0
0 0 0 0 0 0 0 0 0 0 0 0 0 0 0 0 0 0 0 0 0 0 0 0 0 0 0 0 0 0 0 0 0 0 0 0 0 0 0 0 0 0 0 0 0 0 0 0 0 0 0 0 0 0 0 0 0 0 0 0 0 0 0 0 0
0 0 0 0 0 0 0 0 0 0 0 0 0 0 -1 0 0 0 0 0 0 0 0 0 0 0 0 0 0 0 0 0 0 0 0 0 0 0 0 0 0 0 0 0 0 0 0 0 0 0 0 0 0 0 0 0 0 0 0 0 0 0 0 0
0 0 0 0 0 0 0 0 0 0 0 0 0 0 0 0 0 0 0 0 0 0 0 0 0 0 0 0 0 0 0 0 0 0 0;
Contrast "Ccl2 Slope Diff" Log_Total_GSH*TwoGroups 1 -1 Log_Total_GSH*TwoGroups*Protein  0 0 0 0 0 0 0 0 1 0 0 0 0 0 0 0 0 0 0 0
0 0 0 0 0 0 0 0 0 0 0 0 0 0 0 0 0 0 0 0 0 0 0 0 0 0 0 0 0 0 0 0 0 0 0 0 0 0 0 0 0 0 0 0 0 0 0 0 0 0 0 0 0 0 0 0 0 0 0 0 0 0 0 0 0
0 0 0 0 0 0 0 0 0 0 0 0 0 0 0 -1 0 0 0 0 0 0 0 0 0 0 0 0 0 0 0 0 0 0 0 0 0 0 0 0 0 0 0 0 0 0 0 0 0 0 0 0 0 0 0 0 0 0 0 0 0 0 0 0
0 0 0 0 0 0 0 0 0 0 0 0 0 0 0 0 0 0 0 0 0 0 0 0 0 0 0 0 0 0 0 0 0 0 0;
Contrast "Ccl20 Slope Diff" Log_Total_GSH*TwoGroups 1 -1 Log_Total_GSH*TwoGroups*Protein  0 0 0 0 0 0 0 0 0 1 0 0 0 0 0 0 0 0 0 0
0 0 0 0 0 0 0 0 0 0 0 0 0 0 0 0 0 0 0 0 0 0 0 0 0 0 0 0 0 0 0 0 0 0 0 0 0 0 0 0 0 0 0 0 0 0 0 0 0 0 0 0 0 0 0 0 0 0 0 0 0 0 0 0 0
0 0 0 0 0 0 0 0 0 0 0 0 0 0 0 0 -1 0 0 0 0 0 0 0 0 0 0 0 0 0 0 0 0 0 0 0 0 0 0 0 0 0 0 0 0 0 0 0 0 0 0 0 0 0 0 0 0 0 0 0 0 0 0 0
0 0 0 0 0 0 0 0 0 0 0 0 0 0 0 0 0 0 0 0 0 0 0 0 0 0 0 0 0 0 0 0 0 0 0;
Contrast "Ccl3 Slope Diff" Log_Total_GSH*TwoGroups 1 -1 Log_Total_GSH*TwoGroups*Protein  0 0 0 0 0 0 0 0 0 0 1 0 0 0 0 0 0 0 0 0
0 0 0 0 0 0 0 0 0 0 0 0 0 0 0 0 0 0 0 0 0 0 0 0 0 0 0 0 0 0 0 0 0 0 0 0 0 0 0 0 0 0 0 0 0 0 0 0 0 0 0 0 0 0 0 0 0 0 0 0 0 0 0 0 0
0 0 0 0 0 0 0 0 0 0 0 0 0 0 0 0 0 -1 0 0 0 0 0 0 0 0 0 0 0 0 0 0 0 0 0 0 0 0 0 0 0 0 0 0 0 0 0 0 0 0 0 0 0 0 0 0 0 0 0 0 0 0 0 0
0 0 0 0 0 0 0 0 0 0 0 0 0 0 0 0 0 0 0 0 0 0 0 0 0 0 0 0 0 0 0 0 0 0 0;
Contrast "Ccl5 Slope Diff" Log_Total_GSH*TwoGroups 1 -1 Log_Total_GSH*TwoGroups*Protein  0 0 0 0 0 0 0 0 0 0 0 1 0 0 0 0 0 0 0 0
0 0 0 0 0 0 0 0 0 0 0 0 0 0 0 0 0 0 0 0 0 0 0 0 0 0 0 0 0 0 0 0 0 0 0 0 0 0 0 0 0 0 0 0 0 0 0 0 0 0 0 0 0 0 0 0 0 0 0 0 0 0 0 0 0
0 0 0 0 0 0 0 0 0 0 0 0 0 0 0 0 0 0 -1 0 0 0 0 0 0 0 0 0 0 0 0 0 0 0 0 0 0 0 0 0 0 0 0 0 0 0 0 0 0 0 0 0 0 0 0 0 0 0 0 0 0 0 0 0
0 0 0 0 0 0 0 0 0 0 0 0 0 0 0 0 0 0 0 0 0 0 0 0 0 0 0 0 0 0 0 0 0 0 0;
Contrast "Cdh6 Slope Diff" Log_Total_GSH*TwoGroups 1 -1 Log_Total_GSH*TwoGroups*Protein  0 0 0 0 0 0 0 0 0 0 0 0 1 0 0 0 0 0 0 0
0 0 0 0 0 0 0 0 0 0 0 0 0 0 0 0 0 0 0 0 0 0 0 0 0 0 0 0 0 0 0 0 0 0 0 0 0 0 0 0 0 0 0 0 0 0 0 0 0 0 0 0 0 0 0 0 0 0 0 0 0 0 0 0 0
0 0 0 0 0 0 0 0 0 0 0 0 0 0 0 0 0 0 0 -1 0 0 0 0 0 0 0 0 0 0 0 0 0 0 0 0 0 0 0 0 0 0 0 0 0 0 0 0 0 0 0 0 0 0 0 0 0 0 0 0 0 0 0 0
0 0 0 0 0 0 0 0 0 0 0 0 0 0 0 0 0 0 0 0 0 0 0 0 0 0 0 0 0 0 0 0 0 0 0;
Contrast "Clmp Slope Diff" Log_Total_GSH*TwoGroups 1 -1 Log_Total_GSH*TwoGroups*Protein  0 0 0 0 0 0 0 0 0 0 0 0 0 1 0 0 0 0 0 0
0 0 0 0 0 0 0 0 0 0 0 0 0 0 0 0 0 0 0 0 0 0 0 0 0 0 0 0 0 0 0 0 0 0 0 0 0 0 0 0 0 0 0 0 0 0 0 0 0 0 0 0 0 0 0 0 0 0 0 0 0 0 0 0 0
0 0 0 0 0 0 0 0 0 0 0 0 0 0 0 0 0 0 0 0 -1 0 0 0 0 0 0 0 0 0 0 0 0 0 0 0 0 0 0 0 0 0 0 0 0 0 0 0 0 0 0 0 0 0 0 0 0 0 0 0 0 0 0 0
0 0 0 0 0 0 0 0 0 0 0 0 0 0 0 0 0 0 0 0 0 0 0 0 0 0 0 0 0 0 0 0 0 0 0;
Contrast "Clstn2 Slope Diff" Log_Total_GSH*TwoGroups 1 -1 Log_Total_GSH*TwoGroups*Protein  0 0 0 0 0 0 0 0 0 0 0 0 0 0 1 0 0 0 0
0 0 0 0 0 0 0 0 0 0 0 0 0 0 0 0 0 0 0 0 0 0 0 0 0 0 0 0 0 0 0 0 0 0 0 0 0 0 0 0 0 0 0 0 0 0 0 0 0 0 0 0 0 0 0 0 0 0 0 0 0 0 0 0 0
0 0 0 0 0 0 0 0 0 0 0 0 0 0 0 0 0 0 0 0 0 0 -1 0 0 0 0 0 0 0 0 0 0 0 0 0 0 0 0 0 0 0 0 0 0 0 0 0 0 0 0 0 0 0 0 0 0 0 0 0 0 0 0 0
0 0 0 0 0 0 0 0 0 0 0 0 0 0 0 0 0 0 0 0 0 0 0 0 0 0 0 0 0 0 0 0 0 0 0 0;
Contrast "Cntn1 Slope Diff" Log_Total_GSH*TwoGroups 1 -1 Log_Total_GSH*TwoGroups*Protein  0 0 0 0 0 0 0 0 0 0 0 0 0 0 0 1 0 0 0 0
0 0 0 0 0 0 0 0 0 0 0 0 0 0 0 0 0 0 0 0 0 0 0 0 0 0 0 0 0 0 0 0 0 0 0 0 0 0 0 0 0 0 0 0 0 0 0 0 0 0 0 0 0 0 0 0 0 0 0 0 0 0 0 0 0
0 0 0 0 0 0 0 0 0 0 0 0 0 0 0 0 0 0 0 0 0 0 -1 0 0 0 0 0 0 0 0 0 0 0 0 0 0 0 0 0 0 0 0 0 0 0 0 0 0 0 0 0 0 0 0 0 0 0 0 0 0 0 0 0
0 0 0 0 0 0 0 0 0 0 0 0 0 0 0 0 0 0 0 0 0 0 0 0 0 0 0 0 0 0 0 0 0 0 0;
Contrast "Cntn4 Slope Diff" Log_Total_GSH*TwoGroups 1 -1 Log_Total_GSH*TwoGroups*Protein  0 0 0 0 0 0 0 0 0 0 0 0 0 0 0 0 1 0 0 0
0 0 0 0 0 0 0 0 0 0 0 0 0 0 0 0 0 0 0 0 0 0 0 0 0 0 0 0 0 0 0 0 0 0 0 0 0 0 0 0 0 0 0 0 0 0 0 0 0 0 0 0 0 0 0 0 0 0 0 0 0 0 0 0 0
0 0 0 0 0 0 0 0 0 0 0 0 0 0 0 0 0 0 0 0 0 0 0 -1 0 0 0 0 0 0 0 0 0 0 0 0 0 0 0 0 0 0 0 0 0 0 0 0 0 0 0 0 0 0 0 0 0 0 0 0 0 0 0 0
0 0 0 0 0 0 0 0 0 0 0 0 0 0 0 0 0 0 0 0 0 0 0 0 0 0 0 0 0 0 0 0 0 0 0;
Contrast "Cpe Slope Diff" Log_Total_GSH*TwoGroups 1 -1 Log_Total_GSH*TwoGroups*Protein  0 0 0 0 0 0 0 0 0 0 0 0 0 0 0 0 0 1 0 0 0
0 0 0 0 0 0 0 0 0 0 0 0 0 0 0 0 0 0 0 0 0 0 0 0 0 0 0 0 0 0 0 0 0 0 0 0 0 0 0 0 0 0 0 0 0 0 0 0 0 0 0 0 0 0 0 0 0 0 0 0 0 0 0 0 0
0 0 0 0 0 0 0 0 0 0 0 0 0 0 0 0 0 0 0 0 0 0 0 -1 0 0 0 0 0 0 0 0 0 0 0 0 0 0 0 0 0 0 0 0 0 0 0 0 0 0 0 0 0 0 0 0 0 0 0 0 0 0 0 0
0 0 0 0 0 0 0 0 0 0 0 0 0 0 0 0 0 0 0 0 0 0 0 0 0 0 0 0 0 0 0 0 0 0;
Contrast "Crim1 Slope Diff" Log_Total_GSH*TwoGroups 1 -1 Log_Total_GSH*TwoGroups*Protein  0 0 0 0 0 0 0 0 0 0 0 0 0 0 0 0 0 0 1 0
0 0 0 0 0 0 0 0 0 0 0 0 0 0 0 0 0 0 0 0 0 0 0 0 0 0 0 0 0 0 0 0 0 0 0 0 0 0 0 0 0 0 0 0 0 0 0 0 0 0 0 0 0 0 0 0 0 0 0 0 0 0 0 0 0
0 0 0 0 0 0 0 0 0 0 0 0 0 0 0 0 0 0 0 0 0 0 0 0 0 -1 0 0 0 0 0 0 0 0 0 0 0 0 0 0 0 0 0 0 0 0 0 0 0 0 0 0 0 0 0 0 0 0 0 0 0 0 0 0
0 0 0 0 0 0 0 0 0 0 0 0 0 0 0 0 0 0 0 0 0 0 0 0 0 0 0 0 0 0 0 0 0 0 0;
Contrast "Csf2 Slope Diff" Log_Total_GSH*TwoGroups 1 -1 Log_Total_GSH*TwoGroups*Protein  0 0 0 0 0 0 0 0 0 0 0 0 0 0 0 0 0 0 0 1
0 0 0 0 0 0 0 0 0 0 0 0 0 0 0 0 0 0 0 0 0 0 0 0 0 0 0 0 0 0 0 0 0 0 0 0 0 0 0 0 0 0 0 0 0 0 0 0 0 0 0 0 0 0 0 0 0 0 0 0 0 0 0 0 0
0 0 0 0 0 0 0 0 0 0 0 0 0 0 0 0 0 0 0 0 0 0 0 0 0 0 -1 0 0 0 0 0 0 0 0 0 0 0 0 0 0 0 0 0 0 0 0 0 0 0 0 0 0 0 0 0 0 0 0 0 0 0 0 0
0 0 0 0 0 0 0 0 0 0 0 0 0 0 0 0 0 0 0 0 0 0 0 0 0 0 0 0 0 0 0 0 0 0 0;
Contrast "Cxcl1 Slope Diff" Log_Total_GSH*TwoGroups 1 -1 Log_Total_GSH*TwoGroups*Protein  0 0 0 0 0 0 0 0 0 0 0 0 0 0 0 0 0 0 0 0
1 0 0 0 0 0 0 0 0 0 0 0 0 0 0 0 0 0 0 0 0 0 0 0 0 0 0 0 0 0 0 0 0 0 0 0 0 0 0 0 0 0 0 0 0 0 0 0 0 0 0 0 0 0 0 0 0 0 0 0 0 0 0 0 0
0 0 0 0 0 0 0 0 0 0 0 0 0 0 0 0 0 0 0 0 0 0 0 0 0 0 0 -1 0 0 0 0 0 0 0 0 0 0 0 0 0 0 0 0 0 0 0 0 0 0 0 0 0 0 0 0 0 0 0 0 0 0 0 0
0 0 0 0 0 0 0 0 0 0 0 0 0 0 0 0 0 0 0 0 0 0 0 0 0 0 0 0 0 0 0 0 0 0 0;
Contrast "Cxcl9 Slope Diff" Log_Total_GSH*TwoGroups 1 -1 Log_Total_GSH*TwoGroups*Protein  0 0 0 0 0 0 0 0 0 0 0 0 0 0 0 0 0 0 0 0
0 1 0 0 0 0 0 0 0 0 0 0 0 0 0 0 0 0 0 0 0 0 0 0 0 0 0 0 0 0 0 0 0 0 0 0 0 0 0 0 0 0 0 0 0 0 0 0 0 0 0 0 0 0 0 0 0 0 0 0 0 0 0 0 0
0 0 0 0 0 0 0 0 0 0 0 0 0 0 0 0 0 0 0 0 0 0 0 0 0 0 0 0 -1 0 0 0 0 0 0 0 0 0 0 0 0 0 0 0 0 0 0 0 0 0 0 0 0 0 0 0 0 0 0 0 0 0 0 0
0 0 0 0 0 0 0 0 0 0 0 0 0 0 0 0 0 0 0 0 0 0 0 0 0 0 0 0 0 0 0 0 0 0 0;
```

```
Contrast "Cyr61 Slope Diff" Log_Total_GSH*TwoGroups 1 -1 Log_Total_GSH*TwoGroups*Protein  0 0 0 0 0 0 0 0 0 0 0 0 0 0 0 0 0 0 0 0
0 0 1 0 0 0 0 0 0 0 0 0 0 0 0 0 0 0 0 0 0 0 0 0 0 0 0 0 0 0 0 0 0 0 0 0 0 0 0 0 0 0 0 0 0 0 0 0 0 0 0 0 0 0 0 0 0 0 0 0 0 0 0 0 0
0 0 0 0 0 0 0 0 0 0 0 0 0 0 0 0 0 0 0 0 0 0 0 0 0 0 0 0 0 -1 0 0 0 0 0 0 0 0 0 0 0 0 0 0 0 0 0 0 0 0 0 0 0 0 0 0 0 0 0 0 0 0 0 0
0 0 0 0 0 0 0 0 0 0 0 0 0 0 0 0 0 0 0 0 0 0 0 0 0 0 0 0 0 0 0 0 0 0 0;
Contrast "Dctn2 Slope Diff" Log_Total_GSH*TwoGroups 1 -1 Log_Total_GSH*TwoGroups*Protein  0 0 0 0 0 0 0 0 0 0 0 0 0 0 0 0 0 0 0 0
0 0 0 1 0 0 0 0 0 0 0 0 0 0 0 0 0 0 0 0 0 0 0 0 0 0 0 0 0 0 0 0 0 0 0 0 0 0 0 0 0 0 0 0 0 0 0 0 0 0 0 0 0 0 0 0 0 0 0 0 0 0 0 0 0
0 0 0 0 0 0 0 0 0 0 0 0 0 0 0 0 0 0 0 0 0 0 0 0 0 0 0 0 0 0 -1 0 0 0 0 0 0 0 0 0 0 0 0 0 0 0 0 0 0 0 0 0 0 0 0 0 0 0 0 0 0 0 0 0
0 0 0 0 0 0 0 0 0 0 0 0 0 0 0 0 0 0 0 0 0 0 0 0 0 0 0 0 0 0 0 0 0 0 0;
Contrast "Ddah1 Slope Diff" Log_Total_GSH*TwoGroups 1 -1 Log_Total_GSH*TwoGroups*Protein  0 0 0 0 0 0 0 0 0 0 0 0 0 0 0 0 0 0 0 0
0 0 0 0 1 0 0 0 0 0 0 0 0 0 0 0 0 0 0 0 0 0 0 0 0 0 0 0 0 0 0 0 0 0 0 0 0 0 0 0 0 0 0 0 0 0 0 0 0 0 0 0 0 0 0 0 0 0 0 0 0 0 0 0 0
0 0 0 0 0 0 0 0 0 0 0 0 0 0 0 0 0 0 0 0 0 0 0 0 0 0 0 0 0 0 0 -1 0 0 0 0 0 0 0 0 0 0 0 0 0 0 0 0 0 0 0 0 0 0 0 0 0 0 0 0 0 0 0 0
0 0 0 0 0 0 0 0 0 0 0 0 0 0 0 0 0 0 0 0 0 0 0 0 0 0 0 0 0 0 0 0 0 0 0;
Contrast "Dlk1 Slope Diff" Log_Total_GSH*TwoGroups 1 -1 Log_Total_GSH*TwoGroups*Protein  0 0 0 0 0 0 0 0 0 0 0 0 0 0 0 0 0 0 0 0
0 0 0 0 0 1 0 0 0 0 0 0 0 0 0 0 0 0 0 0 0 0 0 0 0 0 0 0 0 0 0 0 0 0 0 0 0 0 0 0 0 0 0 0 0 0 0 0 0 0 0 0 0 0 0 0 0 0 0 0 0 0 0 0 0
0 0 0 0 0 0 0 0 0 0 0 0 0 0 0 0 0 0 0 0 0 0 0 0 0 0 0 0 0 0 0 0 -1 0 0 0 0 0 0 0 0 0 0 0 0 0 0 0 0 0 0 0 0 0 0 0 0 0 0 0 0 0 0 0
0 0 0 0 0 0 0 0 0 0 0 0 0 0 0 0 0 0 0 0 0 0 0 0 0 0 0 0 0 0 0 0 0 0 0;
Contrast "Dll1 Slope Diff" Log_Total_GSH*TwoGroups 1 -1 Log_Total_GSH*TwoGroups*Protein  0 0 0 0 0 0 0 0 0 0 0 0 0 0 0 0 0 0 0 0
0 0 0 0 0 0 1 0 0 0 0 0 0 0 0 0 0 0 0 0 0 0 0 0 0 0 0 0 0 0 0 0 0 0 0 0 0 0 0 0 0 0 0 0 0 0 0 0 0 0 0 0 0 0 0 0 0 0 0 0 0 0 0 0 0
0 0 0 0 0 0 0 0 0 0 0 0 0 0 0 0 0 0 0 0 0 0 0 0 0 0 0 0 0 0 0 0 0 -1 0 0 0 0 0 0 0 0 0 0 0 0 0 0 0 0 0 0 0 0 0 0 0 0 0 0 0 0 0 0
0 0 0 0 0 0 0 0 0 0 0 0 0 0 0 0 0 0 0 0 0 0 0 0 0 0 0 0 0 0 0 0 0 0 0;
Contrast "Eda2r Slope Diff" Log_Total_GSH*TwoGroups 1 -1 Log_Total_GSH*TwoGroups*Protein  0 0 0 0 0 0 0 0 0 0 0 0 0 0 0 0 0 0 0 0
0 0 0 0 0 0 0 1 0 0 0 0 0 0 0 0 0 0 0 0 0 0 0 0 0 0 0 0 0 0 0 0 0 0 0 0 0 0 0 0 0 0 0 0 0 0 0 0 0 0 0 0 0 0 0 0 0 0 0 0 0 0 0 0 0
0 0 0 0 0 0 0 0 0 0 0 0 0 0 0 0 0 0 0 0 0 0 0 0 0 0 0 0 0 0 0 0 0 0 -1 0 0 0 0 0 0 0 0 0 0 0 0 0 0 0 0 0 0 0 0 0 0 0 0 0 0 0 0 0
0 0 0 0 0 0 0 0 0 0 0 0 0 0 0 0 0 0 0 0 0 0 0 0 0 0 0 0 0 0 0 0 0 0 0;
Contrast "Eno2 Slope Diff" Log_Total_GSH*TwoGroups 1 -1 Log_Total_GSH*TwoGroups*Protein  0 0 0 0 0 0 0 0 0 0 0 0 0 0 0 0 0 0 0 0
0 0 0 0 0 0 0 0 1 0 0 0 0 0 0 0 0 0 0 0 0 0 0 0 0 0 0 0 0 0 0 0 0 0 0 0 0 0 0 0 0 0 0 0 0 0 0 0 0 0 0 0 0 0 0 0 0 0 0 0 0 0 0 0 0
0 0 0 0 0 0 0 0 0 0 0 0 0 0 0 0 0 0 0 0 0 0 0 0 0 0 0 0 0 0 0 0 0 0 0 -1 0 0 0 0 0 0 0 0 0 0 0 0 0 0 0 0 0 0 0 0 0 0 0 0 0 0 0 0
0 0 0 0 0 0 0 0 0 0 0 0 0 0 0 0 0 0 0 0 0 0 0 0 0 0 0 0 0 0 0 0 0 0 0;
Contrast "Epcam Slope Diff" Log_Total_GSH*TwoGroups 1 -1 Log_Total_GSH*TwoGroups*Protein  0 0 0 0 0 0 0 0 0 0 0 0 0 0 0 0 0 0 0 0
0 0 0 0 0 0 0 0 0 1 0 0 0 0 0 0 0 0 0 0 0 0 0 0 0 0 0 0 0 0 0 0 0 0 0 0 0 0 0 0 0 0 0 0 0 0 0 0 0 0 0 0 0 0 0 0 0 0 0 0 0 0 0 0 0
0 0 0 0 0 0 0 0 0 0 0 0 0 0 0 0 0 0 0 0 0 0 0 0 0 0 0 0 0 0 0 0 0 0 0 0 -1 0 0 0 0 0 0 0 0 0 0 0 0 0 0 0 0 0 0 0 0 0 0 0 0 0 0 0
0 0 0 0 0 0 0 0 0 0 0 0 0 0 0 0 0 0 0 0 0 0 0 0 0 0 0 0 0 0 0 0 0 0 0;
Contrast "Epo Slope Diff" Log_Total_GSH*TwoGroups 1 -1 Log_Total_GSH*TwoGroups*Protein  0 0 0 0 0 0 0 0 0 0 0 0 0 0 0 0 0 0 0 0 0
0 0 0 0 0 0 0 0 0 1 0 0 0 0 0 0 0 0 0 0 0 0 0 0 0 0 0 0 0 0 0 0 0 0 0 0 0 0 0 0 0 0 0 0 0 0 0 0 0 0 0 0 0 0 0 0 0 0 0 0 0 0 0 0 0
0 0 0 0 0 0 0 0 0 0 0 0 0 0 0 0 0 0 0 0 0 0 0 0 0 0 0 0 0 0 0 0 0 0 0 0 -1 0 0 0 0 0 0 0 0 0 0 0 0 0 0 0 0 0 0 0 0 0 0 0 0 0 0 0
0 0 0 0 0 0 0 0 0 0 0 0 0 0 0 0 0 0 0 0 0 0 0 0 0 0 0 0 0 0 0 0 0 0;
Contrast "Erbb4 Slope Diff" Log_Total_GSH*TwoGroups 1 -1 Log_Total_GSH*TwoGroups*Protein  0 0 0 0 0 0 0 0 0 0 0 0 0 0 0 0 0 0 0 0
0 0 0 0 0 0 0 0 0 0 0 1 0 0 0 0 0 0 0 0 0 0 0 0 0 0 0 0 0 0 0 0 0 0 0 0 0 0 0 0 0 0 0 0 0 0 0 0 0 0 0 0 0 0 0 0 0 0 0 0 0 0 0 0 0
0 0 0 0 0 0 0 0 0 0 0 0 0 0 0 0 0 0 0 0 0 0 0 0 0 0 0 0 0 0 0 0 0 0 0 0 0 0 -1 0 0 0 0 0 0 0 0 0 0 0 0 0 0 0 0 0 0 0 0 0 0 0 0 0
0 0 0 0 0 0 0 0 0 0 0 0 0 0 0 0 0 0 0 0 0 0 0 0 0 0 0 0 0 0 0 0 0 0 0;
Contrast "Fas Slope Diff" Log_Total_GSH*TwoGroups 1 -1 Log_Total_GSH*TwoGroups*Protein  0 0 0 0 0 0 0 0 0 0 0 0 0 0 0 0 0 0 0 0 0
0 0 0 0 0 0 0 0 0 0 0 1 0 0 0 0 0 0 0 0 0 0 0 0 0 0 0 0 0 0 0 0 0 0 0 0 0 0 0 0 0 0 0 0 0 0 0 0 0 0 0 0 0 0 0 0 0 0 0 0 0 0 0 0 0
0 0 0 0 0 0 0 0 0 0 0 0 0 0 0 0 0 0 0 0 0 0 0 0 0 0 0 0 0 0 0 0 0 0 0 0 0 0 -1 0 0 0 0 0 0 0 0 0 0 0 0 0 0 0 0 0 0 0 0 0 0 0 0 0
0 0 0 0 0 0 0 0 0 0 0 0 0 0 0 0 0 0 0 0 0 0 0 0 0 0 0 0 0 0 0 0 0 0;
Contrast "Fli1 Slope Diff" Log_Total_GSH*TwoGroups 1 -1 Log_Total_GSH*TwoGroups*Protein  0 0 0 0 0 0 0 0 0 0 0 0 0 0 0 0 0 0 0 0
0 0 0 0 0 0 0 0 0 0 0 0 0 1 0 0 0 0 0 0 0 0 0 0 0 0 0 0 0 0 0 0 0 0 0 0 0 0 0 0 0 0 0 0 0 0 0 0 0 0 0 0 0 0 0 0 0 0 0 0 0 0 0 0 0
0 0 0 0 0 0 0 0 0 0 0 0 0 0 0 0 0 0 0 0 0 0 0 0 0 0 0 0 0 0 0 0 0 0 0 0 0 0 0 0 -1 0 0 0 0 0 0 0 0 0 0 0 0 0 0 0 0 0 0 0 0 0 0 0
0 0 0 0 0 0 0 0 0 0 0 0 0 0 0 0 0 0 0 0 0 0 0 0 0 0 0 0 0 0 0 0 0 0 0;
Contrast "Flrt2 Slope Diff" Log_Total_GSH*TwoGroups 1 -1 Log_Total_GSH*TwoGroups*Protein  0 0 0 0 0 0 0 0 0 0 0 0 0 0 0 0 0 0 0 0
0 0 0 0 0 0 0 0 0 0 0 0 0 0 1 0 0 0 0 0 0 0 0 0 0 0 0 0 0 0 0 0 0 0 0 0 0 0 0 0 0 0 0 0 0 0 0 0 0 0 0 0 0 0 0 0 0 0 0 0 0 0 0 0 0
0 0 0 0 0 0 0 0 0 0 0 0 0 0 0 0 0 0 0 0 0 0 0 0 0 0 0 0 0 0 0 0 0 0 0 0 0 0 0 0 0 -1 0 0 0 0 0 0 0 0 0 0 0 0 0 0 0 0 0 0 0 0 0 0
0 0 0 0 0 0 0 0 0 0 0 0 0 0 0 0 0 0 0 0 0 0 0 0 0 0 0 0 0 0 0 0 0 0 0;
Contrast "Foxo1 Slope Diff" Log_Total_GSH*TwoGroups 1 -1 Log_Total_GSH*TwoGroups*Protein  0 0 0 0 0 0 0 0 0 0 0 0 0 0 0 0 0 0 0 0
0 0 0 0 0 0 0 0 0 0 0 0 0 0 0 1 0 0 0 0 0 0 0 0 0 0 0 0 0 0 0 0 0 0 0 0 0 0 0 0 0 0 0 0 0 0 0 0 0 0 0 0 0 0 0 0 0 0 0 0 0 0 0 0 0
0 0 0 0 0 0 0 0 0 0 0 0 0 0 0 0 0 0 0 0 0 0 0 0 0 0 0 0 0 0 0 0 0 0 0 0 0 0 0 0 0 0 -1 0 0 0 0 0 0 0 0 0 0 0 0 0 0 0 0 0 0 0 0 0
0 0 0 0 0 0 0 0 0 0 0 0 0 0 0 0 0 0 0 0 0 0 0 0 0 0 0 0 0 0 0 0 0 0 0;
Contrast "Fst Slope Diff" Log_Total_GSH*TwoGroups 1 -1 Log_Total_GSH*TwoGroups*Protein  0 0 0 0 0 0 0 0 0 0 0 0 0 0 0 0 0 0 0 0 0
0 0 0 0 0 0 0 0 0 0 0 0 0 0 0 1 0 0 0 0 0 0 0 0 0 0 0 0 0 0 0 0 0 0 0 0 0 0 0 0 0 0 0 0 0 0 0 0 0 0 0 0 0 0 0 0 0 0 0 0 0 0 0 0 0
0 0 0 0 0 0 0 0 0 0 0 0 0 0 0 0 0 0 0 0 0 0 0 0 0 0 0 0 0 0 0 0 0 0 0 0 0 0 0 0 0 0 -1 0 0 0 0 0 0 0 0 0 0 0 0 0 0 0 0 0 0 0 0 0
0 0 0 0 0 0 0 0 0 0 0 0 0 0 0 0 0 0 0 0 0 0 0 0 0 0 0 0 0 0 0 0 0 0;
Contrast "Fstl3 Slope Diff" Log_Total_GSH*TwoGroups 1 -1 Log_Total_GSH*TwoGroups*Protein  0 0 0 0 0 0 0 0 0 0 0 0 0 0 0 0 0 0 0 0
0 0 0 0 0 0 0 0 0 0 0 0 0 0 0 0 0 1 0 0 0 0 0 0 0 0 0 0 0 0 0 0 0 0 0 0 0 0 0 0 0 0 0 0 0 0 0 0 0 0 0 0 0 0 0 0 0 0 0 0 0 0 0 0 0
0 0 0 0 0 0 0 0 0 0 0 0 0 0 0 0 0 0 0 0 0 0 0 0 0 0 0 0 0 0 0 0 0 0 0 0 0 0 0 0 0 0 0 0 -1 0 0 0 0 0 0 0 0 0 0 0 0 0 0 0 0 0 0 0
0 0 0 0 0 0 0 0 0 0 0 0 0 0 0 0 0 0 0 0 0 0 0 0 0 0 0 0 0 0 0 0 0 0 0;
Contrast "Gcg Slope Diff" Log_Total_GSH*TwoGroups 1 -1 Log_Total_GSH*TwoGroups*Protein  0 0 0 0 0 0 0 0 0 0 0 0 0 0 0 0 0 0 0 0 0
0 0 0 0 0 0 0 0 0 0 0 0 0 0 0 0 0 1 0 0 0 0 0 0 0 0 0 0 0 0 0 0 0 0 0 0 0 0 0 0 0 0 0 0 0 0 0 0 0 0 0 0 0 0 0 0 0 0 0 0 0 0 0 0 0
0 0 0 0 0 0 0 0 0 0 0 0 0 0 0 0 0 0 0 0 0 0 0 0 0 0 0 0 0 0 0 0 0 0 0 0 0 0 0 0 0 0 0 0 -1 0 0 0 0 0 0 0 0 0 0 0 0 0 0 0 0 0 0 0
0 0 0 0 0 0 0 0 0 0 0 0 0 0 0 0 0 0 0 0 0 0 0 0 0 0 0 0 0 0 0 0 0 0;
Contrast "Gdnf Slope Diff" Log_Total_GSH*TwoGroups 1 -1 Log_Total_GSH*TwoGroups*Protein  0 0 0 0 0 0 0 0 0 0 0 0 0 0 0 0 0 0 0 0
0 0 0 0 0 0 0 0 0 0 0 0 0 0 0 0 0 0 0 1 0 0 0 0 0 0 0 0 0 0 0 0 0 0 0 0 0 0 0 0 0 0 0 0 0 0 0 0 0 0 0 0 0 0 0 0 0 0 0 0 0 0 0 0 0
0 0 0 0 0 0 0 0 0 0 0 0 0 0 0 0 0 0 0 0 0 0 0 0 0 0 0 0 0 0 0 0 0 0 0 0 0 0 0 0 0 0 0 0 0 0 -1 0 0 0 0 0 0 0 0 0 0 0 0 0 0 0 0 0
0 0 0 0 0 0 0 0 0 0 0 0 0 0 0 0 0 0 0 0 0 0 0 0 0 0 0 0 0 0 0 0 0 0 0;
Contrast "Gfra1 Slope Diff" Log_Total_GSH*TwoGroups 1 -1 Log_Total_GSH*TwoGroups*Protein  0 0 0 0 0 0 0 0 0 0 0 0 0 0 0 0 0 0 0 0
0 0 0 0 0 0 0 0 0 0 0 0 0 0 0 0 0 0 0 0 1 0 0 0 0 0 0 0 0 0 0 0 0 0 0 0 0 0 0 0 0 0 0 0 0 0 0 0 0 0 0 0 0 0 0 0 0 0 0 0 0 0 0 0 0
0 0 0 0 0 0 0 0 0 0 0 0 0 0 0 0 0 0 0 0 0 0 0 0 0 0 0 0 0 0 0 0 0 0 0 0 0 0 0 0 0 0 0 0 0 0 0 -1 0 0 0 0 0 0 0 0 0 0 0 0 0 0 0 0
0 0 0 0 0 0 0 0 0 0 0 0 0 0 0 0 0 0 0 0 0 0 0 0 0 0 0 0 0 0 0 0 0 0 0;
Contrast "Ghrl Slope Diff" Log_Total_GSH*TwoGroups 1 -1 Log_Total_GSH*TwoGroups*Protein  0 0 0 0 0 0 0 0 0 0 0 0 0 0 0 0 0 0 0 0
0 0 0 0 0 0 0 0 0 0 0 0 0 0 0 0 0 0 0 0 0 1 0 0 0 0 0 0 0 0 0 0 0 0 0 0 0 0 0 0 0 0 0 0 0 0 0 0 0 0 0 0 0 0 0 0 0 0 0 0 0 0 0 0 0
0 0 0 0 0 0 0 0 0 0 0 0 0 0 0 0 0 0 0 0 0 0 0 0 0 0 0 0 0 0 0 0 0 0 0 0 0 0 0 0 0 0 0 0 0 0 0 0 -1 0 0 0 0 0 0 0 0 0 0 0 0 0 0 0
0 0 0 0 0 0 0 0 0 0 0 0 0 0 0 0 0 0 0 0 0 0 0 0 0 0 0 0 0 0 0 0 0 0 0;
Contrast "Hgf Slope Diff" Log_Total_GSH*TwoGroups 1 -1 Log_Total_GSH*TwoGroups*Protein  0 0 0 0 0 0 0 0 0 0 0 0 0 0 0 0 0 0 0 0 0
0 0 0 0 0 0 0 0 0 0 0 0 0 0 0 0 0 0 0 0 0 1 0 0 0 0 0 0 0 0 0 0 0 0 0 0 0 0 0 0 0 0 0 0 0 0 0 0 0 0 0 0 0 0 0 0 0 0 0 0 0 0 0 0 0
0 0 0 0 0 0 0 0 0 0 0 0 0 0 0 0 0 0 0 0 0 0 0 0 0 0 0 0 0 0 0 0 0 0 0 0 0 0 0 0 0 0 0 0 0 0 0 0 -1 0 0 0 0 0 0 0 0 0 0 0 0 0 0 0
0 0 0 0 0 0 0 0 0 0 0 0 0 0 0 0 0 0 0 0 0 0 0 0 0 0 0 0 0 0 0 0 0 0;
Contrast "Igsf3 Slope Diff" Log_Total_GSH*TwoGroups 1 -1 Log_Total_GSH*TwoGroups*Protein  0 0 0 0 0 0 0 0 0 0 0 0 0 0 0 0 0 0 0 0
0 0 0 0 0 0 0 0 0 0 0 0 0 0 0 0 0 0 0 0 0 0 0 1 0 0 0 0 0 0 0 0 0 0 0 0 0 0 0 0 0 0 0 0 0 0 0 0 0 0 0 0 0 0 0 0 0 0 0 0 0 0 0 0 0
0 0 0 0 0 0 0 0 0 0 0 0 0 0 0 0 0 0 0 0 0 0 0 0 0 0 0 0 0 0 0 0 0 0 0 0 0 0 0 0 0 0 0 0 0 0 0 0 0 0 -1 0 0 0 0 0 0 0 0 0 0 0 0 0
0 0 0 0 0 0 0 0 0 0 0 0 0 0 0 0 0 0 0 0 0 0 0 0 0 0 0 0 0 0 0 0 0 0 0;
Contrast "Il10 Slope Diff" Log_Total_GSH*TwoGroups 1 -1 Log_Total_GSH*TwoGroups*Protein  0 0 0 0 0 0 0 0 0 0 0 0 0 0 0 0 0 0 0 0
0 0 0 0 0 0 0 0 0 0 0 0 0 0 0 0 0 0 0 0 0 0 0 0 1 0 0 0 0 0 0 0 0 0 0 0 0 0 0 0 0 0 0 0 0 0 0 0 0 0 0 0 0 0 0 0 0 0 0 0 0 0 0 0 0
0 0 0 0 0 0 0 0 0 0 0 0 0 0 0 0 0 0 0 0 0 0 0 0 0 0 0 0 0 0 0 0 0 0 0 0 0 0 0 0 0 0 0 0 0 0 0 0 0 0 0 -1 0 0 0 0 0 0 0 0 0 0 0 0
0 0 0 0 0 0 0 0 0 0 0 0 0 0 0 0 0 0 0 0 0 0 0 0 0 0 0 0 0 0 0 0 0 0 0;
Contrast "Il17a Slope Diff" Log_Total_GSH*TwoGroups 1 -1 Log_Total_GSH*TwoGroups*Protein  0 0 0 0 0 0 0 0 0 0 0 0 0 0 0 0 0 0 0 0
0 0 0 0 0 0 0 0 0 0 0 0 0 0 0 0 0 0 0 0 0 0 0 0 0 1 0 0 0 0 0 0 0 0 0 0 0 0 0 0 0 0 0 0 0 0 0 0 0 0 0 0 0 0 0 0 0 0 0 0 0 0 0 0 0
```

```
0 0 0 0 0 0 0 0 0 0 0 0 0 0 0 0 0 0 0 0 0 0 0 0 0 0 0 0 0 0 0 0 0 0 0 0 0 0 0 0 0 0 0 0 0 0 0 0 0 0 0 0 -1 0 0 0 0 0 0 0 0 0 0 0
0 0 0 0 0 0 0 0 0 0 0 0 0 0 0 0 0 0 0 0 0 0 0 0 0 0 0 0 0 0 0 0 0 0 0;
Contrast "Il17f Slope Diff" Log_Total_GSH*TwoGroups 1 -1 Log_Total_GSH*TwoGroups*Protein  0 0 0 0 0 0 0 0 0 0 0 0 0 0 0 0 0 0 0 0
0 0 0 0 0 0 0 0 0 0 0 0 0 0 0 0 0 0 0 0 0 0 0 0 0 0 1 0 0 0 0 0 0 0 0 0 0 0 0 0 0 0 0 0 0 0 0 0 0 0 0 0 0 0 0 0 0 0 0 0 0 0 0 0 0
0 0 0 0 0 0 0 0 0 0 0 0 0 0 0 0 0 0 0 0 0 0 0 0 0 0 0 0 0 0 0 0 0 0 0 0 0 0 0 0 0 0 0 0 0 0 0 0 0 0 0 0 0 -1 0 0 0 0 0 0 0 0 0 0
0 0 0 0 0 0 0 0 0 0 0 0 0 0 0 0 0 0 0 0 0 0 0 0 0 0 0 0 0 0 0 0 0 0 0;
Contrast "Il1a Slope Diff" Log_Total_GSH*TwoGroups 1 -1 Log_Total_GSH*TwoGroups*Protein  0 0 0 0 0 0 0 0 0 0 0 0 0 0 0 0 0 0 0 0
0 0 0 0 0 0 0 0 0 0 0 0 0 0 0 0 0 0 0 0 0 0 0 0 0 0 0 1 0 0 0 0 0 0 0 0 0 0 0 0 0 0 0 0 0 0 0 0 0 0 0 0 0 0 0 0 0 0 0 0 0 0 0 0 0
0 0 0 0 0 0 0 0 0 0 0 0 0 0 0 0 0 0 0 0 0 0 0 0 0 0 0 0 0 0 0 0 0 0 0 0 0 0 0 0 0 0 0 0 0 0 0 0 0 0 0 0 0 0 -1 0 0 0 0 0 0 0 0 0
0 0 0 0 0 0 0 0 0 0 0 0 0 0 0 0 0 0 0 0 0 0 0 0 0 0 0 0 0 0 0 0 0 0 0;
Contrast "Il1b Slope Diff" Log_Total_GSH*TwoGroups 1 -1 Log_Total_GSH*TwoGroups*Protein  0 0 0 0 0 0 0 0 0 0 0 0 0 0 0 0 0 0 0 0
0 0 0 0 0 0 0 0 0 0 0 0 0 0 0 0 0 0 0 0 0 0 0 0 0 0 0 0 1 0 0 0 0 0 0 0 0 0 0 0 0 0 0 0 0 0 0 0 0 0 0 0 0 0 0 0 0 0 0 0 0 0 0 0 0
0 0 0 0 0 0 0 0 0 0 0 0 0 0 0 0 0 0 0 0 0 0 0 0 0 0 0 0 0 0 0 0 0 0 0 0 0 0 0 0 0 0 0 0 0 0 0 0 0 0 0 0 0 0 0 -1 0 0 0 0 0 0 0 0
0 0 0 0 0 0 0 0 0 0 0 0 0 0 0 0 0 0 0 0 0 0 0 0 0 0 0 0 0 0 0 0 0 0 0;
Contrast "Il23r Slope Diff" Log_Total_GSH*TwoGroups 1 -1 Log_Total_GSH*TwoGroups*Protein  0 0 0 0 0 0 0 0 0 0 0 0 0 0 0 0 0 0 0 0
0 0 0 0 0 0 0 0 0 0 0 0 0 0 0 0 0 0 0 0 0 0 0 0 0 0 0 0 0 1 0 0 0 0 0 0 0 0 0 0 0 0 0 0 0 0 0 0 0 0 0 0 0 0 0 0 0 0 0 0 0 0 0 0 0
0 0 0 0 0 0 0 0 0 0 0 0 0 0 0 0 0 0 0 0 0 0 0 0 0 0 0 0 0 0 0 0 0 0 0 0 0 0 0 0 0 0 0 0 0 0 0 0 0 0 0 0 0 0 0 0 -1 0 0 0 0 0 0 0
0 0 0 0 0 0 0 0 0 0 0 0 0 0 0 0 0 0 0 0 0 0 0 0 0 0 0 0 0 0 0 0 0 0 0;
Contrast "Il5 Slope Diff" Log_Total_GSH*TwoGroups 1 -1 Log_Total_GSH*TwoGroups*Protein  0 0 0 0 0 0 0 0 0 0 0 0 0 0 0 0 0 0 0 0 0
0 0 0 0 0 0 0 0 0 0 0 0 0 0 0 0 0 0 0 0 0 0 0 0 0 0 0 0 0 1 0 0 0 0 0 0 0 0 0 0 0 0 0 0 0 0 0 0 0 0 0 0 0 0 0 0 0 0 0 0 0 0 0 0 0
0 0 0 0 0 0 0 0 0 0 0 0 0 0 0 0 0 0 0 0 0 0 0 0 0 0 0 0 0 0 0 0 0 0 0 0 0 0 0 0 0 0 0 0 0 0 0 0 0 0 0 0 0 0 0 0 -1 0 0 0 0 0 0 0
0 0 0 0 0 0 0 0 0 0 0 0 0 0 0 0 0 0 0 0 0 0 0 0 0 0 0 0 0 0 0 0 0 0;
Contrast "Il6 Slope Diff" Log_Total_GSH*TwoGroups 1 -1 Log_Total_GSH*TwoGroups*Protein  0 0 0 0 0 0 0 0 0 0 0 0 0 0 0 0 0 0 0 0 0
0 0 0 0 0 0 0 0 0 0 0 0 0 0 0 0 0 0 0 0 0 0 0 0 0 0 0 0 0 0 1 0 0 0 0 0 0 0 0 0 0 0 0 0 0 0 0 0 0 0 0 0 0 0 0 0 0 0 0 0 0 0 0 0 0
0 0 0 0 0 0 0 0 0 0 0 0 0 0 0 0 0 0 0 0 0 0 0 0 0 0 0 0 0 0 0 0 0 0 0 0 0 0 0 0 0 0 0 0 0 0 0 0 0 0 0 0 0 0 0 0 0 -1 0 0 0 0 0 0
0 0 0 0 0 0 0 0 0 0 0 0 0 0 0 0 0 0 0 0 0 0 0 0 0 0 0 0 0 0 0 0 0 0;
Contrast "Itgb1bp2 Slope Diff" Log_Total_GSH*TwoGroups 1 -1 Log_Total_GSH*TwoGroups*Protein  0 0 0 0 0 0 0 0 0 0 0 0 0 0 0 0 0 0
0 0 0 0 0 0 0 0 0 0 0 0 0 0 0 0 0 0 0 0 0 0 0 0 0 0 0 0 0 0 0 0 0 0 1 0 0 0 0 0 0 0 0 0 0 0 0 0 0 0 0 0 0 0 0 0 0 0 0 0 0 0 0 0 0
0 0 0 0 0 0 0 0 0 0 0 0 0 0 0 0 0 0 0 0 0 0 0 0 0 0 0 0 0 0 0 0 0 0 0 0 0 0 0 0 0 0 0 0 0 0 0 0 0 0 0 0 0 0 0 0 0 0 0 0 0 -1 0 0
0 0 0 0 0 0 0 0 0 0 0 0 0 0 0 0 0 0 0 0 0 0 0 0 0 0 0 0 0 0 0 0 0 0 0 0 0;
Contrast "Itgb6 Slope Diff" Log_Total_GSH*TwoGroups 1 -1 Log_Total_GSH*TwoGroups*Protein  0 0 0 0 0 0 0 0 0 0 0 0 0 0 0 0 0 0 0 0
0 0 0 0 0 0 0 0 0 0 0 0 0 0 0 0 0 0 0 0 0 0 0 0 0 0 0 0 0 0 0 0 0 1 0 0 0 0 0 0 0 0 0 0 0 0 0 0 0 0 0 0 0 0 0 0 0 0 0 0 0 0 0 0 0
0 0 0 0 0 0 0 0 0 0 0 0 0 0 0 0 0 0 0 0 0 0 0 0 0 0 0 0 0 0 0 0 0 0 0 0 0 0 0 0 0 0 0 0 0 0 0 0 0 0 0 0 0 0 0 0 0 0 0 0 -1 0 0 0
0 0 0 0 0 0 0 0 0 0 0 0 0 0 0 0 0 0 0 0 0 0 0 0 0 0 0 0 0 0 0 0 0 0 0;
Contrast "Kitlg Slope Diff" Log_Total_GSH*TwoGroups 1 -1 Log_Total_GSH*TwoGroups*Protein  0 0 0 0 0 0 0 0 0 0 0 0 0 0 0 0 0 0 0 0
0 0 0 0 0 0 0 0 0 0 0 0 0 0 0 0 0 0 0 0 0 0 0 0 0 0 0 0 0 0 0 0 0 0 1 0 0 0 0 0 0 0 0 0 0 0 0 0 0 0 0 0 0 0 0 0 0 0 0 0 0 0 0 0 0
0 0 0 0 0 0 0 0 0 0 0 0 0 0 0 0 0 0 0 0 0 0 0 0 0 0 0 0 0 0 0 0 0 0 0 0 0 0 0 0 0 0 0 0 0 0 0 0 0 0 0 0 0 0 0 0 0 0 0 0 0 -1 0 0
0 0 0 0 0 0 0 0 0 0 0 0 0 0 0 0 0 0 0 0 0 0 0 0 0 0 0 0 0 0 0 0 0 0 0;
Contrast "Lgmn Slope Diff" Log_Total_GSH*TwoGroups 1 -1 Log_Total_GSH*TwoGroups*Protein  0 0 0 0 0 0 0 0 0 0 0 0 0 0 0 0 0 0 0 0
0 0 0 0 0 0 0 0 0 0 0 0 0 0 0 0 0 0 0 0 0 0 0 0 0 0 0 0 0 0 0 0 0 0 0 1 0 0 0 0 0 0 0 0 0 0 0 0 0 0 0 0 0 0 0 0 0 0 0 0 0 0 0 0 0
0 0 0 0 0 0 0 0 0 0 0 0 0 0 0 0 0 0 0 0 0 0 0 0 0 0 0 0 0 0 0 0 0 0 0 0 0 0 0 0 0 0 0 0 0 0 0 0 0 0 0 0 0 0 0 0 0 0 0 0 0 0 -1 0
0 0 0 0 0 0 0 0 0 0 0 0 0 0 0 0 0 0 0 0 0 0 0 0 0 0 0 0 0 0 0 0 0 0 0;
Contrast "Lpl Slope Diff" Log_Total_GSH*TwoGroups 1 -1 Log_Total_GSH*TwoGroups*Protein  0 0 0 0 0 0 0 0 0 0 0 0 0 0 0 0 0 0 0 0 0
0 0 0 0 0 0 0 0 0 0 0 0 0 0 0 0 0 0 0 0 0 0 0 0 0 0 0 0 0 0 0 0 0 0 0 1 0 0 0 0 0 0 0 0 0 0 0 0 0 0 0 0 0 0 0 0 0 0 0 0 0 0 0 0 0
0 0 0 0 0 0 0 0 0 0 0 0 0 0 0 0 0 0 0 0 0 0 0 0 0 0 0 0 0 0 0 0 0 0 0 0 0 0 0 0 0 0 0 0 0 0 0 0 0 0 0 0 0 0 0 0 0 0 0 0 0 0 -1 0
0 0 0 0 0 0 0 0 0 0 0 0 0 0 0 0 0 0 0 0 0 0 0 0 0 0 0 0 0 0 0 0 0 0;
Contrast "Map2k6 Slope Diff" Log_Total_GSH*TwoGroups 1 -1 Log_Total_GSH*TwoGroups*Protein  0 0 0 0 0 0 0 0 0 0 0 0 0 0 0 0 0 0 0
0 0 0 0 0 0 0 0 0 0 0 0 0 0 0 0 0 0 0 0 0 0 0 0 0 0 0 0 0 0 0 0 0 0 0 0 0 0 1 0 0 0 0 0 0 0 0 0 0 0 0 0 0 0 0 0 0 0 0 0 0 0 0 0 0
0 0 0 0 0 0 0 0 0 0 0 0 0 0 0 0 0 0 0 0 0 0 0 0 0 0 0 0 0 0 0 0 0 0 0 0 0 0 0 0 0 0 0 0 0 0 0 0 0 0 0 0 0 0 0 0 0 0 0 0 0 0 0 0 0
-1 0 0 0 0 0 0 0 0 0 0 0 0 0 0 0 0 0 0 0 0 0 0 0 0 0 0 0 0 0 0 0 0 0 0;
Contrast "Matn2 Slope Diff" Log_Total_GSH*TwoGroups 1 -1 Log_Total_GSH*TwoGroups*Protein  0 0 0 0 0 0 0 0 0 0 0 0 0 0 0 0 0 0 0 0
0 0 0 0 0 0 0 0 0 0 0 0 0 0 0 0 0 0 0 0 0 0 0 0 0 0 0 0 0 0 0 0 0 0 0 0 0 0 1 0 0 0 0 0 0 0 0 0 0 0 0 0 0 0 0 0 0 0 0 0 0 0 0 0 0
0 0 0 0 0 0 0 0 0 0 0 0 0 0 0 0 0 0 0 0 0 0 0 0 0 0 0 0 0 0 0 0 0 0 0 0 0 0 0 0 0 0 0 0 0 0 0 0 0 0 0 0 0 0 0 0 0 0 0 0 0 0 0 0 0
-1 0 0 0 0 0 0 0 0 0 0 0 0 0 0 0 0 0 0 0 0 0 0 0 0 0 0 0 0 0 0 0 0 0;
Contrast "Mia Slope Diff" Log_Total_GSH*TwoGroups 1 -1 Log_Total_GSH*TwoGroups*Protein  0 0 0 0 0 0 0 0 0 0 0 0 0 0 0 0 0 0 0 0 0
0 0 0 0 0 0 0 0 0 0 0 0 0 0 0 0 0 0 0 0 0 0 0 0 0 0 0 0 0 0 0 0 0 0 0 0 0 0 1 0 0 0 0 0 0 0 0 0 0 0 0 0 0 0 0 0 0 0 0 0 0 0 0 0 0
0 0 0 0 0 0 0 0 0 0 0 0 0 0 0 0 0 0 0 0 0 0 0 0 0 0 0 0 0 0 0 0 0 0 0 0 0 0 0 0 0 0 0 0 0 0 0 0 0 0 0 0 0 0 0 0 0 0 0 0 0 0 0 0 0
-1 0 0 0 0 0 0 0 0 0 0 0 0 0 0 0 0 0 0 0 0 0 0 0 0 0 0 0 0 0 0 0 0;
Contrast "Nadk Slope Diff" Log_Total_GSH*TwoGroups 1 -1 Log_Total_GSH*TwoGroups*Protein  0 0 0 0 0 0 0 0 0 0 0 0 0 0 0 0 0 0 0 0
0 0 0 0 0 0 0 0 0 0 0 0 0 0 0 0 0 0 0 0 0 0 0 0 0 0 0 0 0 0 0 0 0 0 0 0 0 0 0 0 1 0 0 0 0 0 0 0 0 0 0 0 0 0 0 0 0 0 0 0 0 0 0 0 0
0 0 0 0 0 0 0 0 0 0 0 0 0 0 0 0 0 0 0 0 0 0 0 0 0 0 0 0 0 0 0 0 0 0 0 0 0 0 0 0 0 0 0 0 0 0 0 0 0 0 0 0 0 0 0 0 0 0 0 0 0 0 0 0 0
0 0 -1 0 0 0 0 0 0 0 0 0 0 0 0 0 0 0 0 0 0 0 0 0 0 0 0 0 0 0 0 0 0 0;
Contrast "Notch3 Slope Diff" Log_Total_GSH*TwoGroups 1 -1 Log_Total_GSH*TwoGroups*Protein  0 0 0 0 0 0 0 0 0 0 0 0 0 0 0 0 0 0 0
0 0 0 0 0 0 0 0 0 0 0 0 0 0 0 0 0 0 0 0 0 0 0 0 0 0 0 0 0 0 0 0 0 0 0 0 0 0 0 0 0 0 1 0 0 0 0 0 0 0 0 0 0 0 0 0 0 0 0 0 0 0 0 0 0
0 0 0 0 0 0 0 0 0 0 0 0 0 0 0 0 0 0 0 0 0 0 0 0 0 0 0 0 0 0 0 0 0 0 0 0 0 0 0 0 0 0 0 0 0 0 0 0 0 0 0 0 0 0 0 0 0 0 0 0 0 0 0 0 0
0 0 0 0 -1 0 0 0 0 0 0 0 0 0 0 0 0 0 0 0 0 0 0 0 0 0 0 0 0 0 0 0 0 0 0;
Contrast "Ntf3 Slope Diff" Log_Total_GSH*TwoGroups 1 -1 Log_Total_GSH*TwoGroups*Protein  0 0 0 0 0 0 0 0 0 0 0 0 0 0 0 0 0 0 0 0
0 0 0 0 0 0 0 0 0 0 0 0 0 0 0 0 0 0 0 0 0 0 0 0 0 0 0 0 0 0 0 0 0 0 0 0 0 0 0 0 0 0 1 0 0 0 0 0 0 0 0 0 0 0 0 0 0 0 0 0 0 0 0 0 0
0 0 0 0 0 0 0 0 0 0 0 0 0 0 0 0 0 0 0 0 0 0 0 0 0 0 0 0 0 0 0 0 0 0 0 0 0 0 0 0 0 0 0 0 0 0 0 0 0 0 0 0 0 0 0 0 0 0 0 0 0 0 0 0 0
0 0 0 0 -1 0 0 0 0 0 0 0 0 0 0 0 0 0 0 0 0 0 0 0 0 0 0 0 0 0 0 0 0 0;
Contrast "Pak4 Slope Diff" Log_Total_GSH*TwoGroups 1 -1 Log_Total_GSH*TwoGroups*Protein  0 0 0 0 0 0 0 0 0 0 0 0 0 0 0 0 0 0 0 0
0 0 0 0 0 0 0 0 0 0 0 0 0 0 0 0 0 0 0 0 0 0 0 0 0 0 0 0 0 0 0 0 0 0 0 0 0 0 0 0 0 0 0 1 0 0 0 0 0 0 0 0 0 0 0 0 0 0 0 0 0 0 0 0 0
0 0 0 0 0 0 0 0 0 0 0 0 0 0 0 0 0 0 0 0 0 0 0 0 0 0 0 0 0 0 0 0 0 0 0 0 0 0 0 0 0 0 0 0 0 0 0 0 0 0 0 0 0 0 0 0 0 0 0 0 0 0 0 0 0
0 0 0 0 0 -1 0 0 0 0 0 0 0 0 0 0 0 0 0 0 0 0 0 0 0 0 0 0 0 0 0 0 0 0;
Contrast "Parp1 Slope Diff" Log_Total_GSH*TwoGroups 1 -1 Log_Total_GSH*TwoGroups*Protein  0 0 0 0 0 0 0 0 0 0 0 0 0 0 0 0 0 0 0 0
0 0 0 0 0 0 0 0 0 0 0 0 0 0 0 0 0 0 0 0 0 0 0 0 0 0 0 0 0 0 0 0 0 0 0 0 0 0 0 0 0 0 0 0 1 0 0 0 0 0 0 0 0 0 0 0 0 0 0 0 0 0 0 0 0
0 0 0 0 0 0 0 0 0 0 0 0 0 0 0 0 0 0 0 0 0 0 0 0 0 0 0 0 0 0 0 0 0 0 0 0 0 0 0 0 0 0 0 0 0 0 0 0 0 0 0 0 0 0 0 0 0 0 0 0 0 0 0 0 0
0 0 0 0 0 0 -1 0 0 0 0 0 0 0 0 0 0 0 0 0 0 0 0 0 0 0 0 0 0 0 0 0 0 0;
Contrast "Pdgfb Slope Diff" Log_Total_GSH*TwoGroups 1 -1 Log_Total_GSH*TwoGroups*Protein  0 0 0 0 0 0 0 0 0 0 0 0 0 0 0 0 0 0 0 0
0 0 0 0 0 0 0 0 0 0 0 0 0 0 0 0 0 0 0 0 0 0 0 0 0 0 0 0 0 0 0 0 0 0 0 0 0 0 0 0 0 0 0 0 0 1 0 0 0 0 0 0 0 0 0 0 0 0 0 0 0 0 0 0 0
0 0 0 0 0 0 0 0 0 0 0 0 0 0 0 0 0 0 0 0 0 0 0 0 0 0 0 0 0 0 0 0 0 0 0 0 0 0 0 0 0 0 0 0 0 0 0 0 0 0 0 0 0 0 0 0 0 0 0 0 0 0 0 0 0
0 0 0 0 0 0 0 -1 0 0 0 0 0 0 0 0 0 0 0 0 0 0 0 0 0 0 0 0 0 0 0 0 0 0;
Contrast "Pla2g4a Slope Diff" Log_Total_GSH*TwoGroups 1 -1 Log_Total_GSH*TwoGroups*Protein  0 0 0 0 0 0 0 0 0 0 0 0 0 0 0 0 0 0 0
0 0 0 0 0 0 0 0 0 0 0 0 0 0 0 0 0 0 0 0 0 0 0 0 0 0 0 0 0 0 0 0 0 0 0 0 0 0 0 0 0 0 0 0 0 0 0 1 0 0 0 0 0 0 0 0 0 0 0 0 0 0 0 0 0
0 0 0 0 0 0 0 0 0 0 0 0 0 0 0 0 0 0 0 0 0 0 0 0 0 0 0 0 0 0 0 0 0 0 0 0 0 0 0 0 0 0 0 0 0 0 0 0 0 0 0 0 0 0 0 0 0 0 0 0 0 0 0 0 0
0 0 0 0 0 0 0 0 0 -1 0 0 0 0 0 0 0 0 0 0 0 0 0 0 0 0 0 0 0 0 0 0 0 0 0;
Contrast "Plin1 Slope Diff" Log_Total_GSH*TwoGroups 1 -1 Log_Total_GSH*TwoGroups*Protein  0 0 0 0 0 0 0 0 0 0 0 0 0 0 0 0 0 0 0 0
0 0 0 0 0 0 0 0 0 0 0 0 0 0 0 0 0 0 0 0 0 0 0 0 0 0 0 0 0 0 0 0 0 0 0 0 0 0 0 0 0 0 0 0 0 0 0 1 0 0 0 0 0 0 0 0 0 0 0 0 0 0 0 0 0
0 0 0 0 0 0 0 0 0 0 0 0 0 0 0 0 0 0 0 0 0 0 0 0 0 0 0 0 0 0 0 0 0 0 0 0 0 0 0 0 0 0 0 0 0 0 0 0 0 0 0 0 0 0 0 0 0 0 0 0 0 0 0 0 0
0 0 0 0 0 0 0 0 0 -1 0 0 0 0 0 0 0 0 0 0 0 0 0 0 0 0 0 0 0 0 0 0 0 0;
Contrast "Plxna4 Slope Diff" Log_Total_GSH*TwoGroups 1 -1 Log_Total_GSH*TwoGroups*Protein  0 0 0 0 0 0 0 0 0 0 0 0 0 0 0 0 0 0 0
0 0 0 0 0 0 0 0 0 0 0 0 0 0 0 0 0 0 0 0 0 0 0 0 0 0 0 0 0 0 0 0 0 0 0 0 0 0 0 0 0 0 0 0 0 0 0 0 0 1 0 0 0 0 0 0 0 0 0 0 0 0 0 0 0
0 0 0 0 0 0 0 0 0 0 0 0 0 0 0 0 0 0 0 0 0 0 0 0 0 0 0 0 0 0 0 0 0 0 0 0 0 0 0 0 0 0 0 0 0 0 0 0 0 0 0 0 0 0 0 0 0 0 0 0 0 0 0 0 0
0 0 0 0 0 0 0 0 0 0 0 -1 0 0 0 0 0 0 0 0 0 0 0 0 0 0 0 0 0 0 0 0 0 0 0;
```

```
Contrast "Ppp1r2 Slope Diff" Log_Total_GSH*TwoGroups 1 -1 Log_Total_GSH*TwoGroups*Protein  0 0 0 0 0 0 0 0 0 0 0 0 0 0 0 0 0 0 0
0 0 0 0 0 0 0 0 0 0 0 0 0 0 0 0 0 0 0 0 0 0 0 0 0 0 0 0 0 0 0 0 0 0 0 0 0 0 0 0 0 0 0 0 0 0 0 0 0 0 1 0 0 0 0 0 0 0 0 0 0 0 0 0 0
0 0 0 0 0 0 0 0 0 0 0 0 0 0 0 0 0 0 0 0 0 0 0 0 0 0 0 0 0 0 0 0 0 0 0 0 0 0 0 0 0 0 0 0 0 0 0 0 0 0 0 0 0 0 0 0 0 0 0 0 0 0 0 0 0
0 0 0 0 0 0 0 0 0 0 0 0 -1 0 0 0 0 0 0 0 0 0 0 0 0 0 0 0 0 0 0 0 0 0 0;
Contrast "Prdx5 Slope Diff" Log_Total_GSH*TwoGroups 1 -1 Log_Total_GSH*TwoGroups*Protein  0 0 0 0 0 0 0 0 0 0 0 0 0 0 0 0 0 0 0 0
0 0 0 0 0 0 0 0 0 0 0 0 0 0 0 0 0 0 0 0 0 0 0 0 0 0 0 0 0 0 0 0 0 0 0 0 0 0 0 0 0 0 0 0 0 0 0 0 0 0 1 0 0 0 0 0 0 0 0 0 0 0 0 0 0
0 0 0 0 0 0 0 0 0 0 0 0 0 0 0 0 0 0 0 0 0 0 0 0 0 0 0 0 0 0 0 0 0 0 0 0 0 0 0 0 0 0 0 0 0 0 0 0 0 0 0 0 0 0 0 0 0 0 0 0 0 0 0 0 0
0 0 0 0 0 0 0 0 0 0 0 0 -1 0 0 0 0 0 0 0 0 0 0 0 0 0 0 0 0 0 0 0 0 0;
Contrast "Qdpr Slope Diff" Log_Total_GSH*TwoGroups 1 -1 Log_Total_GSH*TwoGroups*Protein  0 0 0 0 0 0 0 0 0 0 0 0 0 0 0 0 0 0 0 0
0 0 0 0 0 0 0 0 0 0 0 0 0 0 0 0 0 0 0 0 0 0 0 0 0 0 0 0 0 0 0 0 0 0 0 0 0 0 0 0 0 0 0 0 0 0 0 0 0 0 0 1 0 0 0 0 0 0 0 0 0 0 0 0 0
0 0 0 0 0 0 0 0 0 0 0 0 0 0 0 0 0 0 0 0 0 0 0 0 0 0 0 0 0 0 0 0 0 0 0 0 0 0 0 0 0 0 0 0 0 0 0 0 0 0 0 0 0 0 0 0 0 0 0 0 0 0 0 0 0
0 0 0 0 0 0 0 0 0 0 0 0 0 -1 0 0 0 0 0 0 0 0 0 0 0 0 0 0 0 0 0 0 0 0;
Contrast "Rgma Slope Diff" Log_Total_GSH*TwoGroups 1 -1 Log_Total_GSH*TwoGroups*Protein  0 0 0 0 0 0 0 0 0 0 0 0 0 0 0 0 0 0 0 0
0 0 0 0 0 0 0 0 0 0 0 0 0 0 0 0 0 0 0 0 0 0 0 0 0 0 0 0 0 0 0 0 0 0 0 0 0 0 0 0 0 0 0 0 0 0 0 0 0 0 0 0 1 0 0 0 0 0 0 0 0 0 0 0 0
0 0 0 0 0 0 0 0 0 0 0 0 0 0 0 0 0 0 0 0 0 0 0 0 0 0 0 0 0 0 0 0 0 0 0 0 0 0 0 0 0 0 0 0 0 0 0 0 0 0 0 0 0 0 0 0 0 0 0 0 0 0 0 0 0
0 0 0 0 0 0 0 0 0 0 0 0 0 0 -1 0 0 0 0 0 0 0 0 0 0 0 0 0 0 0 0 0 0 0;
Contrast "Riox2 Slope Diff" Log_Total_GSH*TwoGroups 1 -1 Log_Total_GSH*TwoGroups*Protein  0 0 0 0 0 0 0 0 0 0 0 0 0 0 0 0 0 0 0 0
0 0 0 0 0 0 0 0 0 0 0 0 0 0 0 0 0 0 0 0 0 0 0 0 0 0 0 0 0 0 0 0 0 0 0 0 0 0 0 0 0 0 0 0 0 0 0 0 0 0 0 0 0 1 0 0 0 0 0 0 0 0 0 0 0
0 0 0 0 0 0 0 0 0 0 0 0 0 0 0 0 0 0 0 0 0 0 0 0 0 0 0 0 0 0 0 0 0 0 0 0 0 0 0 0 0 0 0 0 0 0 0 0 0 0 0 0 0 0 0 0 0 0 0 0 0 0 0 0 0
0 0 0 0 0 0 0 0 0 0 0 0 0 0 0 -1 0 0 0 0 0 0 0 0 0 0 0 0 0 0 0 0 0 0;
Contrast "S100a4 Slope Diff" Log_Total_GSH*TwoGroups 1 -1 Log_Total_GSH*TwoGroups*Protein  0 0 0 0 0 0 0 0 0 0 0 0 0 0 0 0 0 0 0
0 0 0 0 0 0 0 0 0 0 0 0 0 0 0 0 0 0 0 0 0 0 0 0 0 0 0 0 0 0 0 0 0 0 0 0 0 0 0 0 0 0 0 0 0 0 0 0 0 0 0 0 0 0 0 1 0 0 0 0 0 0 0 0 0
0 0 0 0 0 0 0 0 0 0 0 0 0 0 0 0 0 0 0 0 0 0 0 0 0 0 0 0 0 0 0 0 0 0 0 0 0 0 0 0 0 0 0 0 0 0 0 0 0 0 0 0 0 0 0 0 0 0 0 0 0 0 0 0 0
0 0 0 0 0 0 0 0 0 0 0 0 0 0 0 0 0 -1 0 0 0 0 0 0 0 0 0 0 0 0 0 0 0 0 0;
Contrast "Sez6l2 Slope Diff" Log_Total_GSH*TwoGroups 1 -1 Log_Total_GSH*TwoGroups*Protein  0 0 0 0 0 0 0 0 0 0 0 0 0 0 0 0 0 0 0
0 0 0 0 0 0 0 0 0 0 0 0 0 0 0 0 0 0 0 0 0 0 0 0 0 0 0 0 0 0 0 0 0 0 0 0 0 0 0 0 0 0 0 0 0 0 0 0 0 0 0 0 0 0 0 0 1 0 0 0 0 0 0 0 0
0 0 0 0 0 0 0 0 0 0 0 0 0 0 0 0 0 0 0 0 0 0 0 0 0 0 0 0 0 0 0 0 0 0 0 0 0 0 0 0 0 0 0 0 0 0 0 0 0 0 0 0 0 0 0 0 0 0 0 0 0 0 0 0 0
0 0 0 0 0 0 0 0 0 0 0 0 0 0 0 0 0 0 -1 0 0 0 0 0 0 0 0 0 0 0 0 0 0 0 0;
Contrast "Snap29 Slope Diff" Log_Total_GSH*TwoGroups 1 -1 Log_Total_GSH*TwoGroups*Protein  0 0 0 0 0 0 0 0 0 0 0 0 0 0 0 0 0 0 0
0 0 0 0 0 0 0 0 0 0 0 0 0 0 0 0 0 0 0 0 0 0 0 0 0 0 0 0 0 0 0 0 0 0 0 0 0 0 0 0 0 0 0 0 0 0 0 0 0 0 0 0 0 0 0 0 0 1 0 0 0 0 0 0 0
0 0 0 0 0 0 0 0 0 0 0 0 0 0 0 0 0 0 0 0 0 0 0 0 0 0 0 0 0 0 0 0 0 0 0 0 0 0 0 0 0 0 0 0 0 0 0 0 0 0 0 0 0 0 0 0 0 0 0 0 0 0 0 0 0
0 0 0 0 0 0 0 0 0 0 0 0 0 0 0 0 0 0 0 -1 0 0 0 0 0 0 0 0 0 0 0 0 0 0 0;
Contrast "Tgfa Slope Diff" Log_Total_GSH*TwoGroups 1 -1 Log_Total_GSH*TwoGroups*Protein  0 0 0 0 0 0 0 0 0 0 0 0 0 0 0 0 0 0 0 0
0 0 0 0 0 0 0 0 0 0 0 0 0 0 0 0 0 0 0 0 0 0 0 0 0 0 0 0 0 0 0 0 0 0 0 0 0 0 0 0 0 0 0 0 0 0 0 0 0 0 0 0 0 0 0 0 0 1 0 0 0 0 0 0 0
0 0 0 0 0 0 0 0 0 0 0 0 0 0 0 0 0 0 0 0 0 0 0 0 0 0 0 0 0 0 0 0 0 0 0 0 0 0 0 0 0 0 0 0 0 0 0 0 0 0 0 0 0 0 0 0 0 0 0 0 0 0 0 0 0
0 0 0 0 0 0 0 0 0 0 0 0 0 0 0 0 0 0 0 -1 0 0 0 0 0 0 0 0 0 0 0 0 0 0;
Contrast "Tgfb1 Slope Diff" Log_Total_GSH*TwoGroups 1 -1 Log_Total_GSH*TwoGroups*Protein  0 0 0 0 0 0 0 0 0 0 0 0 0 0 0 0 0 0 0 0
0 0 0 0 0 0 0 0 0 0 0 0 0 0 0 0 0 0 0 0 0 0 0 0 0 0 0 0 0 0 0 0 0 0 0 0 0 0 0 0 0 0 0 0 0 0 0 0 0 0 0 0 0 0 0 0 0 0 1 0 0 0 0 0 0
0 0 0 0 0 0 0 0 0 0 0 0 0 0 0 0 0 0 0 0 0 0 0 0 0 0 0 0 0 0 0 0 0 0 0 0 0 0 0 0 0 0 0 0 0 0 0 0 0 0 0 0 0 0 0 0 0 0 0 0 0 0 0 0 0
0 0 0 0 0 0 0 0 0 0 0 0 0 0 0 0 0 0 0 0 -1 0 0 0 0 0 0 0 0 0 0 0 0 0;
Contrast "Tgfbr3 Slope Diff" Log_Total_GSH*TwoGroups 1 -1 Log_Total_GSH*TwoGroups*Protein  0 0 0 0 0 0 0 0 0 0 0 0 0 0 0 0 0 0 0
0 0 0 0 0 0 0 0 0 0 0 0 0 0 0 0 0 0 0 0 0 0 0 0 0 0 0 0 0 0 0 0 0 0 0 0 0 0 0 0 0 0 0 0 0 0 0 0 0 0 0 0 0 0 0 0 0 0 0 0 1 0 0 0 0
0 0 0 0 0 0 0 0 0 0 0 0 0 0 0 0 0 0 0 0 0 0 0 0 0 0 0 0 0 0 0 0 0 0 0 0 0 0 0 0 0 0 0 0 0 0 0 0 0 0 0 0 0 0 0 0 0 0 0 0 0 0 0 0 0
0 0 0 0 0 0 0 0 0 0 0 0 0 0 0 0 0 0 0 0 0 0 -1 0 0 0 0 0 0 0 0 0 0 0 0;
Contrast "Tnf Slope Diff" Log_Total_GSH*TwoGroups 1 -1 Log_Total_GSH*TwoGroups*Protein  0 0 0 0 0 0 0 0 0 0 0 0 0 0 0 0 0 0 0 0 0
0 0 0 0 0 0 0 0 0 0 0 0 0 0 0 0 0 0 0 0 0 0 0 0 0 0 0 0 0 0 0 0 0 0 0 0 0 0 0 0 0 0 0 0 0 0 0 0 0 0 0 0 0 0 0 0 0 0 0 1 0 0 0 0 0
0 0 0 0 0 0 0 0 0 0 0 0 0 0 0 0 0 0 0 0 0 0 0 0 0 0 0 0 0 0 0 0 0 0 0 0 0 0 0 0 0 0 0 0 0 0 0 0 0 0 0 0 0 0 0 0 0 0 0 0 0 0 0 0 0
0 0 0 0 0 0 0 0 0 0 0 0 0 0 0 0 0 0 0 0 0 -1 0 0 0 0 0 0 0 0 0 0 0;
Contrast "Tnfrsf11b Slope Diff" Log_Total_GSH*TwoGroups 1 -1 Log_Total_GSH*TwoGroups*Protein  0 0 0 0 0 0 0 0 0 0 0 0 0 0 0 0 0 0
0 0 0 0 0 0 0 0 0 0 0 0 0 0 0 0 0 0 0 0 0 0 0 0 0 0 0 0 0 0 0 0 0 0 0 0 0 0 0 0 0 0 0 0 0 0 0 0 0 0 0 0 0 0 0 0 0 0 0 0 0 0 0 1 0
0 0 0 0 0 0 0 0 0 0 0 0 0 0 0 0 0 0 0 0 0 0 0 0 0 0 0 0 0 0 0 0 0 0 0 0 0 0 0 0 0 0 0 0 0 0 0 0 0 0 0 0 0 0 0 0 0 0 0 0 0 0 0 0 0
0 0 0 0 0 0 0 0 0 0 0 0 0 0 0 0 0 0 0 0 0 0 0 0 0 -1 0 0 0 0 0 0 0 0 0 0;
Contrast "Tnfrsf12a Slope Diff" Log_Total_GSH*TwoGroups 1 -1 Log_Total_GSH*TwoGroups*Protein  0 0 0 0 0 0 0 0 0 0 0 0 0 0 0 0 0 0
0 0 0 0 0 0 0 0 0 0 0 0 0 0 0 0 0 0 0 0 0 0 0 0 0 0 0 0 0 0 0 0 0 0 0 0 0 0 0 0 0 0 0 0 0 0 0 0 0 0 0 0 0 0 0 0 0 0 0 0 0 0 0 0 1
0 0 0 0 0 0 0 0 0 0 0 0 0 0 0 0 0 0 0 0 0 0 0 0 0 0 0 0 0 0 0 0 0 0 0 0 0 0 0 0 0 0 0 0 0 0 0 0 0 0 0 0 0 0 0 0 0 0 0 0 0 0 0 0 0
0 0 0 0 0 0 0 0 0 0 0 0 0 0 0 0 0 0 0 0 0 0 0 0 0 0 -1 0 0 0 0 0 0 0 0 0;
Contrast "Tnfsf12 Slope Diff" Log_Total_GSH*TwoGroups 1 -1 Log_Total_GSH*TwoGroups*Protein  0 0 0 0 0 0 0 0 0 0 0 0 0 0 0 0 0 0 0
0 0 0 0 0 0 0 0 0 0 0 0 0 0 0 0 0 0 0 0 0 0 0 0 0 0 0 0 0 0 0 0 0 0 0 0 0 0 0 0 0 0 0 0 0 0 0 0 0 0 0 0 0 0 0 0 0 0 0 0 0 0 0 0 1
0 0 0 0 0 0 0 0 0 0 0 0 0 0 0 0 0 0 0 0 0 0 0 0 0 0 0 0 0 0 0 0 0 0 0 0 0 0 0 0 0 0 0 0 0 0 0 0 0 0 0 0 0 0 0 0 0 0 0 0 0 0 0 0 0
0 0 0 0 0 0 0 0 0 0 0 0 0 0 0 0 0 0 0 0 0 0 0 0 0 0 -1 0 0 0 0 0 0 0 0;
Contrast "Tnni3 Slope Diff" Log_Total_GSH*TwoGroups 1 -1 Log_Total_GSH*TwoGroups*Protein  0 0 0 0 0 0 0 0 0 0 0 0 0 0 0 0 0 0 0 0
0 0 0 0 0 0 0 0 0 0 0 0 0 0 0 0 0 0 0 0 0 0 0 0 0 0 0 0 0 0 0 0 0 0 0 0 0 0 0 0 0 0 0 0 0 0 0 0 0 0 0 0 0 0 0 0 0 0 0 0 0 0 0 0 1
0 0 0 0 0 0 0 0 0 0 0 0 0 0 0 0 0 0 0 0 0 0 0 0 0 0 0 0 0 0 0 0 0 0 0 0 0 0 0 0 0 0 0 0 0 0 0 0 0 0 0 0 0 0 0 0 0 0 0 0 0 0 0 0 0
0 0 0 0 0 0 0 0 0 0 0 0 0 0 0 0 0 0 0 0 0 0 0 0 0 0 -1 0 0 0 0 0 0 0;
Contrast "Tnr Slope Diff" Log_Total_GSH*TwoGroups 1 -1 Log_Total_GSH*TwoGroups*Protein  0 0 0 0 0 0 0 0 0 0 0 0 0 0 0 0 0 0 0 0 0
0 0 0 0 0 0 0 0 0 0 0 0 0 0 0 0 0 0 0 0 0 0 0 0 0 0 0 0 0 0 0 0 0 0 0 0 0 0 0 0 0 0 0 0 0 0 0 0 0 0 0 0 0 0 0 0 0 0 0 0 0 0 0 0 1
0 0 0 0 0 0 0 0 0 0 0 0 0 0 0 0 0 0 0 0 0 0 0 0 0 0 0 0 0 0 0 0 0 0 0 0 0 0 0 0 0 0 0 0 0 0 0 0 0 0 0 0 0 0 0 0 0 0 0 0 0 0 0 0 0
0 0 0 0 0 0 0 0 0 0 0 0 0 0 0 0 0 0 0 0 0 0 0 0 0 0 -1 0 0 0 0 0 0;
Contrast "Tpp1 Slope Diff" Log_Total_GSH*TwoGroups 1 -1 Log_Total_GSH*TwoGroups*Protein  0 0 0 0 0 0 0 0 0 0 0 0 0 0 0 0 0 0 0 0
0 0 0 0 0 0 0 0 0 0 0 0 0 0 0 0 0 0 0 0 0 0 0 0 0 0 0 0 0 0 0 0 0 0 0 0 0 0 0 0 0 0 0 0 0 0 0 0 0 0 0 0 0 0 0 0 0 0 0 0 0 0 0 0 0
0 1 0 0 0 0 0 0 0 0 0 0 0 0 0 0 0 0 0 0 0 0 0 0 0 0 0 0 0 0 0 0 0 0 0 0 0 0 0 0 0 0 0 0 0 0 0 0 0 0 0 0 0 0 0 0 0 0 0 0 0 0 0 0 0
0 0 0 0 0 0 0 0 0 0 0 0 0 0 0 0 0 0 0 0 0 0 0 0 0 0 0 0 -1 0 0 0 0 0;
Contrast "Vegfd Slope Diff" Log_Total_GSH*TwoGroups 1 -1 Log_Total_GSH*TwoGroups*Protein  0 0 0 0 0 0 0 0 0 0 0 0 0 0 0 0 0 0 0 0
0 0 0 0 0 0 0 0 0 0 0 0 0 0 0 0 0 0 0 0 0 0 0 0 0 0 0 0 0 0 0 0 0 0 0 0 0 0 0 0 0 0 0 0 0 0 0 0 0 0 0 0 0 0 0 0 0 0 0 0 0 0 0 0 0
0 0 1 0 0 0 0 0 0 0 0 0 0 0 0 0 0 0 0 0 0 0 0 0 0 0 0 0 0 0 0 0 0 0 0 0 0 0 0 0 0 0 0 0 0 0 0 0 0 0 0 0 0 0 0 0 0 0 0 0 0 0 0 0 0
0 0 0 0 0 0 0 0 0 0 0 0 0 0 0 0 0 0 0 0 0 0 0 0 0 0 0 0 0 -1 0 0 0 0;
Contrast "Vsig2 Slope Diff" Log_Total_GSH*TwoGroups 1 -1 Log_Total_GSH*TwoGroups*Protein  0 0 0 0 0 0 0 0 0 0 0 0 0 0 0 0 0 0 0 0
0 0 0 0 0 0 0 0 0 0 0 0 0 0 0 0 0 0 0 0 0 0 0 0 0 0 0 0 0 0 0 0 0 0 0 0 0 0 0 0 0 0 0 0 0 0 0 0 0 0 0 0 0 0 0 0 0 0 0 0 0 0 0 0 0
0 0 0 1 0 0 0 0 0 0 0 0 0 0 0 0 0 0 0 0 0 0 0 0 0 0 0 0 0 0 0 0 0 0 0 0 0 0 0 0 0 0 0 0 0 0 0 0 0 0 0 0 0 0 0 0 0 0 0 0 0 0 0 0 0
0 0 0 0 0 0 0 0 0 0 0 0 0 0 0 0 0 0 0 0 0 0 0 0 0 0 0 0 0 0 -1 0 0 0;
Contrast "Wfikkn2 Slope Diff" Log_Total_GSH*TwoGroups 1 -1 Log_Total_GSH*TwoGroups*Protein  0 0 0 0 0 0 0 0 0 0 0 0 0 0 0 0 0 0 0
0 0 0 0 0 0 0 0 0 0 0 0 0 0 0 0 0 0 0 0 0 0 0 0 0 0 0 0 0 0 0 0 0 0 0 0 0 0 0 0 0 0 0 0 0 0 0 0 0 0 0 0 0 0 0 0 0 0 0 0 0 0 0 0 0
0 0 0 0 0 1 0 0 0 0 0 0 0 0 0 0 0 0 0 0 0 0 0 0 0 0 0 0 0 0 0 0 0 0 0 0 0 0 0 0 0 0 0 0 0 0 0 0 0 0 0 0 0 0 0 0 0 0 0 0 0 0 0 0 0
0 0 0 0 0 0 0 0 0 0 0 0 0 0 0 0 0 0 0 0 0 0 0 0 0 0 0 0 0 0 0 0 -1 0 0;
Contrast "Wisp1 Slope Diff" Log_Total_GSH*TwoGroups 1 -1 Log_Total_GSH*TwoGroups*Protein  0 0 0 0 0 0 0 0 0 0 0 0 0 0 0 0 0 0 0 0
0 0 0 0 0 0 0 0 0 0 0 0 0 0 0 0 0 0 0 0 0 0 0 0 0 0 0 0 0 0 0 0 0 0 0 0 0 0 0 0 0 0 0 0 0 0 0 0 0 0 0 0 0 0 0 0 0 0 0 0 0 0 0 0 0
0 0 0 0 0 1 0 0 0 0 0 0 0 0 0 0 0 0 0 0 0 0 0 0 0 0 0 0 0 0 0 0 0 0 0 0 0 0 0 0 0 0 0 0 0 0 0 0 0 0 0 0 0 0 0 0 0 0 0 0 0 0 0 0 0
0 0 0 0 0 0 0 0 0 0 0 0 0 0 0 0 0 0 0 0 0 0 0 0 0 0 0 0 0 0 0 0 -1 0;
Contrast "Yes1 Slope Diff" Log_Total_GSH*TwoGroups 1 -1 Log_Total_GSH*TwoGroups*Protein  0 0 0 0 0 0 0 0 0 0 0 0 0 0 0 0 0 0 0 0
0 0 0 0 0 0 0 0 0 0 0 0 0 0 0 0 0 0 0 0 0 0 0 0 0 0 0 0 0 0 0 0 0 0 0 0 0 0 0 0 0 0 0 0 0 0 0 0 0 0 0 0 0 0 0 0 0 0 0 0 0 0 0 0 0
0 0 0 0 0 0 1 0 0 0 0 0 0 0 0 0 0 0 0 0 0 0 0 0 0 0 0 0 0 0 0 0 0 0 0 0 0 0 0 0 0 0 0 0 0 0 0 0 0 0 0 0 0 0 0 0 0 0 0 0 0 0 0 0 0
0 0 0 0 0 0 0 0 0 0 0 0 0 0 0 0 0 0 0 0 0 0 0 0 0 0 0 0 0 0 0 0 0 -1;

/*The following Estimates, give the regression slope for each protein in each age group,
including significance tests shown in the figure.*/
```

```
Estimate "Acvrl1 Old Slope" Log_Total_GSH 1 Log_Total_GSH*TwoGroups 1 0 Log_Total_GSH*Protein 1 0 0 0 0 0 0 0 0 0 0 0 0 0 0 0 0 0
0 0 0 0 0 0 0 0 0 0 0 0 0 0 0 0 0 0 0 0 0 0 0 0 0 0 0 0 0 0 0 0 0 0 0 0 0 0 0 0 0 0 0 0 0 0 0 0 0 0 0 0 0 0 0 0 0 0 0 0 0 0 0 0 0
0 0 0 0 0 0 0 0 0 Log_Total_GSH*TwoGroups*Protein  1 0 0 0 0 0 0 0 0 0 0 0 0 0 0 0 0 0 0 0 0 0 0 0 0 0 0 0 0 0 0 0 0 0 0 0 0 0 0
0 0 0 0 0 0 0 0 0 0 0 0 0 0 0 0 0 0 0 0 0 0 0 0 0 0 0 0 0 0 0 0 0 0 0 0 0 0 0 0 0 0 0 0 0 0 0 0 0 0 0 0 0 0 0 0 0 0 0 0 0 0 0 0 0
0 0 0 0 0 0 0 0 0 0 0 0 0 0 0 0 0 0 0 0 0 0 0 0 0 0 0 0 0 0 0 0 0 0 0 0 0 0 0 0 0 0 0 0 0 0 0 0 0 0 0 0 0 0 0 0 0 0 0 0 0 0 0 0 0
0 0 0 0 0 0 0 0 0 0 0 0 0 0 0;
Estimate "Adam23 Old Slope" Log_Total_GSH 1 Log_Total_GSH*TwoGroups 1 0 Log_Total_GSH*Protein 0 1 0 0 0 0 0 0 0 0 0 0 0 0 0 0 0 0
0 0 0 0 0 0 0 0 0 0 0 0 0 0 0 0 0 0 0 0 0 0 0 0 0 0 0 0 0 0 0 0 0 0 0 0 0 0 0 0 0 0 0 0 0 0 0 0 0 0 0 0 0 0 0 0 0 0 0 0 0 0 0 0 0
0 0 0 0 0 0 0 0 0 Log_Total_GSH*TwoGroups*Protein  0 1 0 0 0 0 0 0 0 0 0 0 0 0 0 0 0 0 0 0 0 0 0 0 0 0 0 0 0 0 0 0 0 0 0 0 0 0 0
0 0 0 0 0 0 0 0 0 0 0 0 0 0 0 0 0 0 0 0 0 0 0 0 0 0 0 0 0 0 0 0 0 0 0 0 0 0 0 0 0 0 0 0 0 0 0 0 0 0 0 0 0 0 0 0 0 0 0 0 0 0 0 0 0
0 0 0 0 0 0 0 0 0 0 0 0 0 0 0 0 0 0 0 0 0 0 0 0 0 0 0 0 0 0 0 0 0 0 0 0 0 0 0 0 0 0 0 0 0 0 0 0 0 0 0 0 0 0 0 0 0 0 0 0 0 0 0 0 0
0 0 0 0 0 0 0 0 0 0 0 0 0 0 0;
Estimate "Ahr Old Slope" Log_Total_GSH 1 Log_Total_GSH*TwoGroups 1 0 Log_Total_GSH*Protein 0 0 1 0 0 0 0 0 0 0 0 0 0 0 0 0 0 0 0
0 0 0 0 0 0 0 0 0 0 0 0 0 0 0 0 0 0 0 0 0 0 0 0 0 0 0 0 0 0 0 0 0 0 0 0 0 0 0 0 0 0 0 0 0 0 0 0 0 0 0 0 0 0 0 0 0 0 0 0 0 0 0 0 0
0 0 0 0 0 0 0 0 Log_Total_GSH*TwoGroups*Protein  0 0 1 0 0 0 0 0 0 0 0 0 0 0 0 0 0 0 0 0 0 0 0 0 0 0 0 0 0 0 0 0 0 0 0 0 0 0 0 0
0 0 0 0 0 0 0 0 0 0 0 0 0 0 0 0 0 0 0 0 0 0 0 0 0 0 0 0 0 0 0 0 0 0 0 0 0 0 0 0 0 0 0 0 0 0 0 0 0 0 0 0 0 0 0 0 0 0 0 0 0 0 0 0 0
0 0 0 0 0 0 0 0 0 0 0 0 0 0 0 0 0 0 0 0 0 0 0 0 0 0 0 0 0 0 0 0 0 0 0 0 0 0 0 0 0 0 0 0 0 0 0 0 0 0 0 0 0 0 0 0 0 0 0 0 0 0 0 0 0
0 0 0 0 0 0 0 0 0 0 0 0 0 0;
Estimate "Apbb1ip Old Slope" Log_Total_GSH 1 Log_Total_GSH*TwoGroups 1 0 Log_Total_GSH*Protein 0 0 0 1 0 0 0 0 0 0 0 0 0 0 0 0 0
0 0 0 0 0 0 0 0 0 0 0 0 0 0 0 0 0 0 0 0 0 0 0 0 0 0 0 0 0 0 0 0 0 0 0 0 0 0 0 0 0 0 0 0 0 0 0 0 0 0 0 0 0 0 0 0 0 0 0 0 0 0 0 0 0
0 0 0 0 0 0 0 0 0 0 Log_Total_GSH*TwoGroups*Protein  0 0 0 1 0 0 0 0 0 0 0 0 0 0 0 0 0 0 0 0 0 0 0 0 0 0 0 0 0 0 0 0 0 0 0 0 0 0
0 0 0 0 0 0 0 0 0 0 0 0 0 0 0 0 0 0 0 0 0 0 0 0 0 0 0 0 0 0 0 0 0 0 0 0 0 0 0 0 0 0 0 0 0 0 0 0 0 0 0 0 0 0 0 0 0 0 0 0 0 0 0 0 0
0 0 0 0 0 0 0 0 0 0 0 0 0 0 0 0 0 0 0 0 0 0 0 0 0 0 0 0 0 0 0 0 0 0 0 0 0 0 0 0 0 0 0 0 0 0 0 0 0 0 0 0 0 0 0 0 0 0 0 0 0 0 0 0 0
0 0 0 0 0 0 0 0 0 0 0 0 0 0 0 0;
Estimate "Axin1 Old Slope" Log_Total_GSH 1 Log_Total_GSH*TwoGroups 1 0 Log_Total_GSH*Protein 0 0 0 0 1 0 0 0 0 0 0 0 0 0 0 0 0 0
0 0 0 0 0 0 0 0 0 0 0 0 0 0 0 0 0 0 0 0 0 0 0 0 0 0 0 0 0 0 0 0 0 0 0 0 0 0 0 0 0 0 0 0 0 0 0 0 0 0 0 0 0 0 0 0 0 0 0 0 0 0 0 0 0
0 0 0 0 0 0 0 0 0 Log_Total_GSH*TwoGroups*Protein  0 0 0 0 1 0 0 0 0 0 0 0 0 0 0 0 0 0 0 0 0 0 0 0 0 0 0 0 0 0 0 0 0 0 0 0 0 0 0
0 0 0 0 0 0 0 0 0 0 0 0 0 0 0 0 0 0 0 0 0 0 0 0 0 0 0 0 0 0 0 0 0 0 0 0 0 0 0 0 0 0 0 0 0 0 0 0 0 0 0 0 0 0 0 0 0 0 0 0 0 0 0 0 0
0 0 0 0 0 0 0 0 0 0 0 0 0 0 0 0 0 0 0 0 0 0 0 0 0 0 0 0 0 0 0 0 0 0 0 0 0 0 0 0 0 0 0 0 0 0 0 0 0 0 0 0 0 0 0 0 0 0 0 0 0 0 0 0 0
0 0 0 0 0 0 0 0 0 0 0 0 0 0 0;
Estimate "Ca13 Old Slope" Log_Total_GSH 1 Log_Total_GSH*TwoGroups 1 0 Log_Total_GSH*Protein 0 0 0 0 0 1 0 0 0 0 0 0 0 0 0 0 0 0 0
0 0 0 0 0 0 0 0 0 0 0 0 0 0 0 0 0 0 0 0 0 0 0 0 0 0 0 0 0 0 0 0 0 0 0 0 0 0 0 0 0 0 0 0 0 0 0 0 0 0 0 0 0 0 0 0 0 0 0 0 0 0 0 0 0
0 0 0 0 0 0 0 0 Log_Total_GSH*TwoGroups*Protein  0 0 0 0 0 1 0 0 0 0 0 0 0 0 0 0 0 0 0 0 0 0 0 0 0 0 0 0 0 0 0 0 0 0 0 0 0 0 0 0
0 0 0 0 0 0 0 0 0 0 0 0 0 0 0 0 0 0 0 0 0 0 0 0 0 0 0 0 0 0 0 0 0 0 0 0 0 0 0 0 0 0 0 0 0 0 0 0 0 0 0 0 0 0 0 0 0 0 0 0 0 0 0 0 0
0 0 0 0 0 0 0 0 0 0 0 0 0 0 0 0 0 0 0 0 0 0 0 0 0 0 0 0 0 0 0 0 0 0 0 0 0 0 0 0 0 0 0 0 0 0 0 0 0 0 0 0 0 0 0 0 0 0 0 0 0 0 0 0 0
0 0 0 0 0 0 0 0 0 0 0 0 0 0;
Estimate "Cant1 Old Slope" Log_Total_GSH 1 Log_Total_GSH*TwoGroups 1 0 Log_Total_GSH*Protein 0 0 0 0 0 0 1 0 0 0 0 0 0 0 0 0 0 0
0 0 0 0 0 0 0 0 0 0 0 0 0 0 0 0 0 0 0 0 0 0 0 0 0 0 0 0 0 0 0 0 0 0 0 0 0 0 0 0 0 0 0 0 0 0 0 0 0 0 0 0 0 0 0 0 0 0 0 0 0 0 0 0 0
0 0 0 0 0 0 0 0 0 Log_Total_GSH*TwoGroups*Protein  0 0 0 0 0 0 1 0 0 0 0 0 0 0 0 0 0 0 0 0 0 0 0 0 0 0 0 0 0 0 0 0 0 0 0 0 0 0 0
0 0 0 0 0 0 0 0 0 0 0 0 0 0 0 0 0 0 0 0 0 0 0 0 0 0 0 0 0 0 0 0 0 0 0 0 0 0 0 0 0 0 0 0 0 0 0 0 0 0 0 0 0 0 0 0 0 0 0 0 0 0 0 0 0
0 0 0 0 0 0 0 0 0 0 0 0 0 0 0 0 0 0 0 0 0 0 0 0 0 0 0 0 0 0 0 0 0 0 0 0 0 0 0 0 0 0 0 0 0 0 0 0 0 0 0 0 0 0 0 0 0 0 0 0 0 0 0 0 0
0 0 0 0 0 0 0 0 0 0 0 0 0 0 0;
Estimate "Casp3 Old Slope" Log_Total_GSH 1 Log_Total_GSH*TwoGroups 1 0 Log_Total_GSH*Protein 0 0 0 0 0 0 0 1 0 0 0 0 0 0 0 0 0 0
0 0 0 0 0 0 0 0 0 0 0 0 0 0 0 0 0 0 0 0 0 0 0 0 0 0 0 0 0 0 0 0 0 0 0 0 0 0 0 0 0 0 0 0 0 0 0 0 0 0 0 0 0 0 0 0 0 0 0 0 0 0 0 0 0
0 0 0 0 0 0 0 0 0 Log_Total_GSH*TwoGroups*Protein  0 0 0 0 0 0 0 1 0 0 0 0 0 0 0 0 0 0 0 0 0 0 0 0 0 0 0 0 0 0 0 0 0 0 0 0 0 0 0
0 0 0 0 0 0 0 0 0 0 0 0 0 0 0 0 0 0 0 0 0 0 0 0 0 0 0 0 0 0 0 0 0 0 0 0 0 0 0 0 0 0 0 0 0 0 0 0 0 0 0 0 0 0 0 0 0 0 0 0 0 0 0 0 0
0 0 0 0 0 0 0 0 0 0 0 0 0 0 0 0 0 0 0 0 0 0 0 0 0 0 0 0 0 0 0 0 0 0 0 0 0 0 0 0 0 0 0 0 0 0 0 0 0 0 0 0 0 0 0 0 0 0 0 0 0 0 0 0 0
0 0 0 0 0 0 0 0 0 0 0 0 0 0 0;
Estimate "Ccl2 Old Slope" Log_Total_GSH 1 Log_Total_GSH*TwoGroups 1 0 Log_Total_GSH*Protein 0 0 0 0 0 0 0 0 1 0 0 0 0 0 0 0 0 0 0
0 0 0 0 0 0 0 0 0 0 0 0 0 0 0 0 0 0 0 0 0 0 0 0 0 0 0 0 0 0 0 0 0 0 0 0 0 0 0 0 0 0 0 0 0 0 0 0 0 0 0 0 0 0 0 0 0 0 0 0 0 0 0 0 0
0 0 0 0 0 0 0 0 Log_Total_GSH*TwoGroups*Protein  0 0 0 0 0 0 0 0 1 0 0 0 0 0 0 0 0 0 0 0 0 0 0 0 0 0 0 0 0 0 0 0 0 0 0 0 0 0 0 0
0 0 0 0 0 0 0 0 0 0 0 0 0 0 0 0 0 0 0 0 0 0 0 0 0 0 0 0 0 0 0 0 0 0 0 0 0 0 0 0 0 0 0 0 0 0 0 0 0 0 0 0 0 0 0 0 0 0 0 0 0 0 0 0 0
0 0 0 0 0 0 0 0 0 0 0 0 0 0 0 0 0 0 0 0 0 0 0 0 0 0 0 0 0 0 0 0 0 0 0 0 0 0 0 0 0 0 0 0 0 0 0 0 0 0 0 0 0 0 0 0 0 0 0 0 0 0 0 0 0
0 0 0 0 0 0 0 0 0 0 0 0 0 0;
Estimate "Ccl20 Old Slope" Log_Total_GSH 1 Log_Total_GSH*TwoGroups 1 0 Log_Total_GSH*Protein 0 0 0 0 0 0 0 0 0 1 0 0 0 0 0 0 0 0
0 0 0 0 0 0 0 0 0 0 0 0 0 0 0 0 0 0 0 0 0 0 0 0 0 0 0 0 0 0 0 0 0 0 0 0 0 0 0 0 0 0 0 0 0 0 0 0 0 0 0 0 0 0 0 0 0 0 0 0 0 0 0 0 0
0 0 0 0 0 0 0 0 0 Log_Total_GSH*TwoGroups*Protein  0 0 0 0 0 0 0 0 0 1 0 0 0 0 0 0 0 0 0 0 0 0 0 0 0 0 0 0 0 0 0 0 0 0 0 0 0 0 0
0 0 0 0 0 0 0 0 0 0 0 0 0 0 0 0 0 0 0 0 0 0 0 0 0 0 0 0 0 0 0 0 0 0 0 0 0 0 0 0 0 0 0 0 0 0 0 0 0 0 0 0 0 0 0 0 0 0 0 0 0 0 0 0 0
0 0 0 0 0 0 0 0 0 0 0 0 0 0 0 0 0 0 0 0 0 0 0 0 0 0 0 0 0 0 0 0 0 0 0 0 0 0 0 0 0 0 0 0 0 0 0 0 0 0 0 0 0 0 0 0 0 0 0 0 0 0 0 0 0
0 0 0 0 0 0 0 0 0 0 0 0 0 0 0;
Estimate "Ccl3 Old Slope" Log_Total_GSH 1 Log_Total_GSH*TwoGroups 1 0 Log_Total_GSH*Protein 0 0 0 0 0 0 0 0 0 0 1 0 0 0 0 0 0 0 0
0 0 0 0 0 0 0 0 0 0 0 0 0 0 0 0 0 0 0 0 0 0 0 0 0 0 0 0 0 0 0 0 0 0 0 0 0 0 0 0 0 0 0 0 0 0 0 0 0 0 0 0 0 0 0 0 0 0 0 0 0 0 0 0 0
0 0 0 0 0 0 0 0 Log_Total_GSH*TwoGroups*Protein  0 0 0 0 0 0 0 0 0 0 1 0 0 0 0 0 0 0 0 0 0 0 0 0 0 0 0 0 0 0 0 0 0 0 0 0 0 0 0 0
0 0 0 0 0 0 0 0 0 0 0 0 0 0 0 0 0 0 0 0 0 0 0 0 0 0 0 0 0 0 0 0 0 0 0 0 0 0 0 0 0 0 0 0 0 0 0 0 0 0 0 0 0 0 0 0 0 0 0 0 0 0 0 0 0
0 0 0 0 0 0 0 0 0 0 0 0 0 0 0 0 0 0 0 0 0 0 0 0 0 0 0 0 0 0 0 0 0 0 0 0 0 0 0 0 0 0 0 0 0 0 0 0 0 0 0 0 0 0 0 0 0 0 0 0 0 0 0 0 0
0 0 0 0 0 0 0 0 0 0 0 0 0 0;
Estimate "Ccl5 Old Slope" Log_Total_GSH 1 Log_Total_GSH*TwoGroups 1 0 Log_Total_GSH*Protein 0 0 0 0 0 0 0 0 0 0 0 1 0 0 0 0 0 0 0
0 0 0 0 0 0 0 0 0 0 0 0 0 0 0 0 0 0 0 0 0 0 0 0 0 0 0 0 0 0 0 0 0 0 0 0 0 0 0 0 0 0 0 0 0 0 0 0 0 0 0 0 0 0 0 0 0 0 0 0 0 0 0 0 0
0 0 0 0 0 0 0 0 Log_Total_GSH*TwoGroups*Protein  0 0 0 0 0 0 0 0 0 0 0 1 0 0 0 0 0 0 0 0 0 0 0 0 0 0 0 0 0 0 0 0 0 0 0 0 0 0 0 0
0 0 0 0 0 0 0 0 0 0 0 0 0 0 0 0 0 0 0 0 0 0 0 0 0 0 0 0 0 0 0 0 0 0 0 0 0 0 0 0 0 0 0 0 0 0 0 0 0 0 0 0 0 0 0 0 0 0 0 0 0 0 0 0 0
0 0 0 0 0 0 0 0 0 0 0 0 0 0 0 0 0 0 0 0 0 0 0 0 0 0 0 0 0 0 0 0 0 0 0 0 0 0 0 0 0 0 0 0 0 0 0 0 0 0 0 0 0 0 0 0 0 0 0 0 0 0 0 0 0
0 0 0 0 0 0 0 0 0 0 0 0 0 0;
Estimate "Cdh6 Old Slope" Log_Total_GSH 1 Log_Total_GSH*TwoGroups 1 0 Log_Total_GSH*Protein 0 0 0 0 0 0 0 0 0 0 0 0 1 0 0 0 0 0 0
0 0 0 0 0 0 0 0 0 0 0 0 0 0 0 0 0 0 0 0 0 0 0 0 0 0 0 0 0 0 0 0 0 0 0 0 0 0 0 0 0 0 0 0 0 0 0 0 0 0 0 0 0 0 0 0 0 0 0 0 0 0 0 0 0
0 0 0 0 0 0 0 0 Log_Total_GSH*TwoGroups*Protein  0 0 0 0 0 0 0 0 0 0 0 0 1 0 0 0 0 0 0 0 0 0 0 0 0 0 0 0 0 0 0 0 0 0 0 0 0 0 0 0
0 0 0 0 0 0 0 0 0 0 0 0 0 0 0 0 0 0 0 0 0 0 0 0 0 0 0 0 0 0 0 0 0 0 0 0 0 0 0 0 0 0 0 0 0 0 0 0 0 0 0 0 0 0 0 0 0 0 0 0 0 0 0 0 0
0 0 0 0 0 0 0 0 0 0 0 0 0 0 0 0 0 0 0 0 0 0 0 0 0 0 0 0 0 0 0 0 0 0 0 0 0 0 0 0 0 0 0 0 0 0 0 0 0 0 0 0 0 0 0 0 0 0 0 0 0 0 0 0 0
0 0 0 0 0 0 0 0 0 0 0 0 0 0;
Estimate "Clmp Old Slope" Log_Total_GSH 1 Log_Total_GSH*TwoGroups 1 0 Log_Total_GSH*Protein 0 0 0 0 0 0 0 0 0 0 0 0 0 1 0 0 0 0 0
0 0 0 0 0 0 0 0 0 0 0 0 0 0 0 0 0 0 0 0 0 0 0 0 0 0 0 0 0 0 0 0 0 0 0 0 0 0 0 0 0 0 0 0 0 0 0 0 0 0 0 0 0 0 0 0 0 0 0 0 0 0 0 0 0
0 0 0 0 0 0 0 0 Log_Total_GSH*TwoGroups*Protein  0 0 0 0 0 0 0 0 0 0 0 0 0 1 0 0 0 0 0 0 0 0 0 0 0 0 0 0 0 0 0 0 0 0 0 0 0 0 0 0
0 0 0 0 0 0 0 0 0 0 0 0 0 0 0 0 0 0 0 0 0 0 0 0 0 0 0 0 0 0 0 0 0 0 0 0 0 0 0 0 0 0 0 0 0 0 0 0 0 0 0 0 0 0 0 0 0 0 0 0 0 0 0 0 0
0 0 0 0 0 0 0 0 0 0 0 0 0 0 0 0 0 0 0 0 0 0 0 0 0 0 0 0 0 0 0 0 0 0 0 0 0 0 0 0 0 0 0 0 0 0 0 0 0 0 0 0 0 0 0 0 0 0 0 0 0 0 0 0 0
0 0 0 0 0 0 0 0 0 0 0 0 0 0;
Estimate "Clstn2 Old Slope" Log_Total_GSH 1 Log_Total_GSH*TwoGroups 1 0 Log_Total_GSH*Protein 0 0 0 0 0 0 0 0 0 0 0 0 0 0 1 0 0 0
0 0 0 0 0 0 0 0 0 0 0 0 0 0 0 0 0 0 0 0 0 0 0 0 0 0 0 0 0 0 0 0 0 0 0 0 0 0 0 0 0 0 0 0 0 0 0 0 0 0 0 0 0 0 0 0 0 0 0 0 0 0 0 0 0
0 0 0 0 0 0 0 0 0 Log_Total_GSH*TwoGroups*Protein  0 0 0 0 0 0 0 0 0 0 0 0 0 0 1 0 0 0 0 0 0 0 0 0 0 0 0 0 0 0 0 0 0 0 0 0 0 0 0
0 0 0 0 0 0 0 0 0 0 0 0 0 0 0 0 0 0 0 0 0 0 0 0 0 0 0 0 0 0 0 0 0 0 0 0 0 0 0 0 0 0 0 0 0 0 0 0 0 0 0 0 0 0 0 0 0 0 0 0 0 0 0 0 0
0 0 0 0 0 0 0 0 0 0 0 0 0 0 0 0 0 0 0 0 0 0 0 0 0 0 0 0 0 0 0 0 0 0 0 0 0 0 0 0 0 0 0 0 0 0 0 0 0 0 0 0 0 0 0 0 0 0 0 0 0 0 0 0 0
0 0 0 0 0 0 0 0 0 0 0 0 0 0 0;
Estimate "Cntn1 Old Slope" Log_Total_GSH 1 Log_Total_GSH*TwoGroups 1 0 Log_Total_GSH*Protein 0 0 0 0 0 0 0 0 0 0 0 0 0 0 0 1 0 0
0 0 0 0 0 0 0 0 0 0 0 0 0 0 0 0 0 0 0 0 0 0 0 0 0 0 0 0 0 0 0 0 0 0 0 0 0 0 0 0 0 0 0 0 0 0 0 0 0 0 0 0 0 0 0 0 0 0 0 0 0 0 0 0 0
0 0 0 0 0 0 0 0 0 Log_Total_GSH*TwoGroups*Protein  0 0 0 0 0 0 0 0 0 0 0 0 0 0 0 1 0 0 0 0 0 0 0 0 0 0 0 0 0 0 0 0 0 0 0 0 0 0 0
0 0 0 0 0 0 0 0 0 0 0 0 0 0 0 0 0 0 0 0 0 0 0 0 0 0 0 0 0 0 0 0 0 0 0 0 0 0 0 0 0 0 0 0 0 0 0 0 0 0 0 0 0 0 0 0 0 0 0 0 0 0 0 0 0
```

```
0 0 0 0 0 0 0 0 0 0 0 0 0 0 0 0 0 0 0 0 0 0 0 0 0 0 0 0 0 0 0 0 0 0 0 0 0 0 0 0 0 0 0 0 0 0 0 0 0 0 0 0 0 0 0 0 0 0 0 0 0 0 0 0 0
0 0 0 0 0 0 0 0 0 0 0 0 0 0 0;
Estimate "Cntn4 Old Slope" Log_Total_GSH 1 Log_Total_GSH*TwoGroups 1 0 Log_Total_GSH*Protein 0 0 0 0 0 0 0 0 0 0 0 0 0 0 0 0 1 0
0 0 0 0 0 0 0 0 0 0 0 0 0 0 0 0 0 0 0 0 0 0 0 0 0 0 0 0 0 0 0 0 0 0 0 0 0 0 0 0 0 0 0 0 0 0 0 0 0 0 0 0 0 0 0 0 0 0 0 0 0 0 0 0 0
0 0 0 0 0 0 0 0 0 Log_Total_GSH*TwoGroups*Protein  0 0 0 0 0 0 0 0 0 0 0 0 0 0 0 0 1 0 0 0 0 0 0 0 0 0 0 0 0 0 0 0 0 0 0 0 0 0
0 0 0 0 0 0 0 0 0 0 0 0 0 0 0 0 0 0 0 0 0 0 0 0 0 0 0 0 0 0 0 0 0 0 0 0 0 0 0 0 0 0 0 0 0 0 0 0 0 0 0 0 0 0 0 0 0 0 0 0 0 0 0 0 0
0 0 0 0 0 0 0 0 0 0 0 0 0 0 0 0 0 0 0 0 0 0 0 0 0 0 0 0 0 0 0 0 0 0 0 0 0 0 0 0 0 0 0 0 0 0 0 0 0 0 0 0 0 0 0 0 0 0 0 0 0 0 0 0 0
0 0 0 0 0 0 0 0 0 0 0 0 0 0 0;
Estimate "Cpe Old Slope" Log_Total_GSH 1 Log_Total_GSH*TwoGroups 1 0 Log_Total_GSH*Protein 0 0 0 0 0 0 0 0 0 0 0 0 0 0 0 0 0 1 0
0 0 0 0 0 0 0 0 0 0 0 0 0 0 0 0 0 0 0 0 0 0 0 0 0 0 0 0 0 0 0 0 0 0 0 0 0 0 0 0 0 0 0 0 0 0 0 0 0 0 0 0 0 0 0 0 0 0 0 0 0 0 0 0 0
0 0 0 0 0 0 0 0 Log_Total_GSH*TwoGroups*Protein  0 0 0 0 0 0 0 0 0 0 0 0 0 0 0 0 0 1 0 0 0 0 0 0 0 0 0 0 0 0 0 0 0 0 0 0 0 0 0 0
0 0 0 0 0 0 0 0 0 0 0 0 0 0 0 0 0 0 0 0 0 0 0 0 0 0 0 0 0 0 0 0 0 0 0 0 0 0 0 0 0 0 0 0 0 0 0 0 0 0 0 0 0 0 0 0 0 0 0 0 0 0 0 0 0
0 0 0 0 0 0 0 0 0 0 0 0 0 0 0 0 0 0 0 0 0 0 0 0 0 0 0 0 0 0 0 0 0 0 0 0 0 0 0 0 0 0 0 0 0 0 0 0 0 0 0 0 0 0 0 0 0 0 0 0 0 0 0 0 0
0 0 0 0 0 0 0 0 0 0 0 0 0 0;
Estimate "Crim1 Old Slope" Log_Total_GSH 1 Log_Total_GSH*TwoGroups 1 0 Log_Total_GSH*Protein 0 0 0 0 0 0 0 0 0 0 0 0 0 0 0 0 0 0
1 0 0 0 0 0 0 0 0 0 0 0 0 0 0 0 0 0 0 0 0 0 0 0 0 0 0 0 0 0 0 0 0 0 0 0 0 0 0 0 0 0 0 0 0 0 0 0 0 0 0 0 0 0 0 0 0 0 0 0 0 0 0 0 0
0 0 0 0 0 0 0 0 0 Log_Total_GSH*TwoGroups*Protein  0 0 0 0 0 0 0 0 0 0 0 0 0 0 0 0 0 0 1 0 0 0 0 0 0 0 0 0 0 0 0 0 0 0 0 0 0 0 0 0
0 0 0 0 0 0 0 0 0 0 0 0 0 0 0 0 0 0 0 0 0 0 0 0 0 0 0 0 0 0 0 0 0 0 0 0 0 0 0 0 0 0 0 0 0 0 0 0 0 0 0 0 0 0 0 0 0 0 0 0 0 0 0 0 0
0 0 0 0 0 0 0 0 0 0 0 0 0 0 0 0 0 0 0 0 0 0 0 0 0 0 0 0 0 0 0 0 0 0 0 0 0 0 0 0 0 0 0 0 0 0 0 0 0 0 0 0 0 0 0 0 0 0 0 0 0 0 0 0 0
0 0 0 0 0 0 0 0 0 0 0 0 0 0 0;
Estimate "Csf2 Old Slope" Log_Total_GSH 1 Log_Total_GSH*TwoGroups 1 0 Log_Total_GSH*Protein 0 0 0 0 0 0 0 0 0 0 0 0 0 0 0 0 0 0 0
1 0 0 0 0 0 0 0 0 0 0 0 0 0 0 0 0 0 0 0 0 0 0 0 0 0 0 0 0 0 0 0 0 0 0 0 0 0 0 0 0 0 0 0 0 0 0 0 0 0 0 0 0 0 0 0 0 0 0 0 0 0 0 0 0
0 0 0 0 0 0 0 0 Log_Total_GSH*TwoGroups*Protein  0 0 0 0 0 0 0 0 0 0 0 0 0 0 0 0 0 0 0 1 0 0 0 0 0 0 0 0 0 0 0 0 0 0 0 0 0 0 0 0
0 0 0 0 0 0 0 0 0 0 0 0 0 0 0 0 0 0 0 0 0 0 0 0 0 0 0 0 0 0 0 0 0 0 0 0 0 0 0 0 0 0 0 0 0 0 0 0 0 0 0 0 0 0 0 0 0 0 0 0 0 0 0 0 0
0 0 0 0 0 0 0 0 0 0 0 0 0 0 0 0 0 0 0 0 0 0 0 0 0 0 0 0 0 0 0 0 0 0 0 0 0 0 0 0 0 0 0 0 0 0 0 0 0 0 0 0 0 0 0 0 0 0 0 0 0 0 0 0 0
0 0 0 0 0 0 0 0 0 0 0 0 0 0;
Estimate "Cxcl1 Old Slope" Log_Total_GSH 1 Log_Total_GSH*TwoGroups 1 0 Log_Total_GSH*Protein 0 0 0 0 0 0 0 0 0 0 0 0 0 0 0 0 0 0
0 0 1 0 0 0 0 0 0 0 0 0 0 0 0 0 0 0 0 0 0 0 0 0 0 0 0 0 0 0 0 0 0 0 0 0 0 0 0 0 0 0 0 0 0 0 0 0 0 0 0 0 0 0 0 0 0 0 0 0 0 0 0 0 0
0 0 0 0 0 0 0 0 0 Log_Total_GSH*TwoGroups*Protein  0 0 0 0 0 0 0 0 0 0 0 0 0 0 0 0 0 0 0 0 1 0 0 0 0 0 0 0 0 0 0 0 0 0 0 0 0 0 0
0 0 0 0 0 0 0 0 0 0 0 0 0 0 0 0 0 0 0 0 0 0 0 0 0 0 0 0 0 0 0 0 0 0 0 0 0 0 0 0 0 0 0 0 0 0 0 0 0 0 0 0 0 0 0 0 0 0 0 0 0 0 0 0 0
0 0 0 0 0 0 0 0 0 0 0 0 0 0 0 0 0 0 0 0 0 0 0 0 0 0 0 0 0 0 0 0 0 0 0 0 0 0 0 0 0 0 0 0 0 0 0 0 0 0 0 0 0 0 0 0 0 0 0 0 0 0 0 0 0
0 0 0 0 0 0 0 0 0 0 0 0 0 0 0;
Estimate "Cxcl9 Old Slope" Log_Total_GSH 1 Log_Total_GSH*TwoGroups 1 0 Log_Total_GSH*Protein 0 0 0 0 0 0 0 0 0 0 0 0 0 0 0 0 0 0
0 0 0 1 0 0 0 0 0 0 0 0 0 0 0 0 0 0 0 0 0 0 0 0 0 0 0 0 0 0 0 0 0 0 0 0 0 0 0 0 0 0 0 0 0 0 0 0 0 0 0 0 0 0 0 0 0 0 0 0 0 0 0 0 0
0 0 0 0 0 0 0 0 0 Log_Total_GSH*TwoGroups*Protein  0 0 0 0 0 0 0 0 0 0 0 0 0 0 0 0 0 0 0 0 0 1 0 0 0 0 0 0 0 0 0 0 0 0 0 0 0 0 0
0 0 0 0 0 0 0 0 0 0 0 0 0 0 0 0 0 0 0 0 0 0 0 0 0 0 0 0 0 0 0 0 0 0 0 0 0 0 0 0 0 0 0 0 0 0 0 0 0 0 0 0 0 0 0 0 0 0 0 0 0 0 0 0 0
0 0 0 0 0 0 0 0 0 0 0 0 0 0 0 0 0 0 0 0 0 0 0 0 0 0 0 0 0 0 0 0 0 0 0 0 0 0 0 0 0 0 0 0 0 0 0 0 0 0 0 0 0 0 0 0 0 0 0 0 0 0 0 0 0
0 0 0 0 0 0 0 0 0 0 0 0 0 0 0;
Estimate "Cyr61 Old Slope" Log_Total_GSH 1 Log_Total_GSH*TwoGroups 1 0 Log_Total_GSH*Protein 0 0 0 0 0 0 0 0 0 0 0 0 0 0 0 0 0 0
0 0 0 0 1 0 0 0 0 0 0 0 0 0 0 0 0 0 0 0 0 0 0 0 0 0 0 0 0 0 0 0 0 0 0 0 0 0 0 0 0 0 0 0 0 0 0 0 0 0 0 0 0 0 0 0 0 0 0 0 0 0 0 0 0
0 0 0 0 0 0 0 0 0 Log_Total_GSH*TwoGroups*Protein  0 0 0 0 0 0 0 0 0 0 0 0 0 0 0 0 0 0 0 0 0 0 1 0 0 0 0 0 0 0 0 0 0 0 0 0 0 0 0
0 0 0 0 0 0 0 0 0 0 0 0 0 0 0 0 0 0 0 0 0 0 0 0 0 0 0 0 0 0 0 0 0 0 0 0 0 0 0 0 0 0 0 0 0 0 0 0 0 0 0 0 0 0 0 0 0 0 0 0 0 0 0 0 0
0 0 0 0 0 0 0 0 0 0 0 0 0 0 0 0 0 0 0 0 0 0 0 0 0 0 0 0 0 0 0 0 0 0 0 0 0 0 0 0 0 0 0 0 0 0 0 0 0 0 0 0 0 0 0 0 0 0 0 0 0 0 0 0 0
0 0 0 0 0 0 0 0 0 0 0 0 0 0 0;
Estimate "Dctn2 Old Slope" Log_Total_GSH 1 Log_Total_GSH*TwoGroups 1 0 Log_Total_GSH*Protein 0 0 0 0 0 0 0 0 0 0 0 0 0 0 0 0 0 0
0 0 0 0 0 1 0 0 0 0 0 0 0 0 0 0 0 0 0 0 0 0 0 0 0 0 0 0 0 0 0 0 0 0 0 0 0 0 0 0 0 0 0 0 0 0 0 0 0 0 0 0 0 0 0 0 0 0 0 0 0 0 0 0 0
0 0 0 0 0 0 0 0 0 Log_Total_GSH*TwoGroups*Protein  0 0 0 0 0 0 0 0 0 0 0 0 0 0 0 0 0 0 0 0 0 0 0 1 0 0 0 0 0 0 0 0 0 0 0 0 0 0 0
0 0 0 0 0 0 0 0 0 0 0 0 0 0 0 0 0 0 0 0 0 0 0 0 0 0 0 0 0 0 0 0 0 0 0 0 0 0 0 0 0 0 0 0 0 0 0 0 0 0 0 0 0 0 0 0 0 0 0 0 0 0 0 0 0
0 0 0 0 0 0 0 0 0 0 0 0 0 0 0 0 0 0 0 0 0 0 0 0 0 0 0 0 0 0 0 0 0 0 0 0 0 0 0 0 0 0 0 0 0 0 0 0 0 0 0 0 0 0 0 0 0 0 0 0 0 0 0 0 0
0 0 0 0 0 0 0 0 0 0 0 0 0 0 0;
Estimate "Ddah1 Old Slope" Log_Total_GSH 1 Log_Total_GSH*TwoGroups 1 0 Log_Total_GSH*Protein 0 0 0 0 0 0 0 0 0 0 0 0 0 0 0 0 0 0
0 0 0 0 0 0 1 0 0 0 0 0 0 0 0 0 0 0 0 0 0 0 0 0 0 0 0 0 0 0 0 0 0 0 0 0 0 0 0 0 0 0 0 0 0 0 0 0 0 0 0 0 0 0 0 0 0 0 0 0 0 0 0 0 0
0 0 0 0 0 0 0 0 0 Log_Total_GSH*TwoGroups*Protein  0 0 0 0 0 0 0 0 0 0 0 0 0 0 0 0 0 0 0 0 0 0 0 0 1 0 0 0 0 0 0 0 0 0 0 0 0 0 0
0 0 0 0 0 0 0 0 0 0 0 0 0 0 0 0 0 0 0 0 0 0 0 0 0 0 0 0 0 0 0 0 0 0 0 0 0 0 0 0 0 0 0 0 0 0 0 0 0 0 0 0 0 0 0 0 0 0 0 0 0 0 0 0 0
0 0 0 0 0 0 0 0 0 0 0 0 0 0 0 0 0 0 0 0 0 0 0 0 0 0 0 0 0 0 0 0 0 0 0 0 0 0 0 0 0 0 0 0 0 0 0 0 0 0 0 0 0 0 0 0 0 0 0 0 0 0 0 0 0
0 0 0 0 0 0 0 0 0 0 0 0 0 0 0;
Estimate "Dlk1 Old Slope" Log_Total_GSH 1 Log_Total_GSH*TwoGroups 1 0 Log_Total_GSH*Protein 0 0 0 0 0 0 0 0 0 0 0 0 0 0 0 0 0 0 0
0 0 0 0 0 0 1 0 0 0 0 0 0 0 0 0 0 0 0 0 0 0 0 0 0 0 0 0 0 0 0 0 0 0 0 0 0 0 0 0 0 0 0 0 0 0 0 0 0 0 0 0 0 0 0 0 0 0 0 0 0 0 0 0 0
0 0 0 0 0 0 0 0 Log_Total_GSH*TwoGroups*Protein  0 0 0 0 0 0 0 0 0 0 0 0 0 0 0 0 0 0 0 0 0 0 0 0 0 1 0 0 0 0 0 0 0 0 0 0 0 0 0 0
0 0 0 0 0 0 0 0 0 0 0 0 0 0 0 0 0 0 0 0 0 0 0 0 0 0 0 0 0 0 0 0 0 0 0 0 0 0 0 0 0 0 0 0 0 0 0 0 0 0 0 0 0 0 0 0 0 0 0 0 0 0 0 0 0
0 0 0 0 0 0 0 0 0 0 0 0 0 0 0 0 0 0 0 0 0 0 0 0 0 0 0 0 0 0 0 0 0 0 0 0 0 0 0 0 0 0 0 0 0 0 0 0 0 0 0 0 0 0 0 0 0 0 0 0 0 0 0 0 0
0 0 0 0 0 0 0 0 0 0 0 0 0 0;
Estimate "Dll1 Old Slope" Log_Total_GSH 1 Log_Total_GSH*TwoGroups 1 0 Log_Total_GSH*Protein 0 0 0 0 0 0 0 0 0 0 0 0 0 0 0 0 0 0 0
0 0 0 0 0 0 0 1 0 0 0 0 0 0 0 0 0 0 0 0 0 0 0 0 0 0 0 0 0 0 0 0 0 0 0 0 0 0 0 0 0 0 0 0 0 0 0 0 0 0 0 0 0 0 0 0 0 0 0 0 0 0 0 0 0
0 0 0 0 0 0 0 0 Log_Total_GSH*TwoGroups*Protein  0 0 0 0 0 0 0 0 0 0 0 0 0 0 0 0 0 0 0 0 0 0 0 0 0 0 1 0 0 0 0 0 0 0 0 0 0 0 0 0
0 0 0 0 0 0 0 0 0 0 0 0 0 0 0 0 0 0 0 0 0 0 0 0 0 0 0 0 0 0 0 0 0 0 0 0 0 0 0 0 0 0 0 0 0 0 0 0 0 0 0 0 0 0 0 0 0 0 0 0 0 0 0 0 0
0 0 0 0 0 0 0 0 0 0 0 0 0 0 0 0 0 0 0 0 0 0 0 0 0 0 0 0 0 0 0 0 0 0 0 0 0 0 0 0 0 0 0 0 0 0 0 0 0 0 0 0 0 0 0 0 0 0 0 0 0 0 0 0 0
0 0 0 0 0 0 0 0 0 0 0 0 0 0;
Estimate "Eda2r Old Slope" Log_Total_GSH 1 Log_Total_GSH*TwoGroups 1 0 Log_Total_GSH*Protein 0 0 0 0 0 0 0 0 0 0 0 0 0 0 0 0 0 0
0 0 0 0 0 0 0 0 0 1 0 0 0 0 0 0 0 0 0 0 0 0 0 0 0 0 0 0 0 0 0 0 0 0 0 0 0 0 0 0 0 0 0 0 0 0 0 0 0 0 0 0 0 0 0 0 0 0 0 0 0 0 0 0 0
0 0 0 0 0 0 0 0 0 Log_Total_GSH*TwoGroups*Protein  0 0 0 0 0 0 0 0 0 0 0 0 0 0 0 0 0 0 0 0 0 0 0 0 0 0 0 1 0 0 0 0 0 0 0 0 0 0 0
0 0 0 0 0 0 0 0 0 0 0 0 0 0 0 0 0 0 0 0 0 0 0 0 0 0 0 0 0 0 0 0 0 0 0 0 0 0 0 0 0 0 0 0 0 0 0 0 0 0 0 0 0 0 0 0 0 0 0 0 0 0 0 0 0
0 0 0 0 0 0 0 0 0 0 0 0 0 0 0 0 0 0 0 0 0 0 0 0 0 0 0 0 0 0 0 0 0 0 0 0 0 0 0 0 0 0 0 0 0 0 0 0 0 0 0 0 0 0 0 0 0 0 0 0 0 0 0 0 0
0 0 0 0 0 0 0 0 0 0 0 0 0 0 0;
Estimate "Eno2 Old Slope" Log_Total_GSH 1 Log_Total_GSH*TwoGroups 1 0 Log_Total_GSH*Protein 0 0 0 0 0 0 0 0 0 0 0 0 0 0 0 0 0 0 0
0 0 0 0 0 0 0 0 0 1 0 0 0 0 0 0 0 0 0 0 0 0 0 0 0 0 0 0 0 0 0 0 0 0 0 0 0 0 0 0 0 0 0 0 0 0 0 0 0 0 0 0 0 0 0 0 0 0 0 0 0 0 0 0 0
0 0 0 0 0 0 0 0 Log_Total_GSH*TwoGroups*Protein  0 0 0 0 0 0 0 0 0 0 0 0 0 0 0 0 0 0 0 0 0 0 0 0 0 0 0 0 1 0 0 0 0 0 0 0 0 0 0 0
0 0 0 0 0 0 0 0 0 0 0 0 0 0 0 0 0 0 0 0 0 0 0 0 0 0 0 0 0 0 0 0 0 0 0 0 0 0 0 0 0 0 0 0 0 0 0 0 0 0 0 0 0 0 0 0 0 0 0 0 0 0 0 0 0
0 0 0 0 0 0 0 0 0 0 0 0 0 0 0 0 0 0 0 0 0 0 0 0 0 0 0 0 0 0 0 0 0 0 0 0 0 0 0 0 0 0 0 0 0 0 0 0 0 0 0 0 0 0 0 0 0 0 0 0 0 0 0 0 0
0 0 0 0 0 0 0 0 0 0 0 0 0 0;
Estimate "Epcam Old Slope" Log_Total_GSH 1 Log_Total_GSH*TwoGroups 1 0 Log_Total_GSH*Protein 0 0 0 0 0 0 0 0 0 0 0 0 0 0 0 0 0 0
0 0 0 0 0 0 0 0 0 0 0 1 0 0 0 0 0 0 0 0 0 0 0 0 0 0 0 0 0 0 0 0 0 0 0 0 0 0 0 0 0 0 0 0 0 0 0 0 0 0 0 0 0 0 0 0 0 0 0 0 0 0 0 0 0
0 0 0 0 0 0 0 0 0 Log_Total_GSH*TwoGroups*Protein  0 0 0 0 0 0 0 0 0 0 0 0 0 0 0 0 0 0 0 0 0 0 0 0 0 0 0 0 0 1 0 0 0 0 0 0 0 0 0
0 0 0 0 0 0 0 0 0 0 0 0 0 0 0 0 0 0 0 0 0 0 0 0 0 0 0 0 0 0 0 0 0 0 0 0 0 0 0 0 0 0 0 0 0 0 0 0 0 0 0 0 0 0 0 0 0 0 0 0 0 0 0 0 0
0 0 0 0 0 0 0 0 0 0 0 0 0 0 0 0 0 0 0 0 0 0 0 0 0 0 0 0 0 0 0 0 0 0 0 0 0 0 0 0 0 0 0 0 0 0 0 0 0 0 0 0 0 0 0 0 0 0 0 0 0 0 0 0 0
0 0 0 0 0 0 0 0 0 0 0 0 0 0 0;
Estimate "Epo Old Slope" Log_Total_GSH 1 Log_Total_GSH*TwoGroups 1 0 Log_Total_GSH*Protein 0 0 0 0 0 0 0 0 0 0 0 0 0 0 0 0 0 0 0
0 0 0 0 0 0 0 0 0 0 0 1 0 0 0 0 0 0 0 0 0 0 0 0 0 0 0 0 0 0 0 0 0 0 0 0 0 0 0 0 0 0 0 0 0 0 0 0 0 0 0 0 0 0 0 0 0 0 0 0 0 0 0 0 0
0 0 0 0 0 0 0 0 Log_Total_GSH*TwoGroups*Protein  0 0 0 0 0 0 0 0 0 0 0 0 0 0 0 0 0 0 0 0 0 0 0 0 0 0 0 0 0 0 1 0 0 0 0 0 0 0 0 0
0 0 0 0 0 0 0 0 0 0 0 0 0 0 0 0 0 0 0 0 0 0 0 0 0 0 0 0 0 0 0 0 0 0 0 0 0 0 0 0 0 0 0 0 0 0 0 0 0 0 0 0 0 0 0 0 0 0 0 0 0 0 0 0 0
0 0 0 0 0 0 0 0 0 0 0 0 0 0 0 0 0 0 0 0 0 0 0 0 0 0 0 0 0 0 0 0 0 0 0 0 0 0 0 0 0 0 0 0 0 0 0 0 0 0 0 0 0 0 0 0 0 0 0 0 0 0 0 0 0
0 0 0 0 0 0 0 0 0 0 0 0 0 0;
Estimate "Erbb4 Old Slope" Log_Total_GSH 1 Log_Total_GSH*TwoGroups 1 0 Log_Total_GSH*Protein 0 0 0 0 0 0 0 0 0 0 0 0 0 0 0 0 0 0
0 0 0 0 0 0 0 0 0 0 0 0 0 1 0 0 0 0 0 0 0 0 0 0 0 0 0 0 0 0 0 0 0 0 0 0 0 0 0 0 0 0 0 0 0 0 0 0 0 0 0 0 0 0 0 0 0 0 0 0 0 0 0 0 0
0 0 0 0 0 0 0 0 0 Log_Total_GSH*TwoGroups*Protein  0 0 0 0 0 0 0 0 0 0 0 0 0 0 0 0 0 0 0 0 0 0 0 0 0 0 0 0 0 0 0 1 0 0 0 0 0 0 0
```

```
0 0 0 0 0 0 0 0 0 0 0 0 0 0 0 0 0 0 0 0 0 0 0 0 0 0 0 0 0 0 0 0 0 0 0 0 0 0 0 0 0 0 0 0 0 0 0 0 0 0 0 0 0 0 0 0 0 0 0 0 0 0 0 0 0
0 0 0 0 0 0 0 0 0 0 0 0 0 0 0 0 0 0 0 0 0 0 0 0 0 0 0 0 0 0 0 0 0 0 0 0 0 0 0 0 0 0 0 0 0 0 0 0 0 0 0 0 0 0 0 0 0 0 0 0 0 0 0 0 0
0 0 0 0 0 0 0 0 0 0 0 0 0 0 0;
Estimate "Fas Old Slope" Log_Total_GSH 1 Log_Total_GSH*TwoGroups 1 0 Log_Total_GSH*Protein 0 0 0 0 0 0 0 0 0 0 0 0 0 0 0 0 0 0 0
0 0 0 0 0 0 0 0 0 0 0 0 0 1 0 0 0 0 0 0 0 0 0 0 0 0 0 0 0 0 0 0 0 0 0 0 0 0 0 0 0 0 0 0 0 0 0 0 0 0 0 0 0 0 0 0 0 0 0 0 0 0 0 0 0
0 0 0 0 0 0 0 0 Log_Total_GSH*TwoGroups*Protein  0 0 0 0 0 0 0 0 0 0 0 0 0 0 0 0 0 0 0 0 0 0 0 0 0 0 0 0 0 0 0 0 1 0 0 0 0 0 0 0
0 0 0 0 0 0 0 0 0 0 0 0 0 0 0 0 0 0 0 0 0 0 0 0 0 0 0 0 0 0 0 0 0 0 0 0 0 0 0 0 0 0 0 0 0 0 0 0 0 0 0 0 0 0 0 0 0 0 0 0 0 0 0 0 0
0 0 0 0 0 0 0 0 0 0 0 0 0 0 0 0 0 0 0 0 0 0 0 0 0 0 0 0 0 0 0 0 0 0 0 0 0 0 0 0 0 0 0 0 0 0 0 0 0 0 0 0 0 0 0 0 0 0 0 0 0 0 0 0 0
0 0 0 0 0 0 0 0 0 0 0 0 0 0;
Estimate "Fli1 Old Slope" Log_Total_GSH 1 Log_Total_GSH*TwoGroups 1 0 Log_Total_GSH*Protein 0 0 0 0 0 0 0 0 0 0 0 0 0 0 0 0 0 0 0
0 0 0 0 0 0 0 0 0 0 0 0 0 0 1 0 0 0 0 0 0 0 0 0 0 0 0 0 0 0 0 0 0 0 0 0 0 0 0 0 0 0 0 0 0 0 0 0 0 0 0 0 0 0 0 0 0 0 0 0 0 0 0 0 0
0 0 0 0 0 0 0 0 Log_Total_GSH*TwoGroups*Protein  0 0 0 0 0 0 0 0 0 0 0 0 0 0 0 0 0 0 0 0 0 0 0 0 0 0 0 0 0 0 0 0 0 1 0 0 0 0 0 0
0 0 0 0 0 0 0 0 0 0 0 0 0 0 0 0 0 0 0 0 0 0 0 0 0 0 0 0 0 0 0 0 0 0 0 0 0 0 0 0 0 0 0 0 0 0 0 0 0 0 0 0 0 0 0 0 0 0 0 0 0 0 0 0 0
0 0 0 0 0 0 0 0 0 0 0 0 0 0 0 0 0 0 0 0 0 0 0 0 0 0 0 0 0 0 0 0 0 0 0 0 0 0 0 0 0 0 0 0 0 0 0 0 0 0 0 0 0 0 0 0 0 0 0 0 0 0 0 0 0
0 0 0 0 0 0 0 0 0 0 0 0 0 0;
Estimate "Flrt2 Old Slope" Log_Total_GSH 1 Log_Total_GSH*TwoGroups 1 0 Log_Total_GSH*Protein 0 0 0 0 0 0 0 0 0 0 0 0 0 0 0 0 0 0
0 0 0 0 0 0 0 0 0 0 0 0 0 0 0 0 1 0 0 0 0 0 0 0 0 0 0 0 0 0 0 0 0 0 0 0 0 0 0 0 0 0 0 0 0 0 0 0 0 0 0 0 0 0 0 0 0 0 0 0 0 0 0 0 0
0 0 0 0 0 0 0 0 0 Log_Total_GSH*TwoGroups*Protein  0 0 0 0 0 0 0 0 0 0 0 0 0 0 0 0 0 0 0 0 0 0 0 0 0 0 0 0 0 0 0 0 0 0 1 0 0 0 0
0 0 0 0 0 0 0 0 0 0 0 0 0 0 0 0 0 0 0 0 0 0 0 0 0 0 0 0 0 0 0 0 0 0 0 0 0 0 0 0 0 0 0 0 0 0 0 0 0 0 0 0 0 0 0 0 0 0 0 0 0 0 0 0 0
0 0 0 0 0 0 0 0 0 0 0 0 0 0 0 0 0 0 0 0 0 0 0 0 0 0 0 0 0 0 0 0 0 0 0 0 0 0 0 0 0 0 0 0 0 0 0 0 0 0 0 0 0 0 0 0 0 0 0 0 0 0 0 0 0
0 0 0 0 0 0 0 0 0 0 0 0 0 0 0;
Estimate "Foxo1 Old Slope" Log_Total_GSH 1 Log_Total_GSH*TwoGroups 1 0 Log_Total_GSH*Protein 0 0 0 0 0 0 0 0 0 0 0 0 0 0 0 0 0 0
0 0 0 0 0 0 0 0 0 0 0 0 0 0 0 0 0 1 0 0 0 0 0 0 0 0 0 0 0 0 0 0 0 0 0 0 0 0 0 0 0 0 0 0 0 0 0 0 0 0 0 0 0 0 0 0 0 0 0 0 0 0 0 0 0
0 0 0 0 0 0 0 0 0 Log_Total_GSH*TwoGroups*Protein  0 0 0 0 0 0 0 0 0 0 0 0 0 0 0 0 0 0 0 0 0 0 0 0 0 0 0 0 0 0 0 0 0 0 0 1 0 0 0
0 0 0 0 0 0 0 0 0 0 0 0 0 0 0 0 0 0 0 0 0 0 0 0 0 0 0 0 0 0 0 0 0 0 0 0 0 0 0 0 0 0 0 0 0 0 0 0 0 0 0 0 0 0 0 0 0 0 0 0 0 0 0 0 0
0 0 0 0 0 0 0 0 0 0 0 0 0 0 0 0 0 0 0 0 0 0 0 0 0 0 0 0 0 0 0 0 0 0 0 0 0 0 0 0 0 0 0 0 0 0 0 0 0 0 0 0 0 0 0 0 0 0 0 0 0 0 0 0 0
0 0 0 0 0 0 0 0 0 0 0 0 0 0 0;
Estimate "Fst Old Slope" Log_Total_GSH 1 Log_Total_GSH*TwoGroups 1 0 Log_Total_GSH*Protein 0 0 0 0 0 0 0 0 0 0 0 0 0 0 0 0 0 0 0
0 0 0 0 0 0 0 0 0 0 0 0 0 0 0 0 0 1 0 0 0 0 0 0 0 0 0 0 0 0 0 0 0 0 0 0 0 0 0 0 0 0 0 0 0 0 0 0 0 0 0 0 0 0 0 0 0 0 0 0 0 0 0 0 0
0 0 0 0 0 0 0 0 Log_Total_GSH*TwoGroups*Protein  0 0 0 0 0 0 0 0 0 0 0 0 0 0 0 0 0 0 0 0 0 0 0 0 0 0 0 0 0 0 0 0 0 0 0 0 1 0 0 0
0 0 0 0 0 0 0 0 0 0 0 0 0 0 0 0 0 0 0 0 0 0 0 0 0 0 0 0 0 0 0 0 0 0 0 0 0 0 0 0 0 0 0 0 0 0 0 0 0 0 0 0 0 0 0 0 0 0 0 0 0 0 0 0 0
0 0 0 0 0 0 0 0 0 0 0 0 0 0 0 0 0 0 0 0 0 0 0 0 0 0 0 0 0 0 0 0 0 0 0 0 0 0 0 0 0 0 0 0 0 0 0 0 0 0 0 0 0 0 0 0 0 0 0 0 0 0 0 0 0
0 0 0 0 0 0 0 0 0 0 0 0 0 0;
Estimate "Fstl3 Old Slope" Log_Total_GSH 1 Log_Total_GSH*TwoGroups 1 0 Log_Total_GSH*Protein 0 0 0 0 0 0 0 0 0 0 0 0 0 0 0 0 0 0
0 0 0 0 0 0 0 0 0 0 0 0 0 0 0 0 0 0 0 1 0 0 0 0 0 0 0 0 0 0 0 0 0 0 0 0 0 0 0 0 0 0 0 0 0 0 0 0 0 0 0 0 0 0 0 0 0 0 0 0 0 0 0 0 0
0 0 0 0 0 0 0 0 0 Log_Total_GSH*TwoGroups*Protein  0 0 0 0 0 0 0 0 0 0 0 0 0 0 0 0 0 0 0 0 0 0 0 0 0 0 0 0 0 0 0 0 0 0 0 0 0 1 0
0 0 0 0 0 0 0 0 0 0 0 0 0 0 0 0 0 0 0 0 0 0 0 0 0 0 0 0 0 0 0 0 0 0 0 0 0 0 0 0 0 0 0 0 0 0 0 0 0 0 0 0 0 0 0 0 0 0 0 0 0 0 0 0 0
0 0 0 0 0 0 0 0 0 0 0 0 0 0 0 0 0 0 0 0 0 0 0 0 0 0 0 0 0 0 0 0 0 0 0 0 0 0 0 0 0 0 0 0 0 0 0 0 0 0 0 0 0 0 0 0 0 0 0 0 0 0 0 0 0
0 0 0 0 0 0 0 0 0 0 0 0 0 0 0;
Estimate "Gcg Old Slope" Log_Total_GSH 1 Log_Total_GSH*TwoGroups 1 0 Log_Total_GSH*Protein 0 0 0 0 0 0 0 0 0 0 0 0 0 0 0 0 0 0 0
0 0 0 0 0 0 0 0 0 0 0 0 0 0 0 0 0 0 0 1 0 0 0 0 0 0 0 0 0 0 0 0 0 0 0 0 0 0 0 0 0 0 0 0 0 0 0 0 0 0 0 0 0 0 0 0 0 0 0 0 0 0 0 0 0
0 0 0 0 0 0 0 0 Log_Total_GSH*TwoGroups*Protein  0 0 0 0 0 0 0 0 0 0 0 0 0 0 0 0 0 0 0 0 0 0 0 0 0 0 0 0 0 0 0 0 0 0 0 0 0 0 1 0
0 0 0 0 0 0 0 0 0 0 0 0 0 0 0 0 0 0 0 0 0 0 0 0 0 0 0 0 0 0 0 0 0 0 0 0 0 0 0 0 0 0 0 0 0 0 0 0 0 0 0 0 0 0 0 0 0 0 0 0 0 0 0 0 0
0 0 0 0 0 0 0 0 0 0 0 0 0 0 0 0 0 0 0 0 0 0 0 0 0 0 0 0 0 0 0 0 0 0 0 0 0 0 0 0 0 0 0 0 0 0 0 0 0 0 0 0 0 0 0 0 0 0 0 0 0 0 0 0 0
0 0 0 0 0 0 0 0 0 0 0 0 0 0;
Estimate "Gdnf Old Slope" Log_Total_GSH 1 Log_Total_GSH*TwoGroups 1 0 Log_Total_GSH*Protein 0 0 0 0 0 0 0 0 0 0 0 0 0 0 0 0 0 0 0
0 0 0 0 0 0 0 0 0 0 0 0 0 0 0 0 0 0 0 0 1 0 0 0 0 0 0 0 0 0 0 0 0 0 0 0 0 0 0 0 0 0 0 0 0 0 0 0 0 0 0 0 0 0 0 0 0 0 0 0 0 0 0 0 0
0 0 0 0 0 0 0 0 Log_Total_GSH*TwoGroups*Protein  0 0 0 0 0 0 0 0 0 0 0 0 0 0 0 0 0 0 0 0 0 0 0 0 0 0 0 0 0 0 0 0 0 0 0 0 0 0 0 1
0 0 0 0 0 0 0 0 0 0 0 0 0 0 0 0 0 0 0 0 0 0 0 0 0 0 0 0 0 0 0 0 0 0 0 0 0 0 0 0 0 0 0 0 0 0 0 0 0 0 0 0 0 0 0 0 0 0 0 0 0 0 0 0 0
0 0 0 0 0 0 0 0 0 0 0 0 0 0 0 0 0 0 0 0 0 0 0 0 0 0 0 0 0 0 0 0 0 0 0 0 0 0 0 0 0 0 0 0 0 0 0 0 0 0 0 0 0 0 0 0 0 0 0 0 0 0 0 0 0
0 0 0 0 0 0 0 0 0 0 0 0 0 0;
Estimate "Gfra1 Old Slope" Log_Total_GSH 1 Log_Total_GSH*TwoGroups 1 0 Log_Total_GSH*Protein 0 0 0 0 0 0 0 0 0 0 0 0 0 0 0 0 0 0
0 0 0 0 0 0 0 0 0 0 0 0 0 0 0 0 0 0 0 0 0 0 1 0 0 0 0 0 0 0 0 0 0 0 0 0 0 0 0 0 0 0 0 0 0 0 0 0 0 0 0 0 0 0 0 0 0 0 0 0 0 0 0 0 0
0 0 0 0 0 0 0 0 0 Log_Total_GSH*TwoGroups*Protein  0 0 0 0 0 0 0 0 0 0 0 0 0 0 0 0 0 0 0 0 0 0 0 0 0 0 0 0 0 0 0 0 0 0 0 0 0 0 0
0 1 0 0 0 0 0 0 0 0 0 0 0 0 0 0 0 0 0 0 0 0 0 0 0 0 0 0 0 0 0 0 0 0 0 0 0 0 0 0 0 0 0 0 0 0 0 0 0 0 0 0 0 0 0 0 0 0 0 0 0 0 0 0 0
0 0 0 0 0 0 0 0 0 0 0 0 0 0 0 0 0 0 0 0 0 0 0 0 0 0 0 0 0 0 0 0 0 0 0 0 0 0 0 0 0 0 0 0 0 0 0 0 0 0 0 0 0 0 0 0 0 0 0 0 0 0 0 0 0
0 0 0 0 0 0 0 0 0 0 0 0 0 0 0;
Estimate "Ghrl Old Slope" Log_Total_GSH 1 Log_Total_GSH*TwoGroups 1 0 Log_Total_GSH*Protein 0 0 0 0 0 0 0 0 0 0 0 0 0 0 0 0 0 0 0
0 0 0 0 0 0 0 0 0 0 0 0 0 0 0 0 0 0 0 0 0 0 1 0 0 0 0 0 0 0 0 0 0 0 0 0 0 0 0 0 0 0 0 0 0 0 0 0 0 0 0 0 0 0 0 0 0 0 0 0 0 0 0 0 0
0 0 0 0 0 0 0 0 Log_Total_GSH*TwoGroups*Protein  0 0 0 0 0 0 0 0 0 0 0 0 0 0 0 0 0 0 0 0 0 0 0 0 0 0 0 0 0 0 0 0 0 0 0 0 0 0 0 0
0 1 0 0 0 0 0 0 0 0 0 0 0 0 0 0 0 0 0 0 0 0 0 0 0 0 0 0 0 0 0 0 0 0 0 0 0 0 0 0 0 0 0 0 0 0 0 0 0 0 0 0 0 0 0 0 0 0 0 0 0 0 0 0 0
0 0 0 0 0 0 0 0 0 0 0 0 0 0 0 0 0 0 0 0 0 0 0 0 0 0 0 0 0 0 0 0 0 0 0 0 0 0 0 0 0 0 0 0 0 0 0 0 0 0 0 0 0 0 0 0 0 0 0 0 0 0 0 0 0
0 0 0 0 0 0 0 0 0 0 0 0 0 0;
Estimate "Hgf Old Slope" Log_Total_GSH 1 Log_Total_GSH*TwoGroups 1 0 Log_Total_GSH*Protein 0 0 0 0 0 0 0 0 0 0 0 0 0 0 0 0 0 0 0
0 0 0 0 0 0 0 0 0 0 0 0 0 0 0 0 0 0 0 0 0 0 0 1 0 0 0 0 0 0 0 0 0 0 0 0 0 0 0 0 0 0 0 0 0 0 0 0 0 0 0 0 0 0 0 0 0 0 0 0 0 0 0 0 0
0 0 0 0 0 0 0 0 Log_Total_GSH*TwoGroups*Protein  0 0 0 0 0 0 0 0 0 0 0 0 0 0 0 0 0 0 0 0 0 0 0 0 0 0 0 0 0 0 0 0 0 0 0 0 0 0 0 0
0 0 1 0 0 0 0 0 0 0 0 0 0 0 0 0 0 0 0 0 0 0 0 0 0 0 0 0 0 0 0 0 0 0 0 0 0 0 0 0 0 0 0 0 0 0 0 0 0 0 0 0 0 0 0 0 0 0 0 0 0 0 0 0 0
0 0 0 0 0 0 0 0 0 0 0 0 0 0 0 0 0 0 0 0 0 0 0 0 0 0 0 0 0 0 0 0 0 0 0 0 0 0 0 0 0 0 0 0 0 0 0 0 0 0 0 0 0 0 0 0 0 0 0 0 0 0 0 0 0
0 0 0 0 0 0 0 0 0 0 0 0 0 0;
Estimate "Igsf3 Old Slope" Log_Total_GSH 1 Log_Total_GSH*TwoGroups 1 0 Log_Total_GSH*Protein 0 0 0 0 0 0 0 0 0 0 0 0 0 0 0 0 0 0
0 0 0 0 0 0 0 0 0 0 0 0 0 0 0 0 0 0 0 0 0 0 0 0 0 1 0 0 0 0 0 0 0 0 0 0 0 0 0 0 0 0 0 0 0 0 0 0 0 0 0 0 0 0 0 0 0 0 0 0 0 0 0 0 0
0 0 0 0 0 0 0 0 0 Log_Total_GSH*TwoGroups*Protein  0 0 0 0 0 0 0 0 0 0 0 0 0 0 0 0 0 0 0 0 0 0 0 0 0 0 0 0 0 0 0 0 0 0 0 0 0 0 0
0 0 0 0 1 0 0 0 0 0 0 0 0 0 0 0 0 0 0 0 0 0 0 0 0 0 0 0 0 0 0 0 0 0 0 0 0 0 0 0 0 0 0 0 0 0 0 0 0 0 0 0 0 0 0 0 0 0 0 0 0 0 0 0 0
0 0 0 0 0 0 0 0 0 0 0 0 0 0 0 0 0 0 0 0 0 0 0 0 0 0 0 0 0 0 0 0 0 0 0 0 0 0 0 0 0 0 0 0 0 0 0 0 0 0 0 0 0 0 0 0 0 0 0 0 0 0 0 0 0
0 0 0 0 0 0 0 0 0 0 0 0 0 0 0;
Estimate "Il10 Old Slope" Log_Total_GSH 1 Log_Total_GSH*TwoGroups 1 0 Log_Total_GSH*Protein 0 0 0 0 0 0 0 0 0 0 0 0 0 0 0 0 0 0 0
0 0 0 0 0 0 0 0 0 0 0 0 0 0 0 0 0 0 0 0 0 0 0 0 0 1 0 0 0 0 0 0 0 0 0 0 0 0 0 0 0 0 0 0 0 0 0 0 0 0 0 0 0 0 0 0 0 0 0 0 0 0 0 0 0
0 0 0 0 0 0 0 0 Log_Total_GSH*TwoGroups*Protein  0 0 0 0 0 0 0 0 0 0 0 0 0 0 0 0 0 0 0 0 0 0 0 0 0 0 0 0 0 0 0 0 0 0 0 0 0 0 0 0
0 0 0 0 1 0 0 0 0 0 0 0 0 0 0 0 0 0 0 0 0 0 0 0 0 0 0 0 0 0 0 0 0 0 0 0 0 0 0 0 0 0 0 0 0 0 0 0 0 0 0 0 0 0 0 0 0 0 0 0 0 0 0 0 0
0 0 0 0 0 0 0 0 0 0 0 0 0 0 0 0 0 0 0 0 0 0 0 0 0 0 0 0 0 0 0 0 0 0 0 0 0 0 0 0 0 0 0 0 0 0 0 0 0 0 0 0 0 0 0 0 0 0 0 0 0 0 0 0 0
0 0 0 0 0 0 0 0 0 0 0 0 0 0;
Estimate "Il17a Old Slope" Log_Total_GSH 1 Log_Total_GSH*TwoGroups 1 0 Log_Total_GSH*Protein 0 0 0 0 0 0 0 0 0 0 0 0 0 0 0 0 0 0
0 0 0 0 0 0 0 0 0 0 0 0 0 0 0 0 0 0 0 0 0 0 0 0 0 0 0 1 0 0 0 0 0 0 0 0 0 0 0 0 0 0 0 0 0 0 0 0 0 0 0 0 0 0 0 0 0 0 0 0 0 0 0 0 0
0 0 0 0 0 0 0 0 0 Log_Total_GSH*TwoGroups*Protein  0 0 0 0 0 0 0 0 0 0 0 0 0 0 0 0 0 0 0 0 0 0 0 0 0 0 0 0 0 0 0 0 0 0 0 0 0 0 0
0 0 0 0 0 0 1 0 0 0 0 0 0 0 0 0 0 0 0 0 0 0 0 0 0 0 0 0 0 0 0 0 0 0 0 0 0 0 0 0 0 0 0 0 0 0 0 0 0 0 0 0 0 0 0 0 0 0 0 0 0 0 0 0 0
0 0 0 0 0 0 0 0 0 0 0 0 0 0 0 0 0 0 0 0 0 0 0 0 0 0 0 0 0 0 0 0 0 0 0 0 0 0 0 0 0 0 0 0 0 0 0 0 0 0 0 0 0 0 0 0 0 0 0 0 0 0 0 0 0
0 0 0 0 0 0 0 0 0 0 0 0 0 0 0;
Estimate "Il17f Old Slope" Log_Total_GSH 1 Log_Total_GSH*TwoGroups 1 0 Log_Total_GSH*Protein 0 0 0 0 0 0 0 0 0 0 0 0 0 0 0 0 0 0
0 0 0 0 0 0 0 0 0 0 0 0 0 0 0 0 0 0 0 0 0 0 0 0 0 0 0 0 1 0 0 0 0 0 0 0 0 0 0 0 0 0 0 0 0 0 0 0 0 0 0 0 0 0 0 0 0 0 0 0 0 0 0 0 0
0 0 0 0 0 0 0 0 0 Log_Total_GSH*TwoGroups*Protein  0 0 0 0 0 0 0 0 0 0 0 0 0 0 0 0 0 0 0 0 0 0 0 0 0 0 0 0 0 0 0 0 0 0 0 0 0 0 0
0 0 0 0 0 0 0 1 0 0 0 0 0 0 0 0 0 0 0 0 0 0 0 0 0 0 0 0 0 0 0 0 0 0 0 0 0 0 0 0 0 0 0 0 0 0 0 0 0 0 0 0 0 0 0 0 0 0 0 0 0 0 0 0 0
0 0 0 0 0 0 0 0 0 0 0 0 0 0 0 0 0 0 0 0 0 0 0 0 0 0 0 0 0 0 0 0 0 0 0 0 0 0 0 0 0 0 0 0 0 0 0 0 0 0 0 0 0 0 0 0 0 0 0 0 0 0 0 0 0
0 0 0 0 0 0 0 0 0 0 0 0 0 0 0;
Estimate "Il1a Old Slope" Log_Total_GSH 1 Log_Total_GSH*TwoGroups 1 0 Log_Total_GSH*Protein 0 0 0 0 0 0 0 0 0 0 0 0 0 0 0 0 0 0 0
0 0 0 0 0 0 0 0 0 0 0 0 0 0 0 0 0 0 0 0 0 0 0 0 0 0 0 0 1 0 0 0 0 0 0 0 0 0 0 0 0 0 0 0 0 0 0 0 0 0 0 0 0 0 0 0 0 0 0 0 0 0 0 0 0
```

```
0 0 0 0 0 0 0 0 Log_Total_GSH*TwoGroups*Protein  0 0 0 0 0 0 0 0 0 0 0 0 0 0 0 0 0 0 0 0 0 0 0 0 0 0 0 0 0 0 0 0 0 0 0 0 0 0 0 0
0 0 0 0 0 0 0 1 0 0 0 0 0 0 0 0 0 0 0 0 0 0 0 0 0 0 0 0 0 0 0 0 0 0 0 0 0 0 0 0 0 0 0 0 0 0 0 0 0 0 0 0 0 0 0 0 0 0 0 0 0 0 0 0 0
0 0 0 0 0 0 0 0 0 0 0 0 0 0 0 0 0 0 0 0 0 0 0 0 0 0 0 0 0 0 0 0 0 0 0 0 0 0 0 0 0 0 0 0 0 0 0 0 0 0 0 0 0 0 0 0 0 0 0 0 0 0 0 0 0
0 0 0 0 0 0 0 0 0 0 0 0 0 0;
Estimate "Il1b Old Slope" Log_Total_GSH 1 Log_Total_GSH*TwoGroups 1 0 Log_Total_GSH*Protein 0 0 0 0 0 0 0 0 0 0 0 0 0 0 0 0 0 0 0
0 0 0 0 0 0 0 0 0 0 0 0 0 0 0 0 0 0 0 0 0 0 0 0 0 0 0 0 0 1 0 0 0 0 0 0 0 0 0 0 0 0 0 0 0 0 0 0 0 0 0 0 0 0 0 0 0 0 0 0 0 0 0 0 0
0 0 0 0 0 0 0 0 Log_Total_GSH*TwoGroups*Protein  0 0 0 0 0 0 0 0 0 0 0 0 0 0 0 0 0 0 0 0 0 0 0 0 0 0 0 0 0 0 0 0 0 0 0 0 0 0 0 0
0 0 0 0 0 0 0 0 1 0 0 0 0 0 0 0 0 0 0 0 0 0 0 0 0 0 0 0 0 0 0 0 0 0 0 0 0 0 0 0 0 0 0 0 0 0 0 0 0 0 0 0 0 0 0 0 0 0 0 0 0 0 0 0 0
0 0 0 0 0 0 0 0 0 0 0 0 0 0 0 0 0 0 0 0 0 0 0 0 0 0 0 0 0 0 0 0 0 0 0 0 0 0 0 0 0 0 0 0 0 0 0 0 0 0 0 0 0 0 0 0 0 0 0 0 0 0 0 0 0
0 0 0 0 0 0 0 0 0 0 0 0 0 0;
Estimate "Il23r Old Slope" Log_Total_GSH 1 Log_Total_GSH*TwoGroups 1 0 Log_Total_GSH*Protein 0 0 0 0 0 0 0 0 0 0 0 0 0 0 0 0 0 0
0 0 0 0 0 0 0 0 0 0 0 0 0 0 0 0 0 0 0 0 0 0 0 0 0 0 0 0 0 0 0 1 0 0 0 0 0 0 0 0 0 0 0 0 0 0 0 0 0 0 0 0 0 0 0 0 0 0 0 0 0 0 0 0 0
0 0 0 0 0 0 0 0 0 Log_Total_GSH*TwoGroups*Protein  0 0 0 0 0 0 0 0 0 0 0 0 0 0 0 0 0 0 0 0 0 0 0 0 0 0 0 0 0 0 0 0 0 0 0 0 0 0 0
0 0 0 0 0 0 0 0 0 0 1 0 0 0 0 0 0 0 0 0 0 0 0 0 0 0 0 0 0 0 0 0 0 0 0 0 0 0 0 0 0 0 0 0 0 0 0 0 0 0 0 0 0 0 0 0 0 0 0 0 0 0 0 0 0
0 0 0 0 0 0 0 0 0 0 0 0 0 0 0 0 0 0 0 0 0 0 0 0 0 0 0 0 0 0 0 0 0 0 0 0 0 0 0 0 0 0 0 0 0 0 0 0 0 0 0 0 0 0 0 0 0 0 0 0 0 0 0 0 0
0 0 0 0 0 0 0 0 0 0 0 0 0 0 0;
Estimate "Il5 Old Slope" Log_Total_GSH 1 Log_Total_GSH*TwoGroups 1 0 Log_Total_GSH*Protein 0 0 0 0 0 0 0 0 0 0 0 0 0 0 0 0 0 0 0
0 0 0 0 0 0 0 0 0 0 0 0 0 0 0 0 0 0 0 0 0 0 0 0 0 0 0 0 0 0 0 1 0 0 0 0 0 0 0 0 0 0 0 0 0 0 0 0 0 0 0 0 0 0 0 0 0 0 0 0 0 0 0 0 0
0 0 0 0 0 0 0 0 Log_Total_GSH*TwoGroups*Protein  0 0 0 0 0 0 0 0 0 0 0 0 0 0 0 0 0 0 0 0 0 0 0 0 0 0 0 0 0 0 0 0 0 0 0 0 0 0 0 0
0 0 0 0 0 0 0 0 0 0 1 0 0 0 0 0 0 0 0 0 0 0 0 0 0 0 0 0 0 0 0 0 0 0 0 0 0 0 0 0 0 0 0 0 0 0 0 0 0 0 0 0 0 0 0 0 0 0 0 0 0 0 0 0 0
0 0 0 0 0 0 0 0 0 0 0 0 0 0 0 0 0 0 0 0 0 0 0 0 0 0 0 0 0 0 0 0 0 0 0 0 0 0 0 0 0 0 0 0 0 0 0 0 0 0 0 0 0 0 0 0 0 0 0 0 0 0 0 0 0
0 0 0 0 0 0 0 0 0 0 0 0 0 0;
Estimate "Il6 Old Slope" Log_Total_GSH 1 Log_Total_GSH*TwoGroups 1 0 Log_Total_GSH*Protein 0 0 0 0 0 0 0 0 0 0 0 0 0 0 0 0 0 0 0
0 0 0 0 0 0 0 0 0 0 0 0 0 0 0 0 0 0 0 0 0 0 0 0 0 0 0 0 0 0 0 0 1 0 0 0 0 0 0 0 0 0 0 0 0 0 0 0 0 0 0 0 0 0 0 0 0 0 0 0 0 0 0 0 0
0 0 0 0 0 0 0 0 Log_Total_GSH*TwoGroups*Protein  0 0 0 0 0 0 0 0 0 0 0 0 0 0 0 0 0 0 0 0 0 0 0 0 0 0 0 0 0 0 0 0 0 0 0 0 0 0 0 0
0 0 0 0 0 0 0 0 0 0 0 1 0 0 0 0 0 0 0 0 0 0 0 0 0 0 0 0 0 0 0 0 0 0 0 0 0 0 0 0 0 0 0 0 0 0 0 0 0 0 0 0 0 0 0 0 0 0 0 0 0 0 0 0 0
0 0 0 0 0 0 0 0 0 0 0 0 0 0 0 0 0 0 0 0 0 0 0 0 0 0 0 0 0 0 0 0 0 0 0 0 0 0 0 0 0 0 0 0 0 0 0 0 0 0 0 0 0 0 0 0 0 0 0 0 0 0 0 0 0
0 0 0 0 0 0 0 0 0 0 0 0 0 0;
Estimate "Itgb1bp2 Old Slope" Log_Total_GSH 1 Log_Total_GSH*TwoGroups 1 0 Log_Total_GSH*Protein 0 0 0 0 0 0 0 0 0 0 0 0 0 0 0 0 0
0 0 0 0 0 0 0 0 0 0 0 0 0 0 0 0 0 0 0 0 0 0 0 0 0 0 0 0 0 0 0 0 0 0 0 1 0 0 0 0 0 0 0 0 0 0 0 0 0 0 0 0 0 0 0 0 0 0 0 0 0 0 0 0 0
0 0 0 0 0 0 0 0 0 0 Log_Total_GSH*TwoGroups*Protein  0 0 0 0 0 0 0 0 0 0 0 0 0 0 0 0 0 0 0 0 0 0 0 0 0 0 0 0 0 0 0 0 0 0 0 0 0 0
0 0 0 0 0 0 0 0 0 0 0 0 0 0 1 0 0 0 0 0 0 0 0 0 0 0 0 0 0 0 0 0 0 0 0 0 0 0 0 0 0 0 0 0 0 0 0 0 0 0 0 0 0 0 0 0 0 0 0 0 0 0 0 0 0
0 0 0 0 0 0 0 0 0 0 0 0 0 0 0 0 0 0 0 0 0 0 0 0 0 0 0 0 0 0 0 0 0 0 0 0 0 0 0 0 0 0 0 0 0 0 0 0 0 0 0 0 0 0 0 0 0 0 0 0 0 0 0 0 0
0 0 0 0 0 0 0 0 0 0 0 0 0 0 0 0;
Estimate "Itgb6 Old Slope" Log_Total_GSH 1 Log_Total_GSH*TwoGroups 1 0 Log_Total_GSH*Protein 0 0 0 0 0 0 0 0 0 0 0 0 0 0 0 0 0 0
0 0 0 0 0 0 0 0 0 0 0 0 0 0 0 0 0 0 0 0 0 0 0 0 0 0 0 0 0 0 0 0 0 0 0 1 0 0 0 0 0 0 0 0 0 0 0 0 0 0 0 0 0 0 0 0 0 0 0 0 0 0 0 0 0
0 0 0 0 0 0 0 0 0 Log_Total_GSH*TwoGroups*Protein  0 0 0 0 0 0 0 0 0 0 0 0 0 0 0 0 0 0 0 0 0 0 0 0 0 0 0 0 0 0 0 0 0 0 0 0 0 0 0
0 0 0 0 0 0 0 0 0 0 0 0 0 0 1 0 0 0 0 0 0 0 0 0 0 0 0 0 0 0 0 0 0 0 0 0 0 0 0 0 0 0 0 0 0 0 0 0 0 0 0 0 0 0 0 0 0 0 0 0 0 0 0 0 0
0 0 0 0 0 0 0 0 0 0 0 0 0 0 0 0 0 0 0 0 0 0 0 0 0 0 0 0 0 0 0 0 0 0 0 0 0 0 0 0 0 0 0 0 0 0 0 0 0 0 0 0 0 0 0 0 0 0 0 0 0 0 0 0 0
0 0 0 0 0 0 0 0 0 0 0 0 0 0 0;
Estimate "Kitlg Old Slope" Log_Total_GSH 1 Log_Total_GSH*TwoGroups 1 0 Log_Total_GSH*Protein 0 0 0 0 0 0 0 0 0 0 0 0 0 0 0 0 0 0
0 0 0 0 0 0 0 0 0 0 0 0 0 0 0 0 0 0 0 0 0 0 0 0 0 0 0 0 0 0 0 0 0 0 0 0 1 0 0 0 0 0 0 0 0 0 0 0 0 0 0 0 0 0 0 0 0 0 0 0 0 0 0 0 0
0 0 0 0 0 0 0 0 0 Log_Total_GSH*TwoGroups*Protein  0 0 0 0 0 0 0 0 0 0 0 0 0 0 0 0 0 0 0 0 0 0 0 0 0 0 0 0 0 0 0 0 0 0 0 0 0 0 0
0 0 0 0 0 0 0 0 0 0 0 0 0 0 0 1 0 0 0 0 0 0 0 0 0 0 0 0 0 0 0 0 0 0 0 0 0 0 0 0 0 0 0 0 0 0 0 0 0 0 0 0 0 0 0 0 0 0 0 0 0 0 0 0 0
0 0 0 0 0 0 0 0 0 0 0 0 0 0 0 0 0 0 0 0 0 0 0 0 0 0 0 0 0 0 0 0 0 0 0 0 0 0 0 0 0 0 0 0 0 0 0 0 0 0 0 0 0 0 0 0 0 0 0 0 0 0 0 0 0
0 0 0 0 0 0 0 0 0 0 0 0 0 0 0;
Estimate "Lgmn Old Slope" Log_Total_GSH 1 Log_Total_GSH*TwoGroups 1 0 Log_Total_GSH*Protein 0 0 0 0 0 0 0 0 0 0 0 0 0 0 0 0 0 0 0
0 0 0 0 0 0 0 0 0 0 0 0 0 0 0 0 0 0 0 0 0 0 0 0 0 0 0 0 0 0 0 0 0 0 0 0 1 0 0 0 0 0 0 0 0 0 0 0 0 0 0 0 0 0 0 0 0 0 0 0 0 0 0 0 0
0 0 0 0 0 0 0 0 Log_Total_GSH*TwoGroups*Protein  0 0 0 0 0 0 0 0 0 0 0 0 0 0 0 0 0 0 0 0 0 0 0 0 0 0 0 0 0 0 0 0 0 0 0 0 0 0 0 0
0 0 0 0 0 0 0 0 0 0 0 0 0 0 0 1 0 0 0 0 0 0 0 0 0 0 0 0 0 0 0 0 0 0 0 0 0 0 0 0 0 0 0 0 0 0 0 0 0 0 0 0 0 0 0 0 0 0 0 0 0 0 0 0 0
0 0 0 0 0 0 0 0 0 0 0 0 0 0 0 0 0 0 0 0 0 0 0 0 0 0 0 0 0 0 0 0 0 0 0 0 0 0 0 0 0 0 0 0 0 0 0 0 0 0 0 0 0 0 0 0 0 0 0 0 0 0 0 0 0
0 0 0 0 0 0 0 0 0 0 0 0 0 0;
Estimate "Lpl Old Slope" Log_Total_GSH 1 Log_Total_GSH*TwoGroups 1 0 Log_Total_GSH*Protein 0 0 0 0 0 0 0 0 0 0 0 0 0 0 0 0 0 0 0
0 0 0 0 0 0 0 0 0 0 0 0 0 0 0 0 0 0 0 0 0 0 0 0 0 0 0 0 0 0 0 0 0 0 0 0 0 1 0 0 0 0 0 0 0 0 0 0 0 0 0 0 0 0 0 0 0 0 0 0 0 0 0 0 0
0 0 0 0 0 0 0 0 Log_Total_GSH*TwoGroups*Protein  0 0 0 0 0 0 0 0 0 0 0 0 0 0 0 0 0 0 0 0 0 0 0 0 0 0 0 0 0 0 0 0 0 0 0 0 0 0 0 0
0 0 0 0 0 0 0 0 0 0 0 0 0 0 0 0 1 0 0 0 0 0 0 0 0 0 0 0 0 0 0 0 0 0 0 0 0 0 0 0 0 0 0 0 0 0 0 0 0 0 0 0 0 0 0 0 0 0 0 0 0 0 0 0 0
0 0 0 0 0 0 0 0 0 0 0 0 0 0 0 0 0 0 0 0 0 0 0 0 0 0 0 0 0 0 0 0 0 0 0 0 0 0 0 0 0 0 0 0 0 0 0 0 0 0 0 0 0 0 0 0 0 0 0 0 0 0 0 0 0
0 0 0 0 0 0 0 0 0 0 0 0 0 0;
Estimate "Map2k6 Old Slope" Log_Total_GSH 1 Log_Total_GSH*TwoGroups 1 0 Log_Total_GSH*Protein 0 0 0 0 0 0 0 0 0 0 0 0 0 0 0 0 0 0
0 0 0 0 0 0 0 0 0 0 0 0 0 0 0 0 0 0 0 0 0 0 0 0 0 0 0 0 0 0 0 0 0 0 0 0 0 0 0 1 0 0 0 0 0 0 0 0 0 0 0 0 0 0 0 0 0 0 0 0 0 0 0 0 0
0 0 0 0 0 0 0 0 0 Log_Total_GSH*TwoGroups*Protein  0 0 0 0 0 0 0 0 0 0 0 0 0 0 0 0 0 0 0 0 0 0 0 0 0 0 0 0 0 0 0 0 0 0 0 0 0 0 0
0 0 0 0 0 0 0 0 0 0 0 0 0 0 0 0 0 0 1 0 0 0 0 0 0 0 0 0 0 0 0 0 0 0 0 0 0 0 0 0 0 0 0 0 0 0 0 0 0 0 0 0 0 0 0 0 0 0 0 0 0 0 0 0 0
0 0 0 0 0 0 0 0 0 0 0 0 0 0 0 0 0 0 0 0 0 0 0 0 0 0 0 0 0 0 0 0 0 0 0 0 0 0 0 0 0 0 0 0 0 0 0 0 0 0 0 0 0 0 0 0 0 0 0 0 0 0 0 0 0
0 0 0 0 0 0 0 0 0 0 0 0 0 0 0;
Estimate "Matn2 Old Slope" Log_Total_GSH 1 Log_Total_GSH*TwoGroups 1 0 Log_Total_GSH*Protein 0 0 0 0 0 0 0 0 0 0 0 0 0 0 0 0 0 0
0 0 0 0 0 0 0 0 0 0 0 0 0 0 0 0 0 0 0 0 0 0 0 0 0 0 0 0 0 0 0 0 0 0 0 0 0 0 0 0 1 0 0 0 0 0 0 0 0 0 0 0 0 0 0 0 0 0 0 0 0 0 0 0 0
0 0 0 0 0 0 0 0 0 Log_Total_GSH*TwoGroups*Protein  0 0 0 0 0 0 0 0 0 0 0 0 0 0 0 0 0 0 0 0 0 0 0 0 0 0 0 0 0 0 0 0 0 0 0 0 0 0 0
0 0 0 0 0 0 0 0 0 0 0 0 0 0 0 0 0 0 0 1 0 0 0 0 0 0 0 0 0 0 0 0 0 0 0 0 0 0 0 0 0 0 0 0 0 0 0 0 0 0 0 0 0 0 0 0 0 0 0 0 0 0 0 0 0
0 0 0 0 0 0 0 0 0 0 0 0 0 0 0 0 0 0 0 0 0 0 0 0 0 0 0 0 0 0 0 0 0 0 0 0 0 0 0 0 0 0 0 0 0 0 0 0 0 0 0 0 0 0 0 0 0 0 0 0 0 0 0 0 0
0 0 0 0 0 0 0 0 0 0 0 0 0 0 0;
Estimate "Mia Old Slope" Log_Total_GSH 1 Log_Total_GSH*TwoGroups 1 0 Log_Total_GSH*Protein 0 0 0 0 0 0 0 0 0 0 0 0 0 0 0 0 0 0 0
0 0 0 0 0 0 0 0 0 0 0 0 0 0 0 0 0 0 0 0 0 0 0 0 0 0 0 0 0 0 0 0 0 0 0 0 0 0 0 0 1 0 0 0 0 0 0 0 0 0 0 0 0 0 0 0 0 0 0 0 0 0 0 0 0
0 0 0 0 0 0 0 0 Log_Total_GSH*TwoGroups*Protein  0 0 0 0 0 0 0 0 0 0 0 0 0 0 0 0 0 0 0 0 0 0 0 0 0 0 0 0 0 0 0 0 0 0 0 0 0 0 0 0
0 0 0 0 0 0 0 0 0 0 0 0 0 0 0 0 0 0 0 1 0 0 0 0 0 0 0 0 0 0 0 0 0 0 0 0 0 0 0 0 0 0 0 0 0 0 0 0 0 0 0 0 0 0 0 0 0 0 0 0 0 0 0 0 0
0 0 0 0 0 0 0 0 0 0 0 0 0 0 0 0 0 0 0 0 0 0 0 0 0 0 0 0 0 0 0 0 0 0 0 0 0 0 0 0 0 0 0 0 0 0 0 0 0 0 0 0 0 0 0 0 0 0 0 0 0 0 0 0 0
0 0 0 0 0 0 0 0 0 0 0 0 0 0;
Estimate "Nadk Old Slope" Log_Total_GSH 1 Log_Total_GSH*TwoGroups 1 0 Log_Total_GSH*Protein 0 0 0 0 0 0 0 0 0 0 0 0 0 0 0 0 0 0 0
0 0 0 0 0 0 0 0 0 0 0 0 0 0 0 0 0 0 0 0 0 0 0 0 0 0 0 0 0 0 0 0 0 0 0 0 0 0 0 0 0 1 0 0 0 0 0 0 0 0 0 0 0 0 0 0 0 0 0 0 0 0 0 0 0
0 0 0 0 0 0 0 0 Log_Total_GSH*TwoGroups*Protein  0 0 0 0 0 0 0 0 0 0 0 0 0 0 0 0 0 0 0 0 0 0 0 0 0 0 0 0 0 0 0 0 0 0 0 0 0 0 0 0
0 0 0 0 0 0 0 0 0 0 0 0 0 0 0 0 0 0 0 0 1 0 0 0 0 0 0 0 0 0 0 0 0 0 0 0 0 0 0 0 0 0 0 0 0 0 0 0 0 0 0 0 0 0 0 0 0 0 0 0 0 0 0 0 0
0 0 0 0 0 0 0 0 0 0 0 0 0 0 0 0 0 0 0 0 0 0 0 0 0 0 0 0 0 0 0 0 0 0 0 0 0 0 0 0 0 0 0 0 0 0 0 0 0 0 0 0 0 0 0 0 0 0 0 0 0 0 0 0 0
0 0 0 0 0 0 0 0 0 0 0 0 0 0;
Estimate "Notch3 Old Slope" Log_Total_GSH 1 Log_Total_GSH*TwoGroups 1 0 Log_Total_GSH*Protein 0 0 0 0 0 0 0 0 0 0 0 0 0 0 0 0 0 0
0 0 0 0 0 0 0 0 0 0 0 0 0 0 0 0 0 0 0 0 0 0 0 0 0 0 0 0 0 0 0 0 0 0 0 0 0 0 0 0 0 0 0 1 0 0 0 0 0 0 0 0 0 0 0 0 0 0 0 0 0 0 0 0 0
0 0 0 0 0 0 0 0 0 Log_Total_GSH*TwoGroups*Protein  0 0 0 0 0 0 0 0 0 0 0 0 0 0 0 0 0 0 0 0 0 0 0 0 0 0 0 0 0 0 0 0 0 0 0 0 0 0 0
0 0 0 0 0 0 0 0 0 0 0 0 0 0 0 0 0 0 0 0 0 0 1 0 0 0 0 0 0 0 0 0 0 0 0 0 0 0 0 0 0 0 0 0 0 0 0 0 0 0 0 0 0 0 0 0 0 0 0 0 0 0 0 0 0
0 0 0 0 0 0 0 0 0 0 0 0 0 0 0 0 0 0 0 0 0 0 0 0 0 0 0 0 0 0 0 0 0 0 0 0 0 0 0 0 0 0 0 0 0 0 0 0 0 0 0 0 0 0 0 0 0 0 0 0 0 0 0 0 0
0 0 0 0 0 0 0 0 0 0 0 0 0 0 0;
Estimate "Ntf3 Old Slope" Log_Total_GSH 1 Log_Total_GSH*TwoGroups 1 0 Log_Total_GSH*Protein 0 0 0 0 0 0 0 0 0 0 0 0 0 0 0 0 0 0 0
0 0 0 0 0 0 0 0 0 0 0 0 0 0 0 0 0 0 0 0 0 0 0 0 0 0 0 0 0 0 0 0 0 0 0 0 0 0 0 0 0 0 0 1 0 0 0 0 0 0 0 0 0 0 0 0 0 0 0 0 0 0 0 0 0
0 0 0 0 0 0 0 0 Log_Total_GSH*TwoGroups*Protein  0 0 0 0 0 0 0 0 0 0 0 0 0 0 0 0 0 0 0 0 0 0 0 0 0 0 0 0 0 0 0 0 0 0 0 0 0 0 0 0
0 0 0 0 0 0 0 0 0 0 0 0 0 0 0 0 0 0 0 0 0 0 1 0 0 0 0 0 0 0 0 0 0 0 0 0 0 0 0 0 0 0 0 0 0 0 0 0 0 0 0 0 0 0 0 0 0 0 0 0 0 0 0 0 0
0 0 0 0 0 0 0 0 0 0 0 0 0 0 0 0 0 0 0 0 0 0 0 0 0 0 0 0 0 0 0 0 0 0 0 0 0 0 0 0 0 0 0 0 0 0 0 0 0 0 0 0 0 0 0 0 0 0 0 0 0 0 0 0 0
0 0 0 0 0 0 0 0 0 0 0 0 0 0;
```

```
Estimate "Pak4 Old Slope" Log_Total_GSH 1 Log_Total_GSH*TwoGroups 1 0 Log_Total_GSH*Protein 0 0 0 0 0 0 0 0 0 0 0 0 0 0 0 0 0 0 0
0 0 0 0 0 0 0 0 0 0 0 0 0 0 0 0 0 0 0 0 0 0 0 0 0 0 0 0 0 0 0 0 0 0 0 0 0 0 0 0 0 0 0 0 1 0 0 0 0 0 0 0 0 0 0 0 0 0 0 0 0 0 0 0 0
0 0 0 0 0 0 0 0 Log_Total_GSH*TwoGroups*Protein  0 0 0 0 0 0 0 0 0 0 0 0 0 0 0 0 0 0 0 0 0 0 0 0 0 0 0 0 0 0 0 0 0 0 0 0 0 0 0 0
0 0 0 0 0 0 0 0 0 0 0 0 0 0 0 0 0 0 0 0 0 0 0 1 0 0 0 0 0 0 0 0 0 0 0 0 0 0 0 0 0 0 0 0 0 0 0 0 0 0 0 0 0 0 0 0 0 0 0 0 0 0 0 0 0
0 0 0 0 0 0 0 0 0 0 0 0 0 0 0 0 0 0 0 0 0 0 0 0 0 0 0 0 0 0 0 0 0 0 0 0 0 0 0 0 0 0 0 0 0 0 0 0 0 0 0 0 0 0 0 0 0 0 0 0 0 0 0 0 0
0 0 0 0 0 0 0 0 0 0 0 0 0 0;
Estimate "Parp1 Old Slope" Log_Total_GSH 1 Log_Total_GSH*TwoGroups 1 0 Log_Total_GSH*Protein 0 0 0 0 0 0 0 0 0 0 0 0 0 0 0 0 0 0
0 0 0 0 0 0 0 0 0 0 0 0 0 0 0 0 0 0 0 0 0 0 0 0 0 0 0 0 0 0 0 0 0 0 0 0 0 0 0 0 0 0 0 0 0 0 1 0 0 0 0 0 0 0 0 0 0 0 0 0 0 0 0 0
0 0 0 0 0 0 0 0 0 Log_Total_GSH*TwoGroups*Protein  0 0 0 0 0 0 0 0 0 0 0 0 0 0 0 0 0 0 0 0 0 0 0 0 0 0 0 0 0 0 0 0 0 0 0 0 0 0 0
0 0 0 0 0 0 0 0 0 0 0 0 0 0 0 0 0 0 0 0 0 0 0 0 0 1 0 0 0 0 0 0 0 0 0 0 0 0 0 0 0 0 0 0 0 0 0 0 0 0 0 0 0 0 0 0 0 0 0 0 0 0 0 0 0
0 0 0 0 0 0 0 0 0 0 0 0 0 0 0 0 0 0 0 0 0 0 0 0 0 0 0 0 0 0 0 0 0 0 0 0 0 0 0 0 0 0 0 0 0 0 0 0 0 0 0 0 0 0 0 0 0 0 0 0 0 0 0 0 0
0 0 0 0 0 0 0 0 0 0 0 0 0 0 0;
Estimate "Pdgfb Old Slope" Log_Total_GSH 1 Log_Total_GSH*TwoGroups 1 0 Log_Total_GSH*Protein 0 0 0 0 0 0 0 0 0 0 0 0 0 0 0 0 0 0
0 0 0 0 0 0 0 0 0 0 0 0 0 0 0 0 0 0 0 0 0 0 0 0 0 0 0 0 0 0 0 0 0 0 0 0 0 0 0 0 0 0 0 0 0 0 0 1 0 0 0 0 0 0 0 0 0 0 0 0 0 0 0 0
0 0 0 0 0 0 0 0 0 Log_Total_GSH*TwoGroups*Protein  0 0 0 0 0 0 0 0 0 0 0 0 0 0 0 0 0 0 0 0 0 0 0 0 0 0 0 0 0 0 0 0 0 0 0 0 0 0 0
0 0 0 0 0 0 0 0 0 0 0 0 0 0 0 0 0 0 0 0 0 0 0 0 0 0 1 0 0 0 0 0 0 0 0 0 0 0 0 0 0 0 0 0 0 0 0 0 0 0 0 0 0 0 0 0 0 0 0 0 0 0 0 0 0
0 0 0 0 0 0 0 0 0 0 0 0 0 0 0 0 0 0 0 0 0 0 0 0 0 0 0 0 0 0 0 0 0 0 0 0 0 0 0 0 0 0 0 0 0 0 0 0 0 0 0 0 0 0 0 0 0 0 0 0 0 0 0 0 0
0 0 0 0 0 0 0 0 0 0 0 0 0 0 0;
Estimate "Pla2g4a Old Slope" Log_Total_GSH 1 Log_Total_GSH*TwoGroups 1 0 Log_Total_GSH*Protein 0 0 0 0 0 0 0 0 0 0 0 0 0 0 0 0 0
0 0 0 0 0 0 0 0 0 0 0 0 0 0 0 0 0 0 0 0 0 0 0 0 0 0 0 0 0 0 0 0 0 0 0 0 0 0 0 0 0 0 0 0 0 0 0 0 0 1 0 0 0 0 0 0 0 0 0 0 0 0 0 0 0
0 0 0 0 0 0 0 0 0 0 Log_Total_GSH*TwoGroups*Protein  0 0 0 0 0 0 0 0 0 0 0 0 0 0 0 0 0 0 0 0 0 0 0 0 0 0 0 0 0 0 0 0 0 0 0 0 0 0
0 0 0 0 0 0 0 0 0 0 0 0 0 0 0 0 0 0 0 0 0 0 0 0 0 0 0 0 1 0 0 0 0 0 0 0 0 0 0 0 0 0 0 0 0 0 0 0 0 0 0 0 0 0 0 0 0 0 0 0 0 0 0 0 0
0 0 0 0 0 0 0 0 0 0 0 0 0 0 0 0 0 0 0 0 0 0 0 0 0 0 0 0 0 0 0 0 0 0 0 0 0 0 0 0 0 0 0 0 0 0 0 0 0 0 0 0 0 0 0 0 0 0 0 0 0 0 0 0 0
0 0 0 0 0 0 0 0 0 0 0 0 0 0 0 0;
Estimate "Plin1 Old Slope" Log_Total_GSH 1 Log_Total_GSH*TwoGroups 1 0 Log_Total_GSH*Protein 0 0 0 0 0 0 0 0 0 0 0 0 0 0 0 0 0 0
0 0 0 0 0 0 0 0 0 0 0 0 0 0 0 0 0 0 0 0 0 0 0 0 0 0 0 0 0 0 0 0 0 0 0 0 0 0 0 0 0 0 0 0 0 0 0 0 0 1 0 0 0 0 0 0 0 0 0 0 0 0 0 0 0
0 0 0 0 0 0 0 0 0 Log_Total_GSH*TwoGroups*Protein  0 0 0 0 0 0 0 0 0 0 0 0 0 0 0 0 0 0 0 0 0 0 0 0 0 0 0 0 0 0 0 0 0 0 0 0 0 0 0
0 0 0 0 0 0 0 0 0 0 0 0 0 0 0 0 0 0 0 0 0 0 0 0 0 0 0 0 1 0 0 0 0 0 0 0 0 0 0 0 0 0 0 0 0 0 0 0 0 0 0 0 0 0 0 0 0 0 0 0 0 0 0 0 0
0 0 0 0 0 0 0 0 0 0 0 0 0 0 0 0 0 0 0 0 0 0 0 0 0 0 0 0 0 0 0 0 0 0 0 0 0 0 0 0 0 0 0 0 0 0 0 0 0 0 0 0 0 0 0 0 0 0 0 0 0 0 0 0 0
0 0 0 0 0 0 0 0 0 0 0 0 0 0 0;
Estimate "Plxna4 Old Slope" Log_Total_GSH 1 Log_Total_GSH*TwoGroups 1 0 Log_Total_GSH*Protein 0 0 0 0 0 0 0 0 0 0 0 0 0 0 0 0 0 0
0 0 0 0 0 0 0 0 0 0 0 0 0 0 0 0 0 0 0 0 0 0 0 0 0 0 0 0 0 0 0 0 0 0 0 0 0 0 0 0 0 0 0 0 0 0 0 0 0 0 1 0 0 0 0 0 0 0 0 0 0 0 0 0 0
0 0 0 0 0 0 0 0 0 Log_Total_GSH*TwoGroups*Protein  0 0 0 0 0 0 0 0 0 0 0 0 0 0 0 0 0 0 0 0 0 0 0 0 0 0 0 0 0 0 0 0 0 0 0 0 0 0 0
0 0 0 0 0 0 0 0 0 0 0 0 0 0 0 0 0 0 0 0 0 0 0 0 0 0 0 0 0 1 0 0 0 0 0 0 0 0 0 0 0 0 0 0 0 0 0 0 0 0 0 0 0 0 0 0 0 0 0 0 0 0 0 0 0
0 0 0 0 0 0 0 0 0 0 0 0 0 0 0 0 0 0 0 0 0 0 0 0 0 0 0 0 0 0 0 0 0 0 0 0 0 0 0 0 0 0 0 0 0 0 0 0 0 0 0 0 0 0 0 0 0 0 0 0 0 0 0 0 0
0 0 0 0 0 0 0 0 0 0 0 0 0 0 0;
Estimate "Ppp1r2 Old Slope" Log_Total_GSH 1 Log_Total_GSH*TwoGroups 1 0 Log_Total_GSH*Protein 0 0 0 0 0 0 0 0 0 0 0 0 0 0 0 0 0 0
0 0 0 0 0 0 0 0 0 0 0 0 0 0 0 0 0 0 0 0 0 0 0 0 0 0 0 0 0 0 0 0 0 0 0 0 0 0 0 0 0 0 0 0 0 0 0 0 0 0 0 1 0 0 0 0 0 0 0 0 0 0 0 0 0
0 0 0 0 0 0 0 0 0 Log_Total_GSH*TwoGroups*Protein  0 0 0 0 0 0 0 0 0 0 0 0 0 0 0 0 0 0 0 0 0 0 0 0 0 0 0 0 0 0 0 0 0 0 0 0 0 0 0
0 0 0 0 0 0 0 0 0 0 0 0 0 0 0 0 0 0 0 0 0 0 0 0 0 0 0 0 0 0 1 0 0 0 0 0 0 0 0 0 0 0 0 0 0 0 0 0 0 0 0 0 0 0 0 0 0 0 0 0 0 0 0 0 0
0 0 0 0 0 0 0 0 0 0 0 0 0 0 0 0 0 0 0 0 0 0 0 0 0 0 0 0 0 0 0 0 0 0 0 0 0 0 0 0 0 0 0 0 0 0 0 0 0 0 0 0 0 0 0 0 0 0 0 0 0 0 0 0 0
0 0 0 0 0 0 0 0 0 0 0 0 0 0 0;
Estimate "Prdx5 Old Slope" Log_Total_GSH 1 Log_Total_GSH*TwoGroups 1 0 Log_Total_GSH*Protein 0 0 0 0 0 0 0 0 0 0 0 0 0 0 0 0 0 0
0 0 0 0 0 0 0 0 0 0 0 0 0 0 0 0 0 0 0 0 0 0 0 0 0 0 0 0 0 0 0 0 0 0 0 0 0 0 0 0 0 0 0 0 0 0 0 0 0 0 0 0 1 0 0 0 0 0 0 0 0 0 0 0 0
0 0 0 0 0 0 0 0 0 Log_Total_GSH*TwoGroups*Protein  0 0 0 0 0 0 0 0 0 0 0 0 0 0 0 0 0 0 0 0 0 0 0 0 0 0 0 0 0 0 0 0 0 0 0 0 0 0 0
0 0 0 0 0 0 0 0 0 0 0 0 0 0 0 0 0 0 0 0 0 0 0 0 0 0 0 0 0 0 0 1 0 0 0 0 0 0 0 0 0 0 0 0 0 0 0 0 0 0 0 0 0 0 0 0 0 0 0 0 0 0 0 0 0
0 0 0 0 0 0 0 0 0 0 0 0 0 0 0 0 0 0 0 0 0 0 0 0 0 0 0 0 0 0 0 0 0 0 0 0 0 0 0 0 0 0 0 0 0 0 0 0 0 0 0 0 0 0 0 0 0 0 0 0 0 0 0 0 0
0 0 0 0 0 0 0 0 0 0 0 0 0 0 0;
Estimate "Qdpr Old Slope" Log_Total_GSH 1 Log_Total_GSH*TwoGroups 1 0 Log_Total_GSH*Protein 0 0 0 0 0 0 0 0 0 0 0 0 0 0 0 0 0 0 0
0 0 0 0 0 0 0 0 0 0 0 0 0 0 0 0 0 0 0 0 0 0 0 0 0 0 0 0 0 0 0 0 0 0 0 0 0 0 0 0 0 0 0 0 0 0 0 0 0 0 0 0 1 0 0 0 0 0 0 0 0 0 0 0 0
0 0 0 0 0 0 0 0 Log_Total_GSH*TwoGroups*Protein  0 0 0 0 0 0 0 0 0 0 0 0 0 0 0 0 0 0 0 0 0 0 0 0 0 0 0 0 0 0 0 0 0 0 0 0 0 0 0 0
0 0 0 0 0 0 0 0 0 0 0 0 0 0 0 0 0 0 0 0 0 0 0 0 0 0 0 0 0 0 0 1 0 0 0 0 0 0 0 0 0 0 0 0 0 0 0 0 0 0 0 0 0 0 0 0 0 0 0 0 0 0 0 0 0
0 0 0 0 0 0 0 0 0 0 0 0 0 0 0 0 0 0 0 0 0 0 0 0 0 0 0 0 0 0 0 0 0 0 0 0 0 0 0 0 0 0 0 0 0 0 0 0 0 0 0 0 0 0 0 0 0 0 0 0 0 0 0 0 0
0 0 0 0 0 0 0 0 0 0 0 0 0 0;
Estimate "Rgma Old Slope" Log_Total_GSH 1 Log_Total_GSH*TwoGroups 1 0 Log_Total_GSH*Protein 0 0 0 0 0 0 0 0 0 0 0 0 0 0 0 0 0 0 0
0 0 0 0 0 0 0 0 0 0 0 0 0 0 0 0 0 0 0 0 0 0 0 0 0 0 0 0 0 0 0 0 0 0 0 0 0 0 0 0 0 0 0 0 0 0 0 0 0 0 0 0 0 1 0 0 0 0 0 0 0 0 0 0 0
0 0 0 0 0 0 0 0 Log_Total_GSH*TwoGroups*Protein  0 0 0 0 0 0 0 0 0 0 0 0 0 0 0 0 0 0 0 0 0 0 0 0 0 0 0 0 0 0 0 0 0 0 0 0 0 0 0 0
0 0 0 0 0 0 0 0 0 0 0 0 0 0 0 0 0 0 0 0 0 0 0 0 0 0 0 0 0 0 0 0 1 0 0 0 0 0 0 0 0 0 0 0 0 0 0 0 0 0 0 0 0 0 0 0 0 0 0 0 0 0 0 0 0
0 0 0 0 0 0 0 0 0 0 0 0 0 0 0 0 0 0 0 0 0 0 0 0 0 0 0 0 0 0 0 0 0 0 0 0 0 0 0 0 0 0 0 0 0 0 0 0 0 0 0 0 0 0 0 0 0 0 0 0 0 0 0 0 0
0 0 0 0 0 0 0 0 0 0 0 0 0 0;
Estimate "Riox2 Old Slope" Log_Total_GSH 1 Log_Total_GSH*TwoGroups 1 0 Log_Total_GSH*Protein 0 0 0 0 0 0 0 0 0 0 0 0 0 0 0 0 0 0
0 0 0 0 0 0 0 0 0 0 0 0 0 0 0 0 0 0 0 0 0 0 0 0 0 0 0 0 0 0 0 0 0 0 0 0 0 0 0 0 0 0 0 0 0 0 0 0 0 0 0 0 0 0 0 1 0 0 0 0 0 0 0 0 0
0 0 0 0 0 0 0 0 0 Log_Total_GSH*TwoGroups*Protein  0 0 0 0 0 0 0 0 0 0 0 0 0 0 0 0 0 0 0 0 0 0 0 0 0 0 0 0 0 0 0 0 0 0 0 0 0 0 0
0 0 0 0 0 0 0 0 0 0 0 0 0 0 0 0 0 0 0 0 0 0 0 0 0 0 0 0 0 0 0 0 0 0 1 0 0 0 0 0 0 0 0 0 0 0 0 0 0 0 0 0 0 0 0 0 0 0 0 0 0 0 0 0 0
0 0 0 0 0 0 0 0 0 0 0 0 0 0 0 0 0 0 0 0 0 0 0 0 0 0 0 0 0 0 0 0 0 0 0 0 0 0 0 0 0 0 0 0 0 0 0 0 0 0 0 0 0 0 0 0 0 0 0 0 0 0 0 0 0
0 0 0 0 0 0 0 0 0 0 0 0 0 0 0;
Estimate "S100a4 Old Slope" Log_Total_GSH 1 Log_Total_GSH*TwoGroups 1 0 Log_Total_GSH*Protein 0 0 0 0 0 0 0 0 0 0 0 0 0 0 0 0 0 0
0 0 0 0 0 0 0 0 0 0 0 0 0 0 0 0 0 0 0 0 0 0 0 0 0 0 0 0 0 0 0 0 0 0 0 0 0 0 0 0 0 0 0 0 0 0 0 0 0 0 0 0 0 0 0 0 1 0 0 0 0 0 0 0 0
0 0 0 0 0 0 0 0 0 Log_Total_GSH*TwoGroups*Protein  0 0 0 0 0 0 0 0 0 0 0 0 0 0 0 0 0 0 0 0 0 0 0 0 0 0 0 0 0 0 0 0 0 0 0 0 0 0 0
0 0 0 0 0 0 0 0 0 0 0 0 0 0 0 0 0 0 0 0 0 0 0 0 0 0 0 0 0 0 0 0 0 0 0 1 0 0 0 0 0 0 0 0 0 0 0 0 0 0 0 0 0 0 0 0 0 0 0 0 0 0 0 0 0
0 0 0 0 0 0 0 0 0 0 0 0 0 0 0 0 0 0 0 0 0 0 0 0 0 0 0 0 0 0 0 0 0 0 0 0 0 0 0 0 0 0 0 0 0 0 0 0 0 0 0 0 0 0 0 0 0 0 0 0 0 0 0 0 0
0 0 0 0 0 0 0 0 0 0 0 0 0 0 0;
Estimate "Sez6l2 Old Slope" Log_Total_GSH 1 Log_Total_GSH*TwoGroups 1 0 Log_Total_GSH*Protein 0 0 0 0 0 0 0 0 0 0 0 0 0 0 0 0 0 0
0 0 0 0 0 0 0 0 0 0 0 0 0 0 0 0 0 0 0 0 0 0 0 0 0 0 0 0 0 0 0 0 0 0 0 0 0 0 0 0 0 0 0 0 0 0 0 0 0 0 0 0 0 0 0 0 0 1 0 0 0 0 0 0 0
0 0 0 0 0 0 0 0 0 Log_Total_GSH*TwoGroups*Protein  0 0 0 0 0 0 0 0 0 0 0 0 0 0 0 0 0 0 0 0 0 0 0 0 0 0 0 0 0 0 0 0 0 0 0 0 0 0 0
0 0 0 0 0 0 0 0 0 0 0 0 0 0 0 0 0 0 0 0 0 0 0 0 0 0 0 0 0 0 0 0 0 0 0 0 1 0 0 0 0 0 0 0 0 0 0 0 0 0 0 0 0 0 0 0 0 0 0 0 0 0 0 0 0
0 0 0 0 0 0 0 0 0 0 0 0 0 0 0 0 0 0 0 0 0 0 0 0 0 0 0 0 0 0 0 0 0 0 0 0 0 0 0 0 0 0 0 0 0 0 0 0 0 0 0 0 0 0 0 0 0 0 0 0 0 0 0 0 0
0 0 0 0 0 0 0 0 0 0 0 0 0 0 0;
Estimate "Snap29 Old Slope" Log_Total_GSH 1 Log_Total_GSH*TwoGroups 1 0 Log_Total_GSH*Protein 0 0 0 0 0 0 0 0 0 0 0 0 0 0 0 0 0 0
0 0 0 0 0 0 0 0 0 0 0 0 0 0 0 0 0 0 0 0 0 0 0 0 0 0 0 0 0 0 0 0 0 0 0 0 0 0 0 0 0 0 0 0 0 0 0 0 0 0 0 0 0 0 0 0 0 0 1 0 0 0 0 0 0
0 0 0 0 0 0 0 0 0 Log_Total_GSH*TwoGroups*Protein  0 0 0 0 0 0 0 0 0 0 0 0 0 0 0 0 0 0 0 0 0 0 0 0 0 0 0 0 0 0 0 0 0 0 0 0 0 0 0
0 0 0 0 0 0 0 0 0 0 0 0 0 0 0 0 0 0 0 0 0 0 0 0 0 0 0 0 0 0 0 0 0 0 0 0 0 1 0 0 0 0 0 0 0 0 0 0 0 0 0 0 0 0 0 0 0 0 0 0 0 0 0 0 0
0 0 0 0 0 0 0 0 0 0 0 0 0 0 0 0 0 0 0 0 0 0 0 0 0 0 0 0 0 0 0 0 0 0 0 0 0 0 0 0 0 0 0 0 0 0 0 0 0 0 0 0 0 0 0 0 0 0 0 0 0 0 0 0 0
0 0 0 0 0 0 0 0 0 0 0 0 0 0 0;
Estimate "Tgfa Old Slope" Log_Total_GSH 1 Log_Total_GSH*TwoGroups 1 0 Log_Total_GSH*Protein 0 0 0 0 0 0 0 0 0 0 0 0 0 0 0 0 0 0 0
0 0 0 0 0 0 0 0 0 0 0 0 0 0 0 0 0 0 0 0 0 0 0 0 0 0 0 0 0 0 0 0 0 0 0 0 0 0 0 0 0 0 0 0 0 0 0 0 0 0 0 0 0 0 0 0 0 0 1 0 0 0 0 0 0
0 0 0 0 0 0 0 0 Log_Total_GSH*TwoGroups*Protein  0 0 0 0 0 0 0 0 0 0 0 0 0 0 0 0 0 0 0 0 0 0 0 0 0 0 0 0 0 0 0 0 0 0 0 0 0 0 0 0
0 0 0 0 0 0 0 0 0 0 0 0 0 0 0 0 0 0 0 0 0 0 0 0 0 0 0 0 0 0 0 0 0 0 0 0 0 1 0 0 0 0 0 0 0 0 0 0 0 0 0 0 0 0 0 0 0 0 0 0 0 0 0 0 0
0 0 0 0 0 0 0 0 0 0 0 0 0 0 0 0 0 0 0 0 0 0 0 0 0 0 0 0 0 0 0 0 0 0 0 0 0 0 0 0 0 0 0 0 0 0 0 0 0 0 0 0 0 0 0 0 0 0 0 0 0 0 0 0 0
0 0 0 0 0 0 0 0 0 0 0 0 0 0;
Estimate "Tgfb1 Old Slope" Log_Total_GSH 1 Log_Total_GSH*TwoGroups 1 0 Log_Total_GSH*Protein 0 0 0 0 0 0 0 0 0 0 0 0 0 0 0 0 0 0
0 0 0 0 0 0 0 0 0 0 0 0 0 0 0 0 0 0 0 0 0 0 0 0 0 0 0 0 0 0 0 0 0 0 0 0 0 0 0 0 0 0 0 0 0 0 0 0 0 0 0 0 0 0 0 0 0 0 0 0 1 0 0 0 0
0 0 0 0 0 0 0 0 0 Log_Total_GSH*TwoGroups*Protein  0 0 0 0 0 0 0 0 0 0 0 0 0 0 0 0 0 0 0 0 0 0 0 0 0 0 0 0 0 0 0 0 0 0 0 0 0 0 0
0 0 0 0 0 0 0 0 0 0 0 0 0 0 0 0 0 0 0 0 0 0 0 0 0 0 0 0 0 0 0 0 0 0 0 0 0 0 0 1 0 0 0 0 0 0 0 0 0 0 0 0 0 0 0 0 0 0 0 0 0 0 0 0 0
```

```
0 0 0 0 0 0 0 0 0 0 0 0 0 0 0 0 0 0 0 0 0 0 0 0 0 0 0 0 0 0 0 0 0 0 0 0 0 0 0 0 0 0 0 0 0 0 0 0 0 0 0 0 0 0 0 0 0 0 0 0 0 0 0 0 0
0 0 0 0 0 0 0 0 0 0 0 0 0 0 0;
Estimate "Tgfbr3 Old Slope" Log_Total_GSH 1 Log_Total_GSH*TwoGroups 1 0 Log_Total_GSH*Protein 0 0 0 0 0 0 0 0 0 0 0 0 0 0 0 0 0 0
0 0 0 0 0 0 0 0 0 0 0 0 0 0 0 0 0 0 0 0 0 0 0 0 0 0 0 0 0 0 0 0 0 0 0 0 0 0 0 0 0 0 0 0 0 0 0 0 0 0 0 0 0 0 0 0 0 0 0 0 0 1 0 0 0
0 0 0 0 0 0 0 0 0 Log_Total_GSH*TwoGroups*Protein  0 0 0 0 0 0 0 0 0 0 0 0 0 0 0 0 0 0 0 0 0 0 0 0 0 0 0 0 0 0 0 0 0 0 0 0 0 0 0
0 0 0 0 0 0 0 0 0 0 0 0 0 0 0 0 0 0 0 0 0 0 0 0 0 0 0 0 0 0 0 0 0 0 0 0 0 0 0 0 1 0 0 0 0 0 0 0 0 0 0 0 0 0 0 0 0 0 0 0 0 0 0 0 0
0 0 0 0 0 0 0 0 0 0 0 0 0 0 0 0 0 0 0 0 0 0 0 0 0 0 0 0 0 0 0 0 0 0 0 0 0 0 0 0 0 0 0 0 0 0 0 0 0 0 0 0 0 0 0 0 0 0 0 0 0 0 0 0 0
0 0 0 0 0 0 0 0 0 0 0 0 0 0 0;
Estimate "Tnf Old Slope" Log_Total_GSH 1 Log_Total_GSH*TwoGroups 1 0 Log_Total_GSH*Protein 0 0 0 0 0 0 0 0 0 0 0 0 0 0 0 0 0 0 0
0 0 0 0 0 0 0 0 0 0 0 0 0 0 0 0 0 0 0 0 0 0 0 0 0 0 0 0 0 0 0 0 0 0 0 0 0 0 0 0 0 0 0 0 0 0 0 0 0 0 0 0 0 0 0 0 0 0 0 0 0 1 0 0 0
0 0 0 0 0 0 0 0 Log_Total_GSH*TwoGroups*Protein  0 0 0 0 0 0 0 0 0 0 0 0 0 0 0 0 0 0 0 0 0 0 0 0 0 0 0 0 0 0 0 0 0 0 0 0 0 0 0 0
0 0 0 0 0 0 0 0 0 0 0 0 0 0 0 0 0 0 0 0 0 0 0 0 0 0 0 0 0 0 0 0 0 0 0 0 0 0 0 0 1 0 0 0 0 0 0 0 0 0 0 0 0 0 0 0 0 0 0 0 0 0 0 0 0
0 0 0 0 0 0 0 0 0 0 0 0 0 0 0 0 0 0 0 0 0 0 0 0 0 0 0 0 0 0 0 0 0 0 0 0 0 0 0 0 0 0 0 0 0 0 0 0 0 0 0 0 0 0 0 0 0 0 0 0 0 0 0 0 0
0 0 0 0 0 0 0 0 0 0 0 0 0 0;
Estimate "Tnfrsf11b Old Slope" Log_Total_GSH 1 Log_Total_GSH*TwoGroups 1 0 Log_Total_GSH*Protein 0 0 0 0 0 0 0 0 0 0 0 0 0 0 0 0
0 0 0 0 0 0 0 0 0 0 0 0 0 0 0 0 0 0 0 0 0 0 0 0 0 0 0 0 0 0 0 0 0 0 0 0 0 0 0 0 0 0 0 0 0 0 0 0 0 0 0 0 0 0 0 0 0 0 0 0 0 0 0 0 0
1 0 0 0 0 0 0 0 0 0 0 Log_Total_GSH*TwoGroups*Protein  0 0 0 0 0 0 0 0 0 0 0 0 0 0 0 0 0 0 0 0 0 0 0 0 0 0 0 0 0 0 0 0 0 0 0 0 0
0 0 0 0 0 0 0 0 0 0 0 0 0 0 0 0 0 0 0 0 0 0 0 0 0 0 0 0 0 0 0 0 0 0 0 0 0 0 0 0 0 0 0 0 1 0 0 0 0 0 0 0 0 0 0 0 0 0 0 0 0 0 0 0 0
0 0 0 0 0 0 0 0 0 0 0 0 0 0 0 0 0 0 0 0 0 0 0 0 0 0 0 0 0 0 0 0 0 0 0 0 0 0 0 0 0 0 0 0 0 0 0 0 0 0 0 0 0 0 0 0 0 0 0 0 0 0 0 0 0
0 0 0 0 0 0 0 0 0 0 0 0 0 0 0 0 0;
Estimate "Tnfrsf12a Old Slope" Log_Total_GSH 1 Log_Total_GSH*TwoGroups 1 0 Log_Total_GSH*Protein 0 0 0 0 0 0 0 0 0 0 0 0 0 0 0 0
0 0 0 0 0 0 0 0 0 0 0 0 0 0 0 0 0 0 0 0 0 0 0 0 0 0 0 0 0 0 0 0 0 0 0 0 0 0 0 0 0 0 0 0 0 0 0 0 0 0 0 0 0 0 0 0 0 0 0 0 0 0 0 0 0
0 1 0 0 0 0 0 0 0 0 0 Log_Total_GSH*TwoGroups*Protein  0 0 0 0 0 0 0 0 0 0 0 0 0 0 0 0 0 0 0 0 0 0 0 0 0 0 0 0 0 0 0 0 0 0 0 0 0
0 0 0 0 0 0 0 0 0 0 0 0 0 0 0 0 0 0 0 0 0 0 0 0 0 0 0 0 0 0 0 0 0 0 0 0 0 0 0 0 0 0 0 0 0 1 0 0 0 0 0 0 0 0 0 0 0 0 0 0 0 0 0 0 0
0 0 0 0 0 0 0 0 0 0 0 0 0 0 0 0 0 0 0 0 0 0 0 0 0 0 0 0 0 0 0 0 0 0 0 0 0 0 0 0 0 0 0 0 0 0 0 0 0 0 0 0 0 0 0 0 0 0 0 0 0 0 0 0 0
0 0 0 0 0 0 0 0 0 0 0 0 0 0 0 0 0;
Estimate "Tnfsf12 Old Slope" Log_Total_GSH 1 Log_Total_GSH*TwoGroups 1 0 Log_Total_GSH*Protein 0 0 0 0 0 0 0 0 0 0 0 0 0 0 0 0 0
0 0 0 0 0 0 0 0 0 0 0 0 0 0 0 0 0 0 0 0 0 0 0 0 0 0 0 0 0 0 0 0 0 0 0 0 0 0 0 0 0 0 0 0 0 0 0 0 0 0 0 0 0 0 0 0 0 0 0 0 0 0 0 0 0
0 1 0 0 0 0 0 0 0 0 Log_Total_GSH*TwoGroups*Protein  0 0 0 0 0 0 0 0 0 0 0 0 0 0 0 0 0 0 0 0 0 0 0 0 0 0 0 0 0 0 0 0 0 0 0 0 0 0
0 0 0 0 0 0 0 0 0 0 0 0 0 0 0 0 0 0 0 0 0 0 0 0 0 0 0 0 0 0 0 0 0 0 0 0 0 0 0 0 0 0 0 0 0 1 0 0 0 0 0 0 0 0 0 0 0 0 0 0 0 0 0 0 0
0 0 0 0 0 0 0 0 0 0 0 0 0 0 0 0 0 0 0 0 0 0 0 0 0 0 0 0 0 0 0 0 0 0 0 0 0 0 0 0 0 0 0 0 0 0 0 0 0 0 0 0 0 0 0 0 0 0 0 0 0 0 0 0 0
0 0 0 0 0 0 0 0 0 0 0 0 0 0 0 0;
Estimate "Tnni3 Old Slope" Log_Total_GSH 1 Log_Total_GSH*TwoGroups 1 0 Log_Total_GSH*Protein 0 0 0 0 0 0 0 0 0 0 0 0 0 0 0 0 0 0
0 0 0 0 0 0 0 0 0 0 0 0 0 0 0 0 0 0 0 0 0 0 0 0 0 0 0 0 0 0 0 0 0 0 0 0 0 0 0 0 0 0 0 0 0 0 0 0 0 0 0 0 0 0 0 0 0 0 0 0 0 0 0 0 0
0 1 0 0 0 0 0 0 0 Log_Total_GSH*TwoGroups*Protein  0 0 0 0 0 0 0 0 0 0 0 0 0 0 0 0 0 0 0 0 0 0 0 0 0 0 0 0 0 0 0 0 0 0 0 0 0 0 0
0 0 0 0 0 0 0 0 0 0 0 0 0 0 0 0 0 0 0 0 0 0 0 0 0 0 0 0 0 0 0 0 0 0 0 0 0 0 0 0 0 0 0 0 0 1 0 0 0 0 0 0 0 0 0 0 0 0 0 0 0 0 0 0 0
0 0 0 0 0 0 0 0 0 0 0 0 0 0 0 0 0 0 0 0 0 0 0 0 0 0 0 0 0 0 0 0 0 0 0 0 0 0 0 0 0 0 0 0 0 0 0 0 0 0 0 0 0 0 0 0 0 0 0 0 0 0 0 0 0
0 0 0 0 0 0 0 0 0 0 0 0 0 0 0;
Estimate "Tnr Old Slope" Log_Total_GSH 1 Log_Total_GSH*TwoGroups 1 0 Log_Total_GSH*Protein 0 0 0 0 0 0 0 0 0 0 0 0 0 0 0 0 0 0 0
0 0 0 0 0 0 0 0 0 0 0 0 0 0 0 0 0 0 0 0 0 0 0 0 0 0 0 0 0 0 0 0 0 0 0 0 0 0 0 0 0 0 0 0 0 0 0 0 0 0 0 0 0 0 0 0 0 0 0 0 0 0 0 0 0
0 1 0 0 0 0 0 0 Log_Total_GSH*TwoGroups*Protein  0 0 0 0 0 0 0 0 0 0 0 0 0 0 0 0 0 0 0 0 0 0 0 0 0 0 0 0 0 0 0 0 0 0 0 0 0 0 0 0
0 0 0 0 0 0 0 0 0 0 0 0 0 0 0 0 0 0 0 0 0 0 0 0 0 0 0 0 0 0 0 0 0 0 0 0 0 0 0 0 0 0 0 0 0 1 0 0 0 0 0 0 0 0 0 0 0 0 0 0 0 0 0 0 0
0 0 0 0 0 0 0 0 0 0 0 0 0 0 0 0 0 0 0 0 0 0 0 0 0 0 0 0 0 0 0 0 0 0 0 0 0 0 0 0 0 0 0 0 0 0 0 0 0 0 0 0 0 0 0 0 0 0 0 0 0 0 0 0 0
0 0 0 0 0 0 0 0 0 0 0 0 0 0;
Estimate "Tpp1 Old Slope" Log_Total_GSH 1 Log_Total_GSH*TwoGroups 1 0 Log_Total_GSH*Protein 0 0 0 0 0 0 0 0 0 0 0 0 0 0 0 0 0 0 0
0 0 0 0 0 0 0 0 0 0 0 0 0 0 0 0 0 0 0 0 0 0 0 0 0 0 0 0 0 0 0 0 0 0 0 0 0 0 0 0 0 0 0 0 0 0 0 0 0 0 0 0 0 0 0 0 0 0 0 0 0 0 0 0 0
0 0 1 0 0 0 0 0 Log_Total_GSH*TwoGroups*Protein  0 0 0 0 0 0 0 0 0 0 0 0 0 0 0 0 0 0 0 0 0 0 0 0 0 0 0 0 0 0 0 0 0 0 0 0 0 0 0 0
0 0 0 0 0 0 0 0 0 0 0 0 0 0 0 0 0 0 0 0 0 0 0 0 0 0 0 0 0 0 0 0 0 0 0 0 0 0 0 0 0 0 0 0 0 0 1 0 0 0 0 0 0 0 0 0 0 0 0 0 0 0 0 0 0
0 0 0 0 0 0 0 0 0 0 0 0 0 0 0 0 0 0 0 0 0 0 0 0 0 0 0 0 0 0 0 0 0 0 0 0 0 0 0 0 0 0 0 0 0 0 0 0 0 0 0 0 0 0 0 0 0 0 0 0 0 0 0 0 0
0 0 0 0 0 0 0 0 0 0 0 0 0 0;
Estimate "Vegfd Old Slope" Log_Total_GSH 1 Log_Total_GSH*TwoGroups 1 0 Log_Total_GSH*Protein 0 0 0 0 0 0 0 0 0 0 0 0 0 0 0 0 0 0
0 0 0 0 0 0 0 0 0 0 0 0 0 0 0 0 0 0 0 0 0 0 0 0 0 0 0 0 0 0 0 0 0 0 0 0 0 0 0 0 0 0 0 0 0 0 0 0 0 0 0 0 0 0 0 0 0 0 0 0 0 0 0 0 0
0 0 0 0 1 0 0 0 0 Log_Total_GSH*TwoGroups*Protein  0 0 0 0 0 0 0 0 0 0 0 0 0 0 0 0 0 0 0 0 0 0 0 0 0 0 0 0 0 0 0 0 0 0 0 0 0 0 0
0 0 0 0 0 0 0 0 0 0 0 0 0 0 0 0 0 0 0 0 0 0 0 0 0 0 0 0 0 0 0 0 0 0 0 0 0 0 0 0 0 0 0 0 0 0 0 0 1 0 0 0 0 0 0 0 0 0 0 0 0 0 0 0 0
0 0 0 0 0 0 0 0 0 0 0 0 0 0 0 0 0 0 0 0 0 0 0 0 0 0 0 0 0 0 0 0 0 0 0 0 0 0 0 0 0 0 0 0 0 0 0 0 0 0 0 0 0 0 0 0 0 0 0 0 0 0 0 0 0
0 0 0 0 0 0 0 0 0 0 0 0 0 0 0;
Estimate "Vsig2 Old Slope" Log_Total_GSH 1 Log_Total_GSH*TwoGroups 1 0 Log_Total_GSH*Protein 0 0 0 0 0 0 0 0 0 0 0 0 0 0 0 0 0 0
0 0 0 0 0 0 0 0 0 0 0 0 0 0 0 0 0 0 0 0 0 0 0 0 0 0 0 0 0 0 0 0 0 0 0 0 0 0 0 0 0 0 0 0 0 0 0 0 0 0 0 0 0 0 0 0 0 0 0 0 0 0 0 0 0
0 0 0 0 0 1 0 0 0 Log_Total_GSH*TwoGroups*Protein  0 0 0 0 0 0 0 0 0 0 0 0 0 0 0 0 0 0 0 0 0 0 0 0 0 0 0 0 0 0 0 0 0 0 0 0 0 0 0
0 0 0 0 0 0 0 0 0 0 0 0 0 0 0 0 0 0 0 0 0 0 0 0 0 0 0 0 0 0 0 0 0 0 0 0 0 0 0 0 0 0 0 0 0 0 0 0 0 1 0 0 0 0 0 0 0 0 0 0 0 0 0 0 0
0 0 0 0 0 0 0 0 0 0 0 0 0 0 0 0 0 0 0 0 0 0 0 0 0 0 0 0 0 0 0 0 0 0 0 0 0 0 0 0 0 0 0 0 0 0 0 0 0 0 0 0 0 0 0 0 0 0 0 0 0 0 0 0 0
0 0 0 0 0 0 0 0 0 0 0 0 0 0 0;
Estimate "Wfikkn2 Old Slope" Log_Total_GSH 1 Log_Total_GSH*TwoGroups 1 0 Log_Total_GSH*Protein 0 0 0 0 0 0 0 0 0 0 0 0 0 0 0 0 0
0 0 0 0 0 0 0 0 0 0 0 0 0 0 0 0 0 0 0 0 0 0 0 0 0 0 0 0 0 0 0 0 0 0 0 0 0 0 0 0 0 0 0 0 0 0 0 0 0 0 0 0 0 0 0 0 0 0 0 0 0 0 0 0 0
0 0 0 0 0 0 0 1 0 0 Log_Total_GSH*TwoGroups*Protein  0 0 0 0 0 0 0 0 0 0 0 0 0 0 0 0 0 0 0 0 0 0 0 0 0 0 0 0 0 0 0 0 0 0 0 0 0 0
0 0 0 0 0 0 0 0 0 0 0 0 0 0 0 0 0 0 0 0 0 0 0 0 0 0 0 0 0 0 0 0 0 0 0 0 0 0 0 0 0 0 0 0 0 0 0 0 0 0 0 1 0 0 0 0 0 0 0 0 0 0 0 0 0
0 0 0 0 0 0 0 0 0 0 0 0 0 0 0 0 0 0 0 0 0 0 0 0 0 0 0 0 0 0 0 0 0 0 0 0 0 0 0 0 0 0 0 0 0 0 0 0 0 0 0 0 0 0 0 0 0 0 0 0 0 0 0 0 0
0 0 0 0 0 0 0 0 0 0 0 0 0 0 0 0;
Estimate "Wisp1 Old Slope" Log_Total_GSH 1 Log_Total_GSH*TwoGroups 1 0 Log_Total_GSH*Protein 0 0 0 0 0 0 0 0 0 0 0 0 0 0 0 0 0 0
0 0 0 0 0 0 0 0 0 0 0 0 0 0 0 0 0 0 0 0 0 0 0 0 0 0 0 0 0 0 0 0 0 0 0 0 0 0 0 0 0 0 0 0 0 0 0 0 0 0 0 0 0 0 0 0 0 0 0 0 0 0 0 0 0
0 0 0 0 0 0 0 1 0 Log_Total_GSH*TwoGroups*Protein  0 0 0 0 0 0 0 0 0 0 0 0 0 0 0 0 0 0 0 0 0 0 0 0 0 0 0 0 0 0 0 0 0 0 0 0 0 0 0
0 0 0 0 0 0 0 0 0 0 0 0 0 0 0 0 0 0 0 0 0 0 0 0 0 0 0 0 0 0 0 0 0 0 0 0 0 0 0 0 0 0 0 0 0 0 0 0 0 0 0 1 0 0 0 0 0 0 0 0 0 0 0 0 0
0 0 0 0 0 0 0 0 0 0 0 0 0 0 0 0 0 0 0 0 0 0 0 0 0 0 0 0 0 0 0 0 0 0 0 0 0 0 0 0 0 0 0 0 0 0 0 0 0 0 0 0 0 0 0 0 0 0 0 0 0 0 0 0 0
0 0 0 0 0 0 0 0 0 0 0 0 0 0 0;
Estimate "Yes1 Old Slope" Log_Total_GSH 1 Log_Total_GSH*TwoGroups 1 0 Log_Total_GSH*Protein 0 0 0 0 0 0 0 0 0 0 0 0 0 0 0 0 0 0 0
0 0 0 0 0 0 0 0 0 0 0 0 0 0 0 0 0 0 0 0 0 0 0 0 0 0 0 0 0 0 0 0 0 0 0 0 0 0 0 0 0 0 0 0 0 0 0 0 0 0 0 0 0 0 0 0 0 0 0 0 0 0 0 0 0
0 0 0 0 0 0 0 1 Log_Total_GSH*TwoGroups*Protein  0 0 0 0 0 0 0 0 0 0 0 0 0 0 0 0 0 0 0 0 0 0 0 0 0 0 0 0 0 0 0 0 0 0 0 0 0 0 0 0
0 0 0 0 0 0 0 0 0 0 0 0 0 0 0 0 0 0 0 0 0 0 0 0 0 0 0 0 0 0 0 0 0 0 0 0 0 0 0 0 0 0 0 0 0 0 0 0 0 0 0 1 0 0 0 0 0 0 0 0 0 0 0 0 0
0 0 0 0 0 0 0 0 0 0 0 0 0 0 0 0 0 0 0 0 0 0 0 0 0 0 0 0 0 0 0 0 0 0 0 0 0 0 0 0 0 0 0 0 0 0 0 0 0 0 0 0 0 0 0 0 0 0 0 0 0 0 0 0 0
0 0 0 0 0 0 0 0 0 0 0 0 0 0;
Estimate "Acvrl1 Young Slope" Log_Total_GSH 1 Log_Total_GSH*TwoGroups 0 1Log_Total_GSH*Protein 1 0 0 0 0 0 0 0 0 0 0 0 0 0 0 0 0
0 0 0 0 0 0 0 0 0 0 0 0 0 0 0 0 0 0 0 0 0 0 0 0 0 0 0 0 0 0 0 0 0 0 0 0 0 0 0 0 0 0 0 0 0 0 0 0 0 0 0 0 0 0 0 0 0 0 0 0 0 0 0 0 0
0 0 0 0 0 0 0 0 0 0 Log_Total_GSH*TwoGroups*Protein  0 0 0 0 0 0 0 0 0 0 0 0 0 0 0 0 0 0 0 0 0 0 0 0 0 0 0 0 0 0 0 0 0 0 0 0 0 0
0 0 0 0 0 0 0 0 0 0 0 0 0 0 0 0 0 0 0 0 0 0 0 0 0 0 0 0 0 0 0 0 0 0 0 0 0 0 0 0 0 0 0 0 0 0 0 0 0 0 0 0 0 0 1 0 0 0 0 0 0 0 0 0 0
0 0 0 0 0 0 0 0 0 0 0 0 0 0 0 0 0 0 0 0 0 0 0 0 0 0 0 0 0 0 0 0 0 0 0 0 0 0 0 0 0 0 0 0 0 0 0 0 0 0 0 0 0 0 0 0 0 0 0 0 0 0 0 0 0
0 0 0 0 0 0 0 0 0 0 0 0 0 0 0 0;
Estimate "Adam23 Young Slope" Log_Total_GSH 1 Log_Total_GSH*TwoGroups 0 1Log_Total_GSH*Protein 0 1 0 0 0 0 0 0 0 0 0 0 0 0 0 0 0
0 0 0 0 0 0 0 0 0 0 0 0 0 0 0 0 0 0 0 0 0 0 0 0 0 0 0 0 0 0 0 0 0 0 0 0 0 0 0 0 0 0 0 0 0 0 0 0 0 0 0 0 0 0 0 0 0 0 0 0 0 0 0 0 0
0 0 0 0 0 0 0 0 0 0 Log_Total_GSH*TwoGroups*Protein  0 0 0 0 0 0 0 0 0 0 0 0 0 0 0 0 0 0 0 0 0 0 0 0 0 0 0 0 0 0 0 0 0 0 0 0 0 0
0 0 0 0 0 0 0 0 0 0 0 0 0 0 0 0 0 0 0 0 0 0 0 0 0 0 0 0 0 0 0 0 0 0 0 0 0 0 0 0 0 0 0 0 0 0 0 0 0 0 0 0 0 0 0 1 0 0 0 0 0 0 0 0 0
0 0 0 0 0 0 0 0 0 0 0 0 0 0 0 0 0 0 0 0 0 0 0 0 0 0 0 0 0 0 0 0 0 0 0 0 0 0 0 0 0 0 0 0 0 0 0 0 0 0 0 0 0 0 0 0 0 0 0 0 0 0 0 0 0
0 0 0 0 0 0 0 0 0 0 0 0 0 0 0 0;
Estimate "Ahr Young Slope" Log_Total_GSH 1 Log_Total_GSH*TwoGroups 0 1Log_Total_GSH*Protein 0 0 1 0 0 0 0 0 0 0 0 0 0 0 0 0 0 0 0
0 0 0 0 0 0 0 0 0 0 0 0 0 0 0 0 0 0 0 0 0 0 0 0 0 0 0 0 0 0 0 0 0 0 0 0 0 0 0 0 0 0 0 0 0 0 0 0 0 0 0 0 0 0 0 0 0 0 0 0 0 0 0 0 0
0 0 0 0 0 0 0 0 Log_Total_GSH*TwoGroups*Protein  0 0 0 0 0 0 0 0 0 0 0 0 0 0 0 0 0 0 0 0 0 0 0 0 0 0 0 0 0 0 0 0 0 0 0 0 0 0 0 0
```

```
0 0 0 0 0 0 0 0 0 0 0 0 0 0 0 0 0 0 0 0 0 0 0 0 0 0 0 0 0 0 0 0 0 0 0 0 0 0 0 0 0 0 0 0 0 0 0 0 0 0 0 0 0 0 1 0 0 0 0 0 0 0 0 0 0
0 0 0 0 0 0 0 0 0 0 0 0 0 0 0 0 0 0 0 0 0 0 0 0 0 0 0 0 0 0 0 0 0 0 0 0 0 0 0 0 0 0 0 0 0 0 0 0 0 0 0 0 0 0 0 0 0 0 0 0 0 0 0 0 0
0 0 0 0 0 0 0 0 0 0 0 0 0 0;
Estimate "Apbb1ip Young Slope" Log_Total_GSH 1 Log_Total_GSH*TwoGroups 0 1Log_Total_GSH*Protein 0 0 0 1 0 0 0 0 0 0 0 0 0 0 0 0 0
0 0 0 0 0 0 0 0 0 0 0 0 0 0 0 0 0 0 0 0 0 0 0 0 0 0 0 0 0 0 0 0 0 0 0 0 0 0 0 0 0 0 0 0 0 0 0 0 0 0 0 0 0 0 0 0 0 0 0 0 0 0 0 0 0
0 0 0 0 0 0 0 0 0 0 Log_Total_GSH*TwoGroups*Protein  0 0 0 0 0 0 0 0 0 0 0 0 0 0 0 0 0 0 0 0 0 0 0 0 0 0 0 0 0 0 0 0 0 0 0 0 0 0
0 0 0 0 0 0 0 0 0 0 0 0 0 0 0 0 0 0 0 0 0 0 0 0 0 0 0 0 0 0 0 0 0 0 0 0 0 0 0 0 0 0 0 0 0 0 0 0 0 0 0 0 0 0 0 0 0 1 0 0 0 0 0 0 0
0 0 0 0 0 0 0 0 0 0 0 0 0 0 0 0 0 0 0 0 0 0 0 0 0 0 0 0 0 0 0 0 0 0 0 0 0 0 0 0 0 0 0 0 0 0 0 0 0 0 0 0 0 0 0 0 0 0 0 0 0 0 0 0 0
0 0 0 0 0 0 0 0 0 0 0 0 0 0 0 0;
Estimate "Axin1 Young Slope" Log_Total_GSH 1 Log_Total_GSH*TwoGroups 0 1Log_Total_GSH*Protein 0 0 0 0 1 0 0 0 0 0 0 0 0 0 0 0 0 0
0 0 0 0 0 0 0 0 0 0 0 0 0 0 0 0 0 0 0 0 0 0 0 0 0 0 0 0 0 0 0 0 0 0 0 0 0 0 0 0 0 0 0 0 0 0 0 0 0 0 0 0 0 0 0 0 0 0 0 0 0 0 0 0 0
0 0 0 0 0 0 0 0 0 Log_Total_GSH*TwoGroups*Protein  0 0 0 0 0 0 0 0 0 0 0 0 0 0 0 0 0 0 0 0 0 0 0 0 0 0 0 0 0 0 0 0 0 0 0 0 0 0 0
0 0 0 0 0 0 0 0 0 0 0 0 0 0 0 0 0 0 0 0 0 0 0 0 0 0 0 0 0 0 0 0 0 0 0 0 0 0 0 0 0 0 0 0 0 0 0 0 0 0 0 0 0 0 0 0 0 1 0 0 0 0 0 0 0
0 0 0 0 0 0 0 0 0 0 0 0 0 0 0 0 0 0 0 0 0 0 0 0 0 0 0 0 0 0 0 0 0 0 0 0 0 0 0 0 0 0 0 0 0 0 0 0 0 0 0 0 0 0 0 0 0 0 0 0 0 0 0 0 0
0 0 0 0 0 0 0 0 0 0 0 0 0 0 0;
Estimate "Ca13 Young Slope" Log_Total_GSH 1 Log_Total_GSH*TwoGroups 0 1Log_Total_GSH*Protein 0 0 0 0 0 1 0 0 0 0 0 0 0 0 0 0 0 0
0 0 0 0 0 0 0 0 0 0 0 0 0 0 0 0 0 0 0 0 0 0 0 0 0 0 0 0 0 0 0 0 0 0 0 0 0 0 0 0 0 0 0 0 0 0 0 0 0 0 0 0 0 0 0 0 0 0 0 0 0 0 0 0 0
0 0 0 0 0 0 0 0 0 Log_Total_GSH*TwoGroups*Protein  0 0 0 0 0 0 0 0 0 0 0 0 0 0 0 0 0 0 0 0 0 0 0 0 0 0 0 0 0 0 0 0 0 0 0 0 0 0 0
0 0 0 0 0 0 0 0 0 0 0 0 0 0 0 0 0 0 0 0 0 0 0 0 0 0 0 0 0 0 0 0 0 0 0 0 0 0 0 0 0 0 0 0 0 0 0 0 0 0 0 0 0 0 0 0 0 0 1 0 0 0 0 0 0
0 0 0 0 0 0 0 0 0 0 0 0 0 0 0 0 0 0 0 0 0 0 0 0 0 0 0 0 0 0 0 0 0 0 0 0 0 0 0 0 0 0 0 0 0 0 0 0 0 0 0 0 0 0 0 0 0 0 0 0 0 0 0 0 0
0 0 0 0 0 0 0 0 0 0 0 0 0 0 0;
Estimate "Cant1 Young Slope" Log_Total_GSH 1 Log_Total_GSH*TwoGroups 0 1Log_Total_GSH*Protein 0 0 0 0 0 0 1 0 0 0 0 0 0 0 0 0 0 0
0 0 0 0 0 0 0 0 0 0 0 0 0 0 0 0 0 0 0 0 0 0 0 0 0 0 0 0 0 0 0 0 0 0 0 0 0 0 0 0 0 0 0 0 0 0 0 0 0 0 0 0 0 0 0 0 0 0 0 0 0 0 0 0 0
0 0 0 0 0 0 0 0 0 Log_Total_GSH*TwoGroups*Protein  0 0 0 0 0 0 0 0 0 0 0 0 0 0 0 0 0 0 0 0 0 0 0 0 0 0 0 0 0 0 0 0 0 0 0 0 0 0 0
0 0 0 0 0 0 0 0 0 0 0 0 0 0 0 0 0 0 0 0 0 0 0 0 0 0 0 0 0 0 0 0 0 0 0 0 0 0 0 0 0 0 0 0 0 0 0 0 0 0 0 0 0 0 0 0 0 0 0 1 0 0 0 0 0
0 0 0 0 0 0 0 0 0 0 0 0 0 0 0 0 0 0 0 0 0 0 0 0 0 0 0 0 0 0 0 0 0 0 0 0 0 0 0 0 0 0 0 0 0 0 0 0 0 0 0 0 0 0 0 0 0 0 0 0 0 0 0 0 0
0 0 0 0 0 0 0 0 0 0 0 0 0 0 0;
Estimate "Casp3 Young Slope" Log_Total_GSH 1 Log_Total_GSH*TwoGroups 0 1Log_Total_GSH*Protein 0 0 0 0 0 0 0 1 0 0 0 0 0 0 0 0 0 0
0 0 0 0 0 0 0 0 0 0 0 0 0 0 0 0 0 0 0 0 0 0 0 0 0 0 0 0 0 0 0 0 0 0 0 0 0 0 0 0 0 0 0 0 0 0 0 0 0 0 0 0 0 0 0 0 0 0 0 0 0 0 0 0 0
0 0 0 0 0 0 0 0 0 Log_Total_GSH*TwoGroups*Protein  0 0 0 0 0 0 0 0 0 0 0 0 0 0 0 0 0 0 0 0 0 0 0 0 0 0 0 0 0 0 0 0 0 0 0 0 0 0 0
0 0 0 0 0 0 0 0 0 0 0 0 0 0 0 0 0 0 0 0 0 0 0 0 0 0 0 0 0 0 0 0 0 0 0 0 0 0 0 0 0 0 0 0 0 0 0 0 0 0 0 0 0 0 0 0 0 0 0 0 1 0 0 0 0
0 0 0 0 0 0 0 0 0 0 0 0 0 0 0 0 0 0 0 0 0 0 0 0 0 0 0 0 0 0 0 0 0 0 0 0 0 0 0 0 0 0 0 0 0 0 0 0 0 0 0 0 0 0 0 0 0 0 0 0 0 0 0 0 0
0 0 0 0 0 0 0 0 0 0 0 0 0 0 0;
Estimate "Ccl2 Young Slope" Log_Total_GSH 1 Log_Total_GSH*TwoGroups 0 1Log_Total_GSH*Protein 0 0 0 0 0 0 0 0 1 0 0 0 0 0 0 0 0 0
0 0 0 0 0 0 0 0 0 0 0 0 0 0 0 0 0 0 0 0 0 0 0 0 0 0 0 0 0 0 0 0 0 0 0 0 0 0 0 0 0 0 0 0 0 0 0 0 0 0 0 0 0 0 0 0 0 0 0 0 0 0 0 0 0
0 0 0 0 0 0 0 0 0 Log_Total_GSH*TwoGroups*Protein  0 0 0 0 0 0 0 0 0 0 0 0 0 0 0 0 0 0 0 0 0 0 0 0 0 0 0 0 0 0 0 0 0 0 0 0 0 0 0
0 0 0 0 0 0 0 0 0 0 0 0 0 0 0 0 0 0 0 0 0 0 0 0 0 0 0 0 0 0 0 0 0 0 0 0 0 0 0 0 0 0 0 0 0 0 0 0 0 0 0 0 0 0 0 0 0 0 0 0 0 1 0 0 0
0 0 0 0 0 0 0 0 0 0 0 0 0 0 0 0 0 0 0 0 0 0 0 0 0 0 0 0 0 0 0 0 0 0 0 0 0 0 0 0 0 0 0 0 0 0 0 0 0 0 0 0 0 0 0 0 0 0 0 0 0 0 0 0 0
0 0 0 0 0 0 0 0 0 0 0 0 0 0 0;
Estimate "Ccl20 Young Slope" Log_Total_GSH 1 Log_Total_GSH*TwoGroups 0 1Log_Total_GSH*Protein 0 0 0 0 0 0 0 0 0 1 0 0 0 0 0 0 0 0
0 0 0 0 0 0 0 0 0 0 0 0 0 0 0 0 0 0 0 0 0 0 0 0 0 0 0 0 0 0 0 0 0 0 0 0 0 0 0 0 0 0 0 0 0 0 0 0 0 0 0 0 0 0 0 0 0 0 0 0 0 0 0 0 0
0 0 0 0 0 0 0 0 0 Log_Total_GSH*TwoGroups*Protein  0 0 0 0 0 0 0 0 0 0 0 0 0 0 0 0 0 0 0 0 0 0 0 0 0 0 0 0 0 0 0 0 0 0 0 0 0 0 0
0 0 0 0 0 0 0 0 0 0 0 0 0 0 0 0 0 0 0 0 0 0 0 0 0 0 0 0 0 0 0 0 0 0 0 0 0 0 0 0 0 0 0 0 0 0 0 0 0 0 0 0 0 0 0 0 0 0 0 0 0 0 1 0 0
0 0 0 0 0 0 0 0 0 0 0 0 0 0 0 0 0 0 0 0 0 0 0 0 0 0 0 0 0 0 0 0 0 0 0 0 0 0 0 0 0 0 0 0 0 0 0 0 0 0 0 0 0 0 0 0 0 0 0 0 0 0 0 0 0
0 0 0 0 0 0 0 0 0 0 0 0 0 0 0;
Estimate "Ccl3 Young Slope" Log_Total_GSH 1 Log_Total_GSH*TwoGroups 0 1Log_Total_GSH*Protein 0 0 0 0 0 0 0 0 0 0 1 0 0 0 0 0 0 0
0 0 0 0 0 0 0 0 0 0 0 0 0 0 0 0 0 0 0 0 0 0 0 0 0 0 0 0 0 0 0 0 0 0 0 0 0 0 0 0 0 0 0 0 0 0 0 0 0 0 0 0 0 0 0 0 0 0 0 0 0 0 0 0 0
0 0 0 0 0 0 0 0 0 Log_Total_GSH*TwoGroups*Protein  0 0 0 0 0 0 0 0 0 0 0 0 0 0 0 0 0 0 0 0 0 0 0 0 0 0 0 0 0 0 0 0 0 0 0 0 0 0 0
0 0 0 0 0 0 0 0 0 0 0 0 0 0 0 0 0 0 0 0 0 0 0 0 0 0 0 0 0 0 0 0 0 0 0 0 0 0 0 0 0 0 0 0 0 0 0 0 0 0 0 0 0 0 0 0 0 0 0 0 0 0 0 1 0
0 0 0 0 0 0 0 0 0 0 0 0 0 0 0 0 0 0 0 0 0 0 0 0 0 0 0 0 0 0 0 0 0 0 0 0 0 0 0 0 0 0 0 0 0 0 0 0 0 0 0 0 0 0 0 0 0 0 0 0 0 0 0 0 0
0 0 0 0 0 0 0 0 0 0 0 0 0 0 0;
Estimate "Ccl5 Young Slope" Log_Total_GSH 1 Log_Total_GSH*TwoGroups 0 1Log_Total_GSH*Protein 0 0 0 0 0 0 0 0 0 0 0 1 0 0 0 0 0 0
0 0 0 0 0 0 0 0 0 0 0 0 0 0 0 0 0 0 0 0 0 0 0 0 0 0 0 0 0 0 0 0 0 0 0 0 0 0 0 0 0 0 0 0 0 0 0 0 0 0 0 0 0 0 0 0 0 0 0 0 0 0 0 0 0
0 0 0 0 0 0 0 0 0 Log_Total_GSH*TwoGroups*Protein  0 0 0 0 0 0 0 0 0 0 0 0 0 0 0 0 0 0 0 0 0 0 0 0 0 0 0 0 0 0 0 0 0 0 0 0 0 0 0
0 0 0 0 0 0 0 0 0 0 0 0 0 0 0 0 0 0 0 0 0 0 0 0 0 0 0 0 0 0 0 0 0 0 0 0 0 0 0 0 0 0 0 0 0 0 0 0 0 0 0 0 0 0 0 0 0 0 0 0 0 0 0 0 1
0 0 0 0 0 0 0 0 0 0 0 0 0 0 0 0 0 0 0 0 0 0 0 0 0 0 0 0 0 0 0 0 0 0 0 0 0 0 0 0 0 0 0 0 0 0 0 0 0 0 0 0 0 0 0 0 0 0 0 0 0 0 0 0 0
0 0 0 0 0 0 0 0 0 0 0 0 0 0 0;
Estimate "Cdh6 Young Slope" Log_Total_GSH 1 Log_Total_GSH*TwoGroups 0 1Log_Total_GSH*Protein 0 0 0 0 0 0 0 0 0 0 0 0 1 0 0 0 0 0
0 0 0 0 0 0 0 0 0 0 0 0 0 0 0 0 0 0 0 0 0 0 0 0 0 0 0 0 0 0 0 0 0 0 0 0 0 0 0 0 0 0 0 0 0 0 0 0 0 0 0 0 0 0 0 0 0 0 0 0 0 0 0 0 0
0 0 0 0 0 0 0 0 0 Log_Total_GSH*TwoGroups*Protein  0 0 0 0 0 0 0 0 0 0 0 0 0 0 0 0 0 0 0 0 0 0 0 0 0 0 0 0 0 0 0 0 0 0 0 0 0 0 0
0 0 0 0 0 0 0 0 0 0 0 0 0 0 0 0 0 0 0 0 0 0 0 0 0 0 0 0 0 0 0 0 0 0 0 0 0 0 0 0 0 0 0 0 0 0 0 0 0 0 0 0 0 0 0 0 0 0 0 0 0 0 0 0 0
1 0 0 0 0 0 0 0 0 0 0 0 0 0 0 0 0 0 0 0 0 0 0 0 0 0 0 0 0 0 0 0 0 0 0 0 0 0 0 0 0 0 0 0 0 0 0 0 0 0 0 0 0 0 0 0 0 0 0 0 0 0 0 0 0
0 0 0 0 0 0 0 0 0 0 0 0 0 0 0;
Estimate "Clmp Young Slope" Log_Total_GSH 1 Log_Total_GSH*TwoGroups 0 1Log_Total_GSH*Protein 0 0 0 0 0 0 0 0 0 0 0 0 0 1 0 0 0 0
0 0 0 0 0 0 0 0 0 0 0 0 0 0 0 0 0 0 0 0 0 0 0 0 0 0 0 0 0 0 0 0 0 0 0 0 0 0 0 0 0 0 0 0 0 0 0 0 0 0 0 0 0 0 0 0 0 0 0 0 0 0 0 0 0
0 0 0 0 0 0 0 0 0 Log_Total_GSH*TwoGroups*Protein  0 0 0 0 0 0 0 0 0 0 0 0 0 0 0 0 0 0 0 0 0 0 0 0 0 0 0 0 0 0 0 0 0 0 0 0 0 0 0
0 0 0 0 0 0 0 0 0 0 0 0 0 0 0 0 0 0 0 0 0 0 0 0 0 0 0 0 0 0 0 0 0 0 0 0 0 0 0 0 0 0 0 0 0 0 0 0 0 0 0 0 0 0 0 0 0 0 0 0 0 0 0 0 0
0 1 0 0 0 0 0 0 0 0 0 0 0 0 0 0 0 0 0 0 0 0 0 0 0 0 0 0 0 0 0 0 0 0 0 0 0 0 0 0 0 0 0 0 0 0 0 0 0 0 0 0 0 0 0 0 0 0 0 0 0 0 0 0 0
0 0 0 0 0 0 0 0 0 0 0 0 0 0 0;
Estimate "Clstn2 Young Slope" Log_Total_GSH 1 Log_Total_GSH*TwoGroups 0 1Log_Total_GSH*Protein 0 0 0 0 0 0 0 0 0 0 0 0 0 0 1 0 0
0 0 0 0 0 0 0 0 0 0 0 0 0 0 0 0 0 0 0 0 0 0 0 0 0 0 0 0 0 0 0 0 0 0 0 0 0 0 0 0 0 0 0 0 0 0 0 0 0 0 0 0 0 0 0 0 0 0 0 0 0 0 0 0 0
0 0 0 0 0 0 0 0 0 0 Log_Total_GSH*TwoGroups*Protein  0 0 0 0 0 0 0 0 0 0 0 0 0 0 0 0 0 0 0 0 0 0 0 0 0 0 0 0 0 0 0 0 0 0 0 0 0 0
0 0 0 0 0 0 0 0 0 0 0 0 0 0 0 0 0 0 0 0 0 0 0 0 0 0 0 0 0 0 0 0 0 0 0 0 0 0 0 0 0 0 0 0 0 0 0 0 0 0 0 0 0 0 0 0 0 0 0 0 0 0 0 0 0
0 0 0 1 0 0 0 0 0 0 0 0 0 0 0 0 0 0 0 0 0 0 0 0 0 0 0 0 0 0 0 0 0 0 0 0 0 0 0 0 0 0 0 0 0 0 0 0 0 0 0 0 0 0 0 0 0 0 0 0 0 0 0 0 0
0 0 0 0 0 0 0 0 0 0 0 0 0 0 0 0;
Estimate "Cntn1 Young Slope" Log_Total_GSH 1 Log_Total_GSH*TwoGroups 0 1Log_Total_GSH*Protein 0 0 0 0 0 0 0 0 0 0 0 0 0 0 0 1 0 0
0 0 0 0 0 0 0 0 0 0 0 0 0 0 0 0 0 0 0 0 0 0 0 0 0 0 0 0 0 0 0 0 0 0 0 0 0 0 0 0 0 0 0 0 0 0 0 0 0 0 0 0 0 0 0 0 0 0 0 0 0 0 0 0 0
0 0 0 0 0 0 0 0 0 Log_Total_GSH*TwoGroups*Protein  0 0 0 0 0 0 0 0 0 0 0 0 0 0 0 0 0 0 0 0 0 0 0 0 0 0 0 0 0 0 0 0 0 0 0 0 0 0 0
0 0 0 0 0 0 0 0 0 0 0 0 0 0 0 0 0 0 0 0 0 0 0 0 0 0 0 0 0 0 0 0 0 0 0 0 0 0 0 0 0 0 0 0 0 0 0 0 0 0 0 0 0 0 0 0 0 0 0 0 0 0 0 0 0
0 0 0 1 0 0 0 0 0 0 0 0 0 0 0 0 0 0 0 0 0 0 0 0 0 0 0 0 0 0 0 0 0 0 0 0 0 0 0 0 0 0 0 0 0 0 0 0 0 0 0 0 0 0 0 0 0 0 0 0 0 0 0 0 0
0 0 0 0 0 0 0 0 0 0 0 0 0 0 0;
Estimate "Cntn4 Young Slope" Log_Total_GSH 1 Log_Total_GSH*TwoGroups 0 1Log_Total_GSH*Protein 0 0 0 0 0 0 0 0 0 0 0 0 0 0 0 0 1 0
0 0 0 0 0 0 0 0 0 0 0 0 0 0 0 0 0 0 0 0 0 0 0 0 0 0 0 0 0 0 0 0 0 0 0 0 0 0 0 0 0 0 0 0 0 0 0 0 0 0 0 0 0 0 0 0 0 0 0 0 0 0 0 0 0
0 0 0 0 0 0 0 0 0 Log_Total_GSH*TwoGroups*Protein  0 0 0 0 0 0 0 0 0 0 0 0 0 0 0 0 0 0 0 0 0 0 0 0 0 0 0 0 0 0 0 0 0 0 0 0 0 0 0
0 0 0 0 0 0 0 0 0 0 0 0 0 0 0 0 0 0 0 0 0 0 0 0 0 0 0 0 0 0 0 0 0 0 0 0 0 0 0 0 0 0 0 0 0 0 0 0 0 0 0 0 0 0 0 0 0 0 0 0 0 0 0 0 0
0 0 0 0 1 0 0 0 0 0 0 0 0 0 0 0 0 0 0 0 0 0 0 0 0 0 0 0 0 0 0 0 0 0 0 0 0 0 0 0 0 0 0 0 0 0 0 0 0 0 0 0 0 0 0 0 0 0 0 0 0 0 0 0 0
0 0 0 0 0 0 0 0 0 0 0 0 0 0 0;
Estimate "Cpe Young Slope" Log_Total_GSH 1 Log_Total_GSH*TwoGroups 0 1Log_Total_GSH*Protein 0 0 0 0 0 0 0 0 0 0 0 0 0 0 0 0 0 1 0
0 0 0 0 0 0 0 0 0 0 0 0 0 0 0 0 0 0 0 0 0 0 0 0 0 0 0 0 0 0 0 0 0 0 0 0 0 0 0 0 0 0 0 0 0 0 0 0 0 0 0 0 0 0 0 0 0 0 0 0 0 0 0 0 0
0 0 0 0 0 0 0 0 Log_Total_GSH*TwoGroups*Protein  0 0 0 0 0 0 0 0 0 0 0 0 0 0 0 0 0 0 0 0 0 0 0 0 0 0 0 0 0 0 0 0 0 0 0 0 0 0 0 0
0 0 0 0 0 0 0 0 0 0 0 0 0 0 0 0 0 0 0 0 0 0 0 0 0 0 0 0 0 0 0 0 0 0 0 0 0 0 0 0 0 0 0 0 0 0 0 0 0 0 0 0 0 0 0 0 0 0 0 0 0 0 0 0 0
0 0 0 0 1 0 0 0 0 0 0 0 0 0 0 0 0 0 0 0 0 0 0 0 0 0 0 0 0 0 0 0 0 0 0 0 0 0 0 0 0 0 0 0 0 0 0 0 0 0 0 0 0 0 0 0 0 0 0 0 0 0 0 0 0
0 0 0 0 0 0 0 0 0 0 0 0 0 0;
Estimate "Crim1 Young Slope" Log_Total_GSH 1 Log_Total_GSH*TwoGroups 0 1Log_Total_GSH*Protein 0 0 0 0 0 0 0 0 0 0 0 0 0 0 0 0 0 0
1 0 0 0 0 0 0 0 0 0 0 0 0 0 0 0 0 0 0 0 0 0 0 0 0 0 0 0 0 0 0 0 0 0 0 0 0 0 0 0 0 0 0 0 0 0 0 0 0 0 0 0 0 0 0 0 0 0 0 0 0 0 0 0 0
```

```
0 0 0 0 0 0 0 0 0 Log_Total_GSH*TwoGroups*Protein  0 0 0 0 0 0 0 0 0 0 0 0 0 0 0 0 0 0 0 0 0 0 0 0 0 0 0 0 0 0 0 0 0 0 0 0 0 0 0
0 0 0 0 0 0 0 0 0 0 0 0 0 0 0 0 0 0 0 0 0 0 0 0 0 0 0 0 0 0 0 0 0 0 0 0 0 0 0 0 0 0 0 0 0 0 0 0 0 0 0 0 0 0 0 0 0 0 0 0 0 0 0 0 0
0 0 0 0 0 0 1 0 0 0 0 0 0 0 0 0 0 0 0 0 0 0 0 0 0 0 0 0 0 0 0 0 0 0 0 0 0 0 0 0 0 0 0 0 0 0 0 0 0 0 0 0 0 0 0 0 0 0 0 0 0 0 0 0 0
0 0 0 0 0 0 0 0 0 0 0 0 0 0 0;
Estimate "Csf2 Young Slope" Log_Total_GSH 1 Log_Total_GSH*TwoGroups 0 1Log_Total_GSH*Protein 0 0 0 0 0 0 0 0 0 0 0 0 0 0 0 0 0 0
0 1 0 0 0 0 0 0 0 0 0 0 0 0 0 0 0 0 0 0 0 0 0 0 0 0 0 0 0 0 0 0 0 0 0 0 0 0 0 0 0 0 0 0 0 0 0 0 0 0 0 0 0 0 0 0 0 0 0 0 0 0 0 0 0
0 0 0 0 0 0 0 0 0 Log_Total_GSH*TwoGroups*Protein  0 0 0 0 0 0 0 0 0 0 0 0 0 0 0 0 0 0 0 0 0 0 0 0 0 0 0 0 0 0 0 0 0 0 0 0 0 0 0
0 0 0 0 0 0 0 0 0 0 0 0 0 0 0 0 0 0 0 0 0 0 0 0 0 0 0 0 0 0 0 0 0 0 0 0 0 0 0 0 0 0 0 0 0 0 0 0 0 0 0 0 0 0 0 0 0 0 0 0 0 0 0 0 0
0 0 0 0 0 0 0 1 0 0 0 0 0 0 0 0 0 0 0 0 0 0 0 0 0 0 0 0 0 0 0 0 0 0 0 0 0 0 0 0 0 0 0 0 0 0 0 0 0 0 0 0 0 0 0 0 0 0 0 0 0 0 0 0 0
0 0 0 0 0 0 0 0 0 0 0 0 0 0 0;
Estimate "Cxcl1 Young Slope" Log_Total_GSH 1 Log_Total_GSH*TwoGroups 0 1Log_Total_GSH*Protein 0 0 0 0 0 0 0 0 0 0 0 0 0 0 0 0 0 0
0 0 1 0 0 0 0 0 0 0 0 0 0 0 0 0 0 0 0 0 0 0 0 0 0 0 0 0 0 0 0 0 0 0 0 0 0 0 0 0 0 0 0 0 0 0 0 0 0 0 0 0 0 0 0 0 0 0 0 0 0 0 0 0 0
0 0 0 0 0 0 0 0 0 Log_Total_GSH*TwoGroups*Protein  0 0 0 0 0 0 0 0 0 0 0 0 0 0 0 0 0 0 0 0 0 0 0 0 0 0 0 0 0 0 0 0 0 0 0 0 0 0 0
0 0 0 0 0 0 0 0 0 0 0 0 0 0 0 0 0 0 0 0 0 0 0 0 0 0 0 0 0 0 0 0 0 0 0 0 0 0 0 0 0 0 0 0 0 0 0 0 0 0 0 0 0 0 0 0 0 0 0 0 0 0 0 0 0
0 0 0 0 0 0 0 0 1 0 0 0 0 0 0 0 0 0 0 0 0 0 0 0 0 0 0 0 0 0 0 0 0 0 0 0 0 0 0 0 0 0 0 0 0 0 0 0 0 0 0 0 0 0 0 0 0 0 0 0 0 0 0 0 0
0 0 0 0 0 0 0 0 0 0 0 0 0 0 0;
Estimate "Cxcl9 Young Slope" Log_Total_GSH 1 Log_Total_GSH*TwoGroups 0 1Log_Total_GSH*Protein 0 0 0 0 0 0 0 0 0 0 0 0 0 0 0 0 0 0
0 0 0 1 0 0 0 0 0 0 0 0 0 0 0 0 0 0 0 0 0 0 0 0 0 0 0 0 0 0 0 0 0 0 0 0 0 0 0 0 0 0 0 0 0 0 0 0 0 0 0 0 0 0 0 0 0 0 0 0 0 0 0 0 0
0 0 0 0 0 0 0 0 0 Log_Total_GSH*TwoGroups*Protein  0 0 0 0 0 0 0 0 0 0 0 0 0 0 0 0 0 0 0 0 0 0 0 0 0 0 0 0 0 0 0 0 0 0 0 0 0 0 0
0 0 0 0 0 0 0 0 0 0 0 0 0 0 0 0 0 0 0 0 0 0 0 0 0 0 0 0 0 0 0 0 0 0 0 0 0 0 0 0 0 0 0 0 0 0 0 0 0 0 0 0 0 0 0 0 0 0 0 0 0 0 0 0 0
0 0 0 0 0 0 0 0 0 1 0 0 0 0 0 0 0 0 0 0 0 0 0 0 0 0 0 0 0 0 0 0 0 0 0 0 0 0 0 0 0 0 0 0 0 0 0 0 0 0 0 0 0 0 0 0 0 0 0 0 0 0 0 0 0
0 0 0 0 0 0 0 0 0 0 0 0 0 0 0;
Estimate "Cyr61 Young Slope" Log_Total_GSH 1 Log_Total_GSH*TwoGroups 0 1Log_Total_GSH*Protein 0 0 0 0 0 0 0 0 0 0 0 0 0 0 0 0 0 0
0 0 0 0 1 0 0 0 0 0 0 0 0 0 0 0 0 0 0 0 0 0 0 0 0 0 0 0 0 0 0 0 0 0 0 0 0 0 0 0 0 0 0 0 0 0 0 0 0 0 0 0 0 0 0 0 0 0 0 0 0 0 0 0 0
0 0 0 0 0 0 0 0 0 Log_Total_GSH*TwoGroups*Protein  0 0 0 0 0 0 0 0 0 0 0 0 0 0 0 0 0 0 0 0 0 0 0 0 0 0 0 0 0 0 0 0 0 0 0 0 0 0 0
0 0 0 0 0 0 0 0 0 0 0 0 0 0 0 0 0 0 0 0 0 0 0 0 0 0 0 0 0 0 0 0 0 0 0 0 0 0 0 0 0 0 0 0 0 0 0 0 0 0 0 0 0 0 0 0 0 0 0 0 0 0 0 0 0
0 0 0 0 0 0 0 0 0 0 1 0 0 0 0 0 0 0 0 0 0 0 0 0 0 0 0 0 0 0 0 0 0 0 0 0 0 0 0 0 0 0 0 0 0 0 0 0 0 0 0 0 0 0 0 0 0 0 0 0 0 0 0 0 0
0 0 0 0 0 0 0 0 0 0 0 0 0 0 0;
Estimate "Dctn2 Young Slope" Log_Total_GSH 1 Log_Total_GSH*TwoGroups 0 1Log_Total_GSH*Protein 0 0 0 0 0 0 0 0 0 0 0 0 0 0 0 0 0 0
0 0 0 0 0 1 0 0 0 0 0 0 0 0 0 0 0 0 0 0 0 0 0 0 0 0 0 0 0 0 0 0 0 0 0 0 0 0 0 0 0 0 0 0 0 0 0 0 0 0 0 0 0 0 0 0 0 0 0 0 0 0 0 0 0
0 0 0 0 0 0 0 0 0 Log_Total_GSH*TwoGroups*Protein  0 0 0 0 0 0 0 0 0 0 0 0 0 0 0 0 0 0 0 0 0 0 0 0 0 0 0 0 0 0 0 0 0 0 0 0 0 0 0
0 0 0 0 0 0 0 0 0 0 0 0 0 0 0 0 0 0 0 0 0 0 0 0 0 0 0 0 0 0 0 0 0 0 0 0 0 0 0 0 0 0 0 0 0 0 0 0 0 0 0 0 0 0 0 0 0 0 0 0 0 0 0 0 0
0 0 0 0 0 0 0 0 0 0 0 1 0 0 0 0 0 0 0 0 0 0 0 0 0 0 0 0 0 0 0 0 0 0 0 0 0 0 0 0 0 0 0 0 0 0 0 0 0 0 0 0 0 0 0 0 0 0 0 0 0 0 0 0 0
0 0 0 0 0 0 0 0 0 0 0 0 0 0 0;
Estimate "Ddah1 Young Slope" Log_Total_GSH 1 Log_Total_GSH*TwoGroups 0 1Log_Total_GSH*Protein 0 0 0 0 0 0 0 0 0 0 0 0 0 0 0 0 0 0
0 0 0 0 0 0 1 0 0 0 0 0 0 0 0 0 0 0 0 0 0 0 0 0 0 0 0 0 0 0 0 0 0 0 0 0 0 0 0 0 0 0 0 0 0 0 0 0 0 0 0 0 0 0 0 0 0 0 0 0 0 0 0 0 0
0 0 0 0 0 0 0 0 0 Log_Total_GSH*TwoGroups*Protein  0 0 0 0 0 0 0 0 0 0 0 0 0 0 0 0 0 0 0 0 0 0 0 0 0 0 0 0 0 0 0 0 0 0 0 0 0 0 0
0 0 0 0 0 0 0 0 0 0 0 0 0 0 0 0 0 0 0 0 0 0 0 0 0 0 0 0 0 0 0 0 0 0 0 0 0 0 0 0 0 0 0 0 0 0 0 0 0 0 0 0 0 0 0 0 0 0 0 0 0 0 0 0 0
0 0 0 0 0 0 0 0 0 0 0 0 1 0 0 0 0 0 0 0 0 0 0 0 0 0 0 0 0 0 0 0 0 0 0 0 0 0 0 0 0 0 0 0 0 0 0 0 0 0 0 0 0 0 0 0 0 0 0 0 0 0 0 0 0
0 0 0 0 0 0 0 0 0 0 0 0 0 0 0;
Estimate "Dlk1 Young Slope" Log_Total_GSH 1 Log_Total_GSH*TwoGroups 0 1Log_Total_GSH*Protein 0 0 0 0 0 0 0 0 0 0 0 0 0 0 0 0 0 0
0 0 0 0 0 0 0 1 0 0 0 0 0 0 0 0 0 0 0 0 0 0 0 0 0 0 0 0 0 0 0 0 0 0 0 0 0 0 0 0 0 0 0 0 0 0 0 0 0 0 0 0 0 0 0 0 0 0 0 0 0 0 0 0 0
0 0 0 0 0 0 0 0 0 Log_Total_GSH*TwoGroups*Protein  0 0 0 0 0 0 0 0 0 0 0 0 0 0 0 0 0 0 0 0 0 0 0 0 0 0 0 0 0 0 0 0 0 0 0 0 0 0 0
0 0 0 0 0 0 0 0 0 0 0 0 0 0 0 0 0 0 0 0 0 0 0 0 0 0 0 0 0 0 0 0 0 0 0 0 0 0 0 0 0 0 0 0 0 0 0 0 0 0 0 0 0 0 0 0 0 0 0 0 0 0 0 0 0
0 0 0 0 0 0 0 0 0 0 0 0 0 1 0 0 0 0 0 0 0 0 0 0 0 0 0 0 0 0 0 0 0 0 0 0 0 0 0 0 0 0 0 0 0 0 0 0 0 0 0 0 0 0 0 0 0 0 0 0 0 0 0 0 0
0 0 0 0 0 0 0 0 0 0 0 0 0 0 0;
Estimate "Dll1 Young Slope" Log_Total_GSH 1 Log_Total_GSH*TwoGroups 0 1Log_Total_GSH*Protein 0 0 0 0 0 0 0 0 0 0 0 0 0 0 0 0 0 0
0 0 0 0 0 0 0 0 1 0 0 0 0 0 0 0 0 0 0 0 0 0 0 0 0 0 0 0 0 0 0 0 0 0 0 0 0 0 0 0 0 0 0 0 0 0 0 0 0 0 0 0 0 0 0 0 0 0 0 0 0 0 0 0 0
0 0 0 0 0 0 0 0 0 Log_Total_GSH*TwoGroups*Protein  0 0 0 0 0 0 0 0 0 0 0 0 0 0 0 0 0 0 0 0 0 0 0 0 0 0 0 0 0 0 0 0 0 0 0 0 0 0 0
0 0 0 0 0 0 0 0 0 0 0 0 0 0 0 0 0 0 0 0 0 0 0 0 0 0 0 0 0 0 0 0 0 0 0 0 0 0 0 0 0 0 0 0 0 0 0 0 0 0 0 0 0 0 0 0 0 0 0 0 0 0 0 0 0
0 0 0 0 0 0 0 0 0 0 0 0 0 0 1 0 0 0 0 0 0 0 0 0 0 0 0 0 0 0 0 0 0 0 0 0 0 0 0 0 0 0 0 0 0 0 0 0 0 0 0 0 0 0 0 0 0 0 0 0 0 0 0 0 0
0 0 0 0 0 0 0 0 0 0 0 0 0 0 0;
Estimate "Eda2r Young Slope" Log_Total_GSH 1 Log_Total_GSH*TwoGroups 0 1Log_Total_GSH*Protein 0 0 0 0 0 0 0 0 0 0 0 0 0 0 0 0 0 0
0 0 0 0 0 0 0 0 0 1 0 0 0 0 0 0 0 0 0 0 0 0 0 0 0 0 0 0 0 0 0 0 0 0 0 0 0 0 0 0 0 0 0 0 0 0 0 0 0 0 0 0 0 0 0 0 0 0 0 0 0 0 0 0 0
0 0 0 0 0 0 0 0 0 Log_Total_GSH*TwoGroups*Protein  0 0 0 0 0 0 0 0 0 0 0 0 0 0 0 0 0 0 0 0 0 0 0 0 0 0 0 0 0 0 0 0 0 0 0 0 0 0 0
0 0 0 0 0 0 0 0 0 0 0 0 0 0 0 0 0 0 0 0 0 0 0 0 0 0 0 0 0 0 0 0 0 0 0 0 0 0 0 0 0 0 0 0 0 0 0 0 0 0 0 0 0 0 0 0 0 0 0 0 0 0 0 0 0
0 0 0 0 0 0 0 0 0 0 0 0 0 0 0 1 0 0 0 0 0 0 0 0 0 0 0 0 0 0 0 0 0 0 0 0 0 0 0 0 0 0 0 0 0 0 0 0 0 0 0 0 0 0 0 0 0 0 0 0 0 0 0 0 0
0 0 0 0 0 0 0 0 0 0 0 0 0 0 0;
Estimate "Eno2 Young Slope" Log_Total_GSH 1 Log_Total_GSH*TwoGroups 0 1Log_Total_GSH*Protein 0 0 0 0 0 0 0 0 0 0 0 0 0 0 0 0 0 0
0 0 0 0 0 0 0 0 0 0 1 0 0 0 0 0 0 0 0 0 0 0 0 0 0 0 0 0 0 0 0 0 0 0 0 0 0 0 0 0 0 0 0 0 0 0 0 0 0 0 0 0 0 0 0 0 0 0 0 0 0 0 0 0 0
0 0 0 0 0 0 0 0 0 Log_Total_GSH*TwoGroups*Protein  0 0 0 0 0 0 0 0 0 0 0 0 0 0 0 0 0 0 0 0 0 0 0 0 0 0 0 0 0 0 0 0 0 0 0 0 0 0 0
0 0 0 0 0 0 0 0 0 0 0 0 0 0 0 0 0 0 0 0 0 0 0 0 0 0 0 0 0 0 0 0 0 0 0 0 0 0 0 0 0 0 0 0 0 0 0 0 0 0 0 0 0 0 0 0 0 0 0 0 0 0 0 0 0
0 0 0 0 0 0 0 0 0 0 0 0 0 0 0 0 1 0 0 0 0 0 0 0 0 0 0 0 0 0 0 0 0 0 0 0 0 0 0 0 0 0 0 0 0 0 0 0 0 0 0 0 0 0 0 0 0 0 0 0 0 0 0 0 0
0 0 0 0 0 0 0 0 0 0 0 0 0 0 0;
Estimate "Epcam Young Slope" Log_Total_GSH 1 Log_Total_GSH*TwoGroups 0 1Log_Total_GSH*Protein 0 0 0 0 0 0 0 0 0 0 0 0 0 0 0 0 0 0
0 0 0 0 0 0 0 0 0 0 0 1 0 0 0 0 0 0 0 0 0 0 0 0 0 0 0 0 0 0 0 0 0 0 0 0 0 0 0 0 0 0 0 0 0 0 0 0 0 0 0 0 0 0 0 0 0 0 0 0 0 0 0 0 0
0 0 0 0 0 0 0 0 0 Log_Total_GSH*TwoGroups*Protein  0 0 0 0 0 0 0 0 0 0 0 0 0 0 0 0 0 0 0 0 0 0 0 0 0 0 0 0 0 0 0 0 0 0 0 0 0 0 0
0 0 0 0 0 0 0 0 0 0 0 0 0 0 0 0 0 0 0 0 0 0 0 0 0 0 0 0 0 0 0 0 0 0 0 0 0 0 0 0 0 0 0 0 0 0 0 0 0 0 0 0 0 0 0 0 0 0 0 0 0 0 0 0 0
0 0 0 0 0 0 0 0 0 0 0 0 0 0 0 0 0 1 0 0 0 0 0 0 0 0 0 0 0 0 0 0 0 0 0 0 0 0 0 0 0 0 0 0 0 0 0 0 0 0 0 0 0 0 0 0 0 0 0 0 0 0 0 0 0
0 0 0 0 0 0 0 0 0 0 0 0 0 0 0;
Estimate "Epo Young Slope" Log_Total_GSH 1 Log_Total_GSH*TwoGroups 0 1Log_Total_GSH*Protein 0 0 0 0 0 0 0 0 0 0 0 0 0 0 0 0 0 0 0
0 0 0 0 0 0 0 0 0 0 0 1 0 0 0 0 0 0 0 0 0 0 0 0 0 0 0 0 0 0 0 0 0 0 0 0 0 0 0 0 0 0 0 0 0 0 0 0 0 0 0 0 0 0 0 0 0 0 0 0 0 0 0 0 0
0 0 0 0 0 0 0 0 Log_Total_GSH*TwoGroups*Protein  0 0 0 0 0 0 0 0 0 0 0 0 0 0 0 0 0 0 0 0 0 0 0 0 0 0 0 0 0 0 0 0 0 0 0 0 0 0 0 0
0 0 0 0 0 0 0 0 0 0 0 0 0 0 0 0 0 0 0 0 0 0 0 0 0 0 0 0 0 0 0 0 0 0 0 0 0 0 0 0 0 0 0 0 0 0 0 0 0 0 0 0 0 0 0 0 0 0 0 0 0 0 0 0 0
0 0 0 0 0 0 0 0 0 0 0 0 0 0 0 0 0 1 0 0 0 0 0 0 0 0 0 0 0 0 0 0 0 0 0 0 0 0 0 0 0 0 0 0 0 0 0 0 0 0 0 0 0 0 0 0 0 0 0 0 0 0 0 0 0
0 0 0 0 0 0 0 0 0 0 0 0 0 0;
Estimate "Erbb4 Young Slope" Log_Total_GSH 1 Log_Total_GSH*TwoGroups 0 1Log_Total_GSH*Protein 0 0 0 0 0 0 0 0 0 0 0 0 0 0 0 0 0 0
0 0 0 0 0 0 0 0 0 0 0 0 0 1 0 0 0 0 0 0 0 0 0 0 0 0 0 0 0 0 0 0 0 0 0 0 0 0 0 0 0 0 0 0 0 0 0 0 0 0 0 0 0 0 0 0 0 0 0 0 0 0 0 0 0
0 0 0 0 0 0 0 0 0 Log_Total_GSH*TwoGroups*Protein  0 0 0 0 0 0 0 0 0 0 0 0 0 0 0 0 0 0 0 0 0 0 0 0 0 0 0 0 0 0 0 0 0 0 0 0 0 0 0
0 0 0 0 0 0 0 0 0 0 0 0 0 0 0 0 0 0 0 0 0 0 0 0 0 0 0 0 0 0 0 0 0 0 0 0 0 0 0 0 0 0 0 0 0 0 0 0 0 0 0 0 0 0 0 0 0 0 0 0 0 0 0 0 0
0 0 0 0 0 0 0 0 0 0 0 0 0 0 0 0 0 0 0 1 0 0 0 0 0 0 0 0 0 0 0 0 0 0 0 0 0 0 0 0 0 0 0 0 0 0 0 0 0 0 0 0 0 0 0 0 0 0 0 0 0 0 0 0 0
0 0 0 0 0 0 0 0 0 0 0 0 0 0 0;
Estimate "Fas Young Slope" Log_Total_GSH 1 Log_Total_GSH*TwoGroups 0 1Log_Total_GSH*Protein 0 0 0 0 0 0 0 0 0 0 0 0 0 0 0 0 0 0 0
0 0 0 0 0 0 0 0 0 0 0 0 0 1 0 0 0 0 0 0 0 0 0 0 0 0 0 0 0 0 0 0 0 0 0 0 0 0 0 0 0 0 0 0 0 0 0 0 0 0 0 0 0 0 0 0 0 0 0 0 0 0 0 0 0
0 0 0 0 0 0 0 0 Log_Total_GSH*TwoGroups*Protein  0 0 0 0 0 0 0 0 0 0 0 0 0 0 0 0 0 0 0 0 0 0 0 0 0 0 0 0 0 0 0 0 0 0 0 0 0 0 0 0
0 0 0 0 0 0 0 0 0 0 0 0 0 0 0 0 0 0 0 0 0 0 0 0 0 0 0 0 0 0 0 0 0 0 0 0 0 0 0 0 0 0 0 0 0 0 0 0 0 0 0 0 0 0 0 0 0 0 0 0 0 0 0 0 0
0 0 0 0 0 0 0 0 0 0 0 0 0 0 0 0 0 0 0 1 0 0 0 0 0 0 0 0 0 0 0 0 0 0 0 0 0 0 0 0 0 0 0 0 0 0 0 0 0 0 0 0 0 0 0 0 0 0 0 0 0 0 0 0 0
0 0 0 0 0 0 0 0 0 0 0 0 0 0;
Estimate "Fli1 Young Slope" Log_Total_GSH 1 Log_Total_GSH*TwoGroups 0 1Log_Total_GSH*Protein 0 0 0 0 0 0 0 0 0 0 0 0 0 0 0 0 0 0
0 0 0 0 0 0 0 0 0 0 0 0 0 0 0 1 0 0 0 0 0 0 0 0 0 0 0 0 0 0 0 0 0 0 0 0 0 0 0 0 0 0 0 0 0 0 0 0 0 0 0 0 0 0 0 0 0 0 0 0 0 0 0 0 0
0 0 0 0 0 0 0 0 0 Log_Total_GSH*TwoGroups*Protein  0 0 0 0 0 0 0 0 0 0 0 0 0 0 0 0 0 0 0 0 0 0 0 0 0 0 0 0 0 0 0 0 0 0 0 0 0 0 0
0 0 0 0 0 0 0 0 0 0 0 0 0 0 0 0 0 0 0 0 0 0 0 0 0 0 0 0 0 0 0 0 0 0 0 0 0 0 0 0 0 0 0 0 0 0 0 0 0 0 0 0 0 0 0 0 0 0 0 0 0 0 0 0 0
0 0 0 0 0 0 0 0 0 0 0 0 0 0 0 0 0 0 0 0 0 1 0 0 0 0 0 0 0 0 0 0 0 0 0 0 0 0 0 0 0 0 0 0 0 0 0 0 0 0 0 0 0 0 0 0 0 0 0 0 0 0 0 0 0
0 0 0 0 0 0 0 0 0 0 0 0 0 0 0;
```

```
Estimate "Flrt2 Young Slope" Log_Total_GSH 1 Log_Total_GSH*TwoGroups 0 1Log_Total_GSH*Protein 0 0 0 0 0 0 0 0 0 0 0 0 0 0 0 0 0 0
0 0 0 0 0 0 0 0 0 0 0 0 0 0 0 0 1 0 0 0 0 0 0 0 0 0 0 0 0 0 0 0 0 0 0 0 0 0 0 0 0 0 0 0 0 0 0 0 0 0 0 0 0 0 0 0 0 0 0 0 0 0 0 0 0
0 0 0 0 0 0 0 0 0 Log_Total_GSH*TwoGroups*Protein  0 0 0 0 0 0 0 0 0 0 0 0 0 0 0 0 0 0 0 0 0 0 0 0 0 0 0 0 0 0 0 0 0 0 0 0 0 0 0
0 0 0 0 0 0 0 0 0 0 0 0 0 0 0 0 0 0 0 0 0 0 0 0 0 0 0 0 0 0 0 0 0 0 0 0 0 0 0 0 0 0 0 0 0 0 0 0 0 0 0 0 0 0 0 0 0 0 0 0 0 0 0 0 0
0 0 0 0 0 0 0 0 0 0 0 0 0 0 0 0 0 0 0 0 0 0 1 0 0 0 0 0 0 0 0 0 0 0 0 0 0 0 0 0 0 0 0 0 0 0 0 0 0 0 0 0 0 0 0 0 0 0 0 0 0 0 0 0 0
0 0 0 0 0 0 0 0 0 0 0 0 0 0 0;
Estimate "Foxo1 Young Slope" Log_Total_GSH 1 Log_Total_GSH*TwoGroups 0 1Log_Total_GSH*Protein 0 0 0 0 0 0 0 0 0 0 0 0 0 0 0 0 0 0
0 0 0 0 0 0 0 0 0 0 0 0 0 0 0 0 0 1 0 0 0 0 0 0 0 0 0 0 0 0 0 0 0 0 0 0 0 0 0 0 0 0 0 0 0 0 0 0 0 0 0 0 0 0 0 0 0 0 0 0 0 0 0 0 0
0 0 0 0 0 0 0 0 0 Log_Total_GSH*TwoGroups*Protein  0 0 0 0 0 0 0 0 0 0 0 0 0 0 0 0 0 0 0 0 0 0 0 0 0 0 0 0 0 0 0 0 0 0 0 0 0 0 0
0 0 0 0 0 0 0 0 0 0 0 0 0 0 0 0 0 0 0 0 0 0 0 0 0 0 0 0 0 0 0 0 0 0 0 0 0 0 0 0 0 0 0 0 0 0 0 0 0 0 0 0 0 0 0 0 0 0 0 0 0 0 0 0 0
0 0 0 0 0 0 0 0 0 0 0 0 0 0 0 0 0 0 0 0 0 0 0 1 0 0 0 0 0 0 0 0 0 0 0 0 0 0 0 0 0 0 0 0 0 0 0 0 0 0 0 0 0 0 0 0 0 0 0 0 0 0 0 0 0
0 0 0 0 0 0 0 0 0 0 0 0 0 0 0;
Estimate "Fst Young Slope" Log_Total_GSH 1 Log_Total_GSH*TwoGroups 0 1Log_Total_GSH*Protein 0 0 0 0 0 0 0 0 0 0 0 0 0 0 0 0 0 0 0
0 0 0 0 0 0 0 0 0 0 0 0 0 0 0 0 0 1 0 0 0 0 0 0 0 0 0 0 0 0 0 0 0 0 0 0 0 0 0 0 0 0 0 0 0 0 0 0 0 0 0 0 0 0 0 0 0 0 0 0 0 0 0 0 0
0 0 0 0 0 0 0 0 Log_Total_GSH*TwoGroups*Protein  0 0 0 0 0 0 0 0 0 0 0 0 0 0 0 0 0 0 0 0 0 0 0 0 0 0 0 0 0 0 0 0 0 0 0 0 0 0 0 0
0 0 0 0 0 0 0 0 0 0 0 0 0 0 0 0 0 0 0 0 0 0 0 0 0 0 0 0 0 0 0 0 0 0 0 0 0 0 0 0 0 0 0 0 0 0 0 0 0 0 0 0 0 0 0 0 0 0 0 0 0 0 0 0 0
0 0 0 0 0 0 0 0 0 0 0 0 0 0 0 0 0 0 0 0 0 0 0 1 0 0 0 0 0 0 0 0 0 0 0 0 0 0 0 0 0 0 0 0 0 0 0 0 0 0 0 0 0 0 0 0 0 0 0 0 0 0 0 0 0
0 0 0 0 0 0 0 0 0 0 0 0 0 0;
Estimate "Fstl3 Young Slope" Log_Total_GSH 1 Log_Total_GSH*TwoGroups 0 1Log_Total_GSH*Protein 0 0 0 0 0 0 0 0 0 0 0 0 0 0 0 0 0 0
0 0 0 0 0 0 0 0 0 0 0 0 0 0 0 0 0 0 0 1 0 0 0 0 0 0 0 0 0 0 0 0 0 0 0 0 0 0 0 0 0 0 0 0 0 0 0 0 0 0 0 0 0 0 0 0 0 0 0 0 0 0 0 0 0
0 0 0 0 0 0 0 0 0 Log_Total_GSH*TwoGroups*Protein  0 0 0 0 0 0 0 0 0 0 0 0 0 0 0 0 0 0 0 0 0 0 0 0 0 0 0 0 0 0 0 0 0 0 0 0 0 0 0
0 0 0 0 0 0 0 0 0 0 0 0 0 0 0 0 0 0 0 0 0 0 0 0 0 0 0 0 0 0 0 0 0 0 0 0 0 0 0 0 0 0 0 0 0 0 0 0 0 0 0 0 0 0 0 0 0 0 0 0 0 0 0 0 0
0 0 0 0 0 0 0 0 0 0 0 0 0 0 0 0 0 0 0 0 0 0 0 0 0 1 0 0 0 0 0 0 0 0 0 0 0 0 0 0 0 0 0 0 0 0 0 0 0 0 0 0 0 0 0 0 0 0 0 0 0 0 0 0 0
0 0 0 0 0 0 0 0 0 0 0 0 0 0 0;
Estimate "Gcg Young Slope" Log_Total_GSH 1 Log_Total_GSH*TwoGroups 0 1Log_Total_GSH*Protein 0 0 0 0 0 0 0 0 0 0 0 0 0 0 0 0 0 0 0
0 0 0 0 0 0 0 0 0 0 0 0 0 0 0 0 0 0 0 1 0 0 0 0 0 0 0 0 0 0 0 0 0 0 0 0 0 0 0 0 0 0 0 0 0 0 0 0 0 0 0 0 0 0 0 0 0 0 0 0 0 0 0 0 0
0 0 0 0 0 0 0 0 Log_Total_GSH*TwoGroups*Protein  0 0 0 0 0 0 0 0 0 0 0 0 0 0 0 0 0 0 0 0 0 0 0 0 0 0 0 0 0 0 0 0 0 0 0 0 0 0 0 0
0 0 0 0 0 0 0 0 0 0 0 0 0 0 0 0 0 0 0 0 0 0 0 0 0 0 0 0 0 0 0 0 0 0 0 0 0 0 0 0 0 0 0 0 0 0 0 0 0 0 0 0 0 0 0 0 0 0 0 0 0 0 0 0 0
0 0 0 0 0 0 0 0 0 0 0 0 0 0 0 0 0 0 0 0 0 0 0 0 0 1 0 0 0 0 0 0 0 0 0 0 0 0 0 0 0 0 0 0 0 0 0 0 0 0 0 0 0 0 0 0 0 0 0 0 0 0 0 0 0
0 0 0 0 0 0 0 0 0 0 0 0 0 0;
Estimate "Gdnf Young Slope" Log_Total_GSH 1 Log_Total_GSH*TwoGroups 0 1Log_Total_GSH*Protein 0 0 0 0 0 0 0 0 0 0 0 0 0 0 0 0 0 0
0 0 0 0 0 0 0 0 0 0 0 0 0 0 0 0 0 0 0 0 0 1 0 0 0 0 0 0 0 0 0 0 0 0 0 0 0 0 0 0 0 0 0 0 0 0 0 0 0 0 0 0 0 0 0 0 0 0 0 0 0 0 0 0 0
0 0 0 0 0 0 0 0 0 Log_Total_GSH*TwoGroups*Protein  0 0 0 0 0 0 0 0 0 0 0 0 0 0 0 0 0 0 0 0 0 0 0 0 0 0 0 0 0 0 0 0 0 0 0 0 0 0 0
0 0 0 0 0 0 0 0 0 0 0 0 0 0 0 0 0 0 0 0 0 0 0 0 0 0 0 0 0 0 0 0 0 0 0 0 0 0 0 0 0 0 0 0 0 0 0 0 0 0 0 0 0 0 0 0 0 0 0 0 0 0 0 0 0
0 0 0 0 0 0 0 0 0 0 0 0 0 0 0 0 0 0 0 0 0 0 0 0 0 0 0 1 0 0 0 0 0 0 0 0 0 0 0 0 0 0 0 0 0 0 0 0 0 0 0 0 0 0 0 0 0 0 0 0 0 0 0 0 0
0 0 0 0 0 0 0 0 0 0 0 0 0 0 0;
Estimate "Gfra1 Young Slope" Log_Total_GSH 1 Log_Total_GSH*TwoGroups 0 1Log_Total_GSH*Protein 0 0 0 0 0 0 0 0 0 0 0 0 0 0 0 0 0 0
0 0 0 0 0 0 0 0 0 0 0 0 0 0 0 0 0 0 0 0 0 0 1 0 0 0 0 0 0 0 0 0 0 0 0 0 0 0 0 0 0 0 0 0 0 0 0 0 0 0 0 0 0 0 0 0 0 0 0 0 0 0 0 0 0
0 0 0 0 0 0 0 0 0 Log_Total_GSH*TwoGroups*Protein  0 0 0 0 0 0 0 0 0 0 0 0 0 0 0 0 0 0 0 0 0 0 0 0 0 0 0 0 0 0 0 0 0 0 0 0 0 0 0
0 0 0 0 0 0 0 0 0 0 0 0 0 0 0 0 0 0 0 0 0 0 0 0 0 0 0 0 0 0 0 0 0 0 0 0 0 0 0 0 0 0 0 0 0 0 0 0 0 0 0 0 0 0 0 0 0 0 0 0 0 0 0 0 0
0 0 0 0 0 0 0 0 0 0 0 0 0 0 0 0 0 0 0 0 0 0 0 0 0 0 0 0 1 0 0 0 0 0 0 0 0 0 0 0 0 0 0 0 0 0 0 0 0 0 0 0 0 0 0 0 0 0 0 0 0 0 0 0 0
0 0 0 0 0 0 0 0 0 0 0 0 0 0 0;
Estimate "Ghrl Young Slope" Log_Total_GSH 1 Log_Total_GSH*TwoGroups 0 1Log_Total_GSH*Protein 0 0 0 0 0 0 0 0 0 0 0 0 0 0 0 0 0 0
0 0 0 0 0 0 0 0 0 0 0 0 0 0 0 0 0 0 0 0 0 0 0 1 0 0 0 0 0 0 0 0 0 0 0 0 0 0 0 0 0 0 0 0 0 0 0 0 0 0 0 0 0 0 0 0 0 0 0 0 0 0 0 0 0
0 0 0 0 0 0 0 0 0 Log_Total_GSH*TwoGroups*Protein  0 0 0 0 0 0 0 0 0 0 0 0 0 0 0 0 0 0 0 0 0 0 0 0 0 0 0 0 0 0 0 0 0 0 0 0 0 0 0
0 0 0 0 0 0 0 0 0 0 0 0 0 0 0 0 0 0 0 0 0 0 0 0 0 0 0 0 0 0 0 0 0 0 0 0 0 0 0 0 0 0 0 0 0 0 0 0 0 0 0 0 0 0 0 0 0 0 0 0 0 0 0 0 0
0 0 0 0 0 0 0 0 0 0 0 0 0 0 0 0 0 0 0 0 0 0 0 0 0 0 0 0 0 1 0 0 0 0 0 0 0 0 0 0 0 0 0 0 0 0 0 0 0 0 0 0 0 0 0 0 0 0 0 0 0 0 0 0 0
0 0 0 0 0 0 0 0 0 0 0 0 0 0 0;
Estimate "Hgf Young Slope" Log_Total_GSH 1 Log_Total_GSH*TwoGroups 0 1Log_Total_GSH*Protein 0 0 0 0 0 0 0 0 0 0 0 0 0 0 0 0 0 0 0
0 0 0 0 0 0 0 0 0 0 0 0 0 0 0 0 0 0 0 0 0 0 0 1 0 0 0 0 0 0 0 0 0 0 0 0 0 0 0 0 0 0 0 0 0 0 0 0 0 0 0 0 0 0 0 0 0 0 0 0 0 0 0 0 0
0 0 0 0 0 0 0 0 Log_Total_GSH*TwoGroups*Protein  0 0 0 0 0 0 0 0 0 0 0 0 0 0 0 0 0 0 0 0 0 0 0 0 0 0 0 0 0 0 0 0 0 0 0 0 0 0 0 0
0 0 0 0 0 0 0 0 0 0 0 0 0 0 0 0 0 0 0 0 0 0 0 0 0 0 0 0 0 0 0 0 0 0 0 0 0 0 0 0 0 0 0 0 0 0 0 0 0 0 0 0 0 0 0 0 0 0 0 0 0 0 0 0 0
0 0 0 0 0 0 0 0 0 0 0 0 0 0 0 0 0 0 0 0 0 0 0 0 0 0 0 0 0 1 0 0 0 0 0 0 0 0 0 0 0 0 0 0 0 0 0 0 0 0 0 0 0 0 0 0 0 0 0 0 0 0 0 0 0
0 0 0 0 0 0 0 0 0 0 0 0 0 0;
Estimate "Igsf3 Young Slope" Log_Total_GSH 1 Log_Total_GSH*TwoGroups 0 1Log_Total_GSH*Protein 0 0 0 0 0 0 0 0 0 0 0 0 0 0 0 0 0 0
0 0 0 0 0 0 0 0 0 0 0 0 0 0 0 0 0 0 0 0 0 0 0 0 0 1 0 0 0 0 0 0 0 0 0 0 0 0 0 0 0 0 0 0 0 0 0 0 0 0 0 0 0 0 0 0 0 0 0 0 0 0 0 0 0
0 0 0 0 0 0 0 0 0 Log_Total_GSH*TwoGroups*Protein  0 0 0 0 0 0 0 0 0 0 0 0 0 0 0 0 0 0 0 0 0 0 0 0 0 0 0 0 0 0 0 0 0 0 0 0 0 0 0
0 0 0 0 0 0 0 0 0 0 0 0 0 0 0 0 0 0 0 0 0 0 0 0 0 0 0 0 0 0 0 0 0 0 0 0 0 0 0 0 0 0 0 0 0 0 0 0 0 0 0 0 0 0 0 0 0 0 0 0 0 0 0 0 0
0 0 0 0 0 0 0 0 0 0 0 0 0 0 0 0 0 0 0 0 0 0 0 0 0 0 0 0 0 0 0 1 0 0 0 0 0 0 0 0 0 0 0 0 0 0 0 0 0 0 0 0 0 0 0 0 0 0 0 0 0 0 0 0 0
0 0 0 0 0 0 0 0 0 0 0 0 0 0 0;
Estimate "Il10 Young Slope" Log_Total_GSH 1 Log_Total_GSH*TwoGroups 0 1Log_Total_GSH*Protein 0 0 0 0 0 0 0 0 0 0 0 0 0 0 0 0 0 0
0 0 0 0 0 0 0 0 0 0 0 0 0 0 0 0 0 0 0 0 0 0 0 0 0 0 1 0 0 0 0 0 0 0 0 0 0 0 0 0 0 0 0 0 0 0 0 0 0 0 0 0 0 0 0 0 0 0 0 0 0 0 0 0 0
0 0 0 0 0 0 0 0 0 Log_Total_GSH*TwoGroups*Protein  0 0 0 0 0 0 0 0 0 0 0 0 0 0 0 0 0 0 0 0 0 0 0 0 0 0 0 0 0 0 0 0 0 0 0 0 0 0 0
0 0 0 0 0 0 0 0 0 0 0 0 0 0 0 0 0 0 0 0 0 0 0 0 0 0 0 0 0 0 0 0 0 0 0 0 0 0 0 0 0 0 0 0 0 0 0 0 0 0 0 0 0 0 0 0 0 0 0 0 0 0 0 0 0
0 0 0 0 0 0 0 0 0 0 0 0 0 0 0 0 0 0 0 0 0 0 0 0 0 0 0 0 0 0 0 0 1 0 0 0 0 0 0 0 0 0 0 0 0 0 0 0 0 0 0 0 0 0 0 0 0 0 0 0 0 0 0 0 0
0 0 0 0 0 0 0 0 0 0 0 0 0 0 0;
Estimate "Il17a Young Slope" Log_Total_GSH 1 Log_Total_GSH*TwoGroups 0 1Log_Total_GSH*Protein 0 0 0 0 0 0 0 0 0 0 0 0 0 0 0 0 0 0
0 0 0 0 0 0 0 0 0 0 0 0 0 0 0 0 0 0 0 0 0 0 0 0 0 0 0 1 0 0 0 0 0 0 0 0 0 0 0 0 0 0 0 0 0 0 0 0 0 0 0 0 0 0 0 0 0 0 0 0 0 0 0 0 0
0 0 0 0 0 0 0 0 0 Log_Total_GSH*TwoGroups*Protein  0 0 0 0 0 0 0 0 0 0 0 0 0 0 0 0 0 0 0 0 0 0 0 0 0 0 0 0 0 0 0 0 0 0 0 0 0 0 0
0 0 0 0 0 0 0 0 0 0 0 0 0 0 0 0 0 0 0 0 0 0 0 0 0 0 0 0 0 0 0 0 0 0 0 0 0 0 0 0 0 0 0 0 0 0 0 0 0 0 0 0 0 0 0 0 0 0 0 0 0 0 0 0 0
0 0 0 0 0 0 0 0 0 0 0 0 0 0 0 0 0 0 0 0 0 0 0 0 0 0 0 0 0 0 0 0 0 1 0 0 0 0 0 0 0 0 0 0 0 0 0 0 0 0 0 0 0 0 0 0 0 0 0 0 0 0 0 0 0
0 0 0 0 0 0 0 0 0 0 0 0 0 0 0;
Estimate "Il17f Young Slope" Log_Total_GSH 1 Log_Total_GSH*TwoGroups 0 1Log_Total_GSH*Protein 0 0 0 0 0 0 0 0 0 0 0 0 0 0 0 0 0 0
0 0 0 0 0 0 0 0 0 0 0 0 0 0 0 0 0 0 0 0 0 0 0 0 0 0 0 0 1 0 0 0 0 0 0 0 0 0 0 0 0 0 0 0 0 0 0 0 0 0 0 0 0 0 0 0 0 0 0 0 0 0 0 0 0
0 0 0 0 0 0 0 0 0 Log_Total_GSH*TwoGroups*Protein  0 0 0 0 0 0 0 0 0 0 0 0 0 0 0 0 0 0 0 0 0 0 0 0 0 0 0 0 0 0 0 0 0 0 0 0 0 0 0
0 0 0 0 0 0 0 0 0 0 0 0 0 0 0 0 0 0 0 0 0 0 0 0 0 0 0 0 0 0 0 0 0 0 0 0 0 0 0 0 0 0 0 0 0 0 0 0 0 0 0 0 0 0 0 0 0 0 0 0 0 0 0 0 0
0 0 0 0 0 0 0 0 0 0 0 0 0 0 0 0 0 0 0 0 0 0 0 0 0 0 0 0 0 0 0 0 0 0 1 0 0 0 0 0 0 0 0 0 0 0 0 0 0 0 0 0 0 0 0 0 0 0 0 0 0 0 0 0 0
0 0 0 0 0 0 0 0 0 0 0 0 0 0 0;
Estimate "Il1a Young Slope" Log_Total_GSH 1 Log_Total_GSH*TwoGroups 0 1Log_Total_GSH*Protein 0 0 0 0 0 0 0 0 0 0 0 0 0 0 0 0 0 0
0 0 0 0 0 0 0 0 0 0 0 0 0 0 0 0 0 0 0 0 0 0 0 0 0 0 0 0 0 1 0 0 0 0 0 0 0 0 0 0 0 0 0 0 0 0 0 0 0 0 0 0 0 0 0 0 0 0 0 0 0 0 0 0 0
0 0 0 0 0 0 0 0 0 Log_Total_GSH*TwoGroups*Protein  0 0 0 0 0 0 0 0 0 0 0 0 0 0 0 0 0 0 0 0 0 0 0 0 0 0 0 0 0 0 0 0 0 0 0 0 0 0 0
0 0 0 0 0 0 0 0 0 0 0 0 0 0 0 0 0 0 0 0 0 0 0 0 0 0 0 0 0 0 0 0 0 0 0 0 0 0 0 0 0 0 0 0 0 0 0 0 0 0 0 0 0 0 0 0 0 0 0 0 0 0 0 0 0
0 0 0 0 0 0 0 0 0 0 0 0 0 0 0 0 0 0 0 0 0 0 0 0 0 0 0 0 0 0 0 0 0 0 0 1 0 0 0 0 0 0 0 0 0 0 0 0 0 0 0 0 0 0 0 0 0 0 0 0 0 0 0 0 0
0 0 0 0 0 0 0 0 0 0 0 0 0 0 0;
Estimate "Il1b Young Slope" Log_Total_GSH 1 Log_Total_GSH*TwoGroups 0 1Log_Total_GSH*Protein 0 0 0 0 0 0 0 0 0 0 0 0 0 0 0 0 0 0
0 0 0 0 0 0 0 0 0 0 0 0 0 0 0 0 0 0 0 0 0 0 0 0 0 0 0 0 0 0 1 0 0 0 0 0 0 0 0 0 0 0 0 0 0 0 0 0 0 0 0 0 0 0 0 0 0 0 0 0 0 0 0 0 0
0 0 0 0 0 0 0 0 0 Log_Total_GSH*TwoGroups*Protein  0 0 0 0 0 0 0 0 0 0 0 0 0 0 0 0 0 0 0 0 0 0 0 0 0 0 0 0 0 0 0 0 0 0 0 0 0 0 0
0 0 0 0 0 0 0 0 0 0 0 0 0 0 0 0 0 0 0 0 0 0 0 0 0 0 0 0 0 0 0 0 0 0 0 0 0 0 0 0 0 0 0 0 0 0 0 0 0 0 0 0 0 0 0 0 0 0 0 0 0 0 0 0 0
0 0 0 0 0 0 0 0 0 0 0 0 0 0 0 0 0 0 0 0 0 0 0 0 0 0 0 0 0 0 0 0 0 0 0 0 1 0 0 0 0 0 0 0 0 0 0 0 0 0 0 0 0 0 0 0 0 0 0 0 0 0 0 0 0
0 0 0 0 0 0 0 0 0 0 0 0 0 0 0;
Estimate "Il23r Young Slope" Log_Total_GSH 1 Log_Total_GSH*TwoGroups 0 1Log_Total_GSH*Protein 0 0 0 0 0 0 0 0 0 0 0 0 0 0 0 0 0 0
0 0 0 0 0 0 0 0 0 0 0 0 0 0 0 0 0 0 0 0 0 0 0 0 0 0 0 0 0 0 0 1 0 0 0 0 0 0 0 0 0 0 0 0 0 0 0 0 0 0 0 0 0 0 0 0 0 0 0 0 0 0 0 0 0
0 0 0 0 0 0 0 0 0 Log_Total_GSH*TwoGroups*Protein  0 0 0 0 0 0 0 0 0 0 0 0 0 0 0 0 0 0 0 0 0 0 0 0 0 0 0 0 0 0 0 0 0 0 0 0 0 0 0
0 0 0 0 0 0 0 0 0 0 0 0 0 0 0 0 0 0 0 0 0 0 0 0 0 0 0 0 0 0 0 0 0 0 0 0 0 0 0 0 0 0 0 0 0 0 0 0 0 0 0 0 0 0 0 0 0 0 0 0 0 0 0 0 0
```

```
0 0 0 0 0 0 0 0 0 0 0 0 0 0 0 0 0 0 0 0 0 0 0 0 0 0 0 0 0 0 0 0 0 0 0 0 0 1 0 0 0 0 0 0 0 0 0 0 0 0 0 0 0 0 0 0 0 0 0 0 0 0 0 0 0
0 0 0 0 0 0 0 0 0 0 0 0 0 0 0;
Estimate "Il5 Young Slope" Log_Total_GSH 1 Log_Total_GSH*TwoGroups 0 1Log_Total_GSH*Protein 0 0 0 0 0 0 0 0 0 0 0 0 0 0 0 0 0 0 0
0 0 0 0 0 0 0 0 0 0 0 0 0 0 0 0 0 0 0 0 0 0 0 0 0 0 0 0 0 0 0 1 0 0 0 0 0 0 0 0 0 0 0 0 0 0 0 0 0 0 0 0 0 0 0 0 0 0 0 0 0 0 0 0 0
0 0 0 0 0 0 0 0 Log_Total_GSH*TwoGroups*Protein  0 0 0 0 0 0 0 0 0 0 0 0 0 0 0 0 0 0 0 0 0 0 0 0 0 0 0 0 0 0 0 0 0 0 0 0 0 0 0 0
0 0 0 0 0 0 0 0 0 0 0 0 0 0 0 0 0 0 0 0 0 0 0 0 0 0 0 0 0 0 0 0 0 0 0 0 0 0 0 0 0 0 0 0 0 0 0 0 0 0 0 0 0 0 0 0 0 0 0 0 0 0 0 0 0
0 0 0 0 0 0 0 0 0 0 0 0 0 0 0 0 0 0 0 0 0 0 0 0 0 0 0 0 0 0 0 0 0 0 0 0 0 1 0 0 0 0 0 0 0 0 0 0 0 0 0 0 0 0 0 0 0 0 0 0 0 0 0 0 0
0 0 0 0 0 0 0 0 0 0 0 0 0 0;
Estimate "Il6 Young Slope" Log_Total_GSH 1 Log_Total_GSH*TwoGroups 0 1Log_Total_GSH*Protein 0 0 0 0 0 0 0 0 0 0 0 0 0 0 0 0 0 0 0
0 0 0 0 0 0 0 0 0 0 0 0 0 0 0 0 0 0 0 0 0 0 0 0 0 0 0 0 0 0 0 0 1 0 0 0 0 0 0 0 0 0 0 0 0 0 0 0 0 0 0 0 0 0 0 0 0 0 0 0 0 0 0 0 0
0 0 0 0 0 0 0 0 Log_Total_GSH*TwoGroups*Protein  0 0 0 0 0 0 0 0 0 0 0 0 0 0 0 0 0 0 0 0 0 0 0 0 0 0 0 0 0 0 0 0 0 0 0 0 0 0 0 0
0 0 0 0 0 0 0 0 0 0 0 0 0 0 0 0 0 0 0 0 0 0 0 0 0 0 0 0 0 0 0 0 0 0 0 0 0 0 0 0 0 0 0 0 0 0 0 0 0 0 0 0 0 0 0 0 0 0 0 0 0 0 0 0 0
0 0 0 0 0 0 0 0 0 0 0 0 0 0 0 0 0 0 0 0 0 0 0 0 0 0 0 0 0 0 0 0 0 0 0 0 0 0 1 0 0 0 0 0 0 0 0 0 0 0 0 0 0 0 0 0 0 0 0 0 0 0 0 0 0
0 0 0 0 0 0 0 0 0 0 0 0 0 0;
Estimate "Itgb1bp2 Young Slope" Log_Total_GSH 1 Log_Total_GSH*TwoGroups 0 1Log_Total_GSH*Protein 0 0 0 0 0 0 0 0 0 0 0 0 0 0 0 0
0 0 0 0 0 0 0 0 0 0 0 0 0 0 0 0 0 0 0 0 0 0 0 0 0 0 0 0 0 0 0 0 0 0 0 0 1 0 0 0 0 0 0 0 0 0 0 0 0 0 0 0 0 0 0 0 0 0 0 0 0 0 0 0 0
0 0 0 0 0 0 0 0 0 0 0 Log_Total_GSH*TwoGroups*Protein  0 0 0 0 0 0 0 0 0 0 0 0 0 0 0 0 0 0 0 0 0 0 0 0 0 0 0 0 0 0 0 0 0 0 0 0 0
0 0 0 0 0 0 0 0 0 0 0 0 0 0 0 0 0 0 0 0 0 0 0 0 0 0 0 0 0 0 0 0 0 0 0 0 0 0 0 0 0 0 0 0 0 0 0 0 0 0 0 0 0 0 0 0 0 0 0 0 0 0 0 0 0
0 0 0 0 0 0 0 0 0 0 0 0 0 0 0 0 0 0 0 0 0 0 0 0 0 0 0 0 0 0 0 0 0 0 0 0 0 0 0 0 0 0 1 0 0 0 0 0 0 0 0 0 0 0 0 0 0 0 0 0 0 0 0 0 0
0 0 0 0 0 0 0 0 0 0 0 0 0 0 0 0 0;
Estimate "Itgb6 Young Slope" Log_Total_GSH 1 Log_Total_GSH*TwoGroups 0 1Log_Total_GSH*Protein 0 0 0 0 0 0 0 0 0 0 0 0 0 0 0 0 0 0
0 0 0 0 0 0 0 0 0 0 0 0 0 0 0 0 0 0 0 0 0 0 0 0 0 0 0 0 0 0 0 0 0 0 0 1 0 0 0 0 0 0 0 0 0 0 0 0 0 0 0 0 0 0 0 0 0 0 0 0 0 0 0 0 0
0 0 0 0 0 0 0 0 0 Log_Total_GSH*TwoGroups*Protein  0 0 0 0 0 0 0 0 0 0 0 0 0 0 0 0 0 0 0 0 0 0 0 0 0 0 0 0 0 0 0 0 0 0 0 0 0 0 0
0 0 0 0 0 0 0 0 0 0 0 0 0 0 0 0 0 0 0 0 0 0 0 0 0 0 0 0 0 0 0 0 0 0 0 0 0 0 0 0 0 0 0 0 0 0 0 0 0 0 0 0 0 0 0 0 0 0 0 0 0 0 0 0 0
0 0 0 0 0 0 0 0 0 0 0 0 0 0 0 0 0 0 0 0 0 0 0 0 0 0 0 0 0 0 0 0 0 0 0 0 0 0 0 0 0 1 0 0 0 0 0 0 0 0 0 0 0 0 0 0 0 0 0 0 0 0 0 0 0
0 0 0 0 0 0 0 0 0 0 0 0 0 0 0;
Estimate "Kitlg Young Slope" Log_Total_GSH 1 Log_Total_GSH*TwoGroups 0 1Log_Total_GSH*Protein 0 0 0 0 0 0 0 0 0 0 0 0 0 0 0 0 0 0
0 0 0 0 0 0 0 0 0 0 0 0 0 0 0 0 0 0 0 0 0 0 0 0 0 0 0 0 0 0 0 0 0 0 0 0 1 0 0 0 0 0 0 0 0 0 0 0 0 0 0 0 0 0 0 0 0 0 0 0 0 0 0 0 0
0 0 0 0 0 0 0 0 0 Log_Total_GSH*TwoGroups*Protein  0 0 0 0 0 0 0 0 0 0 0 0 0 0 0 0 0 0 0 0 0 0 0 0 0 0 0 0 0 0 0 0 0 0 0 0 0 0 0
0 0 0 0 0 0 0 0 0 0 0 0 0 0 0 0 0 0 0 0 0 0 0 0 0 0 0 0 0 0 0 0 0 0 0 0 0 0 0 0 0 0 0 0 0 0 0 0 0 0 0 0 0 0 0 0 0 0 0 0 0 0 0 0 0
0 0 0 0 0 0 0 0 0 0 0 0 0 0 0 0 0 0 0 0 0 0 0 0 0 0 0 0 0 0 0 0 0 0 0 0 0 0 0 0 0 0 1 0 0 0 0 0 0 0 0 0 0 0 0 0 0 0 0 0 0 0 0 0 0
0 0 0 0 0 0 0 0 0 0 0 0 0 0 0;
Estimate "Lgmn Young Slope" Log_Total_GSH 1 Log_Total_GSH*TwoGroups 0 1Log_Total_GSH*Protein 0 0 0 0 0 0 0 0 0 0 0 0 0 0 0 0 0 0
0 0 0 0 0 0 0 0 0 0 0 0 0 0 0 0 0 0 0 0 0 0 0 0 0 0 0 0 0 0 0 0 0 0 0 0 0 1 0 0 0 0 0 0 0 0 0 0 0 0 0 0 0 0 0 0 0 0 0 0 0 0 0 0 0
0 0 0 0 0 0 0 0 0 Log_Total_GSH*TwoGroups*Protein  0 0 0 0 0 0 0 0 0 0 0 0 0 0 0 0 0 0 0 0 0 0 0 0 0 0 0 0 0 0 0 0 0 0 0 0 0 0 0
0 0 0 0 0 0 0 0 0 0 0 0 0 0 0 0 0 0 0 0 0 0 0 0 0 0 0 0 0 0 0 0 0 0 0 0 0 0 0 0 0 0 0 0 0 0 0 0 0 0 0 0 0 0 0 0 0 0 0 0 0 0 0 0 0
0 0 0 0 0 0 0 0 0 0 0 0 0 0 0 0 0 0 0 0 0 0 0 0 0 0 0 0 0 0 0 0 0 0 0 0 0 0 0 0 0 0 0 1 0 0 0 0 0 0 0 0 0 0 0 0 0 0 0 0 0 0 0 0 0
0 0 0 0 0 0 0 0 0 0 0 0 0 0 0;
Estimate "Lpl Young Slope" Log_Total_GSH 1 Log_Total_GSH*TwoGroups 0 1Log_Total_GSH*Protein 0 0 0 0 0 0 0 0 0 0 0 0 0 0 0 0 0 0 0
0 0 0 0 0 0 0 0 0 0 0 0 0 0 0 0 0 0 0 0 0 0 0 0 0 0 0 0 0 0 0 0 0 0 0 0 0 1 0 0 0 0 0 0 0 0 0 0 0 0 0 0 0 0 0 0 0 0 0 0 0 0 0 0 0
0 0 0 0 0 0 0 0 Log_Total_GSH*TwoGroups*Protein  0 0 0 0 0 0 0 0 0 0 0 0 0 0 0 0 0 0 0 0 0 0 0 0 0 0 0 0 0 0 0 0 0 0 0 0 0 0 0 0
0 0 0 0 0 0 0 0 0 0 0 0 0 0 0 0 0 0 0 0 0 0 0 0 0 0 0 0 0 0 0 0 0 0 0 0 0 0 0 0 0 0 0 0 0 0 0 0 0 0 0 0 0 0 0 0 0 0 0 0 0 0 0 0 0
0 0 0 0 0 0 0 0 0 0 0 0 0 0 0 0 0 0 0 0 0 0 0 0 0 0 0 0 0 0 0 0 0 0 0 0 0 0 0 0 0 0 0 1 0 0 0 0 0 0 0 0 0 0 0 0 0 0 0 0 0 0 0 0 0
0 0 0 0 0 0 0 0 0 0 0 0 0 0;
Estimate "Map2k6 Young Slope" Log_Total_GSH 1 Log_Total_GSH*TwoGroups 0 1Log_Total_GSH*Protein 0 0 0 0 0 0 0 0 0 0 0 0 0 0 0 0 0
0 0 0 0 0 0 0 0 0 0 0 0 0 0 0 0 0 0 0 0 0 0 0 0 0 0 0 0 0 0 0 0 0 0 0 0 0 0 0 0 1 0 0 0 0 0 0 0 0 0 0 0 0 0 0 0 0 0 0 0 0 0 0 0 0
0 0 0 0 0 0 0 0 0 0 Log_Total_GSH*TwoGroups*Protein  0 0 0 0 0 0 0 0 0 0 0 0 0 0 0 0 0 0 0 0 0 0 0 0 0 0 0 0 0 0 0 0 0 0 0 0 0 0
0 0 0 0 0 0 0 0 0 0 0 0 0 0 0 0 0 0 0 0 0 0 0 0 0 0 0 0 0 0 0 0 0 0 0 0 0 0 0 0 0 0 0 0 0 0 0 0 0 0 0 0 0 0 0 0 0 0 0 0 0 0 0 0 0
0 0 0 0 0 0 0 0 0 0 0 0 0 0 0 0 0 0 0 0 0 0 0 0 0 0 0 0 0 0 0 0 0 0 0 0 0 0 0 0 0 0 0 0 0 0 1 0 0 0 0 0 0 0 0 0 0 0 0 0 0 0 0 0 0
0 0 0 0 0 0 0 0 0 0 0 0 0 0 0 0;
Estimate "Matn2 Young Slope" Log_Total_GSH 1 Log_Total_GSH*TwoGroups 0 1Log_Total_GSH*Protein 0 0 0 0 0 0 0 0 0 0 0 0 0 0 0 0 0 0
0 0 0 0 0 0 0 0 0 0 0 0 0 0 0 0 0 0 0 0 0 0 0 0 0 0 0 0 0 0 0 0 0 0 0 0 0 0 0 0 1 0 0 0 0 0 0 0 0 0 0 0 0 0 0 0 0 0 0 0 0 0 0 0 0
0 0 0 0 0 0 0 0 0 Log_Total_GSH*TwoGroups*Protein  0 0 0 0 0 0 0 0 0 0 0 0 0 0 0 0 0 0 0 0 0 0 0 0 0 0 0 0 0 0 0 0 0 0 0 0 0 0 0
0 0 0 0 0 0 0 0 0 0 0 0 0 0 0 0 0 0 0 0 0 0 0 0 0 0 0 0 0 0 0 0 0 0 0 0 0 0 0 0 0 0 0 0 0 0 0 0 0 0 0 0 0 0 0 0 0 0 0 0 0 0 0 0 0
0 0 0 0 0 0 0 0 0 0 0 0 0 0 0 0 0 0 0 0 0 0 0 0 0 0 0 0 0 0 0 0 0 0 0 0 0 0 0 0 0 0 0 0 0 0 1 0 0 0 0 0 0 0 0 0 0 0 0 0 0 0 0 0 0
0 0 0 0 0 0 0 0 0 0 0 0 0 0 0;
Estimate "Mia Young Slope" Log_Total_GSH 1 Log_Total_GSH*TwoGroups 0 1Log_Total_GSH*Protein 0 0 0 0 0 0 0 0 0 0 0 0 0 0 0 0 0 0 0
0 0 0 0 0 0 0 0 0 0 0 0 0 0 0 0 0 0 0 0 0 0 0 0 0 0 0 0 0 0 0 0 0 0 0 0 0 0 0 0 1 0 0 0 0 0 0 0 0 0 0 0 0 0 0 0 0 0 0 0 0 0 0 0 0
0 0 0 0 0 0 0 0 Log_Total_GSH*TwoGroups*Protein  0 0 0 0 0 0 0 0 0 0 0 0 0 0 0 0 0 0 0 0 0 0 0 0 0 0 0 0 0 0 0 0 0 0 0 0 0 0 0 0
0 0 0 0 0 0 0 0 0 0 0 0 0 0 0 0 0 0 0 0 0 0 0 0 0 0 0 0 0 0 0 0 0 0 0 0 0 0 0 0 0 0 0 0 0 0 0 0 0 0 0 0 0 0 0 0 0 0 0 0 0 0 0 0 0
0 0 0 0 0 0 0 0 0 0 0 0 0 0 0 0 0 0 0 0 0 0 0 0 0 0 0 0 0 0 0 0 0 0 0 0 0 0 0 0 0 0 0 0 0 0 1 0 0 0 0 0 0 0 0 0 0 0 0 0 0 0 0 0 0
0 0 0 0 0 0 0 0 0 0 0 0 0 0;
Estimate "Nadk Young Slope" Log_Total_GSH 1 Log_Total_GSH*TwoGroups 0 1Log_Total_GSH*Protein 0 0 0 0 0 0 0 0 0 0 0 0 0 0 0 0 0 0
0 0 0 0 0 0 0 0 0 0 0 0 0 0 0 0 0 0 0 0 0 0 0 0 0 0 0 0 0 0 0 0 0 0 0 0 0 0 0 0 0 0 1 0 0 0 0 0 0 0 0 0 0 0 0 0 0 0 0 0 0 0 0 0 0
0 0 0 0 0 0 0 0 0 Log_Total_GSH*TwoGroups*Protein  0 0 0 0 0 0 0 0 0 0 0 0 0 0 0 0 0 0 0 0 0 0 0 0 0 0 0 0 0 0 0 0 0 0 0 0 0 0 0
0 0 0 0 0 0 0 0 0 0 0 0 0 0 0 0 0 0 0 0 0 0 0 0 0 0 0 0 0 0 0 0 0 0 0 0 0 0 0 0 0 0 0 0 0 0 0 0 0 0 0 0 0 0 0 0 0 0 0 0 0 0 0 0 0
0 0 0 0 0 0 0 0 0 0 0 0 0 0 0 0 0 0 0 0 0 0 0 0 0 0 0 0 0 0 0 0 0 0 0 0 0 0 0 0 0 0 0 0 0 0 0 0 1 0 0 0 0 0 0 0 0 0 0 0 0 0 0 0 0
0 0 0 0 0 0 0 0 0 0 0 0 0 0 0;
Estimate "Notch3 Young Slope" Log_Total_GSH 1 Log_Total_GSH*TwoGroups 0 1Log_Total_GSH*Protein 0 0 0 0 0 0 0 0 0 0 0 0 0 0 0 0 0
0 0 0 0 0 0 0 0 0 0 0 0 0 0 0 0 0 0 0 0 0 0 0 0 0 0 0 0 0 0 0 0 0 0 0 0 0 0 0 0 0 0 0 0 1 0 0 0 0 0 0 0 0 0 0 0 0 0 0 0 0 0 0 0 0
0 0 0 0 0 0 0 0 0 0 Log_Total_GSH*TwoGroups*Protein  0 0 0 0 0 0 0 0 0 0 0 0 0 0 0 0 0 0 0 0 0 0 0 0 0 0 0 0 0 0 0 0 0 0 0 0 0 0
0 0 0 0 0 0 0 0 0 0 0 0 0 0 0 0 0 0 0 0 0 0 0 0 0 0 0 0 0 0 0 0 0 0 0 0 0 0 0 0 0 0 0 0 0 0 0 0 0 0 0 0 0 0 0 0 0 0 0 0 0 0 0 0 0
0 0 0 0 0 0 0 0 0 0 0 0 0 0 0 0 0 0 0 0 0 0 0 0 0 0 0 0 0 0 0 0 0 0 0 0 0 0 0 0 0 0 0 0 0 0 0 0 0 0 1 0 0 0 0 0 0 0 0 0 0 0 0 0 0
0 0 0 0 0 0 0 0 0 0 0 0 0 0 0 0;
Estimate "Ntf3 Young Slope" Log_Total_GSH 1 Log_Total_GSH*TwoGroups 0 1Log_Total_GSH*Protein 0 0 0 0 0 0 0 0 0 0 0 0 0 0 0 0 0 0
0 0 0 0 0 0 0 0 0 0 0 0 0 0 0 0 0 0 0 0 0 0 0 0 0 0 0 0 0 0 0 0 0 0 0 0 0 0 0 0 0 0 0 0 1 0 0 0 0 0 0 0 0 0 0 0 0 0 0 0 0 0 0 0 0
0 0 0 0 0 0 0 0 0 Log_Total_GSH*TwoGroups*Protein  0 0 0 0 0 0 0 0 0 0 0 0 0 0 0 0 0 0 0 0 0 0 0 0 0 0 0 0 0 0 0 0 0 0 0 0 0 0 0
0 0 0 0 0 0 0 0 0 0 0 0 0 0 0 0 0 0 0 0 0 0 0 0 0 0 0 0 0 0 0 0 0 0 0 0 0 0 0 0 0 0 0 0 0 0 0 0 0 0 0 0 0 0 0 0 0 0 0 0 0 0 0 0 0
0 0 0 0 0 0 0 0 0 0 0 0 0 0 0 0 0 0 0 0 0 0 0 0 0 0 0 0 0 0 0 0 0 0 0 0 0 0 0 0 0 0 0 0 0 0 0 0 0 0 1 0 0 0 0 0 0 0 0 0 0 0 0 0 0
0 0 0 0 0 0 0 0 0 0 0 0 0 0 0;
Estimate "Pak4 Young Slope" Log_Total_GSH 1 Log_Total_GSH*TwoGroups 0 1Log_Total_GSH*Protein 0 0 0 0 0 0 0 0 0 0 0 0 0 0 0 0 0 0
0 0 0 0 0 0 0 0 0 0 0 0 0 0 0 0 0 0 0 0 0 0 0 0 0 0 0 0 0 0 0 0 0 0 0 0 0 0 0 0 0 0 0 0 0 1 0 0 0 0 0 0 0 0 0 0 0 0 0 0 0 0 0 0 0
0 0 0 0 0 0 0 0 0 Log_Total_GSH*TwoGroups*Protein  0 0 0 0 0 0 0 0 0 0 0 0 0 0 0 0 0 0 0 0 0 0 0 0 0 0 0 0 0 0 0 0 0 0 0 0 0 0 0
0 0 0 0 0 0 0 0 0 0 0 0 0 0 0 0 0 0 0 0 0 0 0 0 0 0 0 0 0 0 0 0 0 0 0 0 0 0 0 0 0 0 0 0 0 0 0 0 0 0 0 0 0 0 0 0 0 0 0 0 0 0 0 0 0
0 0 0 0 0 0 0 0 0 0 0 0 0 0 0 0 0 0 0 0 0 0 0 0 0 0 0 0 0 0 0 0 0 0 0 0 0 0 0 0 0 0 0 0 0 0 0 0 0 0 0 1 0 0 0 0 0 0 0 0 0 0 0 0 0
0 0 0 0 0 0 0 0 0 0 0 0 0 0 0;
Estimate "Parp1 Young Slope" Log_Total_GSH 1 Log_Total_GSH*TwoGroups 0 1Log_Total_GSH*Protein 0 0 0 0 0 0 0 0 0 0 0 0 0 0 0 0 0 0
0 0 0 0 0 0 0 0 0 0 0 0 0 0 0 0 0 0 0 0 0 0 0 0 0 0 0 0 0 0 0 0 0 0 0 0 0 0 0 0 0 0 0 0 0 0 1 0 0 0 0 0 0 0 0 0 0 0 0 0 0 0 0 0 0
0 0 0 0 0 0 0 0 0 Log_Total_GSH*TwoGroups*Protein  0 0 0 0 0 0 0 0 0 0 0 0 0 0 0 0 0 0 0 0 0 0 0 0 0 0 0 0 0 0 0 0 0 0 0 0 0 0 0
0 0 0 0 0 0 0 0 0 0 0 0 0 0 0 0 0 0 0 0 0 0 0 0 0 0 0 0 0 0 0 0 0 0 0 0 0 0 0 0 0 0 0 0 0 0 0 0 0 0 0 0 0 0 0 0 0 0 0 0 0 0 0 0 0
0 0 0 0 0 0 0 0 0 0 0 0 0 0 0 0 0 0 0 0 0 0 0 0 0 0 0 0 0 0 0 0 0 0 0 0 0 0 0 0 0 0 0 0 0 0 0 0 0 0 0 0 1 0 0 0 0 0 0 0 0 0 0 0 0
0 0 0 0 0 0 0 0 0 0 0 0 0 0 0;
Estimate "Pdgfb Young Slope" Log_Total_GSH 1 Log_Total_GSH*TwoGroups 0 1Log_Total_GSH*Protein 0 0 0 0 0 0 0 0 0 0 0 0 0 0 0 0 0 0
0 0 0 0 0 0 0 0 0 0 0 0 0 0 0 0 0 0 0 0 0 0 0 0 0 0 0 0 0 0 0 0 0 0 0 0 0 0 0 0 0 0 0 0 0 0 0 1 0 0 0 0 0 0 0 0 0 0 0 0 0 0 0 0 0
0 0 0 0 0 0 0 0 0 Log_Total_GSH*TwoGroups*Protein  0 0 0 0 0 0 0 0 0 0 0 0 0 0 0 0 0 0 0 0 0 0 0 0 0 0 0 0 0 0 0 0 0 0 0 0 0 0 0
```

```
0 0 0 0 0 0 0 0 0 0 0 0 0 0 0 0 0 0 0 0 0 0 0 0 0 0 0 0 0 0 0 0 0 0 0 0 0 0 0 0 0 0 0 0 0 0 0 0 0 0 0 0 0 0 0 0 0 0 0 0 0 0 0 0 0
0 0 0 0 0 0 0 0 0 0 0 0 0 0 0 0 0 0 0 0 0 0 0 0 0 0 0 0 0 0 0 0 0 0 0 0 0 0 0 0 0 0 0 0 0 0 0 0 0 0 0 0 0 1 0 0 0 0 0 0 0 0 0 0 0
0 0 0 0 0 0 0 0 0 0 0 0 0 0 0;
Estimate "Pla2g4a Young Slope" Log_Total_GSH 1 Log_Total_GSH*TwoGroups 0 1Log_Total_GSH*Protein 0 0 0 0 0 0 0 0 0 0 0 0 0 0 0 0 0
0 0 0 0 0 0 0 0 0 0 0 0 0 0 0 0 0 0 0 0 0 0 0 0 0 0 0 0 0 0 0 0 0 0 0 0 0 0 0 0 0 0 0 0 0 0 0 0 0 1 0 0 0 0 0 0 0 0 0 0 0 0 0 0 0
0 0 0 0 0 0 0 0 0 0 Log_Total_GSH*TwoGroups*Protein  0 0 0 0 0 0 0 0 0 0 0 0 0 0 0 0 0 0 0 0 0 0 0 0 0 0 0 0 0 0 0 0 0 0 0 0 0 0
0 0 0 0 0 0 0 0 0 0 0 0 0 0 0 0 0 0 0 0 0 0 0 0 0 0 0 0 0 0 0 0 0 0 0 0 0 0 0 0 0 0 0 0 0 0 0 0 0 0 0 0 0 0 0 0 0 0 0 0 0 0 0 0 0
0 0 0 0 0 0 0 0 0 0 0 0 0 0 0 0 0 0 0 0 0 0 0 0 0 0 0 0 0 0 0 0 0 0 0 0 0 0 0 0 0 0 0 0 0 0 0 0 0 0 0 0 0 0 0 1 0 0 0 0 0 0 0 0 0
0 0 0 0 0 0 0 0 0 0 0 0 0 0 0 0;
Estimate "Plin1 Young Slope" Log_Total_GSH 1 Log_Total_GSH*TwoGroups 0 1Log_Total_GSH*Protein 0 0 0 0 0 0 0 0 0 0 0 0 0 0 0 0 0 0
0 0 0 0 0 0 0 0 0 0 0 0 0 0 0 0 0 0 0 0 0 0 0 0 0 0 0 0 0 0 0 0 0 0 0 0 0 0 0 0 0 0 0 0 0 0 0 0 0 1 0 0 0 0 0 0 0 0 0 0 0 0 0 0 0
0 0 0 0 0 0 0 0 0 Log_Total_GSH*TwoGroups*Protein  0 0 0 0 0 0 0 0 0 0 0 0 0 0 0 0 0 0 0 0 0 0 0 0 0 0 0 0 0 0 0 0 0 0 0 0 0 0 0
0 0 0 0 0 0 0 0 0 0 0 0 0 0 0 0 0 0 0 0 0 0 0 0 0 0 0 0 0 0 0 0 0 0 0 0 0 0 0 0 0 0 0 0 0 0 0 0 0 0 0 0 0 0 0 0 0 0 0 0 0 0 0 0 0
0 0 0 0 0 0 0 0 0 0 0 0 0 0 0 0 0 0 0 0 0 0 0 0 0 0 0 0 0 0 0 0 0 0 0 0 0 0 0 0 0 0 0 0 0 0 0 0 0 0 0 0 0 0 0 1 0 0 0 0 0 0 0 0 0
0 0 0 0 0 0 0 0 0 0 0 0 0 0 0;
Estimate "Plxna4 Young Slope" Log_Total_GSH 1 Log_Total_GSH*TwoGroups 0 1Log_Total_GSH*Protein 0 0 0 0 0 0 0 0 0 0 0 0 0 0 0 0 0
0 0 0 0 0 0 0 0 0 0 0 0 0 0 0 0 0 0 0 0 0 0 0 0 0 0 0 0 0 0 0 0 0 0 0 0 0 0 0 0 0 0 0 0 0 0 0 0 0 0 0 1 0 0 0 0 0 0 0 0 0 0 0 0 0
0 0 0 0 0 0 0 0 0 0 Log_Total_GSH*TwoGroups*Protein  0 0 0 0 0 0 0 0 0 0 0 0 0 0 0 0 0 0 0 0 0 0 0 0 0 0 0 0 0 0 0 0 0 0 0 0 0 0
0 0 0 0 0 0 0 0 0 0 0 0 0 0 0 0 0 0 0 0 0 0 0 0 0 0 0 0 0 0 0 0 0 0 0 0 0 0 0 0 0 0 0 0 0 0 0 0 0 0 0 0 0 0 0 0 0 0 0 0 0 0 0 0 0
0 0 0 0 0 0 0 0 0 0 0 0 0 0 0 0 0 0 0 0 0 0 0 0 0 0 0 0 0 0 0 0 0 0 0 0 0 0 0 0 0 0 0 0 0 0 0 0 0 0 0 0 0 0 0 0 0 1 0 0 0 0 0 0 0
0 0 0 0 0 0 0 0 0 0 0 0 0 0 0 0;
Estimate "Ppp1r2 Young Slope" Log_Total_GSH 1 Log_Total_GSH*TwoGroups 0 1Log_Total_GSH*Protein 0 0 0 0 0 0 0 0 0 0 0 0 0 0 0 0 0
0 0 0 0 0 0 0 0 0 0 0 0 0 0 0 0 0 0 0 0 0 0 0 0 0 0 0 0 0 0 0 0 0 0 0 0 0 0 0 0 0 0 0 0 0 0 0 0 0 0 0 0 1 0 0 0 0 0 0 0 0 0 0 0 0
0 0 0 0 0 0 0 0 0 0 Log_Total_GSH*TwoGroups*Protein  0 0 0 0 0 0 0 0 0 0 0 0 0 0 0 0 0 0 0 0 0 0 0 0 0 0 0 0 0 0 0 0 0 0 0 0 0 0
0 0 0 0 0 0 0 0 0 0 0 0 0 0 0 0 0 0 0 0 0 0 0 0 0 0 0 0 0 0 0 0 0 0 0 0 0 0 0 0 0 0 0 0 0 0 0 0 0 0 0 0 0 0 0 0 0 0 0 0 0 0 0 0 0
0 0 0 0 0 0 0 0 0 0 0 0 0 0 0 0 0 0 0 0 0 0 0 0 0 0 0 0 0 0 0 0 0 0 0 0 0 0 0 0 0 0 0 0 0 0 0 0 0 0 0 0 0 0 0 0 0 0 1 0 0 0 0 0 0
0 0 0 0 0 0 0 0 0 0 0 0 0 0 0 0;
Estimate "Prdx5 Young Slope" Log_Total_GSH 1 Log_Total_GSH*TwoGroups 0 1Log_Total_GSH*Protein 0 0 0 0 0 0 0 0 0 0 0 0 0 0 0 0 0 0
0 0 0 0 0 0 0 0 0 0 0 0 0 0 0 0 0 0 0 0 0 0 0 0 0 0 0 0 0 0 0 0 0 0 0 0 0 0 0 0 0 0 0 0 0 0 0 0 0 0 0 0 1 0 0 0 0 0 0 0 0 0 0 0 0
0 0 0 0 0 0 0 0 0 Log_Total_GSH*TwoGroups*Protein  0 0 0 0 0 0 0 0 0 0 0 0 0 0 0 0 0 0 0 0 0 0 0 0 0 0 0 0 0 0 0 0 0 0 0 0 0 0 0
0 0 0 0 0 0 0 0 0 0 0 0 0 0 0 0 0 0 0 0 0 0 0 0 0 0 0 0 0 0 0 0 0 0 0 0 0 0 0 0 0 0 0 0 0 0 0 0 0 0 0 0 0 0 0 0 0 0 0 0 0 0 0 0 0
0 0 0 0 0 0 0 0 0 0 0 0 0 0 0 0 0 0 0 0 0 0 0 0 0 0 0 0 0 0 0 0 0 0 0 0 0 0 0 0 0 0 0 0 0 0 0 0 0 0 0 0 0 0 0 0 0 0 1 0 0 0 0 0 0
0 0 0 0 0 0 0 0 0 0 0 0 0 0 0;
Estimate "Qdpr Young Slope" Log_Total_GSH 1 Log_Total_GSH*TwoGroups 0 1Log_Total_GSH*Protein 0 0 0 0 0 0 0 0 0 0 0 0 0 0 0 0 0 0
0 0 0 0 0 0 0 0 0 0 0 0 0 0 0 0 0 0 0 0 0 0 0 0 0 0 0 0 0 0 0 0 0 0 0 0 0 0 0 0 0 0 0 0 0 0 0 0 0 0 0 0 0 1 0 0 0 0 0 0 0 0 0 0 0
0 0 0 0 0 0 0 0 0 Log_Total_GSH*TwoGroups*Protein  0 0 0 0 0 0 0 0 0 0 0 0 0 0 0 0 0 0 0 0 0 0 0 0 0 0 0 0 0 0 0 0 0 0 0 0 0 0 0
0 0 0 0 0 0 0 0 0 0 0 0 0 0 0 0 0 0 0 0 0 0 0 0 0 0 0 0 0 0 0 0 0 0 0 0 0 0 0 0 0 0 0 0 0 0 0 0 0 0 0 0 0 0 0 0 0 0 0 0 0 0 0 0 0
0 0 0 0 0 0 0 0 0 0 0 0 0 0 0 0 0 0 0 0 0 0 0 0 0 0 0 0 0 0 0 0 0 0 0 0 0 0 0 0 0 0 0 0 0 0 0 0 0 0 0 0 0 0 0 0 0 0 0 1 0 0 0 0 0
0 0 0 0 0 0 0 0 0 0 0 0 0 0 0;
Estimate "Rgma Young Slope" Log_Total_GSH 1 Log_Total_GSH*TwoGroups 0 1Log_Total_GSH*Protein 0 0 0 0 0 0 0 0 0 0 0 0 0 0 0 0 0 0
0 0 0 0 0 0 0 0 0 0 0 0 0 0 0 0 0 0 0 0 0 0 0 0 0 0 0 0 0 0 0 0 0 0 0 0 0 0 0 0 0 0 0 0 0 0 0 0 0 0 0 0 0 0 1 0 0 0 0 0 0 0 0 0 0
0 0 0 0 0 0 0 0 0 Log_Total_GSH*TwoGroups*Protein  0 0 0 0 0 0 0 0 0 0 0 0 0 0 0 0 0 0 0 0 0 0 0 0 0 0 0 0 0 0 0 0 0 0 0 0 0 0 0
0 0 0 0 0 0 0 0 0 0 0 0 0 0 0 0 0 0 0 0 0 0 0 0 0 0 0 0 0 0 0 0 0 0 0 0 0 0 0 0 0 0 0 0 0 0 0 0 0 0 0 0 0 0 0 0 0 0 0 0 0 0 0 0 0
0 0 0 0 0 0 0 0 0 0 0 0 0 0 0 0 0 0 0 0 0 0 0 0 0 0 0 0 0 0 0 0 0 0 0 0 0 0 0 0 0 0 0 0 0 0 0 0 0 0 0 0 0 0 0 0 0 0 0 0 1 0 0 0 0
0 0 0 0 0 0 0 0 0 0 0 0 0 0 0;
Estimate "Riox2 Young Slope" Log_Total_GSH 1 Log_Total_GSH*TwoGroups 0 1Log_Total_GSH*Protein 0 0 0 0 0 0 0 0 0 0 0 0 0 0 0 0 0 0
0 0 0 0 0 0 0 0 0 0 0 0 0 0 0 0 0 0 0 0 0 0 0 0 0 0 0 0 0 0 0 0 0 0 0 0 0 0 0 0 0 0 0 0 0 0 0 0 0 0 0 0 0 0 0 1 0 0 0 0 0 0 0 0 0
0 0 0 0 0 0 0 0 0 Log_Total_GSH*TwoGroups*Protein  0 0 0 0 0 0 0 0 0 0 0 0 0 0 0 0 0 0 0 0 0 0 0 0 0 0 0 0 0 0 0 0 0 0 0 0 0 0 0
0 0 0 0 0 0 0 0 0 0 0 0 0 0 0 0 0 0 0 0 0 0 0 0 0 0 0 0 0 0 0 0 0 0 0 0 0 0 0 0 0 0 0 0 0 0 0 0 0 0 0 0 0 0 0 0 0 0 0 0 0 0 0 0 0
0 0 0 0 0 0 0 0 0 0 0 0 0 0 0 0 0 0 0 0 0 0 0 0 0 0 0 0 0 0 0 0 0 0 0 0 0 0 0 0 0 0 0 0 0 0 0 0 0 0 0 0 0 0 0 0 0 0 0 0 0 1 0 0 0
0 0 0 0 0 0 0 0 0 0 0 0 0 0 0;
Estimate "S100a4 Young Slope" Log_Total_GSH 1 Log_Total_GSH*TwoGroups 0 1Log_Total_GSH*Protein 0 0 0 0 0 0 0 0 0 0 0 0 0 0 0 0 0
0 0 0 0 0 0 0 0 0 0 0 0 0 0 0 0 0 0 0 0 0 0 0 0 0 0 0 0 0 0 0 0 0 0 0 0 0 0 0 0 0 0 0 0 0 0 0 0 0 0 0 0 0 0 0 0 0 1 0 0 0 0 0 0 0
0 0 0 0 0 0 0 0 0 0 Log_Total_GSH*TwoGroups*Protein  0 0 0 0 0 0 0 0 0 0 0 0 0 0 0 0 0 0 0 0 0 0 0 0 0 0 0 0 0 0 0 0 0 0 0 0 0 0
0 0 0 0 0 0 0 0 0 0 0 0 0 0 0 0 0 0 0 0 0 0 0 0 0 0 0 0 0 0 0 0 0 0 0 0 0 0 0 0 0 0 0 0 0 0 0 0 0 0 0 0 0 0 0 0 0 0 0 0 0 0 0 0 0
0 0 0 0 0 0 0 0 0 0 0 0 0 0 0 0 0 0 0 0 0 0 0 0 0 0 0 0 0 0 0 0 0 0 0 0 0 0 0 0 0 0 0 0 0 0 0 0 0 0 0 0 0 0 0 0 0 0 0 0 0 0 0 1 0
0 0 0 0 0 0 0 0 0 0 0 0 0 0 0 0;
Estimate "Sez6l2 Young Slope" Log_Total_GSH 1 Log_Total_GSH*TwoGroups 0 1Log_Total_GSH*Protein 0 0 0 0 0 0 0 0 0 0 0 0 0 0 0 0 0
0 0 0 0 0 0 0 0 0 0 0 0 0 0 0 0 0 0 0 0 0 0 0 0 0 0 0 0 0 0 0 0 0 0 0 0 0 0 0 0 0 0 0 0 0 0 0 0 0 0 0 0 0 0 0 0 0 0 1 0 0 0 0 0 0
0 0 0 0 0 0 0 0 0 0 Log_Total_GSH*TwoGroups*Protein  0 0 0 0 0 0 0 0 0 0 0 0 0 0 0 0 0 0 0 0 0 0 0 0 0 0 0 0 0 0 0 0 0 0 0 0 0 0
0 0 0 0 0 0 0 0 0 0 0 0 0 0 0 0 0 0 0 0 0 0 0 0 0 0 0 0 0 0 0 0 0 0 0 0 0 0 0 0 0 0 0 0 0 0 0 0 0 0 0 0 0 0 0 0 0 0 0 0 0 0 0 0 0
0 0 0 0 0 0 0 0 0 0 0 0 0 0 0 0 0 0 0 0 0 0 0 0 0 0 0 0 0 0 0 0 0 0 0 0 0 0 0 0 0 0 0 0 0 0 0 0 0 0 0 0 0 0 0 0 0 0 0 0 0 0 0 0 1
0 0 0 0 0 0 0 0 0 0 0 0 0 0 0 0;
Estimate "Snap29 Young Slope" Log_Total_GSH 1 Log_Total_GSH*TwoGroups 0 1Log_Total_GSH*Protein 0 0 0 0 0 0 0 0 0 0 0 0 0 0 0 0 0
0 0 0 0 0 0 0 0 0 0 0 0 0 0 0 0 0 0 0 0 0 0 0 0 0 0 0 0 0 0 0 0 0 0 0 0 0 0 0 0 0 0 0 0 0 0 0 0 0 0 0 0 0 0 0 0 0 0 0 1 0 0 0 0 0
0 0 0 0 0 0 0 0 0 0 Log_Total_GSH*TwoGroups*Protein  0 0 0 0 0 0 0 0 0 0 0 0 0 0 0 0 0 0 0 0 0 0 0 0 0 0 0 0 0 0 0 0 0 0 0 0 0 0
0 0 0 0 0 0 0 0 0 0 0 0 0 0 0 0 0 0 0 0 0 0 0 0 0 0 0 0 0 0 0 0 0 0 0 0 0 0 0 0 0 0 0 0 0 0 0 0 0 0 0 0 0 0 0 0 0 0 0 0 0 0 0 0 0
0 0 0 0 0 0 0 0 0 0 0 0 0 0 0 0 0 0 0 0 0 0 0 0 0 0 0 0 0 0 0 0 0 0 0 0 0 0 0 0 0 0 0 0 0 0 0 0 0 0 0 0 0 0 0 0 0 0 0 0 0 0 0 0 0
1 0 0 0 0 0 0 0 0 0 0 0 0 0 0 0;
Estimate "Tgfa Young Slope" Log_Total_GSH 1 Log_Total_GSH*TwoGroups 0 1Log_Total_GSH*Protein 0 0 0 0 0 0 0 0 0 0 0 0 0 0 0 0 0 0
0 0 0 0 0 0 0 0 0 0 0 0 0 0 0 0 0 0 0 0 0 0 0 0 0 0 0 0 0 0 0 0 0 0 0 0 0 0 0 0 0 0 0 0 0 0 0 0 0 0 0 0 0 0 0 0 0 0 0 1 0 0 0 0 0
0 0 0 0 0 0 0 0 0 Log_Total_GSH*TwoGroups*Protein  0 0 0 0 0 0 0 0 0 0 0 0 0 0 0 0 0 0 0 0 0 0 0 0 0 0 0 0 0 0 0 0 0 0 0 0 0 0 0
0 0 0 0 0 0 0 0 0 0 0 0 0 0 0 0 0 0 0 0 0 0 0 0 0 0 0 0 0 0 0 0 0 0 0 0 0 0 0 0 0 0 0 0 0 0 0 0 0 0 0 0 0 0 0 0 0 0 0 0 0 0 0 0 0
0 0 0 0 0 0 0 0 0 0 0 0 0 0 0 0 0 0 0 0 0 0 0 0 0 0 0 0 0 0 0 0 0 0 0 0 0 0 0 0 0 0 0 0 0 0 0 0 0 0 0 0 0 0 0 0 0 0 0 0 0 0 0 0 0
1 0 0 0 0 0 0 0 0 0 0 0 0 0 0;
Estimate "Tgfb1 Young Slope" Log_Total_GSH 1 Log_Total_GSH*TwoGroups 0 1Log_Total_GSH*Protein 0 0 0 0 0 0 0 0 0 0 0 0 0 0 0 0 0 0
0 0 0 0 0 0 0 0 0 0 0 0 0 0 0 0 0 0 0 0 0 0 0 0 0 0 0 0 0 0 0 0 0 0 0 0 0 0 0 0 0 0 0 0 0 0 0 0 0 0 0 0 0 0 0 0 0 0 0 0 1 0 0 0 0
0 0 0 0 0 0 0 0 0 Log_Total_GSH*TwoGroups*Protein  0 0 0 0 0 0 0 0 0 0 0 0 0 0 0 0 0 0 0 0 0 0 0 0 0 0 0 0 0 0 0 0 0 0 0 0 0 0 0
0 0 0 0 0 0 0 0 0 0 0 0 0 0 0 0 0 0 0 0 0 0 0 0 0 0 0 0 0 0 0 0 0 0 0 0 0 0 0 0 0 0 0 0 0 0 0 0 0 0 0 0 0 0 0 0 0 0 0 0 0 0 0 0 0
0 0 0 0 0 0 0 0 0 0 0 0 0 0 0 0 0 0 0 0 0 0 0 0 0 0 0 0 0 0 0 0 0 0 0 0 0 0 0 0 0 0 0 0 0 0 0 0 0 0 0 0 0 0 0 0 0 0 0 0 0 0 0 0 0
0 1 0 0 0 0 0 0 0 0 0 0 0 0 0;
Estimate "Tgfbr3 Young Slope" Log_Total_GSH 1 Log_Total_GSH*TwoGroups 0 1Log_Total_GSH*Protein 0 0 0 0 0 0 0 0 0 0 0 0 0 0 0 0 0
0 0 0 0 0 0 0 0 0 0 0 0 0 0 0 0 0 0 0 0 0 0 0 0 0 0 0 0 0 0 0 0 0 0 0 0 0 0 0 0 0 0 0 0 0 0 0 0 0 0 0 0 0 0 0 0 0 0 0 0 0 0 1 0 0
0 0 0 0 0 0 0 0 0 0 Log_Total_GSH*TwoGroups*Protein  0 0 0 0 0 0 0 0 0 0 0 0 0 0 0 0 0 0 0 0 0 0 0 0 0 0 0 0 0 0 0 0 0 0 0 0 0 0
0 0 0 0 0 0 0 0 0 0 0 0 0 0 0 0 0 0 0 0 0 0 0 0 0 0 0 0 0 0 0 0 0 0 0 0 0 0 0 0 0 0 0 0 0 0 0 0 0 0 0 0 0 0 0 0 0 0 0 0 0 0 0 0 0
0 0 0 0 0 0 0 0 0 0 0 0 0 0 0 0 0 0 0 0 0 0 0 0 0 0 0 0 0 0 0 0 0 0 0 0 0 0 0 0 0 0 0 0 0 0 0 0 0 0 0 0 0 0 0 0 0 0 0 0 0 0 0 0 0
0 0 0 1 0 0 0 0 0 0 0 0 0 0 0 0;
Estimate "Tnf Young Slope" Log_Total_GSH 1 Log_Total_GSH*TwoGroups 0 1Log_Total_GSH*Protein 0 0 0 0 0 0 0 0 0 0 0 0 0 0 0 0 0 0 0
0 0 0 0 0 0 0 0 0 0 0 0 0 0 0 0 0 0 0 0 0 0 0 0 0 0 0 0 0 0 0 0 0 0 0 0 0 0 0 0 0 0 0 0 0 0 0 0 0 0 0 0 0 0 0 0 0 0 0 0 0 1 0 0 0
0 0 0 0 0 0 0 0 Log_Total_GSH*TwoGroups*Protein  0 0 0 0 0 0 0 0 0 0 0 0 0 0 0 0 0 0 0 0 0 0 0 0 0 0 0 0 0 0 0 0 0 0 0 0 0 0 0 0
0 0 0 0 0 0 0 0 0 0 0 0 0 0 0 0 0 0 0 0 0 0 0 0 0 0 0 0 0 0 0 0 0 0 0 0 0 0 0 0 0 0 0 0 0 0 0 0 0 0 0 0 0 0 0 0 0 0 0 0 0 0 0 0 0
0 0 0 0 0 0 0 0 0 0 0 0 0 0 0 0 0 0 0 0 0 0 0 0 0 0 0 0 0 0 0 0 0 0 0 0 0 0 0 0 0 0 0 0 0 0 0 0 0 0 0 0 0 0 0 0 0 0 0 0 0 0 0 0 0
0 0 1 0 0 0 0 0 0 0 0 0 0 0;
Estimate "Tnfrsf11b Young Slope" Log_Total_GSH 1 Log_Total_GSH*TwoGroups 0 1Log_Total_GSH*Protein 0 0 0 0 0 0 0 0 0 0 0 0 0 0 0 0
0 0 0 0 0 0 0 0 0 0 0 0 0 0 0 0 0 0 0 0 0 0 0 0 0 0 0 0 0 0 0 0 0 0 0 0 0 0 0 0 0 0 0 0 0 0 0 0 0 0 0 0 0 0 0 0 0 0 0 0 0 0 0 0 0
```

```
1 0 0 0 0 0 0 0 0 0 0 Log_Total_GSH*TwoGroups*Protein  0 0 0 0 0 0 0 0 0 0 0 0 0 0 0 0 0 0 0 0 0 0 0 0 0 0 0 0 0 0 0 0 0 0 0 0 0
0 0 0 0 0 0 0 0 0 0 0 0 0 0 0 0 0 0 0 0 0 0 0 0 0 0 0 0 0 0 0 0 0 0 0 0 0 0 0 0 0 0 0 0 0 0 0 0 0 0 0 0 0 0 0 0 0 0 0 0 0 0 0 0 0
0 0 0 0 0 0 0 0 0 0 0 0 0 0 0 0 0 0 0 0 0 0 0 0 0 0 0 0 0 0 0 0 0 0 0 0 0 0 0 0 0 0 0 0 0 0 0 0 0 0 0 0 0 0 0 0 0 0 0 0 0 0 0 0 0
0 0 0 0 0 0 1 0 0 0 0 0 0 0 0 0 0;
Estimate "Tnfrsf12a Young Slope" Log_Total_GSH 1 Log_Total_GSH*TwoGroups 0 1Log_Total_GSH*Protein 0 0 0 0 0 0 0 0 0 0 0 0 0 0 0 0
0 0 0 0 0 0 0 0 0 0 0 0 0 0 0 0 0 0 0 0 0 0 0 0 0 0 0 0 0 0 0 0 0 0 0 0 0 0 0 0 0 0 0 0 0 0 0 0 0 0 0 0 0 0 0 0 0 0 0 0 0 0 0 0 0
0 1 0 0 0 0 0 0 0 0 0 Log_Total_GSH*TwoGroups*Protein  0 0 0 0 0 0 0 0 0 0 0 0 0 0 0 0 0 0 0 0 0 0 0 0 0 0 0 0 0 0 0 0 0 0 0 0 0
0 0 0 0 0 0 0 0 0 0 0 0 0 0 0 0 0 0 0 0 0 0 0 0 0 0 0 0 0 0 0 0 0 0 0 0 0 0 0 0 0 0 0 0 0 0 0 0 0 0 0 0 0 0 0 0 0 0 0 0 0 0 0 0 0
0 0 0 0 0 0 0 0 0 0 0 0 0 0 0 0 0 0 0 0 0 0 0 0 0 0 0 0 0 0 0 0 0 0 0 0 0 0 0 0 0 0 0 0 0 0 0 0 0 0 0 0 0 0 0 0 0 0 0 0 0 0 0 0 0
0 0 0 0 0 0 0 1 0 0 0 0 0 0 0 0 0;
Estimate "Tnfsf12 Young Slope" Log_Total_GSH 1 Log_Total_GSH*TwoGroups 0 1Log_Total_GSH*Protein 0 0 0 0 0 0 0 0 0 0 0 0 0 0 0 0 0
0 0 0 0 0 0 0 0 0 0 0 0 0 0 0 0 0 0 0 0 0 0 0 0 0 0 0 0 0 0 0 0 0 0 0 0 0 0 0 0 0 0 0 0 0 0 0 0 0 0 0 0 0 0 0 0 0 0 0 0 0 0 0 0 0
0 1 0 0 0 0 0 0 0 0 Log_Total_GSH*TwoGroups*Protein  0 0 0 0 0 0 0 0 0 0 0 0 0 0 0 0 0 0 0 0 0 0 0 0 0 0 0 0 0 0 0 0 0 0 0 0 0 0
0 0 0 0 0 0 0 0 0 0 0 0 0 0 0 0 0 0 0 0 0 0 0 0 0 0 0 0 0 0 0 0 0 0 0 0 0 0 0 0 0 0 0 0 0 0 0 0 0 0 0 0 0 0 0 0 0 0 0 0 0 0 0 0 0
0 0 0 0 0 0 0 0 0 0 0 0 0 0 0 0 0 0 0 0 0 0 0 0 0 0 0 0 0 0 0 0 0 0 0 0 0 0 0 0 0 0 0 0 0 0 0 0 0 0 0 0 0 0 0 0 0 0 0 0 0 0 0 0 0
0 0 0 0 0 0 0 1 0 0 0 0 0 0 0 0;
Estimate "Tnni3 Young Slope" Log_Total_GSH 1 Log_Total_GSH*TwoGroups 0 1Log_Total_GSH*Protein 0 0 0 0 0 0 0 0 0 0 0 0 0 0 0 0 0 0
0 0 0 0 0 0 0 0 0 0 0 0 0 0 0 0 0 0 0 0 0 0 0 0 0 0 0 0 0 0 0 0 0 0 0 0 0 0 0 0 0 0 0 0 0 0 0 0 0 0 0 0 0 0 0 0 0 0 0 0 0 0 0 0 0
0 1 0 0 0 0 0 0 0 Log_Total_GSH*TwoGroups*Protein  0 0 0 0 0 0 0 0 0 0 0 0 0 0 0 0 0 0 0 0 0 0 0 0 0 0 0 0 0 0 0 0 0 0 0 0 0 0 0
0 0 0 0 0 0 0 0 0 0 0 0 0 0 0 0 0 0 0 0 0 0 0 0 0 0 0 0 0 0 0 0 0 0 0 0 0 0 0 0 0 0 0 0 0 0 0 0 0 0 0 0 0 0 0 0 0 0 0 0 0 0 0 0 0
0 0 0 0 0 0 0 0 0 0 0 0 0 0 0 0 0 0 0 0 0 0 0 0 0 0 0 0 0 0 0 0 0 0 0 0 0 0 0 0 0 0 0 0 0 0 0 0 0 0 0 0 0 0 0 0 0 0 0 0 0 0 0 0 0
0 0 0 0 0 0 0 1 0 0 0 0 0 0 0;
Estimate "Tnr Young Slope" Log_Total_GSH 1 Log_Total_GSH*TwoGroups 0 1Log_Total_GSH*Protein 0 0 0 0 0 0 0 0 0 0 0 0 0 0 0 0 0 0 0
0 0 0 0 0 0 0 0 0 0 0 0 0 0 0 0 0 0 0 0 0 0 0 0 0 0 0 0 0 0 0 0 0 0 0 0 0 0 0 0 0 0 0 0 0 0 0 0 0 0 0 0 0 0 0 0 0 0 0 0 0 0 0 0 0
0 1 0 0 0 0 0 0 Log_Total_GSH*TwoGroups*Protein  0 0 0 0 0 0 0 0 0 0 0 0 0 0 0 0 0 0 0 0 0 0 0 0 0 0 0 0 0 0 0 0 0 0 0 0 0 0 0 0
0 0 0 0 0 0 0 0 0 0 0 0 0 0 0 0 0 0 0 0 0 0 0 0 0 0 0 0 0 0 0 0 0 0 0 0 0 0 0 0 0 0 0 0 0 0 0 0 0 0 0 0 0 0 0 0 0 0 0 0 0 0 0 0 0
0 0 0 0 0 0 0 0 0 0 0 0 0 0 0 0 0 0 0 0 0 0 0 0 0 0 0 0 0 0 0 0 0 0 0 0 0 0 0 0 0 0 0 0 0 0 0 0 0 0 0 0 0 0 0 0 0 0 0 0 0 0 0 0 0
0 0 0 0 0 0 0 1 0 0 0 0 0 0;
Estimate "Tpp1 Young Slope" Log_Total_GSH 1 Log_Total_GSH*TwoGroups 0 1Log_Total_GSH*Protein 0 0 0 0 0 0 0 0 0 0 0 0 0 0 0 0 0 0
0 0 0 0 0 0 0 0 0 0 0 0 0 0 0 0 0 0 0 0 0 0 0 0 0 0 0 0 0 0 0 0 0 0 0 0 0 0 0 0 0 0 0 0 0 0 0 0 0 0 0 0 0 0 0 0 0 0 0 0 0 0 0 0 0
0 0 0 1 0 0 0 0 0 Log_Total_GSH*TwoGroups*Protein  0 0 0 0 0 0 0 0 0 0 0 0 0 0 0 0 0 0 0 0 0 0 0 0 0 0 0 0 0 0 0 0 0 0 0 0 0 0 0
0 0 0 0 0 0 0 0 0 0 0 0 0 0 0 0 0 0 0 0 0 0 0 0 0 0 0 0 0 0 0 0 0 0 0 0 0 0 0 0 0 0 0 0 0 0 0 0 0 0 0 0 0 0 0 0 0 0 0 0 0 0 0 0 0
0 0 0 0 0 0 0 0 0 0 0 0 0 0 0 0 0 0 0 0 0 0 0 0 0 0 0 0 0 0 0 0 0 0 0 0 0 0 0 0 0 0 0 0 0 0 0 0 0 0 0 0 0 0 0 0 0 0 0 0 0 0 0 0 0
0 0 0 0 0 0 0 0 0 1 0 0 0 0 0;
Estimate "Vegfd Young Slope" Log_Total_GSH 1 Log_Total_GSH*TwoGroups 0 1Log_Total_GSH*Protein 0 0 0 0 0 0 0 0 0 0 0 0 0 0 0 0 0 0
0 0 0 0 0 0 0 0 0 0 0 0 0 0 0 0 0 0 0 0 0 0 0 0 0 0 0 0 0 0 0 0 0 0 0 0 0 0 0 0 0 0 0 0 0 0 0 0 0 0 0 0 0 0 0 0 0 0 0 0 0 0 0 0 0
0 0 0 0 1 0 0 0 0 Log_Total_GSH*TwoGroups*Protein  0 0 0 0 0 0 0 0 0 0 0 0 0 0 0 0 0 0 0 0 0 0 0 0 0 0 0 0 0 0 0 0 0 0 0 0 0 0 0
0 0 0 0 0 0 0 0 0 0 0 0 0 0 0 0 0 0 0 0 0 0 0 0 0 0 0 0 0 0 0 0 0 0 0 0 0 0 0 0 0 0 0 0 0 0 0 0 0 0 0 0 0 0 0 0 0 0 0 0 0 0 0 0 0
0 0 0 0 0 0 0 0 0 0 0 0 0 0 0 0 0 0 0 0 0 0 0 0 0 0 0 0 0 0 0 0 0 0 0 0 0 0 0 0 0 0 0 0 0 0 0 0 0 0 0 0 0 0 0 0 0 0 0 0 0 0 0 0 0
0 0 0 0 0 0 0 0 0 0 1 0 0 0 0;
Estimate "Vsig2 Young Slope" Log_Total_GSH 1 Log_Total_GSH*TwoGroups 0 1Log_Total_GSH*Protein 0 0 0 0 0 0 0 0 0 0 0 0 0 0 0 0 0 0
0 0 0 0 0 0 0 0 0 0 0 0 0 0 0 0 0 0 0 0 0 0 0 0 0 0 0 0 0 0 0 0 0 0 0 0 0 0 0 0 0 0 0 0 0 0 0 0 0 0 0 0 0 0 0 0 0 0 0 0 0 0 0 0 0
0 0 0 0 0 1 0 0 0 Log_Total_GSH*TwoGroups*Protein  0 0 0 0 0 0 0 0 0 0 0 0 0 0 0 0 0 0 0 0 0 0 0 0 0 0 0 0 0 0 0 0 0 0 0 0 0 0 0
0 0 0 0 0 0 0 0 0 0 0 0 0 0 0 0 0 0 0 0 0 0 0 0 0 0 0 0 0 0 0 0 0 0 0 0 0 0 0 0 0 0 0 0 0 0 0 0 0 0 0 0 0 0 0 0 0 0 0 0 0 0 0 0 0
0 0 0 0 0 0 0 0 0 0 0 0 0 0 0 0 0 0 0 0 0 0 0 0 0 0 0 0 0 0 0 0 0 0 0 0 0 0 0 0 0 0 0 0 0 0 0 0 0 0 0 0 0 0 0 0 0 0 0 0 0 0 0 0 0
0 0 0 0 0 0 0 0 0 0 0 1 0 0 0;
Estimate "Wfikkn2 Young Slope" Log_Total_GSH 1 Log_Total_GSH*TwoGroups 0 1Log_Total_GSH*Protein 0 0 0 0 0 0 0 0 0 0 0 0 0 0 0 0 0
0 0 0 0 0 0 0 0 0 0 0 0 0 0 0 0 0 0 0 0 0 0 0 0 0 0 0 0 0 0 0 0 0 0 0 0 0 0 0 0 0 0 0 0 0 0 0 0 0 0 0 0 0 0 0 0 0 0 0 0 0 0 0 0 0
0 0 0 0 0 0 0 1 0 0 Log_Total_GSH*TwoGroups*Protein  0 0 0 0 0 0 0 0 0 0 0 0 0 0 0 0 0 0 0 0 0 0 0 0 0 0 0 0 0 0 0 0 0 0 0 0 0 0
0 0 0 0 0 0 0 0 0 0 0 0 0 0 0 0 0 0 0 0 0 0 0 0 0 0 0 0 0 0 0 0 0 0 0 0 0 0 0 0 0 0 0 0 0 0 0 0 0 0 0 0 0 0 0 0 0 0 0 0 0 0 0 0 0
0 0 0 0 0 0 0 0 0 0 0 0 0 0 0 0 0 0 0 0 0 0 0 0 0 0 0 0 0 0 0 0 0 0 0 0 0 0 0 0 0 0 0 0 0 0 0 0 0 0 0 0 0 0 0 0 0 0 0 0 0 0 0 0 0
0 0 0 0 0 0 0 0 0 0 0 0 0 1 0 0;
Estimate "Wisp1 Young Slope" Log_Total_GSH 1 Log_Total_GSH*TwoGroups 0 1Log_Total_GSH*Protein 0 0 0 0 0 0 0 0 0 0 0 0 0 0 0 0 0 0
0 0 0 0 0 0 0 0 0 0 0 0 0 0 0 0 0 0 0 0 0 0 0 0 0 0 0 0 0 0 0 0 0 0 0 0 0 0 0 0 0 0 0 0 0 0 0 0 0 0 0 0 0 0 0 0 0 0 0 0 0 0 0 0 0
0 0 0 0 0 0 0 1 0 Log_Total_GSH*TwoGroups*Protein  0 0 0 0 0 0 0 0 0 0 0 0 0 0 0 0 0 0 0 0 0 0 0 0 0 0 0 0 0 0 0 0 0 0 0 0 0 0 0
0 0 0 0 0 0 0 0 0 0 0 0 0 0 0 0 0 0 0 0 0 0 0 0 0 0 0 0 0 0 0 0 0 0 0 0 0 0 0 0 0 0 0 0 0 0 0 0 0 0 0 0 0 0 0 0 0 0 0 0 0 0 0 0 0
0 0 0 0 0 0 0 0 0 0 0 0 0 0 0 0 0 0 0 0 0 0 0 0 0 0 0 0 0 0 0 0 0 0 0 0 0 0 0 0 0 0 0 0 0 0 0 0 0 0 0 0 0 0 0 0 0 0 0 0 0 0 0 0 0
0 0 0 0 0 0 0 0 0 0 0 0 0 1 0;
Estimate "Yes1 Young Slope" Log_Total_GSH 1 Log_Total_GSH*TwoGroups 0 1Log_Total_GSH*Protein 0 0 0 0 0 0 0 0 0 0 0 0 0 0 0 0 0 0
0 0 0 0 0 0 0 0 0 0 0 0 0 0 0 0 0 0 0 0 0 0 0 0 0 0 0 0 0 0 0 0 0 0 0 0 0 0 0 0 0 0 0 0 0 0 0 0 0 0 0 0 0 0 0 0 0 0 0 0 0 0 0 0 0
0 0 0 0 0 0 0 0 1 Log_Total_GSH*TwoGroups*Protein  0 0 0 0 0 0 0 0 0 0 0 0 0 0 0 0 0 0 0 0 0 0 0 0 0 0 0 0 0 0 0 0 0 0 0 0 0 0 0
0 0 0 0 0 0 0 0 0 0 0 0 0 0 0 0 0 0 0 0 0 0 0 0 0 0 0 0 0 0 0 0 0 0 0 0 0 0 0 0 0 0 0 0 0 0 0 0 0 0 0 0 0 0 0 0 0 0 0 0 0 0 0 0 0
0 0 0 0 0 0 0 0 0 0 0 0 0 0 0 0 0 0 0 0 0 0 0 0 0 0 0 0 0 0 0 0 0 0 0 0 0 0 0 0 0 0 0 0 0 0 0 0 0 0 0 0 0 0 0 0 0 0 0 0 0 0 0 0 0
0 0 0 0 0 0 0 0 0 0 0 0 0 0 1;


RUN;
```

Stereotypy and Proteomics

```
DATA Olink_And_ST_20241125;
INPUT  SubjectID &$ Log_SumAllST_1 TwoGroups &$16. Protein &$9. Norm_NPX; Lines;
StEt 01  2.07944154167984  old  Clmp  2.8683
StEt 01  2.07944154167984  old  Matn2  -0.28036
StEt 01  2.07944154167984  old  Cpe  2.25311
StEt 01  2.07944154167984  old  Gcg  -0.61261
StEt 01  2.07944154167984  old  Gdnf  -3.82092
StEt 01  2.07944154167984  old  Yes1  -2.12839
StEt 01  2.07944154167984  old  Il17a  -1.68211
StEt 01  2.07944154167984  old  Foxo1  -2.86781
StEt 01  2.07944154167984  old  Tnfrsf11b  0.48707
StEt 01  2.07944154167984  old  Tgfb1  -0.17296
StEt 01  2.07944154167984  old  Pla2g4a  1.66151
StEt 01  2.07944154167984  old  Il6  -3.95119
StEt 01  2.07944154167984  old  Prdx5  -1.08282
StEt 01  2.07944154167984  old  Tgfa  2.72297
StEt 01  2.07944154167984  old  Ccl5  -1.62222
StEt 01  2.07944154167984  old  Epo  -1.44117
StEt 01  2.07944154167984  old  Axin1  -2.66709
StEt 01  2.07944154167984  old  Fst  2.94974
StEt 01  2.07944154167984  old  Rgma  -0.65767
StEt 01  2.07944154167984  old  Nadk  2.90602
StEt 01  2.07944154167984  old  Tnni3  8.91236
StEt 01  2.07944154167984  old  Notch3  2.09404
StEt 01  2.07944154167984  old  Snap29  1.48582
StEt 01  2.07944154167984  old  Cntn1  5.34002
StEt 01  2.07944154167984  old  Clstn2  -2.4647
StEt 01  2.07944154167984  old  S100a4  6.0986
StEt 01  2.07944154167984  old  Ca13  -3.16719
StEt 01  2.07944154167984  old  Mia  1.26241
StEt 01  2.07944154167984  old  Cant1  -1.59288
StEt 01  2.07944154167984  old  Kitlg  -4.81995
StEt 01  2.07944154167984  old  Gfra1  -1.28563
StEt 01  2.07944154167984  old  Ppp1r2  -2.6189
StEt 01  2.07944154167984  old  Adam23  -1.14121
StEt 01  2.07944154167984  old  Itgb6  -2.55977
StEt 01  2.07944154167984  old  Cyr61  0.67965
StEt 01  2.07944154167984  old  Dlk1  3.93637
StEt 01  2.07944154167984  old  Ahr  -2.76967
StEt 01  2.07944154167984  old  Ccl2  6.35886
StEt 01  2.07944154167984  old  Eno2  -0.87518
StEt 01  2.07944154167984  old  Plin1  -2.62681
StEt 01  2.07944154167984  old  Wfikkn2  2.58723
StEt 01  2.07944154167984  old  Flrt2  0.00058
StEt 01  2.07944154167984  old  Qdpr  -0.31268
StEt 01  2.07944154167984  old  Fas  1.79292
StEt 01  2.07944154167984  old  Erbb4  0.87806
StEt 01  2.07944154167984  old  Riox2  -0.49543
StEt 01  2.07944154167984  old  Plxna4  -2.65013
StEt 01  2.07944154167984  old  Epcam  -1.07313
StEt 01  2.07944154167984  old  Ccl3  0.40848
StEt 01  2.07944154167984  old  Crim1  -1.35769
StEt 01  2.07944154167984  old  Vsig2  1.32577
StEt 01  2.07944154167984  old  Hgf  -1.14655
StEt 01  2.07944154167984  old  Sez6l2  2.63902
StEt 01  2.07944154167984  old  Il1a  0.07517
StEt 01  2.07944154167984  old  Il23r  4.62269
StEt 01  2.07944154167984  old  Dll1  1.41911
StEt 01  2.07944154167984  old  Ddah1  -2.07286
StEt 01  2.07944154167984  old  Il10  -1.96483
StEt 01  2.07944154167984  old  Tnfrsf12a  1.81618
StEt 01  2.07944154167984  old  Acvrl1  0.26476
StEt 01  2.07944154167984  old  Lgmn  1.47517
StEt 01  2.07944154167984  old  Csf2  -2.60837
StEt 01  2.07944154167984  old  Cxcl9  1.84353
StEt 01  2.07944154167984  old  Map2k6  1.0381
StEt 01  2.07944154167984  old  Itgb1bp2  -5.06866
StEt 01  2.07944154167984  old  Il17f  -4.80761
StEt 01  2.07944154167984  old  Il1b  -3.51375
StEt 01  2.07944154167984  old  Casp3  1.85158
StEt 01  2.07944154167984  old  Apbb1ip  -3.8027
StEt 01  2.07944154167984  old  Wisp1  0.90946
StEt 01  2.07944154167984  old  Cdh6  0.95205
StEt 01  2.07944154167984  old  Pdgfb  0.39275
StEt 01  2.07944154167984  old  Igsf3  -0.35396
StEt 01  2.07944154167984  old  Tgfbr3  0.53982
StEt 01  2.07944154167984  old  Cxcl1  5.75837
StEt 01  2.07944154167984  old  Pak4  -4.01841
StEt 01  2.07944154167984  old  Cntn4  1.20574
StEt 01  2.07944154167984  old  Ghrl  2.61238
StEt 01  2.07944154167984  old  Lpl  0.58407
StEt 01  2.07944154167984  old  Fstl3  1.58249
StEt 01  2.07944154167984  old  Dctn2  -1.71411
StEt 01  2.07944154167984  old  Il5  -3.35842
StEt 01  2.07944154167984  old  Eda2r  1.26479
StEt 01  2.07944154167984  old  Ntf3  -2.33484
StEt 01  2.07944154167984  old  Tnfsf12  0.25906
StEt 01  2.07944154167984  old  Ccl20  6.20999
StEt 01  2.07944154167984  old  Fli1  -4.30614
StEt 01  2.07944154167984  old  Tpp1  3.18126
```

StEt	01	2.07944154167984	old	Tnr	-0.39026
StEt	01	2.07944154167984	old	Vegfd	-0.58948
StEt	01	2.07944154167984	old	Parp1	-1.36957
StEt	01	2.07944154167984	old	Tnf	-3.28854
Stet	02	4.14313472639153	old	Clmp	3.05087
Stet	02	4.14313472639153	old	Matn2	0.63205
Stet	02	4.14313472639153	old	Cpe	2.12765
Stet	02	4.14313472639153	old	Gcg	-2.13358
Stet	02	4.14313472639153	old	Gdnf	-3.93587
Stet	02	4.14313472639153	old	Yes1	-1.23876
Stet	02	4.14313472639153	old	Il17a	-2.09183
Stet	02	4.14313472639153	old	Foxo1	-2.30232
Stet	02	4.14313472639153	old	Tnfrsf11b	0.62819
Stet	02	4.14313472639153	old	Tgfb1	-0.48833
Stet	02	4.14313472639153	old	Pla2g4a	2.56277
Stet	02	4.14313472639153	old	Il6	-4.19358
Stet	02	4.14313472639153	old	Prdx5	-0.71358
Stet	02	4.14313472639153	old	Tgfa	2.2597
Stet	02	4.14313472639153	old	Ccl5	-1.39874
Stet	02	4.14313472639153	old	Epo	0.17692
Stet	02	4.14313472639153	old	Axin1	-2.40962
Stet	02	4.14313472639153	old	Fst	2.64017
Stet	02	4.14313472639153	old	Rgma	-0.73759
Stet	02	4.14313472639153	old	Nadk	4.3223
Stet	02	4.14313472639153	old	Tnni3	9.30576
Stet	02	4.14313472639153	old	Notch3	1.73709
Stet	02	4.14313472639153	old	Snap29	2.49932
Stet	02	4.14313472639153	old	Cntn1	5.09719
Stet	02	4.14313472639153	old	Clstn2	-2.26892
Stet	02	4.14313472639153	old	S100a4	5.99458
Stet	02	4.14313472639153	old	Ca13	-3.0006
Stet	02	4.14313472639153	old	Mia	1.047
Stet	02	4.14313472639153	old	Cant1	-1.78886
Stet	02	4.14313472639153	old	Kitlg	-5.17391
Stet	02	4.14313472639153	old	Gfra1	-1.975
Stet	02	4.14313472639153	old	Ppp1r2	-2.11522
Stet	02	4.14313472639153	old	Adam23	-1.06003
Stet	02	4.14313472639153	old	Itgb6	-3.07225
Stet	02	4.14313472639153	old	Cyr61	0.47948
Stet	02	4.14313472639153	old	Dlk1	0.78281
Stet	02	4.14313472639153	old	Ahr	-1.74437
Stet	02	4.14313472639153	old	Ccl2	6.5617
Stet	02	4.14313472639153	old	Eno2	-0.9463
Stet	02	4.14313472639153	old	Plin1	-1.02285
Stet	02	4.14313472639153	old	Wfikkn2	1.76069
Stet	02	4.14313472639153	old	Flrt2	0.13895
Stet	02	4.14313472639153	old	Qdpr	1.17834
Stet	02	4.14313472639153	old	Fas	1.52895
Stet	02	4.14313472639153	old	Erbb4	0.90071
Stet	02	4.14313472639153	old	Riox2	1.03634
Stet	02	4.14313472639153	old	Plxna4	-2.94623
Stet	02	4.14313472639153	old	Epcam	-1.06176
Stet	02	4.14313472639153	old	Ccl3	-1.04699
Stet	02	4.14313472639153	old	Crim1	-1.84959
Stet	02	4.14313472639153	old	Vsig2	1.53819
Stet	02	4.14313472639153	old	Hgf	-1.07291
Stet	02	4.14313472639153	old	Sez6l2	2.2669
Stet	02	4.14313472639153	old	Il1a	0.21146
Stet	02	4.14313472639153	old	Il23r	4.09248
Stet	02	4.14313472639153	old	Dll1	1.30379
Stet	02	4.14313472639153	old	Ddah1	-0.42534
Stet	02	4.14313472639153	old	Il10	-1.98081
Stet	02	4.14313472639153	old	Tnfrsf12a	2.2169
Stet	02	4.14313472639153	old	Acvrl1	0.10168
Stet	02	4.14313472639153	old	Lgmn	1.97691
Stet	02	4.14313472639153	old	Csf2	-3.42544
Stet	02	4.14313472639153	old	Cxcl9	1.0041
Stet	02	4.14313472639153	old	Map2k6	1.35305
Stet	02	4.14313472639153	old	Itgb1bp2	-5.29071
Stet	02	4.14313472639153	old	Il17f	-5.16294
Stet	02	4.14313472639153	old	Il1b	-3.46715
Stet	02	4.14313472639153	old	Casp3	2.53604
Stet	02	4.14313472639153	old	Apbb1ip	-3.55452
Stet	02	4.14313472639153	old	Wisp1	0.88791
Stet	02	4.14313472639153	old	Cdh6	0.8346
Stet	02	4.14313472639153	old	Pdgfb	1.32723
Stet	02	4.14313472639153	old	Igsf3	-0.72057
Stet	02	4.14313472639153	old	Tgfbr3	0.42348
Stet	02	4.14313472639153	old	Cxcl1	4.37285
Stet	02	4.14313472639153	old	Pak4	-3.65676
Stet	02	4.14313472639153	old	Cntn4	0.48381
Stet	02	4.14313472639153	old	Ghrl	2.70304
Stet	02	4.14313472639153	old	Lpl	0.69613
Stet	02	4.14313472639153	old	Fstl3	1.71683
Stet	02	4.14313472639153	old	Dctn2	-1.51861
Stet	02	4.14313472639153	old	Il5	-3.15302
Stet	02	4.14313472639153	old	Eda2r	0.57831
Stet	02	4.14313472639153	old	Ntf3	-2.47061
Stet	02	4.14313472639153	old	Tnfsf12	-0.14764
Stet	02	4.14313472639153	old	Ccl20	4.48003
Stet	02	4.14313472639153	old	Fli1	-4.58615
Stet	02	4.14313472639153	old	Tpp1	3.27324
Stet	02	4.14313472639153	old	Tnr	0.02873
Stet	02	4.14313472639153	old	Vegfd	-0.73882
Stet	02	4.14313472639153	old	Parp1	-1.67648

Stet 02	4.14313472639153	old	Tnf	-3.09172
StEt 03	2.39789527279837	old	Clmp	3.34347
StEt 03	2.39789527279837	old	Matn2	0.66087
StEt 03	2.39789527279837	old	Cpe	2.25543
StEt 03	2.39789527279837	old	Gcg	0.45925
StEt 03	2.39789527279837	old	Gdnf	-3.90842
StEt 03	2.39789527279837	old	Yes1	-2.10034
StEt 03	2.39789527279837	old	Il17a	-1.19419
StEt 03	2.39789527279837	old	Foxo1	-3.13255
StEt 03	2.39789527279837	old	Tnfrsf11b	1.21489
StEt 03	2.39789527279837	old	Tgfb1	-0.11431
StEt 03	2.39789527279837	old	Pla2g4a	3.39979
StEt 03	2.39789527279837	old	Il6	-3.75407
StEt 03	2.39789527279837	old	Prdx5	-1.23139
StEt 03	2.39789527279837	old	Tgfa	1.84402
StEt 03	2.39789527279837	old	Ccl5	-1.61224
StEt 03	2.39789527279837	old	Epo	0.57383
StEt 03	2.39789527279837	old	Axin1	-2.77797
StEt 03	2.39789527279837	old	Fst	3.56543
StEt 03	2.39789527279837	old	Rgma	-0.81699
StEt 03	2.39789527279837	old	Nadk	1.16004
StEt 03	2.39789527279837	old	Tnni3	8.23016
StEt 03	2.39789527279837	old	Notch3	2.06501
StEt 03	2.39789527279837	old	Snap29	2.05091
StEt 03	2.39789527279837	old	Cntn1	5.41652
StEt 03	2.39789527279837	old	Clstn2	-2.1992
StEt 03	2.39789527279837	old	S100a4	5.73276
StEt 03	2.39789527279837	old	Ca13	-3.37403
StEt 03	2.39789527279837	old	Mia	1.16275
StEt 03	2.39789527279837	old	Cant1	-1.28717
StEt 03	2.39789527279837	old	Kitlg	-4.77739
StEt 03	2.39789527279837	old	Gfra1	-2.11539
StEt 03	2.39789527279837	old	Ppp1r2	-2.78485
StEt 03	2.39789527279837	old	Adam23	-0.7549
StEt 03	2.39789527279837	old	Itgb6	-2.15703
StEt 03	2.39789527279837	old	Cyr61	1.38393
StEt 03	2.39789527279837	old	Dlk1	0.61485
StEt 03	2.39789527279837	old	Ahr	-3.12868
StEt 03	2.39789527279837	old	Ccl2	5.81401
StEt 03	2.39789527279837	old	Eno2	-0.6037
StEt 03	2.39789527279837	old	Plin1	-2.88109
StEt 03	2.39789527279837	old	Wfikkn2	2.11634
StEt 03	2.39789527279837	old	Flrt2	0.29344
StEt 03	2.39789527279837	old	Qdpr	-1.57552
StEt 03	2.39789527279837	old	Fas	1.58556
StEt 03	2.39789527279837	old	Erbb4	1.3025
StEt 03	2.39789527279837	old	Riox2	-2.25249
StEt 03	2.39789527279837	old	Plxna4	0.00154
StEt 03	2.39789527279837	old	Epcam	-1.29581
StEt 03	2.39789527279837	old	Ccl3	-0.884
StEt 03	2.39789527279837	old	Crim1	-1.88958
StEt 03	2.39789527279837	old	Vsig2	1.59367
StEt 03	2.39789527279837	old	Hgf	-1.01478
StEt 03	2.39789527279837	old	Sez6l2	3.09585
StEt 03	2.39789527279837	old	Il1a	1.09365
StEt 03	2.39789527279837	old	Il23r	4.25662
StEt 03	2.39789527279837	old	Dll1	1.61314
StEt 03	2.39789527279837	old	Ddah1	-3.54132
StEt 03	2.39789527279837	old	Il10	-2.11933
StEt 03	2.39789527279837	old	Tnfrsf12a	2.02742
StEt 03	2.39789527279837	old	Acvrl1	0.44909
StEt 03	2.39789527279837	old	Lgmn	1.57327
StEt 03	2.39789527279837	old	Csf2	-2.99764
StEt 03	2.39789527279837	old	Cxcl9	1.24003
StEt 03	2.39789527279837	old	Map2k6	1.72816
StEt 03	2.39789527279837	old	Itgb1bp2	-5.40578
StEt 03	2.39789527279837	old	Il17f	-4.93621
StEt 03	2.39789527279837	old	Il1b	-3.30839
StEt 03	2.39789527279837	old	Casp3	2.11764
StEt 03	2.39789527279837	old	Apbb1ip	-3.53737
StEt 03	2.39789527279837	old	Wisp1	0.77798
StEt 03	2.39789527279837	old	Cdh6	1.08023
StEt 03	2.39789527279837	old	Pdgfb	1.63406
StEt 03	2.39789527279837	old	Igsf3	-0.30871
StEt 03	2.39789527279837	old	Tgfbr3	0.72284
StEt 03	2.39789527279837	old	Cxcl1	5.09483
StEt 03	2.39789527279837	old	Pak4	-3.81065
StEt 03	2.39789527279837	old	Cntn4	0.42184
StEt 03	2.39789527279837	old	Ghrl	2.06717
StEt 03	2.39789527279837	old	Lpl	0.64366
StEt 03	2.39789527279837	old	Fstl3	1.93628
StEt 03	2.39789527279837	old	Dctn2	-2.26418
StEt 03	2.39789527279837	old	Il5	-2.6995
StEt 03	2.39789527279837	old	Eda2r	0.8805
StEt 03	2.39789527279837	old	Ntf3	-1.13016
StEt 03	2.39789527279837	old	Tnfsf12	-0.20807
StEt 03	2.39789527279837	old	Ccl20	5.49602
StEt 03	2.39789527279837	old	Fli1	-4.54442
StEt 03	2.39789527279837	old	Tpp1	3.66258
StEt 03	2.39789527279837	old	Tnr	0.64117
StEt 03	2.39789527279837	old	Vegfd	-0.43825
StEt 03	2.39789527279837	old	Parp1	-2.07371
StEt 03	2.39789527279837	old	Tnf	-3.1531
StEt 04	3.09104245335832	old	Clmp	2.62312
StEt 04	3.09104245335832	old	Matn2	0.39493

StEt 04	3.09104245335832	old	Cpe	1.88616
StEt 04	3.09104245335832	old	Gcg	0.12832
StEt 04	3.09104245335832	old	Gdnf	-4.5977
StEt 04	3.09104245335832	old	Yes1	-0.05845
StEt 04	3.09104245335832	old	Il17a	-2.0778
StEt 04	3.09104245335832	old	Foxo1	-2.23253
StEt 04	3.09104245335832	old	Tnfrsf11b	-0.24401
StEt 04	3.09104245335832	old	Tgfb1	-0.28483
StEt 04	3.09104245335832	old	Pla2g4a	3.36659
StEt 04	3.09104245335832	old	Il6	-5.20267
StEt 04	3.09104245335832	old	Prdx5	0.64364
StEt 04	3.09104245335832	old	Tgfa	2.83293
StEt 04	3.09104245335832	old	Ccl5	-2.23972
StEt 04	3.09104245335832	old	Epo	-0.65893
StEt 04	3.09104245335832	old	Axin1	-1.58248
StEt 04	3.09104245335832	old	Fst	3.02473
StEt 04	3.09104245335832	old	Rgma	-0.89883
StEt 04	3.09104245335832	old	Nadk	3.75466
StEt 04	3.09104245335832	old	Tnni3	10.37774
StEt 04	3.09104245335832	old	Notch3	1.33509
StEt 04	3.09104245335832	old	Snap29	2.83475
StEt 04	3.09104245335832	old	Cntn1	4.48659
StEt 04	3.09104245335832	old	Clstn2	-3.0104
StEt 04	3.09104245335832	old	S100a4	5.53599
StEt 04	3.09104245335832	old	Ca13	-1.10455
StEt 04	3.09104245335832	old	Mia	0.40292
StEt 04	3.09104245335832	old	Cant1	-1.4367
StEt 04	3.09104245335832	old	Kitlg	-5.43481
StEt 04	3.09104245335832	old	Gfra1	-2.40971
StEt 04	3.09104245335832	old	Ppp1r2	-0.91027
StEt 04	3.09104245335832	old	Adam23	-1.64209
StEt 04	3.09104245335832	old	Itgb6	-3.6265
StEt 04	3.09104245335832	old	Cyr61	0.62093
StEt 04	3.09104245335832	old	Dlk1	-0.12399
StEt 04	3.09104245335832	old	Ahr	-2.26501
StEt 04	3.09104245335832	old	Ccl2	5.27498
StEt 04	3.09104245335832	old	Eno2	-0.86411
StEt 04	3.09104245335832	old	Plin1	1.17651
StEt 04	3.09104245335832	old	Wfikkn2	1.04952
StEt 04	3.09104245335832	old	Flrt2	1.8702
StEt 04	3.09104245335832	old	Qdpr	0.50989
StEt 04	3.09104245335832	old	Fas	1.38445
StEt 04	3.09104245335832	old	Erbb4	0.38031
StEt 04	3.09104245335832	old	Riox2	0.98442
StEt 04	3.09104245335832	old	Plxna4	-0.74947
StEt 04	3.09104245335832	old	Epcam	-1.70303
StEt 04	3.09104245335832	old	Ccl3	-1.06051
StEt 04	3.09104245335832	old	Crim1	-1.6311
StEt 04	3.09104245335832	old	Vsig2	1.24253
StEt 04	3.09104245335832	old	Hgf	0.4431
StEt 04	3.09104245335832	old	Sez6l2	2.15871
StEt 04	3.09104245335832	old	Il1a	0.6116
StEt 04	3.09104245335832	old	Il23r	3.27942
StEt 04	3.09104245335832	old	Dll1	0.62134
StEt 04	3.09104245335832	old	Ddah1	-2.20178
StEt 04	3.09104245335832	old	Il10	-2.84683
StEt 04	3.09104245335832	old	Tnfrsf12a	1.56805
StEt 04	3.09104245335832	old	Acvrl1	-0.03611
StEt 04	3.09104245335832	old	Lgmn	0.97395
StEt 04	3.09104245335832	old	Csf2	-3.79696
StEt 04	3.09104245335832	old	Cxcl9	0.98627
StEt 04	3.09104245335832	old	Map2k6	2.67686
StEt 04	3.09104245335832	old	Itgb1bp2	-6.2872
StEt 04	3.09104245335832	old	Il17f	-5.34008
StEt 04	3.09104245335832	old	Il1b	-4.29378
StEt 04	3.09104245335832	old	Casp3	2.67287
StEt 04	3.09104245335832	old	Apbb1ip	-3.12157
StEt 04	3.09104245335832	old	Wisp1	0.27909
StEt 04	3.09104245335832	old	Cdh6	0.17557
StEt 04	3.09104245335832	old	Pdgfb	5.80848
StEt 04	3.09104245335832	old	Igsf3	0.33948
StEt 04	3.09104245335832	old	Tgfbr3	-0.02983
StEt 04	3.09104245335832	old	Cxcl1	4.22814
StEt 04	3.09104245335832	old	Pak4	-3.78828
StEt 04	3.09104245335832	old	Cntn4	-0.37203
StEt 04	3.09104245335832	old	Ghrl	0.79972
StEt 04	3.09104245335832	old	Lpl	0.59936
StEt 04	3.09104245335832	old	Fstl3	1.37194
StEt 04	3.09104245335832	old	Dctn2	-1.06188
StEt 04	3.09104245335832	old	Il5	-4.40246
StEt 04	3.09104245335832	old	Eda2r	0.84394
StEt 04	3.09104245335832	old	Ntf3	-2.70778
StEt 04	3.09104245335832	old	Tnfsf12	0.20823
StEt 04	3.09104245335832	old	Ccl20	5.07031
StEt 04	3.09104245335832	old	Fli1	-3.78194
StEt 04	3.09104245335832	old	Tpp1	3.10541
StEt 04	3.09104245335832	old	Tnr	0.03941
StEt 04	3.09104245335832	old	Vegfd	-1.1949
StEt 04	3.09104245335832	old	Parp1	0.61051
StEt 04	3.09104245335832	old	Tnf	-4.28027
StEt 05	3.2188758248682	old	Clmp	3.3045
StEt 05	3.2188758248682	old	Matn2	0.77116
StEt 05	3.2188758248682	old	Cpe	2.42883
StEt 05	3.2188758248682	old	Gcg	-1.46561
StEt 05	3.2188758248682	old	Gdnf	-3.86328

StEt 05	3.2188758248682	old	Yes1	-1.56228
StEt 05	3.2188758248682	old	Il17a	-3.06094
StEt 05	3.2188758248682	old	Foxo1	-2.90161
StEt 05	3.2188758248682	old	Tnfrsf11b	0.65218
StEt 05	3.2188758248682	old	Tgfb1	-0.27251
StEt 05	3.2188758248682	old	Pla2g4a	2.41254
StEt 05	3.2188758248682	old	Il6	-4.12924
StEt 05	3.2188758248682	old	Prdx5	-0.96324
StEt 05	3.2188758248682	old	Tgfa	2.14297
StEt 05	3.2188758248682	old	Ccl5	-1.78556
StEt 05	3.2188758248682	old	Epo	-0.40537
StEt 05	3.2188758248682	old	Axin1	-2.61966
StEt 05	3.2188758248682	old	Fst	3.12116
StEt 05	3.2188758248682	old	Rgma	-0.75991
StEt 05	3.2188758248682	old	Nadk	3.39793
StEt 05	3.2188758248682	old	Tnni3	7.86763
StEt 05	3.2188758248682	old	Notch3	1.8953
StEt 05	3.2188758248682	old	Snap29	1.55437
StEt 05	3.2188758248682	old	Cntn1	5.30482
StEt 05	3.2188758248682	old	Clstn2	-2.00014
StEt 05	3.2188758248682	old	S100a4	6.11828
StEt 05	3.2188758248682	old	Ca13	-3.43998
StEt 05	3.2188758248682	old	Mia	1.03481
StEt 05	3.2188758248682	old	Cant1	-1.58247
StEt 05	3.2188758248682	old	Kitlg	-4.6022
StEt 05	3.2188758248682	old	Gfra1	-1.65775
StEt 05	3.2188758248682	old	Ppp1r2	-2.67695
StEt 05	3.2188758248682	old	Adam23	-1.02032
StEt 05	3.2188758248682	old	Itgb6	-2.88912
StEt 05	3.2188758248682	old	Cyr61	0.88375
StEt 05	3.2188758248682	old	Dlk1	1.63434
StEt 05	3.2188758248682	old	Ahr	-2.44108
StEt 05	3.2188758248682	old	Ccl2	6.03572
StEt 05	3.2188758248682	old	Eno2	-0.73855
StEt 05	3.2188758248682	old	Plin1	-2.90675
StEt 05	3.2188758248682	old	Wfikkn2	1.99514
StEt 05	3.2188758248682	old	Flrt2	0.5159
StEt 05	3.2188758248682	old	Qdpr	0.49186
StEt 05	3.2188758248682	old	Fas	1.43293
StEt 05	3.2188758248682	old	Erbb4	0.6029
StEt 05	3.2188758248682	old	Riox2	0.14015
StEt 05	3.2188758248682	old	Plxna4	-1.66065
StEt 05	3.2188758248682	old	Epcam	-1.0913
StEt 05	3.2188758248682	old	Ccl3	-1.29577
StEt 05	3.2188758248682	old	Crim1	-2.02988
StEt 05	3.2188758248682	old	Vsig2	1.74725
StEt 05	3.2188758248682	old	Hgf	-1.08114
StEt 05	3.2188758248682	old	Sez6l2	2.59687
StEt 05	3.2188758248682	old	Il1a	0.30258
StEt 05	3.2188758248682	old	Il23r	4.20982
StEt 05	3.2188758248682	old	Dll1	1.1947
StEt 05	3.2188758248682	old	Ddah1	-1.18427
StEt 05	3.2188758248682	old	Il10	-2.34791
StEt 05	3.2188758248682	old	Tnfrsf12a	1.83752
StEt 05	3.2188758248682	old	Acvrl1	0.04461
StEt 05	3.2188758248682	old	Lgmn	1.48683
StEt 05	3.2188758248682	old	Csf2	-2.67794
StEt 05	3.2188758248682	old	Cxcl9	1.71215
StEt 05	3.2188758248682	old	Map2k6	1.18481
StEt 05	3.2188758248682	old	Itgb1bp2	-5.0158
StEt 05	3.2188758248682	old	Il17f	-4.89924
StEt 05	3.2188758248682	old	Il1b	-3.30016
StEt 05	3.2188758248682	old	Casp3	2.18461
StEt 05	3.2188758248682	old	Apbb1ip	-3.84749
StEt 05	3.2188758248682	old	Wisp1	0.80265
StEt 05	3.2188758248682	old	Cdh6	1.24904
StEt 05	3.2188758248682	old	Pdgfb	0.63228
StEt 05	3.2188758248682	old	Igsf3	-0.29162
StEt 05	3.2188758248682	old	Tgfbr3	0.49581
StEt 05	3.2188758248682	old	Cxcl1	5.12701
StEt 05	3.2188758248682	old	Pak4	-4.32047
StEt 05	3.2188758248682	old	Cntn4	1.00806
StEt 05	3.2188758248682	old	Ghrl	2.24656
StEt 05	3.2188758248682	old	Lpl	1.07018
StEt 05	3.2188758248682	old	Fstl3	1.61288
StEt 05	3.2188758248682	old	Dctn2	-1.86107
StEt 05	3.2188758248682	old	Il5	-0.77023
StEt 05	3.2188758248682	old	Eda2r	0.85302
StEt 05	3.2188758248682	old	Ntf3	-2.4723
StEt 05	3.2188758248682	old	Tnfsf12	0.30747
StEt 05	3.2188758248682	old	Ccl20	6.32196
StEt 05	3.2188758248682	old	Fli1	-4.70574
StEt 05	3.2188758248682	old	Tpp1	3.64844
StEt 05	3.2188758248682	old	Tnr	0.58005
StEt 05	3.2188758248682	old	Vegfd	-0.37017
StEt 05	3.2188758248682	old	Parp1	-2.10279
StEt 05	3.2188758248682	old	Tnf	-3.12595
StEt 06	1.6094379124341	old	Clmp	3.33252
StEt 06	1.6094379124341	old	Matn2	0.70617
StEt 06	1.6094379124341	old	Cpe	2.12769
StEt 06	1.6094379124341	old	Gcg	0.27696
StEt 06	1.6094379124341	old	Gdnf	-3.00186
StEt 06	1.6094379124341	old	Yes1	-2.19521
StEt 06	1.6094379124341	old	Il17a	-1.53925
StEt 06	1.6094379124341	old	Foxo1	-2.78895

StEt 06	1.6094379124341	old	Tnfrsf11b	0.54469
StEt 06	1.6094379124341	old	Tgfb1	-0.2317
StEt 06	1.6094379124341	old	Pla2g4a	2.92877
StEt 06	1.6094379124341	old	Il6	-4.06333
StEt 06	1.6094379124341	old	Prdx5	-1.5944
StEt 06	1.6094379124341	old	Tgfa	2.50107
StEt 06	1.6094379124341	old	Ccl5	-1.67006
StEt 06	1.6094379124341	old	Epo	0.14963
StEt 06	1.6094379124341	old	Axin1	-2.63148
StEt 06	1.6094379124341	old	Fst	4.34222
StEt 06	1.6094379124341	old	Rgma	-0.85811
StEt 06	1.6094379124341	old	Nadk	1.62157
StEt 06	1.6094379124341	old	Tnni3	6.1858
StEt 06	1.6094379124341	old	Notch3	2.07648
StEt 06	1.6094379124341	old	Snap29	1.87918
StEt 06	1.6094379124341	old	Cntn1	5.37453
StEt 06	1.6094379124341	old	Clstn2	-2.25556
StEt 06	1.6094379124341	old	S100a4	5.84556
StEt 06	1.6094379124341	old	Ca13	-3.44944
StEt 06	1.6094379124341	old	Mia	1.55923
StEt 06	1.6094379124341	old	Cant1	-1.41038
StEt 06	1.6094379124341	old	Kitlg	-4.40421
StEt 06	1.6094379124341	old	Gfra1	-1.8169
StEt 06	1.6094379124341	old	Ppp1r2	-2.40791
StEt 06	1.6094379124341	old	Adam23	-0.69146
StEt 06	1.6094379124341	old	Itgb6	-2.54895
StEt 06	1.6094379124341	old	Cyr61	1.37599
StEt 06	1.6094379124341	old	Dlk1	0.40692
StEt 06	1.6094379124341	old	Ahr	-2.88126
StEt 06	1.6094379124341	old	Ccl2	5.41162
StEt 06	1.6094379124341	old	Eno2	-0.71528
StEt 06	1.6094379124341	old	Plin1	-3.19092
StEt 06	1.6094379124341	old	Wfikkn2	2.16041
StEt 06	1.6094379124341	old	Flrt2	0.0442
StEt 06	1.6094379124341	old	Qdpr	-1.56677
StEt 06	1.6094379124341	old	Fas	1.77014
StEt 06	1.6094379124341	old	Erbb4	1.33328
StEt 06	1.6094379124341	old	Riox2	-1.32303
StEt 06	1.6094379124341	old	Plxna4	-1.13619
StEt 06	1.6094379124341	old	Epcam	-1.44987
StEt 06	1.6094379124341	old	Ccl3	-0.86685
StEt 06	1.6094379124341	old	Crim1	-1.54724
StEt 06	1.6094379124341	old	Vsig2	1.46895
StEt 06	1.6094379124341	old	Hgf	-1.11998
StEt 06	1.6094379124341	old	Sez6l2	3.19272
StEt 06	1.6094379124341	old	Il1a	0.86332
StEt 06	1.6094379124341	old	Il23r	4.50649
StEt 06	1.6094379124341	old	Dll1	1.67518
StEt 06	1.6094379124341	old	Ddah1	-3.26063
StEt 06	1.6094379124341	old	Il10	-2.13593
StEt 06	1.6094379124341	old	Tnfrsf12a	1.8059
StEt 06	1.6094379124341	old	Acvrl1	0.66909
StEt 06	1.6094379124341	old	Lgmn	1.66492
StEt 06	1.6094379124341	old	Csf2	-3.02627
StEt 06	1.6094379124341	old	Cxcl9	1.5852
StEt 06	1.6094379124341	old	Map2k6	1.80137
StEt 06	1.6094379124341	old	Itgb1bp2	-5.10411
StEt 06	1.6094379124341	old	Il17f	-4.05484
StEt 06	1.6094379124341	old	Il1b	-3.45542
StEt 06	1.6094379124341	old	Casp3	1.98927
StEt 06	1.6094379124341	old	Apbb1ip	-3.51612
StEt 06	1.6094379124341	old	Wisp1	0.97185
StEt 06	1.6094379124341	old	Cdh6	0.68115
StEt 06	1.6094379124341	old	Pdgfb	0.79927
StEt 06	1.6094379124341	old	Igsf3	-0.04698
StEt 06	1.6094379124341	old	Tgfbr3	0.79886
StEt 06	1.6094379124341	old	Cxcl1	4.72088
StEt 06	1.6094379124341	old	Pak4	-3.6365
StEt 06	1.6094379124341	old	Cntn4	0.36007
StEt 06	1.6094379124341	old	Ghrl	2.56829
StEt 06	1.6094379124341	old	Lpl	0.88448
StEt 06	1.6094379124341	old	Fstl3	2.13834
StEt 06	1.6094379124341	old	Dctn2	-2.04055
StEt 06	1.6094379124341	old	Il5	-2.91506
StEt 06	1.6094379124341	old	Eda2r	1.09678
StEt 06	1.6094379124341	old	Ntf3	-1.70498
StEt 06	1.6094379124341	old	Tnfsf12	0.21974
StEt 06	1.6094379124341	old	Ccl20	5.54355
StEt 06	1.6094379124341	old	Fli1	-4.17933
StEt 06	1.6094379124341	old	Tpp1	3.63739
StEt 06	1.6094379124341	old	Tnr	0.69646
StEt 06	1.6094379124341	old	Vegfd	-0.42181
StEt 06	1.6094379124341	old	Parp1	-2.30285
StEt 06	1.6094379124341	old	Tnf	-3.13597
StEt 07	2.89037175789616	old	Clmp	2.88243
StEt 07	2.89037175789616	old	Matn2	0.28957
StEt 07	2.89037175789616	old	Cpe	1.80444
StEt 07	2.89037175789616	old	Gcg	1.17464
StEt 07	2.89037175789616	old	Gdnf	-4.21351
StEt 07	2.89037175789616	old	Yes1	-1.51142
StEt 07	2.89037175789616	old	Il17a	-2.17857
StEt 07	2.89037175789616	old	Foxo1	-2.26049
StEt 07	2.89037175789616	old	Tnfrsf11b	0.31973
StEt 07	2.89037175789616	old	Tgfb1	-0.39104
StEt 07	2.89037175789616	old	Pla2g4a	3.01169

StEt 07 2.89037175789616 old Il6 -3.73865
StEt 07 2.89037175789616 old Prdx5 -0.56892
StEt 07 2.89037175789616 old Tgfa 1.81848
StEt 07 2.89037175789616 old Ccl5 -1.69475
StEt 07 2.89037175789616 old Epo -0.93545
StEt 07 2.89037175789616 old Axin1 -2.20586
StEt 07 2.89037175789616 old Fst 3.57765
StEt 07 2.89037175789616 old Rgma -0.88372
StEt 07 2.89037175789616 old Nadk 3.88448
StEt 07 2.89037175789616 old Tnni3 8.54472
StEt 07 2.89037175789616 old Notch3 1.73221
StEt 07 2.89037175789616 old Snap29 2.38603
StEt 07 2.89037175789616 old Cntn1 4.9714
StEt 07 2.89037175789616 old Clstn2 -2.98196
StEt 07 2.89037175789616 old S100a4 5.30008
StEt 07 2.89037175789616 old Ca13 -2.93708
StEt 07 2.89037175789616 old Mia 0.94086
StEt 07 2.89037175789616 old Cant1 -1.69831
StEt 07 2.89037175789616 old Kitlg -4.44805
StEt 07 2.89037175789616 old Gfra1 -2.39116
StEt 07 2.89037175789616 old Ppp1r2 -1.79482
StEt 07 2.89037175789616 old Adam23 -1.04579
StEt 07 2.89037175789616 old Itgb6 -2.28549
StEt 07 2.89037175789616 old Cyr61 0.88995
StEt 07 2.89037175789616 old Dlk1 0.15946
StEt 07 2.89037175789616 old Ahr -1.91795
StEt 07 2.89037175789616 old Ccl2 5.57374
StEt 07 2.89037175789616 old Eno2 -1.2456
StEt 07 2.89037175789616 old Plin1 -2.27228
StEt 07 2.89037175789616 old Wfikkn2 1.67567
StEt 07 2.89037175789616 old Flrt2 -0.09643
StEt 07 2.89037175789616 old Qdpr 0.8464
StEt 07 2.89037175789616 old Fas 1.46826
StEt 07 2.89037175789616 old Erbb4 0.99294
StEt 07 2.89037175789616 old Riox2 0.88357
StEt 07 2.89037175789616 old Plxna4 -0.56331
StEt 07 2.89037175789616 old Epcam -1.22403
StEt 07 2.89037175789616 old Ccl3 -0.87706
StEt 07 2.89037175789616 old Crim1 -1.78354
StEt 07 2.89037175789616 old Vsig2 1.11033
StEt 07 2.89037175789616 old Hgf -1.24413
StEt 07 2.89037175789616 old Sez6l2 2.49606
StEt 07 2.89037175789616 old Il1a 0.68325
StEt 07 2.89037175789616 old Il23r 3.84836
StEt 07 2.89037175789616 old Dll1 1.4875
StEt 07 2.89037175789616 old Ddah1 -0.35115
StEt 07 2.89037175789616 old Il10 -1.92027
StEt 07 2.89037175789616 old Tnfrsf12a 1.84895
StEt 07 2.89037175789616 old Acvrl1 0.14647
StEt 07 2.89037175789616 old Lgmn 1.51744
StEt 07 2.89037175789616 old Csf2 -3.19903
StEt 07 2.89037175789616 old Cxcl9 1.31399
StEt 07 2.89037175789616 old Map2k6 1.78046
StEt 07 2.89037175789616 old Itgb1bp2 -5.15162
StEt 07 2.89037175789616 old Il17f -4.43867
StEt 07 2.89037175789616 old Il1b -3.35597
StEt 07 2.89037175789616 old Casp3 2.21422
StEt 07 2.89037175789616 old Apbb1ip -3.74618
StEt 07 2.89037175789616 old Wisp1 0.54372
StEt 07 2.89037175789616 old Cdh6 0.75188
StEt 07 2.89037175789616 old Pdgfb 1.17264
StEt 07 2.89037175789616 old Igsf3 -0.52442
StEt 07 2.89037175789616 old Tgfbr3 0.46703
StEt 07 2.89037175789616 old Cxcl1 4.79788
StEt 07 2.89037175789616 old Pak4 -2.79777
StEt 07 2.89037175789616 old Cntn4 -0.24068
StEt 07 2.89037175789616 old Ghrl 2.52539
StEt 07 2.89037175789616 old Lpl -0.03184
StEt 07 2.89037175789616 old Fstl3 1.60821
StEt 07 2.89037175789616 old Dctn2 -1.97253
StEt 07 2.89037175789616 old Il5 -2.72408
StEt 07 2.89037175789616 old Eda2r 0.75414
StEt 07 2.89037175789616 old Ntf3 -1.83157
StEt 07 2.89037175789616 old Tnfsf12 -0.22855
StEt 07 2.89037175789616 old Ccl20 4.79349
StEt 07 2.89037175789616 old Fli1 -4.01718
StEt 07 2.89037175789616 old Tpp1 3.18592
StEt 07 2.89037175789616 old Tnr 0.52833
StEt 07 2.89037175789616 old Vegfd -0.78799
StEt 07 2.89037175789616 old Parp1 -1.87276
StEt 07 2.89037175789616 old Tnf -4.12245
StEt 08 2.77258872223978 young Clmp 3.13069
StEt 08 2.77258872223978 young Matn2 0.06004
StEt 08 2.77258872223978 young Cpe 2.3244
StEt 08 2.77258872223978 young Gcg -0.55291
StEt 08 2.77258872223978 young Gdnf -4.01906
StEt 08 2.77258872223978 young Yes1 -1.77857
StEt 08 2.77258872223978 young Il17a -1.50495
StEt 08 2.77258872223978 young Foxo1 -2.54627
StEt 08 2.77258872223978 young Tnfrsf11b 0.5485
StEt 08 2.77258872223978 young Tgfb1 -0.60888
StEt 08 2.77258872223978 young Pla2g4a 2.78785
StEt 08 2.77258872223978 young Il6 -4.56869
StEt 08 2.77258872223978 young Prdx5 -1.0683
StEt 08 2.77258872223978 young Tgfa 1.83942

StEt 08	2.77258872223978	young	Ccl5	-1.05519
StEt 08	2.77258872223978	young	Epo	-0.55119
StEt 08	2.77258872223978	young	Axin1	-2.70136
StEt 08	2.77258872223978	young	Fst	3.97794
StEt 08	2.77258872223978	young	Rgma	-0.96722
StEt 08	2.77258872223978	young	Nadk	2.40875
StEt 08	2.77258872223978	young	Tnni3	6.96268
StEt 08	2.77258872223978	young	Notch3	1.63323
StEt 08	2.77258872223978	young	Snap29	1.45679
StEt 08	2.77258872223978	young	Cntn1	5.14533
StEt 08	2.77258872223978	young	Clstn2	-2.67392
StEt 08	2.77258872223978	young	S100a4	5.98188
StEt 08	2.77258872223978	young	Ca13	-3.06412
StEt 08	2.77258872223978	young	Mia	1.70931
StEt 08	2.77258872223978	young	Cant1	-1.25394
StEt 08	2.77258872223978	young	Kitlg	-5.41876
StEt 08	2.77258872223978	young	Gfra1	-2.01322
StEt 08	2.77258872223978	young	Ppp1r2	-3.15179
StEt 08	2.77258872223978	young	Adam23	-1.49641
StEt 08	2.77258872223978	young	Itgb6	-2.31871
StEt 08	2.77258872223978	young	Cyr61	0.35546
StEt 08	2.77258872223978	young	Dlk1	1.15483
StEt 08	2.77258872223978	young	Ahr	-2.95857
StEt 08	2.77258872223978	young	Ccl2	6.27167
StEt 08	2.77258872223978	young	Eno2	-0.7564
StEt 08	2.77258872223978	young	Plin1	-0.29575
StEt 08	2.77258872223978	young	Wfikkn2	2.25377
StEt 08	2.77258872223978	young	Flrt2	-0.31317
StEt 08	2.77258872223978	young	Qdpr	-0.69082
StEt 08	2.77258872223978	young	Fas	1.33731
StEt 08	2.77258872223978	young	Erbb4	1.10676
StEt 08	2.77258872223978	young	Riox2	-0.75331
StEt 08	2.77258872223978	young	Plxna4	-0.2122
StEt 08	2.77258872223978	young	Epcam	-1.03075
StEt 08	2.77258872223978	young	Ccl3	-0.49755
StEt 08	2.77258872223978	young	Crim1	-1.77837
StEt 08	2.77258872223978	young	Vsig2	1.76457
StEt 08	2.77258872223978	young	Hgf	-0.91394
StEt 08	2.77258872223978	young	Sez6l2	2.11123
StEt 08	2.77258872223978	young	Il1a	0.96909
StEt 08	2.77258872223978	young	Il23r	4.65518
StEt 08	2.77258872223978	young	Dll1	1.29856
StEt 08	2.77258872223978	young	Ddah1	-1.99629
StEt 08	2.77258872223978	young	Il10	-2.21387
StEt 08	2.77258872223978	young	Tnfrsf12a	2.08241
StEt 08	2.77258872223978	young	Acvrl1	0.29274
StEt 08	2.77258872223978	young	Lgmn	1.90791
StEt 08	2.77258872223978	young	Csf2	-3.22895
StEt 08	2.77258872223978	young	Cxcl9	1.21805
StEt 08	2.77258872223978	young	Map2k6	1.67409
StEt 08	2.77258872223978	young	Itgb1bp2	-3.78783
StEt 08	2.77258872223978	young	Il17f	-4.56744
StEt 08	2.77258872223978	young	Il1b	-3.65087
StEt 08	2.77258872223978	young	Casp3	1.61987
StEt 08	2.77258872223978	young	Apbb1ip	-3.87207
StEt 08	2.77258872223978	young	Wisp1	1.15474
StEt 08	2.77258872223978	young	Cdh6	1.04983
StEt 08	2.77258872223978	young	Pdgfb	1.86251
StEt 08	2.77258872223978	young	Igsf3	-0.72942
StEt 08	2.77258872223978	young	Tgfbr3	0.65116
StEt 08	2.77258872223978	young	Cxcl1	4.48177
StEt 08	2.77258872223978	young	Pak4	-4.25362
StEt 08	2.77258872223978	young	Cntn4	1.11734
StEt 08	2.77258872223978	young	Ghrl	3.43283
StEt 08	2.77258872223978	young	Lpl	0.61673
StEt 08	2.77258872223978	young	Fstl3	1.86097
StEt 08	2.77258872223978	young	Dctn2	-2.10716
StEt 08	2.77258872223978	young	Il5	-2.17131
StEt 08	2.77258872223978	young	Eda2r	0.90561
StEt 08	2.77258872223978	young	Ntf3	-1.9242
StEt 08	2.77258872223978	young	Tnfsf12	0.22816
StEt 08	2.77258872223978	young	Ccl20	5.86854
StEt 08	2.77258872223978	young	Fli1	-4.50732
StEt 08	2.77258872223978	young	Tpp1	3.34997
StEt 08	2.77258872223978	young	Tnr	-0.16651
StEt 08	2.77258872223978	young	Vegfd	-0.4
StEt 08	2.77258872223978	young	Parp1	-2.10551
StEt 08	2.77258872223978	young	Tnf	-3.42356
StEt 09	4.39444915467244	young	Clmp	2.43883
StEt 09	4.39444915467244	young	Matn2	-0.487
StEt 09	4.39444915467244	young	Cpe	1.77448
StEt 09	4.39444915467244	young	Gcg	-1.89502
StEt 09	4.39444915467244	young	Gdnf	-4.72918
StEt 09	4.39444915467244	young	Yes1	0.5085
StEt 09	4.39444915467244	young	Il17a	-0.9332
StEt 09	4.39444915467244	young	Foxo1	-1.14832
StEt 09	4.39444915467244	young	Tnfrsf11b	-0.06051
StEt 09	4.39444915467244	young	Tgfb1	-0.592
StEt 09	4.39444915467244	young	Pla2g4a	3.20186
StEt 09	4.39444915467244	young	Il6	-3.66829
StEt 09	4.39444915467244	young	Prdx5	0.13906
StEt 09	4.39444915467244	young	Tgfa	1.6914
StEt 09	4.39444915467244	young	Ccl5	-1.44652
StEt 09	4.39444915467244	young	Epo	-1.20626
StEt 09	4.39444915467244	young	Axin1	-1.25028

StEt 09 4.39444915467244 young Fst 3.37671
StEt 09 4.39444915467244 young Rgma -1.06517
StEt 09 4.39444915467244 young Nadk 5.32679
StEt 09 4.39444915467244 young Tnni3 9.66264
StEt 09 4.39444915467244 young Notch3 1.9144
StEt 09 4.39444915467244 young Snap29 3.72886
StEt 09 4.39444915467244 young Cntn1 4.56178
StEt 09 4.39444915467244 young Clstn2 -3.91091
StEt 09 4.39444915467244 young S100a4 5.98433
StEt 09 4.39444915467244 young Ca13 -1.69205
StEt 09 4.39444915467244 young Mia 0.87015
StEt 09 4.39444915467244 young Cant1 -2.10222
StEt 09 4.39444915467244 young Kitlg -5.73446
StEt 09 4.39444915467244 young Gfra1 -3.30165
StEt 09 4.39444915467244 young Ppp1r2 -1.42139
StEt 09 4.39444915467244 young Adam23 -2.50186
StEt 09 4.39444915467244 young Itgb6 -3.31137
StEt 09 4.39444915467244 young Cyr61 -0.22579
StEt 09 4.39444915467244 young Dlk1 0.40216
StEt 09 4.39444915467244 young Ahr -0.45503
StEt 09 4.39444915467244 young Ccl2 6.57284
StEt 09 4.39444915467244 young Eno2 -1.38367
StEt 09 4.39444915467244 young Plin1 0.9319
StEt 09 4.39444915467244 young Wfikkn2 1.67182
StEt 09 4.39444915467244 young Flrt2 -0.14344
StEt 09 4.39444915467244 young Qdpr 1.92102
StEt 09 4.39444915467244 young Fas 1.1316
StEt 09 4.39444915467244 young Erbb4 0.31142
StEt 09 4.39444915467244 young Riox2 2.06138
StEt 09 4.39444915467244 young Plxna4 -1.13831
StEt 09 4.39444915467244 young Epcam -2.07841
StEt 09 4.39444915467244 young Ccl3 -0.77586
StEt 09 4.39444915467244 young Crim1 -2.03114
StEt 09 4.39444915467244 young Vsig2 0.97551
StEt 09 4.39444915467244 young Hgf -0.95104
StEt 09 4.39444915467244 young Sez6l2 1.68345
StEt 09 4.39444915467244 young Il1a 1.19452
StEt 09 4.39444915467244 young Il23r 3.8705
StEt 09 4.39444915467244 young Dll1 0.46625
StEt 09 4.39444915467244 young Ddah1 1.27854
StEt 09 4.39444915467244 young Il10 -2.64709
StEt 09 4.39444915467244 young Tnfrsf12a 0.9619
StEt 09 4.39444915467244 young Acvrl1 -0.23399
StEt 09 4.39444915467244 young Lgmn 1.57201
StEt 09 4.39444915467244 young Csf2 -3.23904
StEt 09 4.39444915467244 young Cxcl9 0.38794
StEt 09 4.39444915467244 young Map2k6 3.16054
StEt 09 4.39444915467244 young Itgb1bp2 -6.60631
StEt 09 4.39444915467244 young Il17f -4.74381
StEt 09 4.39444915467244 young Il1b -3.03513
StEt 09 4.39444915467244 young Casp3 3.14571
StEt 09 4.39444915467244 young Apbb1ip -3.39278
StEt 09 4.39444915467244 young Wisp1 0.34629
StEt 09 4.39444915467244 young Cdh6 0.39881
StEt 09 4.39444915467244 young Pdgfb 3.14126
StEt 09 4.39444915467244 young Igsf3 -1.02822
StEt 09 4.39444915467244 young Tgfbr3 0.05155
StEt 09 4.39444915467244 young Cxcl1 4.41689
StEt 09 4.39444915467244 young Pak4 -3.37655
StEt 09 4.39444915467244 young Cntn4 0.72708
StEt 09 4.39444915467244 young Ghrl 1.9423
StEt 09 4.39444915467244 young Lpl -0.33052
StEt 09 4.39444915467244 young Fstl3 1.10526
StEt 09 4.39444915467244 young Dctn2 -1.57517
StEt 09 4.39444915467244 young Il5 -2.02524
StEt 09 4.39444915467244 young Eda2r 0.09026
StEt 09 4.39444915467244 young Ntf3 -2.92551
StEt 09 4.39444915467244 young Tnfsf12 -0.30971
StEt 09 4.39444915467244 young Ccl20 3.90072
StEt 09 4.39444915467244 young Fli1 -4.36768
StEt 09 4.39444915467244 young Tpp1 2.80957
StEt 09 4.39444915467244 young Tnr -0.52792
StEt 09 4.39444915467244 young Vegfd -1.24083
StEt 09 4.39444915467244 young Parp1 -0.79426
StEt 09 4.39444915467244 young Tnf -3.77085
StEt 10 2.07944154167984 old Clmp 3.04362
StEt 10 2.07944154167984 old Matn2 0.48851
StEt 10 2.07944154167984 old Cpe 2.15745
StEt 10 2.07944154167984 old Gcg 1.31749
StEt 10 2.07944154167984 old Gdnf -3.82005
StEt 10 2.07944154167984 old Yes1 -1.92813
StEt 10 2.07944154167984 old Il17a -2.86199
StEt 10 2.07944154167984 old Foxo1 -2.04615
StEt 10 2.07944154167984 old Tnfrsf11b 0.58996
StEt 10 2.07944154167984 old Tgfb1 -0.50103
StEt 10 2.07944154167984 old Pla2g4a 3.20865
StEt 10 2.07944154167984 old Il6 -4.47039
StEt 10 2.07944154167984 old Prdx5 -1.43147
StEt 10 2.07944154167984 old Tgfa 2.22871
StEt 10 2.07944154167984 old Ccl5 -1.61465
StEt 10 2.07944154167984 old Epo -0.81652
StEt 10 2.07944154167984 old Axin1 -2.14714
StEt 10 2.07944154167984 old Fst 3.95173
StEt 10 2.07944154167984 old Rgma -0.98259
StEt 10 2.07944154167984 old Nadk 2.54333

StEt 10	2.07944154167984	old	Tnni3	5.48902
StEt 10	2.07944154167984	old	Notch3	2.08603
StEt 10	2.07944154167984	old	Snap29	2.20215
StEt 10	2.07944154167984	old	Cntn1	5.41875
StEt 10	2.07944154167984	old	Clstn2	-2.62386
StEt 10	2.07944154167984	old	S100a4	5.76552
StEt 10	2.07944154167984	old	Ca13	-2.59454
StEt 10	2.07944154167984	old	Mia	1.15608
StEt 10	2.07944154167984	old	Cant1	-1.42933
StEt 10	2.07944154167984	old	Kitlg	-4.75416
StEt 10	2.07944154167984	old	Gfra1	-1.93532
StEt 10	2.07944154167984	old	Ppp1r2	-2.27899
StEt 10	2.07944154167984	old	Adam23	-0.86143
StEt 10	2.07944154167984	old	Itgb6	-2.6507
StEt 10	2.07944154167984	old	Cyr61	1.20898
StEt 10	2.07944154167984	old	Dlk1	0.46275
StEt 10	2.07944154167984	old	Ahr	-2.36357
StEt 10	2.07944154167984	old	Ccl2	5.05355
StEt 10	2.07944154167984	old	Eno2	-0.81203
StEt 10	2.07944154167984	old	Plin1	-1.95761
StEt 10	2.07944154167984	old	Wfikkn2	1.87505
StEt 10	2.07944154167984	old	Flrt2	0.17474
StEt 10	2.07944154167984	old	Qdpr	-0.57952
StEt 10	2.07944154167984	old	Fas	1.75912
StEt 10	2.07944154167984	old	Erbb4	1.32424
StEt 10	2.07944154167984	old	Riox2	-0.06285
StEt 10	2.07944154167984	old	Plxna4	0.28776
StEt 10	2.07944154167984	old	Epcam	-1.32831
StEt 10	2.07944154167984	old	Ccl3	-0.90762
StEt 10	2.07944154167984	old	Crim1	-1.85194
StEt 10	2.07944154167984	old	Vsig2	1.51855
StEt 10	2.07944154167984	old	Hgf	-1.15263
StEt 10	2.07944154167984	old	Sez6l2	3.10126
StEt 10	2.07944154167984	old	Il1a	0.75143
StEt 10	2.07944154167984	old	Il23r	4.15406
StEt 10	2.07944154167984	old	Dll1	1.56978
StEt 10	2.07944154167984	old	Ddah1	-2.7377
StEt 10	2.07944154167984	old	Il10	-2.35514
StEt 10	2.07944154167984	old	Tnfrsf12a	2.06745
StEt 10	2.07944154167984	old	Acvrl1	0.25085
StEt 10	2.07944154167984	old	Lgmn	1.23358
StEt 10	2.07944154167984	old	Csf2	-2.97548
StEt 10	2.07944154167984	old	Cxcl9	1.21627
StEt 10	2.07944154167984	old	Map2k6	1.84359
StEt 10	2.07944154167984	old	Itgb1bp2	-7.08385
StEt 10	2.07944154167984	old	Il17f	-4.63101
StEt 10	2.07944154167984	old	Il1b	-3.4707
StEt 10	2.07944154167984	old	Casp3	2.35727
StEt 10	2.07944154167984	old	Apbb1ip	-3.43104
StEt 10	2.07944154167984	old	Wisp1	0.85891
StEt 10	2.07944154167984	old	Cdh6	1.32323
StEt 10	2.07944154167984	old	Pdgfb	1.31571
StEt 10	2.07944154167984	old	Igsf3	-0.18661
StEt 10	2.07944154167984	old	Tgfbr3	0.85717
StEt 10	2.07944154167984	old	Cxcl1	5.05874
StEt 10	2.07944154167984	old	Pak4	-2.84291
StEt 10	2.07944154167984	old	Cntn4	0.29
StEt 10	2.07944154167984	old	Ghrl	2.46588
StEt 10	2.07944154167984	old	Lpl	0.7118
StEt 10	2.07944154167984	old	Fstl3	1.91596
StEt 10	2.07944154167984	old	Dctn2	-2.29571
StEt 10	2.07944154167984	old	Il5	-3.38201
StEt 10	2.07944154167984	old	Eda2r	1.03641
StEt 10	2.07944154167984	old	Ntf3	-1.79519
StEt 10	2.07944154167984	old	Tnfsf12	0.07601
StEt 10	2.07944154167984	old	Ccl20	5.20826
StEt 10	2.07944154167984	old	Fli1	-3.46902
StEt 10	2.07944154167984	old	Tpp1	3.37404
StEt 10	2.07944154167984	old	Tnr	0.71903
StEt 10	2.07944154167984	old	Vegfd	-0.49498
StEt 10	2.07944154167984	old	Parp1	-2.27002
StEt 10	2.07944154167984	old	Tnf	-2.88286
StEt 11	3.46573590279973	young	Clmp	1.9663
StEt 11	3.46573590279973	young	Matn2	-0.33552
StEt 11	3.46573590279973	young	Cpe	1.20808
StEt 11	3.46573590279973	young	Gcg	-2.71216
StEt 11	3.46573590279973	young	Gdnf	-4.53374
StEt 11	3.46573590279973	young	Yes1	1.90643
StEt 11	3.46573590279973	young	Il17a	-2.71524
StEt 11	3.46573590279973	young	Foxo1	-0.455
StEt 11	3.46573590279973	young	Tnfrsf11b	-0.63632
StEt 11	3.46573590279973	young	Tgfb1	-0.82621
StEt 11	3.46573590279973	young	Pla2g4a	3.17619
StEt 11	3.46573590279973	young	Il6	-4.94184
StEt 11	3.46573590279973	young	Prdx5	2.5111
StEt 11	3.46573590279973	young	Tgfa	2.16989
StEt 11	3.46573590279973	young	Ccl5	-1.24046
StEt 11	3.46573590279973	young	Epo	-1.56284
StEt 11	3.46573590279973	young	Axin1	-0.35035
StEt 11	3.46573590279973	young	Fst	2.92397
StEt 11	3.46573590279973	young	Rgma	-1.41573
StEt 11	3.46573590279973	young	Nadk	5.17834
StEt 11	3.46573590279973	young	Tnni3	10.26491
StEt 11	3.46573590279973	young	Notch3	1.20112
StEt 11	3.46573590279973	young	Snap29	4.11401

StEt 11	3.46573590279973	young	Cntn1	4.13952
StEt 11	3.46573590279973	young	Clstn2	-3.44919
StEt 11	3.46573590279973	young	S100a4	4.86009
StEt 11	3.46573590279973	young	Ca13	-2.25067
StEt 11	3.46573590279973	young	Mia	0.13899
StEt 11	3.46573590279973	young	Cant1	-2.21042
StEt 11	3.46573590279973	young	Kitlg	-5.58389
StEt 11	3.46573590279973	young	Gfra1	-3.60097
StEt 11	3.46573590279973	young	Ppp1r2	-1.07096
StEt 11	3.46573590279973	young	Adam23	-2.0296
StEt 11	3.46573590279973	young	Itgb6	-3.49595
StEt 11	3.46573590279973	young	Cyr61	-0.29498
StEt 11	3.46573590279973	young	Dlk1	-0.40931
StEt 11	3.46573590279973	young	Ahr	0.20414
StEt 11	3.46573590279973	young	Ccl2	5.27727
StEt 11	3.46573590279973	young	Eno2	-1.43863
StEt 11	3.46573590279973	young	Plin1	1.04221
StEt 11	3.46573590279973	young	Wfikkn2	0.95811
StEt 11	3.46573590279973	young	Flrt2	0.70991
StEt 11	3.46573590279973	young	Qdpr	2.07284
StEt 11	3.46573590279973	young	Fas	1.80744
StEt 11	3.46573590279973	young	Erbb4	0.60518
StEt 11	3.46573590279973	young	Riox2	2.36454
StEt 11	3.46573590279973	young	Plxna4	0.1885
StEt 11	3.46573590279973	young	Epcam	-2.34069
StEt 11	3.46573590279973	young	Ccl3	-1.32466
StEt 11	3.46573590279973	young	Crim1	-1.90625
StEt 11	3.46573590279973	young	Vsig2	0.55527
StEt 11	3.46573590279973	young	Hgf	-0.99284
StEt 11	3.46573590279973	young	Sez6l2	1.76978
StEt 11	3.46573590279973	young	Il1a	1.38757
StEt 11	3.46573590279973	young	Il23r	3.53357
StEt 11	3.46573590279973	young	Dll1	0.45824
StEt 11	3.46573590279973	young	Ddah1	3.04804
StEt 11	3.46573590279973	young	Il10	-2.77094
StEt 11	3.46573590279973	young	Tnfrsf12a	0.6213
StEt 11	3.46573590279973	young	Acvrl1	-0.44377
StEt 11	3.46573590279973	young	Lgmn	1.53211
StEt 11	3.46573590279973	young	Csf2	-4.27197
StEt 11	3.46573590279973	young	Cxcl9	0.54947
StEt 11	3.46573590279973	young	Map2k6	2.99533
StEt 11	3.46573590279973	young	Itgb1bp2	-4.65003
StEt 11	3.46573590279973	young	Il17f	-5.70008
StEt 11	3.46573590279973	young	Il1b	-3.50847
StEt 11	3.46573590279973	young	Casp3	3.96329
StEt 11	3.46573590279973	young	Apbb1ip	-3.19629
StEt 11	3.46573590279973	young	Wisp1	-0.22337
StEt 11	3.46573590279973	young	Cdh6	-0.64498
StEt 11	3.46573590279973	young	Pdgfb	3.21662
StEt 11	3.46573590279973	young	Igsf3	-0.89048
StEt 11	3.46573590279973	young	Tgfbr3	-0.11652
StEt 11	3.46573590279973	young	Cxcl1	3.61749
StEt 11	3.46573590279973	young	Pak4	-3.01062
StEt 11	3.46573590279973	young	Cntn4	-0.58005
StEt 11	3.46573590279973	young	Ghrl	1.2533
StEt 11	3.46573590279973	young	Lpl	-0.38905
StEt 11	3.46573590279973	young	Fstl3	0.70131
StEt 11	3.46573590279973	young	Dctn2	0.68061
StEt 11	3.46573590279973	young	Il5	-4.40606
StEt 11	3.46573590279973	young	Eda2r	-0.33828
StEt 11	3.46573590279973	young	Ntf3	-2.77446
StEt 11	3.46573590279973	young	Tnfsf12	-0.11123
StEt 11	3.46573590279973	young	Ccl20	4.43514
StEt 11	3.46573590279973	young	Fli1	-4.25603
StEt 11	3.46573590279973	young	Tpp1	2.59851
StEt 11	3.46573590279973	young	Tnr	-0.60112
StEt 11	3.46573590279973	young	Vegfd	-1.67915
StEt 11	3.46573590279973	young	Parp1	0.05497
StEt 11	3.46573590279973	young	Tnf	-3.9235
StEt 12	2.30258509299405	young	Clmp	2.92156
StEt 12	2.30258509299405	young	Matn2	0.18178
StEt 12	2.30258509299405	young	Cpe	1.94489
StEt 12	2.30258509299405	young	Gcg	-1.82807
StEt 12	2.30258509299405	young	Gdnf	-4.39794
StEt 12	2.30258509299405	young	Yes1	-1.97324
StEt 12	2.30258509299405	young	Il17a	-1.50401
StEt 12	2.30258509299405	young	Foxo1	-2.9104
StEt 12	2.30258509299405	young	Tnfrsf11b	0.14894
StEt 12	2.30258509299405	young	Tgfb1	-0.25864
StEt 12	2.30258509299405	young	Pla2g4a	4.02371
StEt 12	2.30258509299405	young	Il6	-4.25705
StEt 12	2.30258509299405	young	Prdx5	-0.13004
StEt 12	2.30258509299405	young	Tgfa	1.74972
StEt 12	2.30258509299405	young	Ccl5	-1.23996
StEt 12	2.30258509299405	young	Epo	-1.36546
StEt 12	2.30258509299405	young	Axin1	-1.73077
StEt 12	2.30258509299405	young	Fst	2.81584
StEt 12	2.30258509299405	young	Rgma	-1.08031
StEt 12	2.30258509299405	young	Nadk	3.31624
StEt 12	2.30258509299405	young	Tnni3	8.74332
StEt 12	2.30258509299405	young	Notch3	1.89444
StEt 12	2.30258509299405	young	Snap29	2.96687
StEt 12	2.30258509299405	young	Cntn1	5.02079
StEt 12	2.30258509299405	young	Clstn2	-2.9155
StEt 12	2.30258509299405	young	S100a4	5.42784

StEt 12	2.30258509299405	young	Ca13	-3.43508
StEt 12	2.30258509299405	young	Mia	1.03935
StEt 12	2.30258509299405	young	Cant1	-1.65397
StEt 12	2.30258509299405	young	Kitlg	-5.09275
StEt 12	2.30258509299405	young	Gfra1	-2.4038
StEt 12	2.30258509299405	young	Ppp1r2	-3.02929
StEt 12	2.30258509299405	young	Adam23	-1.70201
StEt 12	2.30258509299405	young	Itgb6	-3.11958
StEt 12	2.30258509299405	young	Cyr61	-0.04757
StEt 12	2.30258509299405	young	Dlk1	2.35273
StEt 12	2.30258509299405	young	Ahr	-2.77346
StEt 12	2.30258509299405	young	Ccl2	6.01697
StEt 12	2.30258509299405	young	Eno2	-1.49515
StEt 12	2.30258509299405	young	Plin1	1.20945
StEt 12	2.30258509299405	young	Wfikkn2	1.66649
StEt 12	2.30258509299405	young	Flrt2	-0.56132
StEt 12	2.30258509299405	young	Qdpr	0.27147
StEt 12	2.30258509299405	young	Fas	1.34749
StEt 12	2.30258509299405	young	Erbb4	0.637
StEt 12	2.30258509299405	young	Riox2	0.51481
StEt 12	2.30258509299405	young	Plxna4	1.24823
StEt 12	2.30258509299405	young	Epcam	-1.30828
StEt 12	2.30258509299405	young	Ccl3	-1.12447
StEt 12	2.30258509299405	young	Crim1	-2.0014
StEt 12	2.30258509299405	young	Vsig2	1.05071
StEt 12	2.30258509299405	young	Hgf	-0.87779
StEt 12	2.30258509299405	young	Sez6l2	2.33139
StEt 12	2.30258509299405	young	Il1a	2.60888
StEt 12	2.30258509299405	young	Il23r	4.45834
StEt 12	2.30258509299405	young	Dll1	0.98433
StEt 12	2.30258509299405	young	Ddah1	-1.29933
StEt 12	2.30258509299405	young	Il10	-2.70917
StEt 12	2.30258509299405	young	Tnfrsf12a	1.5614
StEt 12	2.30258509299405	young	Acvrl1	0.10657
StEt 12	2.30258509299405	young	Lgmn	1.49729
StEt 12	2.30258509299405	young	Csf2	-3.15951
StEt 12	2.30258509299405	young	Cxcl9	1.86278
StEt 12	2.30258509299405	young	Map2k6	3.16425
StEt 12	2.30258509299405	young	Itgb1bp2	-9.05602
StEt 12	2.30258509299405	young	Il17f	-4.9514
StEt 12	2.30258509299405	young	Il1b	-3.64183
StEt 12	2.30258509299405	young	Casp3	3.57504
StEt 12	2.30258509299405	young	Apbb1ip	-3.44288
StEt 12	2.30258509299405	young	Wisp1	0.83435
StEt 12	2.30258509299405	young	Cdh6	0.89463
StEt 12	2.30258509299405	young	Pdgfb	3.83449
StEt 12	2.30258509299405	young	Igsf3	-0.54517
StEt 12	2.30258509299405	young	Tgfbr3	0.34498
StEt 12	2.30258509299405	young	Cxcl1	4.45504
StEt 12	2.30258509299405	young	Pak4	-4.4473
StEt 12	2.30258509299405	young	Cntn4	1.07313
StEt 12	2.30258509299405	young	Ghrl	2.59759
StEt 12	2.30258509299405	young	Lpl	-0.05252
StEt 12	2.30258509299405	young	Fstl3	1.37709
StEt 12	2.30258509299405	young	Dctn2	-0.59507
StEt 12	2.30258509299405	young	Il5	-2.2708
StEt 12	2.30258509299405	young	Eda2r	0.67338
StEt 12	2.30258509299405	young	Ntf3	-2.33675
StEt 12	2.30258509299405	young	Tnfsf12	0.12571
StEt 12	2.30258509299405	young	Ccl20	4.74193
StEt 12	2.30258509299405	young	Fli1	-3.65208
StEt 12	2.30258509299405	young	Tpp1	3.04667
StEt 12	2.30258509299405	young	Tnr	-0.17656
StEt 12	2.30258509299405	young	Vegfd	-0.83407
StEt 12	2.30258509299405	young	Parp1	-1.53717
StEt 12	2.30258509299405	young	Tnf	-3.73536
StEt 14	3.17805383034795	young	Clmp	2.56955
StEt 14	3.17805383034795	young	Matn2	0.3496
StEt 14	3.17805383034795	young	Cpe	1.68929
StEt 14	3.17805383034795	young	Gcg	-1.51044
StEt 14	3.17805383034795	young	Gdnf	-4.37599
StEt 14	3.17805383034795	young	Yes1	0.68192
StEt 14	3.17805383034795	young	Il17a	-1.68404
StEt 14	3.17805383034795	young	Foxo1	-1.33417
StEt 14	3.17805383034795	young	Tnfrsf11b	-0.20033
StEt 14	3.17805383034795	young	Tgfb1	-0.41962
StEt 14	3.17805383034795	young	Pla2g4a	3.38542
StEt 14	3.17805383034795	young	Il6	-4.74154
StEt 14	3.17805383034795	young	Prdx5	1.03777
StEt 14	3.17805383034795	young	Tgfa	2.20313
StEt 14	3.17805383034795	young	Ccl5	-1.76873
StEt 14	3.17805383034795	young	Epo	-1.85933
StEt 14	3.17805383034795	young	Axin1	-0.89036
StEt 14	3.17805383034795	young	Fst	2.42261
StEt 14	3.17805383034795	young	Rgma	-1.39978
StEt 14	3.17805383034795	young	Nadk	4.67885
StEt 14	3.17805383034795	young	Tnni3	7.92697
StEt 14	3.17805383034795	young	Notch3	1.1479
StEt 14	3.17805383034795	young	Snap29	3.17641
StEt 14	3.17805383034795	young	Cntn1	4.42962
StEt 14	3.17805383034795	young	Clstn2	-2.96739
StEt 14	3.17805383034795	young	S100a4	5.94702
StEt 14	3.17805383034795	young	Ca13	-1.32649
StEt 14	3.17805383034795	young	Mia	0.34071
StEt 14	3.17805383034795	young	Cant1	-2.45246

StEt 14	3.17805383034795	young	Kitlg	-5.68124
StEt 14	3.17805383034795	young	Gfra1	-2.62554
StEt 14	3.17805383034795	young	Ppp1r2	-2.09858
StEt 14	3.17805383034795	young	Adam23	-2.38299
StEt 14	3.17805383034795	young	Itgb6	-3.82315
StEt 14	3.17805383034795	young	Cyr61	-0.43705
StEt 14	3.17805383034795	young	Dlk1	0.0093
StEt 14	3.17805383034795	young	Ahr	-0.8317
StEt 14	3.17805383034795	young	Ccl2	5.54698
StEt 14	3.17805383034795	young	Eno2	-0.84846
StEt 14	3.17805383034795	young	Plin1	2.21802
StEt 14	3.17805383034795	young	Wfikkn2	1.00902
StEt 14	3.17805383034795	young	Flrt2	0.38206
StEt 14	3.17805383034795	young	Qdpr	1.58915
StEt 14	3.17805383034795	young	Fas	1.59698
StEt 14	3.17805383034795	young	Erbb4	-0.26963
StEt 14	3.17805383034795	young	Riox2	2.49534
StEt 14	3.17805383034795	young	Plxna4	-0.04755
StEt 14	3.17805383034795	young	Epcam	-1.97951
StEt 14	3.17805383034795	young	Ccl3	-1.24063
StEt 14	3.17805383034795	young	Crim1	-2.01428
StEt 14	3.17805383034795	young	Vsig2	0.75941
StEt 14	3.17805383034795	young	Hgf	-0.71878
StEt 14	3.17805383034795	young	Sez6l2	2.11796
StEt 14	3.17805383034795	young	Il1a	1.89418
StEt 14	3.17805383034795	young	Il23r	3.45282
StEt 14	3.17805383034795	young	Dll1	0.46906
StEt 14	3.17805383034795	young	Ddah1	1.70515
StEt 14	3.17805383034795	young	Il10	-2.22236
StEt 14	3.17805383034795	young	Tnfrsf12a	1.42921
StEt 14	3.17805383034795	young	Acvrl1	-0.20762
StEt 14	3.17805383034795	young	Lgmn	1.30676
StEt 14	3.17805383034795	young	Csf2	-3.97576
StEt 14	3.17805383034795	young	Cxcl9	1.28702
StEt 14	3.17805383034795	young	Map2k6	2.53882
StEt 14	3.17805383034795	young	Itgb1bp2	-6.38184
StEt 14	3.17805383034795	young	Il17f	-5.58118
StEt 14	3.17805383034795	young	Il1b	-3.34651
StEt 14	3.17805383034795	young	Casp3	3.89435
StEt 14	3.17805383034795	young	Apbb1ip	-2.65806
StEt 14	3.17805383034795	young	Wisp1	0.3327
StEt 14	3.17805383034795	young	Cdh6	0.07938
StEt 14	3.17805383034795	young	Pdgfb	4.56074
StEt 14	3.17805383034795	young	Igsf3	-0.34357
StEt 14	3.17805383034795	young	Tgfbr3	-0.30116
StEt 14	3.17805383034795	young	Cxcl1	4.01641
StEt 14	3.17805383034795	young	Pak4	-3.48849
StEt 14	3.17805383034795	young	Cntn4	0.26782
StEt 14	3.17805383034795	young	Ghrl	1.64927
StEt 14	3.17805383034795	young	Lpl	-0.32286
StEt 14	3.17805383034795	young	Fstl3	1.17337
StEt 14	3.17805383034795	young	Dctn2	-0.47047
StEt 14	3.17805383034795	young	Il5	-3.76287
StEt 14	3.17805383034795	young	Eda2r	0.26135
StEt 14	3.17805383034795	young	Ntf3	-2.57788
StEt 14	3.17805383034795	young	Tnfsf12	-0.14316
StEt 14	3.17805383034795	young	Ccl20	3.49891
StEt 14	3.17805383034795	young	Fli1	-2.7496
StEt 14	3.17805383034795	young	Tpp1	2.52128
StEt 14	3.17805383034795	young	Tnr	-0.79879
StEt 14	3.17805383034795	young	Vegfd	-1.41759
StEt 14	3.17805383034795	young	Parp1	0.89643
StEt 14	3.17805383034795	young	Tnf	-4.26604
StEt 15	1.94591014905531	young	Clmp	2.92171
StEt 15	1.94591014905531	young	Matn2	0.22438
StEt 15	1.94591014905531	young	Cpe	1.76516
StEt 15	1.94591014905531	young	Gcg	-1.28425
StEt 15	1.94591014905531	young	Gdnf	-4.4252
StEt 15	1.94591014905531	young	Yes1	-1.33357
StEt 15	1.94591014905531	young	Il17a	-1.96613
StEt 15	1.94591014905531	young	Foxo1	-2.10099
StEt 15	1.94591014905531	young	Tnfrsf11b	0.04315
StEt 15	1.94591014905531	young	Tgfb1	-0.24224
StEt 15	1.94591014905531	young	Pla2g4a	3.5635
StEt 15	1.94591014905531	young	Il6	-4.69575
StEt 15	1.94591014905531	young	Prdx5	0.39996
StEt 15	1.94591014905531	young	Tgfa	1.69566
StEt 15	1.94591014905531	young	Ccl5	-0.38224
StEt 15	1.94591014905531	young	Epo	-1.19892
StEt 15	1.94591014905531	young	Axin1	-1.95848
StEt 15	1.94591014905531	young	Fst	3.32807
StEt 15	1.94591014905531	young	Rgma	-1.35042
StEt 15	1.94591014905531	young	Nadk	4.07486
StEt 15	1.94591014905531	young	Tnni3	6.88776
StEt 15	1.94591014905531	young	Notch3	2.01394
StEt 15	1.94591014905531	young	Snap29	3.16353
StEt 15	1.94591014905531	young	Cntn1	4.87606
StEt 15	1.94591014905531	young	Clstn2	-3.08925
StEt 15	1.94591014905531	young	S100a4	5.13015
StEt 15	1.94591014905531	young	Ca13	-3.06263
StEt 15	1.94591014905531	young	Mia	0.95433
StEt 15	1.94591014905531	young	Cant1	-1.48364
StEt 15	1.94591014905531	young	Kitlg	-5.49492
StEt 15	1.94591014905531	young	Gfra1	-2.4809
StEt 15	1.94591014905531	young	Ppp1r2	-2.75412

StEt 15	1.94591014905531	young	Adam23	-0.96755
StEt 15	1.94591014905531	young	Itgb6	-2.75992
StEt 15	1.94591014905531	young	Cyr61	0.36804
StEt 15	1.94591014905531	young	Dlk1	0.36633
StEt 15	1.94591014905531	young	Ahr	-1.97066
StEt 15	1.94591014905531	young	Ccl2	5.74602
StEt 15	1.94591014905531	young	Eno2	-1.16405
StEt 15	1.94591014905531	young	Plin1	-1.88675
StEt 15	1.94591014905531	young	Wfikkn2	1.60456
StEt 15	1.94591014905531	young	Flrt2	0.10235
StEt 15	1.94591014905531	young	Qdpr	1.24258
StEt 15	1.94591014905531	young	Fas	1.41124
StEt 15	1.94591014905531	young	Erbb4	1.21867
StEt 15	1.94591014905531	young	Riox2	0.77466
StEt 15	1.94591014905531	young	Plxna4	0.08502
StEt 15	1.94591014905531	young	Epcam	-1.97328
StEt 15	1.94591014905531	young	Ccl3	-0.6241
StEt 15	1.94591014905531	young	Crim1	-1.79488
StEt 15	1.94591014905531	young	Vsig2	1.6057
StEt 15	1.94591014905531	young	Hgf	-0.98485
StEt 15	1.94591014905531	young	Sez6l2	2.63717
StEt 15	1.94591014905531	young	Il1a	1.89062
StEt 15	1.94591014905531	young	Il23r	4.4353
StEt 15	1.94591014905531	young	Dll1	1.28865
StEt 15	1.94591014905531	young	Ddah1	0.59293
StEt 15	1.94591014905531	young	Il10	-2.48976
StEt 15	1.94591014905531	young	Tnfrsf12a	1.58738
StEt 15	1.94591014905531	young	Acvrl1	0.13446
StEt 15	1.94591014905531	young	Lgmn	1.59397
StEt 15	1.94591014905531	young	Csf2	-3.72578
StEt 15	1.94591014905531	young	Cxcl9	1.22769
StEt 15	1.94591014905531	young	Map2k6	2.94015
StEt 15	1.94591014905531	young	Itgb1bp2	-6.00718
StEt 15	1.94591014905531	young	Il17f	-4.99899
StEt 15	1.94591014905531	young	Il1b	-3.71913
StEt 15	1.94591014905531	young	Casp3	3.44048
StEt 15	1.94591014905531	young	Apbb1ip	-3.66176
StEt 15	1.94591014905531	young	Wisp1	0.67045
StEt 15	1.94591014905531	young	Cdh6	0.6775
StEt 15	1.94591014905531	young	Pdgfb	3.20568
StEt 15	1.94591014905531	young	Igsf3	-0.67411
StEt 15	1.94591014905531	young	Tgfbr3	0.56113
StEt 15	1.94591014905531	young	Cxcl1	4.39103
StEt 15	1.94591014905531	young	Pak4	-3.49392
StEt 15	1.94591014905531	young	Cntn4	0.23279
StEt 15	1.94591014905531	young	Ghrl	2.43649
StEt 15	1.94591014905531	young	Lpl	-0.13293
StEt 15	1.94591014905531	young	Fstl3	1.5297
StEt 15	1.94591014905531	young	Dctn2	-0.85098
StEt 15	1.94591014905531	young	Il5	-3.56883
StEt 15	1.94591014905531	young	Eda2r	0.53301
StEt 15	1.94591014905531	young	Ntf3	-1.96347
StEt 15	1.94591014905531	young	Tnfsf12	-0.11235
StEt 15	1.94591014905531	young	Ccl20	4.66651
StEt 15	1.94591014905531	young	Fli1	-3.97135
StEt 15	1.94591014905531	young	Tpp1	3.516
StEt 15	1.94591014905531	young	Tnr	0.22242
StEt 15	1.94591014905531	young	Vegfd	-0.90966
StEt 15	1.94591014905531	young	Parp1	-2.07485
StEt 15	1.94591014905531	young	Tnf	-4.19379
StEt 16	3.76120011569356	old	Clmp	2.82676
StEt 16	3.76120011569356	old	Matn2	0.62573
StEt 16	3.76120011569356	old	Cpe	1.43515
StEt 16	3.76120011569356	old	Gcg	-1.31057
StEt 16	3.76120011569356	old	Gdnf	-4.31583
StEt 16	3.76120011569356	old	Yes1	-0.64189
StEt 16	3.76120011569356	old	Il17a	-1.33951
StEt 16	3.76120011569356	old	Foxo1	-1.69306
StEt 16	3.76120011569356	old	Tnfrsf11b	0.40356
StEt 16	3.76120011569356	old	Tgfb1	-0.84279
StEt 16	3.76120011569356	old	Pla2g4a	3.48254
StEt 16	3.76120011569356	old	Il6	-2.40517
StEt 16	3.76120011569356	old	Prdx5	0.23643
StEt 16	3.76120011569356	old	Tgfa	1.93992
StEt 16	3.76120011569356	old	Ccl5	-1.87645
StEt 16	3.76120011569356	old	Epo	0.88123
StEt 16	3.76120011569356	old	Axin1	-1.36937
StEt 16	3.76120011569356	old	Fst	1.79893
StEt 16	3.76120011569356	old	Rgma	-0.9519
StEt 16	3.76120011569356	old	Nadk	4.85924
StEt 16	3.76120011569356	old	Tnni3	9.07413
StEt 16	3.76120011569356	old	Notch3	1.47333
StEt 16	3.76120011569356	old	Snap29	2.96747
StEt 16	3.76120011569356	old	Cntn1	4.85464
StEt 16	3.76120011569356	old	Clstn2	-2.81804
StEt 16	3.76120011569356	old	S100a4	5.63482
StEt 16	3.76120011569356	old	Ca13	-3.50909
StEt 16	3.76120011569356	old	Mia	0.26627
StEt 16	3.76120011569356	old	Cant1	-1.92775
StEt 16	3.76120011569356	old	Kitlg	-5.35967
StEt 16	3.76120011569356	old	Gfra1	-1.95109
StEt 16	3.76120011569356	old	Ppp1r2	-1.31749
StEt 16	3.76120011569356	old	Adam23	-1.32982
StEt 16	3.76120011569356	old	Itgb6	-3.25049
StEt 16	3.76120011569356	old	Cyr61	0.44511

StEt 16 3.76120011569356 old Dlk1 -0.04255
StEt 16 3.76120011569356 old Ahr -1.28792
StEt 16 3.76120011569356 old Ccl2 5.93907
StEt 16 3.76120011569356 old Eno2 -1.09411
StEt 16 3.76120011569356 old Plin1 -1.42443
StEt 16 3.76120011569356 old Wfikkn2 1.14403
StEt 16 3.76120011569356 old Flrt2 -0.71025
StEt 16 3.76120011569356 old Qdpr 1.56839
StEt 16 3.76120011569356 old Fas 1.7234
StEt 16 3.76120011569356 old Erbb4 -0.12386
StEt 16 3.76120011569356 old Riox2 2.00207
StEt 16 3.76120011569356 old Plxna4 -1.4075
StEt 16 3.76120011569356 old Epcam -1.78256
StEt 16 3.76120011569356 old Ccl3 -1.46792
StEt 16 3.76120011569356 old Crim1 -2.01568
StEt 16 3.76120011569356 old Vsig2 0.5255
StEt 16 3.76120011569356 old Hgf -1.01868
StEt 16 3.76120011569356 old Sez6l2 2.48626
StEt 16 3.76120011569356 old Il1a 1.5802
StEt 16 3.76120011569356 old Il23r 3.88767
StEt 16 3.76120011569356 old Dll1 1.29747
StEt 16 3.76120011569356 old Ddah1 1.06453
StEt 16 3.76120011569356 old Il10 -1.75399
StEt 16 3.76120011569356 old Tnfrsf12a 1.87133
StEt 16 3.76120011569356 old Acvrl1 -0.11515
StEt 16 3.76120011569356 old Lgmn 1.56272
StEt 16 3.76120011569356 old Csf2 -3.74151
StEt 16 3.76120011569356 old Cxcl9 1.48281
StEt 16 3.76120011569356 old Map2k6 2.45369
StEt 16 3.76120011569356 old Itgb1bp2 -6.29867
StEt 16 3.76120011569356 old Il17f -5.0789
StEt 16 3.76120011569356 old Il1b -2.35248
StEt 16 3.76120011569356 old Casp3 3.14099
StEt 16 3.76120011569356 old Apbb1ip -3.64697
StEt 16 3.76120011569356 old Wisp1 0.46391
StEt 16 3.76120011569356 old Cdh6 0.56513
StEt 16 3.76120011569356 old Pdgfb 1.42823
StEt 16 3.76120011569356 old Igsf3 -0.51349
StEt 16 3.76120011569356 old Tgfbr3 0.19333
StEt 16 3.76120011569356 old Cxcl1 4.70569
StEt 16 3.76120011569356 old Pak4 -3.43788
StEt 16 3.76120011569356 old Cntn4 -0.12788
StEt 16 3.76120011569356 old Ghrl 2.08605
StEt 16 3.76120011569356 old Lpl -0.42014
StEt 16 3.76120011569356 old Fstl3 1.60957
StEt 16 3.76120011569356 old Dctn2 -1.0302
StEt 16 3.76120011569356 old Il5 -2.76451
StEt 16 3.76120011569356 old Eda2r 0.19498
StEt 16 3.76120011569356 old Ntf3 -2.76229
StEt 16 3.76120011569356 old Tnfsf12 -0.37738
StEt 16 3.76120011569356 old Ccl20 4.1692
StEt 16 3.76120011569356 old Fli1 -4.18736
StEt 16 3.76120011569356 old Tpp1 3.13
StEt 16 3.76120011569356 old Tnr -0.03498
StEt 16 3.76120011569356 old Vegfd -1.02405
StEt 16 3.76120011569356 old Parp1 -1.63143
StEt 16 3.76120011569356 old Tnf -3.59716
StEt 17 0 young Clmp 2.67601
StEt 17 0 young Matn2 -0.30139
StEt 17 0 young Cpe 1.57555
StEt 17 0 young Gcg -0.82814
StEt 17 0 young Gdnf -4.23716
StEt 17 0 young Yes1 -0.29514
StEt 17 0 young Il17a -3.23551
StEt 17 0 young Foxo1 -1.2004
StEt 17 0 young Tnfrsf11b -0.01264
StEt 17 0 young Tgfb1 -0.92535
StEt 17 0 young Pla2g4a 2.84689
StEt 17 0 young Il6 -4.391
StEt 17 0 young Prdx5 0.54677
StEt 17 0 young Tgfa 1.6075
StEt 17 0 young Ccl5 -1.73845
StEt 17 0 young Epo -1.32634
StEt 17 0 young Axin1 -1.15838
StEt 17 0 young Fst 2.97629
StEt 17 0 young Rgma -0.80848
StEt 17 0 young Nadk 4.924
StEt 17 0 young Tnni3 9.55432
StEt 17 0 young Notch3 1.81221
StEt 17 0 young Snap29 3.4811
StEt 17 0 young Cntn1 4.84748
StEt 17 0 young Clstn2 -2.99139
StEt 17 0 young S100a4 5.37486
StEt 17 0 young Ca13 -3.81724
StEt 17 0 young Mia 0.76075
StEt 17 0 young Cant1 -1.83355
StEt 17 0 young Kitlg -5.02471
StEt 17 0 young Gfra1 -2.45777
StEt 17 0 young Ppp1r2 -1.88073
StEt 17 0 young Adam23 -1.11617
StEt 17 0 young Itgb6 -3.23384
StEt 17 0 young Cyr61 0.68959
StEt 17 0 young Dlk1 0.02007
StEt 17 0 young Ahr -1.17671
StEt 17 0 young Ccl2 5.1753

StEt	17	0	young	Eno2	-1.07994
StEt	17	0	young	Plin1	-0.86511
StEt	17	0	young	Wfikkn2	1.29358
StEt	17	0	young	Flrt2	-0.31224
StEt	17	0	young	Qdpr	1.97477
StEt	17	0	young	Fas	1.63466
StEt	17	0	young	Erbb4	0.91314
StEt	17	0	young	Riox2	1.91457
StEt	17	0	young	Plxna4	0.39264
StEt	17	0	young	Epcam	-1.47198
StEt	17	0	young	Ccl3	-1.08747
StEt	17	0	young	Crim1	-1.75324
StEt	17	0	young	Vsig2	1.30443
StEt	17	0	young	Hgf	-1.25183
StEt	17	0	young	Sez6l2	2.5657
StEt	17	0	young	Il1a	1.38093
StEt	17	0	young	Il23r	3.78734
StEt	17	0	young	Dll1	1.10531
StEt	17	0	young	Ddah1	1.79929
StEt	17	0	young	Il10	-2.56996
StEt	17	0	young	Tnfrsf12a	1.55918
StEt	17	0	young	Acvrl1	-0.05924
StEt	17	0	young	Lgmn	1.34954
StEt	17	0	young	Csf2	-3.46222
StEt	17	0	young	Cxcl9	0.90532
StEt	17	0	young	Map2k6	2.7436
StEt	17	0	young	Itgb1bp2	-6.62644
StEt	17	0	young	Il17f	-4.87324
StEt	17	0	young	Il1b	-3.68247
StEt	17	0	young	Casp3	3.43028
StEt	17	0	young	Apbb1ip	-3.44473
StEt	17	0	young	Wisp1	0.08146
StEt	17	0	young	Cdh6	-0.02025
StEt	17	0	young	Pdgfb	1.88864
StEt	17	0	young	Igsf3	-0.69553
StEt	17	0	young	Tgfbr3	0.29329
StEt	17	0	young	Cxcl1	4.98373
StEt	17	0	young	Pak4	-3.28974
StEt	17	0	young	Cntn4	0.03358
StEt	17	0	young	Ghrl	2.1185
StEt	17	0	young	Lpl	-0.10326
StEt	17	0	young	Fstl3	1.38326
StEt	17	0	young	Dctn2	-0.84627
StEt	17	0	young	Il5	-4.06547
StEt	17	0	young	Eda2r	0.36723
StEt	17	0	young	Ntf3	-2.3471
StEt	17	0	young	Tnfsf12	-0.22023
StEt	17	0	young	Ccl20	4.35187
StEt	17	0	young	Fli1	-3.14768
StEt	17	0	young	Tpp1	3.08026
StEt	17	0	young	Tnr	0.0678
StEt	17	0	young	Vegfd	-1.02023
StEt	17	0	young	Parp1	-1.74144
StEt	17	0	young	Tnf	-3.54456
StEt	18	3.71357206670431	young	Clmp	2.39444
StEt	18	3.71357206670431	young	Matn2	0.06651
StEt	18	3.71357206670431	young	Cpe	1.67969
StEt	18	3.71357206670431	young	Gcg	-1.14526
StEt	18	3.71357206670431	young	Gdnf	-4.55243
StEt	18	3.71357206670431	young	Yes1	0.64369
StEt	18	3.71357206670431	young	Il17a	-1.67799
StEt	18	3.71357206670431	young	Foxo1	-1.30451
StEt	18	3.71357206670431	young	Tnfrsf11b	-0.07676
StEt	18	3.71357206670431	young	Tgfb1	-0.75471
StEt	18	3.71357206670431	young	Pla2g4a	2.6647
StEt	18	3.71357206670431	young	Il6	-4.44505
StEt	18	3.71357206670431	young	Prdx5	0.77327
StEt	18	3.71357206670431	young	Tgfa	1.87761
StEt	18	3.71357206670431	young	Ccl5	-1.74286
StEt	18	3.71357206670431	young	Epo	-1.57996
StEt	18	3.71357206670431	young	Axin1	-1.08283
StEt	18	3.71357206670431	young	Fst	2.29019
StEt	18	3.71357206670431	young	Rgma	-0.6749
StEt	18	3.71357206670431	young	Nadk	5.45681
StEt	18	3.71357206670431	young	Tnni3	10.70942
StEt	18	3.71357206670431	young	Notch3	1.53103
StEt	18	3.71357206670431	young	Snap29	3.79708
StEt	18	3.71357206670431	young	Cntn1	4.6567
StEt	18	3.71357206670431	young	Clstn2	-3.30652
StEt	18	3.71357206670431	young	S100a4	5.23817
StEt	18	3.71357206670431	young	Ca13	-3.62412
StEt	18	3.71357206670431	young	Mia	0.62634
StEt	18	3.71357206670431	young	Cant1	-2.33264
StEt	18	3.71357206670431	young	Kitlg	-5.86649
StEt	18	3.71357206670431	young	Gfra1	-2.71566
StEt	18	3.71357206670431	young	Ppp1r2	-1.01124
StEt	18	3.71357206670431	young	Adam23	-1.77088
StEt	18	3.71357206670431	young	Itgb6	-3.6147
StEt	18	3.71357206670431	young	Cyr61	-0.26764
StEt	18	3.71357206670431	young	Dlk1	0.08545
StEt	18	3.71357206670431	young	Ahr	-0.34453
StEt	18	3.71357206670431	young	Ccl2	5.48848
StEt	18	3.71357206670431	young	Eno2	-1.07848
StEt	18	3.71357206670431	young	Plin1	-0.23163
StEt	18	3.71357206670431	young	Wfikkn2	0.84506

StEt	18	3.71357206670431	young	Flrt2	-0.81739
StEt	18	3.71357206670431	young	Qdpr	2.06663
StEt	18	3.71357206670431	young	Fas	1.81713
StEt	18	3.71357206670431	young	Erbb4	0.13726
StEt	18	3.71357206670431	young	Riox2	2.76034
StEt	18	3.71357206670431	young	Plxna4	-0.49652
StEt	18	3.71357206670431	young	Epcam	-1.95178
StEt	18	3.71357206670431	young	Ccl3	-1.37393
StEt	18	3.71357206670431	young	Crim1	-1.95477
StEt	18	3.71357206670431	young	Vsig2	0.98065
StEt	18	3.71357206670431	young	Hgf	-1.0559
StEt	18	3.71357206670431	young	Sez6l2	2.08424
StEt	18	3.71357206670431	young	Il1a	1.48315
StEt	18	3.71357206670431	young	Il23r	4.0391
StEt	18	3.71357206670431	young	Dll1	0.66742
StEt	18	3.71357206670431	young	Ddah1	2.05349
StEt	18	3.71357206670431	young	Il10	-2.94875
StEt	18	3.71357206670431	young	Tnfrsf12a	1.76239
StEt	18	3.71357206670431	young	Acvrl1	-0.20155
StEt	18	3.71357206670431	young	Lgmn	1.73389
StEt	18	3.71357206670431	young	Csf2	-3.57593
StEt	18	3.71357206670431	young	Cxcl9	1.12695
StEt	18	3.71357206670431	young	Map2k6	2.72744
StEt	18	3.71357206670431	young	Itgb1bp2	-5.4558
StEt	18	3.71357206670431	young	Il17f	-5.29642
StEt	18	3.71357206670431	young	Il1b	-3.47064
StEt	18	3.71357206670431	young	Casp3	3.36387
StEt	18	3.71357206670431	young	Apbb1ip	-3.75403
StEt	18	3.71357206670431	young	Wisp1	0.30854
StEt	18	3.71357206670431	young	Cdh6	0.36483
StEt	18	3.71357206670431	young	Pdgfb	2.1279
StEt	18	3.71357206670431	young	Igsf3	-0.90763
StEt	18	3.71357206670431	young	Tgfbr3	0.07754
StEt	18	3.71357206670431	young	Cxcl1	4.26128
StEt	18	3.71357206670431	young	Pak4	-3.7303
StEt	18	3.71357206670431	young	Cntn4	0.3533
StEt	18	3.71357206670431	young	Ghrl	2.28813
StEt	18	3.71357206670431	young	Lpl	-0.2883
StEt	18	3.71357206670431	young	Fstl3	0.93641
StEt	18	3.71357206670431	young	Dctn2	0.03818
StEt	18	3.71357206670431	young	Il5	-2.85121
StEt	18	3.71357206670431	young	Eda2r	0.1832
StEt	18	3.71357206670431	young	Ntf3	-2.49406
StEt	18	3.71357206670431	young	Tnfsf12	-0.18813
StEt	18	3.71357206670431	young	Ccl20	3.47752
StEt	18	3.71357206670431	young	Fli1	-4.09782
StEt	18	3.71357206670431	young	Tpp1	3.16856
StEt	18	3.71357206670431	young	Tnr	-0.54124
StEt	18	3.71357206670431	young	Vegfd	-1.11778
StEt	18	3.71357206670431	young	Parp1	0.17035
StEt	18	3.71357206670431	young	Tnf	-3.61256
StEt	20	1.94591014905531	young	Clmp	2.78969
StEt	20	1.94591014905531	young	Matn2	0.09881
StEt	20	1.94591014905531	young	Cpe	1.96049
StEt	20	1.94591014905531	young	Gcg	0.23129
StEt	20	1.94591014905531	young	Gdnf	-4.07882
StEt	20	1.94591014905531	young	Yes1	-1.3906
StEt	20	1.94591014905531	young	Il17a	-0.38706
StEt	20	1.94591014905531	young	Foxo1	-2.58384
StEt	20	1.94591014905531	young	Tnfrsf11b	0.06074
StEt	20	1.94591014905531	young	Tgfb1	-0.17747
StEt	20	1.94591014905531	young	Pla2g4a	3.88184
StEt	20	1.94591014905531	young	Il6	-4.65681
StEt	20	1.94591014905531	young	Prdx5	-0.38414
StEt	20	1.94591014905531	young	Tgfa	1.5871
StEt	20	1.94591014905531	young	Ccl5	-1.6084
StEt	20	1.94591014905531	young	Epo	-0.38291
StEt	20	1.94591014905531	young	Axin1	-1.61959
StEt	20	1.94591014905531	young	Fst	2.74758
StEt	20	1.94591014905531	young	Rgma	-1.15977
StEt	20	1.94591014905531	young	Nadk	4.06776
StEt	20	1.94591014905531	young	Tnni3	2.99306
StEt	20	1.94591014905531	young	Notch3	1.80533
StEt	20	1.94591014905531	young	Snap29	3.60267
StEt	20	1.94591014905531	young	Cntn1	5.01681
StEt	20	1.94591014905531	young	Clstn2	-2.87586
StEt	20	1.94591014905531	young	S100a4	5.38327
StEt	20	1.94591014905531	young	Ca13	-3.37088
StEt	20	1.94591014905531	young	Mia	0.66098
StEt	20	1.94591014905531	young	Cant1	-1.70559
StEt	20	1.94591014905531	young	Kitlg	-5.52263
StEt	20	1.94591014905531	young	Gfra1	-2.68936
StEt	20	1.94591014905531	young	Ppp1r2	-1.94478
StEt	20	1.94591014905531	young	Adam23	-1.9786
StEt	20	1.94591014905531	young	Itgb6	-3.19841
StEt	20	1.94591014905531	young	Cyr61	-0.17495
StEt	20	1.94591014905531	young	Dlk1	0.46379
StEt	20	1.94591014905531	young	Ahr	-2.4221
StEt	20	1.94591014905531	young	Ccl2	6.39438
StEt	20	1.94591014905531	young	Eno2	-1.11376
StEt	20	1.94591014905531	young	Plin1	-0.59959
StEt	20	1.94591014905531	young	Wfikkn2	1.83174
StEt	20	1.94591014905531	young	Flrt2	-0.35062
StEt	20	1.94591014905531	young	Qdpr	0.9492
StEt	20	1.94591014905531	young	Fas	1.28511

```
StEt 20  1.94591014905531  young  Erbb4  0.65431
StEt 20  1.94591014905531  young  Riox2  0.64305
StEt 20  1.94591014905531  young  Plxna4  1.81061
StEt 20  1.94591014905531  young  Epcam  -1.59221
StEt 20  1.94591014905531  young  Ccl3  -0.79394
StEt 20  1.94591014905531  young  Crim1  -2.24987
StEt 20  1.94591014905531  young  Vsig2  0.81426
StEt 20  1.94591014905531  young  Hgf  -1.01924
StEt 20  1.94591014905531  young  Sez6l2  2.18858
StEt 20  1.94591014905531  young  Il1a  2.53307
StEt 20  1.94591014905531  young  Il23r  4.41664
StEt 20  1.94591014905531  young  Dll1  1.09114
StEt 20  1.94591014905531  young  Ddah1  0.56448
StEt 20  1.94591014905531  young  Il10  -2.46202
StEt 20  1.94591014905531  young  Tnfrsf12a  1.46609
StEt 20  1.94591014905531  young  Acvrl1  -0.0148
StEt 20  1.94591014905531  young  Lgmn  1.70319
StEt 20  1.94591014905531  young  Csf2  -3.43217
StEt 20  1.94591014905531  young  Cxcl9  0.9618
StEt 20  1.94591014905531  young  Map2k6  3.00954
StEt 20  1.94591014905531  young  Itgb1bp2  -4.77398
StEt 20  1.94591014905531  young  Il17f  -5.0945
StEt 20  1.94591014905531  young  Il1b  -3.80929
StEt 20  1.94591014905531  young  Casp3  3.39295
StEt 20  1.94591014905531  young  Apbb1ip  -3.5575
StEt 20  1.94591014905531  young  Wisp1  0.62278
StEt 20  1.94591014905531  young  Cdh6  1.02528
StEt 20  1.94591014905531  young  Pdgfb  3.01943
StEt 20  1.94591014905531  young  Igsf3  -0.54035
StEt 20  1.94591014905531  young  Tgfbr3  0.36041
StEt 20  1.94591014905531  young  Cxcl1  4.39815
StEt 20  1.94591014905531  young  Pak4  -3.9549
StEt 20  1.94591014905531  young  Cntn4  1.02256
StEt 20  1.94591014905531  young  Ghrl  2.57272
StEt 20  1.94591014905531  young  Lpl  0.2592
StEt 20  1.94591014905531  young  Fstl3  1.37222
StEt 20  1.94591014905531  young  Dctn2  -0.45718
StEt 20  1.94591014905531  young  Il5  -2.39602
StEt 20  1.94591014905531  young  Eda2r  0.29734
StEt 20  1.94591014905531  young  Ntf3  -2.04424
StEt 20  1.94591014905531  young  Tnfsf12  -0.11364
StEt 20  1.94591014905531  young  Ccl20  3.71265
StEt 20  1.94591014905531  young  Fli1  -3.50509
StEt 20  1.94591014905531  young  Tpp1  3.18439
StEt 20  1.94591014905531  young  Tnr  -0.3688
StEt 20  1.94591014905531  young  Vegfd  -0.95293
StEt 20  1.94591014905531  young  Parp1  -1.37141
StEt 20  1.94591014905531  young  Tnf  -4.02772
;
RUN;

/*This analysis follows the same concepts as that for Plasma GSH Level and
protein expression above, including troubleshooting REML-related errors.
However, in this case, the interaction with age group is not significant, although it is retained
to ensure the correct calculation of error variance and DF. Accordingly, our Benjamini
Hochberg tests are based on the average regression slope between stereotypy and
each protein. These are figured by the Estimates below. Again, SAS infers a complete
matrix from these simplified matrices, which can be double-checked using
the "/E" option in the Estimate statement*/

PROC MIXED DATA=Olink_And_ST_20241125 ALPHA=0.05;
CLASS SubjectID TwoGroups Protein;
MODEL  Norm_NPX =  TwoGroups Protein TwoGroups*Protein
Log_SumAllST_1 Log_SumAllST_1*Protein Log_SumAllST_1*TwoGroups
Log_SumAllST_1*Protein*TwoGroups/ ddfm=contain;
RANDOM SubjectID(TwoGroups ) ;

Estimate "Acvrl1 Slope" Log_SumAllST_1 1 Log_SumAllST_1*Protein 1 0 0 0 0 0 0 0 0 0 0 0 0 0 0 0 0 0 0 0 0 0 0 0 0 0 0 0 0 0 0 0 0
0 0 0 0 0 0 0 0 0 0 0 0 0 0 0 0 0 0 0 0 0 0 0 0 0 0 0 0 0 0 0 0 0 0 0 0 0 0 0 0 0 0 0 0 0 0 0 0 0 0 0 0 0 0 0 0 0 0 0 ;
Estimate "Adam23 Slope" Log_SumAllST_1 1 Log_SumAllST_1*Protein 0 1 0 0 0 0 0 0 0 0 0 0 0 0 0 0 0 0 0 0 0 0 0 0 0 0 0 0 0 0 0 0 0
0 0 0 0 0 0 0 0 0 0 0 0 0 0 0 0 0 0 0 0 0 0 0 0 0 0 0 0 0 0 0 0 0 0 0 0 0 0 0 0 0 0 0 0 0 0 0 0 0 0 0 0 0 0 0 0 0 0 0  ;
Estimate "Ahr Slope" Log_SumAllST_1 1 Log_SumAllST_1*Protein 0 0 1 0 0 0 0 0 0 0 0 0 0 0 0 0 0 0 0 0 0 0 0 0 0 0 0 0 0 0 0 0 0 0
0 0 0 0 0 0 0 0 0 0 0 0 0 0 0 0 0 0 0 0 0 0 0 0 0 0 0 0 0 0 0 0 0 0 0 0 0 0 0 0 0 0 0 0 0 0 0 0 0 0 0 0 0 0 0 0 0 0  ;
Estimate "Apbb1ip Slope" Log_SumAllST_1 1 Log_SumAllST_1*Protein 0 0 0 1 0 0 0 0 0 0 0 0 0 0 0 0 0 0 0 0 0 0 0 0 0 0 0 0 0 0 0 0
0 0 0 0 0 0 0 0 0 0 0 0 0 0 0 0 0 0 0 0 0 0 0 0 0 0 0 0 0 0 0 0 0 0 0 0 0 0 0 0 0 0 0 0 0 0 0 0 0 0 0 0 0 0 0 0 0 0 0 0  ;
Estimate "Axin1 Slope" Log_SumAllST_1 1 Log_SumAllST_1*Protein 0 0 0 0 1 0 0 0 0 0 0 0 0 0 0 0 0 0 0 0 0 0 0 0 0 0 0 0 0 0 0 0 0
0 0 0 0 0 0 0 0 0 0 0 0 0 0 0 0 0 0 0 0 0 0 0 0 0 0 0 0 0 0 0 0 0 0 0 0 0 0 0 0 0 0 0 0 0 0 0 0 0 0 0 0 0 0 0 0 0 0 0  ;
Estimate "Ca13 Slope" Log_SumAllST_1 1 Log_SumAllST_1*Protein 0 0 0 0 0 1 0 0 0 0 0 0 0 0 0 0 0 0 0 0 0 0 0 0 0 0 0 0 0 0 0 0 0 0
0 0 0 0 0 0 0 0 0 0 0 0 0 0 0 0 0 0 0 0 0 0 0 0 0 0 0 0 0 0 0 0 0 0 0 0 0 0 0 0 0 0 0 0 0 0 0 0 0 0 0 0 0 0 0 0 0 0  ;
Estimate "Cant1 Slope" Log_SumAllST_1 1 Log_SumAllST_1*Protein 0 0 0 0 0 0 1 0 0 0 0 0 0 0 0 0 0 0 0 0 0 0 0 0 0 0 0 0 0 0 0 0 0
0 0 0 0 0 0 0 0 0 0 0 0 0 0 0 0 0 0 0 0 0 0 0 0 0 0 0 0 0 0 0 0 0 0 0 0 0 0 0 0 0 0 0 0 0 0 0 0 0 0 0 0 0 0 0 0 0 0 0  ;
Estimate "Casp3 Slope" Log_SumAllST_1 1 Log_SumAllST_1*Protein 0 0 0 0 0 0 0 1 0 0 0 0 0 0 0 0 0 0 0 0 0 0 0 0 0 0 0 0 0 0 0 0 0
0 0 0 0 0 0 0 0 0 0 0 0 0 0 0 0 0 0 0 0 0 0 0 0 0 0 0 0 0 0 0 0 0 0 0 0 0 0 0 0 0 0 0 0 0 0 0 0 0 0 0 0 0 0 0 0 0 0 0  ;
Estimate "Ccl2 Slope" Log_SumAllST_1 1 Log_SumAllST_1*Protein 0 0 0 0 0 0 0 0 1 0 0 0 0 0 0 0 0 0 0 0 0 0 0 0 0 0 0 0 0 0 0 0 0 0
0 0 0 0 0 0 0 0 0 0 0 0 0 0 0 0 0 0 0 0 0 0 0 0 0 0 0 0 0 0 0 0 0 0 0 0 0 0 0 0 0 0 0 0 0 0 0 0 0 0 0 0 0 0 0 0 0 0  ;
Estimate "Ccl20 Slope" Log_SumAllST_1 1 Log_SumAllST_1*Protein 0 0 0 0 0 0 0 0 0 1 0 0 0 0 0 0 0 0 0 0 0 0 0 0 0 0 0 0 0 0 0 0 0
0 0 0 0 0 0 0 0 0 0 0 0 0 0 0 0 0 0 0 0 0 0 0 0 0 0 0 0 0 0 0 0 0 0 0 0 0 0 0 0 0 0 0 0 0 0 0 0 0 0 0 0 0 0 0 0 0 0 0  ;
Estimate "Ccl3 Slope" Log_SumAllST_1 1 Log_SumAllST_1*Protein 0 0 0 0 0 0 0 0 0 0 1 0 0 0 0 0 0 0 0 0 0 0 0 0 0 0 0 0 0 0 0 0 0 0
0 0 0 0 0 0 0 0 0 0 0 0 0 0 0 0 0 0 0 0 0 0 0 0 0 0 0 0 0 0 0 0 0 0 0 0 0 0 0 0 0 0 0 0 0 0 0 0 0 0 0 0 0 0 0 0 0 0  ;
Estimate "Ccl5 Slope" Log_SumAllST_1 1 Log_SumAllST_1*Protein 0 0 0 0 0 0 0 0 0 0 0 1 0 0 0 0 0 0 0 0 0 0 0 0 0 0 0 0 0 0 0 0 0 0
0 0 0 0 0 0 0 0 0 0 0 0 0 0 0 0 0 0 0 0 0 0 0 0 0 0 0 0 0 0 0 0 0 0 0 0 0 0 0 0 0 0 0 0 0 0 0 0 0 0 0 0 0 0 0 0 0 0  ;
Estimate "Cdh6 Slope" Log_SumAllST_1 1 Log_SumAllST_1*Protein 0 0 0 0 0 0 0 0 0 0 0 0 1 0 0 0 0 0 0 0 0 0 0 0 0 0 0 0 0 0 0 0 0 0
0 0 0 0 0 0 0 0 0 0 0 0 0 0 0 0 0 0 0 0 0 0 0 0 0 0 0 0 0 0 0 0 0 0 0 0 0 0 0 0 0 0 0 0 0 0 0 0 0 0 0 0 0 0 0 0 0 0  ;
Estimate "Clmp Slope" Log_SumAllST_1 1 Log_SumAllST_1*Protein 0 0 0 0 0 0 0 0 0 0 0 0 0 1 0 0 0 0 0 0 0 0 0 0 0 0 0 0 0 0 0 0 0 0
0 0 0 0 0 0 0 0 0 0 0 0 0 0 0 0 0 0 0 0 0 0 0 0 0 0 0 0 0 0 0 0 0 0 0 0 0 0 0 0 0 0 0 0 0 0 0 0 0 0 0 0 0 0 0 0 0 0  ;
```

```
Estimate "Clstn2 Slope" Log_SumAllST_1 1 Log_SumAllST_1*Protein 0 0 0 0 0 0 0 0 0 0 0 0 0 0 1 0 0 0 0 0 0 0 0 0 0 0 0 0 0 0 0 0 0
0 0 0 0 0 0 0 0 0 0 0 0 0 0 0 0 0 0 0 0 0 0 0 0 0 0 0 0 0 0 0 0 0 0 0 0 0 0 0 0 0 0 0 0 0 0 0 0 0 0 0 0 0 0 0 0 0 0 0 ;
Estimate "Cntn1 Slope" Log_SumAllST_1 1 Log_SumAllST_1*Protein 0 0 0 0 0 0 0 0 0 0 0 0 0 0 0 1 0 0 0 0 0 0 0 0 0 0 0 0 0 0 0 0 0
0 0 0 0 0 0 0 0 0 0 0 0 0 0 0 0 0 0 0 0 0 0 0 0 0 0 0 0 0 0 0 0 0 0 0 0 0 0 0 0 0 0 0 0 0 0 0 0 0 0 0 0 0 0 0 0 0 0 0 ;
Estimate "Cntn4 Slope" Log_SumAllST_1 1 Log_SumAllST_1*Protein 0 0 0 0 0 0 0 0 0 0 0 0 0 0 0 0 1 0 0 0 0 0 0 0 0 0 0 0 0 0 0 0 0
0 0 0 0 0 0 0 0 0 0 0 0 0 0 0 0 0 0 0 0 0 0 0 0 0 0 0 0 0 0 0 0 0 0 0 0 0 0 0 0 0 0 0 0 0 0 0 0 0 0 0 0 0 0 0 0 0 0 0 ;
Estimate "Cpe Slope" Log_SumAllST_1 1 Log_SumAllST_1*Protein 0 0 0 0 0 0 0 0 0 0 0 0 0 0 0 0 0 1 0 0 0 0 0 0 0 0 0 0 0 0 0 0 0 0
0 0 0 0 0 0 0 0 0 0 0 0 0 0 0 0 0 0 0 0 0 0 0 0 0 0 0 0 0 0 0 0 0 0 0 0 0 0 0 0 0 0 0 0 0 0 0 0 0 0 0 0 0 0 0 0 0 0 ;
Estimate "Crim1 Slope" Log_SumAllST_1 1 Log_SumAllST_1*Protein 0 0 0 0 0 0 0 0 0 0 0 0 0 0 0 0 0 0 1 0 0 0 0 0 0 0 0 0 0 0 0 0 0
0 0 0 0 0 0 0 0 0 0 0 0 0 0 0 0 0 0 0 0 0 0 0 0 0 0 0 0 0 0 0 0 0 0 0 0 0 0 0 0 0 0 0 0 0 0 0 0 0 0 0 0 0 0 0 0 0 0 0 ;
Estimate "Csf2 Slope" Log_SumAllST_1 1 Log_SumAllST_1*Protein 0 0 0 0 0 0 0 0 0 0 0 0 0 0 0 0 0 0 0 1 0 0 0 0 0 0 0 0 0 0 0 0 0 0
0 0 0 0 0 0 0 0 0 0 0 0 0 0 0 0 0 0 0 0 0 0 0 0 0 0 0 0 0 0 0 0 0 0 0 0 0 0 0 0 0 0 0 0 0 0 0 0 0 0 0 0 0 0 0 0 0 0 ;
Estimate "Cxcl1 Slope" Log_SumAllST_1 1 Log_SumAllST_1*Protein 0 0 0 0 0 0 0 0 0 0 0 0 0 0 0 0 0 0 0 0 1 0 0 0 0 0 0 0 0 0 0 0 0
0 0 0 0 0 0 0 0 0 0 0 0 0 0 0 0 0 0 0 0 0 0 0 0 0 0 0 0 0 0 0 0 0 0 0 0 0 0 0 0 0 0 0 0 0 0 0 0 0 0 0 0 0 0 0 0 0 0 0 ;
Estimate "Cxcl9 Slope" Log_SumAllST_1 1 Log_SumAllST_1*Protein 0 0 0 0 0 0 0 0 0 0 0 0 0 0 0 0 0 0 0 0 0 1 0 0 0 0 0 0 0 0 0 0 0
0 0 0 0 0 0 0 0 0 0 0 0 0 0 0 0 0 0 0 0 0 0 0 0 0 0 0 0 0 0 0 0 0 0 0 0 0 0 0 0 0 0 0 0 0 0 0 0 0 0 0 0 0 0 0 0 0 0 0 ;
Estimate "Cyr61 Slope" Log_SumAllST_1 1 Log_SumAllST_1*Protein 0 0 0 0 0 0 0 0 0 0 0 0 0 0 0 0 0 0 0 0 0 0 1 0 0 0 0 0 0 0 0 0 0
0 0 0 0 0 0 0 0 0 0 0 0 0 0 0 0 0 0 0 0 0 0 0 0 0 0 0 0 0 0 0 0 0 0 0 0 0 0 0 0 0 0 0 0 0 0 0 0 0 0 0 0 0 0 0 0 0 0 0 ;
Estimate "Dctn2 Slope" Log_SumAllST_1 1 Log_SumAllST_1*Protein 0 0 0 0 0 0 0 0 0 0 0 0 0 0 0 0 0 0 0 0 0 0 0 1 0 0 0 0 0 0 0 0 0
0 0 0 0 0 0 0 0 0 0 0 0 0 0 0 0 0 0 0 0 0 0 0 0 0 0 0 0 0 0 0 0 0 0 0 0 0 0 0 0 0 0 0 0 0 0 0 0 0 0 0 0 0 0 0 0 0 0 0 ;
Estimate "Ddah1 Slope" Log_SumAllST_1 1 Log_SumAllST_1*Protein 0 0 0 0 0 0 0 0 0 0 0 0 0 0 0 0 0 0 0 0 0 0 0 0 1 0 0 0 0 0 0 0 0
0 0 0 0 0 0 0 0 0 0 0 0 0 0 0 0 0 0 0 0 0 0 0 0 0 0 0 0 0 0 0 0 0 0 0 0 0 0 0 0 0 0 0 0 0 0 0 0 0 0 0 0 0 0 0 0 0 0 0 ;
Estimate "Dlk1 Slope" Log_SumAllST_1 1 Log_SumAllST_1*Protein 0 0 0 0 0 0 0 0 0 0 0 0 0 0 0 0 0 0 0 0 0 0 0 0 0 1 0 0 0 0 0 0 0 0
0 0 0 0 0 0 0 0 0 0 0 0 0 0 0 0 0 0 0 0 0 0 0 0 0 0 0 0 0 0 0 0 0 0 0 0 0 0 0 0 0 0 0 0 0 0 0 0 0 0 0 0 0 0 0 0 0 0 ;
Estimate "Dll1 Slope" Log_SumAllST_1 1 Log_SumAllST_1*Protein 0 0 0 0 0 0 0 0 0 0 0 0 0 0 0 0 0 0 0 0 0 0 0 0 0 0 1 0 0 0 0 0 0 0
0 0 0 0 0 0 0 0 0 0 0 0 0 0 0 0 0 0 0 0 0 0 0 0 0 0 0 0 0 0 0 0 0 0 0 0 0 0 0 0 0 0 0 0 0 0 0 0 0 0 0 0 0 0 0 0 0 0 ;
Estimate "Eda2r Slope" Log_SumAllST_1 1 Log_SumAllST_1*Protein 0 0 0 0 0 0 0 0 0 0 0 0 0 0 0 0 0 0 0 0 0 0 0 0 0 0 0 1 0 0 0 0 0
0 0 0 0 0 0 0 0 0 0 0 0 0 0 0 0 0 0 0 0 0 0 0 0 0 0 0 0 0 0 0 0 0 0 0 0 0 0 0 0 0 0 0 0 0 0 0 0 0 0 0 0 0 0 0 0 0 0 0 ;
Estimate "Eno2 Slope" Log_SumAllST_1 1 Log_SumAllST_1*Protein 0 0 0 0 0 0 0 0 0 0 0 0 0 0 0 0 0 0 0 0 0 0 0 0 0 0 0 0 1 0 0 0 0 0
0 0 0 0 0 0 0 0 0 0 0 0 0 0 0 0 0 0 0 0 0 0 0 0 0 0 0 0 0 0 0 0 0 0 0 0 0 0 0 0 0 0 0 0 0 0 0 0 0 0 0 0 0 0 0 0 0 0 ;
Estimate "Epcam Slope" Log_SumAllST_1 1 Log_SumAllST_1*Protein 0 0 0 0 0 0 0 0 0 0 0 0 0 0 0 0 0 0 0 0 0 0 0 0 0 0 0 0 0 1 0 0 0
0 0 0 0 0 0 0 0 0 0 0 0 0 0 0 0 0 0 0 0 0 0 0 0 0 0 0 0 0 0 0 0 0 0 0 0 0 0 0 0 0 0 0 0 0 0 0 0 0 0 0 0 0 0 0 0 0 0 0 ;
Estimate "Epo Slope" Log_SumAllST_1 1 Log_SumAllST_1*Protein 0 0 0 0 0 0 0 0 0 0 0 0 0 0 0 0 0 0 0 0 0 0 0 0 0 0 0 0 0 0 1 0 0 0
0 0 0 0 0 0 0 0 0 0 0 0 0 0 0 0 0 0 0 0 0 0 0 0 0 0 0 0 0 0 0 0 0 0 0 0 0 0 0 0 0 0 0 0 0 0 0 0 0 0 0 0 0 0 0 0 0 0 ;
Estimate "Erbb4 Slope" Log_SumAllST_1 1 Log_SumAllST_1*Protein 0 0 0 0 0 0 0 0 0 0 0 0 0 0 0 0 0 0 0 0 0 0 0 0 0 0 0 0 0 0 0 1 0
0 0 0 0 0 0 0 0 0 0 0 0 0 0 0 0 0 0 0 0 0 0 0 0 0 0 0 0 0 0 0 0 0 0 0 0 0 0 0 0 0 0 0 0 0 0 0 0 0 0 0 0 0 0 0 0 0 0 0 ;
Estimate "Fas Slope" Log_SumAllST_1 1 Log_SumAllST_1*Protein 0 0 0 0 0 0 0 0 0 0 0 0 0 0 0 0 0 0 0 0 0 0 0 0 0 0 0 0 0 0 0 0 1 0
0 0 0 0 0 0 0 0 0 0 0 0 0 0 0 0 0 0 0 0 0 0 0 0 0 0 0 0 0 0 0 0 0 0 0 0 0 0 0 0 0 0 0 0 0 0 0 0 0 0 0 0 0 0 0 0 0 0 ;
Estimate "Fli1 Slope" Log_SumAllST_1 1 Log_SumAllST_1*Protein 0 0 0 0 0 0 0 0 0 0 0 0 0 0 0 0 0 0 0 0 0 0 0 0 0 0 0 0 0 0 0 0 0 1
0 0 0 0 0 0 0 0 0 0 0 0 0 0 0 0 0 0 0 0 0 0 0 0 0 0 0 0 0 0 0 0 0 0 0 0 0 0 0 0 0 0 0 0 0 0 0 0 0 0 0 0 0 0 0 0 0 0 ;
Estimate "Flrt2 Slope" Log_SumAllST_1 1 Log_SumAllST_1*Protein 0 0 0 0 0 0 0 0 0 0 0 0 0 0 0 0 0 0 0 0 0 0 0 0 0 0 0 0 0 0 0 0 0
0 1 0 0 0 0 0 0 0 0 0 0 0 0 0 0 0 0 0 0 0 0 0 0 0 0 0 0 0 0 0 0 0 0 0 0 0 0 0 0 0 0 0 0 0 0 0 0 0 0 0 0 0 0 0 0 0 0 0 ;
Estimate "Foxo1 Slope" Log_SumAllST_1 1 Log_SumAllST_1*Protein 0 0 0 0 0 0 0 0 0 0 0 0 0 0 0 0 0 0 0 0 0 0 0 0 0 0 0 0 0 0 0 0 0
0 0 1 0 0 0 0 0 0 0 0 0 0 0 0 0 0 0 0 0 0 0 0 0 0 0 0 0 0 0 0 0 0 0 0 0 0 0 0 0 0 0 0 0 0 0 0 0 0 0 0 0 0 0 0 0 0 0 0 ;
Estimate "Fst Slope" Log_SumAllST_1 1 Log_SumAllST_1*Protein 0 0 0 0 0 0 0 0 0 0 0 0 0 0 0 0 0 0 0 0 0 0 0 0 0 0 0 0 0 0 0 0 0 0
0 0 1 0 0 0 0 0 0 0 0 0 0 0 0 0 0 0 0 0 0 0 0 0 0 0 0 0 0 0 0 0 0 0 0 0 0 0 0 0 0 0 0 0 0 0 0 0 0 0 0 0 0 0 0 0 0 0 ;
Estimate "Fstl3 Slope" Log_SumAllST_1 1 Log_SumAllST_1*Protein 0 0 0 0 0 0 0 0 0 0 0 0 0 0 0 0 0 0 0 0 0 0 0 0 0 0 0 0 0 0 0 0 0
0 0 0 0 1 0 0 0 0 0 0 0 0 0 0 0 0 0 0 0 0 0 0 0 0 0 0 0 0 0 0 0 0 0 0 0 0 0 0 0 0 0 0 0 0 0 0 0 0 0 0 0 0 0 0 0 0 0 0 ;
Estimate "Gcg Slope" Log_SumAllST_1 1 Log_SumAllST_1*Protein 0 0 0 0 0 0 0 0 0 0 0 0 0 0 0 0 0 0 0 0 0 0 0 0 0 0 0 0 0 0 0 0 0 0
0 0 0 0 1 0 0 0 0 0 0 0 0 0 0 0 0 0 0 0 0 0 0 0 0 0 0 0 0 0 0 0 0 0 0 0 0 0 0 0 0 0 0 0 0 0 0 0 0 0 0 0 0 0 0 0 0 0 ;
Estimate "Gdnf Slope" Log_SumAllST_1 1 Log_SumAllST_1*Protein 0 0 0 0 0 0 0 0 0 0 0 0 0 0 0 0 0 0 0 0 0 0 0 0 0 0 0 0 0 0 0 0 0 0
0 0 0 0 0 1 0 0 0 0 0 0 0 0 0 0 0 0 0 0 0 0 0 0 0 0 0 0 0 0 0 0 0 0 0 0 0 0 0 0 0 0 0 0 0 0 0 0 0 0 0 0 0 0 0 0 0 0 ;
Estimate "Gfra1 Slope" Log_SumAllST_1 1 Log_SumAllST_1*Protein 0 0 0 0 0 0 0 0 0 0 0 0 0 0 0 0 0 0 0 0 0 0 0 0 0 0 0 0 0 0 0 0 0
0 0 0 0 0 0 0 1 0 0 0 0 0 0 0 0 0 0 0 0 0 0 0 0 0 0 0 0 0 0 0 0 0 0 0 0 0 0 0 0 0 0 0 0 0 0 0 0 0 0 0 0 0 0 0 0 0 0 0 ;
Estimate "Ghrl Slope" Log_SumAllST_1 1 Log_SumAllST_1*Protein 0 0 0 0 0 0 0 0 0 0 0 0 0 0 0 0 0 0 0 0 0 0 0 0 0 0 0 0 0 0 0 0 0 0
0 0 0 0 0 0 0 1 0 0 0 0 0 0 0 0 0 0 0 0 0 0 0 0 0 0 0 0 0 0 0 0 0 0 0 0 0 0 0 0 0 0 0 0 0 0 0 0 0 0 0 0 0 0 0 0 0 0 ;
Estimate "Hgf Slope" Log_SumAllST_1 1 Log_SumAllST_1*Protein 0 0 0 0 0 0 0 0 0 0 0 0 0 0 0 0 0 0 0 0 0 0 0 0 0 0 0 0 0 0 0 0 0 0
0 0 0 0 0 0 0 0 1 0 0 0 0 0 0 0 0 0 0 0 0 0 0 0 0 0 0 0 0 0 0 0 0 0 0 0 0 0 0 0 0 0 0 0 0 0 0 0 0 0 0 0 0 0 0 0 0 0 ;
Estimate "Igsf3 Slope" Log_SumAllST_1 1 Log_SumAllST_1*Protein 0 0 0 0 0 0 0 0 0 0 0 0 0 0 0 0 0 0 0 0 0 0 0 0 0 0 0 0 0 0 0 0 0
0 0 0 0 0 0 0 0 0 0 1 0 0 0 0 0 0 0 0 0 0 0 0 0 0 0 0 0 0 0 0 0 0 0 0 0 0 0 0 0 0 0 0 0 0 0 0 0 0 0 0 0 0 0 0 0 0 0 0 ;
Estimate "Il10 Slope" Log_SumAllST_1 1 Log_SumAllST_1*Protein 0 0 0 0 0 0 0 0 0 0 0 0 0 0 0 0 0 0 0 0 0 0 0 0 0 0 0 0 0 0 0 0 0 0
0 0 0 0 0 0 0 0 0 0 1 0 0 0 0 0 0 0 0 0 0 0 0 0 0 0 0 0 0 0 0 0 0 0 0 0 0 0 0 0 0 0 0 0 0 0 0 0 0 0 0 0 0 0 0 0 0 0 ;
Estimate "Il17a Slope" Log_SumAllST_1 1 Log_SumAllST_1*Protein 0 0 0 0 0 0 0 0 0 0 0 0 0 0 0 0 0 0 0 0 0 0 0 0 0 0 0 0 0 0 0 0 0
0 0 0 0 0 0 0 0 0 0 0 0 1 0 0 0 0 0 0 0 0 0 0 0 0 0 0 0 0 0 0 0 0 0 0 0 0 0 0 0 0 0 0 0 0 0 0 0 0 0 0 0 0 0 0 0 0 0 0 ;
Estimate "Il17f Slope" Log_SumAllST_1 1 Log_SumAllST_1*Protein 0 0 0 0 0 0 0 0 0 0 0 0 0 0 0 0 0 0 0 0 0 0 0 0 0 0 0 0 0 0 0 0 0
0 0 0 0 0 0 0 0 0 0 0 0 0 1 0 0 0 0 0 0 0 0 0 0 0 0 0 0 0 0 0 0 0 0 0 0 0 0 0 0 0 0 0 0 0 0 0 0 0 0 0 0 0 0 0 0 0 0 0 ;
Estimate "Il1a Slope" Log_SumAllST_1 1 Log_SumAllST_1*Protein 0 0 0 0 0 0 0 0 0 0 0 0 0 0 0 0 0 0 0 0 0 0 0 0 0 0 0 0 0 0 0 0 0 0
0 0 0 0 0 0 0 0 0 0 0 0 0 1 0 0 0 0 0 0 0 0 0 0 0 0 0 0 0 0 0 0 0 0 0 0 0 0 0 0 0 0 0 0 0 0 0 0 0 0 0 0 0 0 0 0 0 0 ;
Estimate "Il1b Slope" Log_SumAllST_1 1 Log_SumAllST_1*Protein 0 0 0 0 0 0 0 0 0 0 0 0 0 0 0 0 0 0 0 0 0 0 0 0 0 0 0 0 0 0 0 0 0 0
0 0 0 0 0 0 0 0 0 0 0 0 0 0 1 0 0 0 0 0 0 0 0 0 0 0 0 0 0 0 0 0 0 0 0 0 0 0 0 0 0 0 0 0 0 0 0 0 0 0 0 0 0 0 0 0 0 0 ;
Estimate "Il23r Slope" Log_SumAllST_1 1 Log_SumAllST_1*Protein 0 0 0 0 0 0 0 0 0 0 0 0 0 0 0 0 0 0 0 0 0 0 0 0 0 0 0 0 0 0 0 0 0
0 0 0 0 0 0 0 0 0 0 0 0 0 0 0 0 1 0 0 0 0 0 0 0 0 0 0 0 0 0 0 0 0 0 0 0 0 0 0 0 0 0 0 0 0 0 0 0 0 0 0 0 0 0 0 0 0 0 0 ;
Estimate "Il5 Slope" Log_SumAllST_1 1 Log_SumAllST_1*Protein 0 0 0 0 0 0 0 0 0 0 0 0 0 0 0 0 0 0 0 0 0 0 0 0 0 0 0 0 0 0 0 0 0 0
0 0 0 0 0 0 0 0 0 0 0 0 0 0 0 0 1 0 0 0 0 0 0 0 0 0 0 0 0 0 0 0 0 0 0 0 0 0 0 0 0 0 0 0 0 0 0 0 0 0 0 0 0 0 0 0 0 0 ;
Estimate "Il6 Slope" Log_SumAllST_1 1 Log_SumAllST_1*Protein 0 0 0 0 0 0 0 0 0 0 0 0 0 0 0 0 0 0 0 0 0 0 0 0 0 0 0 0 0 0 0 0 0 0
0 0 0 0 0 0 0 0 0 0 0 0 0 0 0 0 0 1 0 0 0 0 0 0 0 0 0 0 0 0 0 0 0 0 0 0 0 0 0 0 0 0 0 0 0 0 0 0 0 0 0 0 0 0 0 0 0 0 ;
Estimate "Itgb1bp2 Slope" Log_SumAllST_1 1 Log_SumAllST_1*Protein 0 0 0 0 0 0 0 0 0 0 0 0 0 0 0 0 0 0 0 0 0 0 0 0 0 0 0 0 0 0 0 0
0 0 0 0 0 0 0 0 0 0 0 0 0 0 0 0 0 0 0 0 1 0 0 0 0 0 0 0 0 0 0 0 0 0 0 0 0 0 0 0 0 0 0 0 0 0 0 0 0 0 0 0 0 0 0 0 0 0 0 0 ;
Estimate "Itgb6 Slope" Log_SumAllST_1 1 Log_SumAllST_1*Protein 0 0 0 0 0 0 0 0 0 0 0 0 0 0 0 0 0 0 0 0 0 0 0 0 0 0 0 0 0 0 0 0 0
0 0 0 0 0 0 0 0 0 0 0 0 0 0 0 0 0 0 0 0 1 0 0 0 0 0 0 0 0 0 0 0 0 0 0 0 0 0 0 0 0 0 0 0 0 0 0 0 0 0 0 0 0 0 0 0 0 0 0 ;
Estimate "Kitlg Slope" Log_SumAllST_1 1 Log_SumAllST_1*Protein 0 0 0 0 0 0 0 0 0 0 0 0 0 0 0 0 0 0 0 0 0 0 0 0 0 0 0 0 0 0 0 0 0
0 0 0 0 0 0 0 0 0 0 0 0 0 0 0 0 0 0 0 0 0 1 0 0 0 0 0 0 0 0 0 0 0 0 0 0 0 0 0 0 0 0 0 0 0 0 0 0 0 0 0 0 0 0 0 0 0 0 0 ;
Estimate "Lgmn Slope" Log_SumAllST_1 1 Log_SumAllST_1*Protein 0 0 0 0 0 0 0 0 0 0 0 0 0 0 0 0 0 0 0 0 0 0 0 0 0 0 0 0 0 0 0 0 0 0
0 0 0 0 0 0 0 0 0 0 0 0 0 0 0 0 0 0 0 0 0 1 0 0 0 0 0 0 0 0 0 0 0 0 0 0 0 0 0 0 0 0 0 0 0 0 0 0 0 0 0 0 0 0 0 0 0 0 ;
Estimate "Lpl Slope" Log_SumAllST_1 1 Log_SumAllST_1*Protein 0 0 0 0 0 0 0 0 0 0 0 0 0 0 0 0 0 0 0 0 0 0 0 0 0 0 0 0 0 0 0 0 0 0
0 0 0 0 0 0 0 0 0 0 0 0 0 0 0 0 0 0 0 0 0 0 1 0 0 0 0 0 0 0 0 0 0 0 0 0 0 0 0 0 0 0 0 0 0 0 0 0 0 0 0 0 0 0 0 0 0 0 ;
Estimate "Map2k6 Slope" Log_SumAllST_1 1 Log_SumAllST_1*Protein 0 0 0 0 0 0 0 0 0 0 0 0 0 0 0 0 0 0 0 0 0 0 0 0 0 0 0 0 0 0 0 0 0
0 0 0 0 0 0 0 0 0 0 0 0 0 0 0 0 0 0 0 0 0 0 0 0 1 0 0 0 0 0 0 0 0 0 0 0 0 0 0 0 0 0 0 0 0 0 0 0 0 0 0 0 0 0 0 0 0 0 0 ;
Estimate "Matn2 Slope" Log_SumAllST_1 1 Log_SumAllST_1*Protein 0 0 0 0 0 0 0 0 0 0 0 0 0 0 0 0 0 0 0 0 0 0 0 0 0 0 0 0 0 0 0 0 0
0 0 0 0 0 0 0 0 0 0 0 0 0 0 0 0 0 0 0 0 0 0 0 0 0 1 0 0 0 0 0 0 0 0 0 0 0 0 0 0 0 0 0 0 0 0 0 0 0 0 0 0 0 0 0 0 0 0 0 ;
Estimate "Mia Slope" Log_SumAllST_1 1 Log_SumAllST_1*Protein 0 0 0 0 0 0 0 0 0 0 0 0 0 0 0 0 0 0 0 0 0 0 0 0 0 0 0 0 0 0 0 0 0 0
0 0 0 0 0 0 0 0 0 0 0 0 0 0 0 0 0 0 0 0 0 0 0 0 0 1 0 0 0 0 0 0 0 0 0 0 0 0 0 0 0 0 0 0 0 0 0 0 0 0 0 0 0 0 0 0 0 0 ;
Estimate "Nadk Slope" Log_SumAllST_1 1 Log_SumAllST_1*Protein 0 0 0 0 0 0 0 0 0 0 0 0 0 0 0 0 0 0 0 0 0 0 0 0 0 0 0 0 0 0 0 0 0 0
0 0 0 0 0 0 0 0 0 0 0 0 0 0 0 0 0 0 0 0 0 0 0 0 0 0 1 0 0 0 0 0 0 0 0 0 0 0 0 0 0 0 0 0 0 0 0 0 0 0 0 0 0 0 0 0 0 0 ;
```

```
Estimate "Notch3 Slope" Log_SumAllST_1 1 Log_SumAllST_1*Protein 0 0 0 0 0 0 0 0 0 0 0 0 0 0 0 0 0 0 0 0 0 0 0 0 0 0 0 0 0 0 0 0 0
0 0 0 0 0 0 0 0 0 0 0 0 0 0 0 0 0 0 0 0 0 0 0 0 0 0 0 0 1 0 0 0 0 0 0 0 0 0 0 0 0 0 0 0 0 0 0 0 0 0 0 0 0 0 0 0 0 0 0 ;
Estimate "Ntf3 Slope" Log_SumAllST_1 1 Log_SumAllST_1*Protein 0 0 0 0 0 0 0 0 0 0 0 0 0 0 0 0 0 0 0 0 0 0 0 0 0 0 0 0 0 0 0 0 0 0
0 0 0 0 0 0 0 0 0 0 0 0 0 0 0 0 0 0 0 0 0 0 0 0 0 0 0 0 1 0 0 0 0 0 0 0 0 0 0 0 0 0 0 0 0 0 0 0 0 0 0 0 0 0 0 0 0 0 ;
Estimate "Pak4 Slope" Log_SumAllST_1 1 Log_SumAllST_1*Protein 0 0 0 0 0 0 0 0 0 0 0 0 0 0 0 0 0 0 0 0 0 0 0 0 0 0 0 0 0 0 0 0 0 0
0 0 0 0 0 0 0 0 0 0 0 0 0 0 0 0 0 0 0 0 0 0 0 0 0 0 0 0 0 1 0 0 0 0 0 0 0 0 0 0 0 0 0 0 0 0 0 0 0 0 0 0 0 0 0 0 0 0 ;
Estimate "Parp1 Slope" Log_SumAllST_1 1 Log_SumAllST_1*Protein 0 0 0 0 0 0 0 0 0 0 0 0 0 0 0 0 0 0 0 0 0 0 0 0 0 0 0 0 0 0 0 0 0
0 0 0 0 0 0 0 0 0 0 0 0 0 0 0 0 0 0 0 0 0 0 0 0 0 0 0 0 0 0 0 1 0 0 0 0 0 0 0 0 0 0 0 0 0 0 0 0 0 0 0 0 0 0 0 0 0 0 0 ;
Estimate "Pdgfb Slope" Log_SumAllST_1 1 Log_SumAllST_1*Protein 0 0 0 0 0 0 0 0 0 0 0 0 0 0 0 0 0 0 0 0 0 0 0 0 0 0 0 0 0 0 0 0 0
0 0 0 0 0 0 0 0 0 0 0 0 0 0 0 0 0 0 0 0 0 0 0 0 0 0 0 0 0 0 0 0 1 0 0 0 0 0 0 0 0 0 0 0 0 0 0 0 0 0 0 0 0 0 0 0 0 0 0 ;
Estimate "Pla2g4a Slope" Log_SumAllST_1 1 Log_SumAllST_1*Protein 0 0 0 0 0 0 0 0 0 0 0 0 0 0 0 0 0 0 0 0 0 0 0 0 0 0 0 0 0 0 0 0
0 0 0 0 0 0 0 0 0 0 0 0 0 0 0 0 0 0 0 0 0 0 0 0 0 0 0 0 0 0 0 0 0 0 1 0 0 0 0 0 0 0 0 0 0 0 0 0 0 0 0 0 0 0 0 0 0 0 0 0 ;
Estimate "Plin1 Slope" Log_SumAllST_1 1 Log_SumAllST_1*Protein 0 0 0 0 0 0 0 0 0 0 0 0 0 0 0 0 0 0 0 0 0 0 0 0 0 0 0 0 0 0 0 0 0
0 0 0 0 0 0 0 0 0 0 0 0 0 0 0 0 0 0 0 0 0 0 0 0 0 0 0 0 0 0 0 0 0 0 1 0 0 0 0 0 0 0 0 0 0 0 0 0 0 0 0 0 0 0 0 0 0 0 0 ;
Estimate "Plxna4 Slope" Log_SumAllST_1 1 Log_SumAllST_1*Protein 0 0 0 0 0 0 0 0 0 0 0 0 0 0 0 0 0 0 0 0 0 0 0 0 0 0 0 0 0 0 0 0 0
0 0 0 0 0 0 0 0 0 0 0 0 0 0 0 0 0 0 0 0 0 0 0 0 0 0 0 0 0 0 0 0 0 0 0 1 0 0 0 0 0 0 0 0 0 0 0 0 0 0 0 0 0 0 0 0 0 0 0 ;
Estimate "Ppp1r2 Slope" Log_SumAllST_1 1 Log_SumAllST_1*Protein 0 0 0 0 0 0 0 0 0 0 0 0 0 0 0 0 0 0 0 0 0 0 0 0 0 0 0 0 0 0 0 0 0
0 0 0 0 0 0 0 0 0 0 0 0 0 0 0 0 0 0 0 0 0 0 0 0 0 0 0 0 0 0 0 0 0 0 0 0 1 0 0 0 0 0 0 0 0 0 0 0 0 0 0 0 0 0 0 0 0 0 0 ;
Estimate "Prdx5 Slope" Log_SumAllST_1 1 Log_SumAllST_1*Protein 0 0 0 0 0 0 0 0 0 0 0 0 0 0 0 0 0 0 0 0 0 0 0 0 0 0 0 0 0 0 0 0 0
0 0 0 0 0 0 0 0 0 0 0 0 0 0 0 0 0 0 0 0 0 0 0 0 0 0 0 0 0 0 0 0 0 0 0 0 0 1 0 0 0 0 0 0 0 0 0 0 0 0 0 0 0 0 0 0 0 0 0 ;
Estimate "Qdpr Slope" Log_SumAllST_1 1 Log_SumAllST_1*Protein 0 0 0 0 0 0 0 0 0 0 0 0 0 0 0 0 0 0 0 0 0 0 0 0 0 0 0 0 0 0 0 0 0 0
0 0 0 0 0 0 0 0 0 0 0 0 0 0 0 0 0 0 0 0 0 0 0 0 0 0 0 0 0 0 0 0 0 0 0 0 0 1 0 0 0 0 0 0 0 0 0 0 0 0 0 0 0 0 0 0 0 0 ;
Estimate "Rgma Slope" Log_SumAllST_1 1 Log_SumAllST_1*Protein 0 0 0 0 0 0 0 0 0 0 0 0 0 0 0 0 0 0 0 0 0 0 0 0 0 0 0 0 0 0 0 0 0 0
0 0 0 0 0 0 0 0 0 0 0 0 0 0 0 0 0 0 0 0 0 0 0 0 0 0 0 0 0 0 0 0 0 0 0 0 0 0 1 0 0 0 0 0 0 0 0 0 0 0 0 0 0 0 0 0 0 0 ;
Estimate "Riox2 Slope" Log_SumAllST_1 1 Log_SumAllST_1*Protein 0 0 0 0 0 0 0 0 0 0 0 0 0 0 0 0 0 0 0 0 0 0 0 0 0 0 0 0 0 0 0 0 0
0 0 0 0 0 0 0 0 0 0 0 0 0 0 0 0 0 0 0 0 0 0 0 0 0 0 0 0 0 0 0 0 0 0 0 0 0 0 0 0 1 0 0 0 0 0 0 0 0 0 0 0 0 0 0 0 0 0 0 ;
Estimate "S100a4 Slope" Log_SumAllST_1 1 Log_SumAllST_1*Protein 0 0 0 0 0 0 0 0 0 0 0 0 0 0 0 0 0 0 0 0 0 0 0 0 0 0 0 0 0 0 0 0 0
0 0 0 0 0 0 0 0 0 0 0 0 0 0 0 0 0 0 0 0 0 0 0 0 0 0 0 0 0 0 0 0 0 0 0 0 0 0 0 0 0 1 0 0 0 0 0 0 0 0 0 0 0 0 0 0 0 0 0 ;
Estimate "Sez6l2 Slope" Log_SumAllST_1 1 Log_SumAllST_1*Protein 0 0 0 0 0 0 0 0 0 0 0 0 0 0 0 0 0 0 0 0 0 0 0 0 0 0 0 0 0 0 0 0 0
0 0 0 0 0 0 0 0 0 0 0 0 0 0 0 0 0 0 0 0 0 0 0 0 0 0 0 0 0 0 0 0 0 0 0 0 0 0 0 0 0 0 1 0 0 0 0 0 0 0 0 0 0 0 0 0 0 0 0 ;
Estimate "Snap29 Slope" Log_SumAllST_1 1Log_SumAllST_1*Protein 0 0 0 0 0 0 0 0 0 0 0 0 0 0 0 0 0 0 0 0 0 0 0 0 0 0 0 0 0 0 0 0 0
0 0 0 0 0 0 0 0 0 0 0 0 0 0 0 0 0 0 0 0 0 0 0 0 0 0 0 0 0 0 0 0 0 0 0 0 0 0 0 0 0 0 0 1 0 0 0 0 0 0 0 0 0 0 0 0 0 0 0 ;
Estimate "Tgfa Slope" Log_SumAllST_1 1Log_SumAllST_1*Protein 0 0 0 0 0 0 0 0 0 0 0 0 0 0 0 0 0 0 0 0 0 0 0 0 0 0 0 0 0 0 0 0 0 0
0 0 0 0 0 0 0 0 0 0 0 0 0 0 0 0 0 0 0 0 0 0 0 0 0 0 0 0 0 0 0 0 0 0 0 0 0 0 0 0 0 0 0 1 0 0 0 0 0 0 0 0 0 0 0 0 0 0 ;
Estimate "Tgfb1 Slope" Log_SumAllST_1 1Log_SumAllST_1*Protein 0 0 0 0 0 0 0 0 0 0 0 0 0 0 0 0 0 0 0 0 0 0 0 0 0 0 0 0 0 0 0 0 0 0
0 0 0 0 0 0 0 0 0 0 0 0 0 0 0 0 0 0 0 0 0 0 0 0 0 0 0 0 0 0 0 0 0 0 0 0 0 0 0 0 0 0 0 0 1 0 0 0 0 0 0 0 0 0 0 0 0 0 ;
Estimate "Tgfbr3 Slope" Log_SumAllST_1 1Log_SumAllST_1*Protein 0 0 0 0 0 0 0 0 0 0 0 0 0 0 0 0 0 0 0 0 0 0 0 0 0 0 0 0 0 0 0 0 0
0 0 0 0 0 0 0 0 0 0 0 0 0 0 0 0 0 0 0 0 0 0 0 0 0 0 0 0 0 0 0 0 0 0 0 0 0 0 0 0 0 0 0 0 0 0 1 0 0 0 0 0 0 0 0 0 0 0 0 ;
Estimate "Tnf Slope" Log_SumAllST_1 1Log_SumAllST_1*Protein 0 0 0 0 0 0 0 0 0 0 0 0 0 0 0 0 0 0 0 0 0 0 0 0 0 0 0 0 0 0 0 0 0 0 0
0 0 0 0 0 0 0 0 0 0 0 0 0 0 0 0 0 0 0 0 0 0 0 0 0 0 0 0 0 0 0 0 0 0 0 0 0 0 0 0 0 0 0 0 0 1 0 0 0 0 0 0 0 0 0 0 0 ;
Estimate "Tnfrsf11b Slope" Log_SumAllST_1 1Log_SumAllST_1*Protein 0 0 0 0 0 0 0 0 0 0 0 0 0 0 0 0 0 0 0 0 0 0 0 0 0 0 0 0 0 0 0 0
0 0 0 0 0 0 0 0 0 0 0 0 0 0 0 0 0 0 0 0 0 0 0 0 0 0 0 0 0 0 0 0 0 0 0 0 0 0 0 0 0 0 0 0 0 0 0 0 0 1 0 0 0 0 0 0 0 0 0 0 ;
Estimate "Tnfrsf12a Slope" Log_SumAllST_1 1Log_SumAllST_1*Protein 0 0 0 0 0 0 0 0 0 0 0 0 0 0 0 0 0 0 0 0 0 0 0 0 0 0 0 0 0 0 0 0
0 0 0 0 0 0 0 0 0 0 0 0 0 0 0 0 0 0 0 0 0 0 0 0 0 0 0 0 0 0 0 0 0 0 0 0 0 0 0 0 0 0 0 0 0 0 0 0 0 0 1 0 0 0 0 0 0 0 0 0 ;
Estimate "Tnfsf12 Slope" Log_SumAllST_1 1Log_SumAllST_1*Protein 0 0 0 0 0 0 0 0 0 0 0 0 0 0 0 0 0 0 0 0 0 0 0 0 0 0 0 0 0 0 0 0 0
0 0 0 0 0 0 0 0 0 0 0 0 0 0 0 0 0 0 0 0 0 0 0 0 0 0 0 0 0 0 0 0 0 0 0 0 0 0 0 0 0 0 0 0 0 0 0 0 0 0 1 0 0 0 0 0 0 0 0 ;
Estimate "Tnni3 Slope" Log_SumAllST_1 1Log_SumAllST_1*Protein 0 0 0 0 0 0 0 0 0 0 0 0 0 0 0 0 0 0 0 0 0 0 0 0 0 0 0 0 0 0 0 0 0 0
0 0 0 0 0 0 0 0 0 0 0 0 0 0 0 0 0 0 0 0 0 0 0 0 0 0 0 0 0 0 0 0 0 0 0 0 0 0 0 0 0 0 0 0 0 0 0 0 0 0 1 0 0 0 0 0 0 0 ;
Estimate "Tnr Slope" Log_SumAllST_1 1Log_SumAllST_1*Protein 0 0 0 0 0 0 0 0 0 0 0 0 0 0 0 0 0 0 0 0 0 0 0 0 0 0 0 0 0 0 0 0 0 0 0
0 0 0 0 0 0 0 0 0 0 0 0 0 0 0 0 0 0 0 0 0 0 0 0 0 0 0 0 0 0 0 0 0 0 0 0 0 0 0 0 0 0 0 0 0 0 0 0 0 0 1 0 0 0 0 0 0 ;
Estimate "Tpp1 Slope" Log_SumAllST_1 1Log_SumAllST_1*Protein 0 0 0 0 0 0 0 0 0 0 0 0 0 0 0 0 0 0 0 0 0 0 0 0 0 0 0 0 0 0 0 0 0 0
0 0 0 0 0 0 0 0 0 0 0 0 0 0 0 0 0 0 0 0 0 0 0 0 0 0 0 0 0 0 0 0 0 0 0 0 0 0 0 0 0 0 0 0 0 0 0 0 0 0 0 0 1 0 0 0 0 0 ;
Estimate "Vegfd Slope" Log_SumAllST_1 1Log_SumAllST_1*Protein 0 0 0 0 0 0 0 0 0 0 0 0 0 0 0 0 0 0 0 0 0 0 0 0 0 0 0 0 0 0 0 0 0 0
0 0 0 0 0 0 0 0 0 0 0 0 0 0 0 0 0 0 0 0 0 0 0 0 0 0 0 0 0 0 0 0 0 0 0 0 0 0 0 0 0 0 0 0 0 0 0 0 0 0 0 0 0 1 0 0 0 0 ;
Estimate "Vsig2 Slope" Log_SumAllST_1 1Log_SumAllST_1*Protein 0 0 0 0 0 0 0 0 0 0 0 0 0 0 0 0 0 0 0 0 0 0 0 0 0 0 0 0 0 0 0 0 0 0
0 0 0 0 0 0 0 0 0 0 0 0 0 0 0 0 0 0 0 0 0 0 0 0 0 0 0 0 0 0 0 0 0 0 0 0 0 0 0 0 0 0 0 0 0 0 0 0 0 0 0 0 0 0 1 0 0 0 ;
Estimate "Wfikkn2 Slope" Log_SumAllST_1 1Log_SumAllST_1*Protein 0 0 0 0 0 0 0 0 0 0 0 0 0 0 0 0 0 0 0 0 0 0 0 0 0 0 0 0 0 0 0 0 0
0 0 0 0 0 0 0 0 0 0 0 0 0 0 0 0 0 0 0 0 0 0 0 0 0 0 0 0 0 0 0 0 0 0 0 0 0 0 0 0 0 0 0 0 0 0 0 0 0 0 0 0 0 0 0 0 1 0 0 ;
Estimate "Wisp1 Slope" Log_SumAllST_1 1Log_SumAllST_1*Protein 0 0 0 0 0 0 0 0 0 0 0 0 0 0 0 0 0 0 0 0 0 0 0 0 0 0 0 0 0 0 0 0 0 0
0 0 0 0 0 0 0 0 0 0 0 0 0 0 0 0 0 0 0 0 0 0 0 0 0 0 0 0 0 0 0 0 0 0 0 0 0 0 0 0 0 0 0 0 0 0 0 0 0 0 0 0 0 0 0 0 1 0 ;
Estimate "Yes1 Slope" Log_SumAllST_1 1Log_SumAllST_1*Protein 0 0 0 0 0 0 0 0 0 0 0 0 0 0 0 0 0 0 0 0 0 0 0 0 0 0 0 0 0 0 0 0 0 0
0 0 0 0 0 0 0 0 0 0 0 0 0 0 0 0 0 0 0 0 0 0 0 0 0 0 0 0 0 0 0 0 0 0 0 0 0 0 0 0 0 0 0 0 0 0 0 0 0 0 0 0 0 0 0 0 0 1 ;

RUN;
```

Stereotypy and Proteomics validation

```
DATA CD1_Olink_And_ST_20241125;
INPUT  SubjectID &$ Log_SUMMED_ST_1 Protein &$14. Norm_NPX; Lines;
StEt 31  4.02535169073515  norm_Clmp  3.21721
StEt 31  4.02535169073515  norm_Matn2  1.21811
StEt 31  4.02535169073515  norm_Cpe  2.12441
StEt 31  4.02535169073515  norm_Gcg  0.43969
StEt 31  4.02535169073515  norm_Gdnf  -3.07672
StEt 31  4.02535169073515  norm_Yes1  -2.36307
StEt 31  4.02535169073515  norm_Il17a  -1.47522
StEt 31  4.02535169073515  norm_Foxo1  -2.73765
StEt 31  4.02535169073515  norm_Tnfrsf11b  0.19586
StEt 31  4.02535169073515  norm_Tgfb1  -0.60879
StEt 31  4.02535169073515  norm_Pla2g4a  1.08045
StEt 31  4.02535169073515  norm_Il6  -3.96023
StEt 31  4.02535169073515  norm_Prdx5  -0.81922
StEt 31  4.02535169073515  norm_Tgfa  1.49208
StEt 31  4.02535169073515  norm_Ccl5  -2.69185
StEt 31  4.02535169073515  norm_Epo  -0.97149
StEt 31  4.02535169073515  norm_Axin1  -1.99323
StEt 31  4.02535169073515  norm_Fst  4.98576
StEt 31  4.02535169073515  norm_Rgma  -0.68318
StEt 31  4.02535169073515  norm_Nadk  1.78365
StEt 31  4.02535169073515  norm_Tnni3  10.14307
StEt 31  4.02535169073515  norm_Notch3  1.83393
StEt 31  4.02535169073515  norm_Snap29  1.69664
StEt 31  4.02535169073515  norm_Cntn1  5.62223
StEt 31  4.02535169073515  norm_Clstn2  -0.86102
StEt 31  4.02535169073515  norm_S100a4  6.27646
StEt 31  4.02535169073515  norm_Ca13  -2.89974
StEt 31  4.02535169073515  norm_Mia  1.03037
StEt 31  4.02535169073515  norm_Cant1  -1.68596
StEt 31  4.02535169073515  norm_Kitlg  -4.57816
StEt 31  4.02535169073515  norm_Gfra1  -1.03335
StEt 31  4.02535169073515  norm_Ppp1r2  -2.59998
StEt 31  4.02535169073515  norm_Adam23  -0.98226
StEt 31  4.02535169073515  norm_Itgb6  -2.93954
StEt 31  4.02535169073515  norm_Cyr61  2.96316
StEt 31  4.02535169073515  norm_Dlk1  1.09427
StEt 31  4.02535169073515  norm_Ahr  -3.35277
StEt 31  4.02535169073515  norm_Ccl2  4.27336
StEt 31  4.02535169073515  norm_Eno2  -0.36351
StEt 31  4.02535169073515  norm_Plin1  -1.28024
StEt 31  4.02535169073515  norm_Wfikkn2  1.81435
StEt 31  4.02535169073515  norm_Flrt2  0.1002
StEt 31  4.02535169073515  norm_Qdpr  -1.06343
StEt 31  4.02535169073515  norm_Fas  0.07243
StEt 31  4.02535169073515  norm_Erbb4  -0.59897
StEt 31  4.02535169073515  norm_Riox2  -2.34132
StEt 31  4.02535169073515  norm_Plxna4  0.20674
StEt 31  4.02535169073515  norm_Epcam  -1.5752
StEt 31  4.02535169073515  norm_Ccl3  -1.78423
StEt 31  4.02535169073515  norm_Crim1  -1.73428
StEt 31  4.02535169073515  norm_Vsig2  1.0292
StEt 31  4.02535169073515  norm_Hgf  0.22856
StEt 31  4.02535169073515  norm_Sez6l2  3.03447
StEt 31  4.02535169073515  norm_Il1a  -1.25918
StEt 31  4.02535169073515  norm_Il23r  2.89997
StEt 31  4.02535169073515  norm_Dll1  0.77631
StEt 31  4.02535169073515  norm_Ddah1  -3.46932
StEt 31  4.02535169073515  norm_Il10  -2.85645
StEt 31  4.02535169073515  norm_Tnfrsf12a  2.20269
StEt 31  4.02535169073515  norm_Acvrl1  0.33915
StEt 31  4.02535169073515  norm_Lgmn  1.19901
StEt 31  4.02535169073515  norm_Csf2  -2.61407
StEt 31  4.02535169073515  norm_Cxcl9  3.43426
StEt 31  4.02535169073515  norm_Map2k6  0.36238
StEt 31  4.02535169073515  norm_Itgb1bp2  -4.79564
StEt 31  4.02535169073515  norm_Il17f  -5.04095
StEt 31  4.02535169073515  norm_Il1b  -3.37095
StEt 31  4.02535169073515  norm_Casp3  3.30911
StEt 31  4.02535169073515  norm_Apbb1ip  -3.50435
StEt 31  4.02535169073515  norm_Wisp1  0.64171
StEt 31  4.02535169073515  norm_Cdh6  1.24557
StEt 31  4.02535169073515  norm_Pdgfb  4.24596
StEt 31  4.02535169073515  norm_Igsf3  -0.39024
StEt 31  4.02535169073515  norm_Tgfbr3  0.32187
StEt 31  4.02535169073515  norm_Cxcl1  2.80927
StEt 31  4.02535169073515  norm_Pak4  -3.29091
StEt 31  4.02535169073515  norm_Cntn4  2.041
StEt 31  4.02535169073515  norm_Ghrl  3.07299
StEt 31  4.02535169073515  norm_Lpl  0.58292
StEt 31  4.02535169073515  norm_Fstl3  1.7804
StEt 31  4.02535169073515  norm_Dctn2  -1.48605
StEt 31  4.02535169073515  norm_Il5  -4.10359
StEt 31  4.02535169073515  norm_Eda2r  0.3896
StEt 31  4.02535169073515  norm_Ntf3  -0.93941
StEt 31  4.02535169073515  norm_Tnfsf12  0.55398
StEt 31  4.02535169073515  norm_Ccl20  4.52961
StEt 31  4.02535169073515  norm_Fli1  -4.63949
```

StEt 31 4.02535169073515 norm_Tpp1 3.71277
StEt 31 4.02535169073515 norm_Tnr -0.37619
StEt 31 4.02535169073515 norm_Vegfd -0.66731
StEt 31 4.02535169073515 norm_Parp1 0.47614
StEt 31 4.02535169073515 norm_Tnf -3.02428
Stet 32 1.94591014905531 norm_Clmp 2.28791
Stet 32 1.94591014905531 norm_Matn2 0.96473
Stet 32 1.94591014905531 norm_Cpe 1.59378
Stet 32 1.94591014905531 norm_Gcg -0.86992
Stet 32 1.94591014905531 norm_Gdnf -3.9278
Stet 32 1.94591014905531 norm_Yes1 -2.17915
Stet 32 1.94591014905531 norm_Il17a -1.28021
Stet 32 1.94591014905531 norm_Foxo1 -2.21504
Stet 32 1.94591014905531 norm_Tnfrsf11b 0.60957
Stet 32 1.94591014905531 norm_Tgfb1 -0.17071
Stet 32 1.94591014905531 norm_Pla2g4a 2.22074
Stet 32 1.94591014905531 norm_Il6 -4.08259
Stet 32 1.94591014905531 norm_Prdx5 -0.37164
Stet 32 1.94591014905531 norm_Tgfa 1.28472
Stet 32 1.94591014905531 norm_Ccl5 -1.9357
Stet 32 1.94591014905531 norm_Epo -1.48852
Stet 32 1.94591014905531 norm_Axin1 -1.66501
Stet 32 1.94591014905531 norm_Fst 3.70725
Stet 32 1.94591014905531 norm_Rgma -1.19776
Stet 32 1.94591014905531 norm_Nadk 2.7981
Stet 32 1.94591014905531 norm_Tnni3 10.68586
Stet 32 1.94591014905531 norm_Notch3 1.1965
Stet 32 1.94591014905531 norm_Snap29 2.55772
Stet 32 1.94591014905531 norm_Cntn1 5.1752
Stet 32 1.94591014905531 norm_Clstn2 -0.92499
Stet 32 1.94591014905531 norm_S100a4 6.0431
Stet 32 1.94591014905531 norm_Ca13 -2.41277
Stet 32 1.94591014905531 norm_Mia 0.58405
Stet 32 1.94591014905531 norm_Cant1 -1.85019
Stet 32 1.94591014905531 norm_Kitlg -4.8073
Stet 32 1.94591014905531 norm_Gfra1 -1.61029
Stet 32 1.94591014905531 norm_Ppp1r2 -2.25241
Stet 32 1.94591014905531 norm_Adam23 -0.99761
Stet 32 1.94591014905531 norm_Itgb6 -3.63293
Stet 32 1.94591014905531 norm_Cyr61 2.89489
Stet 32 1.94591014905531 norm_Dlk1 0.5521
Stet 32 1.94591014905531 norm_Ahr -3.32931
Stet 32 1.94591014905531 norm_Ccl2 5.08626
Stet 32 1.94591014905531 norm_Eno2 -0.44446
Stet 32 1.94591014905531 norm_Plin1 1.09498
Stet 32 1.94591014905531 norm_Wfikkn2 3.22223
Stet 32 1.94591014905531 norm_Flrt2 0.13063
Stet 32 1.94591014905531 norm_Qdpr -0.82821
Stet 32 1.94591014905531 norm_Fas 0.871
Stet 32 1.94591014905531 norm_Erbb4 -0.867
Stet 32 1.94591014905531 norm_Riox2 -0.59588
Stet 32 1.94591014905531 norm_Plxna4 0.74145
Stet 32 1.94591014905531 norm_Epcam -2.50871
Stet 32 1.94591014905531 norm_Ccl3 -1.14924
Stet 32 1.94591014905531 norm_Crim1 -1.15566
Stet 32 1.94591014905531 norm_Vsig2 1.67432
Stet 32 1.94591014905531 norm_Hgf 1.25677
Stet 32 1.94591014905531 norm_Sez6l2 2.7226
Stet 32 1.94591014905531 norm_Il1a -1.24585
Stet 32 1.94591014905531 norm_Il23r 1.78537
Stet 32 1.94591014905531 norm_Dll1 0.71624
Stet 32 1.94591014905531 norm_Ddah1 -3.89398
Stet 32 1.94591014905531 norm_Il10 -1.86914
Stet 32 1.94591014905531 norm_Tnfrsf12a 1.62186
Stet 32 1.94591014905531 norm_Acvrl1 -0.20228
Stet 32 1.94591014905531 norm_Lgmn 1.24398
Stet 32 1.94591014905531 norm_Csf2 -2.79814
Stet 32 1.94591014905531 norm_Cxcl9 4.98173
Stet 32 1.94591014905531 norm_Map2k6 1.00398
Stet 32 1.94591014905531 norm_Itgb1bp2 -5.12658
Stet 32 1.94591014905531 norm_Il17f -5.33398
Stet 32 1.94591014905531 norm_Il1b -3.88818
Stet 32 1.94591014905531 norm_Casp3 3.43298
Stet 32 1.94591014905531 norm_Apbb1ip -3.58264
Stet 32 1.94591014905531 norm_Wisp1 0.22515
Stet 32 1.94591014905531 norm_Cdh6 0.65607
Stet 32 1.94591014905531 norm_Pdgfb 5.85888
Stet 32 1.94591014905531 norm_Igsf3 -0.06016
Stet 32 1.94591014905531 norm_Tgfbr3 -0.17635
Stet 32 1.94591014905531 norm_Cxcl1 3.02917
Stet 32 1.94591014905531 norm_Pak4 -3.2144
Stet 32 1.94591014905531 norm_Cntn4 2.16279
Stet 32 1.94591014905531 norm_Ghrl 2.35693
Stet 32 1.94591014905531 norm_Lpl -0.2802
Stet 32 1.94591014905531 norm_Fstl3 1.38075
Stet 32 1.94591014905531 norm_Dctn2 -2.58066
Stet 32 1.94591014905531 norm_Il5 -3.9703
Stet 32 1.94591014905531 norm_Eda2r -0.30326
Stet 32 1.94591014905531 norm_Ntf3 -1.83198
Stet 32 1.94591014905531 norm_Tnfsf12 0.89725
Stet 32 1.94591014905531 norm_Ccl20 3.78679
Stet 32 1.94591014905531 norm_Fli1 -4.83171
Stet 32 1.94591014905531 norm_Tpp1 3.29132
Stet 32 1.94591014905531 norm_Tnr -1.24178
Stet 32 1.94591014905531 norm_Vegfd -0.97682

Stet 32 1.94591014905531 norm_Parp1 0.99229
Stet 32 1.94591014905531 norm_Tnf -3.22019
StEt 33 3.43398720448515 norm_Clmp 2.74681
StEt 33 3.43398720448515 norm_Matn2 1.1625
StEt 33 3.43398720448515 norm_Cpe 2.06252
StEt 33 3.43398720448515 norm_Gcg 0.38794
StEt 33 3.43398720448515 norm_Gdnf -3.58646
StEt 33 3.43398720448515 norm_Yes1 -2.30935
StEt 33 3.43398720448515 norm_Il17a -1.7741
StEt 33 3.43398720448515 norm_Foxo1 -2.1895
StEt 33 3.43398720448515 norm_Tnfrsf11b 0.3681
StEt 33 3.43398720448515 norm_Tgfb1 0.27465
StEt 33 3.43398720448515 norm_Pla2g4a 1.32783
StEt 33 3.43398720448515 norm_Il6 -4.27174
StEt 33 3.43398720448515 norm_Prdx5 -1.20841
StEt 33 3.43398720448515 norm_Tgfa 1.09283
StEt 33 3.43398720448515 norm_Ccl5 -2.77414
StEt 33 3.43398720448515 norm_Epo -1.42756
StEt 33 3.43398720448515 norm_Axin1 -1.34294
StEt 33 3.43398720448515 norm_Fst 5.01267
StEt 33 3.43398720448515 norm_Rgma -1.24287
StEt 33 3.43398720448515 norm_Nadk 3.11935
StEt 33 3.43398720448515 norm_Tnni3 9.47532
StEt 33 3.43398720448515 norm_Notch3 1.44561
StEt 33 3.43398720448515 norm_Snap29 2.42372
StEt 33 3.43398720448515 norm_Cntn1 5.49869
StEt 33 3.43398720448515 norm_Clstn2 -1.51973
StEt 33 3.43398720448515 norm_S100a4 6.29096
StEt 33 3.43398720448515 norm_Ca13 -2.36822
StEt 33 3.43398720448515 norm_Mia 0.32301
StEt 33 3.43398720448515 norm_Cant1 -1.38434
StEt 33 3.43398720448515 norm_Kitlg -4.35921
StEt 33 3.43398720448515 norm_Gfra1 -1.14096
StEt 33 3.43398720448515 norm_Ppp1r2 -2.84142
StEt 33 3.43398720448515 norm_Adam23 -1.52997
StEt 33 3.43398720448515 norm_Itgb6 -3.07256
StEt 33 3.43398720448515 norm_Cyr61 3.21916
StEt 33 3.43398720448515 norm_Dlk1 0.30647
StEt 33 3.43398720448515 norm_Ahr -3.04536
StEt 33 3.43398720448515 norm_Ccl2 3.95179
StEt 33 3.43398720448515 norm_Eno2 -0.00281
StEt 33 3.43398720448515 norm_Plin1 0.83559
StEt 33 3.43398720448515 norm_Wfikkn2 1.25521
StEt 33 3.43398720448515 norm_Flrt2 0.09472
StEt 33 3.43398720448515 norm_Qdpr -1.14863
StEt 33 3.43398720448515 norm_Fas 0.71244
StEt 33 3.43398720448515 norm_Erbb4 -0.26865
StEt 33 3.43398720448515 norm_Riox2 -1.33543
StEt 33 3.43398720448515 norm_Plxna4 0.33931
StEt 33 3.43398720448515 norm_Epcam -1.66746
StEt 33 3.43398720448515 norm_Ccl3 -2.11643
StEt 33 3.43398720448515 norm_Crim1 -1.18707
StEt 33 3.43398720448515 norm_Vsig2 1.75746
StEt 33 3.43398720448515 norm_Hgf 1.7483
StEt 33 3.43398720448515 norm_Sez6l2 2.67227
StEt 33 3.43398720448515 norm_Il1a -1.52933
StEt 33 3.43398720448515 norm_Il23r 3.39082
StEt 33 3.43398720448515 norm_Dll1 0.39547
StEt 33 3.43398720448515 norm_Ddah1 -4.09713
StEt 33 3.43398720448515 norm_Il10 -3.1038
StEt 33 3.43398720448515 norm_Tnfrsf12a 1.97058
StEt 33 3.43398720448515 norm_Acvrl1 -0.10017
StEt 33 3.43398720448515 norm_Lgmn 1.18321
StEt 33 3.43398720448515 norm_Csf2 -2.60044
StEt 33 3.43398720448515 norm_Cxcl9 2.16285
StEt 33 3.43398720448515 norm_Map2k6 0.70028
StEt 33 3.43398720448515 norm_Itgb1bp2 -4.40038
StEt 33 3.43398720448515 norm_Il17f -5.04257
StEt 33 3.43398720448515 norm_Il1b -3.80211
StEt 33 3.43398720448515 norm_Casp3 3.77988
StEt 33 3.43398720448515 norm_Apbb1ip -3.17798
StEt 33 3.43398720448515 norm_Wisp1 0.24899
StEt 33 3.43398720448515 norm_Cdh6 0.42413
StEt 33 3.43398720448515 norm_Pdgfb 6.1104
StEt 33 3.43398720448515 norm_Igsf3 -0.26639
StEt 33 3.43398720448515 norm_Tgfbr3 0.5344
StEt 33 3.43398720448515 norm_Cxcl1 2.6425
StEt 33 3.43398720448515 norm_Pak4 -3.77005
StEt 33 3.43398720448515 norm_Cntn4 2.68953
StEt 33 3.43398720448515 norm_Ghrl 2.4815
StEt 33 3.43398720448515 norm_Lpl -0.13553
StEt 33 3.43398720448515 norm_Fstl3 1.28068
StEt 33 3.43398720448515 norm_Dctn2 -2.03884
StEt 33 3.43398720448515 norm_Il5 -3.85726
StEt 33 3.43398720448515 norm_Eda2r -0.04196
StEt 33 3.43398720448515 norm_Ntf3 -1.99465
StEt 33 3.43398720448515 norm_Tnfsf12 0.33534
StEt 33 3.43398720448515 norm_Ccl20 4.62367
StEt 33 3.43398720448515 norm_Fli1 -3.95835
StEt 33 3.43398720448515 norm_Tpp1 3.66806
StEt 33 3.43398720448515 norm_Tnr -1.08801
StEt 33 3.43398720448515 norm_Vegfd -0.70051
StEt 33 3.43398720448515 norm_Parp1 1.59771
StEt 33 3.43398720448515 norm_Tnf -3.30426
StEt 34 2.56494935746154 norm_Clmp 2.31799

StEt 34 2.56494935746154 norm_Matn2 0.93476
StEt 34 2.56494935746154 norm_Cpe 1.52737
StEt 34 2.56494935746154 norm_Gcg -1.39386
StEt 34 2.56494935746154 norm_Gdnf -3.46874
StEt 34 2.56494935746154 norm_Yes1 -1.56987
StEt 34 2.56494935746154 norm_Il17a -0.94379
StEt 34 2.56494935746154 norm_Foxo1 -2.39181
StEt 34 2.56494935746154 norm_Tnfrsf11b 0.37935
StEt 34 2.56494935746154 norm_Tgfb1 0.69615
StEt 34 2.56494935746154 norm_Pla2g4a 2.06097
StEt 34 2.56494935746154 norm_Il6 -4.00395
StEt 34 2.56494935746154 norm_Prdx5 0.68833
StEt 34 2.56494935746154 norm_Tgfa 1.22494
StEt 34 2.56494935746154 norm_Ccl5 -2.24328
StEt 34 2.56494935746154 norm_Epo -2.18093
StEt 34 2.56494935746154 norm_Axin1 -1.60136
StEt 34 2.56494935746154 norm_Fst 4.72551
StEt 34 2.56494935746154 norm_Rgma -1.38189
StEt 34 2.56494935746154 norm_Nadk 2.84933
StEt 34 2.56494935746154 norm_Tnni3 9.40109
StEt 34 2.56494935746154 norm_Notch3 1.80464
StEt 34 2.56494935746154 norm_Snap29 2.73942
StEt 34 2.56494935746154 norm_Cntn1 5.32252
StEt 34 2.56494935746154 norm_Clstn2 -1.93821
StEt 34 2.56494935746154 norm_S100a4 6.09077
StEt 34 2.56494935746154 norm_Ca13 -2.35957
StEt 34 2.56494935746154 norm_Mia 0.11026
StEt 34 2.56494935746154 norm_Cant1 -1.5184
StEt 34 2.56494935746154 norm_Kitlg -4.73147
StEt 34 2.56494935746154 norm_Gfra1 -1.06301
StEt 34 2.56494935746154 norm_Ppp1r2 -1.59217
StEt 34 2.56494935746154 norm_Adam23 -1.03552
StEt 34 2.56494935746154 norm_Itgb6 -3.10591
StEt 34 2.56494935746154 norm_Cyr61 3.41517
StEt 34 2.56494935746154 norm_Dlk1 0.74464
StEt 34 2.56494935746154 norm_Ahr -3.21865
StEt 34 2.56494935746154 norm_Ccl2 3.82876
StEt 34 2.56494935746154 norm_Eno2 -0.49324
StEt 34 2.56494935746154 norm_Plin1 0.49098
StEt 34 2.56494935746154 norm_Wfikkn2 1.44841
StEt 34 2.56494935746154 norm_Flrt2 -0.18837
StEt 34 2.56494935746154 norm_Qdpr -0.51087
StEt 34 2.56494935746154 norm_Fas 1.02348
StEt 34 2.56494935746154 norm_Erbb4 -0.80776
StEt 34 2.56494935746154 norm_Riox2 -0.05251
StEt 34 2.56494935746154 norm_Plxna4 -0.07339
StEt 34 2.56494935746154 norm_Epcam -2.34098
StEt 34 2.56494935746154 norm_Ccl3 -1.59207
StEt 34 2.56494935746154 norm_Crim1 -0.27622
StEt 34 2.56494935746154 norm_Vsig2 0.63398
StEt 34 2.56494935746154 norm_Hgf 2.93555
StEt 34 2.56494935746154 norm_Sez6l2 2.67598
StEt 34 2.56494935746154 norm_Il1a -0.16269
StEt 34 2.56494935746154 norm_Il23r 4.13265
StEt 34 2.56494935746154 norm_Dll1 0.56521
StEt 34 2.56494935746154 norm_Ddah1 -3.82473
StEt 34 2.56494935746154 norm_Il10 -3.64672
StEt 34 2.56494935746154 norm_Tnfrsf12a 1.83754
StEt 34 2.56494935746154 norm_Acvrl1 -0.01536
StEt 34 2.56494935746154 norm_Lgmn 1.00007
StEt 34 2.56494935746154 norm_Csf2 -2.94684
StEt 34 2.56494935746154 norm_Cxcl9 2.69362
StEt 34 2.56494935746154 norm_Map2k6 1.92309
StEt 34 2.56494935746154 norm_Itgb1bp2 -5.06257
StEt 34 2.56494935746154 norm_Il17f -5.22167
StEt 34 2.56494935746154 norm_Il1b -3.76579
StEt 34 2.56494935746154 norm_Casp3 3.91926
StEt 34 2.56494935746154 norm_Apbb1ip -3.42711
StEt 34 2.56494935746154 norm_Wisp1 0.14494
StEt 34 2.56494935746154 norm_Cdh6 0.56126
StEt 34 2.56494935746154 norm_Pdgfb 6.35201
StEt 34 2.56494935746154 norm_Igsf3 -0.26642
StEt 34 2.56494935746154 norm_Tgfbr3 0.03677
StEt 34 2.56494935746154 norm_Cxcl1 3.19956
StEt 34 2.56494935746154 norm_Pak4 -3.68713
StEt 34 2.56494935746154 norm_Cntn4 -0.3466
StEt 34 2.56494935746154 norm_Ghrl 0.83549
StEt 34 2.56494935746154 norm_Lpl 0.07297
StEt 34 2.56494935746154 norm_Fstl3 1.18918
StEt 34 2.56494935746154 norm_Dctn2 -1.24453
StEt 34 2.56494935746154 norm_Il5 -4.10358
StEt 34 2.56494935746154 norm_Eda2r -0.56467
StEt 34 2.56494935746154 norm_Ntf3 -2.28101
StEt 34 2.56494935746154 norm_Tnfsf12 1.02817
StEt 34 2.56494935746154 norm_Ccl20 4.48173
StEt 34 2.56494935746154 norm_Fli1 -4.55855
StEt 34 2.56494935746154 norm_Tpp1 3.21547
StEt 34 2.56494935746154 norm_Tnr -1.00756
StEt 34 2.56494935746154 norm_Vegfd -0.89272
StEt 34 2.56494935746154 norm_Parp1 1.40912
StEt 34 2.56494935746154 norm_Tnf -3.56471
StEt 35 4.54329478227 norm_Clmp 2.91669
StEt 35 4.54329478227 norm_Matn2 1.7547
StEt 35 4.54329478227 norm_Cpe 2.30924
StEt 35 4.54329478227 norm_Gcg -0.32161

StEt 35 4.54329478227 norm_Gdnf -3.1679
StEt 35 4.54329478227 norm_Yes1 -2.40225
StEt 35 4.54329478227 norm_Il17a -1.11707
StEt 35 4.54329478227 norm_Foxo1 -2.82696
StEt 35 4.54329478227 norm_Tnfrsf11b 0.71739
StEt 35 4.54329478227 norm_Tgfb1 0.02059
StEt 35 4.54329478227 norm_Pla2g4a 1.03224
StEt 35 4.54329478227 norm_Il6 -3.71039
StEt 35 4.54329478227 norm_Prdx5 -1.16576
StEt 35 4.54329478227 norm_Tgfa 1.37016
StEt 35 4.54329478227 norm_Ccl5 -2.38458
StEt 35 4.54329478227 norm_Epo -1.84161
StEt 35 4.54329478227 norm_Axin1 -1.87681
StEt 35 4.54329478227 norm_Fst 5.0016
StEt 35 4.54329478227 norm_Rgma -0.70351
StEt 35 4.54329478227 norm_Nadk 2.23583
StEt 35 4.54329478227 norm_Tnni3 8.81867
StEt 35 4.54329478227 norm_Notch3 1.57168
StEt 35 4.54329478227 norm_Snap29 1.87426
StEt 35 4.54329478227 norm_Cntn1 5.16596
StEt 35 4.54329478227 norm_Clstn2 -1.99499
StEt 35 4.54329478227 norm_S100a4 6.38522
StEt 35 4.54329478227 norm_Ca13 -2.50827
StEt 35 4.54329478227 norm_Mia 0.63634
StEt 35 4.54329478227 norm_Cant1 -1.40294
StEt 35 4.54329478227 norm_Kitlg -3.19136
StEt 35 4.54329478227 norm_Gfra1 -1.16795
StEt 35 4.54329478227 norm_Ppp1r2 -2.57467
StEt 35 4.54329478227 norm_Adam23 -1.37141
StEt 35 4.54329478227 norm_Itgb6 -2.83238
StEt 35 4.54329478227 norm_Cyr61 3.24548
StEt 35 4.54329478227 norm_Dlk1 0.90338
StEt 35 4.54329478227 norm_Ahr -2.68993
StEt 35 4.54329478227 norm_Ccl2 4.05844
StEt 35 4.54329478227 norm_Eno2 -0.21026
StEt 35 4.54329478227 norm_Plin1 0.29037
StEt 35 4.54329478227 norm_Wfikkn2 1.46078
StEt 35 4.54329478227 norm_Flrt2 -0.00152
StEt 35 4.54329478227 norm_Qdpr -1.12237
StEt 35 4.54329478227 norm_Fas 0.69383
StEt 35 4.54329478227 norm_Erbb4 -0.66362
StEt 35 4.54329478227 norm_Riox2 -1.12074
StEt 35 4.54329478227 norm_Plxna4 0.20897
StEt 35 4.54329478227 norm_Epcam -1.21829
StEt 35 4.54329478227 norm_Ccl3 -1.88325
StEt 35 4.54329478227 norm_Crim1 -1.44831
StEt 35 4.54329478227 norm_Vsig2 -0.35257
StEt 35 4.54329478227 norm_Hgf 0.82302
StEt 35 4.54329478227 norm_Sez6l2 2.58358
StEt 35 4.54329478227 norm_Il1a -1.66064
StEt 35 4.54329478227 norm_Il23r 4.86424
StEt 35 4.54329478227 norm_Dll1 0.72236
StEt 35 4.54329478227 norm_Ddah1 -3.58293
StEt 35 4.54329478227 norm_Il10 -3.33264
StEt 35 4.54329478227 norm_Tnfrsf12a 1.6758
StEt 35 4.54329478227 norm_Acvrl1 -0.09059
StEt 35 4.54329478227 norm_Lgmn 1.21201
StEt 35 4.54329478227 norm_Csf2 -2.37153
StEt 35 4.54329478227 norm_Cxcl9 2.61396
StEt 35 4.54329478227 norm_Map2k6 0.63682
StEt 35 4.54329478227 norm_Itgb1bp2 -4.58738
StEt 35 4.54329478227 norm_Il17f -4.52633
StEt 35 4.54329478227 norm_Il1b -3.21165
StEt 35 4.54329478227 norm_Casp3 3.33194
StEt 35 4.54329478227 norm_Apbb1ip -3.44473
StEt 35 4.54329478227 norm_Wisp1 0.41723
StEt 35 4.54329478227 norm_Cdh6 0.73227
StEt 35 4.54329478227 norm_Pdgfb 5.11168
StEt 35 4.54329478227 norm_Igsf3 -0.46557
StEt 35 4.54329478227 norm_Tgfbr3 0.00639
StEt 35 4.54329478227 norm_Cxcl1 2.78675
StEt 35 4.54329478227 norm_Pak4 -3.22405
StEt 35 4.54329478227 norm_Cntn4 2.37645
StEt 35 4.54329478227 norm_Ghrl 2.75098
StEt 35 4.54329478227 norm_Lpl 0.21963
StEt 35 4.54329478227 norm_Fstl3 1.07297
StEt 35 4.54329478227 norm_Dctn2 -2.47503
StEt 35 4.54329478227 norm_Il5 -3.17539
StEt 35 4.54329478227 norm_Eda2r 0.35328
StEt 35 4.54329478227 norm_Ntf3 -1.35441
StEt 35 4.54329478227 norm_Tnfsf12 0.3049
StEt 35 4.54329478227 norm_Ccl20 4.74036
StEt 35 4.54329478227 norm_Fli1 -4.44774
StEt 35 4.54329478227 norm_Tpp1 3.65204
StEt 35 4.54329478227 norm_Tnr -0.69379
StEt 35 4.54329478227 norm_Vegfd -0.81075
StEt 35 4.54329478227 norm_Parp1 -0.10128
StEt 35 4.54329478227 norm_Tnf -2.83036
StEt 36 1.6094379124341 norm_Clmp 2.24798
StEt 36 1.6094379124341 norm_Matn2 1.58388
StEt 36 1.6094379124341 norm_Cpe 2.41762
StEt 36 1.6094379124341 norm_Gcg -1.0624
StEt 36 1.6094379124341 norm_Gdnf -3.84894
StEt 36 1.6094379124341 norm_Yes1 -2.14774
StEt 36 1.6094379124341 norm_Il17a -1.90641

StEt 36 1.6094379124341 norm_Foxo1 -1.76097
StEt 36 1.6094379124341 norm_Tnfrsf11b 0.99458
StEt 36 1.6094379124341 norm_Tgfb1 -0.25672
StEt 36 1.6094379124341 norm_Pla2g4a 1.65742
StEt 36 1.6094379124341 norm_Il6 -3.72068
StEt 36 1.6094379124341 norm_Prdx5 -1.96383
StEt 36 1.6094379124341 norm_Tgfa 1.25546
StEt 36 1.6094379124341 norm_Ccl5 -2.30355
StEt 36 1.6094379124341 norm_Epo -2.32888
StEt 36 1.6094379124341 norm_Axin1 -1.7265
StEt 36 1.6094379124341 norm_Fst 5.15598
StEt 36 1.6094379124341 norm_Rgma -1.16157
StEt 36 1.6094379124341 norm_Nadk 2.56511
StEt 36 1.6094379124341 norm_Tnni3 8.4479
StEt 36 1.6094379124341 norm_Notch3 1.89719
StEt 36 1.6094379124341 norm_Snap29 2.21457
StEt 36 1.6094379124341 norm_Cntn1 5.56556
StEt 36 1.6094379124341 norm_Clstn2 -1.67096
StEt 36 1.6094379124341 norm_S100a4 6.24998
StEt 36 1.6094379124341 norm_Ca13 -2.73565
StEt 36 1.6094379124341 norm_Mia 0.44661
StEt 36 1.6094379124341 norm_Cant1 -1.3204
StEt 36 1.6094379124341 norm_Kitlg -4.26487
StEt 36 1.6094379124341 norm_Gfra1 -0.49501
StEt 36 1.6094379124341 norm_Ppp1r2 -2.47266
StEt 36 1.6094379124341 norm_Adam23 -1.13436
StEt 36 1.6094379124341 norm_Itgb6 -2.65335
StEt 36 1.6094379124341 norm_Cyr61 2.4618
StEt 36 1.6094379124341 norm_Dlk1 0.68444
StEt 36 1.6094379124341 norm_Ahr -2.87437
StEt 36 1.6094379124341 norm_Ccl2 3.92634
StEt 36 1.6094379124341 norm_Eno2 -0.53241
StEt 36 1.6094379124341 norm_Plin1 -1.53166
StEt 36 1.6094379124341 norm_Wfikkn2 2.25346
StEt 36 1.6094379124341 norm_Flrt2 0.35844
StEt 36 1.6094379124341 norm_Qdpr -1.0727
StEt 36 1.6094379124341 norm_Fas 0.99536
StEt 36 1.6094379124341 norm_Erbb4 -0.46059
StEt 36 1.6094379124341 norm_Riox2 -2.08683
StEt 36 1.6094379124341 norm_Plxna4 0.8429
StEt 36 1.6094379124341 norm_Epcam -2.24649
StEt 36 1.6094379124341 norm_Ccl3 -2.07994
StEt 36 1.6094379124341 norm_Crim1 -1.32103
StEt 36 1.6094379124341 norm_Vsig2 1.22543
StEt 36 1.6094379124341 norm_Hgf 0.1053
StEt 36 1.6094379124341 norm_Sez6l2 2.72445
StEt 36 1.6094379124341 norm_Il1a -0.59321
StEt 36 1.6094379124341 norm_Il23r 4.21515
StEt 36 1.6094379124341 norm_Dll1 0.68618
StEt 36 1.6094379124341 norm_Ddah1 -3.90364
StEt 36 1.6094379124341 norm_Il10 -2.42978
StEt 36 1.6094379124341 norm_Tnfrsf12a 1.34304
StEt 36 1.6094379124341 norm_Acvrl1 -0.14516
StEt 36 1.6094379124341 norm_Lgmn 1.56674
StEt 36 1.6094379124341 norm_Csf2 -2.63824
StEt 36 1.6094379124341 norm_Cxcl9 3.5032
StEt 36 1.6094379124341 norm_Map2k6 0.52193
StEt 36 1.6094379124341 norm_Itgb1bp2 -3.79175
StEt 36 1.6094379124341 norm_Il17f -4.51507
StEt 36 1.6094379124341 norm_Il1b -3.23817
StEt 36 1.6094379124341 norm_Casp3 3.37688
StEt 36 1.6094379124341 norm_Apbb1ip -3.5419
StEt 36 1.6094379124341 norm_Wisp1 0.59838
StEt 36 1.6094379124341 norm_Cdh6 0.97263
StEt 36 1.6094379124341 norm_Pdgfb 3.87643
StEt 36 1.6094379124341 norm_Igsf3 -0.11552
StEt 36 1.6094379124341 norm_Tgfbr3 0.1795
StEt 36 1.6094379124341 norm_Cxcl1 2.57353
StEt 36 1.6094379124341 norm_Pak4 -2.99753
StEt 36 1.6094379124341 norm_Cntn4 3.11281
StEt 36 1.6094379124341 norm_Ghrl 2.67232
StEt 36 1.6094379124341 norm_Lpl 0.82683
StEt 36 1.6094379124341 norm_Fstl3 1.19323
StEt 36 1.6094379124341 norm_Dctn2 -2.19999
StEt 36 1.6094379124341 norm_Il5 -3.32246
StEt 36 1.6094379124341 norm_Eda2r -0.12801
StEt 36 1.6094379124341 norm_Ntf3 -1.51755
StEt 36 1.6094379124341 norm_Tnfsf12 0.74492
StEt 36 1.6094379124341 norm_Ccl20 4.50581
StEt 36 1.6094379124341 norm_Fli1 -4.12631
StEt 36 1.6094379124341 norm_Tpp1 3.73533
StEt 36 1.6094379124341 norm_Tnr -0.79681
StEt 36 1.6094379124341 norm_Vegfd -0.54669
StEt 36 1.6094379124341 norm_Parp1 0.05395
StEt 36 1.6094379124341 norm_Tnf -2.84694
StEt 37 4.36944785246702 norm_Clmp 2.41074
StEt 37 4.36944785246702 norm_Matn2 1.14145
StEt 37 4.36944785246702 norm_Cpe 1.88248
StEt 37 4.36944785246702 norm_Gcg -1.89783
StEt 37 4.36944785246702 norm_Gdnf -3.53506
StEt 37 4.36944785246702 norm_Yes1 -1.00463
StEt 37 4.36944785246702 norm_Il17a -0.12786
StEt 37 4.36944785246702 norm_Foxo1 -2.16496
StEt 37 4.36944785246702 norm_Tnfrsf11b 0.1869
StEt 37 4.36944785246702 norm_Tgfb1 0.42947

StEt 37	4.36944785246702	norm_Pla2g4a	1.91722
StEt 37	4.36944785246702	norm_Il6	-4.52546
StEt 37	4.36944785246702	norm_Prdx5	0.85535
StEt 37	4.36944785246702	norm_Tgfa	1.01113
StEt 37	4.36944785246702	norm_Ccl5	-2.87083
StEt 37	4.36944785246702	norm_Epo	-2.57688
StEt 37	4.36944785246702	norm_Axin1	-1.343
StEt 37	4.36944785246702	norm_Fst	5.07862
StEt 37	4.36944785246702	norm_Rgma	-1.48364
StEt 37	4.36944785246702	norm_Nadk	3.49142
StEt 37	4.36944785246702	norm_Tnni3	7.74342
StEt 37	4.36944785246702	norm_Notch3	1.41083
StEt 37	4.36944785246702	norm_Snap29	3.05802
StEt 37	4.36944785246702	norm_Cntn1	4.98384
StEt 37	4.36944785246702	norm_Clstn2	-2.43746
StEt 37	4.36944785246702	norm_S100a4	5.81682
StEt 37	4.36944785246702	norm_Ca13	-2.0923
StEt 37	4.36944785246702	norm_Mia	-0.06063
StEt 37	4.36944785246702	norm_Cant1	-1.67153
StEt 37	4.36944785246702	norm_Kitlg	-4.73015
StEt 37	4.36944785246702	norm_Gfra1	-1.02859
StEt 37	4.36944785246702	norm_Ppp1r2	-0.91886
StEt 37	4.36944785246702	norm_Adam23	-1.4523
StEt 37	4.36944785246702	norm_Itgb6	-3.87063
StEt 37	4.36944785246702	norm_Cyr61	2.7219
StEt 37	4.36944785246702	norm_Dlk1	0.69826
StEt 37	4.36944785246702	norm_Ahr	-3.23102
StEt 37	4.36944785246702	norm_Ccl2	6.71152
StEt 37	4.36944785246702	norm_Eno2	-0.34136
StEt 37	4.36944785246702	norm_Plin1	-0.32476
StEt 37	4.36944785246702	norm_Wfikkn2	1.20035
StEt 37	4.36944785246702	norm_Flrt2	0.09548
StEt 37	4.36944785246702	norm_Qdpr	-0.07175
StEt 37	4.36944785246702	norm_Fas	0.40771
StEt 37	4.36944785246702	norm_Erbb4	-1.06413
StEt 37	4.36944785246702	norm_Riox2	0.75459
StEt 37	4.36944785246702	norm_Plxna4	-0.51491
StEt 37	4.36944785246702	norm_Epcam	-1.58225
StEt 37	4.36944785246702	norm_Ccl3	-0.88877
StEt 37	4.36944785246702	norm_Crim1	0.74798
StEt 37	4.36944785246702	norm_Vsig2	1.14216
StEt 37	4.36944785246702	norm_Hgf	2.29375
StEt 37	4.36944785246702	norm_Sez6l2	1.90259
StEt 37	4.36944785246702	norm_Il1a	-0.47858
StEt 37	4.36944785246702	norm_Il23r	4.24122
StEt 37	4.36944785246702	norm_Dll1	0.49515
StEt 37	4.36944785246702	norm_Ddah1	-3.14447
StEt 37	4.36944785246702	norm_Il10	-3.21969
StEt 37	4.36944785246702	norm_Tnfrsf12a	1.35938
StEt 37	4.36944785246702	norm_Acvrl1	-0.51169
StEt 37	4.36944785246702	norm_Lgmn	1.18762
StEt 37	4.36944785246702	norm_Csf2	-3.51468
StEt 37	4.36944785246702	norm_Cxcl9	1.77238
StEt 37	4.36944785246702	norm_Map2k6	2.07017
StEt 37	4.36944785246702	norm_Itgb1bp2	-5.68681
StEt 37	4.36944785246702	norm_Il17f	-5.29631
StEt 37	4.36944785246702	norm_Il1b	-3.2266
StEt 37	4.36944785246702	norm_Casp3	3.7999
StEt 37	4.36944785246702	norm_Apbb1ip	-3.41788
StEt 37	4.36944785246702	norm_Wisp1	0.07834
StEt 37	4.36944785246702	norm_Cdh6	0.45255
StEt 37	4.36944785246702	norm_Pdgfb	5.74497
StEt 37	4.36944785246702	norm_Igsf3	-0.57989
StEt 37	4.36944785246702	norm_Tgfbr3	-0.21982
StEt 37	4.36944785246702	norm_Cxcl1	3.21825
StEt 37	4.36944785246702	norm_Pak4	-3.32806
StEt 37	4.36944785246702	norm_Cntn4	1.94188
StEt 37	4.36944785246702	norm_Ghrl	1.38095
StEt 37	4.36944785246702	norm_Lpl	0.15853
StEt 37	4.36944785246702	norm_Fstl3	0.79261
StEt 37	4.36944785246702	norm_Dctn2	-1.26281
StEt 37	4.36944785246702	norm_Il5	-4.18262
StEt 37	4.36944785246702	norm_Eda2r	-0.48365
StEt 37	4.36944785246702	norm_Ntf3	-2.41213
StEt 37	4.36944785246702	norm_Tnfsf12	0.47064
StEt 37	4.36944785246702	norm_Ccl20	5.08772
StEt 37	4.36944785246702	norm_Fli1	-3.89264
StEt 37	4.36944785246702	norm_Tpp1	2.82856
StEt 37	4.36944785246702	norm_Tnr	-1.50511
StEt 37	4.36944785246702	norm_Vegfd	-1.14363
StEt 37	4.36944785246702	norm_Parp1	1.37692
StEt 37	4.36944785246702	norm_Tnf	-3.23301
StEt 38	2.63905732961526	norm_Clmp	2.82062
StEt 38	2.63905732961526	norm_Matn2	1.41063
StEt 38	2.63905732961526	norm_Cpe	2.13409
StEt 38	2.63905732961526	norm_Gcg	-0.74388
StEt 38	2.63905732961526	norm_Gdnf	-3.82374
StEt 38	2.63905732961526	norm_Yes1	-2.25873
StEt 38	2.63905732961526	norm_Il17a	1.06621
StEt 38	2.63905732961526	norm_Foxo1	-2.53409
StEt 38	2.63905732961526	norm_Tnfrsf11b	0.95108
StEt 38	2.63905732961526	norm_Tgfb1	-0.6521
StEt 38	2.63905732961526	norm_Pla2g4a	0.24274
StEt 38	2.63905732961526	norm_Il6	-2.42829
StEt 38	2.63905732961526	norm_Prdx5	-1.56155

StEt 38	2.63905732961526	norm_Tgfa	1.06899
StEt 38	2.63905732961526	norm_Ccl5	-1.86452
StEt 38	2.63905732961526	norm_Epo	-0.26786
StEt 38	2.63905732961526	norm_Axin1	-2.12597
StEt 38	2.63905732961526	norm_Fst	4.93301
StEt 38	2.63905732961526	norm_Rgma	-1.15202
StEt 38	2.63905732961526	norm_Nadk	2.2315
StEt 38	2.63905732961526	norm_Tnni3	10.99435
StEt 38	2.63905732961526	norm_Notch3	1.89448
StEt 38	2.63905732961526	norm_Snap29	1.67017
StEt 38	2.63905732961526	norm_Cntn1	5.6224
StEt 38	2.63905732961526	norm_Clstn2	-1.06372
StEt 38	2.63905732961526	norm_S100a4	6.35186
StEt 38	2.63905732961526	norm_Ca13	-3.22465
StEt 38	2.63905732961526	norm_Mia	0.96512
StEt 38	2.63905732961526	norm_Cant1	-1.54581
StEt 38	2.63905732961526	norm_Kitlg	-4.69381
StEt 38	2.63905732961526	norm_Gfra1	-0.95279
StEt 38	2.63905732961526	norm_Ppp1r2	-2.73716
StEt 38	2.63905732961526	norm_Adam23	-0.60737
StEt 38	2.63905732961526	norm_Itgb6	-2.99758
StEt 38	2.63905732961526	norm_Cyr61	2.70597
StEt 38	2.63905732961526	norm_Dlk1	0.65549
StEt 38	2.63905732961526	norm_Ahr	-3.31681
StEt 38	2.63905732961526	norm_Ccl2	4.94729
StEt 38	2.63905732961526	norm_Eno2	-0.26707
StEt 38	2.63905732961526	norm_Plin1	-2.1719
StEt 38	2.63905732961526	norm_Wfikkn2	3.02447
StEt 38	2.63905732961526	norm_Flrt2	-0.09478
StEt 38	2.63905732961526	norm_Qdpr	-0.76011
StEt 38	2.63905732961526	norm_Fas	1.22114
StEt 38	2.63905732961526	norm_Erbb4	-0.35836
StEt 38	2.63905732961526	norm_Riox2	-1.79618
StEt 38	2.63905732961526	norm_Plxna4	0.62857
StEt 38	2.63905732961526	norm_Epcam	-1.52143
StEt 38	2.63905732961526	norm_Ccl3	-2.19128
StEt 38	2.63905732961526	norm_Crim1	-1.80061
StEt 38	2.63905732961526	norm_Vsig2	1.70672
StEt 38	2.63905732961526	norm_Hgf	-0.48766
StEt 38	2.63905732961526	norm_Sez6l2	2.96562
StEt 38	2.63905732961526	norm_Il1a	-1.67264
StEt 38	2.63905732961526	norm_Il23r	6.01346
StEt 38	2.63905732961526	norm_Dll1	1.02955
StEt 38	2.63905732961526	norm_Ddah1	-3.48434
StEt 38	2.63905732961526	norm_Il10	-2.89743
StEt 38	2.63905732961526	norm_Tnfrsf12a	2.06124
StEt 38	2.63905732961526	norm_Acvrl1	0.04707
StEt 38	2.63905732961526	norm_Lgmn	1.71658
StEt 38	2.63905732961526	norm_Csf2	-2.59638
StEt 38	2.63905732961526	norm_Cxcl9	3.37755
StEt 38	2.63905732961526	norm_Map2k6	0.05062
StEt 38	2.63905732961526	norm_Itgb1bp2	-4.64406
StEt 38	2.63905732961526	norm_Il17f	-4.50362
StEt 38	2.63905732961526	norm_Il1b	-3.21327
StEt 38	2.63905732961526	norm_Casp3	2.62668
StEt 38	2.63905732961526	norm_Apbb1ip	-3.81642
StEt 38	2.63905732961526	norm_Wisp1	1.04019
StEt 38	2.63905732961526	norm_Cdh6	1.21284
StEt 38	2.63905732961526	norm_Pdgfb	2.69557
StEt 38	2.63905732961526	norm_Igsf3	-0.46271
StEt 38	2.63905732961526	norm_Tgfbr3	0.46123
StEt 38	2.63905732961526	norm_Cxcl1	2.58503
StEt 38	2.63905732961526	norm_Pak4	-3.33568
StEt 38	2.63905732961526	norm_Cntn4	-0.35492
StEt 38	2.63905732961526	norm_Ghrl	2.32406
StEt 38	2.63905732961526	norm_Lpl	-0.31126
StEt 38	2.63905732961526	norm_Fstl3	1.64481
StEt 38	2.63905732961526	norm_Dctn2	-2.31557
StEt 38	2.63905732961526	norm_Il5	-4.29118
StEt 38	2.63905732961526	norm_Eda2r	-0.1109
StEt 38	2.63905732961526	norm_Ntf3	-1.23495
StEt 38	2.63905732961526	norm_Tnfsf12	0.54662
StEt 38	2.63905732961526	norm_Ccl20	5.03155
StEt 38	2.63905732961526	norm_Fli1	-4.90517
StEt 38	2.63905732961526	norm_Tpp1	4.07067
StEt 38	2.63905732961526	norm_Tnr	-1.05111
StEt 38	2.63905732961526	norm_Vegfd	-0.74961
StEt 38	2.63905732961526	norm_Parp1	-0.0047
StEt 38	2.63905732961526	norm_Tnf	-2.79038
StEt 39	3.85014760171006	norm_Clmp	2.71041
StEt 39	3.85014760171006	norm_Matn2	1.6427
StEt 39	3.85014760171006	norm_Cpe	2.05394
StEt 39	3.85014760171006	norm_Gcg	0.37144
StEt 39	3.85014760171006	norm_Gdnf	-3.42271
StEt 39	3.85014760171006	norm_Yes1	-1.83149
StEt 39	3.85014760171006	norm_Il17a	-2.74666
StEt 39	3.85014760171006	norm_Foxo1	-2.21303
StEt 39	3.85014760171006	norm_Tnfrsf11b	0.69555
StEt 39	3.85014760171006	norm_Tgfb1	0.0649
StEt 39	3.85014760171006	norm_Pla2g4a	1.6483
StEt 39	3.85014760171006	norm_Il6	-3.67976
StEt 39	3.85014760171006	norm_Prdx5	-1.4455
StEt 39	3.85014760171006	norm_Tgfa	2.08774
StEt 39	3.85014760171006	norm_Ccl5	-2.06667
StEt 39	3.85014760171006	norm_Epo	-0.47835

StEt 39	3.85014760171006	norm_Axin1	-1.78175
StEt 39	3.85014760171006	norm_Fst	4.2571
StEt 39	3.85014760171006	norm_Rgma	-0.77757
StEt 39	3.85014760171006	norm_Nadk	3.07399
StEt 39	3.85014760171006	norm_Tnni3	10.5367
StEt 39	3.85014760171006	norm_Notch3	1.72992
StEt 39	3.85014760171006	norm_Snap29	1.78119
StEt 39	3.85014760171006	norm_Cntn1	5.64249
StEt 39	3.85014760171006	norm_Clstn2	-2.6997
StEt 39	3.85014760171006	norm_S100a4	6.37931
StEt 39	3.85014760171006	norm_Ca13	-2.73386
StEt 39	3.85014760171006	norm_Mia	0.32078
StEt 39	3.85014760171006	norm_Cant1	-1.54295
StEt 39	3.85014760171006	norm_Kitlg	-3.96159
StEt 39	3.85014760171006	norm_Gfra1	-0.6102
StEt 39	3.85014760171006	norm_Ppp1r2	-2.48026
StEt 39	3.85014760171006	norm_Adam23	-1.44365
StEt 39	3.85014760171006	norm_Itgb6	-2.84085
StEt 39	3.85014760171006	norm_Cyr61	3.08306
StEt 39	3.85014760171006	norm_Dlk1	0.93526
StEt 39	3.85014760171006	norm_Ahr	-3.14712
StEt 39	3.85014760171006	norm_Ccl2	4.40716
StEt 39	3.85014760171006	norm_Eno2	-0.15614
StEt 39	3.85014760171006	norm_Plin1	-1.31172
StEt 39	3.85014760171006	norm_Wfikkn2	1.38602
StEt 39	3.85014760171006	norm_Flrt2	0.30608
StEt 39	3.85014760171006	norm_Qdpr	-0.20062
StEt 39	3.85014760171006	norm_Fas	1.31004
StEt 39	3.85014760171006	norm_Erbb4	-0.56706
StEt 39	3.85014760171006	norm_Riox2	-1.14709
StEt 39	3.85014760171006	norm_Plxna4	-0.09884
StEt 39	3.85014760171006	norm_Epcam	-1.70397
StEt 39	3.85014760171006	norm_Ccl3	-2.116
StEt 39	3.85014760171006	norm_Crim1	-1.0977
StEt 39	3.85014760171006	norm_Vsig2	1.70625
StEt 39	3.85014760171006	norm_Hgf	0.93892
StEt 39	3.85014760171006	norm_Sez6l2	2.90452
StEt 39	3.85014760171006	norm_Il1a	-1.31298
StEt 39	3.85014760171006	norm_Il23r	3.53432
StEt 39	3.85014760171006	norm_Dll1	0.33912
StEt 39	3.85014760171006	norm_Ddah1	-2.5244
StEt 39	3.85014760171006	norm_Il10	-3.06171
StEt 39	3.85014760171006	norm_Tnfrsf12a	1.66139
StEt 39	3.85014760171006	norm_Acvrl1	-0.36215
StEt 39	3.85014760171006	norm_Lgmn	0.85662
StEt 39	3.85014760171006	norm_Csf2	-2.43566
StEt 39	3.85014760171006	norm_Cxcl9	3.08431
StEt 39	3.85014760171006	norm_Map2k6	0.79013
StEt 39	3.85014760171006	norm_Itgb1bp2	-4.93818
StEt 39	3.85014760171006	norm_Il17f	-4.93264
StEt 39	3.85014760171006	norm_Il1b	-3.469
StEt 39	3.85014760171006	norm_Casp3	1.9201
StEt 39	3.85014760171006	norm_Apbb1ip	-4.0991
StEt 39	3.85014760171006	norm_Wisp1	0.61298
StEt 39	3.85014760171006	norm_Cdh6	0.62789
StEt 39	3.85014760171006	norm_Pdgfb	5.73498
StEt 39	3.85014760171006	norm_Igsf3	-0.18829
StEt 39	3.85014760171006	norm_Tgfbr3	0.14121
StEt 39	3.85014760171006	norm_Cxcl1	2.44252
StEt 39	3.85014760171006	norm_Pak4	-3.33578
StEt 39	3.85014760171006	norm_Cntn4	2.05003
StEt 39	3.85014760171006	norm_Ghrl	1.76042
StEt 39	3.85014760171006	norm_Lpl	0.25238
StEt 39	3.85014760171006	norm_Fstl3	1.51929
StEt 39	3.85014760171006	norm_Dctn2	-2.47012
StEt 39	3.85014760171006	norm_Il5	-3.68148
StEt 39	3.85014760171006	norm_Eda2r	-0.38421
StEt 39	3.85014760171006	norm_Ntf3	-1.69529
StEt 39	3.85014760171006	norm_Tnfsf12	0.65993
StEt 39	3.85014760171006	norm_Ccl20	4.58507
StEt 39	3.85014760171006	norm_Fli1	-4.68187
StEt 39	3.85014760171006	norm_Tpp1	3.52968
StEt 39	3.85014760171006	norm_Tnr	-0.67519
StEt 39	3.85014760171006	norm_Vegfd	-0.6087
StEt 39	3.85014760171006	norm_Parp1	0.0473
StEt 39	3.85014760171006	norm_Tnf	-2.96376
StEt 40	2.56494935746154	norm_Clmp	2.97188
StEt 40	2.56494935746154	norm_Matn2	1.95852
StEt 40	2.56494935746154	norm_Cpe	2.441
StEt 40	2.56494935746154	norm_Gcg	1.38477
StEt 40	2.56494935746154	norm_Gdnf	-2.9594
StEt 40	2.56494935746154	norm_Yes1	-2.02318
StEt 40	2.56494935746154	norm_Il17a	-1.52799
StEt 40	2.56494935746154	norm_Foxo1	-3.22854
StEt 40	2.56494935746154	norm_Tnfrsf11b	1.39117
StEt 40	2.56494935746154	norm_Tgfb1	-0.2527
StEt 40	2.56494935746154	norm_Pla2g4a	1.27069
StEt 40	2.56494935746154	norm_Il6	-3.86029
StEt 40	2.56494935746154	norm_Prdx5	-2.41587
StEt 40	2.56494935746154	norm_Tgfa	1.96715
StEt 40	2.56494935746154	norm_Ccl5	-1.79298
StEt 40	2.56494935746154	norm_Epo	-1.08023
StEt 40	2.56494935746154	norm_Axin1	-2.54909
StEt 40	2.56494935746154	norm_Fst	5.39224
StEt 40	2.56494935746154	norm_Rgma	-0.91846

StEt 40	2.56494935746154	norm_Nadk	1.02386
StEt 40	2.56494935746154	norm_Tnni3	6.37581
StEt 40	2.56494935746154	norm_Notch3	2.16397
StEt 40	2.56494935746154	norm_Snap29	0.50938
StEt 40	2.56494935746154	norm_Cntn1	6.02117
StEt 40	2.56494935746154	norm_Clstn2	-0.77667
StEt 40	2.56494935746154	norm_S100a4	6.99919
StEt 40	2.56494935746154	norm_Ca13	-3.49178
StEt 40	2.56494935746154	norm_Mia	0.61
StEt 40	2.56494935746154	norm_Cant1	-1.35473
StEt 40	2.56494935746154	norm_Kitlg	-4.05611
StEt 40	2.56494935746154	norm_Gfra1	-0.18954
StEt 40	2.56494935746154	norm_Ppp1r2	-3.6868
StEt 40	2.56494935746154	norm_Adam23	-0.39831
StEt 40	2.56494935746154	norm_Itgb6	-2.41501
StEt 40	2.56494935746154	norm_Cyr61	3.44427
StEt 40	2.56494935746154	norm_Dlk1	1.04922
StEt 40	2.56494935746154	norm_Ahr	-3.04607
StEt 40	2.56494935746154	norm_Ccl2	4.28484
StEt 40	2.56494935746154	norm_Eno2	0.03332
StEt 40	2.56494935746154	norm_Plin1	-1.02569
StEt 40	2.56494935746154	norm_Wfikkn2	2.17433
StEt 40	2.56494935746154	norm_Flrt2	0.1553
StEt 40	2.56494935746154	norm_Qdpr	-2.25815
StEt 40	2.56494935746154	norm_Fas	1.15653
StEt 40	2.56494935746154	norm_Erbb4	0.18449
StEt 40	2.56494935746154	norm_Riox2	-2.32648
StEt 40	2.56494935746154	norm_Plxna4	0.06393
StEt 40	2.56494935746154	norm_Epcam	-1.33441
StEt 40	2.56494935746154	norm_Ccl3	-1.6686
StEt 40	2.56494935746154	norm_Crim1	-1.42241
StEt 40	2.56494935746154	norm_Vsig2	1.52589
StEt 40	2.56494935746154	norm_Hgf	-0.10285
StEt 40	2.56494935746154	norm_Sez6l2	3.39548
StEt 40	2.56494935746154	norm_Il1a	-1.63214
StEt 40	2.56494935746154	norm_Il23r	4.8489
StEt 40	2.56494935746154	norm_Dll1	1.17411
StEt 40	2.56494935746154	norm_Ddah1	-4.07958
StEt 40	2.56494935746154	norm_Il10	-2.65109
StEt 40	2.56494935746154	norm_Tnfrsf12a	1.96989
StEt 40	2.56494935746154	norm_Acvrl1	0.23469
StEt 40	2.56494935746154	norm_Lgmn	1.83142
StEt 40	2.56494935746154	norm_Csf2	-2.58086
StEt 40	2.56494935746154	norm_Cxcl9	2.92175
StEt 40	2.56494935746154	norm_Map2k6	-0.49602
StEt 40	2.56494935746154	norm_Itgb1bp2	-4.57931
StEt 40	2.56494935746154	norm_Il17f	-4.25998
StEt 40	2.56494935746154	norm_Il1b	-3.27687
StEt 40	2.56494935746154	norm_Casp3	1.34769
StEt 40	2.56494935746154	norm_Apbb1ip	-4.21889
StEt 40	2.56494935746154	norm_Wisp1	1.02263
StEt 40	2.56494935746154	norm_Cdh6	1.66283
StEt 40	2.56494935746154	norm_Pdgfb	2.24004
StEt 40	2.56494935746154	norm_Igsf3	0.06548
StEt 40	2.56494935746154	norm_Tgfbr3	0.60024
StEt 40	2.56494935746154	norm_Cxcl1	2.04178
StEt 40	2.56494935746154	norm_Pak4	-3.8111
StEt 40	2.56494935746154	norm_Cntn4	2.26662
StEt 40	2.56494935746154	norm_Ghrl	2.36212
StEt 40	2.56494935746154	norm_Lpl	1.42217
StEt 40	2.56494935746154	norm_Fstl3	1.82224
StEt 40	2.56494935746154	norm_Dctn2	-2.53742
StEt 40	2.56494935746154	norm_Il5	-3.646
StEt 40	2.56494935746154	norm_Eda2r	0.11183
StEt 40	2.56494935746154	norm_Ntf3	-1.3418
StEt 40	2.56494935746154	norm_Tnfsf12	0.86507
StEt 40	2.56494935746154	norm_Ccl20	4.7978
StEt 40	2.56494935746154	norm_Fli1	-4.56417
StEt 40	2.56494935746154	norm_Tpp1	4.22157
StEt 40	2.56494935746154	norm_Tnr	-0.07515
StEt 40	2.56494935746154	norm_Vegfd	-0.15087
StEt 40	2.56494935746154	norm_Parp1	-1.08778
StEt 40	2.56494935746154	norm_Tnf	-2.62818
StEt 41	2.19722457733622	norm_Clmp	2.45144
StEt 41	2.19722457733622	norm_Matn2	1.27615
StEt 41	2.19722457733622	norm_Cpe	2.02485
StEt 41	2.19722457733622	norm_Gcg	0.37151
StEt 41	2.19722457733622	norm_Gdnf	-3.411
StEt 41	2.19722457733622	norm_Yes1	-2.38771
StEt 41	2.19722457733622	norm_Il17a	-1.16677
StEt 41	2.19722457733622	norm_Foxo1	-2.83724
StEt 41	2.19722457733622	norm_Tnfrsf11b	-0.03433
StEt 41	2.19722457733622	norm_Tgfb1	-0.38497
StEt 41	2.19722457733622	norm_Pla2g4a	0.8317
StEt 41	2.19722457733622	norm_Il6	-3.6236
StEt 41	2.19722457733622	norm_Prdx5	-0.75514
StEt 41	2.19722457733622	norm_Tgfa	1.32403
StEt 41	2.19722457733622	norm_Ccl5	-2.03531
StEt 41	2.19722457733622	norm_Epo	-1.57787
StEt 41	2.19722457733622	norm_Axin1	-1.82105
StEt 41	2.19722457733622	norm_Fst	5.19508
StEt 41	2.19722457733622	norm_Rgma	-0.81318
StEt 41	2.19722457733622	norm_Nadk	3.20966
StEt 41	2.19722457733622	norm_Tnni3	9.78781
StEt 41	2.19722457733622	norm_Notch3	1.70794

StEt 41	2.19722457733622	norm_Snap29	1.65153
StEt 41	2.19722457733622	norm_Cntn1	5.43071
StEt 41	2.19722457733622	norm_Clstn2	-2.23274
StEt 41	2.19722457733622	norm_S100a4	6.61426
StEt 41	2.19722457733622	norm_Ca13	-3.62893
StEt 41	2.19722457733622	norm_Mia	0.83354
StEt 41	2.19722457733622	norm_Cant1	-1.44122
StEt 41	2.19722457733622	norm_Kitlg	-4.18314
StEt 41	2.19722457733622	norm_Gfra1	-0.80493
StEt 41	2.19722457733622	norm_Ppp1r2	-3.09398
StEt 41	2.19722457733622	norm_Adam23	-1.42222
StEt 41	2.19722457733622	norm_Itgb6	-2.72988
StEt 41	2.19722457733622	norm_Cyr61	3.11364
StEt 41	2.19722457733622	norm_Dlk1	-0.23281
StEt 41	2.19722457733622	norm_Ahr	-2.98569
StEt 41	2.19722457733622	norm_Ccl2	3.73331
StEt 41	2.19722457733622	norm_Eno2	-0.61271
StEt 41	2.19722457733622	norm_Plin1	-2.79754
StEt 41	2.19722457733622	norm_Wfikkn2	1.54898
StEt 41	2.19722457733622	norm_Flrt2	-0.43818
StEt 41	2.19722457733622	norm_Qdpr	0.22601
StEt 41	2.19722457733622	norm_Fas	1.38903
StEt 41	2.19722457733622	norm_Erbb4	-0.07314
StEt 41	2.19722457733622	norm_Riox2	-0.66392
StEt 41	2.19722457733622	norm_Plxna4	0.41015
StEt 41	2.19722457733622	norm_Epcam	-1.02283
StEt 41	2.19722457733622	norm_Ccl3	-1.67761
StEt 41	2.19722457733622	norm_Crim1	-1.91381
StEt 41	2.19722457733622	norm_Vsig2	1.70598
StEt 41	2.19722457733622	norm_Hgf	0.28461
StEt 41	2.19722457733622	norm_Sez6l2	2.88793
StEt 41	2.19722457733622	norm_Il1a	-1.78694
StEt 41	2.19722457733622	norm_Il23r	3.74408
StEt 41	2.19722457733622	norm_Dll1	0.39145
StEt 41	2.19722457733622	norm_Ddah1	-1.96145
StEt 41	2.19722457733622	norm_Il10	-2.65522
StEt 41	2.19722457733622	norm_Tnfrsf12a	1.71467
StEt 41	2.19722457733622	norm_Acvrl1	-0.24243
StEt 41	2.19722457733622	norm_Lgmn	0.89888
StEt 41	2.19722457733622	norm_Csf2	-2.4411
StEt 41	2.19722457733622	norm_Cxcl9	2.11529
StEt 41	2.19722457733622	norm_Map2k6	-0.08571
StEt 41	2.19722457733622	norm_Itgb1bp2	-4.21328
StEt 41	2.19722457733622	norm_Il17f	-4.60208
StEt 41	2.19722457733622	norm_Il1b	-1.68214
StEt 41	2.19722457733622	norm_Casp3	2.09008
StEt 41	2.19722457733622	norm_Apbb1ip	-4.11692
StEt 41	2.19722457733622	norm_Wisp1	0.34948
StEt 41	2.19722457733622	norm_Cdh6	0.66864
StEt 41	2.19722457733622	norm_Pdgfb	4.42363
StEt 41	2.19722457733622	norm_Igsf3	-0.53677
StEt 41	2.19722457733622	norm_Tgfbr3	0.51119
StEt 41	2.19722457733622	norm_Cxcl1	2.34994
StEt 41	2.19722457733622	norm_Pak4	-3.19455
StEt 41	2.19722457733622	norm_Cntn4	2.37444
StEt 41	2.19722457733622	norm_Ghrl	2.86978
StEt 41	2.19722457733622	norm_Lpl	1.16475
StEt 41	2.19722457733622	norm_Fstl3	1.58113
StEt 41	2.19722457733622	norm_Dctn2	-2.2093
StEt 41	2.19722457733622	norm_Il5	-3.04709
StEt 41	2.19722457733622	norm_Eda2r	0.27156
StEt 41	2.19722457733622	norm_Ntf3	-1.39836
StEt 41	2.19722457733622	norm_Tnfsf12	0.53962
StEt 41	2.19722457733622	norm_Ccl20	5.50152
StEt 41	2.19722457733622	norm_Fli1	-4.4224
StEt 41	2.19722457733622	norm_Tpp1	3.54785
StEt 41	2.19722457733622	norm_Tnr	-0.57827
StEt 41	2.19722457733622	norm_Vegfd	-0.56602
StEt 41	2.19722457733622	norm_Parp1	0.15997
StEt 41	2.19722457733622	norm_Tnf	-2.73446
StEt 42	2.70805020110221	norm_Clmp	2.54882
StEt 42	2.70805020110221	norm_Matn2	1.47561
StEt 42	2.70805020110221	norm_Cpe	1.98281
StEt 42	2.70805020110221	norm_Gcg	1.718
StEt 42	2.70805020110221	norm_Gdnf	-3.60882
StEt 42	2.70805020110221	norm_Yes1	-2.61297
StEt 42	2.70805020110221	norm_Il17a	-0.50588
StEt 42	2.70805020110221	norm_Foxo1	-3.63012
StEt 42	2.70805020110221	norm_Tnfrsf11b	1.12797
StEt 42	2.70805020110221	norm_Tgfb1	-0.10215
StEt 42	2.70805020110221	norm_Pla2g4a	1.17002
StEt 42	2.70805020110221	norm_Il6	-2.42736
StEt 42	2.70805020110221	norm_Prdx5	-1.4112
StEt 42	2.70805020110221	norm_Tgfa	1.70554
StEt 42	2.70805020110221	norm_Ccl5	-1.49274
StEt 42	2.70805020110221	norm_Epo	-0.6467
StEt 42	2.70805020110221	norm_Axin1	-2.61269
StEt 42	2.70805020110221	norm_Fst	5.13063
StEt 42	2.70805020110221	norm_Rgma	-1.2276
StEt 42	2.70805020110221	norm_Nadk	1.22163
StEt 42	2.70805020110221	norm_Tnni3	6.32303
StEt 42	2.70805020110221	norm_Notch3	1.82035
StEt 42	2.70805020110221	norm_Snap29	0.94096
StEt 42	2.70805020110221	norm_Cntn1	5.50531
StEt 42	2.70805020110221	norm_Clstn2	-2.29498

StEt 42	2.70805020110221	norm_S100a4	6.47598
StEt 42	2.70805020110221	norm_Ca13	-3.10651
StEt 42	2.70805020110221	norm_Mia	0.87581
StEt 42	2.70805020110221	norm_Cant1	-1.17903
StEt 42	2.70805020110221	norm_Kitlg	-4.21882
StEt 42	2.70805020110221	norm_Gfra1	-0.89338
StEt 42	2.70805020110221	norm_Ppp1r2	-4.13842
StEt 42	2.70805020110221	norm_Adam23	-0.27168
StEt 42	2.70805020110221	norm_Itgb6	-2.63158
StEt 42	2.70805020110221	norm_Cyr61	2.84259
StEt 42	2.70805020110221	norm_Dlk1	0.6604
StEt 42	2.70805020110221	norm_Ahr	-3.16585
StEt 42	2.70805020110221	norm_Ccl2	5.70465
StEt 42	2.70805020110221	norm_Eno2	-0.65581
StEt 42	2.70805020110221	norm_Plin1	-2.69218
StEt 42	2.70805020110221	norm_Wfikkn2	2.38408
StEt 42	2.70805020110221	norm_Flrt2	0.07107
StEt 42	2.70805020110221	norm_Qdpr	-2.02551
StEt 42	2.70805020110221	norm_Fas	1.5818
StEt 42	2.70805020110221	norm_Erbb4	-0.76257
StEt 42	2.70805020110221	norm_Riox2	-3.50376
StEt 42	2.70805020110221	norm_Plxna4	0.20812
StEt 42	2.70805020110221	norm_Epcam	-1.38678
StEt 42	2.70805020110221	norm_Ccl3	-1.09827
StEt 42	2.70805020110221	norm_Crim1	-1.49166
StEt 42	2.70805020110221	norm_Vsig2	2.26324
StEt 42	2.70805020110221	norm_Hgf	-0.10882
StEt 42	2.70805020110221	norm_Sez6l2	2.66643
StEt 42	2.70805020110221	norm_Il1a	-1.68973
StEt 42	2.70805020110221	norm_Il23r	3.67579
StEt 42	2.70805020110221	norm_Dll1	1.28153
StEt 42	2.70805020110221	norm_Ddah1	-4.00051
StEt 42	2.70805020110221	norm_Il10	-1.48197
StEt 42	2.70805020110221	norm_Tnfrsf12a	1.83194
StEt 42	2.70805020110221	norm_Acvrl1	0.24354
StEt 42	2.70805020110221	norm_Lgmn	1.89785
StEt 42	2.70805020110221	norm_Csf2	-1.78324
StEt 42	2.70805020110221	norm_Cxcl9	5.08074
StEt 42	2.70805020110221	norm_Map2k6	-0.58106
StEt 42	2.70805020110221	norm_Itgb1bp2	-4.79302
StEt 42	2.70805020110221	norm_Il17f	-4.54561
StEt 42	2.70805020110221	norm_Il1b	-2.12899
StEt 42	2.70805020110221	norm_Casp3	1.22818
StEt 42	2.70805020110221	norm_Apbb1ip	-3.80125
StEt 42	2.70805020110221	norm_Wisp1	1.10423
StEt 42	2.70805020110221	norm_Cdh6	1.14696
StEt 42	2.70805020110221	norm_Pdgfb	2.85988
StEt 42	2.70805020110221	norm_Igsf3	-0.72819
StEt 42	2.70805020110221	norm_Tgfbr3	0.54623
StEt 42	2.70805020110221	norm_Cxcl1	2.58464
StEt 42	2.70805020110221	norm_Pak4	-3.45712
StEt 42	2.70805020110221	norm_Cntn4	2.79391
StEt 42	2.70805020110221	norm_Ghrl	2.70178
StEt 42	2.70805020110221	norm_Lpl	0.8484
StEt 42	2.70805020110221	norm_Fstl3	1.81883
StEt 42	2.70805020110221	norm_Dctn2	-2.5066
StEt 42	2.70805020110221	norm_Il5	-2.893
StEt 42	2.70805020110221	norm_Eda2r	0.39019
StEt 42	2.70805020110221	norm_Ntf3	-1.26075
StEt 42	2.70805020110221	norm_Tnfsf12	0.55684
StEt 42	2.70805020110221	norm_Ccl20	5.21721
StEt 42	2.70805020110221	norm_Fli1	-4.39734
StEt 42	2.70805020110221	norm_Tpp1	3.94834
StEt 42	2.70805020110221	norm_Tnr	-0.97152
StEt 42	2.70805020110221	norm_Vegfd	-0.46305
StEt 42	2.70805020110221	norm_Parp1	-0.6696
StEt 42	2.70805020110221	norm_Tnf	-2.10528
StEt 43	1.6094379124341	norm_Clmp	2.57559
StEt 43	1.6094379124341	norm_Matn2	1.60868
StEt 43	1.6094379124341	norm_Cpe	2.31399
StEt 43	1.6094379124341	norm_Gcg	-0.34617
StEt 43	1.6094379124341	norm_Gdnf	-3.50853
StEt 43	1.6094379124341	norm_Yes1	-1.78197
StEt 43	1.6094379124341	norm_Il17a	-0.90833
StEt 43	1.6094379124341	norm_Foxo1	-2.35307
StEt 43	1.6094379124341	norm_Tnfrsf11b	1.37454
StEt 43	1.6094379124341	norm_Tgfb1	-0.32317
StEt 43	1.6094379124341	norm_Pla2g4a	0.85542
StEt 43	1.6094379124341	norm_Il6	-3.76802
StEt 43	1.6094379124341	norm_Prdx5	-1.28588
StEt 43	1.6094379124341	norm_Tgfa	3.10607
StEt 43	1.6094379124341	norm_Ccl5	-1.57149
StEt 43	1.6094379124341	norm_Epo	-1.00126
StEt 43	1.6094379124341	norm_Axin1	-1.48939
StEt 43	1.6094379124341	norm_Fst	4.90112
StEt 43	1.6094379124341	norm_Rgma	-0.6393
StEt 43	1.6094379124341	norm_Nadk	3.36085
StEt 43	1.6094379124341	norm_Tnni3	9.1996
StEt 43	1.6094379124341	norm_Notch3	1.60322
StEt 43	1.6094379124341	norm_Snap29	2.3638
StEt 43	1.6094379124341	norm_Cntn1	5.52751
StEt 43	1.6094379124341	norm_Clstn2	-1.80382
StEt 43	1.6094379124341	norm_S100a4	5.9511
StEt 43	1.6094379124341	norm_Ca13	-3.28862
StEt 43	1.6094379124341	norm_Mia	0.70253

StEt 43 1.6094379124341 norm_Cant1 -1.5304
StEt 43 1.6094379124341 norm_Kitlg -4.47039
StEt 43 1.6094379124341 norm_Gfra1 -0.13861
StEt 43 1.6094379124341 norm_Ppp1r2 -2.92574
StEt 43 1.6094379124341 norm_Adam23 -0.56471
StEt 43 1.6094379124341 norm_Itgb6 -3.15083
StEt 43 1.6094379124341 norm_Cyr61 3.18028
StEt 43 1.6094379124341 norm_Dlk1 0.73832
StEt 43 1.6094379124341 norm_Ahr -2.83167
StEt 43 1.6094379124341 norm_Ccl2 4.4109
StEt 43 1.6094379124341 norm_Eno2 -0.94175
StEt 43 1.6094379124341 norm_Plin1 -1.64622
StEt 43 1.6094379124341 norm_Wfikkn2 1.17061
StEt 43 1.6094379124341 norm_Flrt2 -0.13294
StEt 43 1.6094379124341 norm_Qdpr 0.77456
StEt 43 1.6094379124341 norm_Fas 1.61666
StEt 43 1.6094379124341 norm_Erbb4 -0.56706
StEt 43 1.6094379124341 norm_Riox2 -0.55514
StEt 43 1.6094379124341 norm_Plxna4 0.10675
StEt 43 1.6094379124341 norm_Epcam -1.815
StEt 43 1.6094379124341 norm_Ccl3 -2.49471
StEt 43 1.6094379124341 norm_Crim1 -1.46591
StEt 43 1.6094379124341 norm_Vsig2 2.31699
StEt 43 1.6094379124341 norm_Hgf -0.16417
StEt 43 1.6094379124341 norm_Sez6l2 2.70111
StEt 43 1.6094379124341 norm_Il1a -1.28018
StEt 43 1.6094379124341 norm_Il23r 3.44912
StEt 43 1.6094379124341 norm_Dll1 0.79011
StEt 43 1.6094379124341 norm_Ddah1 -1.38607
StEt 43 1.6094379124341 norm_Il10 -2.61366
StEt 43 1.6094379124341 norm_Tnfrsf12a 2.01421
StEt 43 1.6094379124341 norm_Acvrl1 0.3441
StEt 43 1.6094379124341 norm_Lgmn 2.04127
StEt 43 1.6094379124341 norm_Csf2 -3.04445
StEt 43 1.6094379124341 norm_Cxcl9 3.66
StEt 43 1.6094379124341 norm_Map2k6 0.2972
StEt 43 1.6094379124341 norm_Itgb1bp2 -5.10228
StEt 43 1.6094379124341 norm_Il17f -4.76727
StEt 43 1.6094379124341 norm_Il1b -3.40928
StEt 43 1.6094379124341 norm_Casp3 1.87304
StEt 43 1.6094379124341 norm_Apbb1ip -4.3279
StEt 43 1.6094379124341 norm_Wisp1 0.60319
StEt 43 1.6094379124341 norm_Cdh6 0.76922
StEt 43 1.6094379124341 norm_Pdgfb 2.34014
StEt 43 1.6094379124341 norm_Igsf3 -0.72745
StEt 43 1.6094379124341 norm_Tgfbr3 0.28591
StEt 43 1.6094379124341 norm_Cxcl1 1.68815
StEt 43 1.6094379124341 norm_Pak4 -4.0028
StEt 43 1.6094379124341 norm_Cntn4 0.19809
StEt 43 1.6094379124341 norm_Ghrl 2.4273
StEt 43 1.6094379124341 norm_Lpl 0.75973
StEt 43 1.6094379124341 norm_Fstl3 1.78603
StEt 43 1.6094379124341 norm_Dctn2 -1.81703
StEt 43 1.6094379124341 norm_Il5 -3.80749
StEt 43 1.6094379124341 norm_Eda2r 0.21058
StEt 43 1.6094379124341 norm_Ntf3 -1.58535
StEt 43 1.6094379124341 norm_Tnfsf12 0.5394
StEt 43 1.6094379124341 norm_Ccl20 5.62995
StEt 43 1.6094379124341 norm_Fli1 -4.60413
StEt 43 1.6094379124341 norm_Tpp1 3.58071
StEt 43 1.6094379124341 norm_Tnr -1.01168
StEt 43 1.6094379124341 norm_Vegfd -0.55918
StEt 43 1.6094379124341 norm_Parp1 -1.31535
StEt 43 1.6094379124341 norm_Tnf -2.92165
StEt 44 1.94591014905531 norm_Clmp 2.51373
StEt 44 1.94591014905531 norm_Matn2 1.43645
StEt 44 1.94591014905531 norm_Cpe 1.64076
StEt 44 1.94591014905531 norm_Gcg -5.04574
StEt 44 1.94591014905531 norm_Gdnf -3.51449
StEt 44 1.94591014905531 norm_Yes1 -2.56621
StEt 44 1.94591014905531 norm_Il17a -3.50603
StEt 44 1.94591014905531 norm_Foxo1 -3.6351
StEt 44 1.94591014905531 norm_Tnfrsf11b 0.92202
StEt 44 1.94591014905531 norm_Tgfb1 0.66823
StEt 44 1.94591014905531 norm_Pla2g4a 3.268
StEt 44 1.94591014905531 norm_Il6 -0.88191
StEt 44 1.94591014905531 norm_Prdx5 2.51562
StEt 44 1.94591014905531 norm_Tgfa -3.06662
StEt 44 1.94591014905531 norm_Ccl5 -1.95214
StEt 44 1.94591014905531 norm_Epo -1.52293
StEt 44 1.94591014905531 norm_Axin1 -1.8057
StEt 44 1.94591014905531 norm_Fst 4.8218
StEt 44 1.94591014905531 norm_Rgma -1.38556
StEt 44 1.94591014905531 norm_Nadk 3.58663
StEt 44 1.94591014905531 norm_Tnni3 5.57746
StEt 44 1.94591014905531 norm_Notch3 1.52148
StEt 44 1.94591014905531 norm_Snap29 -0.07531
StEt 44 1.94591014905531 norm_Cntn1 5.61547
StEt 44 1.94591014905531 norm_Clstn2 -1.96872
StEt 44 1.94591014905531 norm_S100a4 5.2964
StEt 44 1.94591014905531 norm_Ca13 -2.11713
StEt 44 1.94591014905531 norm_Mia -0.56073
StEt 44 1.94591014905531 norm_Cant1 -0.90556
StEt 44 1.94591014905531 norm_Kitlg -3.9003
StEt 44 1.94591014905531 norm_Gfra1 -0.37064

StEt 44 1.94591014905531 norm_Ppp1r2 -2.04314
StEt 44 1.94591014905531 norm_Adam23 -1.1583
StEt 44 1.94591014905531 norm_Itgb6 -2.4798
StEt 44 1.94591014905531 norm_Cyr61 2.28944
StEt 44 1.94591014905531 norm_Dlk1 0.03473
StEt 44 1.94591014905531 norm_Ahr -3.33495
StEt 44 1.94591014905531 norm_Ccl2 5.09654
StEt 44 1.94591014905531 norm_Eno2 -1.32484
StEt 44 1.94591014905531 norm_Plin1 -2.93108
StEt 44 1.94591014905531 norm_Wfikkn2 0.77364
StEt 44 1.94591014905531 norm_Flrt2 -0.45914
StEt 44 1.94591014905531 norm_Qdpr 1.0211
StEt 44 1.94591014905531 norm_Fas 1.68877
StEt 44 1.94591014905531 norm_Erbb4 -0.38111
StEt 44 1.94591014905531 norm_Riox2 0.38016
StEt 44 1.94591014905531 norm_Plxna4 0.77641
StEt 44 1.94591014905531 norm_Epcam -3.01828
StEt 44 1.94591014905531 norm_Ccl3 -1.82146
StEt 44 1.94591014905531 norm_Crim1 0.38235
StEt 44 1.94591014905531 norm_Vsig2 1.57956
StEt 44 1.94591014905531 norm_Hgf 3.30141
StEt 44 1.94591014905531 norm_Sez6l2 2.66994
StEt 44 1.94591014905531 norm_Il1a 1.14397
StEt 44 1.94591014905531 norm_Il23r 3.96455
StEt 44 1.94591014905531 norm_Dll1 0.55793
StEt 44 1.94591014905531 norm_Ddah1 0.03872
StEt 44 1.94591014905531 norm_Il10 -2.79117
StEt 44 1.94591014905531 norm_Tnfrsf12a 1.00614
StEt 44 1.94591014905531 norm_Acvrl1 -0.42797
StEt 44 1.94591014905531 norm_Lgmn 1.60227
StEt 44 1.94591014905531 norm_Csf2 -2.78433
StEt 44 1.94591014905531 norm_Cxcl9 2.09382
StEt 44 1.94591014905531 norm_Map2k6 1.90909
StEt 44 1.94591014905531 norm_Itgb1bp2 -4.53658
StEt 44 1.94591014905531 norm_Il17f -4.98228
StEt 44 1.94591014905531 norm_Il1b -1.9038
StEt 44 1.94591014905531 norm_Casp3 4.39744
StEt 44 1.94591014905531 norm_Apbb1ip -3.36606
StEt 44 1.94591014905531 norm_Wisp1 0.35244
StEt 44 1.94591014905531 norm_Cdh6 0.85546
StEt 44 1.94591014905531 norm_Pdgfb 6.74047
StEt 44 1.94591014905531 norm_Igsf3 -0.56521
StEt 44 1.94591014905531 norm_Tgfbr3 -0.36193
StEt 44 1.94591014905531 norm_Cxcl1 6.06458
StEt 44 1.94591014905531 norm_Pak4 -4.04443
StEt 44 1.94591014905531 norm_Cntn4 1.33929
StEt 44 1.94591014905531 norm_Ghrl -3.05256
StEt 44 1.94591014905531 norm_Lpl 0.77157
StEt 44 1.94591014905531 norm_Fstl3 1.28531
StEt 44 1.94591014905531 norm_Dctn2 -1.9334
StEt 44 1.94591014905531 norm_Il5 -3.4549
StEt 44 1.94591014905531 norm_Eda2r 0.08019
StEt 44 1.94591014905531 norm_Ntf3 -2.17721
StEt 44 1.94591014905531 norm_Tnfsf12 1.22355
StEt 44 1.94591014905531 norm_Ccl20 3.03831
StEt 44 1.94591014905531 norm_Fli1 -4.36905
StEt 44 1.94591014905531 norm_Tpp1 3.67496
StEt 44 1.94591014905531 norm_Tnr -1.01195
StEt 44 1.94591014905531 norm_Vegfd -0.97813
StEt 44 1.94591014905531 norm_Parp1 2.01358
StEt 44 1.94591014905531 norm_Tnf -3.05769
StEt 45 2.484906649788 norm_Clmp 2.91413
StEt 45 2.484906649788 norm_Matn2 1.77886
StEt 45 2.484906649788 norm_Cpe 2.347
StEt 45 2.484906649788 norm_Gcg 1.89353
StEt 45 2.484906649788 norm_Gdnf -3.49816
StEt 45 2.484906649788 norm_Yes1 -2.47507
StEt 45 2.484906649788 norm_Il17a -0.66305
StEt 45 2.484906649788 norm_Foxo1 -3.03542
StEt 45 2.484906649788 norm_Tnfrsf11b 1.1487
StEt 45 2.484906649788 norm_Tgfb1 0.16045
StEt 45 2.484906649788 norm_Pla2g4a 0.75342
StEt 45 2.484906649788 norm_Il6 -4.38416
StEt 45 2.484906649788 norm_Prdx5 -2.03342
StEt 45 2.484906649788 norm_Tgfa 1.91449
StEt 45 2.484906649788 norm_Ccl5 -2.85372
StEt 45 2.484906649788 norm_Epo -0.90578
StEt 45 2.484906649788 norm_Axin1 -2.25465
StEt 45 2.484906649788 norm_Fst 4.73145
StEt 45 2.484906649788 norm_Rgma -1.20455
StEt 45 2.484906649788 norm_Nadk 3.1142
StEt 45 2.484906649788 norm_Tnni3 4.94074
StEt 45 2.484906649788 norm_Notch3 1.98143
StEt 45 2.484906649788 norm_Snap29 1.37388
StEt 45 2.484906649788 norm_Cntn1 5.49058
StEt 45 2.484906649788 norm_Clstn2 -1.17282
StEt 45 2.484906649788 norm_S100a4 6.46341
StEt 45 2.484906649788 norm_Ca13 -3.09264
StEt 45 2.484906649788 norm_Mia 0.41913
StEt 45 2.484906649788 norm_Cant1 -1.65835
StEt 45 2.484906649788 norm_Kitlg -4.45549
StEt 45 2.484906649788 norm_Gfra1 -0.40519
StEt 45 2.484906649788 norm_Ppp1r2 -3.69937
StEt 45 2.484906649788 norm_Adam23 -0.84909
StEt 45 2.484906649788 norm_Itgb6 -3.00476

StEt 45 2.484906649788 norm_Cyr61 3.26221
StEt 45 2.484906649788 norm_Dlk1 0.79328
StEt 45 2.484906649788 norm_Ahr -3.32584
StEt 45 2.484906649788 norm_Ccl2 4.39975
StEt 45 2.484906649788 norm_Eno2 -0.44032
StEt 45 2.484906649788 norm_Plin1 -2.24088
StEt 45 2.484906649788 norm_Wfikkn2 1.7434
StEt 45 2.484906649788 norm_Flrt2 0.11678
StEt 45 2.484906649788 norm_Qdpr 0.09564
StEt 45 2.484906649788 norm_Fas 1.47731
StEt 45 2.484906649788 norm_Erbb4 -0.10894
StEt 45 2.484906649788 norm_Riox2 -0.84381
StEt 45 2.484906649788 norm_Plxna4 0.38713
StEt 45 2.484906649788 norm_Epcam -0.57315
StEt 45 2.484906649788 norm_Ccl3 -1.89893
StEt 45 2.484906649788 norm_Crim1 -1.76202
StEt 45 2.484906649788 norm_Vsig2 1.91997
StEt 45 2.484906649788 norm_Hgf 0.47488
StEt 45 2.484906649788 norm_Sez6l2 3.27801
StEt 45 2.484906649788 norm_Il1a -1.84977
StEt 45 2.484906649788 norm_Il23r 4.03408
StEt 45 2.484906649788 norm_Dll1 0.54226
StEt 45 2.484906649788 norm_Ddah1 -2.18068
StEt 45 2.484906649788 norm_Il10 -2.77868
StEt 45 2.484906649788 norm_Tnfrsf12a 1.79405
StEt 45 2.484906649788 norm_Acvrl1 0.0608
StEt 45 2.484906649788 norm_Lgmn 1.74944
StEt 45 2.484906649788 norm_Csf2 -2.57913
StEt 45 2.484906649788 norm_Cxcl9 3.26359
StEt 45 2.484906649788 norm_Map2k6 -0.43562
StEt 45 2.484906649788 norm_Itgb1bp2 -4.75113
StEt 45 2.484906649788 norm_Il17f -4.83985
StEt 45 2.484906649788 norm_Il1b -3.4399
StEt 45 2.484906649788 norm_Casp3 2.05904
StEt 45 2.484906649788 norm_Apbb1ip -4.34858
StEt 45 2.484906649788 norm_Wisp1 0.53246
StEt 45 2.484906649788 norm_Cdh6 1.0682
StEt 45 2.484906649788 norm_Pdgfb 5.06596
StEt 45 2.484906649788 norm_Igsf3 -0.36679
StEt 45 2.484906649788 norm_Tgfbr3 0.57359
StEt 45 2.484906649788 norm_Cxcl1 2.33558
StEt 45 2.484906649788 norm_Pak4 -3.45299
StEt 45 2.484906649788 norm_Cntn4 2.88014
StEt 45 2.484906649788 norm_Ghrl 2.56529
StEt 45 2.484906649788 norm_Lpl 0.96631
StEt 45 2.484906649788 norm_Fstl3 1.67544
StEt 45 2.484906649788 norm_Dctn2 -2.68335
StEt 45 2.484906649788 norm_Il5 -3.64253
StEt 45 2.484906649788 norm_Eda2r -0.02374
StEt 45 2.484906649788 norm_Ntf3 -1.62045
StEt 45 2.484906649788 norm_Tnfsf12 1.13968
StEt 45 2.484906649788 norm_Ccl20 4.8847
StEt 45 2.484906649788 norm_Fli1 -5.06983
StEt 45 2.484906649788 norm_Tpp1 3.69276
StEt 45 2.484906649788 norm_Tnr -0.30602
StEt 45 2.484906649788 norm_Vegfd -0.33678
StEt 45 2.484906649788 norm_Parp1 0.39609
StEt 45 2.484906649788 norm_Tnf -3.10807
StEt 46 2.39789527279837 norm_Clmp 3.23729
StEt 46 2.39789527279837 norm_Matn2 1.7323
StEt 46 2.39789527279837 norm_Cpe 2.49301
StEt 46 2.39789527279837 norm_Gcg 1.3025
StEt 46 2.39789527279837 norm_Gdnf -2.92567
StEt 46 2.39789527279837 norm_Yes1 -2.50395
StEt 46 2.39789527279837 norm_Il17a -1.6591
StEt 46 2.39789527279837 norm_Foxo1 -3.07507
StEt 46 2.39789527279837 norm_Tnfrsf11b 1.03793
StEt 46 2.39789527279837 norm_Tgfb1 -0.02915
StEt 46 2.39789527279837 norm_Pla2g4a 1.25899
StEt 46 2.39789527279837 norm_Il6 -3.13392
StEt 46 2.39789527279837 norm_Prdx5 -1.88522
StEt 46 2.39789527279837 norm_Tgfa 1.2155
StEt 46 2.39789527279837 norm_Ccl5 -1.95397
StEt 46 2.39789527279837 norm_Epo -0.17603
StEt 46 2.39789527279837 norm_Axin1 -2.52099
StEt 46 2.39789527279837 norm_Fst 4.92535
StEt 46 2.39789527279837 norm_Rgma -1.21955
StEt 46 2.39789527279837 norm_Nadk 1.35373
StEt 46 2.39789527279837 norm_Tnni3 7.72466
StEt 46 2.39789527279837 norm_Notch3 2.0337
StEt 46 2.39789527279837 norm_Snap29 0.80535
StEt 46 2.39789527279837 norm_Cntn1 5.68262
StEt 46 2.39789527279837 norm_Clstn2 -0.60722
StEt 46 2.39789527279837 norm_S100a4 6.64516
StEt 46 2.39789527279837 norm_Ca13 -3.30844
StEt 46 2.39789527279837 norm_Mia 1.02834
StEt 46 2.39789527279837 norm_Cant1 -1.19093
StEt 46 2.39789527279837 norm_Kitlg -4.17668
StEt 46 2.39789527279837 norm_Gfra1 -0.32849
StEt 46 2.39789527279837 norm_Ppp1r2 -3.31921
StEt 46 2.39789527279837 norm_Adam23 -0.3139
StEt 46 2.39789527279837 norm_Itgb6 -2.8665
StEt 46 2.39789527279837 norm_Cyr61 2.87629
StEt 46 2.39789527279837 norm_Dlk1 1.27716
StEt 46 2.39789527279837 norm_Ahr -3.02887

StEt 46 2.39789527279837 norm_Ccl2 4.6116
StEt 46 2.39789527279837 norm_Eno2 -0.38751
StEt 46 2.39789527279837 norm_Plin1 -2.67695
StEt 46 2.39789527279837 norm_Wfikkn2 1.87783
StEt 46 2.39789527279837 norm_Flrt2 0.4084
StEt 46 2.39789527279837 norm_Qdpr -1.9784
StEt 46 2.39789527279837 norm_Fas 1.25219
StEt 46 2.39789527279837 norm_Erbb4 -0.36373
StEt 46 2.39789527279837 norm_Riox2 -2.29904
StEt 46 2.39789527279837 norm_Plxna4 -0.04503
StEt 46 2.39789527279837 norm_Epcam -1.22508
StEt 46 2.39789527279837 norm_Ccl3 -1.63259
StEt 46 2.39789527279837 norm_Crim1 -1.32759
StEt 46 2.39789527279837 norm_Vsig2 1.85928
StEt 46 2.39789527279837 norm_Hgf 0.37243
StEt 46 2.39789527279837 norm_Sez6l2 3.16176
StEt 46 2.39789527279837 norm_Il1a -1.89681
StEt 46 2.39789527279837 norm_Il23r 4.39466
StEt 46 2.39789527279837 norm_Dll1 1.08833
StEt 46 2.39789527279837 norm_Ddah1 -3.87664
StEt 46 2.39789527279837 norm_Il10 -2.56621
StEt 46 2.39789527279837 norm_Tnfrsf12a 2.09051
StEt 46 2.39789527279837 norm_Acvrl1 0.36023
StEt 46 2.39789527279837 norm_Lgmn 1.84309
StEt 46 2.39789527279837 norm_Csf2 -2.35504
StEt 46 2.39789527279837 norm_Cxcl9 3.64664
StEt 46 2.39789527279837 norm_Map2k6 -0.88812
StEt 46 2.39789527279837 norm_Itgb1bp2 -4.45346
StEt 46 2.39789527279837 norm_Il17f -4.7924
StEt 46 2.39789527279837 norm_Il1b -2.89297
StEt 46 2.39789527279837 norm_Casp3 1.47897
StEt 46 2.39789527279837 norm_Apbb1ip -4.0825
StEt 46 2.39789527279837 norm_Wisp1 0.8641
StEt 46 2.39789527279837 norm_Cdh6 1.06691
StEt 46 2.39789527279837 norm_Pdgfb 3.52191
StEt 46 2.39789527279837 norm_Igsf3 -0.19773
StEt 46 2.39789527279837 norm_Tgfbr3 0.72618
StEt 46 2.39789527279837 norm_Cxcl1 2.77034
StEt 46 2.39789527279837 norm_Pak4 -3.70978
StEt 46 2.39789527279837 norm_Cntn4 2.24426
StEt 46 2.39789527279837 norm_Ghrl 2.32914
StEt 46 2.39789527279837 norm_Lpl 0.94143
StEt 46 2.39789527279837 norm_Fstl3 1.74083
StEt 46 2.39789527279837 norm_Dctn2 -2.456
StEt 46 2.39789527279837 norm_Il5 -4.19234
StEt 46 2.39789527279837 norm_Eda2r 0.29724
StEt 46 2.39789527279837 norm_Ntf3 -1.69976
StEt 46 2.39789527279837 norm_Tnfsf12 0.81241
StEt 46 2.39789527279837 norm_Ccl20 4.36741
StEt 46 2.39789527279837 norm_Fli1 -4.58909
StEt 46 2.39789527279837 norm_Tpp1 3.96294
StEt 46 2.39789527279837 norm_Tnr -0.41653
StEt 46 2.39789527279837 norm_Vegfd -0.13981
StEt 46 2.39789527279837 norm_Parp1 -0.44761
StEt 46 2.39789527279837 norm_Tnf -2.90938
StEt 47 2.19722457733622 norm_Clmp 3.14402
StEt 47 2.19722457733622 norm_Matn2 1.68066
StEt 47 2.19722457733622 norm_Cpe 2.27594
StEt 47 2.19722457733622 norm_Gcg 0.85686
StEt 47 2.19722457733622 norm_Gdnf -3.84801
StEt 47 2.19722457733622 norm_Yes1 -2.6487
StEt 47 2.19722457733622 norm_Il17a -0.45313
StEt 47 2.19722457733622 norm_Foxo1 -3.02212
StEt 47 2.19722457733622 norm_Tnfrsf11b 1.12246
StEt 47 2.19722457733622 norm_Tgfb1 -0.24051
StEt 47 2.19722457733622 norm_Pla2g4a 0.64802
StEt 47 2.19722457733622 norm_Il6 -3.37061
StEt 47 2.19722457733622 norm_Prdx5 -1.53517
StEt 47 2.19722457733622 norm_Tgfa 2.29698
StEt 47 2.19722457733622 norm_Ccl5 -2.59534
StEt 47 2.19722457733622 norm_Epo -1.15279
StEt 47 2.19722457733622 norm_Axin1 -2.46373
StEt 47 2.19722457733622 norm_Fst 4.95913
StEt 47 2.19722457733622 norm_Rgma -0.84981
StEt 47 2.19722457733622 norm_Nadk 2.16356
StEt 47 2.19722457733622 norm_Tnni3 5.87172
StEt 47 2.19722457733622 norm_Notch3 1.89313
StEt 47 2.19722457733622 norm_Snap29 0.86438
StEt 47 2.19722457733622 norm_Cntn1 5.80782
StEt 47 2.19722457733622 norm_Clstn2 -0.98361
StEt 47 2.19722457733622 norm_S100a4 6.82689
StEt 47 2.19722457733622 norm_Ca13 -3.36514
StEt 47 2.19722457733622 norm_Mia 0.85284
StEt 47 2.19722457733622 norm_Cant1 -1.40505
StEt 47 2.19722457733622 norm_Kitlg -4.10044
StEt 47 2.19722457733622 norm_Gfra1 -0.7508
StEt 47 2.19722457733622 norm_Ppp1r2 -4.00398
StEt 47 2.19722457733622 norm_Adam23 -0.52167
StEt 47 2.19722457733622 norm_Itgb6 -2.17482
StEt 47 2.19722457733622 norm_Cyr61 2.96118
StEt 47 2.19722457733622 norm_Dlk1 1.04476
StEt 47 2.19722457733622 norm_Ahr -2.98315
StEt 47 2.19722457733622 norm_Ccl2 4.11823
StEt 47 2.19722457733622 norm_Eno2 -0.01349
StEt 47 2.19722457733622 norm_Plin1 -2.23932

StEt 47 2.19722457733622 norm_Wfikkn2 1.32492
StEt 47 2.19722457733622 norm_Flrt2 0.12925
StEt 47 2.19722457733622 norm_Qdpr -0.98166
StEt 47 2.19722457733622 norm_Fas 1.34834
StEt 47 2.19722457733622 norm_Erbb4 -0.51328
StEt 47 2.19722457733622 norm_Riox2 -1.76159
StEt 47 2.19722457733622 norm_Plxna4 0.9801
StEt 47 2.19722457733622 norm_Epcam -0.39644
StEt 47 2.19722457733622 norm_Ccl3 -2.0881
StEt 47 2.19722457733622 norm_Crim1 -1.70145
StEt 47 2.19722457733622 norm_Vsig2 2.04063
StEt 47 2.19722457733622 norm_Hgf 0.03078
StEt 47 2.19722457733622 norm_Sez6l2 3.05634
StEt 47 2.19722457733622 norm_Il1a -2.48924
StEt 47 2.19722457733622 norm_Il23r 4.37808
StEt 47 2.19722457733622 norm_Dll1 1.03638
StEt 47 2.19722457733622 norm_Ddah1 -3.43821
StEt 47 2.19722457733622 norm_Il10 -2.45184
StEt 47 2.19722457733622 norm_Tnfrsf12a 2.47664
StEt 47 2.19722457733622 norm_Acvrl1 0.10756
StEt 47 2.19722457733622 norm_Lgmn 1.48588
StEt 47 2.19722457733622 norm_Csf2 -2.33425
StEt 47 2.19722457733622 norm_Cxcl9 2.77185
StEt 47 2.19722457733622 norm_Map2k6 -0.57338
StEt 47 2.19722457733622 norm_Itgb1bp2 -4.42206
StEt 47 2.19722457733622 norm_Il17f -4.64126
StEt 47 2.19722457733622 norm_Il1b -3.15553
StEt 47 2.19722457733622 norm_Casp3 1.10007
StEt 47 2.19722457733622 norm_Apbb1ip -3.78623
StEt 47 2.19722457733622 norm_Wisp1 0.83923
StEt 47 2.19722457733622 norm_Cdh6 1.27263
StEt 47 2.19722457733622 norm_Pdgfb 2.98236
StEt 47 2.19722457733622 norm_Igsf3 0.00578
StEt 47 2.19722457733622 norm_Tgfbr3 0.87416
StEt 47 2.19722457733622 norm_Cxcl1 3.1962
StEt 47 2.19722457733622 norm_Pak4 -3.62236
StEt 47 2.19722457733622 norm_Cntn4 2.97363
StEt 47 2.19722457733622 norm_Ghrl 3.39761
StEt 47 2.19722457733622 norm_Lpl 0.48376
StEt 47 2.19722457733622 norm_Fstl3 1.9659
StEt 47 2.19722457733622 norm_Dctn2 -2.53397
StEt 47 2.19722457733622 norm_Il5 -3.4977
StEt 47 2.19722457733622 norm_Eda2r 0.10738
StEt 47 2.19722457733622 norm_Ntf3 -1.20448
StEt 47 2.19722457733622 norm_Tnfsf12 0.33331
StEt 47 2.19722457733622 norm_Ccl20 4.72585
StEt 47 2.19722457733622 norm_Fli1 -4.77978
StEt 47 2.19722457733622 norm_Tpp1 3.99385
StEt 47 2.19722457733622 norm_Tnr -0.49501
StEt 47 2.19722457733622 norm_Vegfd -0.01465
StEt 47 2.19722457733622 norm_Parp1 -0.40545
StEt 47 2.19722457733622 norm_Tnf -2.7974
StEt 48 4.26267987704132 norm_Clmp 1.79434
StEt 48 4.26267987704132 norm_Matn2 0.53696
StEt 48 4.26267987704132 norm_Cpe 1.47101
StEt 48 4.26267987704132 norm_Gcg 0.2096
StEt 48 4.26267987704132 norm_Gdnf -3.667
StEt 48 4.26267987704132 norm_Yes1 0.88547
StEt 48 4.26267987704132 norm_Il17a -2.51812
StEt 48 4.26267987704132 norm_Foxo1 -1.45565
StEt 48 4.26267987704132 norm_Tnfrsf11b -0.36023
StEt 48 4.26267987704132 norm_Tgfb1 0.67982
StEt 48 4.26267987704132 norm_Pla2g4a 2.55917
StEt 48 4.26267987704132 norm_Il6 -3.80629
StEt 48 4.26267987704132 norm_Prdx5 1.23397
StEt 48 4.26267987704132 norm_Tgfa 2.98986
StEt 48 4.26267987704132 norm_Ccl5 -3.26315
StEt 48 4.26267987704132 norm_Epo -2.86741
StEt 48 4.26267987704132 norm_Axin1 -0.43924
StEt 48 4.26267987704132 norm_Fst 4.58327
StEt 48 4.26267987704132 norm_Rgma -2.02846
StEt 48 4.26267987704132 norm_Nadk 4.80053
StEt 48 4.26267987704132 norm_Tnni3 4.9852
StEt 48 4.26267987704132 norm_Notch3 1.19927
StEt 48 4.26267987704132 norm_Snap29 3.92366
StEt 48 4.26267987704132 norm_Cntn1 4.74406
StEt 48 4.26267987704132 norm_Clstn2 -2.00368
StEt 48 4.26267987704132 norm_S100a4 5.58728
StEt 48 4.26267987704132 norm_Ca13 -2.34355
StEt 48 4.26267987704132 norm_Mia -0.36412
StEt 48 4.26267987704132 norm_Cant1 -1.9257
StEt 48 4.26267987704132 norm_Kitlg -4.85344
StEt 48 4.26267987704132 norm_Gfra1 -0.16929
StEt 48 4.26267987704132 norm_Ppp1r2 -1.25256
StEt 48 4.26267987704132 norm_Adam23 -2.17406
StEt 48 4.26267987704132 norm_Itgb6 -3.70853
StEt 48 4.26267987704132 norm_Cyr61 2.1714
StEt 48 4.26267987704132 norm_Dlk1 -0.59225
StEt 48 4.26267987704132 norm_Ahr -1.72056
StEt 48 4.26267987704132 norm_Ccl2 3.51572
StEt 48 4.26267987704132 norm_Eno2 -0.63165
StEt 48 4.26267987704132 norm_Plin1 -0.7477
StEt 48 4.26267987704132 norm_Wfikkn2 0.1991
StEt 48 4.26267987704132 norm_Flrt2 0.92997
StEt 48 4.26267987704132 norm_Qdpr 1.60953

StEt 48	4.26267987704132	norm_Fas	1.50891
StEt 48	4.26267987704132	norm_Erbb4	-1.12254
StEt 48	4.26267987704132	norm_Riox2	2.0739
StEt 48	4.26267987704132	norm_Plxna4	0.06168
StEt 48	4.26267987704132	norm_Epcam	-2.37122
StEt 48	4.26267987704132	norm_Ccl3	-1.98855
StEt 48	4.26267987704132	norm_Crim1	0.29388
StEt 48	4.26267987704132	norm_Vsig2	0.17548
StEt 48	4.26267987704132	norm_Hgf	2.55713
StEt 48	4.26267987704132	norm_Sez6l2	1.78585
StEt 48	4.26267987704132	norm_Il1a	0.80045
StEt 48	4.26267987704132	norm_Il23r	3.07363
StEt 48	4.26267987704132	norm_Dll1	-0.28514
StEt 48	4.26267987704132	norm_Ddah1	0.22076
StEt 48	4.26267987704132	norm_Il10	-3.46243
StEt 48	4.26267987704132	norm_Tnfrsf12a	1.62268
StEt 48	4.26267987704132	norm_Acvrl1	-0.39846
StEt 48	4.26267987704132	norm_Lgmn	0.93943
StEt 48	4.26267987704132	norm_Csf2	-3.6786
StEt 48	4.26267987704132	norm_Cxcl9	2.76807
StEt 48	4.26267987704132	norm_Map2k6	2.31847
StEt 48	4.26267987704132	norm_Itgb1bp2	-5.40062
StEt 48	4.26267987704132	norm_Il17f	-5.48403
StEt 48	4.26267987704132	norm_Il1b	-3.34014
StEt 48	4.26267987704132	norm_Casp3	4.09171
StEt 48	4.26267987704132	norm_Apbb1ip	-3.79188
StEt 48	4.26267987704132	norm_Wisp1	-0.03948
StEt 48	4.26267987704132	norm_Cdh6	0.33026
StEt 48	4.26267987704132	norm_Pdgfb	6.12004
StEt 48	4.26267987704132	norm_Igsf3	-0.02958
StEt 48	4.26267987704132	norm_Tgfbr3	-0.38503
StEt 48	4.26267987704132	norm_Cxcl1	2.53685
StEt 48	4.26267987704132	norm_Pak4	-2.73388
StEt 48	4.26267987704132	norm_Cntn4	1.85592
StEt 48	4.26267987704132	norm_Ghrl	-1.12048
StEt 48	4.26267987704132	norm_Lpl	-0.55639
StEt 48	4.26267987704132	norm_Fstl3	0.90435
StEt 48	4.26267987704132	norm_Dctn2	-0.2204
StEt 48	4.26267987704132	norm_Il5	-4.49274
StEt 48	4.26267987704132	norm_Eda2r	-0.35338
StEt 48	4.26267987704132	norm_Ntf3	-2.74679
StEt 48	4.26267987704132	norm_Tnfsf12	0.73182
StEt 48	4.26267987704132	norm_Ccl20	3.76947
StEt 48	4.26267987704132	norm_Fli1	-3.95542
StEt 48	4.26267987704132	norm_Tpp1	2.70079
StEt 48	4.26267987704132	norm_Tnr	-2.03518
StEt 48	4.26267987704132	norm_Vegfd	-1.29053
StEt 48	4.26267987704132	norm_Parp1	4.06838
StEt 48	4.26267987704132	norm_Tnf	-3.7437
StEt 49	4.39444915467244	norm_Clmp	2.91566
StEt 49	4.39444915467244	norm_Matn2	1.25587
StEt 49	4.39444915467244	norm_Cpe	2.13127
StEt 49	4.39444915467244	norm_Gcg	0.11551
StEt 49	4.39444915467244	norm_Gdnf	-3.5569
StEt 49	4.39444915467244	norm_Yes1	-2.27145
StEt 49	4.39444915467244	norm_Il17a	-2.4382
StEt 49	4.39444915467244	norm_Foxo1	-2.45861
StEt 49	4.39444915467244	norm_Tnfrsf11b	0.24446
StEt 49	4.39444915467244	norm_Tgfb1	-0.06738
StEt 49	4.39444915467244	norm_Pla2g4a	1.93124
StEt 49	4.39444915467244	norm_Il6	-3.49751
StEt 49	4.39444915467244	norm_Prdx5	-1.45447
StEt 49	4.39444915467244	norm_Tgfa	1.29372
StEt 49	4.39444915467244	norm_Ccl5	-2.43003
StEt 49	4.39444915467244	norm_Epo	-1.34774
StEt 49	4.39444915467244	norm_Axin1	-1.82399
StEt 49	4.39444915467244	norm_Fst	5.06154
StEt 49	4.39444915467244	norm_Rgma	-0.77293
StEt 49	4.39444915467244	norm_Nadk	3.14392
StEt 49	4.39444915467244	norm_Tnni3	10.30219
StEt 49	4.39444915467244	norm_Notch3	1.94401
StEt 49	4.39444915467244	norm_Snap29	2.287
StEt 49	4.39444915467244	norm_Cntn1	5.43689
StEt 49	4.39444915467244	norm_Clstn2	-2.74261
StEt 49	4.39444915467244	norm_S100a4	6.55145
StEt 49	4.39444915467244	norm_Ca13	-3.36847
StEt 49	4.39444915467244	norm_Mia	0.63965
StEt 49	4.39444915467244	norm_Cant1	-1.61912
StEt 49	4.39444915467244	norm_Kitlg	-4.25801
StEt 49	4.39444915467244	norm_Gfra1	-1.00438
StEt 49	4.39444915467244	norm_Ppp1r2	-2.76978
StEt 49	4.39444915467244	norm_Adam23	-0.93648
StEt 49	4.39444915467244	norm_Itgb6	-3.05908
StEt 49	4.39444915467244	norm_Cyr61	3.42762
StEt 49	4.39444915467244	norm_Dlk1	0.64511
StEt 49	4.39444915467244	norm_Ahr	-2.96374
StEt 49	4.39444915467244	norm_Ccl2	3.80364
StEt 49	4.39444915467244	norm_Eno2	-0.05658
StEt 49	4.39444915467244	norm_Plin1	-2.5896
StEt 49	4.39444915467244	norm_Wfikkn2	1.34608
StEt 49	4.39444915467244	norm_Flrt2	-0.08323
StEt 49	4.39444915467244	norm_Qdpr	-0.26341
StEt 49	4.39444915467244	norm_Fas	1.50333
StEt 49	4.39444915467244	norm_Erbb4	-0.33453
StEt 49	4.39444915467244	norm_Riox2	-0.22013

StEt 49 4.39444915467244 norm_Plxna4 1.72398
StEt 49 4.39444915467244 norm_Epcam -1.21174
StEt 49 4.39444915467244 norm_Ccl3 -2.04178
StEt 49 4.39444915467244 norm_Crim1 -1.49062
StEt 49 4.39444915467244 norm_Vsig2 1.69799
StEt 49 4.39444915467244 norm_Hgf 0.50013
StEt 49 4.39444915467244 norm_Sez6l2 3.33867
StEt 49 4.39444915467244 norm_Il1a -0.48062
StEt 49 4.39444915467244 norm_Il23r 4.14396
StEt 49 4.39444915467244 norm_Dll1 0.66664
StEt 49 4.39444915467244 norm_Ddah1 -2.7049
StEt 49 4.39444915467244 norm_Il10 -2.76473
StEt 49 4.39444915467244 norm_Tnfrsf12a 1.77037
StEt 49 4.39444915467244 norm_Acvrl1 -0.45884
StEt 49 4.39444915467244 norm_Lgmn 1.42631
StEt 49 4.39444915467244 norm_Csf2 -2.50721
StEt 49 4.39444915467244 norm_Cxcl9 2.93378
StEt 49 4.39444915467244 norm_Map2k6 0.58571
StEt 49 4.39444915467244 norm_Itgb1bp2 -4.71151
StEt 49 4.39444915467244 norm_Il17f -4.87712
StEt 49 4.39444915467244 norm_Il1b -3.37461
StEt 49 4.39444915467244 norm_Casp3 1.60536
StEt 49 4.39444915467244 norm_Apbb1ip -4.44007
StEt 49 4.39444915467244 norm_Wisp1 0.56154
StEt 49 4.39444915467244 norm_Cdh6 0.68552
StEt 49 4.39444915467244 norm_Pdgfb 5.27545
StEt 49 4.39444915467244 norm_Igsf3 -0.27885
StEt 49 4.39444915467244 norm_Tgfbr3 0.29856
StEt 49 4.39444915467244 norm_Cxcl1 2.35525
StEt 49 4.39444915467244 norm_Pak4 -3.81558
StEt 49 4.39444915467244 norm_Cntn4 1.9935
StEt 49 4.39444915467244 norm_Ghrl 2.64865
StEt 49 4.39444915467244 norm_Lpl 0.19096
StEt 49 4.39444915467244 norm_Fstl3 1.44374
StEt 49 4.39444915467244 norm_Dctn2 -2.44714
StEt 49 4.39444915467244 norm_Il5 -5.04212
StEt 49 4.39444915467244 norm_Eda2r -0.29833
StEt 49 4.39444915467244 norm_Ntf3 -1.74118
StEt 49 4.39444915467244 norm_Tnfsf12 0.55753
StEt 49 4.39444915467244 norm_Ccl20 3.94707
StEt 49 4.39444915467244 norm_Fli1 -4.72007
StEt 49 4.39444915467244 norm_Tpp1 3.70861
StEt 49 4.39444915467244 norm_Tnr -0.84343
StEt 49 4.39444915467244 norm_Vegfd -0.31348
StEt 49 4.39444915467244 norm_Parp1 -0.20489
StEt 49 4.39444915467244 norm_Tnf -0.88249
StEt 52 3.25809653802148 norm_Clmp 1.76508
StEt 52 3.25809653802148 norm_Matn2 0.45004
StEt 52 3.25809653802148 norm_Cpe 1.20846
StEt 52 3.25809653802148 norm_Gcg -0.23646
StEt 52 3.25809653802148 norm_Gdnf -3.8006
StEt 52 3.25809653802148 norm_Yes1 -0.27718
StEt 52 3.25809653802148 norm_Il17a -2.10555
StEt 52 3.25809653802148 norm_Foxo1 -1.95637
StEt 52 3.25809653802148 norm_Tnfrsf11b -0.37458
StEt 52 3.25809653802148 norm_Tgfb1 1.00806
StEt 52 3.25809653802148 norm_Pla2g4a 1.99186
StEt 52 3.25809653802148 norm_Il6 -5.05347
StEt 52 3.25809653802148 norm_Prdx5 0.41388
StEt 52 3.25809653802148 norm_Tgfa 1.62011
StEt 52 3.25809653802148 norm_Ccl5 -2.41882
StEt 52 3.25809653802148 norm_Epo -2.508
StEt 52 3.25809653802148 norm_Axin1 -0.73822
StEt 52 3.25809653802148 norm_Fst 4.55991
StEt 52 3.25809653802148 norm_Rgma -1.69014
StEt 52 3.25809653802148 norm_Nadk 4.36827
StEt 52 3.25809653802148 norm_Tnni3 7.71071
StEt 52 3.25809653802148 norm_Notch3 1.16521
StEt 52 3.25809653802148 norm_Snap29 3.37214
StEt 52 3.25809653802148 norm_Cntn1 4.65381
StEt 52 3.25809653802148 norm_Clstn2 -1.96808
StEt 52 3.25809653802148 norm_S100a4 5.8153
StEt 52 3.25809653802148 norm_Ca13 -2.69565
StEt 52 3.25809653802148 norm_Mia -0.45306
StEt 52 3.25809653802148 norm_Cant1 -1.61834
StEt 52 3.25809653802148 norm_Kitlg -5.01963
StEt 52 3.25809653802148 norm_Gfra1 -1.27117
StEt 52 3.25809653802148 norm_Ppp1r2 -1.72519
StEt 52 3.25809653802148 norm_Adam23 -1.84815
StEt 52 3.25809653802148 norm_Itgb6 -3.28229
StEt 52 3.25809653802148 norm_Cyr61 2.41855
StEt 52 3.25809653802148 norm_Dlk1 -0.21408
StEt 52 3.25809653802148 norm_Ahr -2.0417
StEt 52 3.25809653802148 norm_Ccl2 5.04209
StEt 52 3.25809653802148 norm_Eno2 -0.8912
StEt 52 3.25809653802148 norm_Plin1 -0.90659
StEt 52 3.25809653802148 norm_Wfikkn2 0.82957
StEt 52 3.25809653802148 norm_Flrt2 0.2276
StEt 52 3.25809653802148 norm_Qdpr 1.45546
StEt 52 3.25809653802148 norm_Fas 1.55149
StEt 52 3.25809653802148 norm_Erbb4 -1.17964
StEt 52 3.25809653802148 norm_Riox2 1.74494
StEt 52 3.25809653802148 norm_Plxna4 -0.0057
StEt 52 3.25809653802148 norm_Epcam -2.47907
StEt 52 3.25809653802148 norm_Ccl3 -2.37183

StEt 52 3.25809653802148 norm_Crim1 0.46822
StEt 52 3.25809653802148 norm_Vsig2 0.53638
StEt 52 3.25809653802148 norm_Hgf 2.63786
StEt 52 3.25809653802148 norm_Sez6l2 1.9663
StEt 52 3.25809653802148 norm_Il1a 0.33345
StEt 52 3.25809653802148 norm_Il23r 2.8244
StEt 52 3.25809653802148 norm_Dll1 0.16547
StEt 52 3.25809653802148 norm_Ddah1 -0.14593
StEt 52 3.25809653802148 norm_Il10 -3.24025
StEt 52 3.25809653802148 norm_Tnfrsf12a 1.10081
StEt 52 3.25809653802148 norm_Acvrl1 -0.72891
StEt 52 3.25809653802148 norm_Lgmn 1.00255
StEt 52 3.25809653802148 norm_Csf2 -2.11852
StEt 52 3.25809653802148 norm_Cxcl9 4.40914
StEt 52 3.25809653802148 norm_Map2k6 2.03634
StEt 52 3.25809653802148 norm_Itgb1bp2 -5.03467
StEt 52 3.25809653802148 norm_Il17f -5.68835
StEt 52 3.25809653802148 norm_Il1b -3.59367
StEt 52 3.25809653802148 norm_Casp3 3.80551
StEt 52 3.25809653802148 norm_Apbb1ip -3.39849
StEt 52 3.25809653802148 norm_Wisp1 0.13065
StEt 52 3.25809653802148 norm_Cdh6 0.41675
StEt 52 3.25809653802148 norm_Pdgfb 6.06567
StEt 52 3.25809653802148 norm_Igsf3 -0.03941
StEt 52 3.25809653802148 norm_Tgfbr3 -0.54664
StEt 52 3.25809653802148 norm_Cxcl1 2.87237
StEt 52 3.25809653802148 norm_Pak4 -3.01115
StEt 52 3.25809653802148 norm_Cntn4 1.55897
StEt 52 3.25809653802148 norm_Ghrl 0.69766
StEt 52 3.25809653802148 norm_Lpl -0.01962
StEt 52 3.25809653802148 norm_Fstl3 0.89358
StEt 52 3.25809653802148 norm_Dctn2 -0.98878
StEt 52 3.25809653802148 norm_Il5 -4.03565
StEt 52 3.25809653802148 norm_Eda2r -0.71274
StEt 52 3.25809653802148 norm_Ntf3 -2.56861
StEt 52 3.25809653802148 norm_Tnfsf12 0.48242
StEt 52 3.25809653802148 norm_Ccl20 3.87029
StEt 52 3.25809653802148 norm_Fli1 -3.31572
StEt 52 3.25809653802148 norm_Tpp1 2.7367
StEt 52 3.25809653802148 norm_Tnr -1.41485
StEt 52 3.25809653802148 norm_Vegfd -1.38131
StEt 52 3.25809653802148 norm_Parp1 2.04612
StEt 52 3.25809653802148 norm_Tnf -3.31629
StEt 53 3.09104245335832 norm_Clmp 2.52278
StEt 53 3.09104245335832 norm_Matn2 1.37939
StEt 53 3.09104245335832 norm_Cpe 2.24077
StEt 53 3.09104245335832 norm_Gcg 1.79452
StEt 53 3.09104245335832 norm_Gdnf -3.40519
StEt 53 3.09104245335832 norm_Yes1 -2.60169
StEt 53 3.09104245335832 norm_Il17a -0.77637
StEt 53 3.09104245335832 norm_Foxo1 -2.89484
StEt 53 3.09104245335832 norm_Tnfrsf11b 0.71785
StEt 53 3.09104245335832 norm_Tgfb1 -0.0028
StEt 53 3.09104245335832 norm_Pla2g4a 0.93875
StEt 53 3.09104245335832 norm_Il6 -3.77748
StEt 53 3.09104245335832 norm_Prdx5 -2.22443
StEt 53 3.09104245335832 norm_Tgfa 1.92373
StEt 53 3.09104245335832 norm_Ccl5 -1.98647
StEt 53 3.09104245335832 norm_Epo -1.25831
StEt 53 3.09104245335832 norm_Axin1 -1.95133
StEt 53 3.09104245335832 norm_Fst 4.90558
StEt 53 3.09104245335832 norm_Rgma -1.30693
StEt 53 3.09104245335832 norm_Nadk 3.08015
StEt 53 3.09104245335832 norm_Tnni3 4.71772
StEt 53 3.09104245335832 norm_Notch3 2.02244
StEt 53 3.09104245335832 norm_Snap29 1.70312
StEt 53 3.09104245335832 norm_Cntn1 5.72949
StEt 53 3.09104245335832 norm_Clstn2 -1.0049
StEt 53 3.09104245335832 norm_S100a4 6.23529
StEt 53 3.09104245335832 norm_Ca13 -3.78817
StEt 53 3.09104245335832 norm_Mia 0.84559
StEt 53 3.09104245335832 norm_Cant1 -1.19382
StEt 53 3.09104245335832 norm_Kitlg -4.26028
StEt 53 3.09104245335832 norm_Gfra1 -1.36912
StEt 53 3.09104245335832 norm_Ppp1r2 -3.63868
StEt 53 3.09104245335832 norm_Adam23 -0.39676
StEt 53 3.09104245335832 norm_Itgb6 -2.79133
StEt 53 3.09104245335832 norm_Cyr61 3.21344
StEt 53 3.09104245335832 norm_Dlk1 1.17795
StEt 53 3.09104245335832 norm_Ahr -2.88118
StEt 53 3.09104245335832 norm_Ccl2 4.80141
StEt 53 3.09104245335832 norm_Eno2 -1.01818
StEt 53 3.09104245335832 norm_Plin1 -2.43204
StEt 53 3.09104245335832 norm_Wfikkn2 1.70143
StEt 53 3.09104245335832 norm_Flrt2 0.02369
StEt 53 3.09104245335832 norm_Qdpr 0.15835
StEt 53 3.09104245335832 norm_Fas 1.32622
StEt 53 3.09104245335832 norm_Erbb4 -0.37916
StEt 53 3.09104245335832 norm_Riox2 -0.26496
StEt 53 3.09104245335832 norm_Plxna4 -0.31164
StEt 53 3.09104245335832 norm_Epcam -1.36748
StEt 53 3.09104245335832 norm_Ccl3 -2.38509
StEt 53 3.09104245335832 norm_Crim1 -1.60925
StEt 53 3.09104245335832 norm_Vsig2 2.18022
StEt 53 3.09104245335832 norm_Hgf 0.58914

StEt 53	3.09104245335832	norm_Sez6l2	2.75035
StEt 53	3.09104245335832	norm_Il1a	-0.73021
StEt 53	3.09104245335832	norm_Il23r	3.52676
StEt 53	3.09104245335832	norm_Dll1	0.85997
StEt 53	3.09104245335832	norm_Ddah1	-2.68101
StEt 53	3.09104245335832	norm_Il10	-2.53964
StEt 53	3.09104245335832	norm_Tnfrsf12a	1.5758
StEt 53	3.09104245335832	norm_Acvrl1	-0.15248
StEt 53	3.09104245335832	norm_Lgmn	1.59594
StEt 53	3.09104245335832	norm_Csf2	-2.69101
StEt 53	3.09104245335832	norm_Cxcl9	3.0702
StEt 53	3.09104245335832	norm_Map2k6	-0.28896
StEt 53	3.09104245335832	norm_Itgb1bp2	-4.59925
StEt 53	3.09104245335832	norm_Il17f	-4.89184
StEt 53	3.09104245335832	norm_Il1b	-3.04882
StEt 53	3.09104245335832	norm_Casp3	1.71036
StEt 53	3.09104245335832	norm_Apbb1ip	-4.44901
StEt 53	3.09104245335832	norm_Wisp1	0.59653
StEt 53	3.09104245335832	norm_Cdh6	1.25366
StEt 53	3.09104245335832	norm_Pdgfb	5.20637
StEt 53	3.09104245335832	norm_Igsf3	-0.59394
StEt 53	3.09104245335832	norm_Tgfbr3	0.4193
StEt 53	3.09104245335832	norm_Cxcl1	2.81519
StEt 53	3.09104245335832	norm_Pak4	-2.85623
StEt 53	3.09104245335832	norm_Cntn4	2.13571
StEt 53	3.09104245335832	norm_Ghrl	2.85829
StEt 53	3.09104245335832	norm_Lpl	0.72696
StEt 53	3.09104245335832	norm_Fstl3	1.51806
StEt 53	3.09104245335832	norm_Dctn2	-2.57938
StEt 53	3.09104245335832	norm_Il5	-2.60146
StEt 53	3.09104245335832	norm_Eda2r	0.16698
StEt 53	3.09104245335832	norm_Ntf3	-1.7232
StEt 53	3.09104245335832	norm_Tnfsf12	0.52481
StEt 53	3.09104245335832	norm_Ccl20	4.84868
StEt 53	3.09104245335832	norm_Fli1	-4.43617
StEt 53	3.09104245335832	norm_Tpp1	3.87966
StEt 53	3.09104245335832	norm_Tnr	-0.64974
StEt 53	3.09104245335832	norm_Vegfd	-0.65857
StEt 53	3.09104245335832	norm_Parp1	0.26014
StEt 53	3.09104245335832	norm_Tnf	-2.78014
StEt 54	2.56494935746154	norm_Clmp	2.06553
StEt 54	2.56494935746154	norm_Matn2	1.79978
StEt 54	2.56494935746154	norm_Cpe	1.15036
StEt 54	2.56494935746154	norm_Gcg	1.24735
StEt 54	2.56494935746154	norm_Gdnf	-3.98034
StEt 54	2.56494935746154	norm_Yes1	-1.9532
StEt 54	2.56494935746154	norm_Il17a	-0.72737
StEt 54	2.56494935746154	norm_Foxo1	-2.10511
StEt 54	2.56494935746154	norm_Tnfrsf11b	1.42498
StEt 54	2.56494935746154	norm_Tgfb1	-0.31729
StEt 54	2.56494935746154	norm_Pla2g4a	1.82721
StEt 54	2.56494935746154	norm_Il6	-1.92337
StEt 54	2.56494935746154	norm_Prdx5	-0.92584
StEt 54	2.56494935746154	norm_Tgfa	1.03242
StEt 54	2.56494935746154	norm_Ccl5	-2.03404
StEt 54	2.56494935746154	norm_Epo	-1.53297
StEt 54	2.56494935746154	norm_Axin1	-1.25844
StEt 54	2.56494935746154	norm_Fst	4.81369
StEt 54	2.56494935746154	norm_Rgma	-1.31547
StEt 54	2.56494935746154	norm_Nadk	4.55207
StEt 54	2.56494935746154	norm_Tnni3	7.39406
StEt 54	2.56494935746154	norm_Notch3	1.24098
StEt 54	2.56494935746154	norm_Snap29	3.1518
StEt 54	2.56494935746154	norm_Cntn1	4.79962
StEt 54	2.56494935746154	norm_Clstn2	-1.66529
StEt 54	2.56494935746154	norm_S100a4	5.56818
StEt 54	2.56494935746154	norm_Ca13	-2.86957
StEt 54	2.56494935746154	norm_Mia	0.25011
StEt 54	2.56494935746154	norm_Cant1	-1.80529
StEt 54	2.56494935746154	norm_Kitlg	-4.63139
StEt 54	2.56494935746154	norm_Gfra1	-0.66416
StEt 54	2.56494935746154	norm_Ppp1r2	-2.56251
StEt 54	2.56494935746154	norm_Adam23	-0.94037
StEt 54	2.56494935746154	norm_Itgb6	-3.74926
StEt 54	2.56494935746154	norm_Cyr61	2.68125
StEt 54	2.56494935746154	norm_Dlk1	0.47467
StEt 54	2.56494935746154	norm_Ahr	-2.72432
StEt 54	2.56494935746154	norm_Ccl2	4.69343
StEt 54	2.56494935746154	norm_Eno2	-0.92488
StEt 54	2.56494935746154	norm_Plin1	-0.09507
StEt 54	2.56494935746154	norm_Wfikkn2	1.49253
StEt 54	2.56494935746154	norm_Flrt2	-0.31933
StEt 54	2.56494935746154	norm_Qdpr	0.74892
StEt 54	2.56494935746154	norm_Fas	1.50127
StEt 54	2.56494935746154	norm_Erbb4	-1.05694
StEt 54	2.56494935746154	norm_Riox2	0.01799
StEt 54	2.56494935746154	norm_Plxna4	-0.29696
StEt 54	2.56494935746154	norm_Epcam	-2.09453
StEt 54	2.56494935746154	norm_Ccl3	-1.77125
StEt 54	2.56494935746154	norm_Crim1	-1.53666
StEt 54	2.56494935746154	norm_Vsig2	0.7534
StEt 54	2.56494935746154	norm_Hgf	0.3624
StEt 54	2.56494935746154	norm_Sez6l2	2.26353
StEt 54	2.56494935746154	norm_Il1a	-0.7373
StEt 54	2.56494935746154	norm_Il23r	4.03919

StEt 54 2.56494935746154 norm_Dll1 0.79344
StEt 54 2.56494935746154 norm_Ddah1 -1.40129
StEt 54 2.56494935746154 norm_Il10 -1.98795
StEt 54 2.56494935746154 norm_Tnfrsf12a 1.12417
StEt 54 2.56494935746154 norm_Acvrl1 -0.24652
StEt 54 2.56494935746154 norm_Lgmn 1.33763
StEt 54 2.56494935746154 norm_Csf2 -2.42481
StEt 54 2.56494935746154 norm_Cxcl9 5.01227
StEt 54 2.56494935746154 norm_Map2k6 0.20278
StEt 54 2.56494935746154 norm_Itgb1bp2 -5.09691
StEt 54 2.56494935746154 norm_Il17f -5.08591
StEt 54 2.56494935746154 norm_Il1b -2.93705
StEt 54 2.56494935746154 norm_Casp3 2.89267
StEt 54 2.56494935746154 norm_Apbb1ip -3.55112
StEt 54 2.56494935746154 norm_Wisp1 0.0337
StEt 54 2.56494935746154 norm_Cdh6 0.92048
StEt 54 2.56494935746154 norm_Pdgfb 4.35285
StEt 54 2.56494935746154 norm_Igsf3 -0.62066
StEt 54 2.56494935746154 norm_Tgfbr3 0.00309
StEt 54 2.56494935746154 norm_Cxcl1 3.76707
StEt 54 2.56494935746154 norm_Pak4 -3.7725
StEt 54 2.56494935746154 norm_Cntn4 1.44693
StEt 54 2.56494935746154 norm_Ghrl 1.59968
StEt 54 2.56494935746154 norm_Lpl -0.23959
StEt 54 2.56494935746154 norm_Fstl3 0.89006
StEt 54 2.56494935746154 norm_Dctn2 -2.04012
StEt 54 2.56494935746154 norm_Il5 -4.39342
StEt 54 2.56494935746154 norm_Eda2r -0.29813
StEt 54 2.56494935746154 norm_Ntf3 -2.07155
StEt 54 2.56494935746154 norm_Tnfsf12 0.24115
StEt 54 2.56494935746154 norm_Ccl20 3.97905
StEt 54 2.56494935746154 norm_Fli1 -4.46791
StEt 54 2.56494935746154 norm_Tpp1 3.04282
StEt 54 2.56494935746154 norm_Tnr -1.00078
StEt 54 2.56494935746154 norm_Vegfd -1.02871
StEt 54 2.56494935746154 norm_Parp1 0.89259
StEt 54 2.56494935746154 norm_Tnf -2.69557
StEt 55 2.70805020110221 norm_Clmp 2.9459
StEt 55 2.70805020110221 norm_Matn2 1.00621
StEt 55 2.70805020110221 norm_Cpe 2.24225
StEt 55 2.70805020110221 norm_Gcg 1.3829
StEt 55 2.70805020110221 norm_Gdnf -3.4052
StEt 55 2.70805020110221 norm_Yes1 -2.24066
StEt 55 2.70805020110221 norm_Il17a 0.29962
StEt 55 2.70805020110221 norm_Foxo1 -2.21657
StEt 55 2.70805020110221 norm_Tnfrsf11b 0.86253
StEt 55 2.70805020110221 norm_Tgfb1 -0.39861
StEt 55 2.70805020110221 norm_Pla2g4a 1.13628
StEt 55 2.70805020110221 norm_Il6 -3.5945
StEt 55 2.70805020110221 norm_Prdx5 -2.02905
StEt 55 2.70805020110221 norm_Tgfa 1.60079
StEt 55 2.70805020110221 norm_Ccl5 -2.17884
StEt 55 2.70805020110221 norm_Epo -0.44091
StEt 55 2.70805020110221 norm_Axin1 -2.19582
StEt 55 2.70805020110221 norm_Fst 5.22284
StEt 55 2.70805020110221 norm_Rgma -1.348
StEt 55 2.70805020110221 norm_Nadk 1.68867
StEt 55 2.70805020110221 norm_Tnni3 8.00744
StEt 55 2.70805020110221 norm_Notch3 1.87955
StEt 55 2.70805020110221 norm_Snap29 1.5394
StEt 55 2.70805020110221 norm_Cntn1 5.78306
StEt 55 2.70805020110221 norm_Clstn2 -0.69598
StEt 55 2.70805020110221 norm_S100a4 6.71622
StEt 55 2.70805020110221 norm_Ca13 -2.83309
StEt 55 2.70805020110221 norm_Mia 0.99481
StEt 55 2.70805020110221 norm_Cant1 -1.46958
StEt 55 2.70805020110221 norm_Kitlg -4.21175
StEt 55 2.70805020110221 norm_Gfra1 -0.93735
StEt 55 2.70805020110221 norm_Ppp1r2 -3.15676
StEt 55 2.70805020110221 norm_Adam23 -1.11654
StEt 55 2.70805020110221 norm_Itgb6 -2.66223
StEt 55 2.70805020110221 norm_Cyr61 3.1078
StEt 55 2.70805020110221 norm_Dlk1 0.94534
StEt 55 2.70805020110221 norm_Ahr -3.27299
StEt 55 2.70805020110221 norm_Ccl2 4.361
StEt 55 2.70805020110221 norm_Eno2 -0.29342
StEt 55 2.70805020110221 norm_Plin1 -2.65666
StEt 55 2.70805020110221 norm_Wfikkn2 2.65472
StEt 55 2.70805020110221 norm_Flrt2 0.01162
StEt 55 2.70805020110221 norm_Qdpr -1.52784
StEt 55 2.70805020110221 norm_Fas 1.2732
StEt 55 2.70805020110221 norm_Erbb4 -0.21404
StEt 55 2.70805020110221 norm_Riox2 -2.44005
StEt 55 2.70805020110221 norm_Plxna4 -1.14578
StEt 55 2.70805020110221 norm_Epcam -1.37719
StEt 55 2.70805020110221 norm_Ccl3 -2.06758
StEt 55 2.70805020110221 norm_Crim1 -1.81506
StEt 55 2.70805020110221 norm_Vsig2 1.5609
StEt 55 2.70805020110221 norm_Hgf -0.42714
StEt 55 2.70805020110221 norm_Sez6l2 3.1813
StEt 55 2.70805020110221 norm_Il1a -1.92235
StEt 55 2.70805020110221 norm_Il23r 4.44693
StEt 55 2.70805020110221 norm_Dll1 1.06681
StEt 55 2.70805020110221 norm_Ddah1 -3.4603
StEt 55 2.70805020110221 norm_Il10 -2.5431

StEt 55 2.70805020110221 norm_Tnfrsf12a 2.35677
StEt 55 2.70805020110221 norm_Acvrl1 0.44304
StEt 55 2.70805020110221 norm_Lgmn 1.68048
StEt 55 2.70805020110221 norm_Csf2 -2.2944
StEt 55 2.70805020110221 norm_Cxcl9 2.90208
StEt 55 2.70805020110221 norm_Map2k6 -0.21325
StEt 55 2.70805020110221 norm_Itgb1bp2 -4.84391
StEt 55 2.70805020110221 norm_Il17f -4.58385
StEt 55 2.70805020110221 norm_Il1b -3.34986
StEt 55 2.70805020110221 norm_Casp3 2.43767
StEt 55 2.70805020110221 norm_Apbb1ip -3.88788
StEt 55 2.70805020110221 norm_Wisp1 0.88312
StEt 55 2.70805020110221 norm_Cdh6 1.32055
StEt 55 2.70805020110221 norm_Pdgfb 2.58966
StEt 55 2.70805020110221 norm_Igsf3 -0.4328
StEt 55 2.70805020110221 norm_Tgfbr3 0.56819
StEt 55 2.70805020110221 norm_Cxcl1 2.34411
StEt 55 2.70805020110221 norm_Pak4 -3.1961
StEt 55 2.70805020110221 norm_Cntn4 2.32608
StEt 55 2.70805020110221 norm_Ghrl 2.47848
StEt 55 2.70805020110221 norm_Lpl 0.35488
StEt 55 2.70805020110221 norm_Fstl3 1.93291
StEt 55 2.70805020110221 norm_Dctn2 -2.27511
StEt 55 2.70805020110221 norm_Il5 -3.19363
StEt 55 2.70805020110221 norm_Eda2r 0.28358
StEt 55 2.70805020110221 norm_Ntf3 -1.25491
StEt 55 2.70805020110221 norm_Tnfsf12 0.43867
StEt 55 2.70805020110221 norm_Ccl20 4.69204
StEt 55 2.70805020110221 norm_Fli1 -4.43718
StEt 55 2.70805020110221 norm_Tpp1 3.9658
StEt 55 2.70805020110221 norm_Tnr -0.61917
StEt 55 2.70805020110221 norm_Vegfd -0.41643
StEt 55 2.70805020110221 norm_Parp1 0.15255
StEt 55 2.70805020110221 norm_Tnf -2.77574
StEt 56 4.48863636973214 norm_Clmp 2.06691
StEt 56 4.48863636973214 norm_Matn2 0.89313
StEt 56 4.48863636973214 norm_Cpe 1.63502
StEt 56 4.48863636973214 norm_Gcg 0.86766
StEt 56 4.48863636973214 norm_Gdnf -3.80641
StEt 56 4.48863636973214 norm_Yes1 -1.50183
StEt 56 4.48863636973214 norm_Il17a -0.57278
StEt 56 4.48863636973214 norm_Foxo1 -1.72117
StEt 56 4.48863636973214 norm_Tnfrsf11b 0.58876
StEt 56 4.48863636973214 norm_Tgfb1 0.59169
StEt 56 4.48863636973214 norm_Pla2g4a 2.09013
StEt 56 4.48863636973214 norm_Il6 -3.9805
StEt 56 4.48863636973214 norm_Prdx5 -1.03321
StEt 56 4.48863636973214 norm_Tgfa 1.24367
StEt 56 4.48863636973214 norm_Ccl5 -2.77211
StEt 56 4.48863636973214 norm_Epo -0.89474
StEt 56 4.48863636973214 norm_Axin1 -0.613
StEt 56 4.48863636973214 norm_Fst 4.73272
StEt 56 4.48863636973214 norm_Rgma -1.18478
StEt 56 4.48863636973214 norm_Nadk 4.13384
StEt 56 4.48863636973214 norm_Tnni3 9.95804
StEt 56 4.48863636973214 norm_Notch3 1.72162
StEt 56 4.48863636973214 norm_Snap29 3.34675
StEt 56 4.48863636973214 norm_Cntn1 5.28357
StEt 56 4.48863636973214 norm_Clstn2 -2.74028
StEt 56 4.48863636973214 norm_S100a4 5.62517
StEt 56 4.48863636973214 norm_Ca13 -2.9469
StEt 56 4.48863636973214 norm_Mia -0.05639
StEt 56 4.48863636973214 norm_Cant1 -1.95923
StEt 56 4.48863636973214 norm_Kitlg -4.87891
StEt 56 4.48863636973214 norm_Gfra1 -1.54819
StEt 56 4.48863636973214 norm_Ppp1r2 -2.08041
StEt 56 4.48863636973214 norm_Adam23 -0.82219
StEt 56 4.48863636973214 norm_Itgb6 -3.39765
StEt 56 4.48863636973214 norm_Cyr61 2.93734
StEt 56 4.48863636973214 norm_Dlk1 -0.08985
StEt 56 4.48863636973214 norm_Ahr -3.05383
StEt 56 4.48863636973214 norm_Ccl2 3.83644
StEt 56 4.48863636973214 norm_Eno2 -0.65454
StEt 56 4.48863636973214 norm_Plin1 -2.13357
StEt 56 4.48863636973214 norm_Wfikkn2 0.72431
StEt 56 4.48863636973214 norm_Flrt2 0.58459
StEt 56 4.48863636973214 norm_Qdpr 0.71143
StEt 56 4.48863636973214 norm_Fas 1.2014
StEt 56 4.48863636973214 norm_Erbb4 -1.15692
StEt 56 4.48863636973214 norm_Riox2 1.52755
StEt 56 4.48863636973214 norm_Plxna4 -1.03947
StEt 56 4.48863636973214 norm_Epcam -1.80902
StEt 56 4.48863636973214 norm_Ccl3 -2.22226
StEt 56 4.48863636973214 norm_Crim1 -0.90718
StEt 56 4.48863636973214 norm_Vsig2 0.46732
StEt 56 4.48863636973214 norm_Hgf 2.04953
StEt 56 4.48863636973214 norm_Sez6l2 2.95866
StEt 56 4.48863636973214 norm_Il1a -0.83416
StEt 56 4.48863636973214 norm_Il23r 4.55716
StEt 56 4.48863636973214 norm_Dll1 0.29238
StEt 56 4.48863636973214 norm_Ddah1 -1.55796
StEt 56 4.48863636973214 norm_Il10 -3.41284
StEt 56 4.48863636973214 norm_Tnfrsf12a 1.40605
StEt 56 4.48863636973214 norm_Acvrl1 -0.3884
StEt 56 4.48863636973214 norm_Lgmn 0.9799

StEt 56	4.48863636973214	norm_Csf2	-2.97132
StEt 56	4.48863636973214	norm_Cxcl9	2.19793
StEt 56	4.48863636973214	norm_Map2k6	1.46892
StEt 56	4.48863636973214	norm_Itgb1bp2	-5.59841
StEt 56	4.48863636973214	norm_Il17f	-5.14144
StEt 56	4.48863636973214	norm_Il1b	-3.97544
StEt 56	4.48863636973214	norm_Casp3	4.00973
StEt 56	4.48863636973214	norm_Apbb1ip	-3.68957
StEt 56	4.48863636973214	norm_Wisp1	-0.01261
StEt 56	4.48863636973214	norm_Cdh6	0.25092
StEt 56	4.48863636973214	norm_Pdgfb	6.59218
StEt 56	4.48863636973214	norm_Igsf3	0.0405
StEt 56	4.48863636973214	norm_Tgfbr3	-0.39116
StEt 56	4.48863636973214	norm_Cxcl1	2.44764
StEt 56	4.48863636973214	norm_Pak4	-3.1261
StEt 56	4.48863636973214	norm_Cntn4	1.51758
StEt 56	4.48863636973214	norm_Ghrl	1.36654
StEt 56	4.48863636973214	norm_Lpl	0.20723
StEt 56	4.48863636973214	norm_Fstl3	1.09804
StEt 56	4.48863636973214	norm_Dctn2	-1.88648
StEt 56	4.48863636973214	norm_Il5	-4.40339
StEt 56	4.48863636973214	norm_Eda2r	-0.56586
StEt 56	4.48863636973214	norm_Ntf3	-2.18604
StEt 56	4.48863636973214	norm_Tnfsf12	0.93157
StEt 56	4.48863636973214	norm_Ccl20	3.78592
StEt 56	4.48863636973214	norm_Fli1	-4.08114
StEt 56	4.48863636973214	norm_Tpp1	3.41309
StEt 56	4.48863636973214	norm_Tnr	-1.27737
StEt 56	4.48863636973214	norm_Vegfd	-0.7944
StEt 56	4.48863636973214	norm_Parp1	2.96407
StEt 56	4.48863636973214	norm_Tnf	-3.43349
StEt 57	2.63905732961526	norm_Clmp	2.50267
StEt 57	2.63905732961526	norm_Matn2	1.42606
StEt 57	2.63905732961526	norm_Cpe	1.86822
StEt 57	2.63905732961526	norm_Gcg	0.49935
StEt 57	2.63905732961526	norm_Gdnf	-3.23457
StEt 57	2.63905732961526	norm_Yes1	-2.30082
StEt 57	2.63905732961526	norm_Il17a	-1.46457
StEt 57	2.63905732961526	norm_Foxo1	-1.68273
StEt 57	2.63905732961526	norm_Tnfrsf11b	0.8392
StEt 57	2.63905732961526	norm_Tgfb1	0.07606
StEt 57	2.63905732961526	norm_Pla2g4a	2.50243
StEt 57	2.63905732961526	norm_Il6	-4.5094
StEt 57	2.63905732961526	norm_Prdx5	-2.00824
StEt 57	2.63905732961526	norm_Tgfa	1.89317
StEt 57	2.63905732961526	norm_Ccl5	-2.04256
StEt 57	2.63905732961526	norm_Epo	-1.52316
StEt 57	2.63905732961526	norm_Axin1	-1.76092
StEt 57	2.63905732961526	norm_Fst	3.87025
StEt 57	2.63905732961526	norm_Rgma	-0.75506
StEt 57	2.63905732961526	norm_Nadk	3.14065
StEt 57	2.63905732961526	norm_Tnni3	8.88329
StEt 57	2.63905732961526	norm_Notch3	1.41806
StEt 57	2.63905732961526	norm_Snap29	1.99207
StEt 57	2.63905732961526	norm_Cntn1	5.53071
StEt 57	2.63905732961526	norm_Clstn2	-1.73712
StEt 57	2.63905732961526	norm_S100a4	6.09526
StEt 57	2.63905732961526	norm_Ca13	-2.73882
StEt 57	2.63905732961526	norm_Mia	0.12413
StEt 57	2.63905732961526	norm_Cant1	-1.72803
StEt 57	2.63905732961526	norm_Kitlg	-4.68582
StEt 57	2.63905732961526	norm_Gfra1	2.2248
StEt 57	2.63905732961526	norm_Ppp1r2	-2.90156
StEt 57	2.63905732961526	norm_Adam23	-1.05087
StEt 57	2.63905732961526	norm_Itgb6	-3.0037
StEt 57	2.63905732961526	norm_Cyr61	3.41624
StEt 57	2.63905732961526	norm_Dlk1	0.66056
StEt 57	2.63905732961526	norm_Ahr	-3.24318
StEt 57	2.63905732961526	norm_Ccl2	4.39027
StEt 57	2.63905732961526	norm_Eno2	-0.40756
StEt 57	2.63905732961526	norm_Plin1	-0.15173
StEt 57	2.63905732961526	norm_Wfikkn2	0.86139
StEt 57	2.63905732961526	norm_Flrt2	0.16908
StEt 57	2.63905732961526	norm_Qdpr	-1.41194
StEt 57	2.63905732961526	norm_Fas	1.13032
StEt 57	2.63905732961526	norm_Erbb4	-0.7991
StEt 57	2.63905732961526	norm_Riox2	-0.77371
StEt 57	2.63905732961526	norm_Plxna4	-0.23544
StEt 57	2.63905732961526	norm_Epcam	-1.50917
StEt 57	2.63905732961526	norm_Ccl3	-2.21867
StEt 57	2.63905732961526	norm_Crim1	-1.43579
StEt 57	2.63905732961526	norm_Vsig2	1.28743
StEt 57	2.63905732961526	norm_Hgf	1.55217
StEt 57	2.63905732961526	norm_Sez6l2	2.97791
StEt 57	2.63905732961526	norm_Il1a	-2.68317
StEt 57	2.63905732961526	norm_Il23r	4.22419
StEt 57	2.63905732961526	norm_Dll1	0.4923
StEt 57	2.63905732961526	norm_Ddah1	-3.66957
StEt 57	2.63905732961526	norm_Il10	-2.99716
StEt 57	2.63905732961526	norm_Tnfrsf12a	2.27073
StEt 57	2.63905732961526	norm_Acvrl1	0.22859
StEt 57	2.63905732961526	norm_Lgmn	1.25065
StEt 57	2.63905732961526	norm_Csf2	-3.0033
StEt 57	2.63905732961526	norm_Cxcl9	3.0112
StEt 57	2.63905732961526	norm_Map2k6	0.04105

StEt 57	2.63905732961526	norm_Itgb1bp2	-4.7175
StEt 57	2.63905732961526	norm_Il17f	-5.1358
StEt 57	2.63905732961526	norm_Il1b	-3.45582
StEt 57	2.63905732961526	norm_Casp3	2.64695
StEt 57	2.63905732961526	norm_Apbb1ip	-3.80046
StEt 57	2.63905732961526	norm_Wisp1	0.43413
StEt 57	2.63905732961526	norm_Cdh6	0.3977
StEt 57	2.63905732961526	norm_Pdgfb	5.93638
StEt 57	2.63905732961526	norm_Igsf3	0.22867
StEt 57	2.63905732961526	norm_Tgfbr3	0.26939
StEt 57	2.63905732961526	norm_Cxcl1	2.64056
StEt 57	2.63905732961526	norm_Pak4	-3.85496
StEt 57	2.63905732961526	norm_Cntn4	1.84765
StEt 57	2.63905732961526	norm_Ghrl	2.56811
StEt 57	2.63905732961526	norm_Lpl	-0.19721
StEt 57	2.63905732961526	norm_Fstl3	1.8847
StEt 57	2.63905732961526	norm_Dctn2	-2.6221
StEt 57	2.63905732961526	norm_Il5	-3.82495
StEt 57	2.63905732961526	norm_Eda2r	-0.07316
StEt 57	2.63905732961526	norm_Ntf3	-1.47227
StEt 57	2.63905732961526	norm_Tnfsf12	0.64618
StEt 57	2.63905732961526	norm_Ccl20	4.85366
StEt 57	2.63905732961526	norm_Fli1	-4.31265
StEt 57	2.63905732961526	norm_Tpp1	3.52927
StEt 57	2.63905732961526	norm_Tnr	-0.97781
StEt 57	2.63905732961526	norm_Vegfd	-0.63986
StEt 57	2.63905732961526	norm_Parp1	1.16461
StEt 57	2.63905732961526	norm_Tnf	-3.14633
StEt 58	2.77258872223978	norm_Clmp	1.74999
StEt 58	2.77258872223978	norm_Matn2	0.74099
StEt 58	2.77258872223978	norm_Cpe	1.47096
StEt 58	2.77258872223978	norm_Gcg	0.21119
StEt 58	2.77258872223978	norm_Gdnf	-4.05154
StEt 58	2.77258872223978	norm_Yes1	0.14782
StEt 58	2.77258872223978	norm_Il17a	-3.21774
StEt 58	2.77258872223978	norm_Foxo1	-0.81009
StEt 58	2.77258872223978	norm_Tnfrsf11b	0.04526
StEt 58	2.77258872223978	norm_Tgfb1	0.54286
StEt 58	2.77258872223978	norm_Pla2g4a	3.09964
StEt 58	2.77258872223978	norm_Il6	-5.11921
StEt 58	2.77258872223978	norm_Prdx5	0.30125
StEt 58	2.77258872223978	norm_Tgfa	2.49328
StEt 58	2.77258872223978	norm_Ccl5	-2.71216
StEt 58	2.77258872223978	norm_Epo	-2.25488
StEt 58	2.77258872223978	norm_Axin1	0.25974
StEt 58	2.77258872223978	norm_Fst	4.64427
StEt 58	2.77258872223978	norm_Rgma	-1.3855
StEt 58	2.77258872223978	norm_Nadk	5.2593
StEt 58	2.77258872223978	norm_Tnni3	9.39582
StEt 58	2.77258872223978	norm_Notch3	1.41519
StEt 58	2.77258872223978	norm_Snap29	4.14584
StEt 58	2.77258872223978	norm_Cntn1	4.8144
StEt 58	2.77258872223978	norm_Clstn2	-3.24906
StEt 58	2.77258872223978	norm_S100a4	5.64981
StEt 58	2.77258872223978	norm_Ca13	-2.38525
StEt 58	2.77258872223978	norm_Mia	-0.46366
StEt 58	2.77258872223978	norm_Cant1	-2.01318
StEt 58	2.77258872223978	norm_Kitlg	-4.65897
StEt 58	2.77258872223978	norm_Gfra1	-1.70496
StEt 58	2.77258872223978	norm_Ppp1r2	-1.18998
StEt 58	2.77258872223978	norm_Adam23	-1.54421
StEt 58	2.77258872223978	norm_Itgb6	-3.82983
StEt 58	2.77258872223978	norm_Cyr61	2.73795
StEt 58	2.77258872223978	norm_Dlk1	-0.16309
StEt 58	2.77258872223978	norm_Ahr	-1.44075
StEt 58	2.77258872223978	norm_Ccl2	4.4044
StEt 58	2.77258872223978	norm_Eno2	-0.28634
StEt 58	2.77258872223978	norm_Plin1	-0.96783
StEt 58	2.77258872223978	norm_Wfikkn2	0.41986
StEt 58	2.77258872223978	norm_Flrt2	0.58653
StEt 58	2.77258872223978	norm_Qdpr	1.94131
StEt 58	2.77258872223978	norm_Fas	1.64228
StEt 58	2.77258872223978	norm_Erbb4	-1.29656
StEt 58	2.77258872223978	norm_Riox2	1.18875
StEt 58	2.77258872223978	norm_Plxna4	-1.20204
StEt 58	2.77258872223978	norm_Epcam	-2.69131
StEt 58	2.77258872223978	norm_Ccl3	-1.89157
StEt 58	2.77258872223978	norm_Crim1	-0.83857
StEt 58	2.77258872223978	norm_Vsig2	0.94632
StEt 58	2.77258872223978	norm_Hgf	2.65562
StEt 58	2.77258872223978	norm_Sez6l2	2.23981
StEt 58	2.77258872223978	norm_Il1a	-0.25595
StEt 58	2.77258872223978	norm_Il23r	2.90666
StEt 58	2.77258872223978	norm_Dll1	-0.12284
StEt 58	2.77258872223978	norm_Ddah1	0.06812
StEt 58	2.77258872223978	norm_Il10	-3.55474
StEt 58	2.77258872223978	norm_Tnfrsf12a	1.17211
StEt 58	2.77258872223978	norm_Acvrl1	-0.76308
StEt 58	2.77258872223978	norm_Lgmn	1.12784
StEt 58	2.77258872223978	norm_Csf2	-3.40663
StEt 58	2.77258872223978	norm_Cxcl9	2.07578
StEt 58	2.77258872223978	norm_Map2k6	2.31917
StEt 58	2.77258872223978	norm_Itgb1bp2	-5.06314
StEt 58	2.77258872223978	norm_Il17f	-5.3897
StEt 58	2.77258872223978	norm_Il1b	-3.85681

StEt 58 2.77258872223978 norm_Casp3 4.14843
StEt 58 2.77258872223978 norm_Apbb1ip -3.42764
StEt 58 2.77258872223978 norm_Wisp1 -0.25315
StEt 58 2.77258872223978 norm_Cdh6 -0.20744
StEt 58 2.77258872223978 norm_Pdgfb 6.32432
StEt 58 2.77258872223978 norm_Igsf3 -0.31798
StEt 58 2.77258872223978 norm_Tgfbr3 -0.14143
StEt 58 2.77258872223978 norm_Cxcl1 2.12849
StEt 58 2.77258872223978 norm_Pak4 -2.89409
StEt 58 2.77258872223978 norm_Cntn4 1.27565
StEt 58 2.77258872223978 norm_Ghrl 0.14075
StEt 58 2.77258872223978 norm_Lpl -0.58801
StEt 58 2.77258872223978 norm_Fstl3 0.84522
StEt 58 2.77258872223978 norm_Dctn2 -0.53137
StEt 58 2.77258872223978 norm_Il5 -4.53073
StEt 58 2.77258872223978 norm_Eda2r -0.68931
StEt 58 2.77258872223978 norm_Ntf3 -2.8133
StEt 58 2.77258872223978 norm_Tnfsf12 0.82622
StEt 58 2.77258872223978 norm_Ccl20 3.99115
StEt 58 2.77258872223978 norm_Fli1 -4.19285
StEt 58 2.77258872223978 norm_Tpp1 3.19347
StEt 58 2.77258872223978 norm_Tnr -1.48664
StEt 58 2.77258872223978 norm_Vegfd -1.38163
StEt 58 2.77258872223978 norm_Parp1 3.01077
StEt 58 2.77258872223978 norm_Tnf -3.46756
StEt 59 2.77258872223978 norm_Clmp 1.48162
StEt 59 2.77258872223978 norm_Matn2 -0.36437
StEt 59 2.77258872223978 norm_Cpe 0.41813
StEt 59 2.77258872223978 norm_Gcg -0.44958
StEt 59 2.77258872223978 norm_Gdnf -4.09502
StEt 59 2.77258872223978 norm_Yes1 1.2832
StEt 59 2.77258872223978 norm_Il17a -2.80289
StEt 59 2.77258872223978 norm_Foxo1 -0.41856
StEt 59 2.77258872223978 norm_Tnfrsf11b -0.10699
StEt 59 2.77258872223978 norm_Tgfb1 -0.32638
StEt 59 2.77258872223978 norm_Pla2g4a 2.86834
StEt 59 2.77258872223978 norm_Il6 -2.23612
StEt 59 2.77258872223978 norm_Prdx5 1.53158
StEt 59 2.77258872223978 norm_Tgfa 2.70437
StEt 59 2.77258872223978 norm_Ccl5 -2.16487
StEt 59 2.77258872223978 norm_Epo -2.85965
StEt 59 2.77258872223978 norm_Axin1 1.05143
StEt 59 2.77258872223978 norm_Fst 4.2014
StEt 59 2.77258872223978 norm_Rgma -2.12359
StEt 59 2.77258872223978 norm_Nadk 4.11845
StEt 59 2.77258872223978 norm_Tnni3 5.76829
StEt 59 2.77258872223978 norm_Notch3 0.19128
StEt 59 2.77258872223978 norm_Snap29 4.03698
StEt 59 2.77258872223978 norm_Cntn1 4.20579
StEt 59 2.77258872223978 norm_Clstn2 -3.45876
StEt 59 2.77258872223978 norm_S100a4 5.44596
StEt 59 2.77258872223978 norm_Ca13 -2.02226
StEt 59 2.77258872223978 norm_Mia -1.09524
StEt 59 2.77258872223978 norm_Cant1 -2.42073
StEt 59 2.77258872223978 norm_Kitlg -4.62114
StEt 59 2.77258872223978 norm_Gfra1 1.71439
StEt 59 2.77258872223978 norm_Ppp1r2 -1.25617
StEt 59 2.77258872223978 norm_Adam23 -1.83335
StEt 59 2.77258872223978 norm_Itgb6 -3.23698
StEt 59 2.77258872223978 norm_Cyr61 2.30692
StEt 59 2.77258872223978 norm_Dlk1 -0.69365
StEt 59 2.77258872223978 norm_Ahr -0.67014
StEt 59 2.77258872223978 norm_Ccl2 3.88463
StEt 59 2.77258872223978 norm_Eno2 -0.91532
StEt 59 2.77258872223978 norm_Plin1 -0.10887
StEt 59 2.77258872223978 norm_Wfikkn2 -0.29034
StEt 59 2.77258872223978 norm_Flrt2 0.49961
StEt 59 2.77258872223978 norm_Qdpr 1.81425
StEt 59 2.77258872223978 norm_Fas 2.94679
StEt 59 2.77258872223978 norm_Erbb4 -1.6106
StEt 59 2.77258872223978 norm_Riox2 3.23115
StEt 59 2.77258872223978 norm_Plxna4 -0.57817
StEt 59 2.77258872223978 norm_Epcam -2.91294
StEt 59 2.77258872223978 norm_Ccl3 -2.15556
StEt 59 2.77258872223978 norm_Crim1 -1.35165
StEt 59 2.77258872223978 norm_Vsig2 -0.5329
StEt 59 2.77258872223978 norm_Hgf 1.74969
StEt 59 2.77258872223978 norm_Sez6l2 1.42508
StEt 59 2.77258872223978 norm_Il1a 1.41658
StEt 59 2.77258872223978 norm_Il23r 3.28895
StEt 59 2.77258872223978 norm_Dll1 -0.31603
StEt 59 2.77258872223978 norm_Ddah1 0.88905
StEt 59 2.77258872223978 norm_Il10 -3.31108
StEt 59 2.77258872223978 norm_Tnfrsf12a 0.46286
StEt 59 2.77258872223978 norm_Acvrl1 -1.12277
StEt 59 2.77258872223978 norm_Lgmn 0.87592
StEt 59 2.77258872223978 norm_Csf2 -3.58484
StEt 59 2.77258872223978 norm_Cxcl9 3.98069
StEt 59 2.77258872223978 norm_Map2k6 2.12158
StEt 59 2.77258872223978 norm_Itgb1bp2 -6.45886
StEt 59 2.77258872223978 norm_Il17f -6.26594
StEt 59 2.77258872223978 norm_Il1b -2.87564
StEt 59 2.77258872223978 norm_Casp3 3.76715
StEt 59 2.77258872223978 norm_Apbb1ip -3.19653
StEt 59 2.77258872223978 norm_Wisp1 -0.66334

StEt 59 2.77258872223978 norm_Cdh6 -0.65493
StEt 59 2.77258872223978 norm_Pdgfb 5.98328
StEt 59 2.77258872223978 norm_Igsf3 -0.78638
StEt 59 2.77258872223978 norm_Tgfbr3 -0.48908
StEt 59 2.77258872223978 norm_Cxcl1 2.49805
StEt 59 2.77258872223978 norm_Pak4 -1.7841
StEt 59 2.77258872223978 norm_Cntn4 0.44151
StEt 59 2.77258872223978 norm_Ghrl -0.20863
StEt 59 2.77258872223978 norm_Lpl -1.46845
StEt 59 2.77258872223978 norm_Fstl3 0.46278
StEt 59 2.77258872223978 norm_Dctn2 0.62072
StEt 59 2.77258872223978 norm_Il5 -4.41419
StEt 59 2.77258872223978 norm_Eda2r -0.7029
StEt 59 2.77258872223978 norm_Ntf3 -3.08414
StEt 59 2.77258872223978 norm_Tnfsf12 -0.37177
StEt 59 2.77258872223978 norm_Ccl20 6.58799
StEt 59 2.77258872223978 norm_Fli1 -1.0853
StEt 59 2.77258872223978 norm_Tpp1 2.4478
StEt 59 2.77258872223978 norm_Tnr -1.60182
StEt 59 2.77258872223978 norm_Vegfd -1.55129
StEt 59 2.77258872223978 norm_Parp1 5.32997
StEt 59 2.77258872223978 norm_Tnf -4.34344
StEt 60 2.89037175789616 norm_Clmp 2.37219
StEt 60 2.89037175789616 norm_Matn2 0.79447
StEt 60 2.89037175789616 norm_Cpe 1.86975
StEt 60 2.89037175789616 norm_Gcg -0.49244
StEt 60 2.89037175789616 norm_Gdnf -3.65239
StEt 60 2.89037175789616 norm_Yes1 -1.76408
StEt 60 2.89037175789616 norm_Il17a -3.48254
StEt 60 2.89037175789616 norm_Foxo1 -1.89672
StEt 60 2.89037175789616 norm_Tnfrsf11b 0.681
StEt 60 2.89037175789616 norm_Tgfb1 0.29266
StEt 60 2.89037175789616 norm_Pla2g4a 2.07054
StEt 60 2.89037175789616 norm_Il6 -4.37018
StEt 60 2.89037175789616 norm_Prdx5 -1.45163
StEt 60 2.89037175789616 norm_Tgfa 1.96084
StEt 60 2.89037175789616 norm_Ccl5 -2.38668
StEt 60 2.89037175789616 norm_Epo -1.55912
StEt 60 2.89037175789616 norm_Axin1 -1.27893
StEt 60 2.89037175789616 norm_Fst 4.85457
StEt 60 2.89037175789616 norm_Rgma -0.92234
StEt 60 2.89037175789616 norm_Nadk 3.70137
StEt 60 2.89037175789616 norm_Tnni3 10.57933
StEt 60 2.89037175789616 norm_Notch3 1.62647
StEt 60 2.89037175789616 norm_Snap29 2.67516
StEt 60 2.89037175789616 norm_Cntn1 5.58609
StEt 60 2.89037175789616 norm_Clstn2 -2.61127
StEt 60 2.89037175789616 norm_S100a4 6.194
StEt 60 2.89037175789616 norm_Ca13 -3.09886
StEt 60 2.89037175789616 norm_Mia 0.06839
StEt 60 2.89037175789616 norm_Cant1 -1.71949
StEt 60 2.89037175789616 norm_Kitlg -4.37831
StEt 60 2.89037175789616 norm_Gfra1 -1.12265
StEt 60 2.89037175789616 norm_Ppp1r2 -2.22868
StEt 60 2.89037175789616 norm_Adam23 -1.33181
StEt 60 2.89037175789616 norm_Itgb6 -3.08964
StEt 60 2.89037175789616 norm_Cyr61 3.22284
StEt 60 2.89037175789616 norm_Dlk1 0.48061
StEt 60 2.89037175789616 norm_Ahr -2.7633
StEt 60 2.89037175789616 norm_Ccl2 4.05068
StEt 60 2.89037175789616 norm_Eno2 -0.40144
StEt 60 2.89037175789616 norm_Plin1 -1.16786
StEt 60 2.89037175789616 norm_Wfikkn2 1.13786
StEt 60 2.89037175789616 norm_Flrt2 0.0511
StEt 60 2.89037175789616 norm_Qdpr 0.51519
StEt 60 2.89037175789616 norm_Fas 0.93341
StEt 60 2.89037175789616 norm_Erbb4 -0.70567
StEt 60 2.89037175789616 norm_Riox2 0.25874
StEt 60 2.89037175789616 norm_Plxna4 -0.09992
StEt 60 2.89037175789616 norm_Epcam -2.14498
StEt 60 2.89037175789616 norm_Ccl3 -2.17076
StEt 60 2.89037175789616 norm_Crim1 -0.84737
StEt 60 2.89037175789616 norm_Vsig2 0.73421
StEt 60 2.89037175789616 norm_Hgf 1.76107
StEt 60 2.89037175789616 norm_Sez6l2 3.00384
StEt 60 2.89037175789616 norm_Il1a -0.90339
StEt 60 2.89037175789616 norm_Il23r 3.41571
StEt 60 2.89037175789616 norm_Dll1 0.4301
StEt 60 2.89037175789616 norm_Ddah1 -2.04131
StEt 60 2.89037175789616 norm_Il10 -3.24189
StEt 60 2.89037175789616 norm_Tnfrsf12a 2.13884
StEt 60 2.89037175789616 norm_Acvrl1 -0.28727
StEt 60 2.89037175789616 norm_Lgmn 1.18979
StEt 60 2.89037175789616 norm_Csf2 -2.99406
StEt 60 2.89037175789616 norm_Cxcl9 2.48674
StEt 60 2.89037175789616 norm_Map2k6 1.07927
StEt 60 2.89037175789616 norm_Itgb1bp2 -5.46403
StEt 60 2.89037175789616 norm_Il17f -5.04116
StEt 60 2.89037175789616 norm_Il1b -3.55878
StEt 60 2.89037175789616 norm_Casp3 2.62479
StEt 60 2.89037175789616 norm_Apbb1ip -4.09154
StEt 60 2.89037175789616 norm_Wisp1 0.12589
StEt 60 2.89037175789616 norm_Cdh6 0.83705
StEt 60 2.89037175789616 norm_Pdgfb 6.18292
StEt 60 2.89037175789616 norm_Igsf3 -0.23188

```
StEt 60  2.89037175789616  norm_Tgfbr3  0.44725
StEt 60  2.89037175789616  norm_Cxcl1  2.36051
StEt 60  2.89037175789616  norm_Pak4  -3.70148
StEt 60  2.89037175789616  norm_Cntn4  2.91803
StEt 60  2.89037175789616  norm_Ghrl  2.4864
StEt 60  2.89037175789616  norm_Lpl  -0.05771
StEt 60  2.89037175789616  norm_Fstl3  1.59081
StEt 60  2.89037175789616  norm_Dctn2  -2.28504
StEt 60  2.89037175789616  norm_Il5  -3.60778
StEt 60  2.89037175789616  norm_Eda2r  -0.23523
StEt 60  2.89037175789616  norm_Ntf3  -1.54839
StEt 60  2.89037175789616  norm_Tnfsf12  0.8654
StEt 60  2.89037175789616  norm_Ccl20  4.84111
StEt 60  2.89037175789616  norm_Fli1  -4.54588
StEt 60  2.89037175789616  norm_Tpp1  3.1513
StEt 60  2.89037175789616  norm_Tnr  -0.6504
StEt 60  2.89037175789616  norm_Vegfd  -0.6513
StEt 60  2.89037175789616  norm_Parp1  0.97355
StEt 60  2.89037175789616  norm_Tnf  -3.34469
;
RUN;

/*This analysis follows the same concepts as the analyses for Plasma GSH Level and Stereotypy
vs protein expression above, including troubleshooting REML-related errors.
In this case the model is considerably simpler, as we do not include an age effect (see text).
As before the Estimates generate the regression slopes and p-values for each protein
to be used in the final Benjamini Hochberg calculations*/


PROC MIXED DATA=CD1_Olink_And_ST_20241125 ALPHA=0.05;
CLASS SubjectID Protein;
MODEL  Norm_NPX =  Log_SUMMED_ST_1 Protein Log_SUMMED_ST_1*Protein / DDFM=CONTAIN;
RANDOM SubjectID  ;

Estimate "Acvrl1 Slope" Log_SUMMED_ST_1 1 Log_SUMMED_ST_1*Protein 1 0 0 0 0 0 0 0 0 0 0 0 0 0 0 0 0 0 0 0 0 0 0 0 0 0 0 0 0 0 0 0
0 0 0 0 0 0 0 0 0 0 0 0 0 0 0 0 0 0 0 0 0 0 0 0 0 0 0 0 0 0 0 0 0 0 0 0 0 0 0 0 0 0 0 0 0 0 0 0 0 0 0 0 0 0 0 0 0 0 0 0 ;
Estimate "Adam23 Slope" Log_SUMMED_ST_1 1 Log_SUMMED_ST_1*Protein 0 1 0 0 0 0 0 0 0 0 0 0 0 0 0 0 0 0 0 0 0 0 0 0 0 0 0 0 0 0 0 0
0 0 0 0 0 0 0 0 0 0 0 0 0 0 0 0 0 0 0 0 0 0 0 0 0 0 0 0 0 0 0 0 0 0 0 0 0 0 0 0 0 0 0 0 0 0 0 0 0 0 0 0 0 0 0 0 0 0 0 0  ;
Estimate "Ahr Slope" Log_SUMMED_ST_1 1 Log_SUMMED_ST_1*Protein 0 0 1 0 0 0 0 0 0 0 0 0 0 0 0 0 0 0 0 0 0 0 0 0 0 0 0 0 0 0 0 0 0
0 0 0 0 0 0 0 0 0 0 0 0 0 0 0 0 0 0 0 0 0 0 0 0 0 0 0 0 0 0 0 0 0 0 0 0 0 0 0 0 0 0 0 0 0 0 0 0 0 0 0 0 0 0 0 0 0 0 0  ;
Estimate "Apbb1ip Slope" Log_SUMMED_ST_1 1 Log_SUMMED_ST_1*Protein 0 0 0 1 0 0 0 0 0 0 0 0 0 0 0 0 0 0 0 0 0 0 0 0 0 0 0 0 0 0 0
0 0 0 0 0 0 0 0 0 0 0 0 0 0 0 0 0 0 0 0 0 0 0 0 0 0 0 0 0 0 0 0 0 0 0 0 0 0 0 0 0 0 0 0 0 0 0 0 0 0 0 0 0 0 0 0 0 0 0 0 0  ;
Estimate "Axin1 Slope" Log_SUMMED_ST_1 1 Log_SUMMED_ST_1*Protein 0 0 0 0 1 0 0 0 0 0 0 0 0 0 0 0 0 0 0 0 0 0 0 0 0 0 0 0 0 0 0 0
0 0 0 0 0 0 0 0 0 0 0 0 0 0 0 0 0 0 0 0 0 0 0 0 0 0 0 0 0 0 0 0 0 0 0 0 0 0 0 0 0 0 0 0 0 0 0 0 0 0 0 0 0 0 0 0 0 0 0 0  ;
Estimate "Cal3 Slope" Log_SUMMED_ST_1 1 Log_SUMMED_ST_1*Protein 0 0 0 0 0 1 0 0 0 0 0 0 0 0 0 0 0 0 0 0 0 0 0 0 0 0 0 0 0 0 0 0 0
0 0 0 0 0 0 0 0 0 0 0 0 0 0 0 0 0 0 0 0 0 0 0 0 0 0 0 0 0 0 0 0 0 0 0 0 0 0 0 0 0 0 0 0 0 0 0 0 0 0 0 0 0 0 0 0 0 0 0  ;
Estimate "Cant1 Slope" Log_SUMMED_ST_1 1 Log_SUMMED_ST_1*Protein 0 0 0 0 0 0 1 0 0 0 0 0 0 0 0 0 0 0 0 0 0 0 0 0 0 0 0 0 0 0 0 0
0 0 0 0 0 0 0 0 0 0 0 0 0 0 0 0 0 0 0 0 0 0 0 0 0 0 0 0 0 0 0 0 0 0 0 0 0 0 0 0 0 0 0 0 0 0 0 0 0 0 0 0 0 0 0 0 0 0 0 0  ;
Estimate "Casp3 Slope" Log_SUMMED_ST_1 1 Log_SUMMED_ST_1*Protein 0 0 0 0 0 0 0 1 0 0 0 0 0 0 0 0 0 0 0 0 0 0 0 0 0 0 0 0 0 0 0 0
0 0 0 0 0 0 0 0 0 0 0 0 0 0 0 0 0 0 0 0 0 0 0 0 0 0 0 0 0 0 0 0 0 0 0 0 0 0 0 0 0 0 0 0 0 0 0 0 0 0 0 0 0 0 0 0 0 0 0 0  ;
Estimate "Ccl2 Slope" Log_SUMMED_ST_1 1 Log_SUMMED_ST_1*Protein 0 0 0 0 0 0 0 0 1 0 0 0 0 0 0 0 0 0 0 0 0 0 0 0 0 0 0 0 0 0 0 0 0
0 0 0 0 0 0 0 0 0 0 0 0 0 0 0 0 0 0 0 0 0 0 0 0 0 0 0 0 0 0 0 0 0 0 0 0 0 0 0 0 0 0 0 0 0 0 0 0 0 0 0 0 0 0 0 0 0 0 0  ;
Estimate "Ccl20 Slope" Log_SUMMED_ST_1 1 Log_SUMMED_ST_1*Protein 0 0 0 0 0 0 0 0 0 1 0 0 0 0 0 0 0 0 0 0 0 0 0 0 0 0 0 0 0 0 0 0
0 0 0 0 0 0 0 0 0 0 0 0 0 0 0 0 0 0 0 0 0 0 0 0 0 0 0 0 0 0 0 0 0 0 0 0 0 0 0 0 0 0 0 0 0 0 0 0 0 0 0 0 0 0 0 0 0 0 0 0  ;
Estimate "Ccl3 Slope" Log_SUMMED_ST_1 1 Log_SUMMED_ST_1*Protein 0 0 0 0 0 0 0 0 0 0 1 0 0 0 0 0 0 0 0 0 0 0 0 0 0 0 0 0 0 0 0 0 0
0 0 0 0 0 0 0 0 0 0 0 0 0 0 0 0 0 0 0 0 0 0 0 0 0 0 0 0 0 0 0 0 0 0 0 0 0 0 0 0 0 0 0 0 0 0 0 0 0 0 0 0 0 0 0 0 0 0 0  ;
Estimate "Ccl5 Slope" Log_SUMMED_ST_1 1 Log_SUMMED_ST_1*Protein 0 0 0 0 0 0 0 0 0 0 0 1 0 0 0 0 0 0 0 0 0 0 0 0 0 0 0 0 0 0 0 0 0
0 0 0 0 0 0 0 0 0 0 0 0 0 0 0 0 0 0 0 0 0 0 0 0 0 0 0 0 0 0 0 0 0 0 0 0 0 0 0 0 0 0 0 0 0 0 0 0 0 0 0 0 0 0 0 0 0 0 0  ;
Estimate "Cdh6 Slope" Log_SUMMED_ST_1 1 Log_SUMMED_ST_1*Protein 0 0 0 0 0 0 0 0 0 0 0 0 1 0 0 0 0 0 0 0 0 0 0 0 0 0 0 0 0 0 0 0 0
0 0 0 0 0 0 0 0 0 0 0 0 0 0 0 0 0 0 0 0 0 0 0 0 0 0 0 0 0 0 0 0 0 0 0 0 0 0 0 0 0 0 0 0 0 0 0 0 0 0 0 0 0 0 0 0 0 0 0  ;
Estimate "Clmp Slope" Log_SUMMED_ST_1 1 Log_SUMMED_ST_1*Protein 0 0 0 0 0 0 0 0 0 0 0 0 0 1 0 0 0 0 0 0 0 0 0 0 0 0 0 0 0 0 0 0 0
0 0 0 0 0 0 0 0 0 0 0 0 0 0 0 0 0 0 0 0 0 0 0 0 0 0 0 0 0 0 0 0 0 0 0 0 0 0 0 0 0 0 0 0 0 0 0 0 0 0 0 0 0 0 0 0 0 0 0  ;
Estimate "Clstn2 Slope" Log_SUMMED_ST_1 1 Log_SUMMED_ST_1*Protein 0 0 0 0 0 0 0 0 0 0 0 0 0 0 1 0 0 0 0 0 0 0 0 0 0 0 0 0 0 0 0 0
0 0 0 0 0 0 0 0 0 0 0 0 0 0 0 0 0 0 0 0 0 0 0 0 0 0 0 0 0 0 0 0 0 0 0 0 0 0 0 0 0 0 0 0 0 0 0 0 0 0 0 0 0 0 0 0 0 0 0 0  ;
Estimate "Cntn1 Slope" Log_SUMMED_ST_1 1 Log_SUMMED_ST_1*Protein 0 0 0 0 0 0 0 0 0 0 0 0 0 0 0 1 0 0 0 0 0 0 0 0 0 0 0 0 0 0 0 0
0 0 0 0 0 0 0 0 0 0 0 0 0 0 0 0 0 0 0 0 0 0 0 0 0 0 0 0 0 0 0 0 0 0 0 0 0 0 0 0 0 0 0 0 0 0 0 0 0 0 0 0 0 0 0 0 0 0 0 0  ;
Estimate "Cntn4 Slope" Log_SUMMED_ST_1 1 Log_SUMMED_ST_1*Protein 0 0 0 0 0 0 0 0 0 0 0 0 0 0 0 0 1 0 0 0 0 0 0 0 0 0 0 0 0 0 0 0
0 0 0 0 0 0 0 0 0 0 0 0 0 0 0 0 0 0 0 0 0 0 0 0 0 0 0 0 0 0 0 0 0 0 0 0 0 0 0 0 0 0 0 0 0 0 0 0 0 0 0 0 0 0 0 0 0 0 0 0  ;
Estimate "Cpe Slope" Log_SUMMED_ST_1 1 Log_SUMMED_ST_1*Protein 0 0 0 0 0 0 0 0 0 0 0 0 0 0 0 0 0 1 0 0 0 0 0 0 0 0 0 0 0 0 0 0 0
0 0 0 0 0 0 0 0 0 0 0 0 0 0 0 0 0 0 0 0 0 0 0 0 0 0 0 0 0 0 0 0 0 0 0 0 0 0 0 0 0 0 0 0 0 0 0 0 0 0 0 0 0 0 0 0 0 0 0  ;
Estimate "Crim1 Slope" Log_SUMMED_ST_1 1 Log_SUMMED_ST_1*Protein 0 0 0 0 0 0 0 0 0 0 0 0 0 0 0 0 0 0 1 0 0 0 0 0 0 0 0 0 0 0 0 0
0 0 0 0 0 0 0 0 0 0 0 0 0 0 0 0 0 0 0 0 0 0 0 0 0 0 0 0 0 0 0 0 0 0 0 0 0 0 0 0 0 0 0 0 0 0 0 0 0 0 0 0 0 0 0 0 0 0 0 0  ;
Estimate "Csf2 Slope" Log_SUMMED_ST_1 1 Log_SUMMED_ST_1*Protein 0 0 0 0 0 0 0 0 0 0 0 0 0 0 0 0 0 0 0 1 0 0 0 0 0 0 0 0 0 0 0 0 0
0 0 0 0 0 0 0 0 0 0 0 0 0 0 0 0 0 0 0 0 0 0 0 0 0 0 0 0 0 0 0 0 0 0 0 0 0 0 0 0 0 0 0 0 0 0 0 0 0 0 0 0 0 0 0 0 0 0 0  ;
Estimate "Cxcl1 Slope" Log_SUMMED_ST_1 1 Log_SUMMED_ST_1*Protein 0 0 0 0 0 0 0 0 0 0 0 0 0 0 0 0 0 0 0 0 1 0 0 0 0 0 0 0 0 0 0 0
0 0 0 0 0 0 0 0 0 0 0 0 0 0 0 0 0 0 0 0 0 0 0 0 0 0 0 0 0 0 0 0 0 0 0 0 0 0 0 0 0 0 0 0 0 0 0 0 0 0 0 0 0 0 0 0 0 0 0 0  ;
Estimate "Cxcl9 Slope" Log_SUMMED_ST_1 1 Log_SUMMED_ST_1*Protein 0 0 0 0 0 0 0 0 0 0 0 0 0 0 0 0 0 0 0 0 0 1 0 0 0 0 0 0 0 0 0 0
0 0 0 0 0 0 0 0 0 0 0 0 0 0 0 0 0 0 0 0 0 0 0 0 0 0 0 0 0 0 0 0 0 0 0 0 0 0 0 0 0 0 0 0 0 0 0 0 0 0 0 0 0 0 0 0 0 0 0 0  ;
Estimate "Cyr61 Slope" Log_SUMMED_ST_1 1 Log_SUMMED_ST_1*Protein 0 0 0 0 0 0 0 0 0 0 0 0 0 0 0 0 0 0 0 0 0 0 1 0 0 0 0 0 0 0 0 0
0 0 0 0 0 0 0 0 0 0 0 0 0 0 0 0 0 0 0 0 0 0 0 0 0 0 0 0 0 0 0 0 0 0 0 0 0 0 0 0 0 0 0 0 0 0 0 0 0 0 0 0 0 0 0 0 0 0 0 0  ;
Estimate "Dctn2 Slope" Log_SUMMED_ST_1 1 Log_SUMMED_ST_1*Protein 0 0 0 0 0 0 0 0 0 0 0 0 0 0 0 0 0 0 0 0 0 0 0 1 0 0 0 0 0 0 0 0
0 0 0 0 0 0 0 0 0 0 0 0 0 0 0 0 0 0 0 0 0 0 0 0 0 0 0 0 0 0 0 0 0 0 0 0 0 0 0 0 0 0 0 0 0 0 0 0 0 0 0 0 0 0 0 0 0 0 0 0  ;
Estimate "Ddah1 Slope" Log_SUMMED_ST_1 1 Log_SUMMED_ST_1*Protein 0 0 0 0 0 0 0 0 0 0 0 0 0 0 0 0 0 0 0 0 0 0 0 0 1 0 0 0 0 0 0 0
0 0 0 0 0 0 0 0 0 0 0 0 0 0 0 0 0 0 0 0 0 0 0 0 0 0 0 0 0 0 0 0 0 0 0 0 0 0 0 0 0 0 0 0 0 0 0 0 0 0 0 0 0 0 0 0 0 0 0 0  ;
Estimate "Dlk1 Slope" Log_SUMMED_ST_1 1 Log_SUMMED_ST_1*Protein 0 0 0 0 0 0 0 0 0 0 0 0 0 0 0 0 0 0 0 0 0 0 0 0 0 1 0 0 0 0 0 0 0
0 0 0 0 0 0 0 0 0 0 0 0 0 0 0 0 0 0 0 0 0 0 0 0 0 0 0 0 0 0 0 0 0 0 0 0 0 0 0 0 0 0 0 0 0 0 0 0 0 0 0 0 0 0 0 0 0 0 0  ;
Estimate "Dll1 Slope" Log_SUMMED_ST_1 1 Log_SUMMED_ST_1*Protein 0 0 0 0 0 0 0 0 0 0 0 0 0 0 0 0 0 0 0 0 0 0 0 0 0 0 1 0 0 0 0 0 0
0 0 0 0 0 0 0 0 0 0 0 0 0 0 0 0 0 0 0 0 0 0 0 0 0 0 0 0 0 0 0 0 0 0 0 0 0 0 0 0 0 0 0 0 0 0 0 0 0 0 0 0 0 0 0 0 0 0 0  ;
Estimate "Eda2r Slope" Log_SUMMED_ST_1 1 Log_SUMMED_ST_1*Protein 0 0 0 0 0 0 0 0 0 0 0 0 0 0 0 0 0 0 0 0 0 0 0 0 0 0 0 1 0 0 0 0
0 0 0 0 0 0 0 0 0 0 0 0 0 0 0 0 0 0 0 0 0 0 0 0 0 0 0 0 0 0 0 0 0 0 0 0 0 0 0 0 0 0 0 0 0 0 0 0 0 0 0 0 0 0 0 0 0 0 0 0  ;
Estimate "Eno2 Slope" Log_SUMMED_ST_1 1 Log_SUMMED_ST_1*Protein 0 0 0 0 0 0 0 0 0 0 0 0 0 0 0 0 0 0 0 0 0 0 0 0 0 0 0 0 1 0 0 0 0
0 0 0 0 0 0 0 0 0 0 0 0 0 0 0 0 0 0 0 0 0 0 0 0 0 0 0 0 0 0 0 0 0 0 0 0 0 0 0 0 0 0 0 0 0 0 0 0 0 0 0 0 0 0 0 0 0 0 0  ;
Estimate "Epcam Slope" Log_SUMMED_ST_1 1 Log_SUMMED_ST_1*Protein 0 0 0 0 0 0 0 0 0 0 0 0 0 0 0 0 0 0 0 0 0 0 0 0 0 0 0 0 0 1 0 0
0 0 0 0 0 0 0 0 0 0 0 0 0 0 0 0 0 0 0 0 0 0 0 0 0 0 0 0 0 0 0 0 0 0 0 0 0 0 0 0 0 0 0 0 0 0 0 0 0 0 0 0 0 0 0 0 0 0 0 0  ;
```

```
Estimate "Epo Slope" Log_SUMMED_ST_1 1 Log_SUMMED_ST_1*Protein 0 0 0 0 0 0 0 0 0 0 0 0 0 0 0 0 0 0 0 0 0 0 0 0 0 0 0 0 0 0 1 0 0
0 0 0 0 0 0 0 0 0 0 0 0 0 0 0 0 0 0 0 0 0 0 0 0 0 0 0 0 0 0 0 0 0 0 0 0 0 0 0 0 0 0 0 0 0 0 0 0 0 0 0 0 0 0 0 0 0 0 0 ;
Estimate "Erbb4 Slope" Log_SUMMED_ST_1 1 Log_SUMMED_ST_1*Protein 0 0 0 0 0 0 0 0 0 0 0 0 0 0 0 0 0 0 0 0 0 0 0 0 0 0 0 0 0 0 0 1
0 0 0 0 0 0 0 0 0 0 0 0 0 0 0 0 0 0 0 0 0 0 0 0 0 0 0 0 0 0 0 0 0 0 0 0 0 0 0 0 0 0 0 0 0 0 0 0 0 0 0 0 0 0 0 0 0 0 0 0 ;
Estimate "Fas Slope" Log_SUMMED_ST_1 1 Log_SUMMED_ST_1*Protein 0 0 0 0 0 0 0 0 0 0 0 0 0 0 0 0 0 0 0 0 0 0 0 0 0 0 0 0 0 0 0 0 1
0 0 0 0 0 0 0 0 0 0 0 0 0 0 0 0 0 0 0 0 0 0 0 0 0 0 0 0 0 0 0 0 0 0 0 0 0 0 0 0 0 0 0 0 0 0 0 0 0 0 0 0 0 0 0 0 0 0 0 ;
Estimate "Fli1 Slope" Log_SUMMED_ST_1 1 Log_SUMMED_ST_1*Protein 0 0 0 0 0 0 0 0 0 0 0 0 0 0 0 0 0 0 0 0 0 0 0 0 0 0 0 0 0 0 0 0 0
1 0 0 0 0 0 0 0 0 0 0 0 0 0 0 0 0 0 0 0 0 0 0 0 0 0 0 0 0 0 0 0 0 0 0 0 0 0 0 0 0 0 0 0 0 0 0 0 0 0 0 0 0 0 0 0 0 0 0 ;
Estimate "Flrt2 Slope" Log_SUMMED_ST_1 1 Log_SUMMED_ST_1*Protein 0 0 0 0 0 0 0 0 0 0 0 0 0 0 0 0 0 0 0 0 0 0 0 0 0 0 0 0 0 0 0 0
0 0 1 0 0 0 0 0 0 0 0 0 0 0 0 0 0 0 0 0 0 0 0 0 0 0 0 0 0 0 0 0 0 0 0 0 0 0 0 0 0 0 0 0 0 0 0 0 0 0 0 0 0 0 0 0 0 0 0 0 ;
Estimate "Foxo1 Slope" Log_SUMMED_ST_1 1 Log_SUMMED_ST_1*Protein 0 0 0 0 0 0 0 0 0 0 0 0 0 0 0 0 0 0 0 0 0 0 0 0 0 0 0 0 0 0 0 0
0 0 0 1 0 0 0 0 0 0 0 0 0 0 0 0 0 0 0 0 0 0 0 0 0 0 0 0 0 0 0 0 0 0 0 0 0 0 0 0 0 0 0 0 0 0 0 0 0 0 0 0 0 0 0 0 0 0 0 0 ;
Estimate "Fst Slope" Log_SUMMED_ST_1 1 Log_SUMMED_ST_1*Protein 0 0 0 0 0 0 0 0 0 0 0 0 0 0 0 0 0 0 0 0 0 0 0 0 0 0 0 0 0 0 0 0 0
0 0 0 1 0 0 0 0 0 0 0 0 0 0 0 0 0 0 0 0 0 0 0 0 0 0 0 0 0 0 0 0 0 0 0 0 0 0 0 0 0 0 0 0 0 0 0 0 0 0 0 0 0 0 0 0 0 0 0 ;
Estimate "Fstl3 Slope" Log_SUMMED_ST_1 1 Log_SUMMED_ST_1*Protein 0 0 0 0 0 0 0 0 0 0 0 0 0 0 0 0 0 0 0 0 0 0 0 0 0 0 0 0 0 0 0 0
0 0 0 0 0 1 0 0 0 0 0 0 0 0 0 0 0 0 0 0 0 0 0 0 0 0 0 0 0 0 0 0 0 0 0 0 0 0 0 0 0 0 0 0 0 0 0 0 0 0 0 0 0 0 0 0 0 0 0 0 ;
Estimate "Gcg Slope" Log_SUMMED_ST_1 1 Log_SUMMED_ST_1*Protein 0 0 0 0 0 0 0 0 0 0 0 0 0 0 0 0 0 0 0 0 0 0 0 0 0 0 0 0 0 0 0 0 0
0 0 0 0 0 1 0 0 0 0 0 0 0 0 0 0 0 0 0 0 0 0 0 0 0 0 0 0 0 0 0 0 0 0 0 0 0 0 0 0 0 0 0 0 0 0 0 0 0 0 0 0 0 0 0 0 0 0 0 ;
Estimate "Gdnf Slope" Log_SUMMED_ST_1 1 Log_SUMMED_ST_1*Protein 0 0 0 0 0 0 0 0 0 0 0 0 0 0 0 0 0 0 0 0 0 0 0 0 0 0 0 0 0 0 0 0 0
0 0 0 0 0 0 1 0 0 0 0 0 0 0 0 0 0 0 0 0 0 0 0 0 0 0 0 0 0 0 0 0 0 0 0 0 0 0 0 0 0 0 0 0 0 0 0 0 0 0 0 0 0 0 0 0 0 0 0 ;
Estimate "Gfra1 Slope" Log_SUMMED_ST_1 1 Log_SUMMED_ST_1*Protein 0 0 0 0 0 0 0 0 0 0 0 0 0 0 0 0 0 0 0 0 0 0 0 0 0 0 0 0 0 0 0 0
0 0 0 0 0 0 0 0 1 0 0 0 0 0 0 0 0 0 0 0 0 0 0 0 0 0 0 0 0 0 0 0 0 0 0 0 0 0 0 0 0 0 0 0 0 0 0 0 0 0 0 0 0 0 0 0 0 0 0 0 ;
Estimate "Ghrl Slope" Log_SUMMED_ST_1 1 Log_SUMMED_ST_1*Protein 0 0 0 0 0 0 0 0 0 0 0 0 0 0 0 0 0 0 0 0 0 0 0 0 0 0 0 0 0 0 0 0 0
0 0 0 0 0 0 0 0 1 0 0 0 0 0 0 0 0 0 0 0 0 0 0 0 0 0 0 0 0 0 0 0 0 0 0 0 0 0 0 0 0 0 0 0 0 0 0 0 0 0 0 0 0 0 0 0 0 0 0 ;
Estimate "Hgf Slope" Log_SUMMED_ST_1 1 Log_SUMMED_ST_1*Protein 0 0 0 0 0 0 0 0 0 0 0 0 0 0 0 0 0 0 0 0 0 0 0 0 0 0 0 0 0 0 0 0 0
0 0 0 0 0 0 0 0 0 1 0 0 0 0 0 0 0 0 0 0 0 0 0 0 0 0 0 0 0 0 0 0 0 0 0 0 0 0 0 0 0 0 0 0 0 0 0 0 0 0 0 0 0 0 0 0 0 0 0 ;
Estimate "Igsf3 Slope" Log_SUMMED_ST_1 1 Log_SUMMED_ST_1*Protein 0 0 0 0 0 0 0 0 0 0 0 0 0 0 0 0 0 0 0 0 0 0 0 0 0 0 0 0 0 0 0 0
0 0 0 0 0 0 0 0 0 0 0 1 0 0 0 0 0 0 0 0 0 0 0 0 0 0 0 0 0 0 0 0 0 0 0 0 0 0 0 0 0 0 0 0 0 0 0 0 0 0 0 0 0 0 0 0 0 0 0 0 ;
Estimate "Il10 Slope" Log_SUMMED_ST_1 1 Log_SUMMED_ST_1*Protein 0 0 0 0 0 0 0 0 0 0 0 0 0 0 0 0 0 0 0 0 0 0 0 0 0 0 0 0 0 0 0 0 0
0 0 0 0 0 0 0 0 0 0 0 1 0 0 0 0 0 0 0 0 0 0 0 0 0 0 0 0 0 0 0 0 0 0 0 0 0 0 0 0 0 0 0 0 0 0 0 0 0 0 0 0 0 0 0 0 0 0 0 ;
Estimate "Il17a Slope" Log_SUMMED_ST_1 1 Log_SUMMED_ST_1*Protein 0 0 0 0 0 0 0 0 0 0 0 0 0 0 0 0 0 0 0 0 0 0 0 0 0 0 0 0 0 0 0 0
0 0 0 0 0 0 0 0 0 0 0 0 0 1 0 0 0 0 0 0 0 0 0 0 0 0 0 0 0 0 0 0 0 0 0 0 0 0 0 0 0 0 0 0 0 0 0 0 0 0 0 0 0 0 0 0 0 0 0 0 ;
Estimate "Il17f Slope" Log_SUMMED_ST_1 1 Log_SUMMED_ST_1*Protein 0 0 0 0 0 0 0 0 0 0 0 0 0 0 0 0 0 0 0 0 0 0 0 0 0 0 0 0 0 0 0 0
0 0 0 0 0 0 0 0 0 0 0 0 0 0 1 0 0 0 0 0 0 0 0 0 0 0 0 0 0 0 0 0 0 0 0 0 0 0 0 0 0 0 0 0 0 0 0 0 0 0 0 0 0 0 0 0 0 0 0 0 ;
Estimate "Il1a Slope" Log_SUMMED_ST_1 1 Log_SUMMED_ST_1*Protein 0 0 0 0 0 0 0 0 0 0 0 0 0 0 0 0 0 0 0 0 0 0 0 0 0 0 0 0 0 0 0 0 0
0 0 0 0 0 0 0 0 0 0 0 0 0 0 1 0 0 0 0 0 0 0 0 0 0 0 0 0 0 0 0 0 0 0 0 0 0 0 0 0 0 0 0 0 0 0 0 0 0 0 0 0 0 0 0 0 0 0 0 ;
Estimate "Il1b Slope" Log_SUMMED_ST_1 1 Log_SUMMED_ST_1*Protein 0 0 0 0 0 0 0 0 0 0 0 0 0 0 0 0 0 0 0 0 0 0 0 0 0 0 0 0 0 0 0 0 0
0 0 0 0 0 0 0 0 0 0 0 0 0 0 0 1 0 0 0 0 0 0 0 0 0 0 0 0 0 0 0 0 0 0 0 0 0 0 0 0 0 0 0 0 0 0 0 0 0 0 0 0 0 0 0 0 0 0 0 ;
Estimate "Il23r Slope" Log_SUMMED_ST_1 1 Log_SUMMED_ST_1*Protein 0 0 0 0 0 0 0 0 0 0 0 0 0 0 0 0 0 0 0 0 0 0 0 0 0 0 0 0 0 0 0 0
0 0 0 0 0 0 0 0 0 0 0 0 0 0 0 0 0 1 0 0 0 0 0 0 0 0 0 0 0 0 0 0 0 0 0 0 0 0 0 0 0 0 0 0 0 0 0 0 0 0 0 0 0 0 0 0 0 0 0 0 ;
Estimate "Il5 Slope" Log_SUMMED_ST_1 1 Log_SUMMED_ST_1*Protein 0 0 0 0 0 0 0 0 0 0 0 0 0 0 0 0 0 0 0 0 0 0 0 0 0 0 0 0 0 0 0 0 0
0 0 0 0 0 0 0 0 0 0 0 0 0 0 0 0 0 1 0 0 0 0 0 0 0 0 0 0 0 0 0 0 0 0 0 0 0 0 0 0 0 0 0 0 0 0 0 0 0 0 0 0 0 0 0 0 0 0 0 ;
Estimate "Il6 Slope" Log_SUMMED_ST_1 1 Log_SUMMED_ST_1*Protein 0 0 0 0 0 0 0 0 0 0 0 0 0 0 0 0 0 0 0 0 0 0 0 0 0 0 0 0 0 0 0 0 0
0 0 0 0 0 0 0 0 0 0 0 0 0 0 0 0 0 0 1 0 0 0 0 0 0 0 0 0 0 0 0 0 0 0 0 0 0 0 0 0 0 0 0 0 0 0 0 0 0 0 0 0 0 0 0 0 0 0 0 ;
Estimate "Itgb1bp2 Slope" Log_SUMMED_ST_1 1 Log_SUMMED_ST_1*Protein 0 0 0 0 0 0 0 0 0 0 0 0 0 0 0 0 0 0 0 0 0 0 0 0 0 0 0 0 0 0 0
0 0 0 0 0 0 0 0 0 0 0 0 0 0 0 0 0 0 0 0 0 1 0 0 0 0 0 0 0 0 0 0 0 0 0 0 0 0 0 0 0 0 0 0 0 0 0 0 0 0 0 0 0 0 0 0 0 0 0 0 0 ;
Estimate "Itgb6 Slope" Log_SUMMED_ST_1 1 Log_SUMMED_ST_1*Protein 0 0 0 0 0 0 0 0 0 0 0 0 0 0 0 0 0 0 0 0 0 0 0 0 0 0 0 0 0 0 0 0
0 0 0 0 0 0 0 0 0 0 0 0 0 0 0 0 0 0 0 0 0 1 0 0 0 0 0 0 0 0 0 0 0 0 0 0 0 0 0 0 0 0 0 0 0 0 0 0 0 0 0 0 0 0 0 0 0 0 0 0 ;
Estimate "Kitlg Slope" Log_SUMMED_ST_1 1 Log_SUMMED_ST_1*Protein 0 0 0 0 0 0 0 0 0 0 0 0 0 0 0 0 0 0 0 0 0 0 0 0 0 0 0 0 0 0 0 0
0 0 0 0 0 0 0 0 0 0 0 0 0 0 0 0 0 0 0 0 0 0 1 0 0 0 0 0 0 0 0 0 0 0 0 0 0 0 0 0 0 0 0 0 0 0 0 0 0 0 0 0 0 0 0 0 0 0 0 0 ;
Estimate "Lgmn Slope" Log_SUMMED_ST_1 1 Log_SUMMED_ST_1*Protein 0 0 0 0 0 0 0 0 0 0 0 0 0 0 0 0 0 0 0 0 0 0 0 0 0 0 0 0 0 0 0 0 0
0 0 0 0 0 0 0 0 0 0 0 0 0 0 0 0 0 0 0 0 0 0 1 0 0 0 0 0 0 0 0 0 0 0 0 0 0 0 0 0 0 0 0 0 0 0 0 0 0 0 0 0 0 0 0 0 0 0 0 ;
Estimate "Lpl Slope" Log_SUMMED_ST_1 1 Log_SUMMED_ST_1*Protein 0 0 0 0 0 0 0 0 0 0 0 0 0 0 0 0 0 0 0 0 0 0 0 0 0 0 0 0 0 0 0 0 0
0 0 0 0 0 0 0 0 0 0 0 0 0 0 0 0 0 0 0 0 0 0 0 1 0 0 0 0 0 0 0 0 0 0 0 0 0 0 0 0 0 0 0 0 0 0 0 0 0 0 0 0 0 0 0 0 0 0 0 ;
Estimate "Map2k6 Slope" Log_SUMMED_ST_1 1 Log_SUMMED_ST_1*Protein 0 0 0 0 0 0 0 0 0 0 0 0 0 0 0 0 0 0 0 0 0 0 0 0 0 0 0 0 0 0 0 0
0 0 0 0 0 0 0 0 0 0 0 0 0 0 0 0 0 0 0 0 0 0 0 0 0 1 0 0 0 0 0 0 0 0 0 0 0 0 0 0 0 0 0 0 0 0 0 0 0 0 0 0 0 0 0 0 0 0 0 0 ;
Estimate "Matn2 Slope" Log_SUMMED_ST_1 1 Log_SUMMED_ST_1*Protein 0 0 0 0 0 0 0 0 0 0 0 0 0 0 0 0 0 0 0 0 0 0 0 0 0 0 0 0 0 0 0 0
0 0 0 0 0 0 0 0 0 0 0 0 0 0 0 0 0 0 0 0 0 0 0 0 0 0 1 0 0 0 0 0 0 0 0 0 0 0 0 0 0 0 0 0 0 0 0 0 0 0 0 0 0 0 0 0 0 0 0 0 ;
Estimate "Mia Slope" Log_SUMMED_ST_1 1 Log_SUMMED_ST_1*Protein 0 0 0 0 0 0 0 0 0 0 0 0 0 0 0 0 0 0 0 0 0 0 0 0 0 0 0 0 0 0 0 0 0
0 0 0 0 0 0 0 0 0 0 0 0 0 0 0 0 0 0 0 0 0 0 0 0 0 0 1 0 0 0 0 0 0 0 0 0 0 0 0 0 0 0 0 0 0 0 0 0 0 0 0 0 0 0 0 0 0 0 0 ;
Estimate "Nadk Slope" Log_SUMMED_ST_1 1 Log_SUMMED_ST_1*Protein 0 0 0 0 0 0 0 0 0 0 0 0 0 0 0 0 0 0 0 0 0 0 0 0 0 0 0 0 0 0 0 0 0
0 0 0 0 0 0 0 0 0 0 0 0 0 0 0 0 0 0 0 0 0 0 0 0 0 0 0 1 0 0 0 0 0 0 0 0 0 0 0 0 0 0 0 0 0 0 0 0 0 0 0 0 0 0 0 0 0 0 0 ;
Estimate "Notch3 Slope" Log_SUMMED_ST_1 1 Log_SUMMED_ST_1*Protein 0 0 0 0 0 0 0 0 0 0 0 0 0 0 0 0 0 0 0 0 0 0 0 0 0 0 0 0 0 0 0 0
0 0 0 0 0 0 0 0 0 0 0 0 0 0 0 0 0 0 0 0 0 0 0 0 0 0 0 0 0 1 0 0 0 0 0 0 0 0 0 0 0 0 0 0 0 0 0 0 0 0 0 0 0 0 0 0 0 0 0 0 ;
Estimate "Ntf3 Slope" Log_SUMMED_ST_1 1 Log_SUMMED_ST_1*Protein 0 0 0 0 0 0 0 0 0 0 0 0 0 0 0 0 0 0 0 0 0 0 0 0 0 0 0 0 0 0 0 0 0
0 0 0 0 0 0 0 0 0 0 0 0 0 0 0 0 0 0 0 0 0 0 0 0 0 0 0 0 0 1 0 0 0 0 0 0 0 0 0 0 0 0 0 0 0 0 0 0 0 0 0 0 0 0 0 0 0 0 0 ;
Estimate "Pak4 Slope" Log_SUMMED_ST_1 1 Log_SUMMED_ST_1*Protein 0 0 0 0 0 0 0 0 0 0 0 0 0 0 0 0 0 0 0 0 0 0 0 0 0 0 0 0 0 0 0 0 0
0 0 0 0 0 0 0 0 0 0 0 0 0 0 0 0 0 0 0 0 0 0 0 0 0 0 0 0 0 0 1 0 0 0 0 0 0 0 0 0 0 0 0 0 0 0 0 0 0 0 0 0 0 0 0 0 0 0 0 ;
Estimate "Parp1 Slope" Log_SUMMED_ST_1 1 Log_SUMMED_ST_1*Protein 0 0 0 0 0 0 0 0 0 0 0 0 0 0 0 0 0 0 0 0 0 0 0 0 0 0 0 0 0 0 0 0
0 0 0 0 0 0 0 0 0 0 0 0 0 0 0 0 0 0 0 0 0 0 0 0 0 0 0 0 0 0 0 0 1 0 0 0 0 0 0 0 0 0 0 0 0 0 0 0 0 0 0 0 0 0 0 0 0 0 0 0 ;
Estimate "Pdgfb Slope" Log_SUMMED_ST_1 1 Log_SUMMED_ST_1*Protein 0 0 0 0 0 0 0 0 0 0 0 0 0 0 0 0 0 0 0 0 0 0 0 0 0 0 0 0 0 0 0 0
0 0 0 0 0 0 0 0 0 0 0 0 0 0 0 0 0 0 0 0 0 0 0 0 0 0 0 0 0 0 0 0 0 1 0 0 0 0 0 0 0 0 0 0 0 0 0 0 0 0 0 0 0 0 0 0 0 0 0 0 ;
Estimate "Pla2g4a Slope" Log_SUMMED_ST_1 1 Log_SUMMED_ST_1*Protein 0 0 0 0 0 0 0 0 0 0 0 0 0 0 0 0 0 0 0 0 0 0 0 0 0 0 0 0 0 0 0
0 0 0 0 0 0 0 0 0 0 0 0 0 0 0 0 0 0 0 0 0 0 0 0 0 0 0 0 0 0 0 0 0 0 0 1 0 0 0 0 0 0 0 0 0 0 0 0 0 0 0 0 0 0 0 0 0 0 0 0 0 ;
Estimate "Plin1 Slope" Log_SUMMED_ST_1 1 Log_SUMMED_ST_1*Protein 0 0 0 0 0 0 0 0 0 0 0 0 0 0 0 0 0 0 0 0 0 0 0 0 0 0 0 0 0 0 0 0
0 0 0 0 0 0 0 0 0 0 0 0 0 0 0 0 0 0 0 0 0 0 0 0 0 0 0 0 0 0 0 0 0 0 0 1 0 0 0 0 0 0 0 0 0 0 0 0 0 0 0 0 0 0 0 0 0 0 0 0 ;
Estimate "Plxna4 Slope" Log_SUMMED_ST_1 1 Log_SUMMED_ST_1*Protein 0 0 0 0 0 0 0 0 0 0 0 0 0 0 0 0 0 0 0 0 0 0 0 0 0 0 0 0 0 0 0 0
0 0 0 0 0 0 0 0 0 0 0 0 0 0 0 0 0 0 0 0 0 0 0 0 0 0 0 0 0 0 0 0 0 0 0 0 1 0 0 0 0 0 0 0 0 0 0 0 0 0 0 0 0 0 0 0 0 0 0 0 ;
Estimate "Ppp1r2 Slope" Log_SUMMED_ST_1 1 Log_SUMMED_ST_1*Protein 0 0 0 0 0 0 0 0 0 0 0 0 0 0 0 0 0 0 0 0 0 0 0 0 0 0 0 0 0 0 0 0
0 0 0 0 0 0 0 0 0 0 0 0 0 0 0 0 0 0 0 0 0 0 0 0 0 0 0 0 0 0 0 0 0 0 0 0 0 1 0 0 0 0 0 0 0 0 0 0 0 0 0 0 0 0 0 0 0 0 0 0 ;
Estimate "Prdx5 Slope" Log_SUMMED_ST_1 1 Log_SUMMED_ST_1*Protein 0 0 0 0 0 0 0 0 0 0 0 0 0 0 0 0 0 0 0 0 0 0 0 0 0 0 0 0 0 0 0 0
0 0 0 0 0 0 0 0 0 0 0 0 0 0 0 0 0 0 0 0 0 0 0 0 0 0 0 0 0 0 0 0 0 0 0 0 0 0 1 0 0 0 0 0 0 0 0 0 0 0 0 0 0 0 0 0 0 0 0 0 ;
Estimate "Qdpr Slope" Log_SUMMED_ST_1 1 Log_SUMMED_ST_1*Protein 0 0 0 0 0 0 0 0 0 0 0 0 0 0 0 0 0 0 0 0 0 0 0 0 0 0 0 0 0 0 0 0 0
0 0 0 0 0 0 0 0 0 0 0 0 0 0 0 0 0 0 0 0 0 0 0 0 0 0 0 0 0 0 0 0 0 0 0 0 0 0 1 0 0 0 0 0 0 0 0 0 0 0 0 0 0 0 0 0 0 0 0 ;
Estimate "Rgma Slope" Log_SUMMED_ST_1 1 Log_SUMMED_ST_1*Protein 0 0 0 0 0 0 0 0 0 0 0 0 0 0 0 0 0 0 0 0 0 0 0 0 0 0 0 0 0 0 0 0 0
0 0 0 0 0 0 0 0 0 0 0 0 0 0 0 0 0 0 0 0 0 0 0 0 0 0 0 0 0 0 0 0 0 0 0 0 0 0 0 1 0 0 0 0 0 0 0 0 0 0 0 0 0 0 0 0 0 0 0 ;
Estimate "Riox2 Slope" Log_SUMMED_ST_1 1 Log_SUMMED_ST_1*Protein 0 0 0 0 0 0 0 0 0 0 0 0 0 0 0 0 0 0 0 0 0 0 0 0 0 0 0 0 0 0 0 0
0 0 0 0 0 0 0 0 0 0 0 0 0 0 0 0 0 0 0 0 0 0 0 0 0 0 0 0 0 0 0 0 0 0 0 0 0 0 0 0 0 1 0 0 0 0 0 0 0 0 0 0 0 0 0 0 0 0 0 0 ;
Estimate "S100a4 Slope" Log_SUMMED_ST_1 1 Log_SUMMED_ST_1*Protein 0 0 0 0 0 0 0 0 0 0 0 0 0 0 0 0 0 0 0 0 0 0 0 0 0 0 0 0 0 0 0 0
0 0 0 0 0 0 0 0 0 0 0 0 0 0 0 0 0 0 0 0 0 0 0 0 0 0 0 0 0 0 0 0 0 0 0 0 0 0 0 0 0 0 1 0 0 0 0 0 0 0 0 0 0 0 0 0 0 0 0 0 ;
Estimate "Sez6l2 Slope" Log_SUMMED_ST_1 1 Log_SUMMED_ST_1*Protein 0 0 0 0 0 0 0 0 0 0 0 0 0 0 0 0 0 0 0 0 0 0 0 0 0 0 0 0 0 0 0 0
0 0 0 0 0 0 0 0 0 0 0 0 0 0 0 0 0 0 0 0 0 0 0 0 0 0 0 0 0 0 0 0 0 0 0 0 0 0 0 0 0 0 0 1 0 0 0 0 0 0 0 0 0 0 0 0 0 0 0 0 ;
Estimate "Snap29 Slope" Log_SUMMED_ST_1 1Log_SUMMED_ST_1*Protein 0 0 0 0 0 0 0 0 0 0 0 0 0 0 0 0 0 0 0 0 0 0 0 0 0 0 0 0 0 0 0 0
0 0 0 0 0 0 0 0 0 0 0 0 0 0 0 0 0 0 0 0 0 0 0 0 0 0 0 0 0 0 0 0 0 0 0 0 0 0 0 0 0 0 0 0 1 0 0 0 0 0 0 0 0 0 0 0 0 0 0 0 ;
```

```
Estimate "Tgfa Slope" Log_SUMMED_ST_1 1Log_SUMMED_ST_1*Protein 0 0 0 0 0 0 0 0 0 0 0 0 0 0 0 0 0 0 0 0 0 0 0 0 0 0 0 0 0 0 0 0 0
0 0 0 0 0 0 0 0 0 0 0 0 0 0 0 0 0 0 0 0 0 0 0 0 0 0 0 0 0 0 0 0 0 0 0 0 0 0 0 0 0 0 0 0 1 0 0 0 0 0 0 0 0 0 0 0 0 0 0 ;
Estimate "Tgfb1 Slope" Log_SUMMED_ST_1 1Log_SUMMED_ST_1*Protein 0 0 0 0 0 0 0 0 0 0 0 0 0 0 0 0 0 0 0 0 0 0 0 0 0 0 0 0 0 0 0 0 0
0 0 0 0 0 0 0 0 0 0 0 0 0 0 0 0 0 0 0 0 0 0 0 0 0 0 0 0 0 0 0 0 0 0 0 0 0 0 0 0 0 0 0 0 0 1 0 0 0 0 0 0 0 0 0 0 0 0 0 ;
Estimate "Tgfbr3 Slope" Log_SUMMED_ST_1 1Log_SUMMED_ST_1*Protein 0 0 0 0 0 0 0 0 0 0 0 0 0 0 0 0 0 0 0 0 0 0 0 0 0 0 0 0 0 0 0 0
0 0 0 0 0 0 0 0 0 0 0 0 0 0 0 0 0 0 0 0 0 0 0 0 0 0 0 0 0 0 0 0 0 0 0 0 0 0 0 0 0 0 0 0 0 0 0 1 0 0 0 0 0 0 0 0 0 0 0 0 ;
Estimate "Tnf Slope" Log_SUMMED_ST_1 1Log_SUMMED_ST_1*Protein 0 0 0 0 0 0 0 0 0 0 0 0 0 0 0 0 0 0 0 0 0 0 0 0 0 0 0 0 0 0 0 0 0 0
0 0 0 0 0 0 0 0 0 0 0 0 0 0 0 0 0 0 0 0 0 0 0 0 0 0 0 0 0 0 0 0 0 0 0 0 0 0 0 0 0 0 0 0 0 0 1 0 0 0 0 0 0 0 0 0 0 0 ;
Estimate "Tnfrsf11b Slope" Log_SUMMED_ST_1 1Log_SUMMED_ST_1*Protein 0 0 0 0 0 0 0 0 0 0 0 0 0 0 0 0 0 0 0 0 0 0 0 0 0 0 0 0 0 0 0
0 0 0 0 0 0 0 0 0 0 0 0 0 0 0 0 0 0 0 0 0 0 0 0 0 0 0 0 0 0 0 0 0 0 0 0 0 0 0 0 0 0 0 0 0 0 0 0 0 0 1 0 0 0 0 0 0 0 0 0 0 ;
Estimate "Tnfrsf12a Slope" Log_SUMMED_ST_1 1Log_SUMMED_ST_1*Protein 0 0 0 0 0 0 0 0 0 0 0 0 0 0 0 0 0 0 0 0 0 0 0 0 0 0 0 0 0 0 0
0 0 0 0 0 0 0 0 0 0 0 0 0 0 0 0 0 0 0 0 0 0 0 0 0 0 0 0 0 0 0 0 0 0 0 0 0 0 0 0 0 0 0 0 0 0 0 0 0 0 0 1 0 0 0 0 0 0 0 0 0 ;
Estimate "Tnfsf12 Slope" Log_SUMMED_ST_1 1Log_SUMMED_ST_1*Protein 0 0 0 0 0 0 0 0 0 0 0 0 0 0 0 0 0 0 0 0 0 0 0 0 0 0 0 0 0 0 0 0
0 0 0 0 0 0 0 0 0 0 0 0 0 0 0 0 0 0 0 0 0 0 0 0 0 0 0 0 0 0 0 0 0 0 0 0 0 0 0 0 0 0 0 0 0 0 0 0 0 0 0 1 0 0 0 0 0 0 0 0 ;
Estimate "Tnni3 Slope" Log_SUMMED_ST_1 1Log_SUMMED_ST_1*Protein 0 0 0 0 0 0 0 0 0 0 0 0 0 0 0 0 0 0 0 0 0 0 0 0 0 0 0 0 0 0 0 0 0
0 0 0 0 0 0 0 0 0 0 0 0 0 0 0 0 0 0 0 0 0 0 0 0 0 0 0 0 0 0 0 0 0 0 0 0 0 0 0 0 0 0 0 0 0 0 0 0 0 0 0 1 0 0 0 0 0 0 0 ;
Estimate "Tnr Slope" Log_SUMMED_ST_1 1Log_SUMMED_ST_1*Protein 0 0 0 0 0 0 0 0 0 0 0 0 0 0 0 0 0 0 0 0 0 0 0 0 0 0 0 0 0 0 0 0 0 0
0 0 0 0 0 0 0 0 0 0 0 0 0 0 0 0 0 0 0 0 0 0 0 0 0 0 0 0 0 0 0 0 0 0 0 0 0 0 0 0 0 0 0 0 0 0 0 0 0 0 0 1 0 0 0 0 0 0 ;
Estimate "Tpp1 Slope" Log_SUMMED_ST_1 1Log_SUMMED_ST_1*Protein 0 0 0 0 0 0 0 0 0 0 0 0 0 0 0 0 0 0 0 0 0 0 0 0 0 0 0 0 0 0 0 0 0
0 0 0 0 0 0 0 0 0 0 0 0 0 0 0 0 0 0 0 0 0 0 0 0 0 0 0 0 0 0 0 0 0 0 0 0 0 0 0 0 0 0 0 0 0 0 0 0 0 0 0 0 0 1 0 0 0 0 0 ;
Estimate "Vegfd Slope" Log_SUMMED_ST_1 1Log_SUMMED_ST_1*Protein 0 0 0 0 0 0 0 0 0 0 0 0 0 0 0 0 0 0 0 0 0 0 0 0 0 0 0 0 0 0 0 0 0
0 0 0 0 0 0 0 0 0 0 0 0 0 0 0 0 0 0 0 0 0 0 0 0 0 0 0 0 0 0 0 0 0 0 0 0 0 0 0 0 0 0 0 0 0 0 0 0 0 0 0 0 0 0 1 0 0 0 0 ;
Estimate "Vsig2 Slope" Log_SUMMED_ST_1 1Log_SUMMED_ST_1*Protein 0 0 0 0 0 0 0 0 0 0 0 0 0 0 0 0 0 0 0 0 0 0 0 0 0 0 0 0 0 0 0 0 0
0 0 0 0 0 0 0 0 0 0 0 0 0 0 0 0 0 0 0 0 0 0 0 0 0 0 0 0 0 0 0 0 0 0 0 0 0 0 0 0 0 0 0 0 0 0 0 0 0 0 0 0 0 0 0 1 0 0 0 ;
Estimate "Wfikkn2 Slope" Log_SUMMED_ST_1 1Log_SUMMED_ST_1*Protein 0 0 0 0 0 0 0 0 0 0 0 0 0 0 0 0 0 0 0 0 0 0 0 0 0 0 0 0 0 0 0 0
0 0 0 0 0 0 0 0 0 0 0 0 0 0 0 0 0 0 0 0 0 0 0 0 0 0 0 0 0 0 0 0 0 0 0 0 0 0 0 0 0 0 0 0 0 0 0 0 0 0 0 0 0 0 0 0 0 1 0 0 ;
Estimate "Wisp1 Slope" Log_SUMMED_ST_1 1Log_SUMMED_ST_1*Protein 0 0 0 0 0 0 0 0 0 0 0 0 0 0 0 0 0 0 0 0 0 0 0 0 0 0 0 0 0 0 0 0 0
0 0 0 0 0 0 0 0 0 0 0 0 0 0 0 0 0 0 0 0 0 0 0 0 0 0 0 0 0 0 0 0 0 0 0 0 0 0 0 0 0 0 0 0 0 0 0 0 0 0 0 0 0 0 0 0 0 1 0 ;
Estimate "Yes1 Slope" Log_SUMMED_ST_1 1Log_SUMMED_ST_1*Protein 0 0 0 0 0 0 0 0 0 0 0 0 0 0 0 0 0 0 0 0 0 0 0 0 0 0 0 0 0 0 0 0 0
0 0 0 0 0 0 0 0 0 0 0 0 0 0 0 0 0 0 0 0 0 0 0 0 0 0 0 0 0 0 0 0 0 0 0 0 0 0 0 0 0 0 0 0 0 0 0 0 0 0 0 0 0 0 0 0 0 0 1 ;

RUN;
```

SI References

Kiernan, K., J. Tao and P. Gibbs (2012). Tips and Strategies for Mixed Modeling with SAS/STAT® Procedures. SAS Global Forum, Florida, SAS Institute Inc., Cary, NC, USA.

Littell, R. C., W. W. Stroup and R. J. Freund (2002). SAS for linear models. Cary, NC, SAS Institute.
